# Identification of mitochondria metabolism-related biomarkers associated with the development of rheumatoid arthritis using bioinformatics: An observational study

**DOI:** 10.1097/MD.0000000000044435

**Published:** 2026-01-09

**Authors:** Ruiyang Xu, Zhixuan Zhang, Min Zhou, Zenan Xu, Jinchen Huang, Ke Gan, Yan Lu

**Affiliations:** a Affiliated Hospital of Nanjing University of Chinese Medicine, Jiangsu Province Academy of Traditional Chinese Medicine, Nanjing, Jiangsu, China; b The Second Affiliated Hospital of Nanjing University of Chinese Medicine, Nanjing, Jiangsu, China.

**Keywords:** immune infiltration landscape, machine learning, mitochondria metabolism, molecular regulatory network, rheumatoid arthritis

## Abstract

Rheumatoid arthritis (RA) is a systemic inflammatory autoimmune disorder that has serious physical and mental health implications. It is evident that disruptions to mitochondrial function have a considerable impact on the survival, activation, and differentiation of RA-associated immune and nonimmune cells. However, the mechanisms of mitochondria metabolism in RA remain unclear. This study identified mitochondrial metabolism-related genes that may contribute to the pathogenesis of RA. The following data were sourced from public databases: transcriptome data of RA and mitochondrial metabolism-related genes. The protein-protein interaction network, machine learning, and gene expression analyses were used to screen the biomarkers. A nomogram was developed to assess the risk of RA. The validity of this nomogram was then tested through the calibration curve and the receiver operating characteristic curve. The biological characteristics and immune infiltration landscape of the biomarkers and RA were then evaluated using functional enrichment and immune infiltration analyses. Ultimately, a molecular regulatory pathway of the biomarkers was constructed. *COX7B*, *NDUFB3*, and *UQCRQ* were identified as the biomarkers. Notably, the ribosome and oxidative phosphorylation were the pathway co-enriched by these 3 biomarkers. The 8 immune cell types showed notable distinctions between RA and control groups. Among them, *COX7B*, *NDUFB3*, and *UQCRQ* were all significantly shown to have an inverse relationship with regulatory T cells (cor < −0.40, *P* < .001). Finally, long noncoding RNA (lncRNA, NEAT1) exerted a regulatory influence on *COX7B*, *NDUFB3*, and *UQCRQ* by regulating hsa-miR-514a-3p, hsa-miR-379-5p, and hsa-miR-144-3p, respectively. This study identified and validated 3 biomarkers (*COX7B*, *NDUFB3*, and *UQCRQ*) associated with RA, which proved a foundation for further research into the relationship between mitochondria metabolism and RA.

## 1. Introduction

Rheumatoid arthritis (RA) is an autoimmune disease. Its main clinical features are symmetrical polyarthritis, chronic synovial inflammation, and progressive joint destruction.^[1]^ RA had a slow onset. In the early stage of the disease, it was mainly manifested as morning stiffness, joint swelling, and pain. With further development, joint deformities might occur and the normal functions of the joints might be lost.^[2]^ Epidemiological surveys showed that the incidence of RA had been continuously increasing in the past few decades. By then, the global incidence rate ranged from 0.4% to 1.3%, with China’s at 0.42%. It was predicted to keep rising. Among RA patients, most were aged between 45 and 60.^[3]^ The disability rate of RA could reach 61.3%.^[4]^ It led to the decline of patients’ physical functions, quality of life, and social participation, and imposed a huge economic burden on families and society. Studies had shown that factors like genetic susceptibility, environmental factors, abnormal immune system, and estrogen-level imbalance were closely associated with the occurrence and progression of RA. However, its exact etiology and pathogenesis remained unclear.^[5]^ Therefore, it is clinically crucial to emphasize early diagnosis and intervention in RA and to reduce its disease burden.

Mitochondria were the sites where carbohydrates, fats, and amino acids were finally oxidized to release energy. They were also important organelles affecting intracellular oxygen supply. They were involved in the tricarboxylic acid cycle and oxidative phosphorylation during aerobic respiration, playing a vital role in sustaining cell functions and homeostasis.^[6]^ As the energy factory of the cell, its oxygen-dependent metabolic processes are crucial for the body to maintain normal vital activities.^[7]^ Mitochondrial metabolic dysfunction has led to the onset of diseases like Alzheimer disease, systemic lupus erythematosus, antiphospholipid syndrome, and RA.^[8,9]^ In RA, mitochondrial reactive oxygen species (mtROS) led to lysosomal permeabilization, depletion, and autophagic vacuole accumulation, thus activating the disease state.^[10]^ Mitochondrial disorder mechanisms in RA mainly involved mitochondrial DNA (mtDNA) mutations, oxidative stress, abnormal energy metabolism, hypoxemia, apoptosis, etc. mtDNA was crucial for normal mitochondrial function. In recent years, the link between mtDNA mutations and RA had drawn wide attention. Relevant studies showed a positive correlation between mtDNA mutations and RA onset.^[11]^ Oxidative stress response was a key link in RA pathogenesis. Mitochondria had been proven to play a prominent role in regulating oxidative stress. Mitochondrial damage resulted in increased reactive oxygen species production, potentially triggering oxidative stress, and inflammation.^[12]^ Mitochondrial metabolic disorders can lead to a shift in cellular metabolism from oxidative phosphorylation to glycolysis. This metabolic reprogramming was common in the inflammatory cells of RA joints. Glycolysis-produced lactic acid and other metabolic products accumulated locally in joints, acidifying the joint microenvironment, and further exacerbating the inflammatory response and joint destruction.^[13–16]^ Given the significance of mitochondrial metabolism in RA pathogenesis, it may become a potential target for treatment. However, the regulatory mechanisms of mitochondrial metabolism-related genes (MMRGs) in RA required further in-depth exploration.

In order to understand the regulatory mechanisms of mitochondrial metabolism in RA in a more systematic and comprehensive manner, this study obtained the candidate genes related to MMRGs in RA based on the transcriptomic data of RA in public databases and the MMRGs in the databases. Additionally, biomarkers were identified through machine learning algorithms and expression validation. Subsequently, the relevant biological pathways, immunological characteristics, and potential regulatory mechanisms of the biomarkers were analyzed, providing new insights for exploring the potential mechanisms of mitochondrial metabolism in RA.

## 2. Results

### 2.1. The 39 candidate genes were associated with mitochondria metabolism and RA

**Figure 1.**
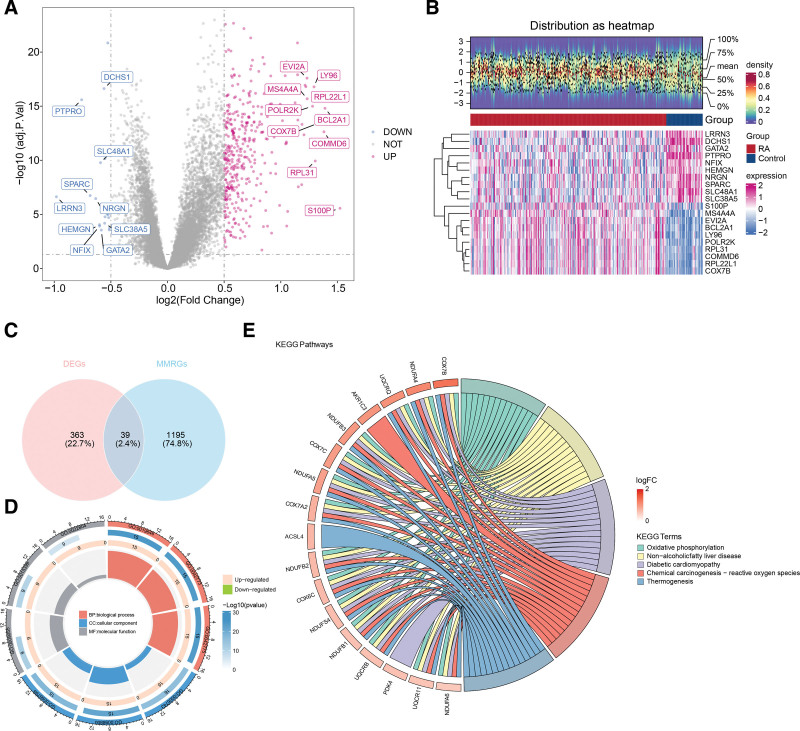
Candidate genes and functional analysis. (A) Volcano plot of differentially expressed genes (DEGs) between RA and control groups. The red dots represent genes that are significantly upregulated in the RA group compared to the control group. The blue dots represent genes that are significantly downregulated in the RA group. (B) Heatmap of the top 10 upregulated and downregulated genes in RA versus control groups. Red indicates higher expression; blue indicates lower expression. (C) Venn diagram showing the intersection of differentially expressed genes (DEGs) and mitochondrial metabolism-related genes (MMRGs). The overlapping region represents the candidate genes shared between the 2 sets. (D) GO enrichment analyses of potential candidate genes. Green for BPs, yellow for CCs, and blue for MFs. (E) KEGG enrichment analyses of potential candidate genes. The bubble chart shows the significantly enriched pathways, with bubble size representing the number of genes involved and color intensity indicating the significance level (*P*-value). BPs = biological processes, CCs = cellular components, GO = Gene Ontology, KEGG = Kyoto Encyclopedia of Genes and Genomes, MFs = molecular functions, RA = rheumatoid arthritis.

First, 402 differentially expressed genes (DEGs) were detected between the RA and control groups, of which 383 were upregulated and 19 were downregulated in the RA group (Fig. 1a, Table S1, Supplemental Digital Content, https://links.lww.com/MD/P915). In addition, a heat map of the top 10 upregulated and downregulated genes showed that these genes could effectively discriminate between RA and control groups (Fig. 1b). To validate the stability of these DEGs identified in the training dataset GSE93272, we applied the same criteria and analytical methods to the validation dataset GSE15573, identifying 134 DEGs (74 upregulated and 60 downregulated in the RA group; Figure S1a, Supplemental Digital Content, https://links.lww.com/MD/P914). The top 10 DEGs in GSE15573 could also clearly distinguish the RA group from the control group (Figure S1b, Supplemental Digital Content, https://links.lww.com/MD/P914), In addition, 18 shared genes between DEGs and MMRGs were identified in GSE15573 (Figure S1c, Supplemental Digital Content, https://links.lww.com/MD/P914) further supporting their potential biological significance. An intersection analysis of DEGs from GSE93272 and GSE15573 identified 43 shared genes (Figure S2a, Supplemental Digital Content, https://links.lww.com/MD/P914), and these genes showed consistent expression trends in both datasets (Figure S2b and c, Supplemental Digital Content, https://links.lww.com/MD/P914), confirming the stability of DEG screening. The training set GSE93272, 39 shared genes were identified between DEGs and MMRGs, and these genes were selected as candidate genes for further investigation (Fig. 1c). A total of 403 Gene Ontology (GO) entries were enriched based on candidate genes, including 309 biological processes (like mitochondrial ATP synthesis-coupled electron transport and ATP synthesis-coupled electron transport), 26 cellular components (like mitochondrial inner membrane protein complex and mitochondrial inner membrane), and 68 molecular functions (such as primary active transmembrane transporter activity and electron transfer activity) (Fig. 1d, Table S2, Supplemental Digital Content, https://links.lww.com/MD/P915). The results of the Kyoto Encyclopedia of Genes and Genomes (KEGG) analysis revealed that the candidate genes were significantly implicated in 21 different pathways, mainly related to oxidative phosphorylation, chemical carcinogenesis, reactive oxygen species, and related processes (Fig. 1e, Table S3, Supplemental Digital Content, https://links.lww.com/MD/P915).^[17–19]^

### 2.2. The cytochrome c oxidase subunit 7b (COX7B), NADH dehydrogenase [ubiquinone] 1 beta subcomplex subunit 3 (NDUFB3), and ubiquinol-cytochrome c reductase subunit VII (UQCRQ) were identified as biomarkers

**Figure 2.**
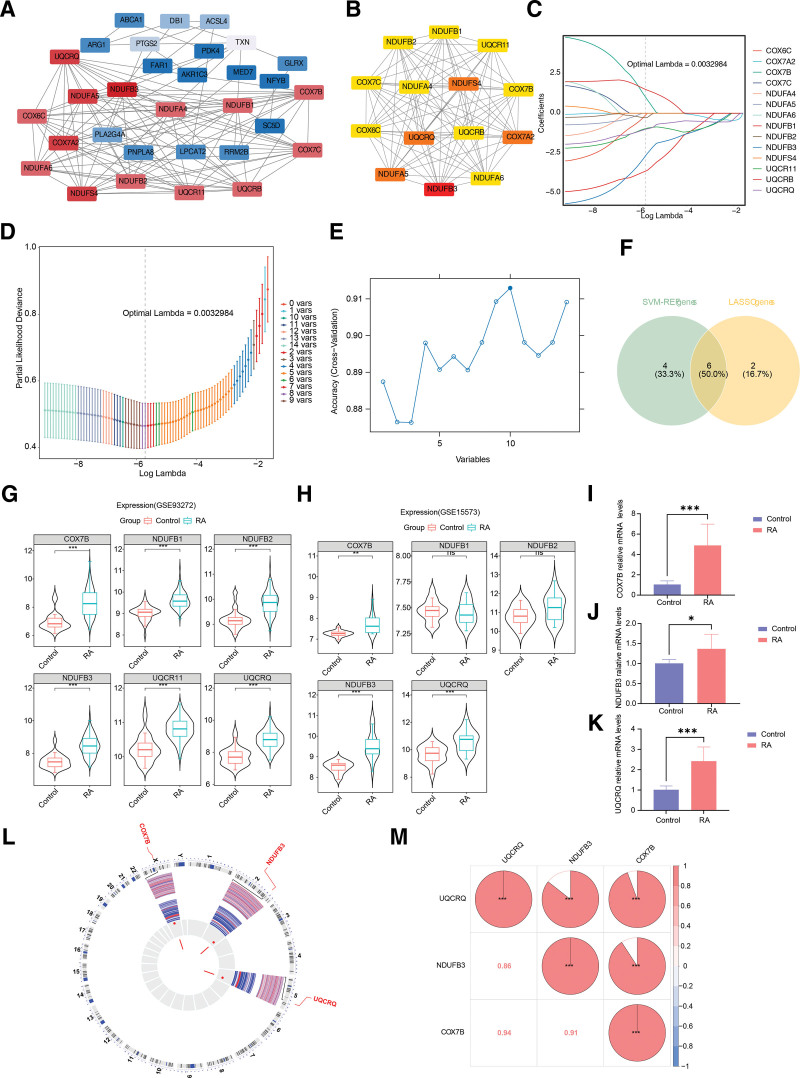
Identification and validation of biomarkers associated with rheumatoid arthritis. (A) PPI network of the candidate genes. (B) The top 14 genes in Degree score were selected as candidate biomarkers. (C and D) Candidate biomarkers screened by the LASSO algorithm. (E) Candidate biomarkers screened by the SVM-RFE algorithm. (F) Candidate key biomarkers identified by both LASSO and SVM-RFE algorithms. (G and H) Differential expression of *COX7B*, *NDUFB3*, and *UQCRQ* in RA versus control groups in GSE93272 and GSE15573. Box plots show expression levels with median (center line) and IQR (box). Colors indicate expression levels, with red for higher and blue for lower. Asterisks (*) denote significance: *P < .05; **P < .01; ***P < .001. (I–K) RT-qPCR validation of upregulated expression of *COX7B*, *NDUFB3*, and *UQCRQ* in RA. Blue represents the control group, and red represents the RA group.The asterisks (*) indicate the level of statistical significance: *P < .05, **P < .01, and ***P < .001, highlighting the significant upregulation of these genes in RA patients. (L) Chromosomal localization of biomarkers. (M) Correlation analysis among biomarkers. Blue color indicates a negative correlation, and red color indicates a positive correlation. IQR = interquartile range, LASSO = least absolute shrinkage and selection operator, PPI = protein-protein interaction, RA = rheumatoid arthritis, RT-qPCR = real-time quantitative polymerase chain reaction, SVM-RFE = support vector machine-recursive feature elimination.

Protein-protein interaction (PPI) network analysis revealed 31 PPI relationships among the candidate genes in the training set, such as interactions between UQCRQ and NDUFB3, and between COX7A2 and NDUFS4 (Fig. 2a), showing the characteristic of multi-network synergistic interactions. Subsequently, the top 14 genes in Degree score were selected as candidate biomarkers among 31 genes (Fig. 2b). From the network, it was determined that NDUFB3 was involved in interactions with numerous other proteins, including NDUFA5, COX7A2, and NDUFB1, among others. To further verify the stability of the interaction relationships, a PPI network analysis was performed on the 18 candidate genes (the intersection of DEGs and mitochondria-related genes) in GSE15573, identifying 12 core interacting genes (Figure S3a, Supplemental Digital Content, https://links.lww.com/MD/P914). Among them, key interactions such as NDUFB3 with UQCRQ and COX7A2 with NDUFS5 were consistent with those in the training set. The top 10 core genes based on Degree scores were selected using the cytoHubba plugin, and NDUFB3, like in the training set, was the gene with the most interactions (Figure S3b, Supplemental Digital Content, https://links.lww.com/MD/P914). This confirmed the stability of the core interaction relationships and validated the reliability of the core network.

For the result of the least absolute shrinkage and selection operator (LASSO) algorithm, when the minimum lambda value was 0.0032984, a total of 8 LASSO genes were screened among the training set candidate biomarkers, namely UQCR11, NDUFB2, NDUFB1, COX6C, UQCRQ, NDUFB3, COX7B and UQCRB (Fig. 2c and d). The support vector machine-recursive feature elimination (SVM-RFE) algorithm revealed that the optimal prediction accuracy was achieved with 10 genes selected (Fig. 2e). This configuration allowed for the selection of 10 SVM-RFE genes from the identified biomarker candidates, namely NDUFB3, UQCRQ, NDUFB1, COX7A2, NDUFA4, COX7B, NDUFB2, UQCR11, COX7C, and NDUFA6. The subsequent identification of UQCR11, NDUFB2, NDUFB1, UQCRQ, NDUFB3, and COX7B as candidate key biomarkers by both the LASSO and SVM-RFE algorithms was a significant finding (Fig. 2f). According to the data in the GSE93272 and GSE15573 datasets, COX7B, NDUFB3, and UQCRQ were found to be considerably upregulated in the RA group (*P* < .01), with consistent expression trends, and these genes were identified as biomarkers (Fig. 2g and h). The RT-qPCR findings indicated that COX7B, NDUFB3, and UQCRQ were strongly expressed in the RA group (*P* < .05) (Fig. 2i–k).

Interestingly, COX7B, NDUFB3, and UQCRQ were localized on chromosomes X, 2, and 5, respectively (Fig. 2l). The association between the biomarkers was examined and the outcomes demonstrated that all genes were statistically significant (cor > 0.80, *P* < .001) (Fig. 2m). The most significant positive association was observed between COX7B and UQCRQ (cor = 0.94, *P* < .001). The locational information and correlation of biomarkers facilitated a deeper understanding not only of the distinct impacts on RA, but also provided a novel perspective for exploring their functional associations.

### 2.3. The nomogram model was found to be a more effective tool for predicting RA

**Figure 3.**
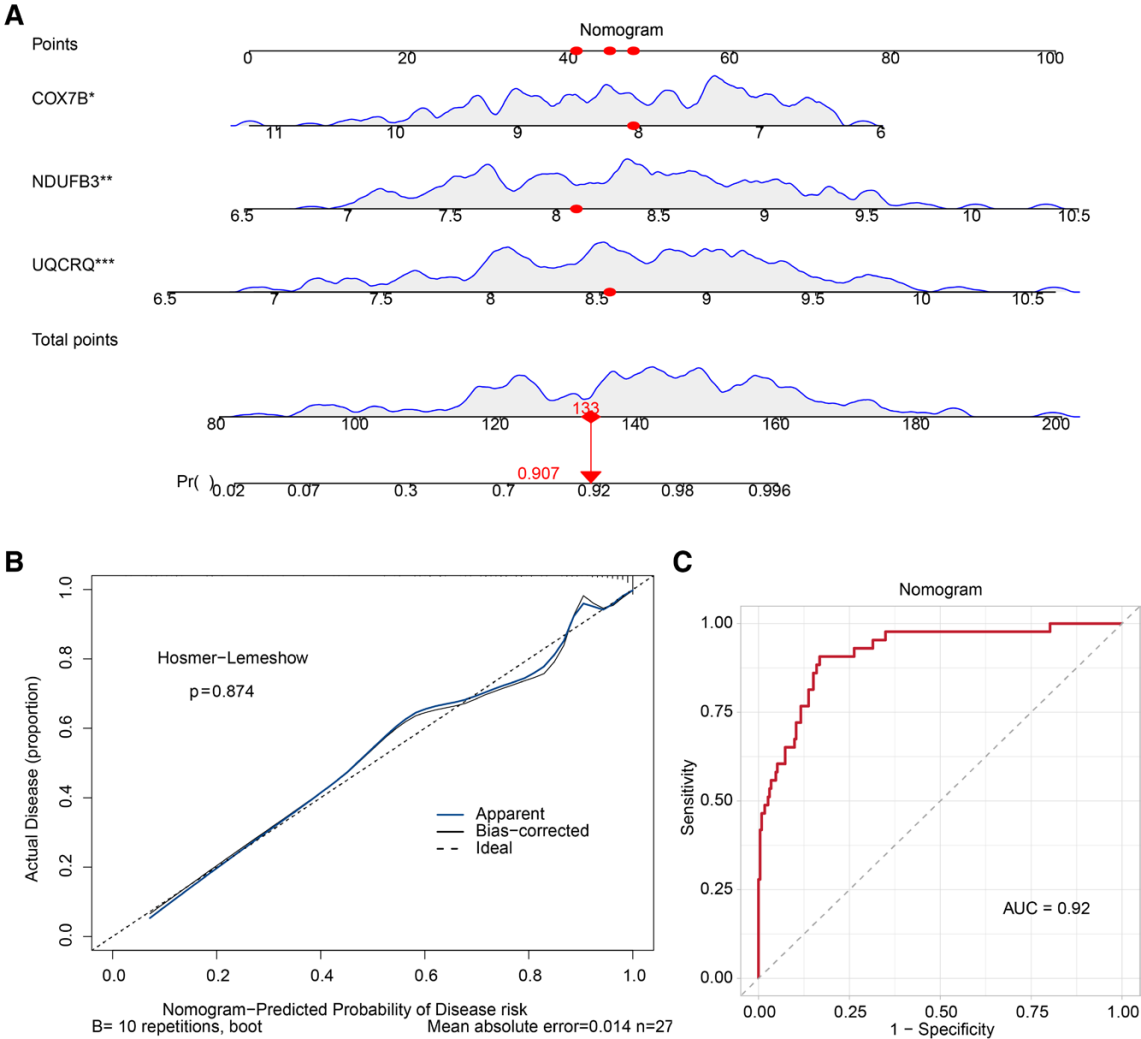
RA risk prediction nomogram and accuracy assessment. (A) Construction of the RA risk prediction nomogram. RA risk prediction nomogram based on the identified biomarkers (COX7B, NDUFB3, and UQCRQ). The red dots on the nomogram represent the contribution of each biomarker to the overall risk score. (B) Calibration curve for assessing nomogram accuracy. The blue line represents the calibration of the nomogram model, with closer alignment to the diagonal line indicating higher accuracy. The calibration curve demonstrates the agreement between the predicted and actual outcomes. (C) ROC curve for evaluating nomogram predictive performance. The area under the curve (AUC) is used as a measure of predictive accuracy, with an AUC of 0.92 indicating high predictive performance. RA = rheumatoid arthritis, ROC = receiver operating characteristic.

A nomogram was constructed for the purpose of predicting the risk of RA using the biomarkers (COX7B, NDUFB3, and UQCRQ) (Fig. 3a). In order to appraise the capacity of the nomogram model for prediction, calibration curves and receiver operating characteristic (ROC) curves were utilized. It was evident from the calibration curve (*P* = .874) that there was a minimal discrepancy between the actual and predicted risks of RA (Fig. 3b). Moreover, the nomogram demonstrated an area under the curve (AUC) value of 0.92, indicating a high degree of accuracy (Fig. 3c). These results demonstrated that the nomogram exhibited a high degree of predictive accuracy with regard to the progression of RA. For validation, a prediction model based on the same biomarkers was built in GSE15573 (Figure S4a, Supplemental Digital Content, https://links.lww.com/MD/P914). The calibration curve showed good fit between actual risk and predicted values (*P* = .586) (Figure S4b, Supplemental Digital Content, https://links.lww.com/MD/P914), with the ROC curve yielding an AUC of 0.95 (Figure S4c, Supplemental Digital Content, https://links.lww.com/MD/P914), close to the training set performance. This confirmed the nomogram’s high predictive accuracy in the independent dataset, highlighting the model’s reliability.

### 2.4. Biomarkers were involved in multiple signaling pathways

**Figure 4.**
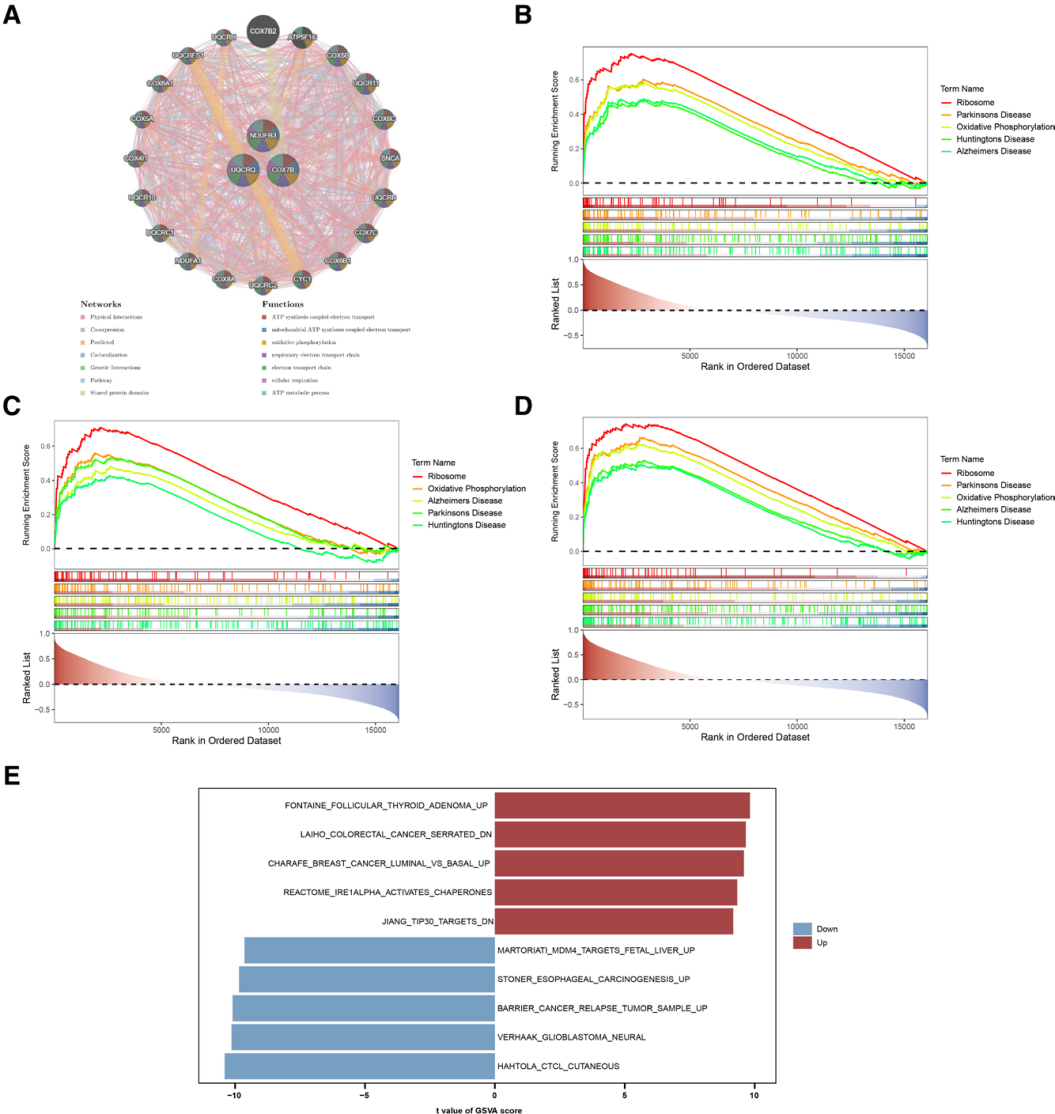
Pathways and functional enrichment analysis involving biomarkers. (A) GeneMANIA network showing interactions and functions of the identified biomarkers (COX7B, NDUFB3, and UQCRQ). Colors indicate interaction types: red for co-expression, blue for predicted interactions, and green for shared domains. (B–D) GSEA analysis of pathways enriched by COX7B, NDUFB3, and UQCRQ expression, respectively. Red indicates positive enrichment, and blue indicates negative enrichment. (E) GSVA analysis comparing pathway enrichment between RA and control groups. Red represents higher pathway activity in RA, and blue represents lower activity. GSEA = gene set enrichment analysis, GSVA = gene set variation analysis, RA = rheumatoid arthritis.

In the GeneMANIA network, biomarkers interacted with 20 genes, including UQCR11, COX5B, and UQCRC1, and were involved in functions like respiratory electron transport, mitochondrial ATP synthesis, oxidative phosphorylation, and ATP metabolism (Fig. 4a).

Subsequently, gene set enrichment analysis (GSEA) analysis observed that pathways associated with expression of COX7B, NDUFB3, and UQCRQ were 44, 7, and 55, respectively (Table S4, Supplemental Digital Content, https://links.lww.com/MD/P915). In addition, COX7B, NDUFB3, and UQCRQ were both primarily enriched in some pathways, such as ribosome, Huntington disease, oxidative phosphorylation, Alzheimer disease, and Parkinson disease (Fig. 4b–d).

To investigate more deeply into the functional differences between the RA and control groups, we carried out a gene set variation analysis (GSVA) analysis. Pathway enrichment analysis by GSVA revealed significant increase of fontaine follicular thyroid adenoma up, laiho colorectal cancer serrated dn, charafe breast cancer luminal vs basal up, and other signaling pathways in RA group, while hahtola ctcl cutaneous, verhaak glioblastoma neural, barrier cancer relapse tumor sample up, and other signaling pathways were significantly enhanced in control group (Fig. 4e, Table S5, Supplemental Digital Content, https://links.lww.com/MD/P915).

### 2.5. A strong correlation was demonstrated between biomarkers and immune cells in RA

**Figure 5.**
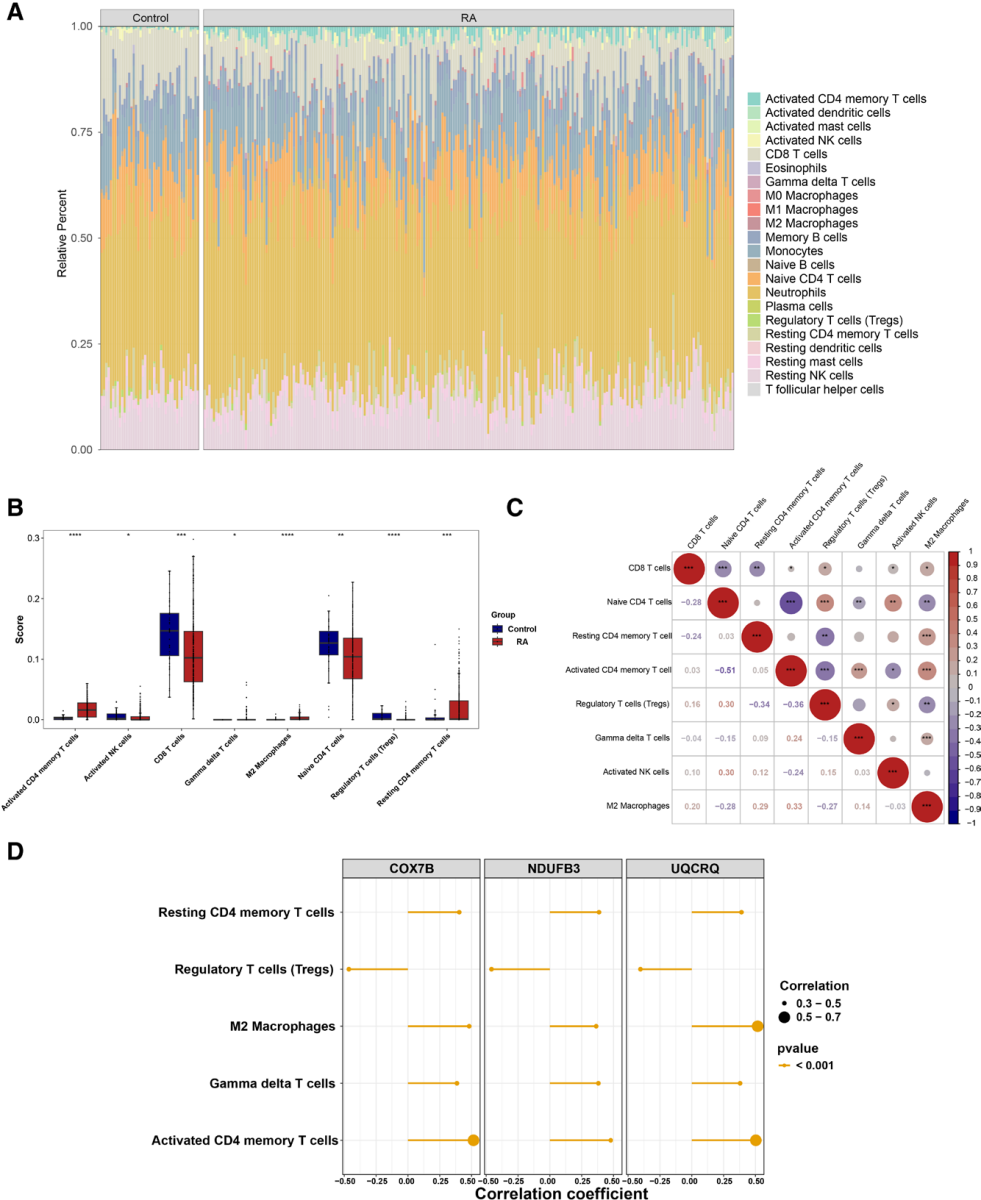
Immune cell infiltration patterns and biomarker correlations in rheumatoid arthritis (RA). (A) Heatmap showing the infiltration levels of 22 immune cell types in RA and control samples. Red representing higher infiltration, and blue representing lower infiltration. (B) Box plot highlighting significant differences in immune cell infiltration between RA and control groups. Asterisks (*) denote statistical significance: *P < .05, **P < .01, ***P < .001, and ****P < .0001. (C) Correlation analysis among differentially infiltrated immune cells. Colors in the correlation matrix represent the strength and direction of the correlation: red for positive correlation and blue for negative correlation, with intensity indicating the magnitude. (D) Correlation between identified biomarkers (COX7B, NDUFB3, UQCRQ) and immune cells.

In the present study, the CIBERSORT algorithm was used to evaluate and quantify the infiltration abundance of 22 different immune cells in 2 designated groups: the RA group and the control group. As shown in Figure 5a, a graphical representation of the percentage infiltration abundance of each immune cell across the entire sample set was generated. The box plot demonstrated that compared to the control group, the levels of CD8 T cells, naive CD4 T cells, regulatory T cells (Tregs), and activated natural killer cells were significantly reduced in the RA group, while resting CD4 memory T cells, activated CD4 memory T cells, gamma delta T cells, and M2 macrophages showed a high level of abundance (*P* < .05) (Fig. 5b, Table S6, Supplemental Digital Content, https://links.lww.com/MD/P915). The strongest positive correlation was found between activated CD4 memory T cells and M2 macrophages (cor = 0.33, *P* < .001) and the most pronounced negative correlation was found between activated CD4 memory T cells and naive CD4 T cells (cor = −0.51, *P* < .001) (Fig. 5c). Moreover, correlation analysis showed that the biomarkers were all significantly associated with 5 different immune cells, including resting CD4 memory T cells, Tregs, M2 macrophages, gamma delta T cells, and activated CD4 memory T cells (|cor| > 0.30, *P* < .001) (Fig. 5d, Table S7, Supplemental Digital Content, https://links.lww.com/MD/P915). Among these, COX7B, NDUFB3, and UQCRQ were all significantly inversely correlated with Tregs (cor < −0.40, *P* < .001), and UQCRQ was significantly positively associated with M2 macrophages (cor > 0.50, *P* < .001).

### 2.6. Interaction networks were identified between biomarkers and transcription factors (TFs), long noncoding RNAs (lncRNAs), microRNAs (miRNAs), drugs, and diseases

**Figure 6.**
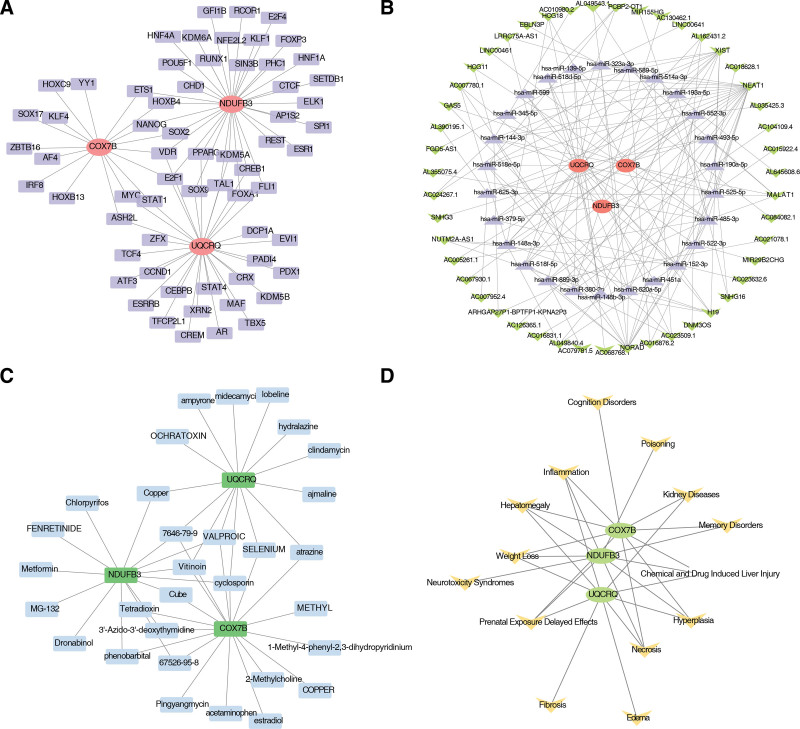
Interaction networks and functional predictions of biomarkers in RA. (A) Transcription factor (TF)-mRNA regulatory network of the identified biomarkers (COX7B, NDUFB3, and UQCRQ). The network shows regulatory interactions between TFs and the biomarkers. Colors indicate the type of interaction: red for positive regulation and blue for negative regulation. (B) Long noncoding RNA (lncRNA)-microRNA (miRNA)-mRNA regulatory network. Green for lncRNAs, yellow for miRNAs, and blue for mRNAs. (C) Potential drug targets for biomarkers in RA treatment. Purple for drugs and blue for biomarkers. (D) Diseases associated with biomarkers identified by Comparative Toxicogenomics Database (CTD) analysis. Orange for diseases and blue for biomarkers.

In the TF-mRNA network, COX7B was regulated by 17 TFs, NDUFB3 was regulated by 34 TFs, and UQCRQ was regulated by 31 TFs (Fig. 6a). Additionally, COX7B, NDUFB3, and UQCRQ were regulated by VDR and E2F1. Subsequently, COX7B, NDUFB3, and UQCRQ were found to be modulated by 3, 12, and 62 miRNAs, respectively (Table S8, Supplemental Digital Content, https://links.lww.com/MD/P915). The lncRNA-miRNA-mRNA regulatory network then contained 76 nodes, including 3 mRNAs, 26 miRNAs, and 47 lncRNAs, connected by a total of 146 edges (Fig. 6b). In detail, lncRNA (NEAT1) exerted a regulatory influence on COX7B, NDUFB3, and UQCRQ by regulating hsa-miR-514a-3p, hsa-miR-379-5p, and hsa-miR-144-3p, respectively.

Potential medications of biomarkers for RA were obtained according to the DSigDB database as shown in Figure 6c. These included 18 drugs that targeted COX7B, 14 drugs that targeted NDUFB3, and 14 drugs that targeted UQCRQ. Among these, the vitinoin, 7646-79-9, cyclosporin, and valproic drug were associated to 3 biomarkers. Based on Comparative Toxicogenomics Database database, the top 10 scoring diseases for each biomarker were selected for mapping (Fig. 6d). The following diseases had the strongest coordination with the 3 biomarkers studied: weight loss, prenatal exposure delayed effects, chemical and drug induced liver injury, inflammation, necrosis, hepatomegaly, and hyperplasia.

## 3. Discussion

This study, leveraging DEGs identified from RA transcriptomic data, overcomes the limitations of current RA research which predominantly relies on single-omics data analysis (e.g., transcriptomics or PPI networks) or conventional biostatistical methods for biomarker screening.^[20,21]^ By integrating transcriptomics, PPI networks, and machine learning algorithms, we identified key MMRG-associated biomarkers (COX7B, NDUFB3, UQCRQ). Through in-depth analysis of the interactions between these genes and the immune microenvironment, we constructed an lncRNA-miRNA-mRNA regulatory network to reveal the impact of epigenetic regulation on mitochondrial function. Furthermore, we developed a molecular signature-based predictive model. This work offers valuable insights for the early diagnosis of RA and provides a deeper understanding of the role of MMRGs in RA pathogenesis, particularly from a “metabolism-immune” regulation perspective. These innovative findings establish a crucial theoretical foundation for a deeper understanding of RA pathogenesis and subsequent clinical translational research, while also offering a new scientific reference for related studies.

COX7B, or cytochrome c oxidase subunit 7b, is a nuclear-encoded subunit of cytochrome c oxidase (COX) and is involved in autoimmune responses and cell activation.^[22]^ COX is the terminal enzyme of the mitochondrial respiratory chain and has a complex structure. Its currently known function is relatively simple, that is, to transfer electrons during the process of cellular aerobic respiration and generate ATP for the use of cells.^[23,24]^ Dysfunction of COX or impairment of electron transport may lead to the accumulation of electrons within the mitochondrial intermembrane space, consequently promoting the generation of mtROS.^[25]^ Both COX dysfunction and imbalances in mtROS levels can result in hyperactivation or dysfunction of immune cells, thereby triggering the development of immune-related disorders.^[26]^ In vertebrates, COX7B was crucial for central nervous system development. Its normal function contributed to nervous system formation and maintenance. COX7B interacted with COX-complex proteins to complete cytochrome c oxidase assembly and regulation, ensuring efficient mitochondrial respiratory chain operation.^[27–29]^ Prior research has shown that COX7B is strongly associated with disorders such as Alzheimer disease, psoriatic arthritis, and ankylosing spondylitis.^[30,31]^ In this study, COX7B, identified as a subunit of COX, was found to be significantly upregulated in RA. This upregulation may enhance the efficiency of electron transport within the mitochondrial respiratory chain, potentially leading to increased electron leakage during oxidative phosphorylation and consequently promoting the generation of mtROS. KEGG pathway analysis further revealed that COX7B is enriched in the “Oxidative phosphorylation” pathway, suggesting that its aberrant expression could disrupt mitochondrial redox homeostasis, thereby amplifying inflammatory signaling.

NDUFB3, namely subunit 3 of the NADH dehydrogenase [ubiquinone] 1 beta subcomplex. The protein encoded by NDUFB3 is part of the NADH dehydrogenase complex I.^[21]^ Complex I was the first enzyme complex in the mitochondrial respiratory chain and was vital for transferring electrons from NADH to ubiquinone. As a subunit of complex I, NDUFB3 was crucial in oxidative phosphorylation. It started the electron transport chain, promoted proton transfer, and provided energy for ATP synthesis. Also, it could induce NLRP3 activation and pyroptosis.^[32]^ In RA, NDUFB3 showed high expression in plasma cells and was closely connected with the onset and progression of the disease.^[30,33]^ Functioning as a subunit of ubiquinol-cytochrome c reductase (complex III), this protein was found to be upregulated in RA. Its overexpression may enhance the proton-pumping function of complex III, thereby exacerbating dysregulation of the mitochondrial membrane potential,^[34]^ promoting mtROS generation, and activating apoptotic signaling.

UQCRQ was subunit VII of ubiquinol-cytochrome c reductase complex III. The protein encoded by the gene was part of the mitochondrial respiratory chain complex III (cytochrome bc1 complex). UQCRQ played a key role in cellular respiration. The mitochondrial respiratory chain, an energy-generating pathway in cells, consisted of multiple protein complexes. Complex III, where UQCRQ was located, transferred electrons from ubiquinol to cytochrome c and aided in generating the electrochemical proton gradient.^[20]^ Given the critical role of UQCRQ in the respiratory chain, its mutations or abnormal expressions were closely associated with mitochondrial diseases. Mitochondrial diseases, a group of disorders that stemmed from mitochondrial dysfunction (including Parkinson disease, Huntington disease, Alzheimer disease, systemic lupus erythematosus, ankylosing spondylitis, etc), could impact multiple systems.^[35–39]^ When there were problems with UQCRQ, it might have led to disorders in energy metabolism and then triggered a series of symptoms, such as muscle weakness, exercise intolerance, and neurological abnormalities.^[34,39,40]^ As a subunit of ubiquinone-cytochrome c reductase (complex III), it is upregulated in RA. Its overexpression may enhance the proton pump function of complex III, aggravate mitochondrial membrane potential imbalance,^[34]^ and promote mtROS production and activation of apoptosis signals.

This study found that COX7B, NDUFB3, and UQCRQ were correlated with several immune cells using immune infiltration analysis. In RA patients, resting CD4 memory T cells may have driven disease chronicity and relapse through antigen memory.^[41]^ For example, in the joint microenvironment, pathogen antigens like self-antigens or damaged-tissue-released fragments could activate resting CD4 memory T cells. Once activated, these cells secreted cytokines such as IFN-γ and interleukin-2 (IL-2), and could also indirectly prompt synovial cells to secrete MMPs and other inflammatory mediators, causing joint cartilage and bone destruction.^[42,43]^ Regulatory T cells, a subset of T cells, had immunosuppressive functions. They were vital for self-immunological tolerance and immune homeostasis, suppressing over-activation of other immune cells. Under normal conditions, they maintained immune tolerance to self-tissues. However, in RA patients, their functions and numbers could be abnormal. Studies found that in some RA patients, regulatory T cells in peripheral blood and joint synovial tissues decreased. This might have lowered immune tolerance to joint-related antigens, over-activated autoreactive T and B cells, triggered inflammation, and contributed to RA onset.^[44,45]^

M2 macrophages are a subset of macrophages and belong to immune cells with anti-inflammatory and tissue repair functions.^[46]^ They have a essential impact on the later stages of the immune response and promote tissue repair and restoration of homeostasis. M2 macrophages had a positive role in the tissue repair process.^[47]^ They could promote angiogenesis and provide nutrients and oxygen for damaged tissues, which had potential benefits for alleviating local tissue damage in the joints of patients with RA. However, under certain circumstances, M2 macrophages might lead to fibrosis. In the joints of RA patients, an excessive M2 macrophage response may promote the proliferation of fibrous tissues, resulting in joint stiffness and limited mobility.^[48]^

γδT cells are a special subset of T lymphocytes. Their T cell receptors consist of γ chains and δ chains, rather than α chains and β chains as in traditional αβT cells. γδT cells are crucial in the local immune microenvironment of the joints. They could recognize abnormal cells or antigens in joint tissues, such as damaged synovial cells or changes in extracellular matrix components. This recognition will trigger the activation of γδT cells and local inflammatory responses. Moreover, the continuous activation of γδT cells may change the joint microenvironment, making it more conducive to the infiltration of inflammatory cells and the accumulation of inflammatory mediators.^[49,50]^

Activated CD4 memory T cells are a subset of memory cells that differentiate from CD4+ T cells after the body’s primary immune response. They possess a memory function and can be rapidly activated upon reexposure to the same antigen, initiating a rapid and intense immune response. Like resting CD4 memory T cells, activated CD4 memory T cells serve a crucial function in the chronicity and relapse of disease.^[41,51]^

Based on this, it can be speculated that the biomarkers COX7B, NDUFB3, and UQCRQ may further affect the behaviors or related functions of these immune cells and regulate the immune microenvironment in RA. The pathways that the above 3 biomarkers were commonly enriched in include the ribosome pathway and the oxidative phosphorylation pathway. Abnormalities in the ribosome pathway can lead to the dysregulation of the synthesis of certain proteins. For example, abnormal ribosome functions could cause over-synthesis of joint-related self-antigens or production of abnormal proteins. The immune system recognized these as foreign, triggering an autoimmune response. Citrullinated proteins, key in RA pathogenesis, might have had their synthesis affected by the ribosome pathway. Excessive production could prompt immune cell attacks. The activity of the ribosome pathway also provided a material basis for the synthesis of inflammatory mediators. Cells use ribosomes to synthesize proteins related to various inflammatory factors like tumor necrosis factor-alpha, IL-1beta, and IL-6. In the synovial cells and immune cells of RA patients, the enhanced ribosome pathway may lead to massive synthesis of these inflammatory mediators, which then continuously stimulate the synovial tissue, causing synovial inflammation, hyperplasia, and joint destruction.^[52–55]^

The process of oxidative phosphorylation may also be disturbed in synovial cells and immune cells of people with RA. Changes in energy metabolism will affect the functions of cells. For example, insufficient energy supply to synovial cells may lead to abnormal functions and increase the release of inflammatory mediators. Meanwhile, under stress conditions such as hypoxia, inflammatory cells shift from oxidative phosphorylation to glycolysis, and the metabolic products such as lactic acid will further acidify the joint microenvironment and aggravate the inflammatory response. In T cells, oxidative phosphorylation provides energy support for their activation, proliferation, and cytokine secretion. Abnormal oxidative phosphorylation can impair energy supply to T cells, hindering their proliferation and differentiation. Alternatively, it may cause metabolic reprogramming, increasing their reliance on glycolysis, which alters their cytokine secretion profile and intensifies the inflammatory response.^[56–58]^

This study, along with previous ones, confirms that mitochondrial metabolic abnormalities and immune cell imbalance are associated with RA.^[14,59]^ Through cross-validation of multiple algorithms, a multigene combination of COX7B, NDUFB3, and UQCRQ was screened out, the lncRNA NEAT1/miRNA regulatory axis was revealed, and pathways shared with neurodegenerative diseases were discovered, forming differences by virtue of the systematicness of the analysis strategy and the integration of tools. Mechanistically, these markers affect the RA microenvironment by regulating the balance of Tregs/M2 macrophages and metabolic reprogramming. The combined diagnostic model shows promising potential when compared to traditional markers, but multicenter validation and solving tissue specificity issues are required. As therapeutic targets, intervention can be carried out by regulating mitochondrial metabolism or the NEAT1/miRNA axis. These findings expand the understanding of the pathogenesis of RA and provide multilevel innovative diagnostic and therapeutic pathways. The study applied bioinformatics and machine learning methods to screen and validate key biomarkers of RA, developed an exploratory model with promising predictive performance, and explored their functions in the immune microenvironment and signaling pathways, offering new insights and potential targets for the clinical diagnosis and treatment of RA., but there are still limitations such as the need for multicenter validation in clinical application. First, the data were derived from public databases, which may have issues such as insufficient sample size, large sample heterogeneity, and lack of detailed clinical information. Second, although various bioinformatics tools and machine learning algorithms were used, these methods are mainly based on statistical principles and may not fully capture complex biological processes. Moreover, the clinical application of the risk prediction model still needs to be validated. In addition, this study lacks in-depth validation of the functions of biomarkers. Future research should deeply explore the functions of these biomarkers through in vitro cell experiments and in vivo animal models, integrate multi-omics data to more comprehensively understand the pathogenesis of RA, and expand the scope of clinical samples to verify the clinical application value of biomarkers. Meanwhile, developing more advanced bioinformatics methods, such as deep learning algorithms, will help improve the accuracy and efficiency of biomarker screening.

## 4. Materials and methods

### 4.1. Data source

The data were extracted from the Gene Expression Omnibus database (https://www.ncbi.nlm.nih.gov/geo/). The GSE93272 dataset (Sequencing platform: GPL570) was employed as a training set, which included 232 RA blood samples and 43 control blood samples. The GSE15573 dataset (Sequencing platform: GPL6102) served as a validation set, which comprised 18 RA peripheral mononuclear blood cells samples and 15 control peripheral mononuclear blood cells samples. In addition, the study pinpointed 1234 MMRGs from the Molecular Signatures Database (MSigDB, https://www.gsea-msigdb.org/gsea/msigdb/) (Table S9, Supplemental Digital Content, https://links.lww.com/MD/P915).

### 4.2. Differential expression analysis

Characterizing the differential expression of genes (DEGs) were screened from the transcriptome data of RA patients and the control group to provide a list of candidate genes for subsequent analysis. DEGs in the training set, the limma package (v 3.54.0) was used to perform differential expression analysis on RA and control groups (|log2 fold change (FC)| > 0.5, adj. *P* < .05).^[60]^ In addition, A DEGs volcano diagram was constructed with the ggplot2 package (v 3.4.1), and the top 10 genes (sorted by |log2 FC| from high to low) with significant upregulation and downregulation differences were labeled, and then the heat map of these genes was plotted using the pheatmap package (v 1.0.12) (https://CRAN.R-project.org/package=pheatmap).^[61]^ To verify the stability of the DEGs identified in the training set GSE93272, we applied the same screening criteria and analytical methods to the validation set GSE15573. In the validation set (GSE15573), differential expression analysis was also performed with the thresholds of |log2 FC| > 0.5 and adjusted *P* < .05. A volcano plot of DEGs was generated for this dataset, with the top 10 genes showing the most significant upregulation and downregulation (sorted by |log2 FC| from high to low) labeled. Additionally, a heatmap of these genes was plotted to display their expression difference patterns between the RA group and the control group.

### 4.3. Identification and functional enrichment of candidate genes

Screened the intersection genes of DEGs and MMRGs in RA, and analyzed their functions and pathway enrichment through GO and KEGG to reveal their potential role in RA. The ggvenn package (v 0.1.9) (https://CRAN.R-project.org/package=ggvenn) was employed to determine the intersection genes between DEGs and MMRGs. GO and KEGG analyses were then performed to determine the potential biological functions and mechanisms of the candidate genes using the clusterProfiler package (v 4.7.1.3) (*P* < .05).^[62]^

### 4.4. PPI network analysis

First, construct PPI networks for candidate genes in the training and validation sets, and screen key node genes and core genes to narrow down the range of biomarkers. The candidate genes were input to the Search Tool for the Retrieval of Interacting Genes/Proteins database (https://string-db.org/) at a confidence score of 0.4. A PPI network was generated and visualized for the candidate genes by Cytoscape software (v 3.9.1, The Cytoscape Consortium, San Diego), with discrete genes removed.^[63]^ In addition, the PPI network was evaluated using the CytoHubba plug-in in Cytoscape software (v 3.9.1). In addition, evaluate the number of interactions (Degree) between the proteins encoded by the genes and other proteins in the PPI network through the CytoHubba plugin in Cytoscape software (v 3.9.1). Genes with higher Degree scores are often core nodes in the network, participating in the regulation of key biological pathways through interactions with multiple genes and playing a more important role in disease mechanisms.

### 4.5. Machine learning algorithms and expression validation

The LASSO and SVM-RFE algorithms were used to screen RA-related genes, take the intersections, and verify their expression differences to determine effective biomarkers. An SVM-RFE of the candidate biomarkers was performed with the caret package (v 6.0.93) (https://CRAN.R-project.org/package=caret), which screened the SVM-RFE genes based on model prediction accuracy. In addition, the glmnet package (v 4.1.4) was used to implement LASSO regression analysis of candidate biomarkers, and the ideal lambda value was selected through 10-fold cross-validation to screen for LASSO genes.^[64]^ Furthermore, this experiment utilized the Venn diagram package (v 1.7.3) (https://CRAN.R-project.org/package=VennDiagram) to obtain the intersection genes between LASSO genes and SVM-RFE genes, defined as candidate key biomarkers.

The study also compared the expression of key candidate biomarkers in the RA and control groups in the GSE93272 and GSE15573 datasets using the Wilcoxon test (*P* < .05) in the rstatix package (v 0.7.2) (https://github.com/kassambara/rstatix/issues), with results visualized through the ggplot2 package (v 3.4.1). Genes exhibiting consistent expression trends and significant intergroup differences in both datasets were identified as biomarkers. The biomarkers were then evaluated via pROC package (v 1.18.0) to plot ROC curves, assessing their ability to differentiate between RA and control samples. The AUC values were calculated (AUC > 0.7).

### 4.6. Chromosome localization, correlation, and GeneMANIA network analyses of biomarkers

Through chromosome localization, correlation analysis, and GeneMANIA network construction, the genetic background, synergistic mechanism, and potential biological functions of biomarkers were explored. In this study, the RCircos package (v 1.2.2) was applied to determine the chromosomal localization of biomarkers.^[65]^ Spearman correlation analysis was performed between the biomarkers in all samples of the training set, with the correlation matrix plotted using the psych package (v 2.2.9) (https://CRAN.R-project.org/package=psychversion=2.2.9) (|correlation (cor)| > 0.3, *P* < .05). The GeneMANIA platform (http://www.genemania.org/) was used to build a gene co-expression network to further predict the interactions between biomarkers and their involved biological functions.

### 4.7. Development of a nomogram model

A nomogram model was constructed to predict the risk of RA onset, and its accuracy was verified by calibration curves and ROC curves. Both the training set and the validation set used the rms package (v 6.5.0) (https://CRAN.R-project.org/package=rms) to develop a biomarker-based nomogram for determining the probability of RA onset, and the package was also used to draw calibration curves (with *P* > .05 indicating accuracy). The calibration curve was used to determine the accuracy of the nomogram using the rms package (v 6.5.0) (*P* > .05). The ROC curve served to evaluate the predictive efficacy of the nomogram in a clinical setting, using a diagnostic effect of an AUC exceeding 0.7 as a valuable reference point. This analysis was performed by employing the pROC package (v 1.18.0).^[66]^

### 4.8. GSEA and GSVA

The functional enrichment and pathway activity differences of biomarker-related gene sets in RA were analyzed using GSEA and GSVA to reveal the specific pathway changes of RA. This research executed GSEA with the aim of elucidating the biological functions of the biomarkers during RA progression. First, the Spearman correlation coefficients between each biomarker and all other genes in all samples of the training set were calculated by the means of psych package (v 2.2.9), and all sorted genes were ranked from large to small based on the Spearman correlation coefficient and used as the tested gene set. Meanwhile, the reference gene set used was “c2.cp.kegg.v7.4.symbols.gmt” from the MSigDB database. GSEA was then conducted using the clusterProfiler package (v 4.7.1.3), with a criterion of *P* < .05 and |normalized enrichment score| > 1.^[62]^

GSVA analysis was leveraged to elucidate the discrepancies in enrichment pathways between the RA and control groups within the training set. The target gene set used was “c2.all.v7.2.symbols.gmt” from the MsigDB database. The ssGSEA algorithm in the GSVA package (v 1.46.0) was used to evaluate the GSVA score of each gene set in different samples.^[67]^ The difference in GSVA scores between the RA and control groups was then compared using the limma package (v 3.54.0), with a threshold of |*t*| > 2 and *P* < .05.

### 4.9. Immune microenvironment analysis

The CIBERSORT algorithm was used to analyze the infiltration level of immune cells in RA, identify the types of immune cells with significant differences, and analyze their correlations with biomarkers. We quantified the relative infiltration levels of 22 types of immune cells with the CIBERSORT algorithm in the training set.^[68,69]^ Samples with *P* > .05 were removed and the remaining data were visualized using the ggplot2 package (v 3.4.1). Then, the Wilcoxon test in the rstatix package (v 0.7.2) was conducted to ascertain the immune cells exhibiting notable disparities between 2 groups (*P* < .05). The results were then depicted in a box plot using the ggplot2 package (v 3.4.1). Spearman correlation analysis was then conducted to examine the relationship between the differential immune cells using the psych package (v 2.2.9), with a threshold of |cor| > 0.3 and *P* < .05. Eventually, Spearman correlation analysis was performed to examine the association between the biomarkers and the differential immune cells using the psych package (v 2.2.9) (|cor| > 0.3, *P* < .05), and the results were presented in a bar chart using the ggplot2 package (v 3.4.1).

### 4.10. Molecular regulatory network, potential drugs, and diseases prediction

Construct regulatory networks, reveal the transcriptional and epigenetic regulatory mechanisms of biomarkers, and explored potential drugs and related diseases. To further explore the mechanisms underlying RA biomarkers, we constructed 2 networks: one representing the interactions between mRNA, miRNA, and lncRNA, and another representing the interactions between TFs and mRNA. The miRNet database (https://www.mirnet.ca/) facilitated the prediction of the TFs corresponding to the biomarkers. The miRNAs that regulate these biomarkers were predicted using the MicroCosm database (http://www.ebi.ac.uk/enright-srv/microcosm/htdocs/targets/v5/). According to the predicted mRNA-miRNA relationship pairs, further prediction of lncRNAs was carried out using the StarBase database (http://starbase.sysu.edu.cn/). The therapeutic drugs targeting the biomarkers were extracted from the Drug Signatures Database (DSigDB, http://tanlab.ucdenver.edu/DSigDB). Similarly, the Comparative Toxicogenomics Database (https://ctdbase.org/) enabled the prediction of the diseases associated with the biomarkers, and the top 10 diseases for each biomarker were selected separately. The above regulatory networks were illustrated using Cytoscape software (v 3.9.1).

### 4.11. In vitro validation of the targets using real-time quantitative polymerase chain reaction (RT-qPCR)

#### 4.11.1. Sample collection and processing

Verify the expression differences of biomarkers in peripheral blood mononuclear cell (PBMCs) between RA patients and healthy controls. This study strictly adheres to the provisions of the Declaration of Helsinki and has been approved by the Ethics Committee of the Affiliated Hospital of Nanjing University of Chinese Medicine (No. 2023NL-052-03). All participants included in the study have signed informed consent. Nine patients with RA diagnosed in the outpatient and inpatient departments of the Department of Rheumatology and Immunology, Jiangsu Provincial Hospital of Traditional Chinese Medicine were selected. A control group of 7 healthy individuals, who had undergone physical examinations, was also included. Patient inclusion criteria: patients with RA included in this study were selected by relevant clinicians according to the current criteria refined by the American College of Rheumatology in 2010. Patient exclusion criteria: patients with concomitant systemic lupus erythematosus, Sjögren’s syndrome, and other autoimmune diseases. Patients with malignant tumors and systemic infections. Patients with severe heart, liver, kidney, or other organ failure. Individuals who declined to take part in this study. Inclusion criteria for the healthy control group: no RA diagnosis; no autoimmune diseases, including lupus or Sjögren syndrome; no family history of autoimmune diseases; subjects who agreed to participate.

#### 4.11.2. Isolation of human blood PBMCs and extraction of their RNA

To analyze the expression of genes related to RA patients by RT-qPCR, we collected 9 blood samples from RA patients and 7 samples from normal individuals. The detailed experimental procedure was as follows: 5 mL of venous blood from each subject was collected in an EDTA tube, diluted with 5 mL of PBS, and transferred to a 15 mL centrifuge tube. Take a new 50-mL one, add 10 mL PBMCs separation medium, tilt it, and slowly add the diluted blood along the wall to the medium’s upper layer with a pipette. Centrifuge at 1000 × *g* for 30 minutes at room temperature (RT) in a horizontal rotor. After that, 4 liquid layers were visible. Aspirate the buffy coat into a new tube and add PBS to 10 mL. Centrifuge at 250 × *g* for 10 minutes at RT in a horizontal rotor to get a visible cell pellet. The supernatant was discarded, and the pellet was resuspended with 5 mL PBS by pipetting up and down, centrifuged again under the same conditions and repeated twice. One milliliter of VeZol reagent was added to the washed cell pellet, pipetted up and down, and transferred to a 1.5 mL enzyme-free tube. After incubation at RT for 5 minutes, 200 µL of chloroform was added to the lysate, vortexed for 15 seconds, and incubated at RT for a further 5 minutes. The sample was centrifuged at 11,200 rpm for 15 minutes at 4°C. The upper ~500 μL aqueous phase was aspirated into a new enzyme-free tube. An equal volume of isopropanol was then incorporated, the tube inverted to mix, and left at RT for 10 minutes. The sample was centrifuged at 11,200 rpm for 10 minutes at 4°C to obtain a visible white precipitate. The supernatant was removed and 1 mL of 75% ethanol (RNase-free ddH_2_O prepared) was introduced. The bottom of the tube was gently swirled to resuspend the precipitate and the tube was inverted several times. The sample was centrifuged at 11,200 rpm (12,000 × *g*) for 5 minutes at 4°C and the supernatant discarded. The precipitate was dried in an open container at RT for 2 to 5 minutes in a clean area. Twenty to 100 μL of RNase-free ddH_2_O was added to dissolve it. The mixture was vortexed or pipetted repeatedly at RT to completely dissolve the RNA and then stored at −80°C.

#### 4.11.3. Experimental consumables

Enzyme-free pipette tips, 1.5 mL enzyme-free centrifuge tubes (KeyGEN, China); pipettes (Ebende); human peripheral blood mononuclear cell separation medium (Solarbio); PBS; isopropanol; ethanol; chloroform; 0.2 mL PCR tubes (axygen); fluorescence-quantitative 96-well PCR plates, fluorescence-quantitative nuclease-free sealing films (Mona, China), VeZol reagent (Vazyme, China); reverse transcription reagent HisyGo RT Red SuperMix (Vazyme, China); ChamQ Blue Universal SYBR RT-qPCR Master Mix (Vazyme, China).

#### 4.11.4. cDNA synthesis

Synthesise cDNA in a 20 µL reaction system. Measure the concentration of the extracted RNA using a spectrophotometer, calculate the amount to be added and pipette into a 0.2 mL PCR tube. Supplement RNase-free ddH_2_O to make the volume up to 15 μL. Then add 3 μL of 5 × gDNA Wiper Mix and 5 μL of 4 × HisyGo qRT Red SuperMix. Pipette up and down to mix well and react at 37°C for 15 minutes, then at 85°C for 5 seconds to give cDNA. Store at −20°C.

#### 4.11.5. Real-time quantitative PCR

A 20 µL reaction system was used containing 1 µL cDNA, 8.2 µL RNase-free ddH_2_O, 0.4 µL each of 10 µM forward and reverse primers, and 10 µL 2 × ChamQ Blue Universal SYBR RT-qPCR Master Mix. Amplification was performed using the specified reaction parameters. Results were computed using the 2^−ΔΔCt^ method with GAPDH as the reference gene. RT-qPCR steps and primer sequences are given in Tables S10 and S11, Supplemental Digital Content, https://links.lww.com/MD/P915. In this article, GraphPad Prism 10 (v 10.1.2, GraphPad Software LLC, San Diego) was used to analyze and plot the RT-qPCR data.^[70]^ Adobe Illustrator was used to organize the images. Statistical distinctions between groups were tested by *t*-test.

### 4.12. Statistical analysis

Bioinformatic analyses were performed by means of the R programming language (v 4.2.2). The Wilcoxon test was applied to assess disparities between the 2 groups. *P* < .05 was interpreted as statistically significant. The RT-qPCR data were analyzed using *t*-test in GraphPad Prism 10 software (*P < .05, **P < .01,***P < .001).

## Acknowledgments

We would like to express our sincere gratitude to all individuals and organizations who supported and assisted us throughout this research. Special thanks to the following authors: Ruiyang Xu, Zhixuan Zhang, Min Zhou, Zenan Xu, Jinchen Huang, Ke Gan, and Yan Lu. In conclusion, we extend our thanks to everyone who has supported and assisted us along the way. Without your support, this research would not have been possible.

## Author contributions

**Conceptualization:** Ruiyang Xu, Zhixuan Zhang, Min Zhou.

**Data curation:** Ruiyang Xu, Zhixuan Zhang, Jinchen Huang, Yan Lu.

**Formal analysis:** Ruiyang Xu, Min Zhou, Ke Gan.

**Funding acquisition:** Ruiyang Xu.

**Investigation:** Ruiyang Xu, Jinchen Huang, Ke Gan.

**Methodology:** Ruiyang Xu, Min Zhou, Zenan Xu.

**Project administration:** Ruiyang Xu, Zenan Xu.

**Resources:** Ruiyang Xu, Zhixuan Zhang, Min Zhou, Zenan Xu, Ke Gan.

**Software:** Ruiyang Xu, Ke Gan.

**Supervision:** Ruiyang Xu, Min Zhou, Ke Gan.

**Validation:** Ruiyang Xu, Jinchen Huang.

**Visualization:** Ruiyang Xu.

**Writing – original draft:** Ruiyang Xu, Zhixuan Zhang, Min Zhou, Yan Lu.

**Writing – review & editing:** Ruiyang Xu, Zhixuan Zhang, Yan Lu.

## Supplementary Material




