# Supplementary material for: Identification of mitochondria metabolism-related biomarkers associated with the development of rheumatoid arthritis using bioinformatics: An observational study
Source: Medicine (Baltimore). 2026 Jan 9;105(2):e44435. doi: 10.1097/MD.0000000000044435 (PMC12795057; doi:10.1097/MD.0000000000044435)
Supplement: Supplementary file 1 [file medi-105-e44435-s001.pdf]

S1 Table Detailed Information of Differentially Expressed Genes (DEGs) Identified in the Study.

	logFC	P.Value	adj.P.Val	change
MYL6	0.582820904747392	2.68936313888352e-27	2.16359264523179e-23	UP
BLOC1S1	0.511657529599939	6.69064917060141e-27	2.69131362887442e-23	UP
UBL5	0.674794459704589	2.10309638617841e-26	6.76776417072214e-23	UP
C1GALT1C1	0.734393388087309	2.74948601684629e-25	4.91547000122853e-22	UP
GLG1	-0.527368324	9.65149841733905e-25	1.42006306133837e-21	DOWN
CKS2	1.14769586621161	9.70832422294721e-25	1.42006306133837e-21	UP
POLB	0.539218651758821	4.9126172829936e-24	6.08030862179746e-21	UP
GGCX	0.559800441537989	7.47965551898819e-24	8.023177153368e-21	UP
CHCHD1	0.515844478344226	8.9169652729876e-24	8.96712320264816e-21	UP
CNIH4	0.841508576512732	3.68067951906732e-23	3.11695439272596e-20	UP
COX7A2	0.750435405104646	7.15619039569696e-23	5.48300492698876e-20	UP
UQCRQ	1.05153549839605	1.08965394073124e-22	7.96933268471165e-20	UP
LAMTOR3	0.854158895599136	1.86431354151972e-22	1.24660114277777e-19	UP
CKLF	0.740003057608558	3.25993274944818e-22	1.87329706923647e-19	UP
ORC4	0.578128258701984	6.78911305133904e-22	3.6412276332015e-19	UP
CD58	0.732548883129407	7.41841542214326e-22	3.85039690781565e-19	UP
PSMA2	0.805892944298215	1.20225783113249e-21	6.04510265716306e-19	UP
STARD3NL	0.590265635725236	2.58407902395713e-21	1.22287739692559e-18	UP
NDUFA4	1.14839087678709	2.69834143253199e-21	1.24046610426971e-18	UP
PHF5A	0.589069817540998	3.24568718001208e-21	1.45064185351095e-18	UP
S100A8	1.0389647415958	3.54015744143396e-	1.53450063141796e-	UP

	3	21	18	
POLE4	0.564352680086203	3.62405369756884e-21	1.53450063141796e-18	UP
COQ2	0.634062996833698	3.91238697930863e-21	1.60332174406583e-18	UP
RPL39	0.911310450467116	3.98588376399211e-21	1.60332174406583e-18	UP
UQCR11	0.570866395504005	5.50029600264852e-21	2.15853079713695e-18	UP
EVI2A	1.2315745770026	6.67398832898855e-21	2.55677314793871e-18	UP
CGRRF1	0.738014887048013	1.34627348715365e-20	4.70903052354397e-18	UP
MCTS1	0.621466929229351	2.90777651973052e-20	9.35722484049283e-18	UP
CLEC4D	1.21442530000972	3.7140812610499e-20	1.1492224517364e-17	UP
FAM91A1	0.573733929661287	4.30527867698124e-20	1.2828135909746e-17	UP
CSTA	1.22162487254912	5.51549080449485e-20	1.58471869722004e-17	UP
LY96	1.29362537089104	5.82251279573415e-20	1.62085094390102e-17	UP
DCHS1	-0.560527733	8.92472693349841e-20	2.35407961245884e-17	DOWN
NDUFB3	0.976960170309742	1.0522935130562e-19	2.68752422620226e-17	UP
TMCO1	0.892211819444466	1.25618454690294e-19	3.0624256605558e-17	UP
GLRX	0.670724591811345	1.61420606772366e-19	3.76414139560488e-17	UP
SF3B6	0.731527654753407	1.90611557265755e-19	4.31880430943956e-17	UP
MRPS28	0.96602520817953	1.93259111423026e-19	4.31880430943956e-17	UP
CASP3	0.718124590754008	2.83484634906461e-19	6.08169036752661e-17	UP
CAPZA2	0.777141554148358	3.03354954894946e-19	6.42234371613115e-17	UP
TXN	0.852034209374599	3.35269743408832e-19	7.00583139149104e-17	UP
RPS15A	0.720941816731152	4.11665795375228e-19	8.27962830948427e-17	UP
ATP6V1G1	0.5367898984880	4.30876421680226e-	8.55901435164795e-	UP

	69	19	17	
LSM1	0.528506268733863	5.24304224672088e-19	1.02878719206999e-16	UP
RPL22L1	1.35692364572484	5.84140716746126e-19	1.13238844969219e-16	UP
KIN	0.521174062903066	5.99340674961697e-19	1.14802279287306e-16	UP
PEX2	0.723412282692061	6.16417956879621e-19	1.16684293249331e-16	UP
NDUFB1	0.600646167502507	7.24211405041614e-19	1.35494901245576e-16	UP
STX7	0.589833543981857	8.88285883755295e-19	1.6428183758187e-16	UP
MS4A4A	1.23283106091139	1.16203743833149e-18	2.05463542667623e-16	UP
TMEM256	0.639978113060045	1.46503356534455e-18	2.50770107089296e-16	UP
PTPRO	-0.758280997	1.50837372176244e-18	2.55470875612186e-16	DOWN
BLOC1S2	0.729124756088714	2.50229476019166e-18	4.06686087792766e-16	UP
COMMD8	1.12649155208971	3.04586382300599e-18	4.83516988128026e-16	UP
MTHFD2	0.708964730864775	3.06517916650458e-18	4.83516988128026e-16	UP
RPL26L1	0.918865049347634	3.87634043727527e-18	5.94003025102468e-16	UP
TIMM8B	0.906133169171409	4.22804624295655e-18	6.41785509897838e-16	UP
TXNDC17	0.668024581528665	4.30530342221995e-18	6.47404972556252e-16	UP
LPAR6	0.994751209517743	4.37260782441219e-18	6.51437591618445e-16	UP
SMIM20	0.545408089391738	5.0446631589214e-18	7.31248920964373e-16	UP
PTRH2	0.529369045558541	5.83457461112339e-18	8.23493907833117e-16	UP
POLR2K	1.28039928235946	7.32056622729118e-18	1.00673427860782e-15	UP
NAT1	0.798421419661788	7.63495964148634e-18	1.04107203925013e-15	UP
TMEM126A	0.758102914339211	8.10171864945834e-18	1.09543405940996e-15	UP
CHMP5	0.9210339708109	8.36774340254694e-	1.10358189628672e-	UP

	48	18	15	
ANXA3	1.13088924140848	8.44256190434998e-18	1.10439691903245e-15	UP
HMGB2	0.832565871126699	9.41087155670747e-18	1.20389866114669e-15	UP
TBK1	0.544125527055332	9.46558249092464e-18	1.20389866114669e-15	UP
BCL2A1	1.39034773686638	1.2741727128709e-17	1.56499533970174e-15	UP
HAT1	1.07536355965146	1.53740736871237e-17	1.8341484030888e-15	UP
AMN1	0.534458389977848	2.18171999826477e-17	2.50741962657715e-15	UP
COX7C	0.952008189835205	2.6182261565328e-17	2.98774885522076e-15	UP
NMI	0.527421646609161	2.68375555720752e-17	2.99872409135201e-15	UP
RPL9	0.966786480821769	2.75443680075811e-17	3.05647504304814e-15	UP
CENPW	0.615126096022652	2.8346153027748e-17	3.12390138504428e-15	UP
DPY30	0.599204754613272	3.26946043499852e-17	3.56296420473957e-15	UP
HNMT	1.03066206651794	3.27730703729929e-17	3.56296420473957e-15	UP
NIPSNAP3A	0.759912459209604	3.80923078746638e-17	4.11345794431772e-15	UP
MIER1	0.505570392508318	4.27124989623991e-17	4.58162738870001e-15	UP
DYNLT1	0.554368623389033	4.77835118877173e-17	5.03768878711357e-15	UP
C3orf14	0.580548449718425	4.79034421645977e-17	5.03768878711357e-15	UP
TFEC	1.15432885954401	4.88074883566611e-17	5.09943173804336e-15	UP
GPR65	0.829346959993585	5.23361984953766e-17	5.39406955919554e-15	UP
NDUFB2	0.662601837649657	5.27499659423964e-17	5.39406955919554e-15	UP
VBP1	0.679574895846029	5.29179864006913e-17	5.39406955919554e-15	UP
SRP14	0.539200315716718	5.29684891456119e-17	5.39406955919554e-15	UP
GALNT3	0.6231454523835	7.46533842615175e-	7.27983607738071e-	UP

	17	17	15	
MRPL40	0.718517864410584	9.71396960844278e-17	9.19398652940261e-15	UP
PDCD10	0.882237912766239	9.84721365817864e-17	9.26559460585347e-15	UP
RPS27	0.714358502082998	1.11254819013495e-16	1.03473412596944e-14	UP
LRRK2	0.659876055582897	1.14310418480488e-16	1.04503104167673e-14	UP
KYNU	0.721958256900962	1.2527661053357e-16	1.12608975613695e-14	UP
CKS1B	0.531706860284783	1.43191369878721e-16	1.27997174519367e-14	UP
TNFSF10	0.559598710210503	1.82497825435129e-16	1.57870430712432e-14	UP
CMC2	0.59448567688051	2.20401883021106e-16	1.83744367762155e-14	UP
SUB1	1.15103824213532	2.3235166798558e-16	1.89996430384711e-14	UP
RPL27	0.5513506284573	2.35964486932941e-16	1.90787366570403e-14	UP
TBC1D15	0.502055657897855	2.72300483884987e-16	2.13722672473631e-14	UP
AKR1C3	1.01501101269757	2.80335222972408e-16	2.17903079112369e-14	UP
ATG5	0.528037402112069	3.60344193652301e-16	2.72203665533593e-14	UP
NXT2	0.656704992562249	3.62226218934434e-16	2.72346722553974e-14	UP
FAS	0.765227216091718	3.66317496101589e-16	2.74141791268584e-14	UP
C2orf76	0.81484695295409	3.71355447255339e-16	2.76625423441593e-14	UP
RBX1	0.762972242597634	4.20310749181158e-16	3.11649767480407e-14	UP
TMEM126B	0.706298184933842	4.27005063861244e-16	3.13721985275224e-14	UP
FBXO30	0.520151362811847	4.41232474773655e-16	3.22701387232187e-14	UP
C9orf72	0.694988304049318	4.47932342724578e-16	3.2611906762165e-14	UP
CHAC2	0.704249992832496	5.01768169600315e-16	3.60421868253084e-14	UP
MRPL22	0.5397686588543	5.07594533158634e-	3.62986490600997e-	UP

	5	16	14	
COX7B	1.33131246312229	5.21995275348569e-16	3.69995770059845e-14	UP
FAM76B	0.698653818512629	5.29466797079219e-16	3.73645647587922e-14	UP
UBE2W	0.558923292560143	6.34097193390841e-16	4.45529425399941e-14	UP
CARD16	0.832227485637731	6.6774937389083e-16	4.61119632013024e-14	UP
RPS7	1.18732571186628	6.93792575094318e-16	4.77056518515708e-14	UP
MRPL15	0.565099000199078	7.32112336641413e-16	5.01263297725971e-14	UP
COMMD3	0.511125044572874	7.40796313581627e-16	5.03167657145085e-14	UP
HINT1	0.809662439751301	7.6712830505186e-16	5.16447465618595e-14	UP
CMTM2	0.656792168070066	8.52739268078958e-16	5.66965901792994e-14	UP
PLA2G4A	0.743454903420208	8.89612847380619e-16	5.86634045670252e-14	UP
DPM1	0.697193008185344	9.95975813943912e-16	6.51432961234047e-14	UP
ZNHIT3	0.665894831883318	1.11010688089291e-15	7.14464788542676e-14	UP
VRK2	0.51445939459212	1.24422478269529e-15	7.82014716936221e-14	UP
MRPL13	0.904313100470027	1.2655529384126e-15	7.92324777395279e-14	UP
MRPS18C	0.792275989630613	1.42006131796506e-15	8.82192533052425e-14	UP
ANXA1	0.92892631380894	1.44804558159324e-15	8.95780470844492e-14	UP
PRDX4	0.520438343578888	1.4530683834084e-15	8.95780470844492e-14	UP
TWF1	0.685719337499699	1.58199577642503e-15	9.71538627583158e-14	UP
VPS29	0.524898153229248	1.90254930616111e-15	1.14223949015419e-13	UP
VCPKMT	0.593480679013028	1.95697464655817e-15	1.16621192826374e-13	UP
ZNF230	0.635598512056536	2.28865549293285e-15	1.34888157074321e-13	UP
GNG10	0.5719936084963	2.44993123979951e-	1.43343249630452e-	UP

	9	15	13	
TXNDC9	0.69105923149599	2.88139496700506e-15	1.64988060566233e-13	UP
SPAG1	0.74230280575371	3.01192477003327e-15	1.71243355299771e-13	UP
SNX14	0.576162855366479	3.38832680925682e-15	1.89958809620008e-13	UP
GAPT	0.569066635146452	3.5795805674569e-15	1.98605004587523e-13	UP
IMMP1L	0.777710469156372	3.73206286670864e-15	2.04945022270792e-13	UP
DCUN1D5	0.574325796320166	3.75180431372849e-15	2.05328338122079e-13	UP
COPS2	0.917254886700781	4.28021334720685e-15	2.31102794485094e-13	UP
COMMD6	1.3811499780414	4.39346865882563e-15	2.34853523988387e-13	UP
RPS17	0.841859344613868	4.5583525402493e-15	2.42059050734691e-13	UP
CD52	0.837209241346331	4.75692260438311e-15	2.51772647054356e-13	UP
PSMA4	0.828003149015638	5.03382734050947e-15	2.65555022651795e-13	UP
MPP7	0.579486898218122	5.2832066401426e-15	2.7779998313691e-13	UP
RPL41	0.58419758631215	5.35015471053371e-15	2.80403873916897e-13	UP
SLC38A6	0.585775095355252	5.54270095057553e-15	2.88615075387574e-13	UP
GIMAP2	0.683612296781673	6.27246054715774e-15	3.23474007063359e-13	UP
GAB1	0.580299700079489	6.89809561313818e-15	3.53472479029915e-13	UP
SNRPG	0.965241974516038	7.95351653863174e-15	4.03697416740015e-13	UP
RPA3	0.518215346649961	8.83916867824152e-15	4.40924430168722e-13	UP
CLEC2B	1.20512521843394	8.88335072153929e-15	4.41151583671504e-13	UP
BAZ2B	0.577289817261123	9.43487761364211e-15	4.67099017856928e-13	UP
NAB1	0.587730001369085	1.20862707634063e-14	5.82239810129366e-13	UP
CHUK	0.5377005665820	1.28379030093517e-	6.12943203028098e-	UP

	96	14	13	
RBBP8	0.629457494424115	1.46708597769701e-14	6.88204471753498e-13	UP
PSMC6	0.941143711231856	1.54526809571334e-14	7.16523448415784e-13	UP
ECHDC1	0.592992650957997	1.88540043900228e-14	8.59379406899337e-13	UP
ST3GAL6	0.723624427809042	1.93281486035824e-14	8.78502573535711e-13	UP
ACSL4	0.683609899431535	2.16659347798097e-14	9.76484287414953e-13	UP
ERH	0.579614225420205	2.18100610253298e-14	9.80234306976416e-13	UP
DBI	0.870307186541796	2.28115043056271e-14	1.0167232805472e-12	UP
NUDCD1	0.768003351864274	2.4981965005799e-14	1.10074039016231e-12	UP
ZNF267	0.881153910940759	2.50385943318462e-14	1.10074039016231e-12	UP
SAMSN1	0.666094761643244	2.56376866724705e-14	1.12400648109006e-12	UP
SNX16	0.681264114239576	2.64533312998197e-14	1.15661440384266e-12	UP
EMC2	0.726186327202986	2.87127984540161e-14	1.24525317284399e-12	UP
CPEB2	0.754877461009422	3.06693109054452e-14	1.3194363969749e-12	UP
CCDC126	0.604408318805331	3.30552147294608e-14	1.40703281745244e-12	UP
RAB18	0.616055615137631	3.39355222879321e-14	1.43313006197592e-12	UP
SAMD9	0.686234653315758	4.09734347833362e-14	1.70793410793751e-12	UP
ZFYVE16	0.719450893505813	4.16574331720725e-14	1.73195891405335e-12	UP
TOMM5	0.763614173951383	4.17819740599413e-14	1.73265969748571e-12	UP
RSL24D1	1.04927302676834	4.92945380946749e-14	2.01039835773986e-12	UP
SCOC	1.05020695990988	6.65509732102149e-14	2.62756446174018e-12	UP
MS4A6A	0.610901311561546	7.4637019194792e-14	2.89375816588965e-12	UP
LPCAT2	0.6661944010997	7.81799768776548e-	3.01658471933205e-	UP

	39	14	12	
CD69	1.04255305659994	7.92344289708201e-14	3.04995684722607e-12	UP
HIGD1A	0.511809945422412	1.03635125654915e-13	3.9142938304873e-12	UP
TRIAP1	0.537692291697373	1.21306481708957e-13	4.49728407994727e-12	UP
NABP1	0.56627366290156	1.26634736625707e-13	4.6732864961184e-12	UP
MRPL47	0.708973261980852	1.36341614129644e-13	4.98576493487719e-12	UP
GTF2H5	0.621486881161988	1.41056382185605e-13	5.12324421978869e-12	UP
AZI2	0.543127807016239	1.54215289815078e-13	5.54379510518095e-12	UP
DNAJC15	0.591252072317062	1.90286714669362e-13	6.67039921357306e-12	UP
ZC3H12C	0.528830039271049	1.92369429598742e-13	6.72874809183425e-12	UP
YIPF4	0.552090643482155	1.9901805884128e-13	6.93117005791385e-12	UP
MYNN	0.651947955753108	2.60308909536026e-13	8.87366600515818e-12	UP
IL15	0.638401474599238	2.6467782120039e-13	9.00352250129867e-12	UP
IMPA1	0.542891265642041	2.68904682462652e-13	9.12800915785670e-12	UP
TTC33	0.73433216942592	3.00983059215352e-13	1.01016059302445e-11	UP
SCRN3	0.519011121249497	3.34981273947625e-13	1.11204920182628e-11	UP
BCL11B	-0.513836816	3.35204389612024e-13	1.11204920182628e-11	DOWN
HINT3	0.613915180569766	4.2119161911831e-13	1.37185691328211e-11	UP
JAK2	0.554615089260726	4.33496935326006e-13	1.40341361959667e-11	UP
FMR1	0.537300457946069	4.74540587903664e-13	1.5240235647445e-11	UP
RRM2B	0.570640569366377	4.86949004478819e-13	1.56075886096896e-11	UP
RWDD1	0.644305713929231	4.95658570950287e-13	1.58237031876788e-11	UP
GSKIP	0.6822591497825	5.04274821246655e-	1.60668947997202e-	UP

	78	13	11	
HAUS1	0.768741013920509	5.3826971160757e-13	1.70152449111313e-11	UP
ZCCHC10	0.921590640915898	5.8870487061018e-13	1.83927405206171e-11	UP
FCGR2B	0.500610095779066	6.49114571653923e-13	2.01238024237218e-11	UP
NDUFA6	0.519651265150761	6.79481363967066e-13	2.09041207384897e-11	UP
MTHFD2L	0.50525267729992	7.1998684432159e-13	2.19671422198305e-11	UP
LYSMD3	0.564659263203188	8.81759835596165e-13	2.61762283297828e-11	UP
LSM7	0.513714814168603	9.18117121129518e-13	2.71553391157609e-11	UP
ACTR6	0.837638007502204	1.14688357580803e-12	3.29755974517925e-11	UP
SLC35A1	0.586175001206596	1.14769015369818e-12	3.29755974517925e-11	UP
PARP9	0.580078026178429	1.16631954115489e-12	3.339749358338e-11	UP
SKIL	0.629304041742679	1.22409265188841e-12	3.48595588829815e-11	UP
XRCC4	0.541981305079791	1.26082982473517e-12	3.58423178091676e-11	UP
LSM3	0.651216956531474	1.28798755822106e-12	3.64213001964444e-11	UP
RPL7	0.749112967166198	1.31213911500367e-12	3.70391550182616e-11	UP
DCTN6	0.503716451052725	1.75408239000624e-12	4.87447075219351e-11	UP
BTLA	0.686577422543303	1.79768150837617e-12	4.97843295521042e-11	UP
IFT57	0.680353684431132	1.85678571351392e-12	5.11569899493818e-11	UP
SENP7	0.702371291237267	1.96562411970117e-12	5.38788621567152e-11	UP
MFN1	0.52484290330062	2.21077662919557e-12	5.97451076370596e-11	UP
ESCO1	0.510259196350138	2.6070524422236e-12	6.91062171258281e-11	UP
FCGBP	-0.552258251	2.61942575352638e-12	6.92061746703438e-11	DOWN
BIRC2	0.5005470798863	2.81635381272363e-	7.41655202074031e-	UP

	28	12	11	
MRPL3	0.631370430582699	2.93069516258786e-12	7.66746100260792e-11	UP
TPRKB	0.5714586749166	3.14144801223755e-12	8.15256427691971e-11	UP
SNX13	0.532592981918403	3.17369501904556e-12	8.22298757752705e-11	UP
TRAPPC13	0.718502464411887	3.32612474055117e-12	8.56277553207493e-11	UP
CCDC82	0.770715241413291	3.53610994837917e-12	9.03111255070172e-11	UP
ZNF780A	0.515331323456495	3.59034162046431e-12	9.15508663601754e-11	UP
PNPLA8	0.508673016880611	3.62660855195303e-12	9.20380624620256e-11	UP
GMNN	0.547936856313953	3.89153700995915e-12	9.86060322680988e-11	UP
CMC1	0.617870350211908	4.13509955072346e-12	1.04284877384233e-10	UP
NMD3	0.528196800094626	4.20385773645598e-12	1.05752341150295e-10	UP
RPL31	1.30439013003187	4.68642920341973e-12	1.16185895043179e-10	UP
PTS	0.538488170691158	4.77008793637316e-12	1.17896643465813e-10	UP
ABHD13	0.634826291124701	5.44792623967107e-12	1.33015376625656e-10	UP
ANAPC10	0.730403916657576	5.65710736008212e-12	1.37704776737854e-10	UP
FAR1	0.571440879205893	5.85106770065667e-12	1.41144946482108e-10	UP
SLC48A1	-0.591587916	6.96680256615727e-12	1.65089621928528e-10	DOWN
PRDM5	0.769635477448777	7.67201633056007e-12	1.79945689152641e-10	UP
S100A12	0.859390401029671	7.81222757935024e-12	1.81908454054624e-10	UP
IL18	0.52140935864685	8.20754589746847e-12	1.9056192422838e-10	UP
RPS27L	0.954824686974238	8.96855718384354e-12	2.05561374769291e-10	UP
MAD2L1	0.670703561744888	9.13187226106043e-12	2.07824363055817e-10	UP
LMNB1	0.6167537813214	9.72739459493466e-	2.19206973434872e-	UP

	72	12	10	
CRBN	0.549874192615878	1.00055764527346e-11	2.2516045471958e-10	UP
KMO	0.596565890458097	1.41197866008891e-11	3.09098457698374e-10	UP
TVP23B	0.657181610422211	1.41610458476431e-11	3.09580472402958e-10	UP
GIN1	0.657095982059441	1.62388789527743e-11	3.50246062131554e-10	UP
KRCC1	0.585977676280073	1.7147379997302e-11	3.67868458875452e-10	UP
EEF1E1	0.74197249071281	1.89379388057729e-11	4.02525013718476e-10	UP
IKBIP	0.569831800025361	1.90465709640095e-11	4.04299903444475e-10	UP
AIM2	0.677012542747795	2.22229581525951e-11	4.63170202947222e-10	UP
NDUFS4	0.624445995596632	2.26570498072723e-11	4.71602144642201e-10	UP
SLIRP	0.526412866148254	2.28034748011085e-11	4.72819471069376e-10	UP
AKAP7	0.607479971900559	2.2997777569872e-11	4.75622417865348e-10	UP
RNF217	0.655574856409382	2.56200959298075e-11	5.21147084084201e-10	UP
DNAJB14	0.502457508587311	2.80386236817509e-11	5.64928922881259e-10	UP
SC5D	0.561747641305633	2.80533380597965e-11	5.64928922881259e-10	UP
RPL34	1.10689591832137	2.85441346954808e-11	5.72300840929033e-10	UP
TMX1	0.585375886021147	2.85616889537609e-11	5.72300840929033e-10	UP
CPEB4	0.514340112048714	3.08658572003882e-11	6.14643121725552e-10	UP
ITGAV	0.672782166531175	3.28709396964335e-11	6.51346576004452e-10	UP
SAP30	0.58348857702546	3.71473050689615e-11	7.25364245824745e-10	UP
ZNF22	0.503822015623195	4.80573393193286e-11	9.18340367752966e-10	UP
PPIL4	0.534144090860464	5.20632407770464e-11	9.85526522473738e-10	UP
DOCK4	0.7592680707484	5.79565587067796e-	1.08812255494992e-	UP

	95	11	09	
ZDHHC17	0.524290901396553	7.35120272517014e-11	1.35332782434768e-09	UP
NDUFA5	0.909829951556535	8.2458650892007e-11	1.50768146915045e-09	UP
CETN3	0.955532618809442	9.70485498990682e-11	1.73694234468966e-09	UP
ZNF184	0.554954584225442	1.01232963889409e-10	1.80781175247569e-09	UP
RAB33B	0.615105085597535	1.06264099487944e-10	1.89345444159581e-09	UP
FCGR1B	0.824023857447472	1.10667467749075e-10	1.96755752053328e-09	UP
PPP4R2	0.512468653279667	1.12808263429423e-10	2.00340503154462e-09	UP
SULT1B1	0.594059097002005	1.33271768147655e-10	2.33843266030073e-09	UP
ABCA1	0.61128954569647	1.42553460825105e-10	2.49042908216715e-09	UP
FANCL	0.568781315251202	2.02567606265407e-10	3.42724793355457e-09	UP
NDC80	0.575964649173816	2.35537047199349e-10	3.90700112313147e-09	UP
NFYB	0.606968227880312	2.43466475047742e-10	4.0137044913096e-09	UP
GZMA	0.792831040827787	2.96883528876574e-10	4.82023812272863e-09	UP
COX16	0.714311246643042	3.00919724146065e-10	4.87592987060442e-09	UP
COX6C	0.656853057826586	3.06142946192508e-10	4.9456224942143e-09	UP
EAF2	0.697786286789195	3.50892727771418e-10	5.59550444979397e-09	UP
RECQL	0.512110688283578	3.95329302449434e-10	6.16959115073851e-09	UP
ARID4B	0.51499209115547	4.16895237158502e-10	6.44985035180798e-09	UP
FAM126B	0.616048415378506	4.56496315451163e-10	6.98642391886635e-09	UP
PTBP2	0.567777823017139	5.55663082968771e-10	8.30141040386956e-09	UP
FCHO2	0.585963704682939	5.79444849161718e-10	8.62128584779515e-09	UP
IFIT5	0.7237982530380	6.38506745823709e-	9.46001246805108e-	UP

	9	10	09	
FPGT	0.552177806840517	7.43885974299077e-10	1.08612752508822e-08	UP
PET100	0.525355854354547	7.91009560093714e-10	1.14867724024439e-08	UP
KBTBD8	0.696170467526562	9.82822131459801e-10	1.40067387911321e-08	UP
TNFAIP6	0.823980850923615	9.94246925108487e-10	1.41570203761023e-08	UP
ARG1	1.18449283703669	1.29302600155372e-09	1.79197143540046e-08	UP
RNFT1	0.537818294297812	1.30631801368619e-09	1.80727917800609e-08	UP
LIN7A	0.629112317528067	1.43091521411081e-09	1.9644561258569e-08	UP
TRAT1	0.785045144446372	1.45256828040104e-09	1.99078565857349e-08	UP
MRPL1	0.565073124263334	1.46322896235196e-09	2.00118094782079e-08	UP
ANKRD22	1.15485541250922	2.13595377930358e-09	2.80780198602897e-08	UP
CASP5	0.561205636322874	2.16757191619426e-09	2.84008404980176e-08	UP
AP1AR	0.560783960539996	2.25292372197354e-09	2.93994668990707e-08	UP
VNN2	0.530055615195367	2.35430147896799e-09	3.05983124366679e-08	UP
SGMS2	0.609287897691156	2.37338148732945e-09	3.07717229098557e-08	UP
RWDD3	0.531022867700883	2.40458847464588e-09	3.11261693942496e-08	UP
METTL18	0.581963500544406	2.45895250338299e-09	3.17277833034741e-08	UP
MED7	0.529424709946873	2.48769594390457e-09	3.2047259997938e-08	UP
CCNT2	0.510347879619185	2.55085261441388e-09	3.27037598134815e-08	UP
SAMD9L	0.569867781078889	2.94856977548616e-09	3.70644435059158e-08	UP
CEACAM1	0.544781423424216	3.17936570373641e-09	3.97482472207606e-08	UP
TMEM170B	0.547801820458603	3.27309631265102e-09	4.07643111428533e-08	UP
SEMA3C	0.5710534726739	3.32017638017179e-	4.11568859452728e-	UP

	16	09	08	
MIER3	0.540023339733561	3.41263909372599e-09	4.22055057786711e-08	UP
SNRPE	0.57267475609232	4.90814888614233e-09	5.85412272631802e-08	UP
HECW2	0.589772209060143	4.99878326272538e-09	5.94899576163102e-08	UP
SNRPD2	0.518204743766638	5.06972161410364e-09	6.01562100080587e-08	UP
RFX3	0.513735929443964	5.86893720212593e-09	6.88776072809673e-08	UP
B3GNT5	0.523091137249296	5.88703211303175e-09	6.90202750914783e-08	UP
NRG1	0.870888776825882	7.55052633975941e-09	8.52674899692416e-08	UP
BMX	0.664372862006916	9.19967802803320e-09	1.01803864835663e-07	UP
PFDN5	0.618707418139932	9.36167492073474e-09	1.03454223540262e-07	UP
POLI	0.505254813783479	1.01277257128452e-08	1.1085381409502e-07	UP
MAP4K3	0.62269948500401	1.23589315014567e-08	1.31517994615369e-07	UP
RNASE4	0.519997053057537	1.30856093759714e-08	1.38245210019291e-07	UP
AGL	0.550295499730452	1.57341850951049e-08	1.6364772991612e-07	UP
SPARC	-0.68274682	1.78538116174896e-08	1.82508150524401e-07	DOWN
MCEMP1	0.504065254891138	2.26264584671867e-08	2.24036748761252e-07	UP
PHTF2	0.507714222867182	2.31922041436464e-08	2.28793724507216e-07	UP
SLPI	0.625197625157079	2.36922856200723e-08	2.33142361186752e-07	UP
LRRN3	-0.980850064	2.37054632008406e-08	2.33142361186752e-07	DOWN
RAP2A	0.532367949834001	2.42290276946259e-08	2.38000644448432e-07	UP
NRGN	-0.634071525	3.63699789017213e-08	3.44231153252174e-07	DOWN
RNASE2	0.763097983595729	3.65675538377205e-08	3.45745610056787e-07	UP
ECT2	0.5191198858700	3.75838590432303e-	3.5405403513207e-0	UP

	87	08	7	
YOD1	0.655473436745686	4.09652705050853e-08	3.82993144931332e-07	UP
SARNP	0.552636499999298	4.27009822347146e-08	3.9619727855419e-07	UP
FMNL2	0.528291173882618	4.27223292909584e-08	3.9619727855419e-07	UP
ZNF117	0.696729739502508	4.31074359148558e-08	3.99538389326054e-07	UP
PMAIP1	0.510663030727948	4.35530363560071e-08	4.03436013222887e-07	UP
DDX58	0.505740106734063	4.94779725699311e-08	4.52073014565697e-07	UP
PRPF39	0.519984493565556	5.34288343169282e-08	4.84321095301056e-07	UP
CHURC1	0.660597134980353	6.28466745338295e-08	5.59913063814682e-07	UP
RPS24	0.816397432141033	6.35120221039414e-08	5.65527634561382e-07	UP
MRPL50	0.605743719961808	9.21004443435829e-08	7.94124582980293e-07	UP
KLRB1	0.571916189348736	1.22869143064643e-07	1.0280626687e-06	UP
C1QB	0.521539670981154	1.38858433931859e-07	1.1434146376477e-06	UP
IFIT1	0.989312721010121	1.82749689722103e-07	1.47095673218041e-06	UP
IFI6	0.737986464285585	1.9191500832593e-07	1.53094322457323e-06	UP
ZBTB41	0.506660281264035	3.01199079601743e-07	2.28706615893915e-06	UP
PTGS2	0.531555106491177	3.3320851893924e-07	2.51587286238028e-06	UP
CD274	0.624555066149858	3.36348769851734e-07	2.53720192541697e-06	UP
S100P	1.52296422227566	3.6280263967489e-07	2.71133045627913e-06	UP
RTP4	0.590690331485064	3.65283343032871e-07	2.72607095983252e-06	UP
MACROD2	0.644274964564455	5.35450021783291e-07	3.83930073551388e-06	UP
EPSTI1	0.866887160716217	6.1207755228043e-07	4.3442116524888e-06	UP
CXCL10	0.6908505037193	6.56363464900795e-	4.62993781247426e-	UP

	27	07	06	
ZNF92	0.508255156928626	7.27925918626163e-07	5.07027187475972e-06	UP
SMPDL3A	0.502201606301522	7.48364054396006e-07	5.19912678550593e-06	UP
IFI44	1.06076594335255	7.60732029886295e-07	5.26459284338515e-06	UP
IFIT3	0.603993804180934	7.64470525745632e-07	5.28819035221291e-06	UP
RGS1	0.550713781761928	1.05524237204365e-06	7.06568862512786e-06	UP
THBS1	-0.533294649	1.47613759391111e-06	9.60414633482807e-06	DOWN
TUBB1	-0.523531525	1.58321795654519e-06	1.02387367045065e-05	DOWN
CAMP	0.513828439538593	1.66136111806132e-06	1.07053665957576e-05	UP
TNFRSF17	0.729817730986266	2.66624448152196e-06	1.64053054331504e-05	UP
KLRF1	0.535151416843923	2.6743194358554e-06	1.64424148731041e-05	UP
VWCE	-0.551711752	2.72694934240506e-06	1.67276457946235e-05	DOWN
MYBL1	0.514604700528269	2.84120500468871e-06	1.73031750664047e-05	UP
JAZF1	-0.519818197	3.24442444369303e-06	1.95442865215353e-05	DOWN
IFIT2	0.508660916918904	3.383362402829e-06	2.02900861205809e-05	UP
PDK4	0.589472752952186	4.13542148211124e-06	2.43464806612403e-05	UP
CLC	0.668938234524457	4.43970210758628e-06	2.5938564600967e-05	UP
P2RY14	0.584031534900559	5.29330973138974e-06	3.02555430117446e-05	UP
DDX60	0.542689978257014	1.12624487735937e-05	5.94824045256452e-05	UP
HERC5	0.696666559794105	1.17631343235094e-05	6.18276684482631e-05	UP
VNN1	0.601757231853547	1.19782762184498e-05	6.2799108620025e-05	UP
SLC38A5	-0.555332705	1.24696264673739e-05	6.50993802271404e-05	DOWN
UQCRB	0.5962065035454	1.33585949777212e-	6.92237659231996e-	UP

	07	05	05	
KLRC3	0.629326770714012	1.70667024376532e-05	8.64892101486112e-05	UP
HEMGN	-0.601041866	1.74161115093358e-05	8.81065973114929e-05	DOWN
RSAD2	1.05244784652977	2.41889084084251e-05	0.000118225861570948	UP
NFIX	-0.59956073	2.45827785237492e-05	0.000119896000741778	DOWN
CRISP3	0.721417928866678	2.47311404438367e-05	0.0001205465161288 5	UP
PSPH	0.883023733634923	2.48886516637575e-05	0.000121240812979067	UP
DEFA4	0.847083505835905	2.79692642915851e-05	0.000134496551838495	UP
CXCL8	0.573824226285184	3.12074368572092e-05	0.00014809859058685	UP
CLU	-0.512298857	3.97180416654657e-05	0.000183797322518649	DOWN
TGFB1I1	-0.514775091	6.06882021809658e-05	0.000267307192195932	DOWN
GATA2	-0.58437337	6.34440751082028e-05	0.000278086076444065	DOWN
TMTC1	0.646841063782577	6.41347484965837e-05	0.000280644031359813	UP
BATF2	0.528328558086606	6.71586484022355e-05	0.00029260293874681	UP
HP	0.519349097639835	8.78173520245846e-05	0.000368467722651621	UP
GBP5	0.543218843096128	9.19755336161191e-05	0.000383489592092085	UP
ISG15	0.66520566676072	0.000100804568451048	0.000416096846171718	UP
ITLN1	0.557307976242481	0.000104248408567419	0.000428357836992031	UP
IFI44L	0.91884094405072	0.00013365881807322	0.000534037840277653	UP
BPGM	0.561086528133618	0.000220474672997827	0.000835084154551563	UP
LAIR2	0.557871102840518	0.000234442045987985	0.000880937066778765	UP
CMPK2	0.683458674136524	0.000292186847793559	0.00106774616874821	UP
XK	0.5816452835937	0.0003366358603855	0.0012060723655317	UP

	22	15	2	
CLEC12A	0.60202974902476	0.000382305415997209	0.00134808111842978	UP
SCN3A	0.500609175538792	0.000406910474467942	0.00142547126805774	UP
CLEC12B	0.64116918423326	0.000462509078421815	0.00159525855344886	UP
OAS1	0.506265596082095	0.000714893851808682	0.00235661587289525	UP
SERPING1	0.507757351876803	0.00121816122814453	0.00374478680948519	UP
CA1	0.516455462819664	0.00382932067211895	0.0101656112216456	UP
IFI27	0.833276941011627	0.00808359683264481	0.0194795676257683	UP

S2 Table Complete Results of Gene Ontology (GO) Enrichment Analysis for Candidate Genes.

	ONTOLOGY	ID	Description	Gene Ratio	BgRatio	pvalue	p.adjust	qvalue	geneID	Count	richFactor
GO:0019646	BP	GO:0019646	aerobic electron transport chain	15/39	94/18870	2.0413517613603 6e-25	2.0250209472694 7e-22	1.4676244768517 1e-22	COX7A2/UQCRQ/NDUFA4/UQCR11/NDUFB3/NDUFB1/COX7C/NDUFB2/COX7B/DNAJC15/NDUFA6/NDUFS4/NDUFA5/COX6C/UQCRB	15	1.59574468085106
GO:0042773	BP	GO:0042773	ATP synthesis coupled electron transport	15/39	102/18870	7.5878782260083 6e-25	2.5090584000667 6e-22	1.8184283608293 7e-22	COX7A2/UQCRQ/NDUFA4/UQCR11/NDUFB3/NDUFB1/C	15	1.47058823529412

									OX7C/NDUFB2/COX7B/DNAJC15/NDUFA6/NDUFS4/NDUFA5/COX6C/UQCRB		
GO:0042775	BP	GO:0042775	mitochondrial ATP synthesis coupled electron transport	15/39	102/18870	7.5878782260083 6e-25	2.5090584000667 6e-22	1.8184283608293 7e-22	COX7A2/UQCRQ/NDUFA4/UQCR11/NDUFB3/NDUFB1/COX7C/NDUFB2/COX7B/DNAJC15/NDUFA6/NDUFS4/NDUFA5/COX6C/UQCRB	15	1.47058823529412

GO:0022904	BP	GO:0022904	respiratory electron transport chain	15/39	124/18870	1.6900431702058 9e-23	4.1913070621106e-21	3.0376302243437 4e-21	COX7A2/UQCRQ/NDUFA4/UQCR11/NDUFB3/NDUFB1/COX7C/NDUFB2/COX7B/DNAJC15/NDUFA6/NDUFS4/NDUFA5/COX6C/UQCRB	15	1.20967741935484
GO:0006119	BP	GO:0006119	oxidative phosphorylation	15/39	148/18870	2.7056347499960 9e-22	5.3679793439922 4e-20	3.8904179668364 8e-20	COX7A2/UQCRQ/NDUFA4/UQCR11/NDUFB3/NDUFB1/COX7C/NDUFB2/COX7B/	15	1.01351351351351

									DNAJC15/NDUFA6/NDUFS4/NDUFA5/COX6C/UQCRB		
GO:0022900	BP	GO:0022900	electron transport chain	15/39	176/18870	3.9682348412199e-21	6.5608149374835 7e-19	4.7549199939529 7e-19	COX7A2/UQCRQ/NDUFA4/UQCR11/NDUFB3/NDUFB1/COX7C/NDUFB2/COX7B/DNAJC15/NDUFA6/NDUFS4/NDUFA5/COX6C/UQCRB	15	0.852272727272727
GO:0009060	BP	GO:0009060	aerobic respiration	15/39	197/18870	2.2442504107544 2e-20	3.1804234392405 5e-18	2.3049970384139 4e-18	COX7A2/UQCRQ/NDUFA	15	0.761421319796954

									4/UQCR11/NDUFB3/NDUFB1/COX7C/NDUFB2/COX7B/DNAJC15/NDUFA6/NDUFS4/NDUFA5/COX6C/UQCRB		
GO:0045333	BP		GO:0045333	cellular respiration	15/39	243/18870	5.49495674275607e-19	6.81374636101753e-17	4.93823086224e-17	COX7A2/UQCRQ/NDUFA4/UQCR11/NDUFB3/NDUFB1/COX7C/NDUFB2/COX7B/DNAJC15/NDUFA6/NDU	15	0.617283950617284

										FS4/NDUFA5/COX6C/UQCRB		
GO:0015980	BP		GO:0015980	energy derivation by oxidation of organic compounds	15/39	337/18870	7.50189429693931e-17	8.26875460284866e-15	5.99274129217491e-15	COX7A2/UQCRQ/NDUFA4/UQCR11/NDUFB3/NDUFB1/COX7C/NDUFB2/COX7B/DNAJC15/NDUFA6/NDUFS4/NDUFA5/COX6C/UQCRB	15	0.445103857566766
GO:0006120	BP		GO:0006120	mitochondrial electron transport, NADH to ubiquinone	8/39	51/18870	9.24042191981434e-14	9.16649854445583e-12	6.64337702235073e-12	NDUFA4/NDUFB3/NDUFB1/NDUFB2/DNAJC15/N	8	1.56862745098039

									DUFA6/NDUFS4/NDUFA5		
GO:0006123	BP	GO:0006123	mitochondrial electron transport, cytochrome c to oxygen	5/39	25/18870	1.4896201147259e-09	1.3433665034619 1e-07	9.7359860129932 3e-08	COX7A2/NDUFA4/COX7C/COX7B/COX6C	5	2
GO:0010257	BP	GO:0010257	NADH dehydrogenase complex assembly	6/39	60/18870	2.4039876785923e-09	1.8344275208950 5e-07	1.3294927809542 8e-07	NDUFB3/NDUFB1/NDUFB2/NDUFA6/NDUFS4/NDUFA5	6	1
GO:0032981	BP	GO:0032981	mitochondrial respiratory chain complex I assembly	6/39	60/18870	2.4039876785923e-09	1.8344275208950 5e-07	1.3294927809542 8e-07	NDUFB3/NDUFB1/NDUFB2/NDUFA6/NDUFS4/NDUFA5	6	1
GO:0042776	BP	GO:0042776	proton motive force-drive	6/39	67/18870	4.7419178335929 1e-09	3.3599874935172 6e-07	2.4351352483789 2e-07	NDUFB3/NDUFB1/NDUF	6	0.895522388059701

			n mitochondrial ATP synthesis						B2/NDUFA6/NDUFS4/NDUFA5		
GO:0009165	BP	GO:0009165	nucleotide biosynthetic process	9/39	295/18870	6.9365309080479 1e-09	4.5609109471836 1e-07	3.3054989143955 9e-07	NDUFB3/NDUFB1/NDUFB2/ACSL4/RRM2B/NDUFA6/NDUFS4/NDUFA5/PDK4	9	0.305084745762712
GO:1901293	BP	GO:1901293	nucleoside phosphate biosynthetic process	9/39	297/18870	7.3563079793284 1e-09	4.5609109471836 1e-07	3.3054989143955 9e-07	NDUFB3/NDUFB1/NDUFB2/ACSL4/RRM2B/NDUFA6/NDUFS4/NDUFA5/PDK4	9	0.303030303030303
GO:0015986	BP	GO:0015986	proton motive force-drive	6/39	76/18870	1.0248472660710 4e-08	5.8005486635783 7e-07	4.2039205615704 8e-07	NDUFB3/NDUFB1/NDUF	6	0.789473684210526

			n ATP synthesis						B2/NDUFA6/NDUFS4/NDUFA5		
GO:0009152	BP	GO:0009152	purine ribonucleotide biosynthetic process	8/39	213/18870	1.05251891072995e-08	5.80054866357837e-07	4.20392056157048e-07	NDUFB3/NDUFB1/NDUFB2/ACSL4/NDUFA6/NDUFS4/NDUFA5/PDK4	8	0.375586854460094
GO:0009260	BP	GO:0009260	ribonucleotide biosynthetic process	8/39	228/18870	1.79032946802983e-08	9.34740438045048e-07	6.7744876823511e-07	NDUFB3/NDUFB1/NDUFB2/ACSL4/NDUFA6/NDUFS4/NDUFA5/PDK4	8	0.350877192982456
GO:0046390	BP	GO:0046390	ribose phosphate biosynthetic process	8/39	235/18870	2.2654151174399e-08	1.12364589825019e-06	8.14357118532342e-07	NDUFB3/NDUFB1/NDUFB2/ACSL4/NDU	8	0.340425531914894

									FA6/NDUFS4/NDUFA5/PDK4		
GO:0009150	BP	GO:0009150	purine ribonucleotide metabolic process	9/39	365/18870	4.36222883162564e-08	2.0606338099869 7e-06	1.4934347328322 4e-06	NDUFB3/NDUFB1/NDUFB2/ACSL4/NDUFA6/FAR1/NDUFS4/NDUFA5/PDK4	9	0.246575342465753
GO:0006164	BP	GO:0006164	purine nucleotide biosynthetic process	8/39	258/18870	4.67269912400721e-08	2.1069625140978e-06	1.5270112448310 6e-06	NDUFB3/NDUFB1/NDUFB2/ACSL4/NDUFA6/NDUFS4/NDUFA5/PDK4	8	0.310077519379845
GO:0033108	BP	GO:0033108	mitochondrial respiratory chain	6/39	101/18870	5.72206295912955e-08	2.4162140376667 7e-06	1.7511398426638 4e-06	NDUFB3/NDUFB1/NDUFB2/NDU	6	0.594059405940594

			complex assembly						FA6/NDUFS4/NDUFA5		
GO:0006754	BP	GO:0006754	ATP biosynthetic process	6/39	102/18870	6.0705631037879 1e-08	2.4162140376667 7e-06	1.7511398426638 4e-06	NDUFB3/NDUFB1/NDUFB2/NDUFA6/NDUFS4/NDUFA5	6	0.588235294117647
GO:0072522	BP	GO:0072522	purine-containing compound biosynthetic process	8/39	267/18870	6.0892490868618 1e-08	2.4162140376667 7e-06	1.7511398426638 4e-06	NDUFB3/NDUFB1/NDUFB2/ACSL4/NDUFA6/NDUFS4/NDUFA5/PDK4	8	0.299625468164794
GO:0009259	BP	GO:0009259	ribonucleotide metabolic process	9/39	385/18870	6.8846892054060 3e-08	2.6267737276010 7e-06	1.9037419948551 9e-06	NDUFB3/NDUFB1/NDUFB2/ACSL4/NDUFA6/FAR1/NDUFS4/NDU	9	0.233766233766234

									FA5/PDK4		
GO:0019693	BP	GO:0019693	ribose phosphate metabolic process	9/39	393/18870	8.2048307922255 2e-08	3.0145156095880 4e-06	2.1847561134853 9e-06	NDUFB3/NDUFB1/NDUFB2/ACSL4/NDUFA6/FAR1/NDUFS4/NDUFA5/PDK4	9	0.229007633587786
GO:0009141	BP	GO:0009141	nucleoside triphosphate metabolic process	7/39	184/18870	8.8349785914999 9e-08	3.1301067009885 7e-06	2.2685302172911 6e-06	NDUFB3/NDUFB1/NDUFB2/RRM2B/NDUFA6/NDUFS4/NDUFA5	7	0.380434782608696
GO:0009206	BP	GO:0009206	purine ribonucleoside triphosphate biosynthetic process	6/39	113/18870	1.1203602572249 8e-07	3.8324047419558 1e-06	2.7775174435015 e-06	NDUFB3/NDUFB1/NDUFB2/NDUFA6/NDUFS4/NDUFA5	6	0.530973451327434

GO:0009145	BP	GO:0009145	purine nucleoside triphosphate biosynthetic process	6/39	114/18870	1.1808264957742e-07	3.9045996126933 7e-06	2.8298403389957 2e-06	NDUFB3/NDUFB1/NDUFB2/NDUFA6/NDUFS4/NDUFA5	6	0.526315789473684
GO:0009201	BP	GO:0009201	ribonucleoside triphosphate biosynthetic process	6/39	119/18870	1.5249460589689 2e-07	4.8798273887005 3e-06	3.5366321163863 1e-06	NDUFB3/NDUFB1/NDUFB2/NDUFA6/NDUFS4/NDUFA5	6	0.504201680672269
GO:0009142	BP	GO:0009142	nucleoside triphosphate biosynthetic process	6/39	127/18870	2.2445412428048 6e-07	6.9580778526950 5e-06	5.0428344369595 9e-06	NDUFB3/NDUFB1/NDUFB2/NDUFA6/NDUFS4/NDUFA5	6	0.47244094488189
GO:0006163	BP	GO:0006163	purine nucleotide metabolic process	9/39	463/18870	3.2862931699126 2e-07	9.8787964380403 7e-06	7.1596115950568 5e-06	NDUFB3/NDUFB1/NDUFB2/ACSL4/NDUFA6/FAR	9	0.19438444924406

									1/NDUFS4/NDUFA5/PDK4		
GO:0046034	BP	GO:0046034	ATP metabolic process	6/39	137/18870	3.5152129722648 7e-07	1.0256150789666 9e-05	7.4330973995569 9e-06	NDUFB3/NDUFB1/NDUFB2/NDUFA6/NDUFS4/NDUFA5	6	0.437956204379562
GO:0001516	BP	GO:0001516	prostaglandin biosynthetic process	4/39	32/18870	5.3723705402047 6e-07	1.4803865488564 2e-05	1.0729032394619 4e-05	AKR1C3/PLA2G4A/PNPLA8/PTGS2	4	1.25
GO:0046457	BP	GO:0046457	prostanoid biosynthetic process	4/39	32/18870	5.3723705402047 6e-07	1.4803865488564 2e-05	1.0729032394619 4e-05	AKR1C3/PLA2G4A/PNPLA8/PTGS2	4	1.25
GO:0072521	BP	GO:0072521	purine-containing compound metabolic process	9/39	493/18870	5.5619257952954 8e-07	1.4911974024143 6e-05	1.0807383551029 3e-05	NDUFB3/NDUFB1/NDUFB2/ACSL4/NDUFA6/FAR	9	0.182555780933063

									1/NDUFS4/NDUFA5/PDK4		
GO:0009205	BP	GO:0009205	purine ribonucleoside triphosphate metabolic process	6/39	161/18870	9.0819688987554 1e-07	2.3708718809382 5e-05	1.7182783262742 2e-05	NDUFB3/NDUFB1/NDUFB2/NDUFA6/NDUFS4/NDUFA5	6	0.3726708074534 16
GO:0009144	BP	GO:0009144	purine nucleoside triphosphate metabolic process	6/39	167/18870	1.1248427138793 8e-06	2.8611383901752 4e-05	2.0735966898235 2e-05	NDUFB3/NDUFB1/NDUFB2/NDUFA6/NDUFS4/NDUFA5	6	0.3592814371257 49
GO:0009199	BP	GO:0009199	ribonucleoside triphosphate metabolic process	6/39	168/18870	1.1647504675489 4e-06	2.8885811595213 8e-05	2.0934857087787 6e-05	NDUFB3/NDUFB1/NDUFB2/NDUFA6/NDUFS4/NDUFA5	6	0.3571428571428 57
GO:0008611	BP	GO:0008611	ether lipid biosyntheti	3/39	11/18870	1.3314122233846	3.1446688704704	2.2790840816334	PLA2G4A/LPCA	3	2.72727272727273

			611	c process			6e-06	4e-05	e-05	T2/FAR1		
GO:0046504	BP		GO:0046504	glycerol ether biosynthetic process	3/39	11/18870	1.3314122233846 6e-06	3.1446688704704 4e-05	2.2790840816334 e-05	PLA2G4A/LPCAT2/FAR1	3	2.72727272727273
GO:0097384	BP		GO:0097384	cellular lipid biosynthetic process	3/39	12/18870	1.7726769225341 8e-06	3.9965806980770 6e-05	2.8965032011742 7e-05	PLA2G4A/LPCAT2/FAR1	3	2.5
GO:1901503	BP		GO:1901503	ether biosynthetic process	3/39	12/18870	1.7726769225341 8e-06	3.9965806980770 6e-05	2.8965032011742 7e-05	PLA2G4A/LPCAT2/FAR1	3	2.5
GO:0006122	BP		GO:0006122	mitochondrial electron transport, ubiquinol to cytochrome c	3/39	14/18870	2.9245902157958 6e-06	6.3069423784119 3e-05	4.5709270420791 1e-05	UQCRQ/UQCR11/UQCRB	3	2.14285714285714
GO:0036151	BP		GO:0036151	phosphatidylcholine acyl-chain remodeling	3/39	14/18870	2.9245902157958 6e-06	6.3069423784119 3e-05	4.5709270420791 1e-05	PLA2G4A/DBI/LPCAT2	3	2.14285714285714
GO:0001676	BP		GO:0001676	long-chain fatty acid metabolic process	5/39	110/18870	3.0199282136663 9e-06	6.3739761445895 e-05	4.6195094511403 1e-05	AKR1C3/PLA2G4A/ACSL4/PNPL	5	0.454545454545455

									A8/PTGS2		
GO:0006693	BP	GO:0006693	prostaglandin metabolic process	4/39	50/18870	3.3499421440042e-06	6.9232137642753 4e-05	5.0175668516554 1e-05	AKR1C3/PLA2G4A/PNPLA8/PTGS2	4	0.8
GO:0006692	BP	GO:0006692	prostanoid metabolic process	4/39	51/18870	3.6296532265973 9e-06	7.3481959199686e-05	5.3255706847819 9e-05	AKR1C3/PLA2G4A/PNPLA8/PTGS2	4	0.7843137254901 96
GO:0006636	BP	GO:0006636	unsaturated fatty acid biosynthetic process	4/39	52/18870	3.9262935255715 5e-06	7.7897663547339 5e-05	5.6455967957165 6e-05	AKR1C3/PLA2G4A/PNPLA8/PTGS2	4	0.7692307692307 69
GO:0019369	BP	GO:0019369	arachidonic acid metabolic process	4/39	58/18870	6.0985996185505 3e-06	0.000118623741600042	8.5972002878638e-05	AKR1C3/PLA2G4A/PNPLA8/PTGS2	4	0.6896551724137 93
GO:0046456	BP	GO:0046456	icosanoid biosynthetic process	4/39	61/18870	7.4680035213484 6e-06	0.000142466528714955	0.000103251951519858	AKR1C3/PLA2G4A/PNPLA8/PTGS2	4	0.6557377049180 33

GO:0006644	BP	GO:0006644	phospholipid metabolic process	7/39	375/18870	1.0290233479362 2e-05	0.000190815595545783	0.000138292711967073	PLA2G4A/DBI/LPCAT2/PNPLA8/FAR1/SGMS2/BMX	7	0.186666666666666667
GO:0006662	BP	GO:0006662	glycerol ether metabolic process	3/39	21/18870	1.0579493704655 3e-05	0.000190815595545783	0.000138292711967073	PLA2G4A/LPCAT2/FAR1	3	1.42857142857143
GO:0046485	BP	GO:0046485	ether lipid metabolic process	3/39	21/18870	1.0579493704655 3e-05	0.000190815595545783	0.000138292711967073	PLA2G4A/LPCAT2/FAR1	3	1.42857142857143
GO:0006631	BP	GO:0006631	fatty acid metabolic process	7/39	401/18870	1.5881730280586 3e-05	0.000281333507827528	0.000203895146271437	AKR1C3/PLA2G4A/ACSL4/DBI/PNPLA8/PTGS2/PDK4	7	0.174563591022444
GO:0046470	BP	GO:0046470	phosphatidylcholine metabolic process	4/39	76/18870	1.7955971214699 9e-05	0.000312496902543549	0.000226480671092152	PLA2G4A/DBI/LPCAT2/PNPLA8	4	0.526315789473684
GO:0018904	BP	GO:0018904	ether metabolic	3/39	26/18870	2.0534312781058	0.0003512075565	0.0002545360368	PLA2G4A/LPCA	3	1.15384615384615

		904	process			2e-05	31203	32355	T2/FAR1		
GO:0006633	BP	GO:0006633	fatty acid biosynthetic process	5/39	168/18870	2.37498026774373e-05	0.000399318716203692	0.000289404375177336	AKR1C3/PLA2G4A/PNPLA8/PTGS2/PDK4	5	0.297619047619048
GO:0006650	BP	GO:0006650	glycerophospholipid metabolic process	6/39	298/18870	3.10386849058789e-05	0.000513172923777197	0.000371919680538864	PLA2G4A/DBI/LPCAT2/PNPLA8/FAR1/BMX	6	0.201342281879195
GO:0033559	BP	GO:0033559	unsaturated fatty acid metabolic process	4/39	115/18870	9.13200293258657e-05	0.00148507326379113	0.00107629991422893	AKR1C3/PLA2G4A/PNPLA8/PTGS2	4	0.347826086956522
GO:0072330	BP	GO:0072330	monocarboxylic acid biosynthetic process	5/39	226/18870	9.73937769319974e-05	0.00155830043091196	0.0011293709617072	AKR1C3/PLA2G4A/PNPLA8/PTGS2/PDK4	5	0.221238938053097
GO:0032309	BP	GO:0032309	icosanoid secretion	3/39	46/18870	0.000116509514282001	0.00183456251059913	0.00132959061411206	PLA2G4A/PNPLA8/PTGS2	3	0.652173913043478
GO:0006	BP	GO:	icosanoid	4/39	125/1887	0.000126	0.001955	0.001416	AKR1C3	4	0.32

690		0006690	metabolic process		0	13270119748	05686856094	91833746511	/PLA2G4A/PNPLA8/PTGS2		
GO:0046486	BP	GO:0046486	glycerolipid metabolic process	6/39	390/18870	0.00013740099703745	0.00209695060094077	0.00151975515751543	PLA2G4A/DBI/LPCAT2/PNPLA8/FAR1/BMX	6	0.1538461538461554
GO:0008654	BP	GO:0008654	phospholipid biosynthetic process	5/39	254/18870	0.000168277145969281	0.00252925649699283	0.00183306683727303	PLA2G4A/LPCAT2/FAR1/SGMS2/BMX	5	0.196850393700787
GO:0070542	BP	GO:0070542	response to fatty acid	3/39	56/18870	0.000209739088791338	0.00310539068777623	0.00225061740211287	AKR1C3/PTGS2/PDK4	3	0.5357142857142857146
GO:0071715	BP	GO:0071715	icosanoid transport	3/39	63/18870	0.000297479199369442	0.00433969655550715	0.00314517481686267	PLA2G4A/PNPLA8/PTGS2	3	0.476190476190476
GO:0006869	BP	GO:0006869	lipid transport	6/39	453/18870	0.000308508785697857	0.00443537268713441	0.00321451564655433	STARD3NL/PLA2G4A/DBI/PNPLA8/ABC	6	0.132450331125828

									A1/PTGS2		
GO:0120254	BP	GO:0120254	olefinic compound metabolic process	4/39	162/18870	0.000340634544190269	0.004827278111953 53	0.00349854727341284	AKR1C3/PLA2G4A/PNPLA8/PTGS2	4	0.2469135802469134
GO:0034638	BP	GO:0034638	phosphatidylcholine catabolic process	2/39	16/18870	0.00049041209664 8314	0.00685195492781869	0.0049659223426 36	PLA2G4A/PNPLA8	2	1.25
GO:0032310	BP	GO:0032310	prostaglandin secretion	2/39	17/18870	0.000555075027843351	0.0076477003836195	0.00554263514644749	PLA2G4A/PTGS2	2	1.17647058823529
GO:0046394	BP	GO:0046394	carboxylic acid biosynthetic process	5/39	334/18870	0.00059226154629343	0.00804826649209702	0.0058329435633513	AKR1C3/PLA2G4A/PNPLA8/PTGS2/PDK4	5	0.1497005988023955
GO:0016053	BP	GO:0016053	organic acid biosynthetic process	5/39	337/18870	0.000616722501325639	0.00826741515290587	0.00599177053208267	AKR1C3/PLA2G4A/PNPLA8/PTGS2/PDK4	5	0.1483679525222255
GO:0032370	BP	GO:0032370	positive regulation of lipid	3/39	86/18870	0.000741894317463329	0.00981278883898163	0.00711177289582391	PLA2G4A/DBI/ABCA1	3	0.3488372093023226

			transport								
GO:0046474	BP	GO:0046474	glycerophospholipid biosynthetic process	4/39	210/18870	0.000903615667619564	0.0117945623984027	0.0085480540302 5156	PLA2G4A/LPCAT2/FAR1/BMX	4	0.19047619047619
GO:0006637	BP	GO:0006637	acyl-CoA metabolic process	3/39	96/18870	0.00102112434267387	0.0129866070247754	0.00941198280764178	ACSL4/FAR1/PDK4	3	0.3125
GO:0035383	BP	GO:0035383	thioester metabolic process	3/39	96/18870	0.00102112434267387	0.0129866070247754	0.00941198280764178	ACSL4/FAR1/PDK4	3	0.3125
GO:0002082	BP	GO:0002082	regulation of oxidative phosphorylation	2/39	24/18870	0.00111623034383967	0.0140164620391007	0.0101583654209527	COX7A2/DNAJC15	2	0.833333333333333
GO:0035336	BP	GO:0035336	long-chain fatty-acyl-CoA metabolic process	2/39	25/18870	0.0012117120284495	0.0150252291527738	0.0108894646767238	ACSL4/FAR1	2	0.8
GO:0006979	BP	GO:0006979	response to oxidative stress	5/39	400/18870	0.00132794926658847	0.0162632799068613	0.0117867361803759	TXN/RRM2B/NDUFA6/PNPLA8/PTGS2	5	0.125
GO:0072593	BP	GO:0072593	reactive oxygen	4/39	234/18870	0.0013484860998	0.0163133928172	0.0118230552782	TXN/AKR1C3/N	4	0.17094017094017

		593	species metabolic process			1321	525	082	DUFS4/PDK4		1
GO:0015732	BP	GO:0015732	prostaglandin transport	2/39	28/18870	0.0015207949393 0276	0.0181762479492 571	0.0131731508375 876	PLA2G4A/PTGS2	2	0.714285714285714
GO:1905954	BP	GO:1905954	positive regulation of lipid localization	3/39	111/18870	0.0015517460813 4115	0.0183253822939 336	0.0132812352575 94	PLA2G4A/DBI/ABCA1	3	0.27027027027027
GO:0045017	BP	GO:0045017	glycerolipid biosynthetic process	4/39	250/18870	0.0017186786662 018	0.0200579910220 258	0.0145369353438 493	PLA2G4A/LPCAT2/FAR1/BMX	4	0.16
GO:0015908	BP	GO:0015908	fatty acid transport	3/39	116/18870	0.0017605505679 2701	0.0203077460858 557	0.0147179441602 711	PLA2G4A/PNPLA8/PTGS2	3	0.258620689655172
GO:0050482	BP	GO:0050482	arachidonic acid secretion	2/39	31/18870	0.0018635160998 5704	0.0210069087620 248	0.0152246590454 828	PLA2G4A/PNPLA8	2	0.645161290322581
GO:1903963	BP	GO:1903963	arachidonate transport	2/39	31/18870	0.0018635160998 5704	0.0210069087620 248	0.0152246590454 828	PLA2G4A/PNPLA8	2	0.645161290322581
GO:1903715	BP	GO:1903715	regulation of aerobic respiration	2/39	33/18870	0.0021104838584 5714	0.0233847701717 251	0.0169480030000 936	COX7A2/DNAJC15	2	0.606060606060606

GO:0033865	BP	GO:0033865	nucleoside bisphosphate metabolic process	3/39	125/18870	0.0021790325528756	0.0233847701717251	0.0169480030000936	ACSL4/FAR1/PDK4	3	0.24
GO:0033875	BP	GO:0033875	ribonucleoside bisphosphate metabolic process	3/39	125/18870	0.0021790325528756	0.0233847701717251	0.0169480030000936	ACSL4/FAR1/PDK4	3	0.24
GO:0034032	BP	GO:0034032	purine nucleoside bisphosphate metabolic process	3/39	125/18870	0.0021790325528756	0.0233847701717251	0.0169480030000936	ACSL4/FAR1/PDK4	3	0.24
GO:0034405	BP	GO:0034405	response to fluid shear stress	2/39	34/18870	0.00223946891765513	0.0233847701717251	0.0169480030000936	ABCA1/PTGS2	2	0.588235294117647
GO:0046475	BP	GO:0046475	glycerophospholipid catabolic process	2/39	34/18870	0.00223946891765513	0.0233847701717251	0.0169480030000936	PLA2G4A/PNPLA8	2	0.588235294117647
GO:0071398	BP	GO:0071398	cellular response to fatty acid	2/39	34/18870	0.00223946891765513	0.0233847701717251	0.0169480030000936	AKR1C3/PDK4	2	0.588235294117647

GO:0046471	BP	GO:0046471	phosphatidylglycerol metabolic process	2/39	35/18870	0.0023721015687989	0.0245117162107255	0.0177647518802266	PLA2G4A/PNPLA8	2	0.571428571428571
GO:0043467	BP	GO:0043467	regulation of generation of precursor metabolites and energy	3/39	139/18870	0.0029444010646719 4	0.030111812950047	0.021823395845588	COX7A2/NDUFA4/DNAJC15	3	0.215827338129496
GO:0035337	BP	GO:0035337	fatty-acyl-CoA metabolic process	2/39	40/18870	0.0030894607361986	0.0312729086766226	0.0226648945523485	ACSL4/FAR1	2	0.5
GO:0010565	BP	GO:0010565	regulation of cellular ketone metabolic process	3/39	144/18870	0.00325299847900848	0.0325957019310749	0.0236235827874832	AKR1C3/PTGS2/PDK4	3	0.208333333333333
GO:0031667	BP	GO:0031667	response to nutrient levels	5/39	495/18870	0.0033577736490206	0.0333091145982844	0.0241406252871692	AKR1C3/DNAJC15/ABCA1/PTGS2/PDK4	5	0.101010101010101
GO:0032368	BP	GO:0032368	regulation of lipid	3/39	153/18870	0.0038570685336	0.0378832869839	0.0274557353671	PLA2G4A/DBI/A	3	0.19607843137254

		368	transport			4631	32	749	BCA1		9
GO:0007584	BP	GO:0007584	response to nutrient	3/39	155/18870	0.0039999629011 6114	0.0389015999799 201	0.0281937529565 847	AKR1C3/ABCA1/PTGS2	3	0.193548387096774
GO:0035384	BP	GO:0035384	thioester biosynthetic process	2/39	47/18870	0.0042428459224 23	0.0400847919527 963	0.0290512658146 858	ACSL4/PDK4	2	0.425531914893617
GO:0071616	BP	GO:0071616	acyl-CoA biosynthetic process	2/39	47/18870	0.0042428459224 23	0.0400847919527 963	0.0290512658146 858	ACSL4/PDK4	2	0.425531914893617
GO:0140353	BP	GO:0140353	lipid export from cell	2/39	47/18870	0.0042428459224 23	0.0400847919527 963	0.0290512658146 858	PLA2G4A/PTGS2	2	0.425531914893617
GO:0006261	BP	GO:0006261	DNA-templated DNA replication	3/39	161/18870	0.0044479118508 0274	0.0416257410943 049	0.0301680615104 098	POLB/RRM2B/GMNN	3	0.186335403726708
GO:0043457	BP	GO:0043457	regulation of cellular respiration	2/39	49/18870	0.0046037221667 0033	0.0426812372838 012	0.0309330274457 09	COX7A2/DNAJC15	2	0.408163265306122
GO:1901570	BP	GO:1901570	fatty acid derivative biosynthetic process	2/39	52/18870	0.0051707218609 1267	0.0474940378335 682	0.0344210821735 219	ACSL4/FAR1	2	0.384615384615385
GO:0019216	BP	GO:0019216	regulation of lipid metabolic process	4/39	342/18870	0.0052894326351 7713	0.0481386896706 029	0.0348882905825 783	AKR1C3/DNAJC15/PTGS2/PDK4	4	0.116959064327485

GO:0071496	BP	GO:0071496	cellular response to external stimulus	4/39	346/18870	0.00550982536087642	0.0496886068908128	0.0360115858514698	AKR1C3/DNAJC15/PTGS2/PDK4	4	0.115606936416185
GO:0009395	BP	GO:0009395	phospholipid catabolic process	2/39	54/18870	0.00556567993716905	0.0497401306096549	0.03604892742614	PLA2G4A/PNPLA8	2	0.37037037037037
GO:0042304	BP	GO:0042304	regulation of fatty acid biosynthetic process	2/39	55/18870	0.00576820420876804	0.0510898087062312	0.0370271003250805	PTGS2/PDK4	2	0.363636363636364
GO:0009267	BP	GO:0009267	cellular response to starvation	3/39	179/18870	0.005969952852929	0.0519490634219787	0.0376498411685181	AKR1C3/DNAJC15/PDK4	3	0.167597765363128
GO:0015718	BP	GO:0015718	monocarboxylic acid transport	3/39	179/18870	0.005969952852929	0.0519490634219787	0.0376498411685181	PLA2G4A/PNPLA8/PTGS2	3	0.167597765363128
GO:1905952	BP	GO:1905952	regulation of lipid localization	3/39	183/18870	0.00634546136478909	0.0537679978308047	0.0389681053888366	PLA2G4A/DBI/ABCA1	3	0.163934426229508
GO:0033866	BP	GO:0033866	nucleoside bisphosphate biosynthetic process	2/39	58/18870	0.00639579006455137	0.0537679978308047	0.0389681053888366	ACSL4/PDK4	2	0.344827586206897
GO:0034	BP	GO:	ribonucleos	2/39	58/18870	0.006395	0.053767	0.038968	ACSL4/P	2	0.344827

030		0034030	ide bisphosphate biosynthetic process			79006455137	9978308047	1053888366	DK4		586206897
GO:0034033	BP	GO:0034033	purine nucleoside bisphosphate biosynthetic process	2/39	58/18870	0.00639579006455137	0.0537679978308047	0.0389681053888366	ACSL4/PDK4	2	0.344827586206897
GO:0046890	BP	GO:0046890	regulation of lipid biosynthetic process	3/39	189/18870	0.00693475130173566	0.0578090192548048	0.041896816798633	AKR1C3/PTGS2/PDK4	3	0.158730158730159
GO:0006066	BP	GO:0006066	alcohol metabolic process	4/39	373/18870	0.00716054222294722	0.05919381570969 7	0.0429004415637978	AKR1C3/PLA2G4A/SC5D/ABCA1	4	0.107238605898123
GO:0046503	BP	GO:0046503	glycerolipid catabolic process	2/39	65/18870	0.0079751212792403	0.0653828124711271	0.0473858880706492	PLA2G4A/PNPLA8	2	0.307692307692308
GO:0015909	BP	GO:0015909	long-chain fatty acid transport	2/39	67/18870	0.00845542681985784	0.0681933610186909	0.0494228200082405	PLA2G4A/PNPLA8	2	0.298507462686567
GO:0071806	BP	GO:0071	protein transmembr	2/39	67/18870	0.0084554268198	0.0681933610186	0.0494228200082	DNAJC15/ABCA	2	0.29850746268656

		806	ane transport			5784	909	405	1		7
GO:0042594	BP	GO:0042594	response to starvation	3/39	218/18870	0.0102355729044652	0.081884583235722	0.0593454693866703	AKR1C3/DNAJC15/PDK4	3	0.137614678899083
GO:0044242	BP	GO:0044242	cellular lipid catabolic process	3/39	223/18870	0.0108823309491221	0.086362178412233	0.0625905855852667	AKR1C3/PLA2G4A/PNPLA8	3	0.134529147982063
GO:0042180	BP	GO:0042180	cellular ketone metabolic process	3/39	226/18870	0.011281557320171	0.0884333091930245	0.0640916279486797	AKR1C3/PTGS2/PDK4	3	0.132743362831858
GO:1901568	BP	GO:1901568	fatty acid derivative metabolic process	2/39	78/18870	0.0113216030922521	0.0884333091930245	0.0640916279486797	ACSL4/FAR1	2	0.256410256410256
GO:0032414	BP	GO:0032414	positive regulation of ion transmembrane transporter activity	2/39	85/18870	0.0133377836872749	0.103367823576381	0.0749153475198091	NDUFA4/GLRX	2	0.235294117647059
GO:0031669	BP	GO:0031669	cellular response to nutrient	3/39	251/18870	0.0149385805338589	0.114876526275876	0.0832562260679368	AKR1C3/DNAJC15/PDK4	3	0.119521912350598

			levels								
GO:0097305	BP	GO:0097305	response to alcohol	3/39	254/18870	0.0154174542877701	0.11764703579590 7	0.0852641399072632	POLB/AKR1C3/ABCA1	3	0.1181102362204722
GO:0032411	BP	GO:0032411	positive regulation of transporter activity	2/39	94/18870	0.0161413380523033	0.1222305904418 7	0.0885860497366266	NDUFA4/GLRX	2	0.2127659574468 09
GO:0042158	BP	GO:0042158	lipoprotein biosynthetic process	2/39	97/18870	0.0171272428078617	0.1287138247378 7	0.0932847435228829	DBI/ABCA1	2	0.2061855670103 09
GO:0023061	BP	GO:0023061	signal release	4/39	484/18870	0.0173081026595458	0.129095021340371	0.0935610139807657	PLA2G4A/ACSL4/ABCA1/PTGS2	4	0.0826446280991736
GO:0098869	BP	GO:0098869	cellular oxidant detoxification	2/39	99/18870	0.0177984957587829	0.131334483126793	0.0951840534545835	TXN/PTGS2	2	0.2020202020202 02
GO:0019217	BP	GO:0019217	regulation of fatty acid metabolic process	2/39	100/18870	0.0181382801768934	0.131334483126793	0.0951840534545835	PTGS2/PDK4	2	0.2
GO:0015914	BP	GO:0015914	phospholipid transport	2/39	103/18870	0.0191741182262435	0.131334483126793	0.0951840534545835	DBI/ABCA1	2	0.1941747572815 53

GO:0097306	BP	GO:0097306	cellular response to alcohol	2/39	103/18870	0.0191741182262435	0.131334483126793	0.0951840534545835	AKR1C3/ABCA1	2	0.194174757281553
GO:0033138	BP	GO:0033138	positive regulation of peptidyl-serine phosphorylation	2/39	104/18870	0.0195248518940605	0.131334483126793	0.0951840534545835	TXN/PTGS2	2	0.192307692307692
GO:0006260	BP	GO:0006260	DNA replication	3/39	278/18870	0.0195613068715057	0.131334483126793	0.0951840534545835	POLB/RRM2B/GMNN	3	0.107913669064748
GO:0031668	BP	GO:0031668	cellular response to extracellular stimulus	3/39	281/18870	0.0201185989366083	0.131334483126793	0.0951840534545835	AKR1C3/DNAJC15/PDK4	3	0.106761565836299
GO:0006547	BP	GO:0006547	histidine metabolic process	1/39	10/18870	0.0204814018122945	0.131334483126793	0.0951840534545835	HNMT	1	1
GO:0006591	BP	GO:0006591	ornithine metabolic process	1/39	10/18870	0.0204814018122945	0.131334483126793	0.0951840534545835	ARG1	1	1
GO:0008300	BP	GO:0008300	isoprenoid catabolic process	1/39	10/18870	0.0204814018122945	0.131334483126793	0.0951840534545835	AKR1C3	1	1
GO:0008	BP	GO:	intrinsic	1/39	10/18870	0.020481	0.131334	0.095184	PTGS2	1	1

627		0008627	apoptotic signaling pathway in response to osmotic stress			4018122945	483126793	0534545835			
GO:0010572	BP	GO:0010572	positive regulation of platelet activation	1/39	10/18870	0.0204814018122945	0.13133448312679 3	0.0951840534545835	PLA2G4A	1	1
GO:0016102	BP	GO:0016102	diterpenoid biosynthetic process	1/39	10/18870	0.0204814018122945	0.13133448312679 3	0.0951840534545835	AKR1C3	1	1
GO:0030638	BP	GO:0030638	polyketide metabolic process	1/39	10/18870	0.0204814018122945	0.13133448312679 3	0.0951840534545835	AKR1C3	1	1
GO:0030647	BP	GO:0030647	aminoglycoside antibiotic metabolic process	1/39	10/18870	0.0204814018122945	0.13133448312679 3	0.0951840534545835	AKR1C3	1	1
GO:0042135	BP	GO:0042135	neurotransmitter catabolic process	1/39	10/18870	0.0204814018122945	0.13133448312679 3	0.0951840534545835	HNMT	1	1
GO:0044598	BP	GO:0044	doxorubicin metabolic	1/39	10/18870	0.0204814018122	0.1313344831267	0.0951840534545	AKR1C3	1	1

		598	process			945	93	835			
GO:1901856	BP	GO:1901856	negative regulation of cellular respiration	1/39	10/18870	0.0204814018122945	0.131334483126793	0.0951840534545835	DNAJC15	1	1
GO:1905918	BP	GO:1905918	regulation of CoA-transferase activity	1/39	10/18870	0.0204814018122945	0.131334483126793	0.0951840534545835	DBI	1	1
GO:0006766	BP	GO:0006766	vitamin metabolic process	2/39	109/18870	0.0213188834155157	0.131334483126793	0.0951840534545835	MTHFD2/AKR1C3	2	0.18348623853211
GO:0046822	BP	GO:0046822	regulation of nucleocytoplasmic transport	2/39	109/18870	0.0213188834155157	0.131334483126793	0.0951840534545835	TXN/PTGS2	2	0.18348623853211
GO:0000050	BP	GO:0000050	urea cycle	1/39	11/18870	0.0225069174713254	0.131334483126793	0.0951840534545835	ARG1	1	0.909090909090909
GO:0001660	BP	GO:0001660	fever generation	1/39	11/18870	0.0225069174713254	0.131334483126793	0.0951840534545835	PTGS2	1	0.909090909090909
GO:0002725	BP	GO:0002725	negative regulation of T cell	1/39	11/18870	0.0225069174713254	0.131334483126793	0.0951840534545835	ARG1	1	0.909090909090909

			cytokine production								
GO:0006086	BP	GO:0006086	acetyl-CoA biosynthetic process from pyruvate	1/39	11/18870	0.0225069174713254	0.131334483126793	0.0951840534545835	PDK4	1	0.9090909090909090 9
GO:0006527	BP	GO:0006527	arginine catabolic process	1/39	11/18870	0.0225069174713254	0.131334483126793	0.0951840534545835	ARG1	1	0.9090909090909090 9
GO:0006686	BP	GO:0006686	sphingomyelin biosynthetic process	1/39	11/18870	0.0225069174713254	0.131334483126793	0.0951840534545835	SGMS2	1	0.9090909090909090 9
GO:0010887	BP	GO:0010887	negative regulation of cholesterol storage	1/39	11/18870	0.0225069174713254	0.131334483126793	0.0951840534545835	ABCA1	1	0.9090909090909090 9
GO:0021548	BP	GO:0021548	pons development	1/39	11/18870	0.0225069174713254	0.131334483126793	0.0951840534545835	UQCRQ	1	0.9090909090909090 9
GO:0021860	BP	GO:0021860	pyramidal neuron development	1/39	11/18870	0.0225069174713254	0.131334483126793	0.0951840534545835	UQCRQ	1	0.9090909090909090 9
GO:0031	BP	GO:	positive	1/39	11/18870	0.022506	0.131334	0.095184	PTGS2	1	0.909090

915		0031 915	regulation of synaptic plasticity			9174713 254	4831267 93	0534545 835			90909090 9
GO:0035 561	BP	GO: 0035 561	regulation of chromatin binding	1/39	11/18870	0.022506 9174713 254	0.131334 4831267 93	0.095184 0534545 835	GMNN	1	0.909090 90909090 9
GO:0051 709	BP	GO: 0051 709	regulation of killing of cells of another organism	1/39	11/18870	0.022506 9174713 254	0.131334 4831267 93	0.095184 0534545 835	ARG1	1	0.909090 90909090 9
GO:0061 370	BP	GO: 0061 370	testosterone biosyntheti c process	1/39	11/18870	0.022506 9174713 254	0.131334 4831267 93	0.095184 0534545 835	AKR1C3	1	0.909090 90909090 9
GO:0097 250	BP	GO: 0097 250	mitochondr ial respirasom e assembly	1/39	11/18870	0.022506 9174713 254	0.131334 4831267 93	0.095184 0534545 835	COX7A2	1	0.909090 90909090 9
GO:1903 059	BP	GO: 1903 059	regulation of protein lipidation	1/39	11/18870	0.022506 9174713 254	0.131334 4831267 93	0.095184 0534545 835	DBI	1	0.909090 90909090 9
GO:2000 169	BP	GO: 2000 169	regulation of peptidyl-cy steine S-nitrosylat	1/39	11/18870	0.022506 9174713 254	0.131334 4831267 93	0.095184 0534545 835	TXN	1	0.909090 90909090 9

			ion								
GO:0009063	BP	GO:0009063	amino acid catabolic process	2/39	113/18870	0.0228018258718229	0.13227725885876 2	0.0958673257645741	HNMT/ARG1	2	0.1769911504424778
GO:2001243	BP	GO:2001243	negative regulation of intrinsic apoptotic signaling pathway	2/39	114/18870	0.0231790898971303	0.133684053360194	0.0968868935112608	RRM2B/PTGS2	2	0.1754385964912288
GO:0016446	BP	GO:0016446	somatic hypermutation of immunoglobulin genes	1/39	12/18870	0.0245283518113555	0.134421716077922	0.0974215111218385	POLB	1	0.8333333333333333
GO:0030656	BP	GO:0030656	regulation of vitamin metabolic process	1/39	12/18870	0.0245283518113555	0.134421716077922	0.0974215111218385	AKR1C3	1	0.8333333333333333
GO:0031650	BP	GO:0031650	regulation of heat generation	1/39	12/18870	0.0245283518113555	0.134421716077922	0.0974215111218385	PTGS2	1	0.8333333333333333
GO:0032351	BP	GO:0032351	negative regulation of hormone metabolic process	1/39	12/18870	0.0245283518113555	0.134421716077922	0.0974215111218385	AKR1C3	1	0.8333333333333333

GO:0070943	BP	GO:0070943	neutrophil-mediated killing of symbiont cell	1/39	12/18870	0.0245283518113555	0.134421716077922	0.0974215111218385	ARG1	1	0.833333333333333
GO:0071236	BP	GO:0071236	cellular response to antibiotic	1/39	12/18870	0.0245283518113555	0.134421716077922	0.0974215111218385	PLA2G4A	1	0.833333333333333
GO:1990748	BP	GO:1990748	cellular detoxification	2/39	119/18870	0.025103993244378	0.134421716077922	0.0974215111218385	TXN/PTGS2	2	0.168067226890756
GO:0002566	BP	GO:0002566	somatic diversification of immune receptors via somatic mutation	1/39	13/18870	0.0265457128400625	0.134421716077922	0.0974215111218385	POLB	1	0.769230769230769
GO:0006264	BP	GO:0006264	mitochondrial DNA replication	1/39	13/18870	0.0265457128400625	0.134421716077922	0.0974215111218385	RRM2B	1	0.769230769230769
GO:0019627	BP	GO:0019627	urea metabolic process	1/39	13/18870	0.0265457128400625	0.134421716077922	0.0974215111218385	ARG1	1	0.769230769230769
GO:0021794	BP	GO:0021794	thalamus development	1/39	13/18870	0.0265457128400625	0.134421716077922	0.0974215111218385	UQCRQ	1	0.769230769230769

GO:0031392	BP	GO:0031392	regulation of prostaglandin biosynthetic process	1/39	13/18870	0.0265457128400625	0.134421716077922	0.09742151112183 85	PTGS2	1	0.769230769230769
GO:0032306	BP	GO:0032306	regulation of prostaglandin secretion	1/39	13/18870	0.0265457128400625	0.134421716077922	0.0974215111218385	PLA2G4A	1	0.769230769230769
GO:0032308	BP	GO:0032308	positive regulation of prostaglandin secretion	1/39	13/18870	0.0265457128400625	0.134421716077922	0.0974215111218385	PLA2G4A	1	0.769230769230769
GO:0034380	BP	GO:0034380	high-density lipoprotein particle assembly	1/39	13/18870	0.0265457128400625	0.134421716077922	0.0974215111218385	ABCA1	1	0.769230769230769
GO:0035745	BP	GO:0035745	T-helper 2 cell cytokine production	1/39	13/18870	0.0265457128400625	0.134421716077922	0.0974215111218385	ARG1	1	0.769230769230769
GO:0045986	BP	GO:0045986	negative regulation of smooth	1/39	13/18870	0.0265457128400625	0.134421716077922	0.0974215111218385	PTGS2	1	0.769230769230769

			muscle contraction								
GO:0071941	BP	GO:0071941	nitrogen cycle metabolic process	1/39	13/18870	0.0265457128400625	0.134421716077922	0.0974215111218385	ARG1	1	0.769230769230769
GO:0106049	BP	GO:0106049	regulation of cellular response to osmotic stress	1/39	13/18870	0.0265457128400625	0.134421716077922	0.0974215111218385	PTGS2	1	0.769230769230769
GO:2000551	BP	GO:2000551	regulation of T-helper 2 cell cytokine production	1/39	13/18870	0.0265457128400625	0.134421716077922	0.0974215111218385	ARG1	1	0.769230769230769
GO:0030282	BP	GO:0030282	bone mineralization	2/39	123/18870	0.0266895184695273	0.134421716077922	0.0974215111218385	SGMS2/PTGS2	2	0.16260162601626
GO:0006790	BP	GO:0006790	sulfur compound metabolic process	3/39	317/18870	0.0274912807420568	0.134421716077922	0.0974215111218385	ACSL4/FAR1/PDK4	3	0.0946372239747634
GO:0062197	BP	GO:0062197	cellular response to chemical stress	3/39	317/18870	0.0274912807420568	0.134421716077922	0.0974215111218385	TXN/PNPLA8/PTGS2	3	0.0946372239747634

GO:0003014	BP	GO:0003014	renal system process	2/39	126/18870	0.0279047805068 2	0.134421716077922	0.09742151112183 85	AKR1C3/RRM2B	2	0.158730158730159
GO:0097193	BP	GO:0097193	intrinsic apoptotic signaling pathway	3/39	319/18870	0.0279379810507121	0.134421716077922	0.0974215111218385	POLB/RRM2B/PTGS2	3	0.09404388714733 54
GO:0046683	BP	GO:0046683	response to organophosphorus	2/39	127/18870	0.0283147819517676	0.134421716077922	0.0974215111218385	NDUFS4/PTGS2	2	0.15748031496063
GO:0051099	BP	GO:0051099	positive regulation of binding	2/39	127/18870	0.0283147819517676	0.134421716077922	0.0974215111218385	TXN/GMNN	2	0.15748031496063
GO:0006287	BP	GO:0006287	base-excision repair, gap-filling	1/39	14/18870	0.0285590085498381	0.134421716077922	0.0974215111218385	POLB	1	0.714285714285714
GO:0010745	BP	GO:0010745	negative regulation of macrophage derived foam cell differentiation	1/39	14/18870	0.0285590085498381	0.134421716077922	0.0974215111218385	ABCA1	1	0.714285714285714
GO:0021859	BP	GO:0021859	pyramidal neuron differentiati	1/39	14/18870	0.0285590085498381	0.134421716077922	0.0974215111218385	UQCRQ	1	0.714285714285714

			on								
GO:0033700	BP	GO:0033700	phospholipid efflux	1/39	14/18870	0.0285590085498381	0.134421716077922	0.0974215111218385	ABCA1	1	0.714285714285714
GO:0034616	BP	GO:0034616	response to laminar fluid shear stress	1/39	14/18870	0.0285590085498381	0.134421716077922	0.0974215111218385	ABCA1	1	0.714285714285714
GO:0042762	BP	GO:0042762	regulation of sulfur metabolic process	1/39	14/18870	0.0285590085498381	0.134421716077922	0.0974215111218385	PDK4	1	0.714285714285714
GO:0046462	BP	GO:0046462	monoacylglycerol metabolic process	1/39	14/18870	0.0285590085498381	0.134421716077922	0.0974215111218385	PLA2G4A	1	0.714285714285714
GO:0060330	BP	GO:0060330	regulation of response to type II interferon	1/39	14/18870	0.0285590085498381	0.134421716077922	0.0974215111218385	ARG1	1	0.714285714285714
GO:0060334	BP	GO:0060334	regulation of type II interferon-mediated signaling pathway	1/39	14/18870	0.0285590085498381	0.134421716077922	0.0974215111218385	ARG1	1	0.714285714285714
GO:0070	BP	GO:	neutrophil	1/39	14/18870	0.028559	0.134421	0.097421	ARG1	1	0.714285

942		0070942	mediated cytotoxicity			0085498381	716077922	5111218385			714285714
GO:2001140	BP	GO:2001140	positive regulation of phospholipid transport	1/39	14/18870	0.0285590085498381	0.134421716077922	0.0974215111218385	DBI	1	0.714285714285714
GO:0030301	BP	GO:0030301	cholesterol transport	2/39	128/18870	0.0287272215811688	0.134421716077922	0.0974215111218385	STARD3NL/ABCA1	2	0.15625
GO:0097237	BP	GO:0097237	cellular response to toxic substance	2/39	128/18870	0.0287272215811688	0.134421716077922	0.0974215111218385	TXN/PTGS2	2	0.15625
GO:0008202	BP	GO:0008202	steroid metabolic process	3/39	327/18870	0.0297637668294802	0.134772004188762	0.0976753807946993	AKR1C3/SC5D/ABCA1	3	0.0917431192660551
GO:0002829	BP	GO:0002829	negative regulation of type 2 immune response	1/39	15/18870	0.030568246917814	0.134772004188762	0.0976753807946993	ARG1	1	0.666666666666667
GO:0009263	BP	GO:0009263	deoxyribonucleotide biosynthetic process	1/39	15/18870	0.030568246917814	0.134772004188762	0.0976753807946993	RRM2B	1	0.666666666666667
GO:0009	BP	GO:	2'-deoxyrib	1/39	15/18870	0.030568	0.134772	0.097675	RRM2B	1	0.666666

265		0009265	onucleotide biosynthetic process			246917814	004188762	3807946993			666666667
GO:0030174	BP	GO:0030174	regulation of DNA-templated DNA replication initiation	1/39	15/18870	0.030568246917814	0.134772004188762	0.0976753807946993	GMNN	1	0.6666666666666667
GO:0034310	BP	GO:0034310	primary alcohol catabolic process	1/39	15/18870	0.030568246917814	0.134772004188762	0.0976753807946993	AKR1C3	1	0.6666666666666667
GO:0045838	BP	GO:0045838	positive regulation of membrane potential	1/39	15/18870	0.030568246917814	0.134772004188762	0.0976753807946993	GLRX	1	0.6666666666666667
GO:0046007	BP	GO:0046007	negative regulation of activated T cell proliferation	1/39	15/18870	0.030568246917814	0.134772004188762	0.0976753807946993	ARG1	1	0.6666666666666667
GO:0046385	BP	GO:0046385	deoxyribose phosphate biosyntheti	1/39	15/18870	0.030568246917814	0.134772004188762	0.0976753807946993	RRM2B	1	0.6666666666666667

			c process								
GO:0046655	BP	GO:0046655	folic acid metabolic process	1/39	15/18870	0.030568246917814	0.13477200418876 2	0.0976753807946993	MTHFD2	1	0.6666666666666667
GO:2000651	BP	GO:2000651	positive regulation of sodium ion transmembrane transporter activity	1/39	15/18870	0.030568246917814	0.134772004188762	0.0976753807946993	GLRX	1	0.6666666666666667
GO:2001138	BP	GO:2001138	regulation of phospholipid transport	1/39	15/18870	0.030568246917814	0.134772004188762	0.0976753807946993	DBI	1	0.6666666666666667
GO:2001279	BP	GO:2001279	regulation of unsaturated fatty acid biosynthetic process	1/39	15/18870	0.030568246917814	0.134772004188762	0.0976753807946993	PTGS2	1	0.6666666666666667
GO:0016042	BP	GO:0016042	lipid catabolic process	3/39	334/18870	0.0314124125376916	0.135768270666575	0.0983974202729953	AKR1C3/PLA2G4A/PNPLA8	3	0.0898203592814371
GO:0042	BP	GO:	lipoprotein	2/39	135/1887	0.031681	0.135768	0.098397	DBI/AB	2	0.148148

157		0042157	metabolic process		0	6259320096	270666575	4202729953	CA1		148148148
GO:0062012	BP	GO:0062012	regulation of small molecule metabolic process	3/39	337/18870	0.032133539628328	0.135768270666575	0.0983974202729953	AKR1C3/PTGS2/PDK4	3	0.0890207715133531
GO:0016114	BP	GO:0016114	terpenoid biosynthetic process	1/39	16/18870	0.032573435905892	0.135768270666575	0.0983974202729953	AKR1C3	1	0.625
GO:0017014	BP	GO:0017014	protein nitrosylation	1/39	16/18870	0.032573435905892	0.135768270666575	0.0983974202729953	TXN	1	0.625
GO:0018119	BP	GO:0018119	peptidyl-cysteine S-nitrosylation	1/39	16/18870	0.032573435905892	0.135768270666575	0.0983974202729953	TXN	1	0.625
GO:0032048	BP	GO:0032048	cardiolipin metabolic process	1/39	16/18870	0.032573435905892	0.135768270666575	0.0983974202729953	PNPLA8	1	0.625
GO:0042448	BP	GO:0042448	progesterone metabolic process	1/39	16/18870	0.032573435905892	0.135768270666575	0.0983974202729953	AKR1C3	1	0.625
GO:0046653	BP	GO:0046653	tetrahydrofolate metabolic process	1/39	16/18870	0.032573435905892	0.135768270666575	0.0983974202729953	MTHFD2	1	0.625

GO:0047484	BP	GO:0047484	regulation of response to osmotic stress	1/39	16/18870	0.032573435905892	0.135768270666575	0.0983974202729953	PTGS2	1	0.625
GO:0048385	BP	GO:0048385	regulation of retinoic acid receptor signaling pathway	1/39	16/18870	0.032573435905892	0.135768270666575	0.0983974202729953	AKR1C3	1	0.625
GO:0055091	BP	GO:0055091	phospholipid homeostasis	1/39	16/18870	0.032573435905892	0.135768270666575	0.0983974202729953	ABCA1	1	0.625
GO:0090336	BP	GO:0090336	positive regulation of brown fat cell differentiation	1/39	16/18870	0.032573435905892	0.135768270666575	0.0983974202729953	PTGS2	1	0.625
GO:0033135	BP	GO:0033135	regulation of peptidyl-serine phosphorylation	2/39	138/18870	0.0329832558791284	0.136901212686591	0.0992185147123747	TXN/PTGS2	2	0.144927536231884
GO:0008	BP	GO:	cholesterol	2/39	140/1887	0.033862	0.139422	0.101045	SC5D/A	2	0.142857

203		0008<br>203	metabolic<br>process		0	6079333<br>124	7105409<br>9	9585096<br>52	BCA1		14285714<br>3
GO:0015<br>918	BP	GO:<br>0015<br>918	sterol<br>transport	2/39	141/1887<br>0	0.034305<br>7308928<br>362	0.139422<br>7105409<br>9	0.101045<br>9585096<br>52	STARD3<br>NL/ABC<br>A1	2	0.141843<br>97163120<br>6
GO:0010<br>155	BP	GO:<br>0010<br>155	regulation<br>of proton<br>transport	1/39	17/18870	0.034574<br>5834607<br>698	0.139422<br>7105409<br>9	0.101045<br>9585096<br>52	NDUFA4	1	0.588235<br>29411764<br>7
GO:0031<br>649	BP	GO:<br>0031<br>649	heat<br>generation	1/39	17/18870	0.034574<br>5834607<br>698	0.139422<br>7105409<br>9	0.101045<br>9585096<br>52	PTGS2	1	0.588235<br>29411764<br>7
GO:0032<br>488	BP	GO:<br>0032<br>488	Cdc42<br>protein<br>signal<br>transductio<br>n	1/39	17/18870	0.034574<br>5834607<br>698	0.139422<br>7105409<br>9	0.101045<br>9585096<br>52	ABCA1	1	0.588235<br>29411764<br>7
GO:0033<br>151	BP	GO:<br>0033<br>151	V(D)J<br>recombinati<br>on	1/39	17/18870	0.034574<br>5834607<br>698	0.139422<br>7105409<br>9	0.101045<br>9585096<br>52	POLB	1	0.588235<br>29411764<br>7
GO:0050<br>746	BP	GO:<br>0050<br>746	regulation<br>of<br>lipoprotein<br>metabolic<br>process	1/39	17/18870	0.034574<br>5834607<br>698	0.139422<br>7105409<br>9	0.101045<br>9585096<br>52	DBI	1	0.588235<br>29411764<br>7
GO:0014<br>074	BP	GO:<br>0014<br>074	response to<br>purine-cont<br>aining	2/39	143/1887<br>0	0.035198<br>8165167<br>426	0.140073<br>8375824<br>72	0.101517<br>8597929	NDUFS4<br>/PTGS2	2	0.139860<br>13986014

			compound								
GO:0046942	BP	GO:0046942	carboxylic acid transport	3/39	351/18870	0.0356139390881683	0.14007383758247 2	0.1015178597929	PLA2G4A/PNPLA8/PTGS2	3	0.08547008547008 55
GO:0015849	BP	GO:0015849	organic acid transport	3/39	352/18870	0.0358697735686078	0.1400738375824 72	0.1015178597929	PLA2G4A/PNPLA8/PTGS2	3	0.0852272727272727
GO:0046434	BP	GO:0046434	organophosphate catabolic process	2/39	146/18870	0.0365553827211494	0.140073837582472	0.1015178597929	PLA2G4A/PNPLA8	2	0.136986301369863
GO:0002827	BP	GO:0002827	positive regulation of T-helper 1 type immune response	1/39	18/18870	0.0365716975139722	0.140073837582472	0.1015178597929	PLA2G4A	1	0.5555555555555556
GO:0006085	BP	GO:0006085	acetyl-CoA biosynthetic process	1/39	18/18870	0.0365716975139722	0.140073837582472	0.1015178597929	PDK4	1	0.5555555555555556
GO:0010042	BP	GO:0010042	response to manganese ion	1/39	18/18870	0.0365716975139722	0.140073837582472	0.1015178597929	PTGS2	1	0.5555555555555556
GO:0035065	BP	GO:0035065	regulation of histone	1/39	18/18870	0.0365716975139	0.1400738375824	0.1015178597929	NFYB	1	0.55555555555555

		065	acetylation			722	72				6
GO:0042402	BP	GO:0042402	cellular biogenic amine catabolic process	1/39	18/18870	0.0365716975139722	0.140073837582472	0.1015178597929	HNMT	1	0.555555555555556
GO:0042574	BP	GO:0042574	retinal metabolic process	1/39	18/18870	0.0365716975139722	0.140073837582472	0.1015178597929	AKR1C3	1	0.555555555555556
GO:0048535	BP	GO:0048535	lymph node development	1/39	18/18870	0.0365716975139722	0.140073837582472	0.1015178597929	POLB	1	0.555555555555556
GO:0071318	BP	GO:0071318	cellular response to ATP	1/39	18/18870	0.0365716975139722	0.140073837582472	0.1015178597929	PTGS2	1	0.555555555555556
GO:2000811	BP	GO:2000811	negative regulation of anoikis	1/39	18/18870	0.0365716975139722	0.140073837582472	0.1015178597929	PDK4	1	0.555555555555556
GO:0031960	BP	GO:0031960	response to corticosteroid	2/39	149/18870	0.0379320176236306	0.142747267515011	0.103455415654448	AKR1C3/PTGS2	2	0.134228187919463
GO:1902652	BP	GO:1902652	secondary alcohol metabolic process	2/39	150/18870	0.0383953060775468	0.142747267515011	0.103455415654448	SC5D/ABCA1	2	0.133333333333333
GO:0006684	BP	GO:0006684	sphingomyelin	1/39	19/18870	0.0385647859818	0.1427472675150	0.1034554156544	SGMS2	1	0.52631578947368

		684	metabolic process			778	11	48			4
GO:0010885	BP	GO:0010885	regulation of cholesterol storage	1/39	19/18870	0.0385647859818778	0.142747267515011	0.103455415654448	ABCA1	1	0.5263157894736840
GO:0032305	BP	GO:0032305	positive regulation of icosanoid secretion	1/39	19/18870	0.0385647859818778	0.142747267515011	0.103455415654448	PLA2G4A	1	0.5263157894736840
GO:0035338	BP	GO:0035338	long-chain fatty-acyl-CoA biosynthetic process	1/39	19/18870	0.0385647859818778	0.142747267515011	0.103455415654448	ACSL4	1	0.5263157894736840
GO:0035743	BP	GO:0035743	CD4-positive, alpha-beta T cell cytokine production	1/39	19/18870	0.0385647859818778	0.142747267515011	0.103455415654448	ARG1	1	0.5263157894736840
GO:0035821	BP	GO:0035821	modulation of process of another organism	1/39	19/18870	0.0385647859818778	0.142747267515011	0.103455415654448	ARG1	1	0.5263157894736840
GO:1902	BP	GO:	positive	1/39	19/18870	0.038564	0.142747	0.103455	GLRX	1	0.526315

307		1902307	regulation of sodium ion transmembrane transport			7859818778	267515011	415654448			789473684
GO:0015748	BP	GO:0015748	organophosphate ester transport	2/39	152/18870	0.0393284349347758	0.144191490722654	0.104502109681211	DBI/ABCA1	2	0.131578947368421
GO:0016125	BP	GO:0016125	sterol metabolic process	2/39	154/18870	0.0402702298420102	0.144191490722654	0.104502109681211	SC5D/ABCA1	2	0.12987012987013
GO:0006071	BP	GO:0006071	glycerol metabolic process	1/39	20/18870	0.0405538567657465	0.144191490722654	0.104502109681211	PLA2G4A	1	0.5
GO:0006525	BP	GO:0006525	arginine metabolic process	1/39	20/18870	0.0405538567657465	0.144191490722654	0.104502109681211	ARG1	1	0.5
GO:0009310	BP	GO:0009310	amine catabolic process	1/39	20/18870	0.0405538567657465	0.144191490722654	0.104502109681211	HNMT	1	0.5
GO:0030150	BP	GO:0030150	protein import into mitochondrial matrix	1/39	20/18870	0.0405538567657465	0.144191490722654	0.104502109681211	DNAJC15	1	0.5
GO:0032303	BP	GO:0032	regulation of	1/39	20/18870	0.0405538567657	0.1441914907226	0.1045021096812	PLA2G4A	1	0.5

		303	icosanoid secretion			465	54	11			
GO:0043691	BP	GO:0043691	reverse cholesterol transport	1/39	20/18870	0.0405538567657465	0.144191490722654	0.104502109681211	ABCA1	1	0.5
GO:0055093	BP	GO:0055093	response to hyperoxia	1/39	20/18870	0.0405538567657465	0.144191490722654	0.104502109681211	POLB	1	0.5
GO:0071397	BP	GO:0071397	cellular response to cholesterol	1/39	20/18870	0.0405538567657465	0.144191490722654	0.104502109681211	ABCA1	1	0.5
GO:0071731	BP	GO:0071731	response to nitric oxide	1/39	20/18870	0.0405538567657465	0.144191490722654	0.104502109681211	TXN	1	0.5
GO:0044282	BP	GO:0044282	small molecule catabolic process	3/39	375/18870	0.0420181143104921	0.145512435895636	0.105459458527928	HNMT/AKR1C3/ARG1	3	0.08
GO:0034767	BP	GO:0034767	positive regulation of monoatomic ion transmembrane transport	2/39	158/18870	0.0421794846719685	0.145512435895636	0.105459458527928	NDUFA4/GLRX	2	0.126582278481013
GO:0098	BP	GO:	detoxificati	2/39	158/1887	0.042179	0.145512	0.105459	TXN/PT	2	0.126582

754		0098754	on		0	4846719685	435895636	458527928	GS2		278481013
GO:0002888	BP	GO:0002888	positive regulation of myeloid leukocyte mediated immunity	1/39	21/18870	0.0425389177517485	0.145512435895636	0.105459458527928	ARG1	1	0.476190476190476
GO:0010288	BP	GO:0010288	response to lead ion	1/39	21/18870	0.0425389177517485	0.145512435895636	0.105459458527928	PTGS2	1	0.476190476190476
GO:0010566	BP	GO:0010566	regulation of ketone biosynthetic process	1/39	21/18870	0.0425389177517485	0.145512435895636	0.105459458527928	AKR1C3	1	0.476190476190476
GO:0016137	BP	GO:0016137	glycoside metabolic process	1/39	21/18870	0.0425389177517485	0.145512435895636	0.105459458527928	AKR1C3	1	0.476190476190476
GO:0019370	BP	GO:0019370	leukotriene biosynthetic process	1/39	21/18870	0.0425389177517485	0.145512435895636	0.105459458527928	PLA2G4A	1	0.476190476190476
GO:0043651	BP	GO:0043651	linoleic acid metabolic process	1/39	21/18870	0.0425389177517485	0.145512435895636	0.105459458527928	PNPLA8	1	0.476190476190476
GO:0071379	BP	GO:0071	cellular response to	1/39	21/18870	0.0425389177517	0.1455124358956	0.1054594585279	AKR1C3	1	0.47619047619047

		379	prostaglandin stimulus			485	36	28			6
GO:0071498	BP	GO:0071498	cellular response to fluid shear stress	1/39	21/18870	0.0425389177517485	0.145512435895636	0.105459458527928	PTGS2	1	0.476190476190476
GO:1902600	BP	GO:1902600	proton transmembrane transport	2/39	160/18870	0.0431467792988871	0.1470845534862 4	0.106598843411611	NDUFA4/COX7B	2	0.125
GO:0044272	BP	GO:0044272	sulfur compound biosynthetic process	2/39	161/18870	0.0436335575057812	0.1482345515264 9	0.107432299122021	ACSL4/PDK4	2	0.124223602484472
GO:0010888	BP	GO:0010888	negative regulation of lipid storage	1/39	22/18870	0.044519976810992	0.14869972052695	0.107769428183262	ABCA1	1	0.454545454545455
GO:0019400	BP	GO:0019400	alditol metabolic process	1/39	22/18870	0.044519976810992	0.14869972052695	0.107769428183262	PLA2G4A	1	0.454545454545455
GO:0032042	BP	GO:0032042	mitochondrial DNA metabolic process	1/39	22/18870	0.044519976810992	0.14869972052695	0.107769428183262	RRM2B	1	0.454545454545455
GO:0061687	BP	GO:0061	detoxification of	1/39	22/18870	0.0445199768109	0.1486997205269	0.1077694281832	TXN	1	0.45454545454545

		687	inorganic compound			92	5	62			5
GO:1902644	BP	GO:1902644	tertiary alcohol metabolic process	1/39	22/18870	0.044519976810992	0.14869972052695	0.107769428183262	AKR1C3	1	0.454545454545455
GO:0045932	BP	GO:0045932	negative regulation of muscle contraction	1/39	23/18870	0.0464970417995514	0.151727189030115	0.109963571845892	PTGS2	1	0.434782608695652
GO:0046823	BP	GO:0046823	negative regulation of nucleocytoplasmic transport	1/39	23/18870	0.0464970417995514	0.151727189030115	0.109963571845892	TXN	1	0.434782608695652
GO:0051457	BP	GO:0051457	maintenance of protein location in nucleus	1/39	23/18870	0.0464970417995514	0.151727189030115	0.109963571845892	TXN	1	0.434782608695652
GO:0071636	BP	GO:0071636	positive regulation of transforming growth factor beta production	1/39	23/18870	0.0464970417995514	0.151727189030115	0.109963571845892	PTGS2	1	0.434782608695652

GO:0097164	BP	GO:0097164	ammonium ion metabolic process	1/39	23/18870	0.0464970417995514	0.151727189030115	0.10996357184589 2	HNMT	1	0.4347826086956 52
GO:1902254	BP	GO:1902254	negative regulation of intrinsic apoptotic signaling pathway by p53 class mediator	1/39	23/18870	0.0464970417995514	0.15172718903011 5	0.10996357184589 2	RRM2B	1	0.4347826086956 52
GO:2000193	BP	GO:2000193	positive regulation of fatty acid transport	1/39	23/18870	0.0464970417995514	0.15172718903011 5	0.10996357184589 2	PLA2G4A	1	0.4347826086956 52
GO:0016093	BP	GO:0016093	polyprenol metabolic process	1/39	24/18870	0.0484701205584958	0.15606736450627 1	0.11310909375825 9	AKR1C3	1	0.4166666666666 67
GO:0045723	BP	GO:0045723	positive regulation of fatty acid biosynthetic process	1/39	24/18870	0.0484701205584958	0.15606736450627 1	0.11310909375825 9	PTGS2	1	0.4166666666666 67
GO:2000209	BP	GO:2000209	regulation of anoikis	1/39	24/18870	0.0484701205584958	0.15606736450627 1	0.11310909375825 9	PDK4	1	0.4166666666666 67

GO:2000353	BP	GO:2000353	positive regulation of endothelial cell apoptotic process	1/39	24/18870	0.0484701205584958	0.15606736450627 1	0.11310909375825 9	AKR1C3	1	0.4166666666666667
GO:0051897	BP	GO:0051897	positive regulation of phosphatidylinositol 3-kinase/protein kinase B signal transduction	2/39	171/18870	0.0486137254359252	0.156067364506271	0.113109093758259	TXN/AKR1C3	2	0.116959064327485
GO:0098803	CC	GO:0098803	respiratory chain complex	14/39	91/19886	8.5224546956327 4e-24	5.195737209926e-22	3.5732087478789 3e-22	COX7A2/UQCRQ/NDUFA4/UQCR11/NDUFB3/NDUFB1/COX7C/NDUFB2/COX7B/	14	1.53846153846154

									NDUFA6/NDUFS4/NDUFA5/COX6C/UQCRB		
GO:0005746	CC	GO:0005746	mitochondrial respirasome	14/39	94/19886	1.3855299226469 3e-23	5.195737209926e-22	3.5732087478789 3e-22	COX7A2/UQCRQ/NDUFA4/UQCR11/NDUFB3/NDUFB1/COX7C/NDUFB2/COX7B/NDUFA6/NDUFS4/NDUFA5/COX6C/UQCRB	14	1.48936170212766
GO:0070469	CC	GO:0070469	respirasome	14/39	102/19886	4.6826693514138 2e-23	1.1706673378534 6e-21	8.0509052006763 9e-22	COX7A2/UQCRQ/NDUFA4/UQCR11/NDU	14	1.37254901960784

										FB3/NDUFB1/COX7C/NDUFB2/COX7B/NDUFA6/NDUFS4/NDUFA5/COX6C/UQCRB		
GO:0098800	CC		GO:0098800	inner mitochondrial membrane protein complex	15/39	158/19886	3.43456613207328e-22	6.43981149763741e-21	4.42878264398923e-21	COX7A2/UQCRQ/NDUFA4/UQCR11/NDUFB3/NDUFB1/COX7C/NDUFB2/COX7B/DNAJC15/NDUFA6/NDUFS4/NDUFA5/COX6C/U	15	0.949367088607595

										QCRB		
GO:0098798	CC		GO:0098798	mitochondrial protein-containing complex	15/39	300/19886	6.1048995008484 7e-18	9.1573492512727e-17	6.2976858008752 6e-17	COX7A2/UQCRQ/NDUFA4/UQCR11/NDUFB3/NDUFB1/COX7C/NDUFB2/COX7B/DNAJC15/NDUFA6/NDUFS4/NDUFA5/COX6C/UQCRB	15	0.5
GO:0005743	CC		GO:0005743	mitochondrial inner membrane	16/39	497/19886	4.0545936171420 1e-16	5.0682420214275 1e-15	3.4855278463150 6e-15	COX7A2/UQCRQ/NDUFA4/UQCR11/NDUFB3/NDUFB1/COX7C/NDUFB2/	16	0.321931589537223

GO:1990204	CC	GO:1990204	oxidoreductase complex	11/39	128/19886	7.2952494846980 5e-16	7.8163387336050 5e-15	5.3754469887248 8e-15	COX7B/PLA2G4A/DNAJC15/NDUFA6/NDUFS4/NDUFA5/COX6C/UQCRB UQCRQ/NDUFA4/UQCR11/NDUFB3/NDUFB1/NDUFB2/RRM2B/NDUFA6/NDUFS4/NDUFA5/UQCRB	11	0.859375
GO:0070069	CC	GO:0070069	cytochrome complex	8/39	42/19886	1.1436567982892 9e-14	1.0721782483962 1e-13	7.3735767258125 3e-14	COX7A2/UQCRQ/NDUFA4/UQCR11/COX7	8	1.9047619047619

									C/COX7B/COX6C/UQCRB		
GO:0005747	CC	GO:0005747	mitochondrial respiratory chain complex I	7/39	49/19886	5.10874572684521e-12	3.483235722849e-11	2.3954884269417e-11	NDUFA4/NDUFB3/NDUFB1/NDUFB2/NDUFA6/NDUFS4/NDUFA5	7	1.42857142857143
GO:0030964	CC	GO:0030964	NADH dehydrogenase complex	7/39	49/19886	5.10874572684521e-12	3.483235722849e-11	2.3954884269417e-11	NDUFA4/NDUFB3/NDUFB1/NDUFB2/NDUFA6/NDUFS4/NDUFA5	7	1.42857142857143
GO:0045271	CC	GO:0045271	respiratory chain complex I	7/39	49/19886	5.10874572684521e-12	3.483235722849e-11	2.3954884269417e-11	NDUFA4/NDUFB3/NDUFB1/NDUFB2/NDUFA6/NDUFS4/	7	1.42857142857143

									NDUFA5		
GO:0005751	CC	GO:0005751	mitochondrial respiratory chain complex IV	5/39	25/19886	1.1477670885742 9e-09	7.1735443035893 4e-09	4.9333848543982 8e-09	COX7A2/NDUFA4/COX7C/COX7B/COX6C	5	2
GO:0045277	CC	GO:0045277	respiratory chain complex IV	5/39	28/19886	2.1140774381420 8e-09	1.2196600604665 8e-08	8.3878376088228 3e-09	COX7A2/NDUFA4/COX7C/COX7B/COX6C	5	1.78571428571429
GO:1902495	CC	GO:1902495	transmembrane transporter complex	10/39	394/19886	3.1689502877832 9e-09	1.6976519398839 e-08	1.1675080007622 6e-08	UQCRQ/NDUFA4/UQCR11/NDUFB3/NDUFB1/NDUFB2/NDUFA6/NDUFS4/NDUFA5/UQCRB	10	0.2538071065989 85
GO:1990351	CC	GO:1990	transporter complex	10/39	421/19886	5.9734642402501	2.9867321201250	2.0540333177000	UQCRQ/NDUFA4	10	0.23752969121140

		351				7e-09	9e-08	6e-08	/UQCR11/NDUFB3/NDUFB1/NDUFB2/NDUFA6/NDUFS4/NDUFA5/UQCRB		1
GO:0005750	CC	GO:0005750	mitochondrial respiratory chain complex III	3/39	14/19886	2.5008321089811 9e-06	1.1033082833740 5e-05	7.5876639839057 7e-06	UQCRQ/UQCR11/UQCRB	3	2.14285714285714
GO:0045275	CC	GO:0045275	respiratory chain complex III	3/39	14/19886	2.5008321089811 9e-06	1.1033082833740 5e-05	7.5876639839057 7e-06	UQCRQ/UQCR11/UQCRB	3	2.14285714285714
GO:0005778	CC	GO:0005778	peroxisomal membrane	3/39	65/19886	0.000280042433384477	0.00110543065809662	0.000760225996445396	ACSL4/PNPLA8/FAR1	3	0.461538461538462
GO:0031903	CC	GO:0031903	microbody membrane	3/39	65/19886	0.000280042433384477	0.00110543065809662	0.000760225996445396	ACSL4/PNPLA8/FAR1	3	0.461538461538462
GO:0005777	CC	GO:0005777	peroxisome	3/39	143/19886	0.00275325028178342	0.00983303672065509	0.00676236911315227	ACSL4/PNPLA8/FAR1	3	0.20979020979021

GO:0042579	CC	GO:0042579	microbody	3/39	143/19886	0.00275325028178342	0.00983303672065509	0.00676236911315227	ACSL4/PNPLA8/FAR1	3	0.20979020979021
GO:0044232	CC	GO:0044232	organelle membrane contact site	2/39	50/19886	0.00432620516243079	0.014748426690105	0.0101427776535459	STARD3NL/ACSL4	2	0.4
GO:0005811	CC	GO:0005811	lipid droplet	2/39	104/19886	0.0176995076301625	0.0577157857505298	0.0396922596740486	ACSL4/LPCAT2	2	0.192307692307692
GO:0005744	CC	GO:0005744	TIM23 mitochondrial import inner membrane translocase complex	1/39	16/19886	0.0309330072534816	0.0966656476671299	0.0664788313780964	DNAJC15	1	0.625
GO:0044233	CC	GO:0044233	mitochondria-associated endoplasmic reticulum membrane	1/39	21/19886	0.0404069174390655	0.121220752317197	0.0833658507163879	ACSL4	1	0.476190476190476
GO:0070847	CC	GO:0070847	core mediator complex	1/39	26/19886	0.0497905598611481	0.143626614984081	0.0987747948662452	MED7	1	0.384615384615385
GO:0016655	MF	GO:0016	oxidoreductase activity,	8/38	57/18496	2.2195989184990	3.5513582695985	2.1495063210727	NDUFA4/NDUFB	8	1.40350877192982

		655	acting on NAD(P)H, quinone or similar compound as acceptor			7e-13	1e-11	8e-11	3/NDUFB1/NDUFB2/AKR1C3/NDUFA6/NDUFS4/NDUFA5		
GO:0015453	MF	GO:0015453	oxidoreduction-driven active transmembrane transporter activity	8/38	68/18496	9.7726402658336 9e-13	7.0515762192073 9e-11	4.2680592905728 9e-11	NDUFA4/NDUFB3/NDUFB1/NDUFB2/COX7B/NDUFA6/NDUFS4/NDUFA5	8	1.17647058823529
GO:0009055	MF	GO:0009055	electron transfer activity	9/38	117/18496	1.6582867460136e-12	7.0515762192073 9e-11	4.2680592905728 9e-11	NDUFA4/UQCR11/NDUFB3/NDUFB1/NDUFB2/COX7B/NDUFA6/NDUFS4/NDUFA	9	0.769230769230769

									5		
GO:0008137	MF	GO:0008137	NADH dehydrogenase (ubiquinone) activity	7/38	41/18496	1.8390482795463 2e-12	7.0515762192073 9e-11	4.2680592905728 9e-11	NDUFA4/NDUFB3/NDUFB1/NDUFB2/NDUFA6/NDUFS4/NDUFA5	7	1.70731707317073
GO:0050136	MF	GO:0050136	NADH dehydrogenase (quinone) activity	7/38	42/18496	2.2036175685023 1e-12	7.0515762192073 9e-11	4.2680592905728 9e-11	NDUFA4/NDUFB3/NDUFB1/NDUFB2/NDUFA6/NDUFS4/NDUFA5	7	1.66666666666667
GO:0003954	MF	GO:0003954	NADH dehydrogenase activity	7/38	44/18496	3.1208779540252 1e-12	8.3223412107338 9e-11	5.0372065222863 e-11	NDUFA4/NDUFB3/NDUFB1/NDUFB2/NDUFA6/NDUFS4/NDUFA5	7	1.59090909090909
GO:0003955	MF	GO:0003955	NAD(P)H dehydrogen	7/38	45/18496	3.6903495547521	8.4350846965762	5.1054460005593	NDUFA4/NDUFB	7	1.55555555555556

		955	ase (quinone) activity			2e-12	6e-11	2e-11	3/NDUFB1/NDUFB2/NDUFA6/NDUFS4/NDUFA5		
GO:0016651	MF	GO:0016651	oxidoreductase activity, acting on NAD(P)H	8/38	87/18496	7.5158163320015 3e-12	1.5031632664003 1e-10	9.0980934545281 7e-11	NDUFA4/NDUFB3/NDUFB1/NDUFB2/AKR1C3/NDUFA6/NDUFS4/NDUFA5	8	0.919540229885057
GO:0015399	MF	GO:0015399	primary active transmembrane transporter activity	9/38	164/18496	3.5528429894617 7e-11	6.3161653145987 e-10	3.8229421640992 1e-10	NDUFA4/NDUFB3/NDUFB1/NDUFB2/COX7B/NDUFA6/NDUFS4/NDUFA5/ABCA1	9	0.548780487804878
GO:0022	MF	GO:	active	9/38	454/1849	2.588670	4.141873	2.506923	NDUFA4	9	0.198237

804		0022804	transmembrane transporter activity		6	69204117e-07	10726587e-06	19650303e-06	/NDUFB3/NDUFB1/NDUFB2/COX7B/NDUFA6/NDUFS4/NDUFA5/ABCA1		885462555
GO:0102545	MF	GO:0102545	phosphatidyl phospholipase B activity	2/38	11/18496	0.000223430633475419	0.00324990012327882	0.00196704481145823	PLA2G4A/PNPLA8	2	1.81818181818182
GO:0004622	MF	GO:0004622	lysophospholipase activity	2/38	24/18496	0.00110248103866456	0.0146997471821941	0.00889721539974906	PLA2G4A/PNPLA8	2	0.833333333333333
GO:0015036	MF	GO:0015036	disulfide oxidoreductase activity	2/38	34/18496	0.0022120945924 0189	0.0267786086617538	0.0162081052426405	GLRX/TXN	2	0.588235294117647
GO:0004623	MF	GO:0004623	phospholipase A2 activity	2/38	35/18496	0.00234312825790346	0.0267786086617538	0.0162081052426405	PLA2G4A/PNPLA8	2	0.571428571428571
GO:0016620	MF	GO:0016620	oxidoreductase activity, acting on the	2/38	39/18496	0.00290302687918432	0.0309656200446328	0.018742348974383	AKR1C3/FAR1	2	0.512820512820513

			aldehyde or oxo group of donors, NAD or NADP as acceptor								
GO:0016903	MF	GO:0016903	oxidoreductase activity, acting on the aldehyde or oxo group of donors	2/38	48/18496	0.00436809429086157	0.0436809429086157	0.0264384654446884	AKR1C3/FAR1	2	0.4166666666666667
GO:0015485	MF	GO:0015485	cholesterol binding	2/38	52/18496	0.00510838760607174	0.0469084758694912	0.0283919722367973	STARD3NL/ABCA1	2	0.3846153846153846
GO:0016667	MF	GO:0016667	oxidoreductase activity, acting on a sulfur group of donors	2/38	54/18496	0.00549868806392268	0.0469084758694912	0.0283919722367973	GLRX/TXN	2	0.37037037037037
GO:0008374	MF	GO:0008374	O-acyltransferase activity	2/38	55/18496	0.0056988283324 01	0.0469084758694912	0.0283919722367973	PLA2G4A/LPCAT2	2	0.3636363636363636 4
GO:0016705	MF	GO:0016	oxidoreductase activity,	3/38	179/18496	0.0058635594836	0.0469084758694	0.0283919722367	AKR1C3/SC5D/P	3	0.16759776536312

		705	acting on paired donors, with incorporation or reduction of molecular oxygen			8641	912	973	TGS2		8
GO:0032934	MF	GO:0032934	sterol binding	2/38	62/18496	0.0071916797149 9837	0.0547937502095 114	0.0331646382847 042	STARD3NL/ABCA1	2	0.32258064516129
GO:0016209	MF	GO:0016209	antioxidant activity	2/38	83/18496	0.0125968159821 818	0.0916132071431 4	0.0554500990603 216	TXN/PTGS2	2	0.240963855421687
GO:0043178	MF	GO:0043178	alcohol binding	2/38	89/18496	0.0143844431196 277	0.1000656912669 75	0.0605660762931 693	STARD3NL/ABCA1	2	0.224719101123595
GO:0005496	MF	GO:0005496	steroid binding	2/38	102/18496	0.0186078345928 603	0.1083753991978 91	0.0655956363566 18	STARD3NL/ABCA1	2	0.196078431372549
GO:0004620	MF	GO:0004620	phospholipase activity	2/38	104/18496	0.0192988380494 785	0.1083753991978 91	0.0655956363566 18	PLA2G4A/PNPLA8	2	0.192307692307692
GO:0031957	MF	GO:0031	very long-chain	1/38	10/18496	0.0203609847710	0.1083753991978	0.0655956363566	ACSL4	1	1

		957	fatty acid-CoA ligase activity			394	91	18			
GO:0032052	MF	GO:0032052	bile acid binding	1/38	10/18496	0.0203609847710394	0.108375399197891	0.065595636356618	AKR1C3	1	1
GO:0016717	MF	GO:0016717	oxidoreductase activity, acting on paired donors, with oxidation of a pair of donors resulting in the reduction of molecular oxygen to two molecules of water	1/38	11/18496	0.0223747401847958	0.108375399197891	0.065595636356618	SC5D	1	0.9090909090909099
GO:0016725	MF	GO:0016	oxidoreductase activity,	1/38	11/18496	0.0223747401847	0.1083753991978	0.0655956363566	RRM2B	1	0.90909090909090

		725	acting on CH or CH2 groups			958	91	18			9
GO:0016813	MF	GO:0016813	hydrolase activity, acting on carbon-nitrogen (but not peptide) bonds, in linear amidines	1/38	11/18496	0.0223747401847958	0.108375399197891	0.065595636356618	ARG1	1	0.9090909090909099
GO:0030283	MF	GO:0030283	testosterone dehydrogenase [NAD(P)] activity	1/38	11/18496	0.0223747401847958	0.108375399197891	0.065595636356618	AKR1C3	1	0.9090909090909099
GO:0047676	MF	GO:0047676	arachidonate-CoA ligase activity	1/38	11/18496	0.0223747401847958	0.108375399197891	0.065595636356618	ACSL4	1	0.9090909090909099
GO:0003906	MF	GO:0003906	DNA-(apurinic or apyrimidinic site) endonuclease activity	1/38	12/18496	0.0243844648195254	0.108375399197891	0.065595636356618	POLB	1	0.8333333333333333

GO:0004032	MF	GO:0004032	alditol:NADP+ 1-oxidoreductase activity	1/38	12/18496	0.0243844648195254	0.108375399197891	0.065595636356618	AKR1C3	1	0.8333333333333333
GO:0016668	MF	GO:0016668	oxidoreductase activity, acting on a sulfur group of donors, NAD(P) as acceptor	1/38	12/18496	0.0243844648195254	0.108375399197891	0.065595636356618	TXN	1	0.8333333333333333
GO:0042301	MF	GO:0042301	phosphate ion binding	1/38	12/18496	0.0243844648195254	0.108375399197891	0.065595636356618	MTHFD2	1	0.8333333333333333
GO:0008035	MF	GO:0008035	high-density lipoprotein particle binding	1/38	13/18496	0.0263901665256958	0.110654345215605	0.0669749984199713	ABCA1	1	0.769230769230769
GO:0052650	MF	GO:0052650	NADP-retinol dehydrogenase activity	1/38	13/18496	0.0263901665256958	0.110654345215605	0.0669749984199713	AKR1C3	1	0.769230769230769
GO:0016298	MF	GO:0016	lipase activity	2/38	128/18496	0.0284009429540	0.1106543452156	0.0669749984199	PLA2G4A/PNPL	2	0.15625

		298				036	05	713	A8		
GO:0004467	MF	GO:0004467	long-chain fatty acid-CoA ligase activity	1/38	15/18496	0.030389532479929	0.11065434521560 5	0.0669749984199713	ACSL4	1	0.6666666666666667
GO:0016810	MF	GO:0016810	hydrolase activity, acting on carbon-nitrogen (but not peptide) bonds	2/38	136/18496	0.0317507492726564	0.11065434521560 5	0.0669749984199713	MTHFD2/ARG1	2	0.1470588235294112
GO:0004129	MF	GO:0004129	cytochrome-c oxidase activity	1/38	16/18496	0.0323832123547062	0.11065434521560 5	0.0669749984199713	COX7B	1	0.625
GO:0016675	MF	GO:0016675	oxidoreductase activity, acting on a heme group of donors	1/38	16/18496	0.0323832123547062	0.11065434521560 5	0.0669749984199713	COX7B	1	0.625
GO:0140328	MF	GO:0140328	floppase activity	1/38	16/18496	0.0323832123547062	0.11065434521560 5	0.0669749984199713	ABCA1	1	0.625
GO:0000062	MF	GO:0000062	fatty-acyl-CoA binding	1/38	17/18496	0.0343729005544097	0.11065434521560 5	0.0669749984199713	DBI	1	0.5882352941176 47

GO:0008525	MF	GO:0008525	phosphatidylcholine transporter activity	1/38	17/18496	0.0343729005544097	0.11065434521560 5	0.066974998419 9713	ABCA1	1	0.5882352941176 47
GO:0034185	MF	GO:0034185	apolipoprotein binding	1/38	17/18496	0.0343729005544097	0.11065434521560 5	0.066974998419 9713	ABCA1	1	0.5882352941176 47
GO:0046624	MF	GO:0046624	sphingolipid transporter activity	1/38	17/18496	0.0343729005544097	0.11065434521560 5	0.066974998419 9713	ABCA1	1	0.5882352941176 47
GO:0047498	MF	GO:0047498	calcium-dependent phospholipase A2 activity	1/38	17/18496	0.0343729005544097	0.11065434521560 5	0.066974998419 9713	PLA2G4A	1	0.5882352941176 47
GO:0008970	MF	GO:0008970	phospholipase A1 activity	1/38	18/18496	0.0363586048554507	0.11065434521560 5	0.066974998419 9713	PNPLA8	1	0.5555555555555 56
GO:0016646	MF	GO:0016646	oxidoreductase activity, acting on the CH-NH group of donors, NAD or NADP as	1/38	18/18496	0.0363586048554507	0.11065434521560 5	0.066974998419 9713	MTHFD2	1	0.5555555555555 56

			acceptor								
GO:1901567	MF	GO:1901567	fatty acid derivative binding	1/38	18/18496	0.0363586048554507	0.110654345215605	0.0669749984199713	DBI	1	0.5555555555555556
GO:0004303	MF	GO:0004303	estradiol 17-beta-dehydrogenase [NAD(P)] activity	1/38	19/18496	0.0383403330195103	0.110654345215605	0.0669749984199713	AKR1C3	1	0.5263157894736844
GO:0097001	MF	GO:0097001	ceramide binding	1/38	19/18496	0.0383403330195103	0.110654345215605	0.0669749984199713	PLA2G4A	1	0.5263157894736844
GO:0052689	MF	GO:0052689	carboxylic ester hydrolase activity	2/38	153/18496	0.0393551725519589	0.110654345215605	0.0669749984199713	PLA2G4A/PNPLA8	2	0.1307189542483666
GO:0004745	MF	GO:0004745	NAD-retinol dehydrogenase activity	1/38	20/18496	0.0403180927935674	0.110654345215605	0.0669749984199713	AKR1C3	1	0.5
GO:0010314	MF	GO:0010314	phosphatidylinositol-5-phosphate binding	1/38	20/18496	0.0403180927935674	0.110654345215605	0.0669749984199713	PLA2G4A	1	0.5
GO:0120227	MF	GO:0120227	acyl-CoA binding	1/38	20/18496	0.0403180927935674	0.110654345215605	0.0669749984199713	DBI	1	0.5

GO:0003841	MF	GO:0003841	1-acylglycerol-3-phosphate O-acyltransferase activity	1/38	21/18496	0.0422918919099265	0.11065434521560 5	0.0669749984199713	LPCAT2	1	0.4761904761904766
GO:0015645	MF	GO:0015645	fatty acid ligase activity	1/38	22/18496	0.0442617380862419	0.11065434521560 5	0.0669749984199713	ACSL4	1	0.4545454545454545
GO:0016780	MF	GO:0016780	phosphotransferase activity, for other substituted phosphate groups	1/38	22/18496	0.0442617380862419	0.11065434521560 5	0.0669749984199713	SGMS2	1	0.4545454545454545
GO:0042171	MF	GO:0042171	lysophosphatidic acid acyltransferase activity	1/38	22/18496	0.0442617380862419	0.11065434521560 5	0.0669749984199713	LPCAT2	1	0.4545454545454545
GO:0071617	MF	GO:0071617	lysophospholipid acyltransferase activity	1/38	22/18496	0.0442617380862419	0.11065434521560 5	0.0669749984199713	LPCAT2	1	0.4545454545454545
GO:0120020	MF	GO:0120020	cholesterol transfer activity	1/38	22/18496	0.0442617380862419	0.11065434521560 5	0.0669749984199713	ABCA1	1	0.4545454545454545

GO:0008106	MF	GO:0008106	alcohol dehydrogenase (NADP+) activity	1/38	23/18496	0.0462276390255465	0.11206700369829 5	0.0678300285542309	AKR1C3	1	0.434782608695652
GO:0120015	MF	GO:0120015	sterol transfer activity	1/38	23/18496	0.0462276390255465	0.11206700369829 5	0.0678300285542309	ABCA1	1	0.434782608695652
GO:0003887	MF	GO:0003887	DNA-directed DNA polymerase activity	1/38	24/18496	0.0481896024162807	0.11338729980301 3	0.0686291551439291	POLB	1	0.4166666666666667
GO:0016702	MF	GO:0016702	oxidoreductase activity, acting on single donors with incorporation of molecular oxygen, incorporation of two atoms of oxygen	1/38	24/18496	0.0481896024162807	0.11338729980301 3	0.0686291551439291	PTGS2	1	0.4166666666666667

S3 Table Complete Results of KEGG Pathway Enrichment Analysis for Candidate Genes.

	ID	Description	GeneRatio	BgRatio	pvalue	p.adjust	qvalue	geneID	Count
hsa00190	hsa00190	Oxidative phosphorylation	14/32	138/8865	9.32287603365636e-18	7.9244446286079e-16	6.28067438056849e-16	COX7A2/UQCRQ/NDUFA4/UQCR11/NDUFB3/NDUFB1/COX7C/NDUFB2/COX7B/NDUFA6/NDUFS4/NDUFA5/COX6C/UQCRB	14
hsa04932	hsa04932	Non-alcoholic fatty liver disease	14/32	157/8865	5.95517453842208e-17	1.9640333644624e-15	1.55663325480612e-15	COX7A2/UQCRQ/NDUFA4/UQCR11/NDUFB3/NDUFB1/COX7C/NDUFB2/COX7B/NDUFA6/NDUFS4/NDUFA5/COX6C/UQCRB	14
hsa05415	hsa05415	Diabetic cardiomyopathy	15/32	205/8865	6.93188246280848e-17	1.9640333644624e-15	1.55663325480612e-15	COX7A2/UQCRQ/NDUFA4/UQCR11/NDUFB3/NDUFB1/COX7C/NDUFB2/COX7B/NDUFA6/NDUFS4/NDUFA5/COX6C/PDK4/UQCRB	15
hsa05208	hsa05208	Chemical carcinogenesis - reactive oxygen species	15/32	227/8865	3.23547249517269e-16	6.87537905224197e-15	5.44921683397506e-15	COX7A2/UQCRQ/NDUFA4/UQCR11/NDUFB3/NDUFB1/COX7C/NDUFB2/AKR1C3/COX7B/NDUFA6/NDUFS4/NDUFA5/COX6C/UQCRB	15
hsa04714	hsa04714	Thermogenesis	15/32	235/8865	5.4488875203389e-16	9.26310878457613e-15	7.34165897477242e-15	COX7A2/UQCRQ/NDUFA4/UQCR11/NDUFB3/NDUFB1/COX7C/NDUFB2/COX7B/ACSL4/NDUFA6/NDUFS4/NDUFA5/COX6C/UQCRB	15
hsa05012	hsa05012	Parkinson disease	15/32	271/8865	4.60025323970452e-15	6.51702542291474e-14	5.16519662001911e-14	COX7A2/UQCRQ/NDUFA4/UQCR11/NDUFB3/TXN/NDUFB1/COX7C/NDUFB2/COX7B/NDUFA6/NDUFS4/NDUFA5/COX6C/UQCRB	15
hsa05020	hsa05020	Prion disease	14/32	278/8865	1.82736251793887e-13	2.21894020035434e-12	1.75866467891861e-12	COX7A2/UQCRQ/NDUFA4/UQCR11/NDUFB3/NDUFB1/COX7C/NDUFB2/COX7B/NDUFA6/NDUFS4/NDUFA5/COX6C/UQCRB	14

hsa05016	hsa05016	Huntington disease	14/32	311/8865	8.53920622936114e-13	9.07290661869621e-12	7.1909105089357e-12	COX7A2/UQCRQ/NDUFA4/UQCR11/NDUFB3/NDUFB1/COX7C/NDUFB2/COX7B/NDUFA6/NDUFS4/NDUFA5/COX6C/UQCRB	14
hsa05010	hsa05010	Alzheimer disease	15/32	391/8865	1.01668870991127e-12	9.60206003805092e-12	7.61030145430661e-12	COX7A2/UQCRQ/NDUFA4/UQCR11/NDUFB3/NDUFB1/COX7C/NDUFB2/COX7B/NDUFA6/NDUFS4/NDUFA5/COX6C/PTGS2/UQCRB	15
hsa05014	hsa05014	Amyotrophic lateral sclerosis	14/32	371/8865	9.41882643819738e-12	8.00600247246777e-11	6.34531465310139e-11	COX7A2/UQCRQ/NDUFA4/UQCR11/NDUFB3/NDUFB1/COX7C/NDUFB2/COX7B/NDUFA6/NDUFS4/NDUFA5/COX6C/UQCRB	14
hsa05022	hsa05022	Pathways of neurodegeneration - multiple diseases	15/32	483/8865	2.1444777534351e-11	1.65709644583621e-10	1.31336436574015e-10	COX7A2/UQCRQ/NDUFA4/UQCR11/NDUFB3/NDUFB1/COX7C/NDUFB2/COX7B/NDUFA6/NDUFS4/NDUFA5/COX6C/PTGS2/UQCRB	15
hsa04260	hsa04260	Cardiac muscle contraction	7/32	87/8865	1.8938890483966e-08	1.34150474261426e-07	1.06323595699458e-07	COX7A2/UQCRQ/UQCR11/COX7C/COX7B/COX6C/UQCRB	7
hsa04723	hsa04723	Retrograde endocannabinoid signaling	8/32	149/8865	3.94414883950525e-08	2.57886654890728e-07	2.04393138241568e-07	NDUFA4/NDUFB3/NDUFB1/NDUFB2/NDUFA6/NDUFS4/NDUFA5/PTGS2	8
hsa04913	hsa04913	Ovarian steroidogenesis	3/32	52/8865	0.000837411433354606	0.005084283702510 11	0.00402964900260863	AKR1C3/PLA2G4A/PTGS2	3
hsa00590	hsa00590	Arachidonic acid metabolism	3/32	63/8865	0.00146476381781678	0.00830032830096173	0.00657858837475604	AKR1C3/PLA2G4A/PTGS2	3
hsa005	hsa005	Glycerophospholipid metabolism	3/32	103/88	0.00591626335177	0.03143014905629	0.02491058253378	PLA2G4A/LPCAT2/SGMS2	3

64	64			65	322	52	2		
hsa005 65	hsa005 65	Ether lipid metabolism	2/32	50/886 5	0.013882 53364275 39	0.069412 66821376 95	0.055014 37480719 81	PLA2G4A/LPCAT2	2
hsa041 48	hsa041 48	Efferocytosis	3/32	157 /88 65	0.018562 00299429 31	0.087653 90302860 61	0.069471 82407220 79	ABCA1/ARG1/PTGS2	3
hsa043 70	hsa043 70	VEGF signaling pathway	2/32	60/886 5	0.019615 52274561 99	0.087753 65438829 94	0.069550 88397338 89	PLA2G4A/PTGS2	2
hsa033 20	hsa033 20	PPAR signaling pathway	2/32	76/886 5	0.030477 90150952 87	0.129531 08141549 7	0.102662 40508472 8	ACSL4/DBI	2
hsa041 46	hsa041 46	Peroxisome	2/32	83/886 5	0.035829 74908878 49	0.145025 17488317 7	0.114942 55346778 1	ACSL4/FAR1	2

S4 Table Results of Gene Set Enrichment Analysis (GSEA) for Biomarkers.

ID	Description	setSize	enrichmentScore	NES	pvalue	p.adjust	qvalue	rank	leading_edge	core_enrichment
KEGG_RIBOSOME	KEGG_RIBOSOME	69	0.741848846313512	2.5957838585946	1e-10	6.13333333333333e-09	4.07017543859649e-09	1979	tags=62%, list=12%, signal=55%	RPL26L1/RPL22L1/RPS27L/RPL41/RPL39/RPL31/RPS7/RPS17/RPS15A/RPL9/MRPL13/RPL34/RPS27/RPL7/RPS29/RPL27/RPS24/RPL35/RSL24D1/RPL30/FAU/RPS10/RPS18/RPS21/RPL11/RPL6/RPL24/RPLP0/RPL35A/RPL15/RPS9/RPL36AL/RPS27A/RPS23/RPS25/RPL37/RPL22/RPS6/RPL32/RPL3/RPS5/RPL36A/RPS3
KEGG_PARKINSONS_DISEASE	KEGG_PARKINSONS_DISEASE	93	0.663470978512262	2.4204872378980 2	1e-10	6.13333333333333e-09	4.07017543859649e-09	2654	tags=51%, list=16%, signal=42%	UQCRQ/COX7A2/NDUFA4/COX7B/COX7C/NDUFB2/NDUFB1/NDUFA6/NDUFS4/COX6C/COX6A1/UQCR11/NDUFB3/NDUFA5/NDUFS3/CASP3/COX7A2L/NDUFA1/UQCRC2/UQCRB/NDUFV2/NDUFA2/COX5B/NDUFAB1/UQCR10/NDUFB7/COX4I1/NDUFB5/CYCS/NDUFA8/PARK7/NDUFB6/NDUFA7/LRRK2/VDAC3/NDUFA9/COX5A/NDUFB8/NDUFS6/UBE2J1/SDHB/NDUFB10/NDUFB4/SDHD/UQCRFS1/UBE2L3/HTRA2
KEGG_OXIDATI	KEGG_OXIDATIVE_PHOSPHO	95	0.625769838175547	2.28377039584	1e-10	6.13333333333333e-09	4.07017543859649e-09	2621	tags=51%, list=16%, signal=43%	UQCRQ/COX7A2/NDUFA4/COX7B/COX7C/NDUFB2/NDUFB1/NDUFA6/NDUFS4/COX6C/COX6A1/UQCR11/

VE_PHOSPHORYLATION	RYLATION			21						NDUFB3/NDUFA5/ATP6V1G1/NDUFS3/PPA2/COX7A2L/NDUFA1/UQCRC2/UQCRB/NDUFV2/NDUFA2/COX5B/NDUFAB1/UQCR10/NDUFB7/COX4I1/NDUFB5/ATP6V1D/ATP6V1E1/ATP6V0E1/PPA1/NDUFA8/NDUFB6/NDUFA7/COX17/NDUFA9/COX5A/COX11/NDUFB8/NDUFS6/SDHB/ATP6V1C1/NDUFB10/NDUFB4/SDHD/UQCRFS1
KEGG_ALZHEIMERS_DISEASE	KEGG_ALZHEIMERS_DISEASE	138	0.530135181685373	2.0148542374911 7	1.43107462258 18e-08	6.5829432638763e-07	4.3685435847234e-07	2851	tags=38%, list=18%, signal=32%	UQCRQ/COX7A2/NDUFA4/COX7B/COX7C/NDUFB2/NDUFB1/NDUFA6/NDUFS4/COX6C/COX6A1/UQCR11/NDUFB3/NDUFA5/NDUFS3/CASP3/COX7A2L/NDUFA1/UQCRC2/UQCRB/NDUFV2/NDUFA2/COX5B/NDUFAB1/UQCR10/NDUFB7/COX4I1/NDUFB5/NAE1/CYCS/IDE/ADAM10/NDUFA8/PLCB1/NDUFB6/NDUFA7/FAS/PPP3CB/ITPR1/NDUFA9/COX5A/NDUFB8/NDUFS6/ADAM17/SDHB/NDUFB10/NDUFB4/PPP3CC/SDHD/UQCRFS1/ATF6/NDUFC1/CASP7
KEGG_HUNTIN	KEGG_HUNTINGTONS_DISEA	152	0.505745856446466	1.92883544774	3.65372293463253	1.34457003994477e-06	8.92277600878681e-07	2662	tags=37%, list=17%, signal=31%	UQCRQ/COX7A2/NDUFA4/COX7B/COX7C/NDUFB2/NDUFB1/NDUFA6/NDUFS4/COX6C/POLR2K/COX6A1/

GTONS_DISEASE	SE			103	e-08					UQCR11/NDUFB3/NDUFA5/NDUFS3/CASP3/COX7A2L/IFT57/NDUFA1/UQCRC2/UQCRB/NDUFV2/NDUFA2/COX5B/NDUFAB1/UQCR10/NDUFB7/COX4I1/POLR2F/NDUFB5/CYCS/POLR2H/NDUFA8/DCTN4/TBPL1/SOD1/PLCB1/NDUFB6/NDUFA7/REST/CREB1/VDAC3/ITPR1/NDUFA9/COX5A/NDUFB8/NDUFS6/SDHB/NDUFB10/HDAC2/POLR2B/NDUFB4/SDHD/UQCRFS1/TAF4B
KEGG_PATHWAYS_IN_CANCER	KEGG_PATHWAYS_IN_CANCER	318	-0.305987789	-1.653411995	7.475540163 56926e-08	2.29249898349 457e-06	1.52133799820 006e-06	4034	tags=38%, list=25%, signal=29%	FOS/CCNE1/FZD10/PLCG1/FGF9/ARNT/WNT10A/FGF10/FGF22/LAMA1/WNT7B/WNT2B/TRAF2/CTNNA2/FGFR2/CCNA1/PDGFB/LAMC3/MAPK10/ITGA3/CBLC/WNT2/HDAC1/RALGDS/COL4A2/FGF11/JUN/LAMA4/E2F2/VEGFC/GLI2/FH/IKBKB/PIAS1/SUFU/JUP/DVL2/LAMC1/CTBP1/FGFR1/TP53/BMP4/FZD4/STK36/BCL2L1/EGLN3/MYC/APC2/VHL/RET/CSF1R/ZBTB16/XIAP/ERBB2/SPI1/GLI3/MAP2K2/STAT5B/EPAS1/EGLN2/TPR/SMO/PIK3R5/NTRK1/MET/TRAF3/NFKBIA/TFG/CSF3R/MAP2K1/AXIN1/ITGA2/IKBKG/MITF/EGF/MA

										PK3/AKT1/CBL/HSP90AB1/PLCG2/RALBP1/TCF7/PGF/BCR/CDKN1A/KLK3/ITGA2B/PIK3R2/RAC1/CREBBP/RXRA/PAX8/RXRB/JAK1/PDGFA/RARA/EP300/PPARD/AKT2/CASP9/PRKCB/ARAF/TRAF6/CTNNA1/MAX/TPM3/STAT5A/DVL3/RHOA/RELA/ABL1/PIAS4/GRB2/NFKB1/STK4/TGFB1/STAT3/PIK3CD/RASSF5/RAC2/CRKL
KEGG_FOCAL_ADHESION	KEGG_FOCAL_ADHESION	194	-0.356249742	-1.835051217	1.56442278020767e-07	4.11219702226015e-06	2.72891793239232e-06	3603	tags=40%, list=22%, signal=31%	COL5A3/PDGFB/PAK3/LAMC3/COL6A2/MAPK10/COL11A1/COL6A6/ITGA3/COL6A3/CAV2/COL4A2/JUN/LAMA4/PARVG/VEGFC/FLNC/TLN2/COL1A1/LAMC1/PARVB/XIAP/MYL10/ERBB2/ITGB4/ELK1/FLNB/FYN/PIK3R5/ACTN2/MET/MAP2K1/COL11A2/RAPGEF1/ITGA2/PAK4/EGF/MAPK3/SHC3/AKT1/VASP/MYLK/MYL9/VAV1/PGF/FLNA/ITGA5/ITGA2B/ITGB3/PIK3R2/CCND3/SRC/RAC1/ACTB/PDGFA/VWF/FLT1/PXN/AKT2/PRKCB/ITGB5/PIP5K1C/ILK/ZYX/SHC1/RHOA/THBS1/PPP1CA/VCL/GRB2/TLN1/DIAPH1/ACTN1/ACTN4/PIK3CD/RAC2/CRKL

KEGG_CHEMOKINE_SIGNALING_PATHWAY	KEGG_CHEMOKINE_SIGNALING_PATHWAY	179	-0.365985996	-1.904373756	1.979134772 54776e-07	4.55200997685 985e-06	3.02078465283 605e-06	1967	tags=29%, list=12%, signal=26%	ADCY1/STAT5B/GNG7/PIK3R5/NFKBIA/MAP2K1/ADCY3/IKBKG/HCK/MAPK3/SHC3/AKT1/RASGRP2/GSK3A/PRKCZ/NFKBIB/GNB3/ADCY6/CXCR1/PF4/GRK6/GRK5/VAV1/CXCR2/PRKCD/LYN/PIK3R2/RAC1/PLCB2/PARD3/DOCK2/GNB2/PXN/AKT2/GNB1/PRKCB/CSK/ARRB1/SHC1/RHOA/PRKACA/PREX1/RELA/GRB2/NFKB1/GNAI2/WAS/ELMO1/STAT3/PIK3CD/RAC2/CRKL
KEGG_AXON_GUIDANCE	KEGG_AXON_GUIDANCE	128	-0.387036529	-1.894412205	2.164751163 04117e-06	4.42571348888 417e-05	2.93697233816 112e-05	2981	tags=38%, list=19%, signal=31%	PLXNB2/SEMA6B/LIMK2/EPHB1/SRGAP1/EPHA2/ARHGEF12/RND1/SEMA4F/NFAT5/PLXNA1/EFNA1/EPHA8/SEMA7A/ABLIM1/LRRC4C/EPHB3/EPHA1/EFNA3/FYN/MET/SEMA5B/UNC5B/EPHB2/PLXNA2/PAK4/SEMA5A/MAPK3/EPHA4/SRGAP3/SEMA4C/EPHB6/RHOD/CHP1/RAC1/ABLIM3/SEMA4A/NCK2/NFATC3/SEMA4B/RHOA/NFATC1/ABL1/GNAI2/CFL1/SEMA3F/SEMA4D/RAC2
KEGG_NEUROTROPH	KEGG_NEUROTROPHIN_SIGNALING_P	122	-0.390997232	-1.898616973	3.682505858 49927e-06	6.77581077963 865e-05	4.49653346932 542e-05	2231	tags=36%, list=14%, signal=31%	SORT1/CAMK2B/IRAK1/RPS6KA5/MAP2K2/PIK3R5/NTRK1/RPS6KA1/NFKBIA/CAMK2G/MAP2K1/NTRK3/RAPGEF1/MAPK12/MAPK7/MAPK3

IN_SIGNALING_PATHWAY	ATHWAY									/SHC3/AKT1/NFKBIB/CALM1/PLCG2/ARHGDIB/MAP3K3/PRKCD/PIK3R2/SH2B3/RAC1/ARHGDIA/MAPKAPK2/AKT2/YWHAH/MAGED1/TRAF6/CSK/SHC1/SH2B2/RHOA/RELA/ABL1/GRB2/NFKB1/YWHAB/PIK3CD/CRKL
KEGG_REGULATION_OF_ACTIN_CYTOSKELETON	KEGG_REGULATION_OF_ACTIN_CYTOSKELETON	208	-0.334394427	-1.737519753	4.13634786391448e-06	6.91898188145694e-05	4.59154404032612e-05	2718	tags=32%, list=17%, signal=27%	PIP4K2A/PFN4/ARHGEF12/FGFR1/PIP4K2B/GNA12/APC2/ITGAX/MYL10/ITGAM/ARHGEF4/ITGB4/MAP2K2/CD14/PIK3R5/ACTN2/SSH2/MAP2K1/INSRR/ITGA2/PAK4/EGF/MAPK3/ARHGEF7/MYLK/MYH14/PIP4K2C/ARPC1B/ARHGEF1/MYL9/SSH3/VAV1/ITGA5/ITGA2B/ITGB3/PFN1/PIK3R2/RAC1/ACTB/PDGFA/CYFIP2/GSN/PXN/ARAF/ITGAL/ITGB5/PIP5K1C/WASF2/FGD3/CSK/RHOA/ITGB2/PPP1CA/VCL/SLC9A1/CFL1/WAS/MSN/DIAPH1/MYH9/ACTN1/SSH1/ACTN4/PIK3CD/RAC2/CRKL
KEGG_TIGHT_JUNCTION	KEGG_TIGHT_JUNCTION	126	-0.37373772	-1.828445123	1.13278123672358e-05	0.000160332113505492	0.000106398885392661	1919	tags=28%, list=12%, signal=25%	CLDN7/YBX3/ACTN2/CLDN17/LLGL1/CSNK2B/AKT1/ASH1L/PRKCZ/MYH14/EPB41/CLDN15/MYL9/PRKCD/JAM3/SYMPK/CSNK2A1/SRC/ACTB/PARD3/TJAP1/HCLS1/AKT2/PR

KEGG_ENDOCYTOSIS	KEGG_ENDOCYTOSIS	170	-0.344961237	-1.773690561	1.117003053539 54e-05	0.000160332113505492	0.000106398885392661	2519	tags=36%, list=16%, signal=31%	KCB/SPTAN1/F11R/CTNNA1/CLDN5/CTTN/RHOA/PPP2R1A/GNAI2/MYH9/ACTN1/ACTN4 PIP4K2B/RAB5C/ERBB3/IQSEC1/HLA-A/PSD/RET/CSF1R/RAB11FIP3/CHMP2A/HLA-B/CHMP4B/EHD4/ACAP1/ITCH/IL2RB/ASAP3/HLA-C/ARAP3/DNM1/NTRK1/MET/HSPA1L/IQSEC3/EGF/SH3GLB2/PRKCZ/CBL/RUFY1/HLA-G/PSD4/CXCR1/GRK6/VPS37B/GRK5/LDLRAP1/AP2M1/CXCR2/AP2A2/SMAP2/SRC/VPS28/DNM2/HLA-F/IL2RG/PARD3/VPS37C/FLT1/ARFGAP2/TRAF6/PIP5K1C/HLA-E/SH3GL1/HGS/ARRB1/RAB11FIP1/GIT2/EHD1/RAB5B/EHD3/SH3KBP1
KEGG_FC_GAMMA_R_MEDIATED_PHAGOCYTOSIS	KEGG_FC_GAMMA_R_MEDIATED_PHAGOCYTOSIS	89	-0.413704561	-1.890842392	1.447582576521 13e-05	0.000190253710057063	0.000126255322463497	1872	tags=38%, list=12%, signal=34%	ASAP3/PIK3R5/DNM1/GAB2/MAP2K1/SYK/HCK/MAPK3/AKT1/VASP/PLA2G6/MARCKSL1/ARPC1B/PLCG2/VAV1/WASF3/PRKCD/SPHK2/LYN/PIK3R2/RAC1/DNM2/DOCK2/GSN/AKT2/PRKCB/PIP5K1C/WASF2/INPP5D/CFL1/WAS/PIK3CD/RAC2/CRKL

KEGG_PROTEASOME	KEGG_PROTEASOME	40	0.62737965029954	2.0437887654238 3	1.787385259603 01e-05	0.000219252591844636	0.000145499431658912	2994	tags=57%, list=19%, signal=47%	PSMA2/PSMA4/PSMA3/PSMC2/PSMC6/PSMB1/POMP/PSMA7/PSMD14/PSMB7/PSMB6/IFNG/PSMB4/PSMD6/PSMD12/PSMD4/PSMB5/PSMC3/PSMB8/PSMA5/PSMC1/PSMA8/PSMA1
KEGG_ADHERENS_JUNCTION	KEGG_ADHERENS_JUNCTION	66	-0.4776055	-2.058263636	1.965897963490 01e-05	0.000221451490171423	0.000146958654804834	2254	tags=44%, list=14%, signal=38%	LMO7/ERBB2/FYN/ACTN2/MET/INSR/CSNK2B/MAPK3/PTPRJ/TCF7/WASF3/SORBS1/CSNK2A1/SRC/RAC1/CREBBP/PTPN1/ACTB/PARD3/EP300/WASF2/CTNNA1/RHOA/VCL/PTPN6/WAS/ACTN1/ACTN4/RAC2
KEGG_NEUROACTIVE_LIGAND_RECEPTOR_INTERACTION	KEGG_NEUROACTIVE_LIGAND_RECEPTOR_INTERACTION	262	-0.300748872	-1.612023058	2.046019202670 75e-05	0.000221451490171423	0.000146958654804834	7333	tags=60%, list=46%, signal=33%	TSHB/GPR50/CSH1/LEPR/APLNR/F2RL1/NPY1R/ADRA1D/GABRG1/GABRB2/GRM8/SSTR2/SCTR/LTB4R/GABRA5/GALR2/TACR2/GLRA2/TAAR8/F2RL3/TAAR9/GRM1/SSTR5/MC1R/NTSR2/OPRD1/AVPR2/HTR1D/FPR3/ADRB3/SSTR3/GPR35/GABBR1/GRIN2A/CHRNB4/HTR6/MC3R/S1PR5/GRIK5/CHRNA9/CHRNA4/GABRR2/FSHB/P2RY6/OPRM1/GH2/GRID2/NMUR2/GRM3/GH1/PRL/GPR156/MLNR/GABRG2/GRIA1/EDNRA/CHRND/P2RX2/DRD1/MAS1/P2RY4/GRM6/F2/HRH4/OPRL1/OPRK1/TAAR5/HTR1F/FSHR/GABRR1/ADRA1A/PL

										G/GALR1/BDKRB2/CHRM1/CRHR1/NMBR/GRIN2D/P2RY2/GABRB1/CHRNA6/NPY5R/UTS2R/GRIK2/GABRA3/CCKAR/CHRNB2/GABRA6/C5AR1/ADORA1/ADRA2B/SSTR4/GRIN2B/GHR/LPAR3/KISS1R/S1PR2/GRM4/LEP/NR3C1/CHRM5/F2RL2/GRIN1/NPBWR2/GABRD/HTR5A/HCRTR2/CHRNA1/GABRA4/CALCR/P2RX1/BDKRB1/GRIN3B/PRLR/TAAR2/AGTR2/HTR1B/GABRB3/NPY2R/GABRQ/PRSS3/CRHR2/P2RY1/GLRB/CGA/NMUR1/CHRNA2/DRD2/LPAR2/GIPR/PTGER1/GRM2/S1PR4/MCHR1/FPR1/GRID1/GRIN2C/GALR3/DRD5/P2RY8/HTR4/CNR2/AVPR1B/HRH2/FPR2/ADRA2A/PTGIR/VIPR2/ADRA2C/GRPR/GLP1R/VIPR1/PARD3/THRA/PTAFR/TBXA2R
KEGG_LEUKOCYTE_TRANSENDOT	KEGG_LEUKOCYTE_TRANSENDOTHELIAL_MIGRATION	112	-0.377403631	-1.829531293	3.25902517587885e-05	0.000333144795756505	0.000221080070410495	2212	tags=32%, list=14%, signal=28%	MYL10/ITGAM/THY1/CLDN7/PIK3R5/ACTN2/CLDN17/ICAM1/MAPK12/VASP/ESAM/CLDN15/MYL9/PLCG2/VAV1/JAM3/PIK3R2/RAC1/ACTB/PXN/PRKCB/ITGAL/F11R/CTNNA1/CLDN5/RHOA/ITGB2/VCL/GNAI2/MSN/SIPA1/ACTN1/ACTN4/PIK3CD

HELIAL_MIGRATION										/RASSF5/RAC2
KEGG_BASAL_TRANSCRIPTION_FACTORS	KEGG_BASAL_TRANSCRIPTION_FACTORS	32	0.648214752814698	2.01750767496829	5.56449928214901e-05	0.000538877825218641	0.000357607710099327	2147	tags=47%, list=13%, signal=41%	GTF2B/TAF11/TAF7/GTF2A1/GTF2E2/GTF2A2/TAF13/GTF2E1/TBPL1/TAF2/TAF5/GTF2H1/GTF2F2/TAF12/TAF10
KEGG_ACUTE_MYELOID_LEUKEMIA	KEGG_ACUTE_MYELOID_LEUKEMIA	56	-0.447016544	-1.864842572	0.000167953103992793	0.00154516855673369	0.00102539789806126	2319	tags=45%, list=14%, signal=38%	IKBKB/JUP/MYC/ZBTB16/SPI1/MAP2K2/STAT5B/PIK3R5/MAP2K1/IKBKG/MAPK3/AKT1/TCF7/PIM1/PIK3R2/RARA/PPARD/AKT2/ARAF/STAT5A/RELA/GRB2/NFKB1/STAT3/PIK3CD
KEGG_B_CELL_RECEPTO	KEGG_B_CELL_RECEPTOR_SIGNALING_PATH	75	-0.409393568	-1.827653811	0.0001925755702341	0.00168732880586069	0.0011197376514865	1962	tags=37%, list=12%, signal=33%	LILRB3/MAP2K2/PIK3R5/NFKBIA/MAP2K1/IKBKG/SYK/MAPK3/AKT1/CARD11/NFKBIB/PLCG2/VAV1/CHP1/LYN/PIK3R2/RAC1/NFATC3/AKT2/PRKCB/NFATC1/INPP5D/RELA/

R_SIGNALING_PATHWAY	WAY									GRB2/NFKB1/PTPN6/PIK3CD/RAC2
KEGG_SPLICEOSOME	KEGG_SPLICEOSOME	115	0.447780658081114	1.66704354958918	0.000207349613589144	0.00173419676820011	0.00115083996059046	3049	tags=47%, list=19%, signal=38%	SNRPG/SF3B6/LSM3/LSM7/PHF5A/SNRPE/SNRPD2/PLRG1/SNRPD1/LSM5/PRPF40A/CDC40/LSM8/SNRPB2/SNRNP27/SF3B5/PRPF18/BUD31/CWC15/LSM6/SNRPF/HNRNPC/RBM25/ISY1/RBM8A/SNRPD3/ZMAT2/SYF2/MAGOHB/TRA2B/NCBP1/SRSF6/BCAS2/SLU7/TXNL4A/CDC5L/SF3B1/SNW1/SRSF7/SRSF3/SRSF1/U2SURP/PPIL1/PRPF38A/PPIE/TCERG1/CRNKL1/NCBP2/DHX15/CCDC12/SNRPA1/LSM2/SMNDC1/PPIH
KEGG_MAPK_SIGNALING_PATHWAY	KEGG_MAPK_SIGNALING_PATHWAY	254	-0.276353502	-1.472741669	0.000245199874347547	0.00196159899478038	0.00130174761667348	4051	tags=38%, list=25%, signal=29%	RPS6KA2/FOS/NFATC2/FGF9/MOS/RASGRF1/FGF10/FGF22/ARRB2/TRAF2/PLA2G12A/ECSIT/FGFR2/CACNA2D1/PDGFB/HSPA2/MAP4K4/RPS6KA6/PPP5C/MAPK10/CHP2/NGF/FGF11/PLA2G2D/JUN/MAPKAPK3/CACNB2/RASGRP4/IKBKB/MAPK11/PLA2G2A/FLNC/CACNA2D2/FGFR1

										/TP53/MAP3K14/GNA12/MYC/CACNG8/HSPB1/CACNA1H/DUSP7/CACNB1/CACNB3/RPS6KA5/MAP2K2/ELK1/CD14/FLNB/NTRK1/HSPA1L/RPS6KA1/DUSP16/CACNA1B/CDC25B/MAP2K1/IKBKG/RELB/EGF/MAPK12/MAPK7/MAPK3/AKT1/RASGRP2/PLA2G6/TNFRSF1A/NR4A1/CHP1/MAP3K3/FLNA/DAXX/JUND/MAP3K12/RAC1/MAP4K2/SRF/PDGFA/MAP3K11/CACNG4/MAPKAPK2/AKT2/PRKCB/TRAF6/TAOK3/MAX/ARRB1/PRKACA/MKNK2/RELA/CACNG6/GRB2/NFKB1/STK4/TGFB1/RAC2/CRKL
KEGG_CHRONIC_MYELOID_LEUKEMIA	KEGG_CHRONIC_MYELOID_LEUKEMIA	72	-0.405571023	-1.775785 53	0.000293048802671094	0.00224670748714506	0.00149095004 86775	2550	tags=40%, list=16%, signal=34%	CTBP1/TP53/BCL2L1/MYC/MAP2K2/STAT5B/PIK3R5/GAB2/NFKBIA/MAP2K1/IKBKG/MAPK3/SHC3/AKT1/CBL/BCR/CDKN1A/PIK3R2/AKT2/ARAF/STAT5A/SHC1/RELA/ABL1/GRB2/NFKB1/TGFB1/PIK3CD/CRKL
KEGG_GAP_JU	KEGG_GAP_JUNCTION	83	-0.373527438	-1.688801 619	0.000339942 69025	0.00250197820 029129	0.00166035166 609719	3184	tags=39%, list=20%, signal=31%	GNAQ/GNA11/TUBB/GUCY1A2/ITPR3/DRD2/TUBB3/ADCY1/MAP2K2/TUBA1A/MAP2K1/TUBA8/ITPR2/A

NCTION					6968				DCY3/EGF/MAPK7/MAPK3/TUBB4B/ADCY6/SRC/PLCB2/PDGFA/TUBB1/TUBA1C/PRKCB/TUBA1B/GNAS/PRKACA/TUBA4A/GRB2/GNAI2/CSNK1D
KEGG_ERBB_SIGNALING_PATHWAY	KEGG_ERBB_SIGNALING_PATHWAY	86	-0.376475329	-1.725166615	0.000392477446714853	0.00277753269982819	0.00184321391979445	2451	tags=35%, list=15%, signal=30%	ERBB3/MYC/CAMK2B/ERBB2/MAP2K2/ELK1/STAT5B/PIK3R5/CAMK2G/MAP2K1/PAK4/EGF/MAPK3/SHC3/AKT1/CBL/PLCG2/CDKN1A/PIK3R2/SRC/NCK2/AKT2/PRKCB/ARAF/STAT5A/SHC1/ABL1/GRB2/PIK3CD/CRKL
KEGG_INSULIN_SIGNALING_PATHWAY	KEGG_INSULIN_SIGNALING_PATHWAY	133	-0.320951866	-1.556665326	0.000468186026994403	0.00319060107285075	0.00211733251974077	1962	tags=29%, list=12%, signal=25%	MAP2K2/ELK1/PIK3R5/PHKG1/GYS1/INSR/MAP2K1/RAPGEF1/MAPK3/SHC3/AKT1/PRKCZ/CBL/TSC1/INPP5K/EXOC7/FLOT2/SREBF1/CALM1/SORBS1/PIK3R2/PTPN1/AKT2/TSC2/ARAF/PYGB/PRKAR2A/ACACB/SHC1/SH2B2/PRKACA/HK1/PPP1CA/MKNK2/INPP5D/GRB2/PIK3CD/CRKL
KEGG_VEGF_SIGNA	KEGG_VEGF_SIGNALING_PATHWAY	71	-0.397570197	-1.745151498	0.000490493906229835	0.00322324566951035	0.00213899598205492	3048	tags=41%, list=19%, signal=33%	PLA2G2D/SPHK1/MAPKAPK3/MAPK11/PLA2G2A/NFAT5/HSPB1/MAP2K2/PIK3R5/MAP2K1/MAPK12/MAPK3/AKT1/PLA2G6/PLCG2/CHP1/SPH

LING_PATHWAY										K2/PIK3R2/SRC/RAC1/NFATC3/PXN/MAPKAPK2/AKT2/CASP9/PRKCB/NFATC1/PIK3CD/RAC2
KEGG_FC_EPSILON_RI_SIGNALING_PATHWAY	KEGG_FC_EPSILON_RI_SIGNALING_PATHWAY	73	-0.393014222	-1.731582283	0.000598813413924572	0.00369595213782716	0.00245269135004548	1962	tags=33%, list=12%, signal=29%	MAP2K2/MS4A2/FYN/PIK3R5/GAB2/IL13/MAP2K1/MAPK12/SYK/MAPK3/AKT1/PLA2G6/PLCG2/VAV1/PRKCD/LYN/PIK3R2/RAC1/AKT2/PRKCB/INPP5D/GRB2/PIK3CD/RAC2
KEGG_CALCIUM_SIGNALING_PATHWAY	KEGG_CALCIUM_SIGNALING_PATHWAY	172	-0.294045062	-1.499320829	0.000602600892037036	0.00369595213782716	0.00245269135004548	5561	tags=44%, list=35%, signal=29%	ATP2B1/EGFR/EDNRA/P2RX2/DRD1/CACNA1I/NOS3/MYLK3/PPP3CA/PDE1C/ADRA1A/BDKRB2/CHRM1/ADCY9/CACNA1F/ATP2A2/PRKCG/GRIN2D/GNA14/ATP2B3/TNNC2/CALML6/CCKAR/PRKCA/PLCG1/CHRM5/GRIN1/SLC8A2/GNAL/ATP2A1/ADCY2/HTR5A/ATP2B4/P2RX1/BDKRB1/CHP2/GNAQ/GNA11/GNA15/SPHK1/PHKA2/ITPR3/PLCD3/PTGER1/VDAC1/ERBB3/PDE1B/CAMK2B/ERBB2/CACNA1H/GRIN2C/ADCY1

										/DRD5/PHKG1/PLCD1/HTR4/CACNA1B/CAMK2G/AVPR1B/ITPR2/HRH2/ADCY3/MYLK/GRPR/CALM1/PLCG2/CHP1/SPHK2/PLCB2/PRKCB/PTAFR/GNAS/TBXA2R/ITPKB/PRKACA/ATP2A3
KEGG_OLFACTORY_TRANSDUCTION	KEGG_OLFACTORY_TRANSDUCTION	108	-0.330943482	-1.60049186	0.000637606266190938	0.00378450170900428	0.00251145422336668	7570	tags=62%, list=47%, signal=33%	OR2C3/OR7A5/GUCA1A/OR51B4/OR5K1/OR51E1/OR2L2/OR2J2/OR10C1/OR2S2/OR2B2/PDC/OR14J1/OR51M1/PRKG2/OR2L13/OR7C2/OR8B8/OR1J2/OR5J2/OR1F1/OR5P3/OR1D2/CALML5/OR12D3/OR1C1/GUCA1C/OR1A2/OR51B2/OR5P2/OR8D2/OR2B6/OR3A1/OR5I1/OR51I1/PDE1C/OR5L2/OR2B3/OR10A4/OR10H1/OR1A1/OR1Q1/GUCY2D/OR10A3/OR2A4/OR12D2/GUCA1B/PRKG1/CALML6/OR7E24/CNGB1/OR1G1/OR10A5/ARRB2/GNAL/OR2C1/OR10H2/OR8G1/OR1I1/OR4D2/OR2H1/OR7D2/CAMK2B/CAMK2G/ADCY3/CALM1/PRKACA
KEGG_PHOSPHATID	KEGG_PHOSPHATIDYLINOSITOL_SIG	74	-0.386321985	-1.718159345	0.000873431080827512	0.00502222871475819	0.00333282912421024	2927	tags=43%, list=18%, signal=36%	OCRL/ITPR3/SYNJ2/PIP4K2A/INPP5A/PLCD3/PIP4K2B/IMPA2/CDS1/PIK3R5/PLCD1/ITPR2/INPP5B/DGKA/INPP5K/PIP4K2C/INPPL1/CALM1/PLC

YLINOSITOL_SIGNALING_SYSTEM	NALING_SYSTEM									G2/ITPK1/PIK3R2/PLCB2/DGKD/PRKCB/PIP5K1C/PI4KA/ITPKB/PI4KB/INPP5D/CDIPT/CDS2/PIK3CD
KEGG_NON_SMALL_CELL_LUNG_CANCER	KEGG_NON_SMALL_CELL_LUNG_CANCER	54	-0.436369659	-1.801448 71	0.001158284 27424 506	0.00614880630491525	0.00408044354 33076	2169	tags=35%, list=13%, signal=31%	ERBB2/MAP2K2/PIK3R5/MAP2K1/EGF/MAPK3/AKT1/PLCG2/PIK3R2/RXRA/RXRB/AKT2/CASP9/PRKCB/ARAF/GRB2/STK4/PIK3CD/RASSF5
KEGG_PYRIMIDINE_METABOLISM	KEGG_PYRIMIDINE_METABOLISM	91	0.460203799260694	1.6778051 5763 865	0.001193933 06836 104	0.00614880630491525	0.00408044354 33076	4159	tags=49%, list=26%, signal=37%	POLE4/POLR2K/NME1/POLR2F/POLR3GL/NUDT2/POLR3F/POLR2H/RRM2B/DUT/NME7/NME3/UMPS/POLR3K/CMPK1/PRIM1/AK3/UPRT/POLE2/POLR2B/PNPT1/POLR3G/POLR3D/DCK/ENTPD4/POLR2G/POLE3/NT5C3A/ENTPD1/DTYMK/POLR2L/POLR2D/PRIM2/PNP/UPP1/POLR2I/DPYD/CTPS1/POLR1E/ITPA/POLR1C/CMPK2/TYMS/RRM2/POLD4
KEG	KEGG_AR	72	-0.381573895	-1.67	0.0011	0.00614	0.00408	3803	tags=40%,	ACTN3/CTNNA2/CACNA2D1/CDH2/

G_ARRHYTHMOGENIC_RIGHT_VENTRICULAR_CARDIOMYOPATHY_ARVC	RHYTHMOGENIC_RIGHT_VENTRICULAR_CARDIOMYOPATHY_ARVC			0714532	4177279788185	880630491525	04435433076		list=24%, signal=31%	ITGA3/DES/CACNB2/JUP/CACNA2D2/CACNG8/ITGB4/CACNB1/CACNB3/ACTN2/EMD/ITGA2/DAG1/TCF7/ITGA5/ITGA2B/ITGB3/ACTB/CACNG4/ITGB5/CTNNA1/LMNA/CACNG6/ACTN1/ACTN4
KEGG_DILATED_CARDIOMYOPATHY	KEGG_DILATED_CARDIOMYOPATHY	88	-0.362706786	-1.649367062	0.0012030273205269	0.00614880630491525	0.0040804435433076	2034	tags=34%, list=13%, signal=30%	CACNA2D1/ADCY2/TPM1/ITGA3/DES/CACNB2/CACNA2D2/CACNG8/ITGB4/CACNB1/CACNB3/ADCY1/EMD/ITGA2/ADCY3/DAG1/ADCY6/TPM4/ITGA5/ITGA2B/ITGB3/ACTB/CACNG4/ITGB5/TPM3/LMNA/GNAS/PRKACA/CACNG6/TGFB1
KEGG_VIRAL_	KEGG_VIRAL_MYOCARDIT	64	-0.405851521	-1.747268151	0.00133356046866	0.00663176016849252	0.00440093924224904	2488	tags=34%, list=15%, signal=29%	CD40/HLA-A/PRF1/HLA-B/HLA-C/FYN/EIF4G3/ICAM1/DAG1/MYH14/HLA-G/EIF4G1/RAC1/ACTB/HLA-F/C

MYOCARDITIS	IS				426					ASP9/ITGAL/HLA-E/ITGB2/ABL1/MYH9/RAC2
KEGG_PROTEIN_EXPORT	KEGG_PROTEIN_EXPORT	22	0.638433842036342	1.818363468871 6	0.001629106072361 41	0.00788830308722365	0.00523480067573195	3086	tags=59%, list=19%, signal=48%	SRP14/IMMP1L/SEC11A/SRP19/SEC11C/SRP9/SRP72/SPCS3/SEC61B/SEC61G/SRP54/SPCS2/SRPRB
KEGG_CARDIAC_MUSCLE_CONTRACTION	KEGG_CARDIAC_MUSCLE_CONTRACTION	70	0.467368700811662	1.644014208643 31	0.001680137851811 49	0.00792680422393115	0.00526035062915339	870	tags=20%, list=5%, signal=19%	UQCRQ/COX7A2/COX7B/COX7C/COX6C/COX6A1/UQCR11/COX7A2L/UQCRC2/UQCRB/COX5B/UQCR10/COX4I1/SLC9A6
KEGG_ENDOMETRIAL_CANCER	KEGG_ENDOMETRIAL_CANCER	52	-0.419343182	-1.72437635	0.002536351222020 19	0.0116672156212929	0.00774254583564058	2506	tags=40%, list=16%, signal=34%	TP53/MYC/APC2/ERBB2/MAP2K2/ELK1/PIK3R5/MAP2K1/AXIN1/EGF/MAPK3/AKT1/TCF7/PIK3R2/AKT2/CASP9/ARAF/CTNNA1/ILK/GRB2/PIK3CD
KEGG_RN	KEGG_RNA_DEGRA	54	0.504363852361158	1.7150966	0.002678084	0.01193139184	0.00791785728	3508	tags=50%, list=22%,	LSM1/LSM3/LSM7/LSM5/WDR61/LSM8/EXOSC1/EXOSC3/C1D/LSM6/X

A_DEGRADATION	DATION			5274383	69228662	64578	941136		signal=39%	RN1/PAPOLA/EXOSC8/DCP2/CNOT6/MPHOSPH6/EXOSC7/CNOT7/DCP1A/TTC37/PNPT1/CNOT8/PAPOLG/LSM2/DIS3/HSPD1/EXOSC6
KEGG_PANCREATIC_CANCER	KEGG_PANCREATIC_CANCER	69	-0.372289875	-1.63669667	0.00272346987799 58	0.01193139184 64578	0.00791785728 941136	3160	tags=39%, list=20%, signal=32%	RALGDS/E2F2/VEGFC/IKBKB/TP53/BCL2L1/ERBB2/PIK3R5/MAP2K1/IKBKG/EGF/MAPK3/AKT1/RALBP1/PGF/PIK3R2/RAC1/JAK1/AKT2/CASP9/ARAF/RELA/NFKB1/TGFB1/STAT3/PIK3CD/RAC2
KEGG_CELL_ADHESION_MOLECULES_CAMS	KEGG_CELL_ADHESION_MOLECULES_CAMS	124	-0.30549237	-1.480556121	0.00311457621865869	0.0133274889356558	0.00884432904196837	3412	tags=35%, list=21%, signal=28%	ICAM2/NRXN3/CD8B/CD40LG/CDH2/PTPRC/NRXN2/CADM3/NFASC/PECAM1/CLDN6/ICOSLG/NEO1/CD6/CNTNAP1/CD276/CD40/HLA-A/ITGAM/HLA-B/CLDN7/HLA-C/MPZL1/PVR/CNTN2/CLDN17/ICAM1/NLGN2/ESAM/CLDN15/ICAM3/HLA-G/SELP/JAM3/HLA-F/CD4/ITGAL/F11R/HLA-E/CLDN5/ITGB2/SELPLG/GLG1
KEGG_NOTCH_SIGNALING_PATHW	KEGG_NOTCH_SIGNALING_PATHWAY	46	-0.40155977	-1.59894024	0.00451894552918 78	0.0188974085766035	0.0125406143872197	3312	tags=41%, list=21%, signal=33%	RFNG/HDAC1/DTX4/DLL4/MFNG/DVL2/CTBP1/JAG2/NOTCH1/RBPJ/CREBBP/PTCRA/NUMBL/MAML3/EP300/MAML1/DVL3/NCSTN/NCOR2

AY										
KEGG_NUCLEOTIDE_EXCISION_REPAIR	KEGG_NUCLEOTIDE_EXCISION_REPAIR	43	0.511168181169842	1.67603457033372	0.00545931628165832	0.0223225376850029	0.0148135833607571	4206	tags=60%, list=26%, signal=45%	POLE4/RBX1/GTF2H5/RPA3/CCNH/CUL4B/ERCC8/XPA/RFC3/ERCC4/GTF2H1/RFC4/MNAT1/PCNA/RPA4/POLE2/CDK7/RFC2/POLE3/CUL4A/RPA2/RFC5/CETN2/DDB2/POLD4/ERCC6
KEGG_ECM_RECEPTOR_INTERACTION	KEGG_ECM_RECEPTOR_INTERACTION	81	-0.332473353	-1.492840606	0.00629114841334708	0.0251595227678703	0.0166962508070535	3603	tags=33%, list=22%, signal=26%	COL5A3/LAMC3/COL6A2/COL11A1/COL6A6/ITGA3/COL6A3/COL4A2/LAMA4/SV2B/COL1A1/LAMC1/SV2A/HSPG2/ITGB4/COL11A2/ITGA2/DAG1/ITGA5/ITGA2B/ITGB3/GP9/GP6/VWF/ITGB5/THBS1/GP1BA
KEGG_LYSOSOME	KEGG_LYSOSOME	116	-0.293799732	-1.463196973	0.00642661722874948	0.0251595227678703	0.0166962508070535	2729	tags=32%, list=17%, signal=27%	AP1M2/CLN3/SLC11A1/PSAPL1/IDS/ATP6V0D2/GGA1/NAGPA/SORT1/HEXA/GALNS/MCOLN1/GAA/CTSD/MAN2B1/DNASE2/GUSB/ARSA/CTNS/ATP6V0A1/PSAP/AP1G1/SGSH/HGSNAT/AP1B1/ATP6V0C/IGF2R/SMPD1/AP1M1/ATP6V0D1/LAPTM5/GGA3/AP3D1/TPP1/CTSA/ATP6AP1/LAMP1

KEGG_UBIQUITIN_MEDIATED_PROTEOLYSIS	KEGG_UBIQUITIN_MEDIATED_PROTEOLYSIS	124	0.394079724743491	1.4788488466 4103	0.00659197862122389	0.0252692513813583	0.0167690684224117	2162	tags=29%, list=13%, signal=25%	RBX1/RNF7/ANAPC10/HUWE1/UBE2E1/CUL5/UBA3/CDC26/FBXO4/UBE2N/BIRC2/CUL4B/KLHL9/ERCC8/UBA6/ANAPC13/UBE2W/FANCL/RCHY1/MAP3K1/UBE2A/ANAPC4/MDM2/BIRC3/CUL2/UBE2Q2/TRIM37/HERC4/ANAPC11/SMURF2/UBA2/SKP2/SKP1/UBE4A/NEDD4/UBE2J1
KEGG_T_CELL_RECEPTOR_SIGNALING_PATHWAY	KEGG_T_CELL_RECEPTOR_SIGNALING_PATHWAY	107	-0.298595851	-1.443724443	0.00708437162346826	0.0261028499212638	0.0173222573848204	4034	tags=44%, list=25%, signal=33%	FOS/PLCG1/NFATC2/CSF2/CD247/PRKCQ/CD8B/TEC/PAK3/CD40LG/CHP2/CBLC/PTPRC/JUN/IKBKB/MAPK11/GRAP2/NFAT5/MAP3K14/MAP2K2/FYN/PIK3R5/NFKBIA/MAP2K1/IKBKG/PAK4/MAPK12/MAPK3/AKT1/CD3E/CBL/CARD11/NFKBIB/VAV1/CHP1/PIK3R2/NCK2/CD4/NFATC3/AKT2/RHOA/NFATC1/RELA/GRB2/NFKB1/PTPN6/PIK3CD
KEGG_NATURAL_KILLER_CEL	KEGG_NATURAL_KILLER_CELL_MEDIATED_CYTOTOXICI	122	-0.292052164	-1.41815632	0.00709316573947385	0.0261028499212638	0.0173222573848204	2545	tags=29%, list=16%, signal=24%	NCR1/NFAT5/HLA-A/SH3BP2/PRF1/HLA-B/MAP2K2/HLA-C/FYN/PIK3R5/TNFRSF10C/MAP2K1/ICAM1/SYK/MAPK3/SHC3/HLA-G/PLCG2/VAV1/NCR2/CHP1/PIK3R2/RAC1/NFATC3/PRKCB/ARAF/ITGAL/HLA-E/SHC1/

L_MEDIATED_CYTOTOXICITY	TY									ITGB2/NFATC1/GRB2/PTPN6/PIK3CD/RAC2
KEGG_DNA_REPLICATION	KEGG_DNA_REPLICATION	36	0.519698010569454	1.65545361230281	0.00828721885408389	0.0293240051759891	0.0194598661350958	3644	tags=50%, list=23%, signal=39%	POLE4/RPA3/RNASEH2C/DNA2/SSBP1/PRIM1/RFC3/RFC4/PCNA/RPA4/POLE2/RNASEH2B/RFC2/POLE3/FEN1/PRIM2/RPA2/RFC5
KEGG_GLIOMA	KEGG_GLIOMA	63	-0.363684963	-1.552866286	0.00813841508399953	0.0293240051759891	0.0194598661350958	2196	tags=32%, list=14%, signal=28%	CAMK2B/MAP2K2/PIK3R5/CAMK2G/MAP2K1/EGF/MAPK3/SHC3/AKT1/CALM1/PLCG2/CDKN1A/PIK3R2/PDGFA/AKT2/PRKCB/ARAF/SHC1/GRB2/PIK3CD
KEGG_VASCULAR_SMOOTH_MUSCLE_CONTRA	KEGG_VASCULAR_SMOOTH_MUSCLE_CONTRACTION	103	-0.302104579	-1.435609416	0.0107361788384267	0.0372727718164247	0.0247347913655908	3213	tags=35%, list=20%, signal=28%	RAMP1/GNAQ/PRKCH/GNA11/RAMP2/PLA2G2D/GUCY1A2/RAMP3/ITPR3/PLA2G2A/ARHGEF12/GNA12/ADCY1/MAP2K2/ARHGEF11/MAP2K1/AVPR1B/ITPR2/ADCY3/MAPK3/PTGIR/PLA2G6/MYLK/ARHGEF1/MYL9/ADCY6/CALM1/NPR1/PRKCD/PLCB2/PRKCB/ARAF/RHOA/GNAS/PRKACA/PPP1CA

CTION										
KEGG_TASTE_TRANSDUCTION	KEGG_TASTE_TRANSDUCTION	43	-0.380033539	-1.488587733	0.0119769969598629	0.040810508159533	0.0270824882523216	3923	tags=33%, list=24%, signal=25%	GRM4/TAS2R5/TAS2R4/SCNN1A/KCNB1/ITPR3/TAS1R1/CACNA1B/GNB3/ADCY6/PLCB2/GNB1/GNAS/PRKACA
KEGG_GALACTOSE_METABOLISM	KEGG_GALACTOSE_METABOLISM	24	-0.47247819	-1.599233953	0.0124464166622188	0.0416389211972409	0.0276322360347823	3624	tags=54%, list=23%, signal=42%	MGAM/GCK/GALT/G6PC2/B4GALT1/PFKM/GAA/GALK2/GANC/PGM1/GALE/PFKL/HK1

S5 Table Results of Gene Set Variation Analysis (GSVA) Comparing RA and Control Groups.

	logFC	AveExpr	t	P.Value	adj.P.Val	B	change
FONTAINE_FOLLICULAR_THYROID_ADENOMA_UP	0.277624288884315	0.00258194421858658	9.81924941476955	9.9102987100294 5e-20	1.2302644818630 6e-16	34.1311491778854	Up
LAIHO_COLORECTAL_CANCER_SERRATED_DN	0.315482116147977	0.00232318800664897	9.65961471	3.1963540340136 2e-19	3.2144138833174 2e-16	32.989406930255	Up
CHARAFE_BREAST_CANCER_LUMINAL_VS_BASAL_UP	0.221037244188257	-0.001586775	9.58388386749547	5.5530468730809 8e-19	4.3084702426517 1e-16	32.4509517568757	Up
REACTOME_IRE1ALPHA_ACTIVATES_CHAPERONES	0.338824035546244	0.00159041422530147	9.33016894097437	3.4788337322638 6e-18	1.9630109978328 9e-15	30.6624938142039	Up
JIANG_TIP30_TARGETS_DN	0.397319649464323	0.00185322249185823	9.17311017214855	1.0699698562835 7e-17	4.7437877842515 1e-15	29.5677401341495	Up
DEBIASI_APOPTOSIS_BY_REOVIRUS_INFECTION_DN	0.277999924067327	0.00175808772992029	8.98310516762551	4.1110174694013 7e-17	1.5449935959640 6e-14	28.2565103498285	Up

BILD_MYC_ONCOGENIC_SIGNATURE	0.200759926947058	-0.001973278	8.42251447109639	1.99545727747733e-15	3.75327373372781e-13	24.4771038455187	Up
LUI_THYROID_CANCER_PAX8_PPARG_UP	0.240217822389736	-0.002444483	8.40704394516674	2.21674297152656e-15	3.93123532121867e-13	24.374788772683	Up
LI_INDUCED_T_TO_NATURAL_KILLER_DN	0.177886383264801	0.00554852227373049	8.39948146079064	2.33358887949481e-15	4.02349615972897e-13	24.3248141248252	Up
LUI_THYROID_CANCER_CLUSTER_1	0.196780874843292	0.00997721084647106	8.2293253543584	7.36087422371624e-15	1.17151144375915e-12	23.2074460872928	Up
WP_RETT_SYNDROME_CAUSING_GENES	0.255263675924486	0.00274914630091612	8.21215021529409	8.2596118178431e-15	1.25042464764273e-12	23.0954220426671	Up
BLANCO_MELO_COVID19_SARS_COV_2_POS_PATIENT_LUNG_TISSUE_DN	0.25813706195106	-0.001656733	8.09806554045797	1.76900392927947e-14	2.28754320604952e-12	22.3549128807288	Up
MYLLYKANGAS_AMPLIFICATION_HOT_SPOT_22	0.436225897496214	0.0046553428732502	8.0483009557	2.4612644439539	3.0554136807243	22.03388	Up

		3	5134	e-14	7e-12	00528245	
BIOCARTA_BARRESTIN_PATHWAY	0.353098114018966	-0.003978276	8.04175382894827	2.5703314773405 1e-14	3.0819490983577 4e-12	21.99173488951 78	Up
REACTOME_LOSS_OF_FUNCTION_OF_MECP2_IN_RETT_SYNDROME	0.422538832827415	0.00906139811748845	7.98697994040211	3.6912401081214 4e-14	3.8833097205270 8e-12	21.63997467949 84	Up
BHAT_ESR1_TARGETS_NOT_VIA_AKT1_UP	0.175288651641186	-0.001358242	7.91331595604692	5.9916853868110 6e-14	6.0967854419567 6e-12	21.16925847815 6	Up
GINESTIER_BREAST_CANCER_ZNF217_AMPLIFIED_DN	0.323333403387717	-0.003224986	7.90645589858408	6.2673062553013 4e-14	6.2743822462347 4e-12	21.12556108570 14	Up
MCCABE_HOXC6_TARGETS_CANCER_UP	0.327530035871938	-0.007633282	7.89691064162912	6.6717706898153 2e-14	6.4705751049505 8e-12	21.06479894866 6	Up
NIKOLSKY_BREAST_CANCER_17Q11_Q21_AMPLICON	0.212178367077503	0.00114399385833919	7.86945848169015	7.9844179274252 1e-14	7.6245049346966 5e-12	20.89030398197 54	Up

BIOCARTA_MTA3_PATHWAY	0.329831869347162	0.00793203846518706	7.84581519120532	9.31731964275002e-14	8.30650966859998e-12	20.7403261176325	Up
REACTOME_RUNX1_INTERACTS_WITH_CO_FACTORS_WHOSE_PRECISE_EFFECT_ON_RUNX1_TARGETS_IS_NOT_KNOWN	0.318231972774005	0.0101765459512909	7.84253635264449	9.51874006442315e-14	8.32152388448937e-12	20.7195497473641	Up
MARTINEZ_RB1_AND_TP53_TARGETS_DN	0.0774482720708902	0.00111105390080182	7.80574940166125	1.20956586442783e-13	1.01456423250048e-11	20.4868257852291	Up
REACTOME_CHREBP_ACTIVATES_METABOLIC_GENE_EXPRESSION	0.402009590955607	-0.006219094	7.78739048059744	1.36284092429052e-13	1.11304652856201e-11	20.3709419851305	Up
LOPEZ_TRANSLATION_VIA_FN1_SIGNALING	0.209727238672196	0.000726780560373768	7.74963398863892	1.74089703810027e-13	1.34017076667572e-11	20.1331643673536	Up
GRADE_COLON_VS_RECTAL_CANCER_DN	0.221798751006789	-0.001680741	7.74663430794484	1.77503549638682e-13	1.34361528366744e-11	20.1143050066169	Up
WEST_ADRENOCORTICAL_TUMOR_DN	0.139216051967076	-0.001081418	7.7199242171	2.1096823122394	1.5226509432639	19.94658	Up

			5243	5e-13	9e-11	1362 7342	
REACTOME_REGULATION_OF_MECP2_EXPRESSION_AND_ACTIVITY	0.291503854868328	0.00852447231014874	7.69993545101895	2.4001983299922e-13	1.68275360915273e-11	19.8213066391618	Up
REACTOME_DEACTIVATION_OF_THE_BETA_CATENIN_TRANSACTIVATING_COMPLEX	0.247480773937222	0.00773762426515569	7.69562444280407	2.46785403118252e-13	1.70199666350554e-11	19.7943157883094	Up
CHIANG_LIVER_CANCER_SUBCLASS_INTERFERON_DN	0.209694313428827	-0.003498086	7.66948366574916	2.92033916545775e-13	1.94909088172003e-11	19.6308589947914	Up
FAELT_B_CLL_WITH_VH3_21_UP	0.392954115151519	0.00157378578691733	7.65297248495504	3.24738986609889e-13	2.09964051029957e-11	19.5278003282105	Up
CHEN_HOXA5_TARGETS_9HR_DN	0.22841673358892	-0.000454259	7.6377241538143	3.58141597578605e-13	2.29173700636124e-11	19.4327515465184	Up
BHAT_ESR1_TARGETS_VIA_AKT1_UP	0.190222624618192	-0.003434469	7.57890950729691	5.21874078679874e-13	3.14492466637474e-11	19.0672894495132	Up

CROMER_METASTASIS_DN	0.204852184330465	-0.00344898	7.55234617990632	6.1823767517296 9e-13	3.6546678569510 6e-11	18.9028337457609	Up
MILI_PSEUDOPODIA_CHEMOTAXIS_DN	0.164220881109991	0.00193900597679449	7.53907872187799	6.7274471013717 4e-13	3.9393645432277 7e-11	18.8208351836517	Up
MEISSNER_NPC_ICP_WITH_H3_UNMETHYLATED	0.325931810922209	-0.008591557	7.45981890268732	1.1122893020316 8e-12	6.1097165466465 7e-11	18.3329497820058	Up
MAYBURD_RESPONSE_TO_L663536_UP	0.27442433929984	0.00525162295656556	7.38258273102865	1.8096760854283 5e-12	8.9148090970268 e-11	17.8608045423119	Up
REACTOME_TRANSCRIPTIONAL_REGULATION_BY_MECP2	0.216115826025314	0.00513951505496469	7.36912899334121	1.9691468553212 4e-12	9.4748019619991 6e-11	17.7788962216494	Up
RASHI_RESPONSE_TO_IONIZING_RADIATION_3	0.22824461618815	0.00489197724701448	7.36459172526955	2.0259925905580 8e-12	9.5994931370946 7e-11	17.7512951609332	Up
LIN_APC_TARGETS	0.300910686638315	-0.00194421	7.3212105398	2.6580551753781	1.1955469908385	17.48797	Up

			1529	9e-12	8e-10	3781 3413	
SHEDDEN_LUNG_CANCER_GOOD_SURVIVAL_A5	0.230574514716609	0.000497828219384707	7.31156735737257	2.8230394858024 1e-12	1.2516147205982 6e-10	17.4295819634059	Up
WELCSH_BRCA1_TARGETS_DN	0.294818607925676	-0.000208229	7.29935193059856	3.0465910988514 7e-12	1.3317035880683 9e-10	17.3556888615145	Up
BIOCARTA_CARDIACEGF_PATHWAY	0.315282976402529	0.0062705383061635 8	7.26194654723622	3.8453914486591 1e-12	1.6019023303239 7e-10	17.1299355775967	Up
BAKER_HEMATOPOESIS_STAT5_TARGETS	0.449435867441887	-0.004510397	7.25401888926054	4.0395228097147 2e-12	1.6626493162484 4e-10	17.0821902532919	Up
PID_BETA_CATENIN_NUC_PATHWAY	0.16673813982721	0.0056542336528867 2	7.24074515315995	4.3864168086936 5e-12	1.7795090935661 1e-10	17.0023265064705	Up
WP_MIRNAS_INVOLVED_IN_DNA_DAMAGE_RESPONSE	0.294120918817555	-0.001349869	7.21263998644561	5.2206925899804 9e-12	2.0571507493444 6e-10	16.8335542140151	Up

SAKAI_TUMOR_INFILTRATING_MONOCYTES_UP	0.278812295184958	0.00775850959218645	7.2080461148547	5.3711834120989 6e-12	2.0967883923835 4e-10	16.8060102720553	Up
HOLLEMAN_DAUNORUBICIN_B_ALL_UP	0.372051318382291	-0.00111725	7.20093372591847	5.6126503702814 e-12	2.1773575530210 4e-10	16.763389339597	Up
MARKS_ACETYLATED_NON_HISTONE_PROTEINS	0.356881064268626	0.00105326401972626	7.18802181729713	6.0786531886481 4e-12	2.3290247124653 7e-10	16.6860880246418	Up
VERRECCHIA_RESPONSE_TO_TGFB1_C5	0.332778476828162	-0.006751865	7.16497924545986	7.0068700160834 2e-12	2.6519294018188 9e-10	16.548371270018	Up
LUI_THYROID_CANCER_CLUSTER_2	0.22631689213588	-0.002482298	7.15512378001276	7.4452832191772 9e-12	2.7911986190941 1e-10	16.489561076273	Up
MARCHINI_TRABECTEDIN_RESISTANCE_DN	0.275668175165039	0.00283006284951389	7.15017636573629	7.6754439240637 7e-12	2.8395515905962 7e-10	16.4600594016514	Up
BIOCARTA_THELPER_PATHWAY	0.346031349586101	0.0056736310960443	7.1499616160	7.6855915453548	2.8395515905962	16.45877	Up

		9	6785	1e-12	7e-10	9154 9299	
WP_NCRNAS_INVOLVED_IN_WNT_SIGNALING_IN_HEPATOCELLULAR_CARCINOMA	0.158648130821611	4.01797436328348e-05	7.13178863621595	8.59396220056124e-12	3.13780725758139e-10	16.3505349332562	Up
WANG_CLIM2_TARGETS_UP	0.109667741347757	0.000420994927259545	7.12749287694356	8.82368411152417e-12	3.18422135350177e-10	16.3249755714946	Up
ZHAN_VARIABLE_EARLY_DIFFERENTIATION_GENES_UP	0.285469181796338	-0.002997969	7.1230881268435	9.06551414048398e-12	3.23388771666575e-10	16.2987786934355	Up
HOFMANN_MYELODYSPLASTIC_SYNDROM_HIGH_RISK_UP	0.361535964150044	-0.004858907	7.11448791311363	9.55665188478868e-12	3.38960789993619e-10	16.2476616911141	Up
VERHAAK_GLIOBLASTOMA_CLASSICAL	0.179556909975414	-0.003335972	7.08560074646925	1.140606924295e-11	4.02258362448809e-10	16.0762762152188	Up
WP_LNCRNA_INVOLVEMENT_IN_CANONICAL_WNT_SIGNALING_AND_COLORECTAL_CANCER	0.142859672146611	9.96174985909832e-05	7.07580314123235	1.21100985957781e-11	4.24674474485845e-10	16.0182568006118	Up

XU_CREBBP_TARGETS_DN	0.274891249593906	0.00825458233985794	7.0747038246994	1.2191714315719 1e-11	4.2513466717791 1e-10	16.0117503271334	Up
WEIGEL_OXIDATIVE_STRESS_RESPONSE	0.224620678537082	0.000710782791545966	7.05660738727564	1.3615756010027 e-11	4.6435712941888 8e-10	15.9047441006076	Up
WP_SPLICING_FACTOR_NOVA_REGULATED_SYNAPTIC_PROTEINS	0.196449573601257	0.000181695006781773	7.02431887578755	1.6574596597634 8e-11	5.5311032839526 4e-10	15.7142895977962	Up
BARRIER_COLON_CANCER_RECURRENCE_DN	0.281579963362223	-0.002855194	7.00318152753406	1.8845584027119 e-11	6.1243214689176 6e-10	15.589938617591	Up
BIOCARTA_TERT_PATHWAY	0.315682865430728	0.00436327479747361	6.99676525299469	1.9593661658918 e-11	6.3014434153836 2e-10	15.5522432119034	Up
BARRIER_CANCER_RELAPSE_NORMAL_SAMPLE_DN	0.335686572711872	-0.003393563	6.99175895344416	2.0197581810109 e-11	6.4621850667704 3e-10	15.5228480572997	Up
BORCZUK_MALIGNANT_MESOTHELIOMA_DN	0.203898202127117	-0.004887338	6.9893107299	2.0499550402879	6.5002644741833	15.50847	Up

			2868	1e-11	8e-10	83182396	
BROWNE_HCMV_INFECTION_24HR_UP	0.127629214914523	0.00229136367834171	6.98376566990379	2.12000161285221e-11	6.67961929491048e-10	15.4759447913665	Up
SHANK_TAL1_TARGETS_DN	0.372737300446312	-0.005317644	6.97996509451546	2.16936676272245e-11	6.80063610920115e-10	15.4536567522192	Up
MCCLUNG_COCAINE_REWARD_5D	0.165447231861007	0.00354722204781064	6.97865170418711	2.18668788690179e-11	6.81807502157207e-10	15.44595649	Up
KASLER_HDAC7_TARGETS_1_UP	0.172699583595018	0.00011115634977445	6.97711885024311	2.20707551458095e-11	6.81807502157207e-10	15.4369708154132	Up
WP_SUDDEN_INFANT_DEATH_SYNDROME_SIDS_SUSCEPTIBILITY_PATHWAYS	0.156229308634388	-0.000951734	6.97705841415398	2.2078831630997e-11	6.81807502157207e-10	15.4366165638849	Up
MARTINEZ_TP53_TARGETS_DN	0.07878022999911	0.00184320231356041	6.96639814838293	2.35499016840787e-11	7.19916298662267e-10	15.3741639463627	Up
LINDGREN_BLADDER_CANCER_HIGH_RECURRENCE	0.230639863614111	-0.0057144	6.9611	2.430914	7.360333	15.3	Up

		99	5002219811	23711049e-11	98524138e-10	434425483218	
KAAB_HEART_ATRIUM_VS_VENTRICLE_UP	0.152816983667673	-0.002706051	6.95234913159197	2.5636781833035 4e-11	7.6503608095024 5e-10	15.2919603306119	Up
REACTOME_RUNX1_REGULATES_GENES_INVOLVED_IN_MEGAKARYOCYTE_DIFFERENTIATION_AND_PLATELET_FUNCTION	0.266920578735377	0.000267089456445142	6.95152608808398	2.5764529132103 7e-11	7.6516953264577 9e-10	15.2871481328886	Up
RADAEVA_RESPONSE_TO_IFNA1_DN	0.319518115839493	-0.007936124	6.94371132880052	2.7009123919676 4e-11	7.9831253414014 8e-10	15.2414763901592	Up
BIOCARTA_VDR_PATHWAY	0.333326192201414	0.0107924737016857	6.93270854150279	2.8862298621982 5e-11	8.42594337271e-10	15.1772338145262	Up
LIAO_METASTASIS	0.116272774338103	0.00138649465217126	6.91608588883239	3.1901710761275 6e-11	9.1673110507054 5e-10	15.0803136139014	Up
WP_ALANINE_AND_ASPARTATE_METABOLISM	0.319764837429723	-1.56E-06	6.89969400071658	3.5206674803605 5e-11	9.9784397491314 7e-10	14.984898814	Up

						9749	
REACTOME_HDL_CLEARANCE	0.417889814642762	-0.013263899	6.89848121194151	3.5464167777422 3e-11	1.0005731336111 8e-09	14.9778456670806	Up
BIOCARTA_MEF2D_PATHWAY	0.306564164977418	0.0115188782235586	6.89654322263018	3.5879482422494 3e-11	1.0077101692145 8e-09	14.9665768199945	Up
VERRECCHIA_DELAYED_RESPONSE_TO_TGFB1	0.280758953084932	-0.000738011	6.89497710109763	3.6218597822460 3e-11	1.0126524174955 4e-09	14.9574719026714	Up
REACTOME_REGULATION_OF_RUNX1_EXPRESSION_AND_ACTIVITY	0.261870756450182	-0.002685509	6.88533931299295	3.8375987512901 2e-11	1.0681603340474 3e-09	14.9014729801803	Up
JOHANSSON_BRAIN_CANCER_EARLY_VS_LATE_DN	0.233573251786191	0.000824248396641338	6.88350679913171	3.8800264255358 4e-11	1.0751483938973 7e-09	14.8908316699846	Up
HASLINGER_B_CLL_WITH_17P13_DELETION	0.295906961012704	-0.002564189	6.87386282959167	4.1110300268778 9e-11	1.1241041135167 9e-09	14.8348625277297	Up
REACTOME_NEGATIVE_REGULATION_OF_TCF_DEPEN	0.453799572057111	-0.0052648	6.8441	4.909869	1.313601	14.6	Up

DENT_SIGNALING_BY_DVL_INTERACTING_PROTEINS		92	9377625466	33927548e-11	6805553e-09	630241970596	
FERRANDO_T_ALL_WITH_MLL_ENL_FUSION_UP	0.189663262414758	0.00277121441282674	6.83781710964935	5.1005336191776e-11	1.3529492382151 9e-09	14.6261601443063	Up
ROYLANCE_BREAST_CANCER_16Q_COPY_NUMBER_UP	0.197594892605887	0.00473526134807354	6.83704289583769	5.1241729250799 1e-11	1.3534358019562 1e-09	14.6216860027379	Up
MILI_PSEUDOPODIA_HAPTOTAXIS_DN	0.14929594457687	0.00282679939034471	6.82666425358304	5.4516655233037 9e-11	1.4338342331841 8e-09	14.5617429857359	Up
CAFFAREL_RESPONSE_TO_THC_8HR_3_DN	0.435613017485307	0.00581411983789432	6.77131326674618	7.5779862356529e-11	1.9436595274668 4e-09	14.243148800674	Up
KIM_GASTRIC_CANCER_CHEMOSENSITIVITY	0.181517237364924	0.000117585724088231	6.77043602596118	7.6175308616098 8e-11	1.9457618953914 6e-09	14.2381143479062	Up
REACTOME_REGULATION_OF_TP53_ACTIVITY_THROUGH_ASSOCIATION_WITH_CO_FACTORS	0.286514324630045	0.0104967796103171	6.7451716922536	8.8476569310494 9e-11	2.2055183361857 1e-09	14.093322863	Up

						6366	
ULE_SPLICING_VIA_NOVA2	0.221804083456053	0.00160381457699509	6.72943969867927	9.71021492383721e-11	2.37288598552195e-09	14.0033571336728	Up
REACTOME_ION_HOMEOSTASIS	0.174096718922826	-0.002831341	6.71743063353492	1.04237875584651e-10	2.47894442051313e-09	13.9347826653666	Up
LEE_CALORIE_RESTRICTION_NEOCORTEX_DN	0.139895390484677	-0.000849724	6.71300690527803	1.06994281532394e-10	2.53478437202889e-09	13.9095442848085	Up
WP_GABA_RECEPTOR_SIGNALING	0.273784462389448	-0.00128166	6.70659492522246	1.11117149842136e-10	2.62244923600812e-09	13.8729836234256	Up
CHANDRAN_METASTASIS_TOP50_UP	0.264123705399272	0.0108456163137198	6.69366729862859	1.19909916163081e-10	2.78756872518443e-09	13.7993473959355	Up
MOREAUX_B_LYMPHOCYTE_MATURATION_BY_TACI_UP	0.225834862241133	-0.002079933	6.6715158047612	1.36592274195923e-10	3.12851751267193e-09	13.673408972702	Up
WOOD_EBV_EBNA1_TARGETS_UP	0.152551333886347	0.0042079	6.6698	1.379558	3.148132	13.6	Up

		3733759613	2451787158	49997586e-10	2093199e-09	638057939605	
REACTOME_SYNTHESIS_OF_IP3_AND_IP4_IN_THE_CYTOSOL	0.260504488852422	-0.003464127	6.657462697	1.4833677926402 2e-10	3.3239219815587 9e-09	13.59366822999 54	Up
WP_22Q112_DELETION_SYNDROME	0.175516010829477	-0.00600856	6.65132957948935	1.53768203619478e-10	3.42092917514732e-09	13.5589054045059	Up
PID_NFKAPPAB_ATYPICAL_PATHWAY	0.313684565537731	0.0134675169555132	6.64239671089668	1.6203022406484e-10	3.57908042978813e-09	13.5083147792327	Up
REACTOME_UPTAKE_AND_ACTIONS_OF_BACTERIAL_TOXINS	0.224984761051931	0.00321703361015916	6.64079861386074	1.63553615378926e-10	3.59991947041488e-09	13.4992692504643	Up
LEE_CALORIE_RESTRICTION_NEOCORTEX_UP	0.155227660100175	-0.001357365	6.60313387437141	2.03821907137482e-10	4.39048923721852e-09	13.2865353492223	Up
REACTOME_CLEC7A_DECTIN_1_INDUCES_NFAT_ACTIVATION	0.333929718600218	0.00942637369697581	6.60262925589606	2.044226501621e-10	4.39048923721852e-09	13.283691162	Up

						7534	
ODONNELL_METASTASIS_UP	0.203179972849666	-0.001019068	6.59727975579907	2.10899080500413e-10	4.49845564490056e-09	13.2535494043968	Up
REACTOME_LOSS_OF_MECP2_BINDING_ABILITY_TO_THE_NCOR_SMRT_COMPLEX	0.399149161377779	0.0119336151613616	6.58801577310755	2.2259453352427e-10	4.71550945250902e-09	13.2013933946679	Up
DOUGLAS_BMI1_TARGETS_UP	0.0686821871545924	-0.000825858	6.58637119452166	2.24736312863682e-10	4.74468807464242e-09	13.1921400118284	Up
SENGUPTA_EBNA1_ANTICORRELATED	0.238597690962511	-0.000309303	6.5745762424566	2.40701599409277e-10	5.0304202947252e-09	13.1258237091016	Up
NIKOLSKY_BREAST_CANCER_21Q22_AMPLICON	0.312010511404561	-0.001806954	6.5643384664844	2.55458212522198e-10	5.28543041708427e-09	13.0683325418657	Up
YANG_BREAST_CANCER_ESR1_UP	0.181169714093037	0.00160646201590192	6.54602813517463	2.84097328896627e-10	5.85844558292812e-09	12.9656717745079	Up
BIOCARTA_CSK_PATHWAY	0.252842546011144	0.0016719	6.5196	3.309679	6.691589	12.8	Up

		9251406386	519651576	56576666e-10	92335951e-09	181556848799	
REACTOME_L1CAM_INTERACTIONS	0.199973579539453	-0.001157049	6.50456120759685	3.6111531284890 6e-10	7.2072114046725 5e-09	12.733952079072	Up
POMEROY_MEDULLOBLASTOMA_PROGNOSIS_UP	0.172708988738681	-0.004022697	6.49700011911682	3.7721800563755 4e-10	7.5044620544624 9e-09	12.6918162951909	Up
BURTON_ADIPOGENESIS_2	0.137987998116356	0.00337235785318513	6.47171122079364	4.3636499101975 1e-10	8.5173506266025 e-09	12.5511494277123	Up
REACTOME_FORMATION_OF_THE_BETA_CATENIN_TCF_TRANSACTIVATING_COMPLEX	0.297508181424548	0.00982915339897884	6.46267359909513	4.5963695626143 3e-10	8.9434689263784 2e-09	12.5009762276498	Up
BIOCARTA_BARR_MAPK_PATHWAY	0.272125210629495	0.0041850711065957	6.45352726855732	4.8442779508330 6e-10	9.3038020032145 5e-09	12.4502519662158	Up
KASLER_HDAC7_TARGETS_2_DN	0.239339950679969	0.00161064185207927	6.44576704953164	5.0648673514156 e-10	9.6139546330998 9e-09	12.407256305	Up

						5137	
SCHLINGEMANN_SKIN_CARCINOGENESIS_TPA_DN	0.267314936498613	-0.003968844	6.42957317429204	5.5574058405552 7e-10	1.0452975167371 7e-08	12.3176564469361	Up
LEIN_NEURON_MARKERS	0.15808691787099	-0.001062838	6.42155228265342	5.8184681660960 6e-10	1.0878081899686 2e-08	12.2733387796039	Up
MCBRYAN_PUBERTAL_BREAST_5_6WK_DN	0.180672835229601	0.00657246242925199	6.4112636813619	6.1710111099847 2e-10	1.1468103580741 1e-08	12.2165511300939	Up
CHIARETTI_ACUTE_LYMPHOBLASTIC_LEUKEMIA_ZAP70	0.18314775581765	0.00470176079858856	6.38627760933827	7.1167225236775 1e-10	1.2916227106569 1e-08	12.0789212950558	Up
HUNSBERGER_EXERCISE_REGULATED_GENES	0.221781058500261	9.47235842864958e-05	6.37406129781607	7.6294709772525 9e-10	1.3806450832596 8e-08	12.0117753925657	Up
AMIT_EGF_RESPONSE_480_HELA	0.20713549259902	-0.000404497	6.35383296280674	8.5592472905152 3e-10	1.5354695934169 9e-08	11.9008016645032	Up
HANN_RESISTANCE_TO_BCL2_INHIBITOR_UP	0.19840656366176	-0.0051532	6.3453	8.980278	1.601740	11.8	Up

		5	727090 1847	1281118 e-10	9868158 e-08	5446 5907 0394	
REACTOME_SUMOYLATION_OF_DNA_METHYLATION_PROTEINS	0.2216248390757	-0.0051414 66	6.3397 817531 7566	9.269590 9598661 7e-10	1.639212 2817062 5e-08	11.8 2387 0109 0048	Up
MCBRYAN_PUBERTAL_BREAST_6_7WK_UP	0.18153672797231	0.0068548 711588595	6.3383 279500 0211	9.346305 1616376 2e-10	1.648082 8448376 3e-08	11.8 1591 7639 0678	Up
BYSTRYKH_HEMATOPOIESIS_STEM_CELL_IL3RA	0.31215713770549	0.0087641 212722102	6.3167 751779 3832	1.055934 3926764 2e-09	1.830777 8702074 2e-08	11.6 9818 0702 5457	Up
MATZUK_SPERMATOZOA	0.100294670725201	0.0002353 076092253 87	6.3139 077572 5363	1.073193 8388666 8e-09	1.850365 0438459 7e-08	11.6 8253 9270 0428	Up
MALONEY_RESPONSE_TO_17AAG_UP	0.213853710022307	0.0011745 099287022 3	6.3019 429211 8599	1.148245 3381777 5e-09	1.963404 6319750 2e-08	11.6 1732 9674 974	Up
REACTOME_THE_PHOTOTRANSDUCTION_CASCADE	0.175342993540513	-0.0024774	6.2939 633812 0592	1.201133 2583914 7e-09	2.042584 6944755 7e-08	11.5 7389 1647	Up

						3702	
PARENT_MTOR_SIGNALING_UP	0.13972991376594	0.0032941323045652 7	6.2844510946 6729	1.2673055729485 3e-09	2.1492255987135 3e-08	11.5221635404876	Up
REACTOME_PRE_NOTCH_EXPRESSION_AND_PROCESSI NG	0.196389928414106	0.0019334804248829 4	6.2689916484 4479	1.3825460246098 7e-09	2.3130628503378 6e-08	11.4382193651142	Up
PID_RHOA_REG_PATHWAY	0.234331370881238	0.0046327971491116 7	6.2312849086 639	1.7084249651743 8e-09	2.7832529550754 3e-08	11.2341230117427	Up
WNT_SIGNALING	0.133093675796544	0.0012622266577516 4	6.2221642505 2334	1.7979182809100 1e-09	2.9061663462522 e-08	11.1848941724595	Up
GRADE_COLON_AND_RECTAL_CANCER_DN	0.154682635069812	0.0028361444766889 1	6.2199539790 613	1.8202878285429 8e-09	2.9338189245334 6e-08	11.1729723685512	Up
REACTOME_NEF_MEDIATED_CD4_DOWN_REGULATIO N	0.360345947906691	0.0058722511422751 4	6.2195428243 5328	1.8244789831962 6e-09	2.9338189245334 6e-08	11.1707550257751	Up
IVANOVA_HEMATOPOIESIS_STEM_CELL_AND_PROGE	0.0963258894485352	0.0015072	6.2174	1.846268	2.961185	11.1	Up

NITOR		512078244	2005495267	53447571e-09	73475212e-08	593087588847	
REACTOME_NF_KB_IS_ACTIVATED_AND_SIGNALS_SURVIVAL	0.291874373887444	0.0168442105562439	6.18763338446164	2.1802780683356 4e-09	3.4260723975086 8e-08	10.99900515968 49	Up
NIKOLSKY_BREAST_CANCER_12Q24_AMPLICON	0.299416874589582	-0.004045732	6.18592324365877	2.2011548231008 3e-09	3.4501434310572 8e-08	10.98981926486 42	Up
YANG_BCL3_TARGETS_DN	0.361277439796938	0.00403422083807936	6.16921696376861	2.4156751454063 6e-09	3.7485239068843 1e-08	10.90018351316 7	Up
KEGG_ALANINE_ASPARTATE_AND_GLUTAMATE_METABOLISM	0.170173913264476	0.000339809296850993	6.1558495784313	2.6019474480831 6e-09	3.9975959926366 8e-08	10.82859412689 61	Up
PID_HDAC_CLASSI_PATHWAY	0.182042175579555	0.00510318190321691	6.14944340151616	2.6961315732184 8e-09	4.1320712777696 5e-08	10.79432730078 26	Up
BIOCARTA_TCYTOTOXIC_PATHWAY	0.319933845567537	0.00487734446563732	6.14438527694254	2.7728473125003 e-09	4.2287624738794 5e-08	10.767290321	Up

						0595	
DOANE_RESPONSE_TO_ANDROGEN_UP	0.112602889663534	-0.001900002	6.14198305459233	2.8100259551145e-09	4.26450638224834e-08	10.7544557222609	Up
REACTOME_ROLE_OF_PHOSPHOLIPIDS_IN_PHAGOCYTOSIS	0.206164076292805	0.00688421348878077	6.14023243796219	2.83742682688521e-09	4.29558739377475e-08	10.7451049178482	Up
NIKOLSKY_BREAST_CANCER_17Q21_Q25_AMPLICON	0.0992531098888418	-8.98E-05	6.1240006402898	3.10426886226102e-09	4.64293899471184e-08	10.6585000311102	Up
KEGG_ARRHYTHMOGENIC_RIGHT_VENTRICULAR_CARDIOMYOPATHY_ARVC	0.195160885640386	-0.002595506	6.11367713088325	3.2866021199232e-09	4.85712841865793e-08	10.6035093287416	Up
WP_ARRHYTHMOGENIC_RIGHT_VENTRICULAR_CARDIOMYOPATHY	0.195160885640386	-0.002595506	6.11367713088325	3.2866021199232e-09	4.85712841865793e-08	10.6035093287416	Up
PID_IL2_STAT5_PATHWAY	0.225303920975809	0.0125611399250592	6.11000734495449	3.35391036528646e-09	4.94482699224062e-08	10.5839782830241	Up
REACTOME_EFFECTS_OF_PIP2_HYDROLYSIS	0.211247159862431	-0.0052541	6.0903	3.737194	5.458063	10.4	Up

		77	9258402227	81857014e-09	11502703e-08	7973728	
BANDRES_RESPONSE_TO_CARMUSTIN_MGMT_48HR_DN	0.183367777673504	-0.001461391	6.07940564389803	3.9702842646033 9e-09	5.7713242225745 2e-08	10.4214595348269	Up
GINESTIER_BREAST_CANCER_20Q13_AMPLIFICATION_DN	0.190833759409495	0.00198718452381943	6.07488836641807	4.0701933580602 1e-09	5.8889720684102 e-08	10.3975219011883	Up
LEE_AGING_CEREBELLUM_DN	0.167499125281159	-0.004538937	6.0638202873084	4.3255008931555 e-09	6.2438102427479 6e-08	10.3389280493559	Up
ZAMORA_NOS2_TARGETS_DN	0.186474786732803	0.00441369604211379	6.06071212498664	4.3999717407587 6e-09	6.3365718317609 4e-08	10.3224882485396	Up
DORN_ADENOVIRUS_INFECTION_24HR_UP	0.229866560343866	0.00157274438441738	6.05984113212652	4.4210645847105 2e-09	6.3522101567819 9e-08	10.3178825160174	Up
ST_GAQ_PATHWAY	0.236882976288146	0.00116496972586498	6.05133963333957	4.6322105260103 9e-09	6.6096852264244 8e-08	10.2729538796012	Up

LEE_LIVER_CANCER_SURVIVAL_UP	0.135164826759657	-0.003828704	6.04752520764277	4.7301234575600 8e-09	6.7031681052683 6e-08	10.2528110914452	Up
HENDRICKS_SMARCA4_TARGETS_UP	0.205844836785376	-0.000386906	6.04440805689259	4.8116388115078 2e-09	6.7722997966052 3e-08	10.236357597188	Up
REACTOME_DAG_AND_IP3_SIGNALING	0.16938942666835	-0.001198784	6.01993724965477	5.5011231740448 7e-09	7.6047820804669 3e-08	10.1074168379794	Up
REACTOME_NR1H2_AND_NR1H3_MEDIATED_SIGNALING	0.227253580456798	0.000976130509313775	6.00686344227935	5.9081760509946 9e-09	8.0775437772079 4e-08	10.0386929161946	Up
MULLIGHAN_NPM1_MUTATED_SIGNATURE_2_DN	0.184014254391268	0.000665690029287196	6.00202944867536	6.0660224722025 7e-09	8.2389062330331 2e-08	10.0133114930899	Up
WP_INITIATION_OF_TRANSCRIPTION_AND_TRANSLATION_ELONGATION_AT_THE_HIV1_LTR	0.235683879760241	0.00957996199034517	5.99418718037999	6.3309128854510 7e-09	8.5426035391293 e-08	9.97216812949763	Up
KRASNOSELSKAYA_ILF3_TARGETS_DN	0.132551010899499	0.0002379025044651	5.9933463753	6.3599755787444	8.5632035612292	9.967759	Up

		09	2869	5e-09	4e-08	4134 5936	
BIOCARTA_CALCINEURIN_PATHWAY	0.24797096423159	0.0112875555468367	5.98716058896815	6.5778368687367 7e-09	8.7992744491916 3e-08	9.93 5339 1711 6403	Up
CERVERA_SDHB_TARGETS_1_DN	0.201957069627105	-0.002119869	5.98445229919464	6.6755032198101 e-09	8.9107201043787 8e-08	9.92 1152 8818 5664	Up
WP_ETHANOL_METABOLISM_RESULTING_IN_PRODUCTION_OF_ROS_BY_CYP2E1	0.258637123188762	-0.001049794	5.97476705440812	7.0365128251856 4e-09	9.2926883204100 6e-08	9.87 0460 9696 8882	Up
VALK_AML_CLUSTER_13	0.207132256344459	-0.000770491	5.97308515585164	7.1011202021538 6e-09	9.3581004447492 6e-08	9.86 1664 4587 1414	Up
WEIGEL_OXIDATIVE_STRESS_BY_TBH_AND_H2O2	0.19862605309627	0.00343710052126642	5.96849021734283	7.2805983175193 e-09	9.5742952874665 9e-08	9.83 7642 1436 4592	Up
BIOCARTA_PKC_PATHWAY	0.333746323125646	0.0158591198372151	5.95841527187213	7.6897881124134 8e-09	1.0091017931025 5e-07	9.78 5020 1696 4745	Up

WP_FRAGILE_X_SYNDROME	0.130591969243665	0.0034015222700931 2	5.9535 72949	7.894374 1817114 e-09	1.031586 9588606 9e-07	9.75 9752 8150 3796	Up
WP_LIPID_METABOLISM_PATHWAY	0.150015089194039	0.0014789857322110 6	5.9483 318367 0354	8.121805 6943497 e-09	1.059076 6374964 e-07	9.73 2422 4008 2935	Up
DELASERNA_MYOD_TARGETS_UP	0.140548934173015	-0.0002010 49	5.9427 567791 5956	8.370757 8343942 6e-09	1.084703 4212543 9e-07	9.70 3370 9506 3121	Up
MOOTHA_GLYCOLYSIS	0.292094307905996	0.0060371010956409 6	5.9323 152021 5213	8.857246 3205989 7e-09	1.142971 4742610 8e-07	9.64 9016 7424 0035	Up
REACTOME_COMPETING_ENDOGENOUS_RNAS_CERNAS_REGULATE_PTEN_TRANSLATION	0.316197393949835	0.0051596663333627 4	5.9300 204509 7533	8.967809 3472998 e-09	1.153094 3460666 2e-07	9.63 7081 1734 2386	Up
URS_ADIPOCYTE_DIFFERENTIATION_DN	0.243972255415185	-0.0006923 65	5.9295 339185 9238	8.991423 6103792 8e-09	1.153094 3460666 2e-07	9.63 4551 056	Up
REACTOME_NEF_AND_SIGNAL_TRANSDUCTION	0.341374444200574	0.0228717482907284	5.9265 839063 3794	9.135911 8103105 4e-09	1.169208 3424040 7e-07	9.61 9213 5204	Up

						1996	
TURJANSKI_MAPK8_AND_MAPK9_TARGETS	0.304516787421061	0.00394107947686629	5.91910124750076	9.51265783898502e-09	1.20813304641082e-07	9.58033654465747	Up
BIOCARTA_HSWI_SNF_PATHWAY	0.313457672865446	0.0137123348858753	5.91899887208061	9.51791621870297e-09	1.20813304641082e-07	9.57980490501686	Up
FONTAINE_THYROID_TUMOR_UNCERTAIN_MALIGNANCY_UP	0.163102704194846	0.00431465397181895	5.91481012838769	9.73551855002999e-09	1.23072023706795e-07	9.55805868498323	Up
BILANGES_SERUM_SENSITIVE_VIA_TSC2	0.209619240984785	0.00674406652403721	5.90701064842639	1.01537244707779e-08	1.27579287024531e-07	9.51759873170553	Up
LIU_CMYB_TARGETS_DN	0.332881875033336	0.0130234136562513	5.88592964218925	1.13738924663201e-08	1.41762551282026e-07	9.40844738199367	Up
MCBRYAN_TERMINAL_END_BUD_DN	0.298157647542797	-8.61E-05	5.88096535813716	1.16814324695995e-08	1.44723854967673e-07	9.38278773162151	Up
BIOCARTA_PITX2_PATHWAY	0.247865540754086	0.0130394	5.8679	1.252705	1.536668	9.31	Up

		679592609	4657750078	5983742e-08	70535744e-07	557533744291	
ACEVEDO_LIVER_TUMOR_VS_NORMAL_ADJACENT_TISSUE_DN	0.109119976228003	-0.001672389	5.86322277441948	1.2848437329896e-08	1.5729832447073 8e-07	9.29121621773493	Up
BANDRES_RESPONSE_TO_CARMUSTIN_WITHOUT_MGMT_48HR_DN	0.209201836911299	-0.0014523	5.84973057653175	1.38113707643751e-08	1.6710950942393e-07	9.22172525018191	Up
BIOCARTA_PELP1_PATHWAY	0.390604014836177	0.0016885428585053	5.84822192489359	1.39233273368913e-08	1.68136367276429e-07	9.21396274046001	Up
REACTOME_WNT_MEDIATED_ACTIVATION_OF_DVL	0.356608730242868	0.00275388655475173	5.84498948935607	1.41661976924421e-08	1.70612488142354e-07	9.19733603574977	Up
DING_LUNG_CANCER_MUTATED_FREQUENTLY	0.253865426381451	-6.94E-05	5.80603151986361	1.74393303524688e-08	2.06971173035897e-07	8.99751075422096	Up
KEGG_BASAL_CELL_CARCINOMA	0.155960350175865	-0.002050846	5.80504894326188	1.75307703150933e-08	2.07394378046836e-07	8.9924843402	Up

						4394	
REACTOME_INTERACTION_BETWEEN_L1_AND_ANKYRINS	0.211557397298579	-0.006335596	5.80493059564535	1.7541815446204 1e-08	2.0739437804683 6e-07	8.99187897259824	Up
IVANOVA_HEMATOPOIESIS_STEM_CELL_SHORT_TERM	0.206889940657583	-0.000144299	5.79533724942392	1.8460103774780 8e-08	2.1590361303472 e-07	8.94283946317118	Up
DELACROIX_RAR_BOUND_ES	0.0818306659505561	-0.000709021	5.79523391398058	1.8470246257682 7e-08	2.1590361303472 e-07	8.94231157523074	Up
MULLIGHAN_NPM1_MUTATED_SIGNATURE_1_DN	0.129223922407858	0.000637168377740288	5.78577657866378	1.9421892587220 2e-08	2.2533025661472 1e-07	8.89403002231028	Up
REACTOME_A_TETRASACCHARIDE_LINKER_SEQUENCE_IS_REQUIRED_FOR_GAG_SYNTHESIS	0.198499900674448	-0.009521521	5.7831432441664	1.9695282839204 7e-08	2.2765106253807 e-07	8.88059730269031	Up
COLLIS_PRKDC_SUBSTRATES	0.227294749382514	0.00537418945606124	5.77705636506199	2.0341672621393 4e-08	2.3425002219107 3e-07	8.8495662483449	Up
RIGGINS_TAMOXIFEN_RESISTANCE_UP	0.160436441162184	-0.0053820	5.7678	2.135440	2.418737	8.80	Up

		13	8929876106	83098279e-08	45217339e-07	288057707659	
BIOCARTA_EGFR_SMRTE_PATHWAY	0.29989383327635	0.00818017361105242	5.7658049366499	2.1591455793597 9e-08	2.4411323517461 3e-07	8.79227351855492	Up
REACTOME_EGR2_AND_SOX10_MEDIATED_INITIATION_OF_SCHWANN_CELL_MYELINATION	0.182613715814349	-0.009844183	5.75497896555534	2.2864568636421 6e-08	2.5617396665391 5e-07	8.73722980937093	Up
REACTOME_FCGR3A_MEDIATED_IL10_SYNTHESIS	0.188460502337807	0.00244237176359727	5.75225783350795	2.3195916055070 6e-08	2.5895153049248 8e-07	8.7234071988332	Up
REACTOME_SIGNAL_TRANSDUCTION_BY_L1	0.225441973882776	0.00201295056074494	5.75044706775179	2.3419001467799 2e-08	2.6089497188937 1e-07	8.71421184122814	Up
CHEMELLO_SOLEUS_VS_EDL_MYOFIBERS_UP	0.258427683333403	-0.012763468	5.7458978523329	2.3988740108435 e-08	2.6636513390528 8e-07	8.69112021526974	Up
MYLLYKANGAS_AMPLIFICATION_HOT_SPOT_29	0.348839525087312	-0.005788268	5.74362721114793	2.4278150459364 4e-08	2.6861761123221 9e-07	8.6795998989	Up

						8268	
YAO_HOXA10_TARGETS_VIA_PROGESTERONE_UP	0.118696851734439	-0.002978077	5.73291873751473	2.56895899911778e-08	2.81723118507493e-07	8.62531759600481	Up
WP_IL18_SIGNALING_PATHWAY	0.10975135263396	0.00357716584648103	5.73246407466364	2.57512561806608e-08	2.81901317660249e-07	8.62301462534981	Up
REACTOME_TRANSPORT_OF_VITAMINS_NUCLEOSIDES_AND_RELATED_MOLECULES	0.142111554358474	-0.001837247	5.71485347481537	2.82541711167015e-08	3.0713422087805e-07	8.53392338105746	Up
BOYAULT_LIVER_CANCER_SUBCLASS_G1_UP	0.128840891631397	0.00271819114529159	5.71312182526542	2.85127287039655e-08	3.0924490024712e-07	8.52517467992615	Up
REACTOME_FCERI_MEDIATED_CA_2_MOBILIZATION	0.199036746649058	0.00883931961649685	5.71111451761745	2.88153347574175e-08	3.11597182646848e-07	8.5150358991064	Up
RICKMAN_TUMOR_DIFFERENTIATED_MODERATELY_VS_POORLY_DN	0.298273841522324	-0.007742318	5.70829139470951	2.9246233980603e-08	3.15706737943657e-07	8.50078123172223	Up
BERTUCCI_MEDULLARY_VS_DUCTAL_BREAST_CANC	0.152056082376005	-0.0049456	5.7062	2.956683	3.186134	8.49	Up

ER_DN		27	1709466956	80384517e-08	7865394e-07	031109651686	
MCBRYAN_PUBERTAL_BREAST_4_5WK_DN	0.0959725518815933	0.00240006882417021	5.70529592399374	2.97103111277126e-08	3.19604681403314e-07	8.48566240086903	Up
NIKOLSKY_BREAST_CANCER_1Q32_AMPLICON	0.256277493246572	-0.002408706	5.70455610766642	2.9826029629125e-08	3.20294404685085e-07	8.48192933920235	Up
REACTOME_CARBOXYTERMINAL_POST_TRANSLATIONAL_MODIFICATIONS_OF_TUBULIN	0.221768324270945	-0.001862949	5.70055988161981	3.04587417050982e-08	3.25961051316456e-07	8.46177125010027	Up
WP_WNT_SIGNALING	0.119764467794961	0.00407431095997676	5.69553096455041	3.12735826403314e-08	3.34105210754797e-07	8.43641978454833	Up
REACTOME_ELEVATION_OF_CYTOSOLIC_CA2_LEVELS	0.207616281194909	-0.004122156	5.69518798665539	3.13299236534243e-08	3.34132020819252e-07	8.43469142755695	Up
MAGRANGEAS_MULTIPLE_MYELOMA_IGLL_VS_IGLK_DN	0.169657615190425	-0.002717295	5.67488115393227	3.48481623467989e-08	3.66614480824713e-07	8.3325061452	Up

						5738	
REN_ALVEOLAR_RHABDOMYOSARCOMA_UP	0.154155318787688	-0.000448678	5.66952683581536	3.5838232224265e-08	3.76392398335047e-07	8.30561084714536	Up
WP_AMPACTIVATED_PROTEIN_KINASE_AMPK_SIGNALING	0.135391498356386	0.00377523805577264	5.66148784750781	3.73764250137842e-08	3.90564764411714e-07	8.26526781544611	Up
REACTOME_GABA_SYNTHESIS_RELEASE_REUPTAKE_AND_DEGRADATION	0.25073327193805	-0.003900442	5.6598817382729	3.76913652312996e-08	3.93193788219625e-07	8.25721310185074	Up
MEISSNER_ES_ICP_WITH_H3K4ME3_AND_H3K27ME3	0.297906259242247	-0.001263857	5.65582742403496	3.84979249359352e-08	3.99593010162793e-07	8.23688855778941	Up
AMIT_SERUM_RESPONSE_20_MCF10A	0.207771457651722	0.0039434494579303	5.64518498110383	4.06960163851562e-08	4.19601617446287e-07	8.18359207688969	Up
FONTAINE_PAPILLARY_THYROID_CARCINOMA_DN	0.145277937826465	0.00143454448329613	5.64120484867956	4.15491169400101e-08	4.27687178850154e-07	8.16368030144987	Up
KIM_WT1_TARGETS_12HR_UP	0.117264636953179	6.3674093	5.6339	4.315609	4.413012	8.12	Up

		9234587e-05	1925659613	49715159e-08	87459965e-07	72607947972	
AUNG_GASTRIC_CANCER	0.13133536109524	0.00248424262919441	5.61048181666968	4.87474223516942e-08	4.9359747232784e-07	8.01035377762824	Up
KEGG_THYROID_CANCER	0.16797646668624	0.00832676918868125	5.60793239294192	4.93966008218831e-08	4.99356191044672e-07	7.99766044381258	Up
ALFANO_MYC_TARGETS	0.0940664916088136	0.00340702953574043	5.60570718033282	4.99701029298356e-08	5.03324337110922e-07	7.9865850677972	Up
JAZAG_TGFB1_SIGNALING_VIA_SMAD4_UP	0.135509417290503	0.00327186519848606	5.60546695958301	5.00324014817849e-08	5.03324337110922e-07	7.98538964455451	Up
KEGG_DILATED_CARDIOMYOPATHY	0.171485678401838	-0.006086453	5.59466446042001	5.29135056560929e-08	5.29732467108659e-07	7.93167464081809	Up
REACTOME_RMTS_METHYLATE_HISTONE_ARGININES	0.244177160340448	0.0100794801444904	5.58609964323366	5.53121076213619e-08	5.52853867964241e-07	7.8891449348	Up

QI_HYPOXIA_TARGETS_OF_HIF1A_AND_FOXA2	0.237540885239172	0.0035074490820706	5.57036514464122	5.9997640479524 8e-08	5.9527902980740 7e-07	21437.81114830522917	Up
PID_ARF_3PATHWAY	0.274889515799263	-0.001167583	5.56962029533384	6.0228777732773 9e-08	5.9623608195746 1e-07	7.80746039330499	Up
BIOCARTA_VIP_PATHWAY	0.202995261303245	0.00721754691749043	5.56467062864822	6.1786912677251 8e-08	6.1068689010780 6e-07	7.7829634851252	Up
MIKKELSEN_ES_ICP_WITH_H3K27ME3	0.210880725061302	-0.006930903	5.56369962010922	6.2097156298905 5e-08	6.1277750261892 9e-07	7.77815980123947	Up
REACTOME_INTERLEUKIN_2_SIGNALING	0.308338860172255	0.011779272419024	5.5530641824646	6.5596147649717 5e-08	6.4423305136360 2e-07	7.7255888696741	Up
WP_SOMITOGENESIS_IN_THE_CONTEXT_OF_SPONDYLOCOSTAL_DYSOSTOSIS	0.269300909295746	-0.001043186	5.54980324893357	6.6706921224284 2e-08	6.5410720385328 9e-07	7.7094861474423	Up
SCHLOSSER_SERUM_RESPONSE_AUGMENTED_BY_MY	0.211667488048243	0.0037911	5.5484	6.718764	6.567460	7.70	Up

C		9110836463	0833091766	77252874e-08	30599778e-07	260024608509	
KEGG_TIGHT_JUNCTION	0.127722229108795	-0.001010014	5.54581691500103	6.8089692022343 8e-08	6.6451685280296 9e-07	7.68981159302679	Up
MYLLYKANGAS_AMPLIFICATION_HOT_SPOT_15	0.243101054978876	0.00666583259653926	5.54264146163103	6.9211124646768 9e-08	6.7440102147958 3e-07	7.67414720973352	Up
MONNIER_POSTRADIATION_TUMOR_ESCAPE_UP	0.123060096357582	0.00365841304499233	5.53860536826339	7.0662483980415 6e-08	6.8638816598816 9e-07	7.65424765640796	Up
REACTOME_SUMOYLATION_OF_TRANSCRIPTION_COFACTORS	0.189964253979105	0.00429026637303261	5.53792626112673	7.0909583156672 6e-08	6.8771216039604 2e-07	7.65090052387868	Up
WP_NEURAL_CREST_DIFFERENTIATION	0.146884174657934	-0.000855148	5.53002198539422	7.3848244086738 5e-08	7.1287099696172 e-07	7.61196662367837	Up
REACTOME_PLATELET_CALCIUM_HOMEOSTASIS	0.176334041768624	-0.002948234	5.52270430452427	7.6674318207254 e-08	7.3557572351225 e-07	7.5759616981	Up

						5141	
RORIE_TARGETS_OF_EWSR1_FLI1_FUSION_DN	0.185474600742096	-0.000928445	5.52023407751344	7.76518780303156e-08	7.42665796792397e-07	7.56381612916957	Up
REACTOME_CARDIAC_CONDUCTION	0.160807589011558	-0.004596261	5.51769813752311	7.86680635541939e-08	7.5121949304751e-07	7.55135198226044	Up
WP_DISORDERS_OF_FOLATE_METABOLISM_AND_TRANSPORT	0.259810987784381	-0.00815465	5.51161359882538	8.11593076401104e-08	7.70268841777011e-07	7.52146514676825	Up
SUZUKI_AMPLIFIED_IN_ORAL_CANCER	0.226159931514843	-0.005964569	5.50156364407951	8.54432963126345e-08	8.05997781477998e-07	7.472158241	Up
BIOCARTA_TCR_PATHWAY	0.202365599345988	0.00829886140188838	5.50112912488884	8.5633399334846e-08	8.06565265055219e-07	7.47002803493323	Up
SIMBULAN_PARP1_TARGETS_UP	0.182040884019613	-0.006616499	5.4897451816734	9.0763209007237e-08	8.5229536809065e-07	7.41426691125742	Up
REACTOME_PECAM1_INTERACTIONS	0.218413959888964	0.0017959640069764	5.4887799878	9.1211645618727	8.5521251413208	7.409543	Up

		4	9139	e-08	2e-07	4300 8211	
WHITE_NEUROBLASTOMA_WITH_1P36.3_DELETION	0.223692192544185	-0.0038805 97	5.4878 397652 1401	9.165055 3663315 7e-08	8.580316 5397918 6e-07	7.40 4942 7929 1256	Up
HOFMANN_MYELODYSPLASTIC_SYNDROM_LOW_RIS K_UP	0.193685212537023	0.0018607 483940078 6	5.4843 606257 9029	9.329260 8703566 e-08	8.707777 7777899 9e-07	7.38 7924 3854 7248	Up
MCCOLLUM_GELDANAMYCIN_RESISTANCE_UP	0.250813449544378	0.0010392 306817077 6	5.4775 589804 0345	9.658590 5974814 6e-08	8.961266 3435825 7e-07	7.35 4678 7488 8058	Up
WP_CALCIUM_REGULATION_IN_THE_CARDIAC_CELL	0.13429077898779	-0.0001632 74	5.4711 915771 6274	9.977131 6663886 2e-08	9.204057 2058879 9e-07	7.32 3585 6311 8402	Up
REACTOME_HEPARAN_SULFATE_HEPARIN_HS_GAG_ METABOLISM	0.159721079912872	-0.0042997 1	5.4688 188699 4197	1.009842 6238115 2e-07	9.286063 9496268 3e-07	7.31 2006 7190 8456	Up
SCHAEFFER_PROSTATE_DEVELOPMENT_AND_CANCE R_BOX4_UP	0.284008531575745	0.0104922 990522286	5.4654 237457 9876	1.027448 6558251 9e-07	9.420049 9360516 3e-07	7.29 5445 3813 5054	Up

POOLA_INVASIVE_BREAST_CANCER_DN	0.146819972552645	-0.001538804	5.46111432272777	1.0502265629022 4e-07	9.5960725293423 1e-07	7.27443600864056	Up
REACTOME_LRR_FLII_INTERACTING_PROTEIN_1_LRRFIP1_ACTIVATES_TYPE_I_IFN_PRODUCTION	0.366228909967267	0.0177739010252702	5.45071178602019	1.1072536600146 8e-07	1.0047841327063 e-06	7.22377624858047	Up
MYLLYKANGAS_AMPLIFICATION_HOT_SPOT_8	0.214670440281536	0.00443724323933039	5.44977580998016	1.1125300004855 1e-07	1.0080983522647 6e-06	7.21922190551479	Up
REACTOME_FACTORS_INVOLVED_IN_MEGAKARYOCYTE_DEVELOPMENT_AND_PLATELET_PRODUCTION	0.130639744867807	-0.000990399	5.44264585235071	1.1535342789570 6e-07	1.0422106651363 1e-06	7.18454906212049	Up
VANTVEER_BREAST_CANCER_METASTASIS_UP	0.136171947769757	0.00277271429849939	5.439509392	1.1720342188262 4e-07	1.0527954263754 6e-06	7.16930808133448	Up
BROWNE_HCMV_INFECTION_1HR_DN	0.0773073604467424	-0.000999951	5.43212003166563	1.2167675976882 2e-07	1.0882530949352 7e-06	7.13342896308071	Up
KEGG_MELANOGENESIS	0.128503502498279	-0.003085711	5.4277221367	1.2441751757002	1.1063890136922	7.112093	Up

			9729	7e-07	e-06	57785387	
KEGG_ADHERENS_JUNCTION	0.186071734669143	0.00738277317451167	5.41193326976851	1.3476071461091e-07	1.1902978695928 6e-06	7.03561221607129	Up
CAMPS_COLON_CANCER_COPY_NUMBER_UP	0.127776988219781	-0.002099155	5.41115677064989	1.3529040724832 8e-07	1.1928232354976 9e-06	7.03185548391517	Up
ASTON_MAJOR_DEPRESSIVE_DISORDER_DN	0.0845677269355167	-0.000159403	5.41029240577232	1.3588241949674 6e-07	1.1963435146330 5e-06	7.02767416518904	Up
SA_G1_AND_S_PHASES	0.211703423920363	-0.006152553	5.40454964479562	1.3988015972965 2e-07	1.2280567912898 9e-06	6.99990754949864	Up
WP_TCELL_RECEPTOR_AND_COSTIMULATORY_SIGNALING	0.21118089963219	0.0104540872930534	5.40324173163362	1.4080651022505e-07	1.2327024103905 3e-06	6.99358703231247	Up
SPIELMAN_LYMPHOBLAST_EUROPEAN_VS_ASIAN_2FC_UP	0.236227746306628	0.00177725764999894	5.38525856706714	1.5416451240243 4e-07	1.3415987899923 7e-06	6.90680827898565	Up

DASU_IL6_SIGNALING_SCAR_UP	0.178955809167881	0.0049965101288057	5.38505055980069	1.54326008708644e-07	1.34159878999237e-06	6.9058058956958	Up
REACTOME_VISUAL_PHOTOTRANSDUCTION	0.119833887735284	-0.005845419	5.38302492770724	1.5590732897876e-07	1.35345005730232e-06	6.89604604708329	Up
REACTOME_RUNX3_REGULATES_YAP1_MEDIATED_TRANSCRIPTION	0.312759556741836	5.07111087059949e-05	5.37836295678963	1.59606804156024e-07	1.37977636963293e-06	6.87359513563945	Up
REACTOME_CHYLOMICRON_CLEARANCE	0.304003054371645	-0.016928618	5.37546366053774	1.61950413319333e-07	1.39808931220181e-06	6.85964076670786	Up
GARY_CD5_TARGETS_UP	0.12421611092633	0.00470401046766006	5.37485370693173	1.62447703825117e-07	1.40043458005903e-06	6.85670582297572	Up
TSUTSUMI_FBXW8_TARGETS	0.267508323311646	-0.004482195	5.36524844235157	1.70477346832026e-07	1.46164739192547e-06	6.81052323297019	Up
BIOCARTA_AGPCR_PATHWAY	0.23341682542251	0.0077217321983716	5.3649816850	1.7070576354989	1.4616473919254	6.809241	Up

			1855	3e-07	7e-06	6045 0576	
ZHAN_MULTIPLE_MYELOMA_UP	0.17528874355791	0.0023476083507086 4	5.3579733241 9056	1.7681453911249 5e-07	1.5034080058510 3e-06	6.77 5588 6064 4256	Up
ZHANG_TARGETS_OF_EWSR1_FLI1_FUSION	0.132770431971759	0.0030379932096338 3	5.3496488271 1132	1.8434689092625 8e-07	1.5608807743243 7e-06	6.73 5662 0378 5914	Up
NIKOLSKY_BREAST_CANCER_11Q12_Q14_AMPLICON	0.121100745644101	-0.0043212 32	5.3432570635 8698	1.9034117969834 6e-07	1.6052278565049 4e-06	6.70 5039 5377 7897	Up
ONGUSAHA_TP53_TARGETS	0.13753807058509	-0.0025899 17	5.3394616212 4625	1.9398978475306 1e-07	1.6315644904637 5e-06	6.68 6869 8811 7684	Up
KAMMINGA_SENESCENCE	0.144700840134286	-0.0015247 77	5.3387664044 6773	1.9466543006692 8e-07	1.6350315621453 6e-06	6.68 3542 8530 3401	Up
REACTOME_SUMOYLATION_OF_IMMUNE_RESPONSE_ PROTEINS	0.235891037409458	-0.0059930 88	5.3368339701 2774	1.9655549757459 9e-07	1.6486756397912 7e-06	6.67 4296 8447 677	Up

REACTOME_VLDL_ASSEMBLY	0.364987167899996	0.0125611835272594	5.33461117574822	1.9875161635574 3e-07	1.6626028069004 e-06	6.66366492576136	Up
REACTOME_TRANSPORT_OF_CONNEXONS_TO_THE_PLASMA_MEMBRANE	0.300844755335493	-0.004149143	5.33253371004851	2.0082569462613 6e-07	1.6754369442801 4e-06	6.65373138213791	Up
WP_THE_EFFECT_OF_PROGERIN_ON_THE_INVOLVED_GENES_IN_HUTCHINSONGILFORD_PROGERIA_SYNDROME	0.246084520203381	0.00696257473238976	5.32993909292457	2.0344563249191 1e-07	1.6927440226237 2e-06	6.64132945374985	Up
OZANNE_AP1_TARGETS_UP	0.20005715905993	0.0117205092286752	5.32171218074542	2.1197430211264 2e-07	1.7473100839484 3e-06	6.60203829962698	Up
MATTIOLI_MULTIPLE_MYELOMA_WITH_14Q32_TRANSLOCATIONS	0.176183480545522	0.00182665654113635	5.32091230662515	2.1282180633979 1e-07	1.7519694322958 6e-06	6.59822078619927	Up
REACTOME_PRESYNAPTIC_DEPOLARIZATION_AND_CALCIUM_CHANNEL_OPENING	0.25817451149025	-0.005332558	5.32033139910484	2.1343936784182 6e-07	1.7524049685108 6e-06	6.595448 6143077	Up
REACTOME_ASSEMBLY_OF_ACTIVE_LPL_AND_LIPC_LIPASE_COMPLEXES	0.19457058720009	-0.006348969	5.3190174872	2.1484261389975	1.7615959108002	6.589179	Up

			9759	3e-07	2e-06	3504 6673	
KANG_FLUOROURACIL_RESISTANCE_DN	0.196332368162545	-0.005871404	5.31288447990054	2.215120495077e-07	1.8114957724562 5e-06	6.55993265882 67	Up
REACTOME_NR1H3_NR1H2_REGULATE_GENE_EXPRESSION_LINKED_TO_CHOLESTEROL_TRANSPORT_AND_EFFLUX	0.231368294274912	0.00286374772928965	5.30596847810094	2.2927434194835 8e-07	1.8700470965485 6e-06	6.52698497890288	Up
REACTOME_REELIN_SIGNALLING_PATHWAY	0.322906900778098	0.00116200489656195	5.29898452655107	2.3738079517227 3e-07	1.9336254535883 2e-06	6.49374901010 24	Up
REACTOME_ACTIVATED_NTRK2_SIGNALS_THROUGH_FYN	0.29058517712242	0.00240705956047878	5.2903738282059	2.4775927030328 3e-07	2.0102507068921 2e-06	6.45282054578628	Up
KYNG_ENVIRONMENTAL_STRESS_RESPONSE_NOT_BY_4NQO_IN_OLD	0.208434053298237	-0.000502757	5.28628111105817	2.5284545238884 4e-07	2.0461691303488 3e-06	6.43338599999652	Up
BONOME_OVARIAN_CANCER_POOR_SURVIVAL_DN	0.245475837131919	0.00121689063558717	5.28235850785687	2.5781531443818 9e-07	2.0793077442712 9e-06	6.41477074992523	Up

BIOCARTA_IL2_PATHWAY	0.231476610862543	0.0094370309293480 1	5.2822568403 1268	2.5794537829690 6e-07	2.0793077442712 9e-06	6.41428842235628	Up
WP_MIR124_PREDICTED_INTERACTIONS_WITH_CELL_CYCLE_AND_DIFFERENTIATION	0.28316454234781	-0.0072006 42	5.2758004523 4102	2.6633688097439 7e-07	2.1368782798074 7e-06	6.38367375008486	Up
PEREZ_TP53_AND_TP63_TARGETS	0.134464396098145	0.0030804132934056 5	5.2719790744 9939	2.7142786018480 5e-07	2.1710731033081 e-06	6.36556805455911	Up
REACTOME_RECYCLING_PATHWAY_OF_L1	0.222032583447582	0.0044980669014247 1	5.2692016707 8942	2.7518718659202 e-07	2.1983099963663 7e-06	6.35241542355376	Up
DAZARD_UV_RESPONSE_CLUSTER_G5	0.307612881995303	-0.0032736 71	5.2641787975 2446	2.8211463373875 7e-07	2.2507526113322 1e-06	6.32864353816236	Up
BIOCARTA_SRCRPTP_PATHWAY	0.255629989515805	0.0092041089635785 2	5.2609439113 9282	2.8666544808075 6e-07	2.2811954310734 e-06	6.31334349976357	Up
NIKOLSKY_BREAST_CANCER_20Q12_Q13_AMPLICON	0.116625237464671	0.0002139489668173	5.2517106692	3.000502410538e-	2.3724991671604	6.269715	Up

		34	6515	07	3e-06	2727 9344	
VALK_AML_CLUSTER_9	0.159606750598791	0.0013042585307223 3	5.2487822670 3089	3.0442065706200 3e-07	2.4039936620659 7e-06	6.25 5891 2772 7152	Up
FINETTI_BREAST_CANCERS_KINOME_BLUE	0.194227214740894	-0.0016453 02	5.2481158516 4473	3.0542382871462 e-07	2.4088509591253 5e-06	6.25 2746 2356 025	Up
REACTOME_NEF_MEDIATED_CD8_DOWN_REGULATIO N	0.322377941380471	-0.0023920 58	5.2446782870 2423	3.1064966479784 3e-07	2.4438561082385 4e-06	6.23 6528 3769 7739	Up
BLANCO_MELO_HUMAN_PARAINFLUENZA_VIRUS_3_I NFECTION_A594_CELLS_DN	0.1899569912261	-0.0080507 55	5.2425773504 7845	3.1388616321277 2e-07	2.4648763259417 3e-06	6.22 6620 7827 1149	Up
ASTIER_INTEGRIN_SIGNALING	0.116914389444953	0.0002811391303072 25	5.2424290423 6751	3.1411586496212 5e-07	2.4648763259417 3e-06	6.22 5921 514	Up
HUTTMANN_B_CLL_POOR_SURVIVAL_UP	0.155211059168133	0.0017854981522799	5.2417976554 5551	3.1509559344496 4e-07	2.4694423592334 5e-06	6.22 2944 7231 8346	Up
NIKOLSKY_MUTATED_AND_AMPLIFIED_IN_BREAST_C	0.090041300024799	0.0033766	5.2363	3.237252	2.527500	6.19	Up

ANCER		6557574742	171414609	96289642e-07	52084252e-06	71181356705	
WP_REGULATORY_CIRCUITS_OF_THE_STAT3_SIGNALING_PATHWAY	0.141068882665413	0.00021566145713283	5.23575946796797	3.2461616878653 4e-07	2.5312720598718 8e-06	6.19449137159051	Up
PAPASPYRIDONOS_UNSTABLE_ATEROSCLEROTIC_PLAQUE_DN	0.166195369375685	-0.001176498	5.23501273400875	3.2581278883514 4e-07	2.5374152826847 4e-06	6.19097444963476	Up
REACTOME_HSF1_DEPENDENT_TRANSACTIVATION	0.14801512360049	-0.003107443	5.23459232328959	3.2648836769404 1e-07	2.5394903487179 3e-06	6.18899460541291	Up
AMBROSINI_FLAVOPIRIDOL_TREATMENT_TP53	0.126990718880548	-0.001856356	5.22970641956647	3.3444017274164 9e-07	2.5915981925186 2e-06	6.165994 9107386	Up
PID_RB_1PATHWAY	0.135769731525557	0.00877787091528525	5.22928101282254	3.3514134977071 3e-07	2.5937934638738 3e-06	6.16399320064482	Up
BLANCO_MELO_MERS_COV_INFECTION_MCR5_CELLS_UP	0.117861218485484	-0.001745521	5.21367483445165	3.6187388579902 5e-07	2.7902499492603 1e-06	6.0906519846	Up

						664	
HO_LIVER_CANCER_VASCULAR_INVASION	0.226597133778843	-0.002981034	5.21142770590686	3.6588961182026e-07	2.81771317688382e-06	6.08010639171351	Up
REACTOME_ACTIVATION_OF_THE_PHOTOTRANSDUCTION_CASCADE	0.253971863322617	0.00147157707739581	5.20920935142741	3.69896314551058e-07	2.84503893979977e-06	6.06969948651481	Up
WP_WNT_SIGNALING_PATHWAY_AND_PLURIPOTENCY	0.113723684572723	0.00307531364683952	5.20716701878617	3.73622710725313e-07	2.87014376914853e-06	6.06012155773285	Up
WP_PATHOGENESIS_OF_SARSCOV2_MEDIATED_BY_NSP9NSP10_COMPLEX	0.226040395993917	-0.0078052	5.20227306168855	3.82700943539577e-07	2.93624815395569e-06	6.03718289051054	Up
LIU_LIVER_CANCER	0.177591389107132	-0.006747692	5.19902679701039	3.88840403323754e-07	2.97760407246332e-06	6.02197694524122	Up
SHEDDEN_LUNG_CANCER_GOOD_SURVIVAL_A12	0.0877459848871448	-0.001811216	5.19826121119663	3.90302173791493e-07	2.9835044245367e-06	6.01839197138428	Up
HOFFMANN_SMALL_PRE_BII_TO_IMMATURE_B_LYMP	0.131599228990937	0.0004515	5.1969	3.927609	2.998607	6.01	Up

HOCYTE_DN		83527647666	7968357548	96577822e-07	01815318e-06	239198927922	
REACTOME_PTK6_REGULATES_CELL_CYCLE	0.270890936027265	-0.008238559	5.19627284644042	3.94123603892006e-07	3.00531352500944e-06	6.00908316879883	Up
CONRAD_STEM_CELL	0.128992754608438	0.0005328216516855	5.19211003423692	4.02242001290256e-07	3.05222017360467e-06	5.98960385242962	Up
MAGRANGEAS_MULTIPLE_MYELOMA_IGG_VS_IGA_UP	0.208726972020032	0.000334435338021597	5.19032678621648	4.05769146668223e-07	3.07147450410934e-06	5.98126330329031	Up
NADELLA_PRKAR1A_TARGETS_UP	0.241272227432927	0.00491264594973327	5.18839197141903	4.09630016531169e-07	3.09396201680863e-06	5.97221651036535	Up
WINNEPENNINCKX_MELANOMA_METASTASIS_DN	0.166824608306811	-0.000263268	5.18678510731907	4.12863535791587e-07	3.1100048139058e-06	5.96470525117013	Up
HOLLEMAN_PREDNISOLONE_RESISTANCE_B_ALL_UP	0.239246947197522	0.0125063662849109	5.18016329096212	4.26451937817285e-07	3.19298815202881e-06	5.9337718369	Up

						1284	
WP_LIVER_X_RECEPTOR_PATHWAY	0.251780437443254	0.000276062640967804	5.1776011541693	4.3182516693774e-07	3.2293238688946 4e-06	5.92181167718708	Up
KEGG_CALCIUM_SIGNALING_PATHWAY	0.136057102831247	-0.002485606	5.17139822009437	4.4510710480430 3e-07	3.3166624243941 2e-06	5.89287625644346	Up
REACTOME_REGULATION_OF_TP53_ACTIVITY_THROUGH_ACETYLATION	0.225732596911263	0.000447302831659058	5.15870638075368	4.7352925235351 7e-07	3.5115843122560 1e-06	5.83376027764815	Up
REACTOME_TRAFFICKING_OF_GLUR2_CONTAINING_AMPA_RECEPTORS	0.217320294294021	-0.004398174	5.15350237954207	4.8568505690352 1e-07	3.5931432040526 3e-06	5.80955564921238	Up
MOREAUX_MULTIPLE_MYELOMA_BY_TACI_UP	0.158221004371156	-0.002981713	5.15033470006672	4.9323187181103 7e-07	3.6359741429110 5e-06	5.79483211636178	Up
WP_IL7_SIGNALING_PATHWAY	0.185243482264752	0.014213917806328	5.14762635789244	4.9977440169665 4e-07	3.6668644621930 5e-06	5.78224951549546	Up
REACTOME_REGULATION_OF_LOCALIZATION_OF_FO	0.229422870554825	0.0145632	5.1467	5.018215	3.677457	5.77	Up

XO_TRANSCRIPTION_FACTORS		7014339	8596237381	9151573e-07	63699898e-06	834625631586	
WP_ECTODERM_DIFFERENTIATION	0.0974718418955348	2.38406534924094e-05	5.14316722963176	5.1073002797762e-07	3.7295309219495 1e-06	5.76154486515761	Up
PID_HIF2PATHWAY	0.164074714403404	0.0030663959212286	5.13921318462746	5.2063920798886 3e-07	3.7885200046739 4e-06	5.743197782 2886	Up
MULLIGHAN_NPM1_SIGNATURE_3_DN	0.101658472155156	0.0001794514262453	5.13895717968105	5.2128716329106 7e-07	3.7887932348333 2e-06	5.74201029964908	Up
HOWLIN_CITED1_TARGETS_1_UP	0.177257122821129	0.00357811103972263	5.13809509590976	5.2347487411121 7e-07	3.7958043733742 1e-06	5.73801187000076	Up
WP_PREIMPLANTATION_EMBRYO	0.126855468173379	0.00201238292208777	5.13663324461868	5.2720497506105 4e-07	3.8139408860186e-06	5.73123292258236	Up
REACTOME_NUCLEAR_RECEPTOR_TRANSCRIPTION_PATHWAY	0.163388066579378	-0.002163997	5.13495485929667	5.3151936859672e-07	3.8377495047265 9e-06	5.7234518160	Up

						966	
REACTOME_PLASMA_LIPOPROTEIN_ASSEMBLY_REMODELING_AND_CLEARANCE	0.146258355979309	-0.00218502	5.13350519196216	5.3527336046335e-07	3.85434077540141e-06	5.71673274813585	Up
REACTOME_ERCC6_CSB_AND_EHMT2_G9A_POSITIVELY_REGULATE_RRNA_EXPRESSION	0.254558006711421	0.00443516623530436	5.13284057950472	5.37002991018255e-07	3.86231467584045e-06	5.71365285636882	Up
PID_ALPHA_SYNUCLEIN_PATHWAY	0.187256928046934	0.000945888478498373	5.13048919443425	5.4316598786055e-07	3.9021195447343e-06	5.7027589018462	Up
MARTIN_INTERACT_WITH_HDAC	0.155596818483557	0.00671785843471572	5.13000537419666	5.44442562254217e-07	3.90676876752824e-06	5.70051787569362	Up
PID_REG_GR_PATHWAY	0.160449746342629	0.00739758733530332	5.12557491149225	5.56268530063375e-07	3.97783267984259e-06	5.68000434367409	Up
PEREZ_TP63_TARGETS	0.133332463151494	0.00103736134555511	5.12050980103228	5.70093659951193e-07	4.07019282752131e-06	5.6565702307877	Up
REACTOME_KILLING_MECHANISMS	0.218215885210667	0.0043075	5.1197	5.723089	4.073763	5.65	Up

		3480640733	0926859844	56410968e-07	40876477e-06	286825658928	
SCHAEFFER_PROSTATE_DEVELOPMENT_12HR_DN	0.162296777568202	0.00192790571294108	5.10516036891212	6.1405404218101 6e-07	4.3410403642569 1e-06	5.58567171501992	Up
CHENG_TAF7L_TARGETS	0.304505283969796	0.00482917664482502	5.10325207667361	6.1974373198621 7e-07	4.3713060732255 1e-06	5.5768696603205	Up
WP_IL9_SIGNALING_PATHWAY	0.262106724464671	0.0109582917712982	5.0814005298949	6.8864822964438 6e-07	4.8298752106245 2e-06	5.47627268033285	Up
WP_PATHWAYS_IN_CLEAR_CELL_RENAL_CELL_CARCINOMA	0.145667723723295	0.00576108101093583	5.07824689137815	6.9918614398210 6e-07	4.8926083990226 1e-06	5.46178390096469	Up
TUOMISTO_TUMOR_SUPPRESSION_BY_COL13A1_DN	0.206312814787615	-0.00983134	5.07724676328499	7.0256058605839 4e-07	4.9052795924234 5e-06	5.45719056150321	Up
IRITANI_MAD1_TARGETS_UP	0.207286576299003	0.00798453456485799	5.0765415091983	7.0494959950879 5e-07	4.9164293979225 8e-06	5.4539519551	Up

						7126	
FARMER_BREAST_CANCER_CLUSTER_8	0.31474516006075	-0.004559068	5.06061307527191	7.6105079238272 8e-07	5.2721453887495 4e-06	5.3809060262133	Up
KAUFFMANN_MELANOMA_RELAPSE_DN	0.29573981505182	0.00395056224558721	5.05166472225521	7.9443984863700 8e-07	5.472905816304e-06	5.33995338403242	Up
BIOCARTA_BARRESTIN_SRC_PATHWAY	0.206831810233104	0.00624994780275735	5.04969643305502	8.0197222202091 5e-07	5.5125598915656 9e-06	5.33095346084105	Up
STARK_BRAIN_22Q11_DELETION	0.262469244680219	-0.011671558	5.04034450888409	8.3871588950702 8e-07	5.7397017929108 3e-06	5.28823192045541	Up
ELVIDGE_HYPOXIA_BY_DMOG_DN	0.117245517037378	-0.005786564	5.03803405493712	8.4804174925763 5e-07	5.7971312088569 9e-06	5.27768740889583	Up
SCHLOSSER_SERUM_RESPONSE_UP	0.221616471416207	0.00544611773887294	5.03656516102437	8.5402287817169 2e-07	5.8251868184743 8e-06	5.27098571834862	Up
VERRECCHIA_RESPONSE_TO_TGFB1_C2	0.22702175045071	0.0027035	5.0345	8.624853	5.876450	5.26	Up

		1566767507	038111934	23644939e-07	49820432e-06	158373934664	
PID_ECADHERIN_STABILIZATION_PATHWAY	0.166603177550286	0.00521403425629283	5.03199413891515	8.72897892514715e-07	5.94087414346364e-06	5.25014124044553	Up
SCHAEFFER_SOX9_TARGETS_IN_PROSTATE_DEVELOPMENT_DN	0.141637453343897	0.00864732131538549	5.02738552966079	8.92336714492151e-07	6.06652134375989e-06	5.22914127364456	Up
KEGG_TASTE_TRANSDUCTION	0.172871100226203	-0.000945451	5.01799955155983	9.33230396824354e-07	6.32375663000957e-06	5.18642179895813	Up
WP_VALPROIC_ACID_PATHWAY	0.192425162421671	0.00178262492289408	5.01402721166716	9.51078570550124e-07	6.4166790080485e-06	5.16836202120026	Up
HEDENFALK_BREAST_CANCER_BRACX_DN	0.181267268387679	0.0149369944779179	5.01088074571593	9.65450064835753e-07	6.50656737506571e-06	5.15406542424966	Up
DELACROIX_RARG_BOUND_MEF	0.105471205533537	-0.003872666	5.00629659506528	9.86764993829227e-07	6.63225662124429e-06	5.1332497810	Up

						5211	
ST_WNT_CA2_CYCLIC_GMP_PATHWAY	0.160147300348367	0.0055623554246419	5.00618160396986	9.87305480591224e-07	6.63225662124429e-06	5.1327278349864	Up
SCHAEFFER_PROSTATE_DEVELOPMENT_6HR_DN	0.0863259610630666	0.00294891699869067	5.00022382723352	1.0157038916500 4e-06	6.79361428391355e-06	5.10569905983211	Up
REACTOME_BIOTIN_TRANSPORT_AND_METABOLISM	0.273238417966513	0.00212732372475087	4.99874569205037	1.02287105202493e-06	6.83418796546686e-06	5.09899731982474	Up
WEIGEL_OXIDATIVE_STRESS_BY_HNE_AND_TBH	0.145236260018809	0.0037389094882313	4.99830036619362	1.02503991814015e-06	6.84131480849025e-06	5.09697857337382	Up
SESTO_RESPONSE_TO_UV_C8	0.11748357564871	0.00693351888706614	4.9957480720885	1.0375564364722e-06	6.91741439439631e-06	5.08541142941506	Up
SANSOM_APC_MYC_TARGETS	0.134354764106646	0.00253622769472262	4.98598148732278	1.08683195755374e-06	7.22458360644591e-06	5.04119415028532	Up
WP_GLIAL_CELL_DIFFERENTIATION	0.287872509907745	-0.0053736	4.9859	1.087121	7.224583	5.04	Up

		65	2543940916	16778161e-06	60644591e-06	094060675903	
NIKOLSKY_BREAST_CANCER_7P22_AMPLICON	0.192502603914203	-0.008078574	4.98561709191897	1.08871358945691e-06	7.22742807460861e-06	5.03954578000735	Up
REACTOME_GLUCONEOGENESIS	0.229028178601551	0.00646971888271671	4.97924749214163	1.12211793739236e-06	7.44122439892561e-06	5.01074863053999	Up
BIOCARTA_PDZS_PATHWAY	0.21803257141885	-0.000455267	4.97649123930259	1.13687808810058e-06	7.53105901050194e-06	4.99829704393469	Up
SA_TRKA_RECEPTOR	0.226436442393627	0.00949642743432838	4.96934032771965	1.17605342093632e-06	7.72461754894365e-06	4.96601905745815	Up
WP_BIOMARKERS_FOR_UREA_CYCLE_DISORDERS	0.219165189056895	-0.00661252	4.96822586083891	1.18227531974443e-06	7.74908438189405e-06	4.960992033	Up
KEGG_LYSINE_DEGRADATION	0.14760169319585	0.00675069439345621	4.9675795494576	1.18589815210793e-06	7.76463062250414e-06	4.95807714810341	Up

BROWNE_HCMV_INFECTION_48HR_DN	0.0757600078763775	-0.001276991	4.96625033514023	1.1933827097744 7e-06	7.7925893311733 4e-06	4.95208334376161	Up
REACTOME_TOXICITY_OF_BOTULINUM_TOXIN_TYPE_D_BONT_D_	0.294911948795488	-0.008726726	4.95450540020354	1.2615321565134 9e-06	8.1736222291015 3e-06	4.89918029326591	Up
NUNODA_RESPONSE_TO_DASATINIB_IMATINIB_UP	0.161830894205216	0.00400791663344535	4.94875615832044	1.2962493392605 9e-06	8.3636378885556 1e-06	4.87332195785764	Up
GRAESSMANN_APOPTOSIS_BY_SERUM_DEPRIVATION_DN	0.0758969103727307	0.000299157294695611	4.94573562298954	1.3148574461181 7e-06	8.4748911402445 e-06	4.85974656439275	Up
WP_PRADERWILLI_AND_ANGELMAN_SYNDROME	0.10586638004831	-0.004359651	4.93902028748278	1.3571576216611 9e-06	8.7316013033020 9e-06	4.82959020892513	Up
MOTAMED_RESPONSE_TO_ANDROGEN_DN	0.289814008972921	0.005935959191451	4.93863528418914	1.3596222436904 7e-06	8.7362062801105 e-06	4.82786232455061	Up
BLANCO_MELO_INFLUENZA_A_INFECTION_A594_CELLS_DN	0.14871948996989	-0.003641197	4.9370991540	1.3694990254539	8.7905692357734	4.820969	Up

			0255	9e-06	3e-06	3350 9213	
PID_RXR_VDR_PATHWAY	0.206969204521713	0.0028766 390749958	4.9339 785598 5633	1.389777 4133373 2e-06	8.911516 9468850 9e-06	4.80 6971 9932 1009	Up
REACTOME_RUNX3_REGULATES_WNT_SIGNALING	0.259459189983065	0.0074166 769527673 8	4.9328 558379 7527	1.397143 9066995 8e-06	8.946107 6477534 7e-06	4.80 1937 8649 6344	Up
WP_GENE_REGULATORY_NETWORK_MODELLING_SOMITOGENESIS	0.241978506408466	0.0014233 442729483 5	4.9327 174494 1966	1.398054 5220429 9e-06	8.946107 6477534 7e-06	4.80 1317 4163 382	Up
MODY_HIPPOCAMPUS_POSTNATAL	0.155942349705341	0.0001771 762730143 05	4.9322 392150 2927	1.401205 7887092 2e-06	8.957038 4454357 6e-06	4.79 9173 4214 5636	Up
REACTOME_CARNITINE_METABOLISM	0.208490671897472	0.0062645 740147183 1	4.9274 815541 1023	1.432932 1270173 e-06	9.131632 1482509 3e-06	4.77 7853 6025 066	Up
WP_TUMOR_SUPPRESSOR_ACTIVITY_OF_SMARCB1	0.180017070978852	0.0044378 777116943 6	4.9172 981582 6408	1.503190 8208823 6e-06	9.511014 7046043 1e-06	4.73 2278 1088 5812	Up

FIGUEROA_AML_METHYLATION_CLUSTER_1_DN	0.138501722011125	0.0069089696898158 3	4.9167132745724	1.5073256737345 9e-06	9.5274648236971 7e-06	4.7296628763 5566	Up
REACTOME_TRANSPORT_OF_NUCLEOSIDES_AND_FREE_PURINE_AND_PYRIMIDINE_BASES_ACROSS_THE_PLASMA_MEMBRANE	0.240698493805986	0.0002276445987537 08	4.9135305132 2148	1.5300197069443 1e-06	9.6414541329982 9e-06	4.715436 134	Up
REACTOME_RUNX3_REGULATES_CDKN1A_TRANSCRIPTION	0.276385251530792	-0.0020486 48	4.9101346551 8798	1.5545972767628 2e-06	9.7764795307668 1e-06	4.7002653730 3733	Up
REACTOME_RHO_GTPASE_CYCLE	0.143417071097894	0.0039320876669934 1	4.8983789204 1835	1.6426611986362 8e-06	1.0288595418703 7e-05	4.6478153404 678	Up
HOELZEL_NF1_TARGETS_DN	0.107520912600105	-0.0011618 27	4.8978323421 8896	1.6468708500720 8e-06	1.0304563877416 7e-05	4.6453792616 7113	Up
BIOCARTA_CARM_ER_PATHWAY	0.161390999803833	0.0088719 70791499	4.8944900561 2843	1.6728402503143 8e-06	1.0445995406138 2e-05	4.6304877774 735	Up
KEGG_HYPERTROPHIC_CARDIOMYOPATHY_HCM	0.137153750659582	-0.0054070 43	4.8932266462 3277	1.6827595814492 1e-06	1.0497375600055 5e-05	4.6248609026	Up

						6543	
ZHAN_MULTIPLE_MYELOMA_MF_UP	0.136177160358014	0.0047748838081016	4.88805367836017	1.72396980450008e-06	1.07113919685005e-05	4.60183466137572	Up
WP_GPCRS_OTHER	0.149837241043638	-0.00080381	4.88208110895283	1.7727633103049e-06	1.09706299771311e-05	4.57527461120241	Up
WP_ONE_CARBON_METABOLISM_AND_RELATED_PATHWAYS	0.101722295502692	-0.005384652	4.88114140146524	1.78056082207816e-06	1.10079093850987e-05	4.57109820891133	Up
KEGG_PHENYLALANINE_METABOLISM	0.202706486779235	-0.00798984	4.87965542197921	1.79295886535911e-06	1.10625205539602e-05	4.56449535279231	Up
KEGG_PHOSPHATIDYLINOSITOL_SIGNALING_SYSTEM	0.138841534520436	0.0066263565467491	4.87126913057897	1.86450750384526e-06	1.14019685481454e-05	4.5272630560026	Up
REACTOME_CELL_JUNCTION_ORGANIZATION	0.172137916746833	-0.003771978	4.86160816901669	1.95035161590204e-06	1.19034734315673e-05	4.48443837443003	Up
ABBUD_LIF_SIGNALING_2_UP	0.202801373471084	0.0022199	4.8603	1.961658	1.193727	4.47	Up

		8413193197	6639489271	68990086e-06	00864849e-05	893907753554	
BIOCARTA_TCRA_PATHWAY	0.318927807534084	0.00406145780050136	4.85817970361456	1.9817238714082 7e-06	1.2047561282890 4e-05	4.46925801088562	Up
REACTOME_HCMV_EARLY_EVENTS	0.154367051101082	0.00203179121692694	4.85478401960243	2.0132762873117 5e-06	1.2227403048281 8e-05	4.45423167407682	Up
REACTOME_NEUROTOXICITY_OF_CLOSTRIDIUM_TOXINS	0.259981600395533	-0.010536012	4.85215529965926	2.0380347559120 1e-06	1.2353595439400 3e-05	4.44260532166326	Up
ACEVEDO_LIVER_CANCER_DN	0.090491360891448	0.000444687218479106	4.84510738561591	2.1058748471115 4e-06	1.2715141221810 7e-05	4.411459826797 3	Up
KEGG_WNT_SIGNALING_PATHWAY	0.101636309823381	0.00494000835887096	4.8438978872482	2.1177340663136 5e-06	1.2761917815154 2e-05	4.40611875395945	Up
WP_MAMMARY_GLAND_DEVELOPMENT_PATHWAY_PUBERTY_STAGE_2_OF_4	0.206003542037662	-0.001607364	4.83459040081787	2.2111771185946 1e-06	1.3268786591594 8e-05	4.3650550217	Up

						9666	
GARGALOVIC_RESPONSE_TO_OXIDIZED_PHOSPHOLIPIDS_SALMON_DN	0.210499077211277	-0.001802972	4.83445813369193	2.21253328859363e-06	1.32687865915948e-05	4.36447195144328	Up
SCHAEFFER_PROSTATE_DEVELOPMENT_AND_CANCER_BOX3	0.234637844125387	0.00414799887469908	4.83341703686385	2.22323600428521e-06	1.33201021994192e-05	4.35988297573076	Up
BIOCARTA_PPARG_PATHWAY	0.318239257335194	0.000971176208122251	4.82580635459285	2.30300799510721e-06	1.37317681322098e-05	4.3263616990499	Up
HAMAI_APOPTOSIS_VIA_TRAIL_DN	0.143122754929672	-0.003471708	4.81598593058451	2.41003253498649e-06	1.43149013824509e-05	4.28317342588609	Up
MATZUK_CENTRAL_FOR_FEMALE_FERTILITY	0.178380435668129	-0.004972796	4.81527473805205	2.4179669175979e-06	1.43482989077726e-05	4.28004862416566	Up
KLEIN_PRIMARY_EFFUSION_LYMPHOMA_UP	0.160513867907938	-0.000406247	4.81492482462542	2.42187995688549e-06	1.43577926383842e-05	4.27851133518383	Up
SCHAEFFER_PROSTATE_DEVELOPMENT_AND_CANCE	0.196827654021819	-0.0010486	4.8126	2.447959	1.448473	4.26	Up

R_BOX1_DN		42	066076 0805	1062381 4e-06	0383622 6e-05	8328 9983 6261	
REACTOME_IONOTROPIC_ACTIVITY_OF_KAINATE_RECEPTORS	0.228285758422449	-0.006374503	4.8107332494 4192	2.469231 5584378 2e-06	1.458279 7605350 7e-05	4.26 0103 6443 159	Up
STARK_HYPPOCAMPUS_22Q11_DELETION_DN	0.198038784673983	-0.006471448	4.8087 349394 4664	2.492119 5019330 8e-06	1.470051 3277064 9e-05	4.25 1332 6434 689	Up
ST_T_CELL_SIGNAL_TRANSDUCTION	0.156455223068166	0.0070049 647732251 4	4.7946 910608 3446	2.658847 8796850 8e-06	1.558401 2076681 1e-05	4.18 9777 9287 5182	Up
MATZUK_EARLY_ANTRAL_FOLLICLE	0.181889389601297	-0.003641508	4.7942 947514 9949	2.663705 6076654 6e-06	1.559775 5383754 3e-05	4.18 8043 1000 8691	Up
JIANG_AGING_HYPOTHALAMUS_DN	0.149235001836321	0.0038697 820046821	4.7937 159450 7422	2.670815 6678895 4e-06	1.560993 6770800 7e-05	4.18 5509 6152 2881	Up
KIM_WT1_TARGETS_UP	0.0971207293582947	0.0028778 783979405 6	4.7895 618754 5912	2.722384 2953678 8e-06	1.588142 7933598 2e-05	4.16 7334 4848	Up

						8129	
REACTOME_CELL_EXTRACELLULAR_MATRIX_INTERACTIONS	0.239037619456188	0.00449433356435902	4.78442358196407	2.78750110650208e-06	1.61550134155541e-05	4.14487154692551	Up
REACTOME_AMINO_ACID_TRANSPORT_ACROSS_THE_PLASMA_MEMBRANE	0.155342393601366	-0.003050379	4.78282092968071	2.80811690135617e-06	1.62593112002964e-05	4.13786944638285	Up
REACTOME_MECP2_REGULATES_TRANSCRIPTION_OF_NEURONAL_LIGANDS	0.229970900297731	0.00126321758228009	4.78054736829041	2.83761548882189e-06	1.6399515213331e-05	4.12793950018063	Up
REACTOME_VOLTAGE_GATED_POTASSIUM_CHANNELS	0.1836087892201	-0.003895454	4.7781382016058	2.86919965727415e-06	1.65358609774379e-05	4.1174216456304	Up
DAZARD_UV_RESPONSE_CLUSTER_G4	0.234655940251557	0.00889131418185331	4.76913825686336	2.99021577963557e-06	1.71536685251368e-05	4.07816968737367	Up
BIOCARTA_RACC_PATHWAY	0.224018980035594	-0.010217668	4.767675652	3.01034182265508e-06	1.7253177925411e-05	4.07179666406021	Up
GAL_LEUKEMIC_STEM_CELL_UP	0.0828928207631575	0.0008117	4.7664	3.026969	1.733247	4.06	Up

		21829175749	7434865136	91595907e-06	44173044e-05	656345017688	
BIOCARTA_ION_PATHWAY	0.288330606971056	0.00326570926790461	4.76490253019651	3.048860399375e-06	1.7441729492092 8e-05	4.0597178549429	Up
YAO_TEMPORAL_RESPONSE_TO_PROGESTERONE_CLUSTER_16	0.114783676929011	-0.006311592	4.76254002808336	3.0820500736395e-06	1.75991580561917e-05	4.04943226465209	Up
REACTOME_ATTENUATION_PHASE	0.157358894002105	-0.001136847	4.75881672973833	3.13506533386496e-06	1.78526151626604e-05	4.03323096527094	Up
WP_THYMIC_STROMAL_LYMPHOPOIETIN_TSLP_SIGNALING_PATHWAY	0.152972819011727	0.00461891040626705	4.75443993772055	3.19851043135924e-06	1.81972082928018e-05	4.01419982445227	Up
SCHEIDEREIT_IKK_TARGETS	0.212004284051778	0.00493163464505046	4.75346322098552	3.21283676688165e-06	1.82567498478478e-05	4.00995489553765	Up
NEWMAN_ERCC6_TARGETS_DN	0.196342558118297	-0.00407283	4.74961286726038	3.26991830673495e-06	1.85355095250263e-05	3.9932279930	Up

						177	
REACTOME_GAP_JUNCTION_ASSEMBLY	0.225303819669461	-0.003185843	4.74908292607871	3.27785084162865e-06	1.8546599976289e-05	3.99092669497489	Up
PID_RHODOPSIN_PATHWAY	0.183194604748324	-0.00437204	4.74533350394154	3.33450727841082e-06	1.88328359209244e-05	3.97465084645231	Up
KEGG_AXON_GUIDANCE	0.140057817108039	0.000368261631975516	4.74064450863073	3.40669104362157e-06	1.9181525123119e-05	3.95431174885539	Up
PUIFFE_INVASION_INHIBITED_BY_ASCITES_UP	0.126756575615426	0.00462202838999275	4.74029999782017	3.41205354418473e-06	1.91835293014082e-05	3.95281806304394	Up
HASLINGER_B_CLL_WITH_MUTATED_VH_GENES	0.195714184216387	0.00680702488557363	4.7378543492074	3.45035603643013e-06	1.93637973943235e-05	3.94221718017684	Up
REACTOME_REPRESSION_OF_WNT_TARGET_GENES	0.238376590535506	0.0141486648122859	4.73732656132668	3.45867619109847e-06	1.93929567462947e-05	3.93993004516392	Up
LEE_DIFFERENTIATING_T_LYMPHOCYTE	0.199820458494163	0.0092438	4.7337	3.515723	1.965954	3.92	Up

		9418710935	4038408932	78594859e-06	73327774e-05	439530023534	
REACTOME_PHASE_0_RAPID_DEPOLARISATION	0.160631851561079	-0.005453847	4.73256095966527	3.5346830650605 9e-06	1.9747774783826 3e-05	3.91928840404053	Up
REACTOME_REGULATION_OF_GENE_EXPRESSION_IN_BETA_CELLS	0.189303173560327	-0.007217225	4.73133898030958	3.5544303599214 3e-06	1.9840242125928 3e-05	3.91399838448978	Up
KIM_HYPOXIA	0.163880151220998	0.00577849154827813	4.72858763460372	3.5992823966116 1e-06	2.0004806751245 8e-05	3.90209189349707	Up
NAKAMURA_CANCER_MICROENVIRONMENT_UP	0.194513548256637	-0.005672078	4.72372036848869	3.6799674608828 6e-06	2.0430731690250 4e-05	3.88104312528676	Up
REACTOME_FORMATION_OF_SENESCENCE_ASSOCIATED_HETEROCHROMATIN_FOCI_SAHF_	0.182218690100167	0.00376775299709019	4.72246546246079	3.7010511596982 1e-06	2.0511093346648 9e-05	3.875619 1975877	Up
PID_HNF3A_PATHWAY	0.16705905581673	-0.004960575	4.72191361959558	3.7103594940012 e-06	2.0544336645196 6e-05	3.8732344217	Up

						0083	
WP_WNT_SIGNALING_PATHWAY_NETPATH	0.139369254165174	0.00457364092752915	4.72139827438844	3.71907256780101e-06	2.05742276544928e-05	3.87100758329436	Up
SCHEIDEREIT_IKK_INTERACTING_PROTEINS	0.140213084673634	-0.000255344	4.71815150906556	3.77442166698963e-06	2.0843269828296e-05	3.85698285743386	Up
REACTOME_SCAVENGING_BY_CLASS_A_RECEPTORS	0.217736765163277	0.00172531048401981	4.7175074280334	3.78549558229497e-06	2.08858409593821e-05	3.85420165943934	Up
WP_GPCRS_CLASS_B_SECRETINLIKE	0.224081373338425	-0.015629334	4.71549725492921	3.82025893688329e-06	2.10589229318247e-05	3.84552363126232	Up
PETRETTO_BLOOD_PRESSURE_UP	0.247431784989722	1.82611496844212e-05	4.70808484866545	3.95112526105768e-06	2.16840269632051e-05	3.81355102808874	Up
GUILLAUMOND_KLF10_TARGETS_UP	0.125820998713916	0.00123889624001876	4.7047488728108	4.01142180735697e-06	2.19304429169655e-05	3.79917561251982	Up
PID_ECADHERIN_KERATINOCYTE_PATHWAY	0.192468853305047	0.0102824	4.7037	4.029863	2.199943	3.79	Up

		342764638	3823695631	00621513e-06	68334013e-05	482228175606	
HOFMANN_MYELODYSPLASTIC_SYNDROM_RISK_UP	0.193358846994163	0.00463242403678903	4.70206891394461	4.0605023838797 6e-06	2.2147221701882e-05	3.78763338680236	Up
REACTOME_NCAM_SIGNALING_FOR_NEURITE_OUT_GROWTH	0.134065506595692	-0.001826879	4.70160155811748	4.0691205453661 2e-06	2.2174742076459 6e-05	3.78562112022925	Up
HOFFMANN_LARGE_TO_SMALL_PRE_BII_LYMPHOCYTE_DN	0.112883712954575	-0.001012123	4.69515192167328	4.1898683389862 3e-06	2.2732965716859 7e-05	3.75786867408418	Up
SIG_BCR_SIGNALING_PATHWAY	0.178911786454654	0.0115660842311247	4.6948183148605	4.1962070003141 1e-06	2.2747473232270 5e-05	3.75643406309601	Up
REACTOME_INTEGRIN_CELL_SURFACE_INTERACTIONS	0.152253303806277	-0.004443907	4.68977083670153	4.2932457976207 8e-06	2.3212697444104 7e-05	3.73473895363566	Up
LEIN_CEREBELLUM_MARKERS	0.129980888800702	-0.005300939	4.6865574386229	4.3561464141476 8e-06	2.3491399472297 7e-05	3.7209374496	Up

						8001	
BIOCARTA_ETS_PATHWAY	0.189501794488002	0.00903566300163938	4.6861920456862	4.3633547505291 4e-06	2.3503783033656 5e-05	3.71936860157843	Up
MINGUEZ_LIVER_CANCER_VASCULAR_INVASION_DN	0.164674052155021	-0.001312297	4.68605728873682	4.3660160847117 6e-06	2.3503783033656 5e-05	3.71879003653654	Up
NIKOLSKY_BREAST_CANCER_20P13_AMPLICON	0.241183036211237	-0.005359999	4.684331729	4.4002329188746 3e-06	2.3626510144857 1e-05	3.71138277528728	Up
OKAWA_NEUROBLASTOMA_1P36_31_DELETION	0.189904845892093	-0.001780299	4.68310089508954	4.4247973064020 1e-06	2.3717372090533 1e-05	3.70610063027803	Up
KOHOUTEK_CCNT1_TARGETS	0.135891088446761	-0.002024249	4.67883008110857	4.5110606325313 8e-06	2.4075798233983 e-05	3.68778152295018	Up
NAKAMURA_METASTASIS_MODEL_DN	0.139519824522749	0.000439814898168049	4.67720753358104	4.5442561102149 1e-06	2.4232128587718 2e-05	3.68082554572456	Up
WP_CIRCADIAN_RHYTHM_RELATED_GENES	0.0721401518277461	0.0016287	4.6746	4.597378	2.447335	3.66	Up

		6994278865	3460101751	23426454e-06	05146484e-05	979941380599	
LEE_METASTASIS_AND_ALTERNATIVE_SPLICING_UP	0.13120132415588	-0.003953821	4.67042337852256	4.68561830613591e-06	2.48578058343467e-05	3.65176365476581	Up
MAINA_HYPOXIA_VHL_TARGETS_UP	0.270210389822688	-0.000432802	4.66751371643448	4.74753644801045e-06	2.51303912352583e-05	3.63931029171493	Up
GUO_TARGETS_OF_IRS1_AND_IRS2	0.0889167711336361	0.00455697788961659	4.66603139247998	4.77938278124454e-06	2.52688491679599e-05	3.63296848414363	Up
KAAB_FAILED_HEART_ATRIUM_UP	0.177299639446246	-0.001105844	4.66397628763684	4.82387526292635e-06	2.54823776655182e-05	3.6241789964777	Up
MUELLER_COMMON_TARGETS_OF_AML_FUSIONS_UP	0.256162174595962	0.00915542341528712	4.66333879247318	4.83775772841716e-06	2.55339814798344e-05	3.6214531612948	Up
CREIGHTON_AKT1_SIGNALING_VIA_MTOR_DN	0.244989799188381	0.00241345518361587	4.66250366344254	4.85600220290691e-06	2.56085009969781e-05	3.6178827536	Up

						235	
PID_FRA_PATHWAY	0.144962145093291	-0.000998296	4.65947679763915	4.9226848473855 5e-06	2.5916119463716 8e-05	3.60494663505921	Up
WP_BREAST_CANCER_PATHWAY	0.0889604912985721	0.00160554100096333	4.65834495166632	4.9478454863966 8e-06	2.6004468191417 6e-05	3.60011122988915	Up
ZHONG_SECRETOME_OF_LUNG_CANCER_AND_ENDOTHELIUM	0.139943320931068	0.000303570713384691	4.65805875195192	4.9542272177102 7e-06	2.6015979983356 7e-05	3.59888870317943	Up
CHESLER_BRAIN_QTL_CIS	0.0886629149582676	0.00400062933618308	4.65713415528678	4.9748982403696 5e-06	2.6086740129948 7e-05	3.59493964680752	Up
DELLA_RESPONSE_TO_TSA_AND_BUTYRATE	0.180679327120116	0.0127278657549241	4.65100185289714	5.1141136519932 6e-06	2.6787597837909 e-05	3.56876482307387	Up
WP_TFS_REGULATE_MIRNAS_RELATED_TO_CARDIAC_HYPERTROPHY	0.288479670629372	0.0128736932285418	4.6501768938355	5.1331256735173 3e-06	2.6841879574997 5e-05	3.56524585353196	Up
WP_15Q133_COPY_NUMBER_VARIATION_SYNDROME	0.194778845174904	-0.0027531	4.6496	5.145840	2.688571	3.56	Up

		09	2681067826	69287301e-06	8165541e-05	289969875953	
YAMASHITA_LIVER_CANCER_WITH_EPCAM_DN	0.187606479134387	-0.00916144	4.64794054113822	5.18500756658156e-06	2.70448251813208e-05	3.55570908176954	Up
PID_CD8_TCR_PATHWAY	0.165461160985094	0.00241487453033502	4.64694711128069	5.20821607078007e-06	2.71295805638041e-05	3.55147392480021	Up
REACTOME_GLUCOSE_METABOLISM	0.138781871499391	0.00766579196136508	4.64687099601365	5.20999839407998e-06	2.71295805638041e-05	3.55114946461139	Up
KEGG_CELL_ADHESION_MOLECULES_CAMS	0.116323054298188	-0.002876403	4.64281912207073	5.3057297540967e-06	2.75817961337339e-05	3.53388389766108	Up
WP_TCA_CYCLE_NUTRIENT_UTILIZATION_AND_INVASIVENESS_OF_OVARIAN_CANCER	0.345167071359241	0.00177558229776166	4.63885435441617	5.40104081101819e-06	2.7960183748115e-05	3.51700195194841	Up
MEBARKI_HCC_PROGENITOR_WNT_DN	0.126059506199534	-0.005892933	4.62300772658178	5.798705411507e-06	2.98198545892493e-05	3.4496502204	Up

						9696	
WP_SYNAPTIC_VESICLE_PATHWAY	0.15395564896984	-0.005027369	4.61879125319827	5.90917283265512e-06	3.02875605056072e-05	3.4317624495923	Up
WP_MECP2_AND_ASSOCIATED_RETT_SYNDROME	0.100733646722776	0.004898383171381	4.61647699885941	5.9706618414031e-06	3.05283043194064e-05	3.42195049837291	Up
LEIN_MIDBRAIN_MARKERS	0.0991760531350755	-0.000132361	4.61426037758534	6.03013369540825e-06	3.07804604008216e-05	3.41255643509165	Up
ZHU_CMV_ALL_DN	0.132930007817436	0.000894690720351837	4.61342937370823	6.0525760500909e-06	3.08442853390101e-05	3.40903562709642	Up
CHIBA_RESPONSE_TO_TSA	0.161866897569662	0.0133697762857612	4.60928435218409	6.16572323197328e-06	3.13180393624044e-05	3.39148204993507	Up
REACTOME_CHONDROITIN_SULFATE_DERMATAN_SULFATE_METABOLISM	0.142761562280543	-0.005688916	4.60892039657813	6.17575478690866e-06	3.1343344204695e-05	3.38994139454174	Up
SMID_BREAST_CANCER_BASAL_DN	0.0684580974112366	-0.0017747	4.6073	6.218380	3.153389	3.38	Up

		87	8020273972	59272518e-06	57018343e-05	342277421238	
SETLUR_PROSTATE_CANCER_TMPRSS2_ERG_FUSION_UP	0.102852060564361	0.00801452349594846	4.60672921592897	6.2364820222474 8e-06	3.1579275058770 5e-05	3.38066813893671	Up
WP_GLOBO_SPHINGOLIPID_METABOLISM	0.167077852502549	0.000579077944544645	4.60650988006567	6.2425923146627e-06	3.1579275058770 5e-05	3.37974009946601	Up
NAKAYAMA_SOFT_TISSUE_TUMORS_PCA1_DN	0.162772657655192	-0.007253683	4.60456323575487	6.2970752062543 7e-06	3.1812293856939 5e-05	3.37150524381401	Up
HASLINGER_B_CLL_WITH_13Q14_DELETION	0.149838516035043	0.00269783121363786	4.60112590099276	6.3943986105683 8e-06	3.2242105748008 1e-05	3.35697162329645	Up
HOLLERN_EMT_BREAST_TUMOR_DN	0.127883111739299	-0.003218529	4.59197125377981	6.6607097565028 4e-06	3.3449049723797e-05	3.31830960900121	Up
WALLACE_PROSTATE_CANCER_UP	0.16433203012897	0.00543087728909396	4.58944914124219	6.7359330047116e-06	3.3799463347004 8e-05	3.3076697851	Up

						4832	
REACTOME_RORA_ACTIVATES_GENE_EXPRESSION	0.184843969474163	0.00692292638292336	4.58843184983711	6.7665048863008 1e-06	3.3925440896017 1e-05	3.30337964133122	Up
WP_TRYPTOPHAN_METABOLISM	0.139459948676057	-0.004848486	4.58618974000309	6.8343571747881 e-06	3.4210366922507 8e-05	3.29392704431179	Up
CROONQUIST_STROMAL_STIMULATION_DN	0.221868480234726	0.00484827594384307	4.58539389141268	6.8585986794596 1e-06	3.4304046739247 2e-05	3.29057274799299	Up
ST_B_CELL_ANTIGEN_RECEPTOR	0.187316994141033	0.0121303336640159	4.58375960423059	6.9086384911194 6e-06	3.4498728169250 6e-05	3.28368621410457	Up
MARIADASON_REGULATED_BY_HISTONE_ACETYLATION_UP	0.130108251873712	0.000703858627720544	4.58282775258994	6.9373275832655 2e-06	3.4614141727756 5e-05	3.27976053431975	Up
PID_INSULIN_GLUCOSE_PATHWAY	0.154367280839247	0.0097040227836505	4.57353201342626	7.2298601256106 7e-06	3.5958126442039 6e-05	3.24063716507785	Up
JOSEPH_RESPONSE_TO_SODIUM_BUTYRATE_DN	0.111591120091508	0.0005519	4.5671	7.438499	3.691869	3.21	Up

		851279809<br>65	205141<br>6049	9281797<br>6e-06	7026614<br>1e-05	3692<br>5334<br>4322	
REACTOME_ACTIVATED_PKN1_STIMULATES_TRANSCRIPTION_OF_AR_ANDROGEN_RECEPTOR_REGULATED_GENES_KLK2_AND_KLK3	0.20845915799964	0.0011171<br>629135942	4.5670<br>496078<br>9871	7.440839<br>3717245<br>4e-06	3.691869<br>7026614<br>1e-05	3.21<br>3394<br>7278<br>5664	Up
REACTOME_YAP1_AND_WWTR1_TAZ_STIMULATED_GENE_EXPRESSION	0.223870496523316	-0.0042746<br>57	4.5665<br>920896<br>0515	7.455951<br>5030775<br>6e-06	3.695740<br>1816987<br>8e-05	3.21<br>1473<br>2510<br>3858	Up
MIKKELSEN_NPC_ICP_WITH_H3K27ME3	0.257849630962441	0.0004790<br>435882140<br>71	4.5664<br>530931<br>1299	7.460548<br>4898800<br>9e-06	3.695740<br>1816987<br>8e-05	3.21<br>0889<br>5288<br>8004	Up
MIKI_COEXPRESSED_WITH_CYP19A1	0.27602712150982	-0.0020354<br>28	4.5651<br>966324<br>1435	7.502226<br>8412367<br>2e-06	3.713422<br>8073011<br>4e-05	3.20<br>5613<br>6564<br>4869	Up
REACTOME_GENERATION_OF_SECOND_MESSENGER_MOLECULES	0.174555806994366	0.0020294<br>788702197<br>4	4.5622<br>460119<br>7951	7.600984<br>4342312<br>5e-06	3.756314<br>5209612<br>6e-05	3.19<br>3228<br>9156<br>6973	Up
REACTOME_EXTRINSIC_PATHWAY_OF_FIBRIN_CLOT_FORMATION	0.301484542378341	-0.0196738<br>58	4.5604<br>370172<br>1928	7.662148<br>3812794<br>7e-06	3.780580<br>6876091<br>3e-05	3.18<br>5639<br>3591	Up

						7592	
ZHU_CMV_24_HR_DN	0.144722511950106	-0.00365885	4.56043345702473	7.66226922025501e-06	3.78058068760913e-05	3.18562442508735	Up
ITO_PTTG1_TARGETS_DN	0.287510821045777	0.00651128204673372	4.55315171770985	7.91329545814716e-06	3.90133637082759e-05	3.15510038092682	Up
WP_COMPUTATIONAL_MODEL_OF_AEROBIC_GLYCOLYSIS	0.265136903099372	0.0120316419595183	4.55011927691915	8.02014888749781e-06	3.95087810672214e-05	3.14240116739065	Up
REACTOME_BIOSYNTHESIS_OF_MARESINS	0.24929970476693	0.00150666672871135	4.54502909491361	8.20263504745749e-06	4.02480282526234e-05	3.12110091927692	Up
SERVITJA_ISLET_HNF1A_TARGETS_DN	0.13018108947943	-0.004241525	4.54423181550084	8.23157704016328e-06	4.03581348248764e-05	3.11776650006467	Up
WP_THYROID_HORMONES_PRODUCTION_AND_THEIR_PERIPHERAL_DOWNSTREAM_SIGNALLING_EFFECTS	0.106814716534036	-0.001775126	4.54337791685336	8.26268319506173e-06	4.04786697646e-05	3.11419584293362	Up
ROSS_AML_WITH_PML_RARA_FUSION	0.12342113199041	0.0052133	4.5428	8.282497	4.054374	3.11	Up

		1940399896	3558984287	91747481e-06	17774181e-05	192835130026	
WP_GPR40_PATHWAY	0.182112057151261	0.000542637886647975	4.54059757965352	8.3647510390242 6e-06	4.0881897400963 4e-05	3.10257359994828	Up
REACTOME_MATURATION_OF_SPIKE_PROTEIN	0.310641533213146	0.00880695629348806	4.53991788349765	8.3898867799855 6e-06	4.0948976338200 2e-05	3.09973329453329	Up
REACTOME_TYPE_I_HEMIDESMOSOME_ASSEMBLY	0.231771384852094	-0.009843868	4.53925823341702	8.4143505917180 6e-06	4.1027395226075 4e-05	3.09697710700359	Up
NAKAMURA_METASTASIS_MODEL_UP	0.157331406627593	0.00288763827815779	4.53762148325273	8.4753475788943 9e-06	4.1292372387910 1e-05	3.09013983173037	Up
REACTOME_PTK6_REGULATES_PROTEINS_INVOLVED_IN_RNA_PROCESSING	0.243810285730184	-0.00514574	4.53485908052679	8.5792592854186 1e-06	4.1733120991060 6e-05	3.07860512380446	Up
ST_G_ALPHA_I_PATHWAY	0.166573880273177	0.00487785990866617	4.52798762606133	8.8430755973655 8e-06	4.2882007994412 6e-05	3.0499388428	Up

						1599	
GILMORE_CORE_NFKB_PATHWAY	0.265047796399164	-0.0060756 32	4.5200 928234 3341	9.155804 6154588 3e-06	4.426018 6330337 2e-05	3.01 7049 5514 8701	Up
PID_HES_HEY_PATHWAY	0.151830265255138	0.0064866 736911817 5	4.5198 067392 263	9.167334 1315484 e-06	4.428143 4205852 8e-05	3.01 5858 6689 0801	Up
REACTOME_REGULATION_OF_KIT_SIGNALING	0.170619786300016	0.0099535 717337150 6	4.5091 080084 2601	9.608659 4003029 1e-06	4.622417 9248824 8e-05	2.97 1369 6799 4514	Up
REACTOME_SEMA3A_PLEXIN_REPULSION_SIGNALING _BY_INHIBITING_INTEGRIN_ADHESION	0.224773926706654	-0.0036805 74	4.5089 764246 9196	9.614212 2458889 6e-06	4.622417 9248824 8e-05	2.97 0823 0751 1053	Up
BERNARD_PPAPDC1B_TARGETS_DN	0.128871097932887	0.0048778 068982159	4.5064 475479 4931	9.721531 7830892 7e-06	4.666786 3710468 e-05	2.96 0320 6763 0718	Up
DIERICK_SEROTONIN_FUNCTION_GENES	0.255538584501888	-0.0090920 54	4.5061 454787 2921	9.734427 5404940 4e-06	4.669365 6679943 2e-05	2.95 9066 5254 4598	Up
SHEN_SMARCA2_TARGETS_DN	0.177376585380237	-0.0030148	4.5011	9.952113	4.744452	2.93	Up

		66	0359875513	33416588e-06	1862648e-05	814401502719	
MAHADEVAN_GIST_MORPHOLOGICAL_SWITCH	0.206667951979438	-0.003174994	4.4976088570964	1.0105735535499 4e-05	4.8029326545823e-05	2.92365358170466	Up
CHIANG_LIVER_CANCER_SUBCLASS_POLYSOMY7_UP	0.114295929797922	-0.00161298	4.49588654508115	1.0182280199578 1e-05	4.8319123240658 5e-05	2.91651583780057	Up
CERIBELLI_PROMOTERS_INACTIVE_AND_BOUND_BY_NFY	0.157254819819514	-0.001206292	4.49202682042808	1.0355844707054 4e-05	4.8992933000523 2e-05	2.90052861940779	Up
BIOCARTA_CD40_PATHWAY	0.193194502158263	0.00485562407175747	4.48800947920587	1.0539512438762 7e-05	4.9785961725571 1e-05	2.88390112409848	Up
WP_IL17_SIGNALING_PATHWAY	0.206124805439456	0.00308585571944093	4.4851223531798	1.0673435241291 9e-05	5.0333607713404 8e-05	2.87195943840845	Up
OHASHI_AURKA_TARGETS	0.313131207085737	-0.001346579	4.48397418967794	1.0727146919588 9e-05	5.0480212987026 9e-05	2.8672122654	Up

						917	
KEGG_VIRAL_MYOCARDITIS	0.135544049897882	0.0013792426696366 3	4.4823611609 4535	1.0803043742351 3e-05	5.0760403110351 6e-05	2.86 0544 8425 4097	Up
ASGHARZADEH_NEUROBLASTOMA_POOR_SURVIVAL_DN	0.110873204997571	0.0025155571492714 2	4.4794491119 8918	1.0941369782219 1e-05	5.1325763421581 1e-05	2.84 8513 1885 2512	Up
REACTOME_ARACHIDONATE_PRODUCTION_FROM_DAG	0.282774709295952	-0.005074267	4.4780424709 0498	1.1008794464439 7e-05	5.1493283527337 8e-05	2.84 2703 8158 0277	Up
REACTOME_COPI_DEPENDENT_GOLGI_TO_ER_RETROGRADE_TRAFFIC	0.110888814200532	-0.002334673	4.4772647517 8622	1.1046243986381 6e-05	5.1629545499601 3e-05	2.83 9492 5425 0166	Up
LIAO_HAVE_SOX4_BINDING_SITES	0.126586364893883	0.0058669822717489 6	4.4690973682 1469	1.1446985509103 5e-05	5.3381997787382 1e-05	2.80 5797 7625 2575	Up
REACTOME_WNT5A_DEPENDENT_INTERNALIZATION_OF_FZD4	0.199893764856144	0.0035953328502145 6	4.4672917340 6225	1.1537445529537 3e-05	5.3723124082399 3e-05	2.79 8355 7394 1538	Up
MEISSNER_NPC_HCP_WITH_H3K4ME3_AND_H3K27ME3	0.132815030656848	-0.0056896	4.4648	1.165939	5.420963	2.78	Up

		21	7896495982	36292702e-05	01549666e-05	841543141558	
GARGALOVIC_RESPONSE_TO_OXIDIZED_PHOSPHOLIPIDS_PURPLE_DN	0.226413423195592	0.00465442489261149	4.46399734010119	1.1704261224389 2e-05	5.4377507050736 6e-05	2.78478440451542	Up
HOLLEMAN_PREDNISOLONE_RESISTANCE_ALL_UP	0.212054969462634	0.014573375955697	4.46241390963954	1.17852614175547e-05	5.4712877800121e-05	2.77826450351544	Up
MOREIRA_RESPONSE_TO_TSA_UP	0.20429110957931	0.00731855493513632	4.46146725774736	1.18339439231888e-05	5.4897825060712e-05	2.774367 5438695	Up
CERIBELLI_GENES_INACTIVE_AND_BOUND_BY_NFY	0.126723130963075	-0.002718186	4.4605704585561	1.18802406584811e-05	5.50714367193371e-05	2.77067646558286	Up
REACTOME_METABOLISM_OF_CARBOHYDRATES	0.0902118406279334	0.00158378287461663	4.45847405945781	1.19891450287751e-05	5.54933804575741e-05	2.76205053327405	Up
BIOCARTA_UCALPAIN_PATHWAY	0.244559077071356	0.0101403838696025	4.45481700457118	1.21814197594676e-05	5.6271994301789e-05	2.7470114530	Up

						9416	
PID_INTEGRIN2_PATHWAY	0.220974733300953	0.00151643320881013	4.45295516357882	1.2280441741824e-05	5.66726408115254e-05	2.73935901556677	Up
JACKSON_DNMT1_TARGETS_DN	0.164168041173187	-0.007038504	4.45277036050299	1.22903125155961e-05	5.6676054817463e-05	2.73859959894642	Up
BIOCARTA_BTG2_PATHWAY	0.178062879121902	0.00520429291191777	4.44648417911236	1.26306425093801e-05	5.81159362903796e-05	2.71278384823303	Up
REACTOME_DISEASES_OF_BASE_EXCISION_REPAIR	0.312576248814447	-0.014109581	4.44630038052115	1.26407280350126e-05	5.81192584543133e-05	2.71202950883916	Up
BIOCARTA_CELL2CELL_PATHWAY	0.255370749685977	0.00154942901205545	4.44590332290582	1.26625420385722e-05	5.81764607205164e-05	2.71040001172511	Up
BROWNE_HCMV_INFECTION_18HR_DN	0.117215585754105	-0.003123621	4.4430752009187	1.28189645843653e-05	5.88080659092056e-05	2.6987972364288	Up
BIOCARTA_IL4_PATHWAY	0.222677450074593	0.0149505	4.4416	1.289887	5.913096	2.69	Up

		00554987	4315460263	58855974e-05	20545812e-05	292449815946	
REACTOME_G_PROTEIN_MEDIATED_EVENTS	0.129156903671655	-0.000539681	4.44088843352375	1.29411830243325e-05	5.92363870343749e-05	2.68983009041349	Up
PID_CDC42_REG_PATHWAY	0.135123254258185	0.00875169988918606	4.43497193900494	1.32774846695012e-05	6.05090655973526e-05	2.66558783217475	Up
EBAUER_TARGETS_OF_PAX3_FOXO1_FUSION_UP	0.0968663920532037	-0.001252149	4.43329690377961	1.3374208305729e-05	6.09051437664417e-05	2.6587296214731	Up
VANHARANTA_UTERINE_FIBROID_UP	0.127774818073964	0.00025599965091138	4.43204712379653	1.34468157374558e-05	6.11908983008711e-05	2.65361402367223	Up
STARK_PREFRONTAL_CORTEX_22Q11_DELETION_UP	0.112574923597339	0.0042595344512608	4.42725466605542	1.37287591771073e-05	6.23368019109764e-05	2.63400912185453	Up
REACTOME_SLC_TRANSPORTER_DISORDERS	0.0859928966399464	-0.00323638	4.42698039407827	1.37450651159884e-05	6.23652186951317e-05	2.6328876907	Up

						1304	
BANDRES_RESPONSE_TO_CARMUSTIN_WITHOUT_MGMT_24HR_DN	0.200241728747034	0.0113269270675223	4.42656941768142	1.3769533136386 2e-05	6.2430600567968 9e-05	2.631207421	Up
WANG_TNF_TARGETS	0.183064092084984	-0.007633926	4.42510778339027	1.3856892339829 4e-05	6.2689308129242 7e-05	2.62523265125955	Up
REACTOME_ACTIVATION_OF_PUMA_AND_TRANSLOCATION_TO_MITOCHONDRIA	0.21922248287949	0.00797429187045448	4.42129190037873	1.4087472833949 5e-05	6.3593413731145 2e-05	2.609642401	Up
SHI_SPARC_TARGETS_DN	0.180499702880723	-0.003986517	4.41595136784166	1.4416368603709 2e-05	6.4889340045846 9e-05	2.58784258438422	Up
SCHLOSSER_MYC_AND_SERUM_RESPONSE_SYNERGY	0.206648891296494	0.00130400628459374	4.41091210711261	1.4733452322053 e-05	6.6172603880595 6e-05	2.56729349315408	Up
WP_WNT_SIGNALING_IN_KIDNEY_DISEASE	0.121643741899796	-0.001047746	4.40774001247277	1.4936466451081 9e-05	6.6939095495931 8e-05	2.55436876499369	Up
RIZKI_TUMOR_INVASIVENESS_2D_UP	0.108899085267899	0.00335950508991552	4.40593954454789	1.5052886622965 8e-05	6.7404445924380 2e-05	2.5470363302	Up

						4773	
REACTOME_REGULATION_OF_BETA_CELL_DEVELOPMENT	0.163225586540696	-0.0028609	4.40579883901635	1.5062021346401 7e-05	6.7404445924380 2e-05	2.54646341417466	Up
KAYO_CALORIE_RESTRICTION_MUSCLE_UP	0.129769382930099	0.000400297485855897	4.40186167113113	1.5319791005628 7e-05	6.8360850303333 7e-05	2.53043873432777	Up
BLANCO_MELO_COVID19_SARS_COV_2_LOW_MOI_INFECTION_A594_ACE2_EXPRESSING_CELLS_DN	0.203716281556601	-0.004681858	4.40122876691287	1.5361620285771 3e-05	6.8498259420820 6e-05	2.52786390855963	Up
WP_CANCER_IMMUNOTHERAPY_BY_PD1_BLOCKADE	0.192385527814083	-0.003932518	4.3980085283214	1.5576150216074 8e-05	6.9272715361475 9e-05	2.51476808223569	Up
REACTOME_REGULATION_OF_CYTOSKELETAL_REMODELING_AND_CELL_SPREADING_BY_IPP_COMPLEX_COMPONENTS	0.244478958032793	-0.000341634	4.39795196869782	1.5579943715904 7e-05	6.9272715361475 9e-05	2.5145381441133	Up
WP_MIR5093P_ALTERATION_OF_YAP1ECM_AXIS	0.207215557630026	-0.004055474	4.39677996963199	1.5658750217785 7e-05	6.957327315805e-05	2.50977406371463	Up
KIM_RESPONSE_TO_TSA_AND_DECITABINE_DN	0.198468807034981	-0.0053664	4.3960	1.570738	6.973946	2.50	Up

		25	5943164308	94738169e-05	09899724e-05	684568277408	
PID_EPHA_FWDPATHWAY	0.146051375829778	0.00309989937773879	4.39497996358313	1.5780529033360 3e-05	7.0014112730569 9e-05	2.50245933257187	Up
WIEDERSCHAIN_TARGETS_OF_BMI1_AND_PCGF2	0.133467634548927	-0.002926983	4.38930990354066	1.6170096289909 5e-05	7.1537981234118 4e-05	2.47943476094245	Up
REACTOME_CELL_CELL_COMMUNICATION	0.133037392899315	7.15931447607878e-05	4.38124145611543	1.6740369038235 9e-05	7.3850370021556 5e-05	2.44671549756554	Up
PID_A6B1_A6B4_INTEGRIN_PATHWAY	0.122028837050354	7.31853564173773e-05	4.37964727633644	1.6855301919051 2e-05	7.4304587366158 3e-05	2.44025694876067	Up
BIOCARTA_GATA3_PATHWAY	0.182178486183868	0.00141070886586685	4.37833672482341	1.6950352401252 8e-05	7.4632799596001 1e-05	2.434949 0014835	Up
GARGALOVIC_RESPONSE_TO_OXIDIZED_PHOSPHOLIPIDS_GREEN_DN	0.15817757764788	0.00614040516958611	4.37828929794682	1.6953801744862 5e-05	7.4632799596001 1e-05	2.4347569407	Up

						8481	
MASRI_RESISTANCE_TO_TAMOXIFEN_AND_AROMATASE_INHIBITORS_DN	0.166328380182595	0.00414123369085762	4.37429691802091	1.72465899763888e-05	7.57604982189986e-05	2.41859581291181	Up
REACTOME_INTEGRATION_OF_ENERGY_METABOLISM	0.119225548894705	-0.001540109	4.37107158083729	1.74866571921911e-05	7.65982224360834e-05	2.40554902842925	Up
KEGG_GLYCOLYSIS_GLUCONEOGENESIS	0.127076914896811	0.00271554185283447	4.37083212263409	1.75046075499806e-05	7.66227779003733e-05	2.40458073169528	Up
REACTOME_PTK6_EXPRESSION	0.2398383324687	-6.12E-05	4.36665289388996	1.78207494275909e-05	7.78967547162369e-05	2.38768861936682	Up
REACTOME_ASSEMBLY_AND_CELL_SURFACE_PRESENTATION_OF_NMDA_RECEPTORS	0.14595391581176	-0.003136167	4.36329722281042	1.80785439858618e-05	7.87463315931536e-05	2.37413543484668	Up
LOPEZ_MESOTHELIOMA_SURVIVAL_DN	0.176639769199369	-0.002775237	4.36277880624771	1.81186877590943e-05	7.88658449654264e-05	2.37204241590403	Up
WP_ENDOTHELIN_PATHWAYS	0.151466507469484	-0.0056531	4.3605	1.829325	7.956989	2.36	Up

		58	3715134303	60044819e-05	48982615e-05	299460481871	
REACTOME_P75NTR_SIGNALS_VIA_NF_KB	0.184309915777702	0.0157863056253116	4.34911514990995	1.9208047666314e-05	8.2852225062412 1e-05	2.31695574688606	Up
WP_CEREBRAL_ORGANIC_ACIDURIAS_INCLUDING_DISEASES	0.274543071625463	0.00732973125036675	4.34793174895132	1.9305292501091 5e-05	8.3213854551579 7e-05	2.31219180206043	Up
REACTOME_BMAL1_CLOCK_NPAS2_ACTIVATES_CIRCADIAN_GENE_EXPRESSION	0.153546549710453	0.000256172391116514	4.34454473404974	1.9586229548732 6e-05	8.4307716233691 7e-05	2.29856314309558	Up
REACTOME_NRAGE_SIGNALS_DEATH_THROUGH_JNK	0.138187420487085	0.00754545257119179	4.34330035020422	1.9690425300691 2e-05	8.4697484297567 6e-05	2.29355831909286	Up
LEE_TARGETS_OF_PTCH1_AND_SUFU_DN	0.105677289732791	-0.000692797	4.34252450830886	1.9755657092785 2e-05	8.4919226852436 e-05	2.29043856974139	Up
WP_AFLATOXIN_B1_METABOLISM	0.260838825252412	-0.009164341	4.3398510792661	1.9982024122560 4e-05	8.5654988762936 5e-05	2.2796921233	Up

						4966	
RODRIGUES_NTN1_TARGETS_DN	0.110391849878379	0.0025304564468114 5	4.3368243591 1104	2.0241302433224 7e-05	8.6706531541080 4e-05	2.26 7532 5033 232	Up
PASTURAL_RIZ1_TARGETS_DN	0.280332254206947	-0.0044883 17	4.3353869926 9607	2.0365556716758 e-05	8.7178627959253 e-05	2.26 1760 5808 6334	Up
PID_LKB1_PATHWAY	0.146031047035404	-0.0002385 21	4.3350747128 952	2.0392648379338 9e-05	8.7234437278123 2e-05	2.26 0506 8032 025	Up
REACTOME_VEGFR2_MEDIATED_CELL_PROLIFERATIO N	0.134364507117175	0.0046101660565028 2	4.3331665260 3854	2.0558943081380 2e-05	8.7824748593342 6e-05	2.25 2847 3001 4526	Up
MOREIRA_RESPONSE_TO_TSA_DN	0.168502038428038	0.0048892944782739 6	4.3304837000 5338	2.0794943028759 e-05	8.8649870452958 2e-05	2.24 2083 3502 8083	Up
MEISSNER_BRAIN_HCP_WITH_H3K27ME3	0.148388805584574	-0.0074294 5	4.3286550080 6565	2.0957291271874 e-05	8.9219414900220 9e-05	2.23 4749 6604 9804	Up
REACTOME_DEX_H_BOX_HELICASES_ACTIVATE_TYP	0.246440352690132	-0.0053955	4.3284	2.097169	8.921952	2.23	Up

E_I_IFN_AND_INFLAMMATORY_CYTOKINES_PRODUCTION_		08	9345576929	1794756e-05	77382115e-05	410190951873	
WP_WNTBETACATENIN_SIGNALING_PATHWAY_IN_LEUKEMIA	0.13478318280517	0.00616341069030781	4.31177105941554	2.2514639993558e-05	9.5652546502405 6e-05	2.16716679480406	Up
ROVERSI_GLIOMA_COPY_NUMBER_UP	0.112585725210692	-0.004311654	4.31020620810905	2.2664458678873 8e-05	9.6198232621825 9e-05	2.16091469659796	Up
BIOCARTA_MRP_PATHWAY	0.245450210152782	-0.00347094	4.30908613408499	2.2772279671251 8e-05	9.6548866065204 7e-05	2.15644084700581	Up
REACTOME_PLASMA_LIPOPROTEIN_REMODELING	0.158788884098199	-0.008668475	4.30618467558976	2.3053868013707e-05	9.7676012806197 5e-05	2.14485643240804	Up
KAYO_AGING_MUSCLE_UP	0.0791202957505861	-0.000215239	4.2976584920979	2.3900747501979 7e-05	0.000100919686901217	2.11085407206232	Up
SMID_BREAST_CANCER_LUMINAL_B_UP	0.0875226035376849	-0.004752948	4.2965686326836	2.4011121171951 9e-05	0.000101316811090622	2.1065119631	Up

						9345	
WP_NUCLEAR_RECEPTORS	0.153339031768554	-0.006546408	4.29636640314635	2.40316551837431e-05	0.00010133456774829 7	2.10570636631727	Up
KAPOSI_LIVER_CANCER_MET_DN	0.262865948645405	0.00983863966503518	4.29451630613956	2.42202919914449e-05	0.00010206066014317 6	2.09833790078702	Up
WP_PATHWAYS_REGULATING_HIPPO_SIGNALING	0.110613865455836	0.00150713538471642	4.29340359987624	2.43344252673148e-05	0.00010247203367315	2.09390761026806	Up
WP_NCRNAS_INVOLVED_IN_STAT3_SIGNALING_IN_HEPATOCELLULAR_CARCINOMA	0.198348690405889	0.00625275254754701	4.29068856997464	2.46150762023274e-05	0.00010344331617322	2.08310180561847	Up
WP_HAIR_FOLLICLE_DEVELOPMENT_CYTODIFFERENTIATION_PART_3_OF_3	0.117543054426625	0.000658025269870986	4.28879445756207	2.48126998119821e-05	0.00010420326639578 7	2.07556678567166	Up
WP_ESC_PLURIPOTENCY_PATHWAYS	0.103260603963812	-0.00053495	4.2879391705044	2.49024332944006e-05	0.00010450940058035 5	2.072165 2980213	Up
HERNANDEZ_MITOTIC_ARREST_BY_DOCETAXEL_2_D	0.163148298159341	-0.0062876	4.2771	2.606049	0.000108	2.02	Up

N		07	5811312702	07127574e-05	928764312645	933975609314	
REACTOME_ACTIVATION_OF_RAC1_DOWNSTREAM_OF_NMDARS	0.253166577339414	0.00815890641361407	4.2771555458481	2.6060772515591 7e-05	0.000108928764312645	2.02932956933175	Up
NEBEN_AML_WITH_FLT3_OR_NRAS_UP	0.240123437041747	0.00662650055598438	4.27302577889605	2.65178772919371e-05	0.000110764780855352	2.01294990255766	Up
GHANDHI_DIRECT_IRRADIATION_DN	0.14336275429658	-8.88E-05	4.27271686437977	2.65523760237852e-05	0.000110834295884085	2.01172522840484	Up
REACTOME_NR1H2_NR1H3_REGULATE_GENE_EXPRESSION_TO_CONTROL_BILE_ACID_HOMEOSTASIS	0.286603692507938	0.00592310197966847	4.27082967494852	2.6764065842756e-05	0.000111567868828735	2.00424525471494	Up
LEIN_ASTROCYTE_MARKERS	0.146062456007625	-0.001046024	4.26927851606395	2.69392696730293e-05	0.000112222850241942	1.9980993206831	Up
REACTOME_SEALING_OF_THE_NUCLEAR_ENVELOPE_NE_BY_ESCRT_III	0.191903153382655	0.00334316971269619	4.26861092400811	2.70150114149335e-05	0.00011246289460261	1.9954548179	Up

						4434	
NABA_BASEMENT_MEMBRANES	0.145541189250694	-0.005464225	4.26578940881637	2.73373811929346e-05	0.000113728636102242	1.98428207268229	Up
RAMASWAMY_METASTASIS_DN	0.116916425902371	0.00150459176320958	4.26025916502895	2.79799334472043e-05	0.00011632380904675	1.96240195876685	Up
HOFMANN_MYELODYSPLASTIC_SYNDROM_LOW_RISK_DN	0.157204577419799	0.00206065553703048	4.2598325005197	2.80301024188038e-05	0.000116454381334347	1.96071491604368	Up
REACTOME_HS_GAG_DEGRADATION	0.184409690427463	-0.004379322	4.25950203153181	2.80690194023985e-05	0.000116538062495443	1.95940833462358	Up
LIU_COMMON_CANCER_GENES	0.226731259767882	-0.000130699	4.25772650229824	2.82789965942854e-05	0.000117331371564659	1.95238991106878	Up
INAMURA_LUNG_CANCER_SCC_DN	0.201527402998553	-0.009209984	4.25743880882405	2.83131607581259e-05	0.000117394648514153	1.95125293946133	Up
REACTOME_COPI_MEDIATED_ANTEROGRADE_TRANS	0.104923042505655	0.0035615	4.2564	2.843611	0.000117	1.94	Up

PORT		5197674187	0612160165	9634034e-05	825764064385	717228866164	
SCIAN_INVERSED_TARGETS_OF_TP53_AND_TP73_DN	0.150776077141037	0.0097764209745414	4.25581214112728	2.8507074082876 1e-05	0.00011796227255 4941	1.94482557490173	Up
WP_METAPATHWAY_BIOTRANSFORMATION_PHASE_I_AND_II	0.092975464061925	-0.003953941	4.25516252219191	2.8584868598464 9e-05	0.00011812668401 5094	1.94225937164428	Up
BIOCARTA_RHO_PATHWAY	0.203359137904436	0.0110356571108586	4.24941060229309	2.9282592937572 5e-05	0.00012092951055 4566	1.91955241359068	Up
REACTOME_SCAVENGING_BY_CLASS_F_RECEPTORS	0.237481404596964	0.00424154399300455	4.24832520557146	2.9416067258327 8e-05	0.00012139995310 6676	1.91527059147585	Up
CHARAFE_BREAST_CANCER_LUMINAL_VS_MESENCHYMAL_UP	0.0817502102133828	-0.001767237	4.24309695634255	3.0067186361726 e-05	0.00012367596139 6444	1.894658905 0714	Up
LAMB_CCND1_TARGETS	0.159293015791068	0.00211107410363342	4.23828607202031	3.0678475160273 e-05	0.00012610681809 2592	1.8757122808	Up

						6732	
RICKMAN_METASTASIS_DN	0.159882627633753	0.00436587566082934	4.23804097500429	3.07099338535455e-05	0.000126152587312348	1.87474752392941	Up
BIOCARTA_CDMAC_PATHWAY	0.180242454652922	0.00949597736882164	4.23467435438741	3.11451777343522e-05	0.000127687000130201	1.8615006999468	Up
NIKOLSKY_BREAST_CANCER_22Q13_AMPLICON	0.185372161109009	-0.004685279	4.22561088881166	3.23464040026595e-05	0.000132088243187176	1.82588408619782	Up
GRESHOCK_CANCER_COPY_NUMBER_UP	0.0754511291264395	0.00427956646500279	4.22411651363509	3.25486628650043e-05	0.000132739520632774	1.82001808429884	Up
BIOCARTA_GPCR_PATHWAY	0.149269277946003	0.00796511899018237	4.2237651125497	3.25963989908611e-05	0.000132846913024475	1.81863896323032	Up
REACTOME_NTRK2_ACTIVATES_RAC1	0.250148104670782	0.00474702618838269	4.22007445831814	3.31018123295926e-05	0.000134641513191206	1.80416057436755	Up
BENPORATH_ES_CORE_NINE	0.207345339390749	-0.0050026	4.2178	3.341324	0.000135	1.79	Up

		82	2700296025	54055586e-05	819262758547	534927866674	
PID_TCR_PATHWAY	0.14070491953823	0.00332073742820232	4.21765367050302	3.34373802057559e-05	0.000135828415534769	1.79466988858709	Up
BEGUM_TARGETS_OF_PAX3_FOXO1_FUSION_DN	0.132034084793018	-0.006208571	4.2128839644543	3.41080838132892e-05	0.000138281434506262	1.77598427287418	Up
DANG_MYC_TARGETS_DN	0.149465579579288	0.00819643904067556	4.19767852809431	3.63330491067854e-05	0.000146536215598322	1.71654003756569	Up
FIGUEROA_AML_METHYLATION_CLUSTER_6_DN	0.128171447615336	-0.00775421	4.19316430906917	3.70198383696626e-05	0.000149209179714608	1.69892848678644	Up
MIKKELSEN_IPS_HCP_WITH_H3_UNMETHYLATED	0.137462929320992	-0.006597953	4.18962255597357	3.7567334452965e-05	0.000151219484403083	1.68512253114511	Up
REACTOME_NOTCH_HLH_TRANSCRIPTION_PATHWAY	0.168784308283397	0.0094600405967424	4.18648229748498	3.80592222425017e-05	0.000153100189539344	1.672890206	Up

MCCLUNG_DELTA_FOSB_TARGETS_8WK	0.157061955878361	0.00296203543351646	4.1825947312278	3.8676685499476 9e-05	0.000155282139000811	1.65775805102257	Up
GROSS_ELK3_TARGETS_UP	0.148763405346982	0.00279645325040842	4.17973814375884	3.9136478768485 5e-05	0.00015672266046 1929	1.64664681262517	Up
REACTOME_AGGREPHAGY	0.124583606001826	0.00186161147243132	4.17788501058812	3.9437541473204 4e-05	0.000157724755105786	1.63944227234971	Up
CUI_TCF21_TARGETS_2_UP	0.0914373419069139	-0.004885073	4.17636449852628	3.9686215301263 5e-05	0.000158617088457787	1.63353298376836	Up
FARMER_BREAST_CANCER_CLUSTER_4	0.163568708338029	-0.010434829	4.17471158335036	3.9958240167196 1e-05	0.000159396398918886	1.62711127406644	Up
REACTOME_GAP_JUNCTION_TRAFFICKING_AND_REGULATION	0.186250371664047	-0.001217305	4.17411360734559	4.0057088254217 6e-05	0.000159688084003808	1.62478863990684	Up
DIRMEIER_LMP1_RESPONSE_LATE_UP	0.130977805294892	0.0044684591816654	4.1712765710	4.0529250728249	0.0001613630912	1.613773	Up

		6	0294	6e-05	57374	1296<br>7458	
HATADA_METHYLATED_IN_LUNG_CANCER_UP	0.08402956781282	-0.0034824<br>58	4.1690<br>100401<br>2915	4.091027<br>5824134<br>3e-05	0.000162<br>7756936<br>15642	1.60<br>4977<br>4894<br>6656	Up
WANG_IMMORTALIZED_BY_HOXA9_AND_MEIS1_DN	0.136613661625821	0.0007900<br>897118480<br>99	4.1686<br>515294<br>4357	4.097085<br>6978647<br>7e-05	0.000162<br>9123057<br>44053	1.60<br>3586<br>6162<br>5134	Up
REACTOME_BIOSYNTHESIS_OF_MARESIN_LIKE_SPMS	0.254669492761161	-0.0050962<br>1	4.1664<br>216889<br>323	4.134958<br>1452289<br>2e-05	0.000164<br>2078388<br>19168	1.59<br>4938<br>1221<br>0753	Up
WU_HBX_TARGETS_3_DN	0.1901442593643	-0.0008489<br>36	4.1647<br>930265<br>5553	4.162830<br>7539249<br>6e-05	0.000165<br>2090184<br>75781	1.58<br>8623<br>8882<br>8276	Up
LASTOWSKA_NEUROBLASTOMA_COPY_NUMBER_UP	0.112916527981639	0.0015991<br>200526867<br>1	4.1617<br>925643<br>2303	4.214649<br>9677641<br>e-05	0.000167<br>0519307<br>14635	1.57<br>6996<br>9557<br>0884	Up
REACTOME_RECEPTOR_TYPE_TYROSINE_PROTEIN_PH<br>OSPHATASES	0.164756360530748	-0.0004112<br>23	4.1612<br>901226<br>4607	4.223387<br>2638657<br>2e-05	0.000167<br>2914151<br>04113	1.57<br>5050<br>6920<br>6517	Up

WP_HISTONE_MODIFICATIONS	0.164797242304893	0.0085536511176081 2	4.1591612585337	4.2605995677346 6e-05	0.0001685502964 7501	1.56680659733822	Up
MIKKELSEN_MCV6_HCP_WITH_H3K27ME3	0.141062294163872	-0.0062129 49	4.15702057875242	4.2983336242484 7e-05	0.0001699347567 24269	1.55852049416803	Up
FARMER_BREAST_CANCER_CLUSTER_7	0.146880237954768	0.0025469816513221 2	4.15654702100437	4.3067240398062 4e-05	0.0001701580911 20798	1.55668796347467	Up
WP_TCELL_ANTIGEN_RECEPTOR_TCR_SIGNALING_PATHWAY	0.119561130037658	0.0065195240008353 3	4.1547885351638	4.3380174974439 7e-05	0.0001709960305 77393	1.54988474499092	Up
BONOME_OVARIAN_CANCER_SURVIVAL_SUBOPTIMAL_DEBULKING	0.0455586562865339	0.0015627475504647 6	4.15464085518421	4.3406554114333 6e-05	0.0001709960305 77393	1.54931351713592	Up
PID_P38_MK2_PATHWAY	0.175609390548643	0.0063732296527171 3	4.15344358082472	4.3620981145582 7e-05	0.0001716901902 16	1.54468310682849	Up
REACTOME_TRANSCRIPTIONAL_REGULATION_BY_THE_AP_2_TFAP2_FAMILY_OF_TRANSCRIPTION_FACTOR	0.120226706306221	0.0059897113545731	4.1525702126	4.3778034703625	0.0001721991517	1.541306	Up

S		8	5279	8e-05	14452	1326 3472	
REACTOME_DISEASES_OF_METABOLISM	0.0804969259951085	-0.0022775 93	4.1498 532271 3138	4.427007 2554844 8e-05	0.000173 9145380 47589	1.53 0804 6119 701	Up
RAMPON_ENRICHED_LEARNING_ENVIRONMENT_LAT E_UP	0.156121481362291	-0.0050784 68	4.1498 526698 5005	4.427017 4015658 2e-05	0.000173 9145380 47589	1.53 0802 4586 231	Up
WP_OXYSTEROLS_DERIVED_FROM_CHOLESTEROL	0.168310383955266	-0.0124663 74	4.1465 775343 408	4.487030 3619038 3e-05	0.000175 9380761 61321	1.51 8151 6682 8914	Up
WP_MFAP5_EFFECT_ON_PERMEABILITY_AND_MOTILI TY_OF_ENDOTHELIAL_CELLS_VIA_CYTOSKELETON_R EARRANGEMENT	0.16798058036278	-0.0008355 88	4.1418 553068 6743	4.574926 7490359 2e-05	0.000179 1581724 37009	1.49 9926 7345 8113	Up
DACOSTA_UV_RESPONSE_VIA_ERCC3_TTD_UP	0.148734626798775	-0.0032612 07	4.1408 590021 3433	4.593679 8597199 9e-05	0.000179 7791354 93581	1.49 6083 9424 4837	Up
RADMACHER_AML_PROGNOSIS	0.109749008386757	0.0016827 499292857 8	4.1390 722833 2072	4.627494 385775e-05	0.000180 9883910 05075	1.48 9194 5302 4214	Up

LIM_MAMMARY_LUMINAL_MATURE_UP	0.107321203932503	-0.005451059	4.13888649966162	4.6310240212563 3e-05	0.000181012380981978	1.48847831724984	Up
MEBARKI_HCC_PROGENITOR_WNT_DN_BLOCKED_BY_FZD8CRD	0.115829032396633	-0.002252511	4.132342655	4.7569983902470 5e-05	0.000185469152061956	1.46326929198872	Up
FERRANDO_TAL1_NEIGHBORS	0.134913132273166	0.00102546651492886	4.13172190183682	4.7691165685323 4e-05	0.000185824899817202	1.46087977768659	Up
REACTOME_TANDEM_PORE_DOMAIN_POTASSIUM_CHANNELS	0.222792129112196	-0.00424743	4.12228832560271	4.9569372057227 e-05	0.000192900998344331	1.42460534631208	Up
CHARAFE_BREAST_CANCER_BASAL_VS_MESENCHYMAL_DN	0.100871211189611	0.000146207967510181	4.12103687728166	4.9823766147666 4e-05	0.000193769496540455	1.41979871871103	Up
PID_NCADHERIN_PATHWAY	0.150980830400569	0.00626812296134712	4.12078408402297	4.9875304723717 2e-05	0.000193848476155362	1.41882793372453	Up
LOPEZ_MESOTHELIOMA_SURVIVAL_WORST_VS_BEST_UP	0.171964940354745	-0.001109631	4.1177926795	5.0489052073386	0.0001959987437	1.407344	Up

			0415	3e-05	42398	2375 0209	
REACTOME_IKBA_VARIANT_LEADS_TO_EDA_ID	0.240129346384194	-0.001250489	4.11777984144795	5.0491701505412 4e-05	0.000195998743742398	1.40729496939014	Up
DESERT_PERIPORTAL_HEPATOCELLULAR_CARCINOMA_SUBCLASS_UP	0.0796022406947088	-0.002886116	4.11603745988017	5.0852514324840 6e-05	0.000197275972758928	1.40060955788458	Up
PID_PTP1B_PATHWAY	0.162803106349084	0.00383586952479064	4.11509232195619	5.1049260195703 2e-05	0.000197915526567601	1.39698416713274	Up
REACTOME_PLASMA_LIPOPROTEIN_CLEARANCE	0.15427489407849	0.00162599211190698	4.1143884951502	5.1196244179395 8e-05	0.000198308264508838	1.39428488322403	Up
FRIDMAN_SENESCENCE_DN	0.178365584474708	-0.004562169	4.11430140758285	5.1214459160249 2e-05	0.000198308264508838	1.39395091738574	Up
BIOCARTA_CBL_PATHWAY	0.239410801004449	0.00820078153156614	4.11274522189053	5.1540989322696 e-05	0.000199323938147024	1.38798426593343	Up

WP_INTEGRINMEDIATED_CELL_ADHESION	0.134756339022849	0.00394681662156148	4.10986588886488	5.2150389331405 4e-05	0.000201303846164922	1.37694972671321	Up
MIKKELSEN_ES_HCP_WITH_H3_UNMETHYLATED	0.139334584874172	-0.005861638	4.10971422035202	5.2182678988135 8e-05	0.000201303846164922	1.37636867305471	Up
REACTOME_THE_ROLE_OF_NEF_IN_HIV_1_REPLICATION_AND_DISEASE_PATHOGENESIS	0.165591639976537	0.0126002427270281	4.10768184621469	5.2617205774711 1e-05	0.000202728116848933	1.36858432094044	Up
REACTOME_DEFECTS_IN_BIOTIN_BTN_METABOLISM	0.255685923386898	0.00483134371650679	4.1047243227133	5.3255694568999 6e-05	0.000205016481474229	1.3572625632922	Up
BOYLAN_MULTIPLE_MYELOMA_D_UP	0.0935842007704549	0.000455313762201844	4.10310465428691	5.3608479743928 6e-05	0.000205908312976835	1.35106533156261	Up
KLEIN_TARGETS_OF_BCR_ABL1_FUSION	0.137564651512468	0.00507453070718011	4.10196275996268	5.3858536246452 7e-05	0.00020674083765104	1.3466974753832	Up
WP_PRION_DISEASE_PATHWAY	0.165258593241793	0.0068817708412567	4.0942960022	5.556643053634e-	0.0002126792587	1.317399	Up

		7	6476	05	68974	2279 0782	
REACTOME_SYNTHESIS_OF_PIPS_AT_THE_ER_MEMBRANE	0.254335335290742	0.0007303923729433 89	4.0942596256 3497	5.5574655642866 8e-05	0.0002126792587 68974	1.31 7260 3314 3708	Up
REACTOME_INOSITOL_PHOSPHATE_METABOLISM	0.11054053712727	-0.0021248 99	4.0942497437 1455	5.5576890240579 3e-05	0.0002126792587 68974	1.31 7222 5996 0154	Up
LIM_MAMMARY_STEM_CELL_UP	0.0925980427952397	-0.0038759 42	4.0939403213 9656	5.5646903185833 e-05	0.0002128159754 00163	1.31 6041 1825 4874	Up
REACTOME_HEMOSTASIS	0.102249782683631	-0.0003088 39	4.0913914032 7019	5.6226851220133 9e-05	0.0002149015181 79416	1.30 6312 0672 5295	Up
KEGG_ECM_RECEPTOR_INTERACTION	0.139414414908125	-0.0059376 02	4.0910178983 9137	5.6312315734954 5e-05	0.0002150957192 41146	1.30 4886 8648 5457	Up
GU_PDEF_TARGETS_UP	0.0991218641941201	0.0009103077728485 88	4.0858910183 8757	5.7498012243247 5e-05	0.0002193547400 08505	1.28 5335 5930 6155	Up

HUMMERICH_SKIN_CANCER_PROGRESSION_DN	0.0956721352977406	0.000128873846204579	4.08563294623857	5.7558320843526 4e-05	0.00021944993702442 8	1.28435201275514	Up
VARELA_ZMPSTE24_TARGETS_UP	0.118715675144145	0.00253269876475029	4.08239891776305	5.8319202739149 5e-05	0.00022221442075009 3	1.27203094846226	Up
KEGG_GAP_JUNCTION	0.121652198133199	-0.000154447	4.08207702741233	5.8395456948690 3e-05	0.00022236846704326 4	1.27080507694344	Up
JIANG_AGING_CEREBRAL_CORTEX_DN	0.0937881983782533	0.00377551249520707	4.07940363370428	5.9032449199551 2e-05	0.00022451863491520 5	1.26062716132414	Up
REACTOME_KINESINS	0.122603005135131	-0.005964276	4.07894880753103	5.9141477478268 5e-05	0.00022479556075175 3	1.25889617364971	Up
REACTOME_DEFECTIVE_CHST14_CAUSES_EDS_MUSCULOCONTRACTURAL_TYPE	0.228534238918469	-0.008606286	4.07586370920741	5.9886095661656 8e-05	0.00022748653352013 7	1.24715934809741	Up
WP_PEPTIDE_GPCRS	0.129219430042497	0.0008385311393784	4.0733164725	6.0507614103255	0.0002297068873	1.237474	Up

		11	6295	2e-05	02082	6692969	
BIOCARTA_BCR_PATHWAY	0.156460427528948	0.0108827232790454	4.06766578394374	6.1908357753982 4e-05	0.000233880204856341	1.21600970659015	Up
KEGG_TYROSINE_METABOLISM	0.11113973099137	-0.003564577	4.06542754124664	6.2471682916627 1e-05	0.000235721419977814	1.20751471554708	Up
BIOCARTA_KREB_PATHWAY	0.250718988376324	0.00768000158928851	4.06449781550426	6.2707109317483 e-05	0.000236465994856389	1.20398726702261	Up
REACTOME_REGULATION_OF_PTEN_MRNA_TRANSLATION	0.208814882604705	0.00587915330636109	4.0623804865506	6.3246417434090 4e-05	0.000238134921203139	1.19595663186003	Up
GAUSSMANN_MLL_AF4_FUSION_TARGETS_D_UP	0.126343738308917	-0.001372718	4.06092213280937	6.3620441169902 8e-05	0.000239183572587272	1.19042752405126	Up
REACTOME_ANTIGEN_ACTIVATES_B_CELL_RECEPTOR_BCR_LEADING_TO_GENERATION_OF_SECOND_MESSENGERS	0.177642063377202	0.00554135814945195	4.0530949580582	6.5664111532843 3e-05	0.000246270175398404	1.16078215284259	Up

REACTOME_UNFOLDED_PROTEIN_RESPONSE_UPR_	0.11281572896593	0.0001885264763311 99	4.0524612677 1689	6.5832273051119 5e-05	0.000246602847814302	1.15838427400 52	Up
KEGG_PATHWAYS_IN_CANCER	0.088074793005041	0.0016235729032815 9	4.0517872967 8115	6.6011573400989 5e-05	0.000247125353498156	1.15583433859 971	Up
DACOSTA_LOW_DOSE_UV_RESPONSE_VIA_ERCC3_XPCS_DN	0.220618880593914	-0.0017442 12	4.0503151299 6209	6.6404839664043 4e-05	0.000248298096261878	1.15026577698 365	Up
KEGG_PRIMARY_IMMUNODEFICIENCY	0.167709145608992	0.0027862070886632 7	4.0449188206 9124	6.7865525387620 1e-05	0.000253302054167744	1.12986925377 647	Up
WP_NOTCH_SIGNALING	0.142307228477697	0.0078075857219629 9	4.0447064258 8318	6.7923637558538 5e-05	0.000253366597551592	1.12906695487 362	Up
WP_HEMATOPOIETIC_STEM_CELL_DIFFERENTIATION	0.148557491597543	0.0001405250654608 94	4.0425903682 8388	6.8505191404192 5e-05	0.000255382416243738	1.12107581256 624	Up
HOLLEMAN_DAUNORUBICIN_ALL_UP	0.183797110098138	0.0002944204317882	4.0421084038	6.863830987977e-	0.0002557250836	1.119256	Up

		51	1378	05	87715	2265 2843	
VERRECCHIA_RESPONSE_TO_TGFB1_C4	0.196569639898788	0.0086283163777771 5	4.0387303936 244	6.957824 5220312 6e-05	0.000259 0714865 52178	1.10 6508 4487 3766	Up
REACTOME_COLLAGEN_BIOSYNTHESIS_AND_MODIFYING_ENZYMES	0.14021107009928	-0.0042928 13	4.0371 349915 4582	7.002641 2426991 5e-05	0.000260 4277662 87799	1.10 0491 0831 3512	Up
REACTOME_NOTCH3_INTRACELLULAR_DOMAIN_REGULATES_TRANSCRIPTION	0.155776759889417	0.0090633 712265261 9	4.0230 503378 9907	7.410384 8141800 1e-05	0.000274 4406834 22526	1.04 7459 6883 2759	Up
PIONTEK_PKD1_TARGETS_UP	0.136182491943355	-0.0023077 74	4.0211 757877 4859	7.466324 5594641 7e-05	0.000276 3475047 14336	1.04 0414 0634 9524	Up
SCHWAB_TARGETS_OF_BMYB_POLYMORPHIC_VARIANTS_DN	0.159500479170546	0.0061659 946324145 1	4.0179 595133 8546	7.563240 1937335 4e-05	0.000279 4347135 86334	1.02 8332 2736 9642	Up
REACTOME_ANTI_INFLAMMATORY_RESPONSE_FAVOURING_LEISHMANIA_PARASITE_INFECTION	0.11174669520737	-0.0024636 49	4.0161 948161 5375	7.616922 5463347 5e-05	0.000281 2506736 76977	1.02 1706 9177 2049	Up

REACTOME_NON_INTEGRIN_MEMBRANE_ECM_INTERACTIONS	0.144561801243165	-0.008633854	4.01503129664505	7.6525149216337 7e-05	0.000282396909147329	1.01734003035083	Up
PID_ILK_PATHWAY	0.141531327634336	0.00939496734256709	4.01281901406038	7.7206257990329 4e-05	0.00028474108339 0359	1.00904005802868	Up
BIOCARTA_WNT_PATHWAY	0.142096539552175	0.012320581961467	4.01181940575749	7.7515900047054 5e-05	0.000285713296669874	1.00529109269807	Up
LIU_PROSTATE_CANCER_DN	0.0756957867907224	-0.001841435	4.00811200220101	7.8674657483454 2e-05	0.000289812224925698	0.991393969554871	Up
SCHLESINGER_METHYLATED_DE_NOVO_IN_CANCER	0.105822103150068	-0.003340382	4.00628975373877	7.9250222369625 e-05	0.000291586324984151	0.984567497459373	Up
REACTOME_FORMATION_OF_TUBULIN_FOLDING_INTERMEDIATES_BY_CCT_TRIC	0.157478002769936	-0.005976082	4.00314621363236	8.0252541141082 7e-05	0.000294924525081528	0.97279771669875	Up

						4	
NUNODA_RESPONSE_TO_DASATINIB_IMATINIB_DN	0.174733230455616	0.00342627506476822	3.99821055170519	8.1850623656429 6e-05	0.000300619420731041	0.954334602848273	Up
REACTOME_INTERLEUKIN_21_SIGNALING	0.220320606511181	0.00610608909107472	3.99765438576509	8.2032584644881 2e-05	0.000300931591543013	0.9522553912772	Up
WP_SIGNAL_TRANSDUCTION_OF_S1P_RECEPTOR	0.117232123132666	-0.001411558	3.99353369715071	8.3392775827630 5e-05	0.000305020011527462	0.936858323715803	Up
REACTOME_NEURONAL_SYSTEM	0.117499637235043	-0.002610536	3.98920141097247	8.4845907847724 9e-05	0.000309787382359311	0.920685854332945	Up
MIKKELSEN_NPC_HCP_WITH_H3K27ME3	0.135235340382794	-0.003887245	3.98672208896504	8.5688309876899 8e-05	0.000312679211878846	0.911437549078618	Up
REACTOME_CYTOSOLIC_IRON_SULFUR_CLUSTER_ASS	0.20863857966866	8.9146488	3.9863	8.582725	0.000313	0.90	Up

EMBLY		0270967e-05	1537799799	48938093e-05	002215702629	992093463087	
WP_NEPHROTIC_SYNDROME	0.123527183958812	-0.006750415	3.98578145434925	8.60099846346592e-05	0.00031313935187161	0.90793015656786 1	Up
BAELDE_DIABETIC_NEPHROPATHY_UP	0.120029100808181	-0.003169975	3.9833164414229	8.68584116783744e-05	0.00031602002420 1448	0.89874223222904 3	Up
REACTOME_GLYCOLYSIS	0.115201842765453	0.00876695892089184	3.98065551650812	8.77831897485807e-05	0.000319010690146043	0.88882975931399 5	Up
REACTOME_MUSCLE_CONTRACTION	0.11651170179162	-0.004051772	3.9794496116321	8.82053626753959e-05	0.000320169991886656	0.88433946695205 1	Up
BIOCARTA_RHODOPSIN_PATHWAY	0.221684475452676	0.00190085301839662	3.97847750245833	8.85470896789922e-05	0.000320847510588152	0.8807206154	Up

						32763	
REACTOME_RHO_GTPASES_ACTIVATE_IQGAPS	0.186474720359236	0.0012424828871943	3.97828699609421	8.8614205864826 6e-05	0.000320903369780034	0.88001151345366	Up
NIKOLSKY_BREAST_CANCER_16P13_AMPLICON	0.144205205010288	-0.009404979	3.97239960849051	9.0712337532265 3e-05	0.000327694965572843	0.85811241596979 7	Up
LEIN_LOCALIZED_TO_DISTAL_AND_PROXIMAL_DENDRITES	0.141594744865036	-0.007306247	3.97228510438873	9.0753608155263 e-05	0.000327694965572843	0.85768678574730 4	Up
REACTOME_PHASE_4_RESTING_MEMBRANE_POTENTIAL	0.185174374225264	-0.005120734	3.96975759527888	9.1669150025696 6e-05	0.000330616161655723	0.84829441049468	Up
WP_HEDGEHOG_SIGNALING_PATHWAY_NETPATH	0.16653587212949	0.000192950307223206	3.96676929108469	9.2762921489328 9e-05	0.000334172637077344	0.83719656595572 2	Up
RICKMAN_TUMOR_DIFFERENTIATED_WELL_VS_MOD	0.0974082464924764	-0.0010617	3.9662	9.296571	0.000334	0.83	Up

ERATELY_DN		21	1891154009	66378632e-05	514900389111	5153400661617	
PID_CMYB_PATHWAY	0.0981886942599994	0.00917269816141322	3.96147200825587	9.4732337858468e-05	0.000340476908562542	0.817542035782952	Up
WP_FTO_OBESITY_VARIANT_MECHANISM	0.202840860951426	0.00236453761049014	3.95988887349869	9.53285760494233e-05	0.000342421569177529	0.81672668967762	Up
REACTOME_DISEASES_ASSOCIATED_WITH_GLYCOSAMINOGLYCAN_METABOLISM	0.131894115649053	-0.004523345	3.95954913638405	9.54569908847755e-05	0.00034268452424627	0.810413388821699	Up
YAGI_AML_WITH_T_8_21_TRANSLOCATION	0.0669652287344409	0.00330195228765216	3.95920041215448	9.5588973484975e-05	0.000342959975966034	0.809120896949347	Up
MIKKELSEN_NPC_HCP_WITH_H3K4ME3_AND_H3K27ME3	0.118795696581845	-0.004928705	3.9568074088	9.6499344189562	0.0003455901729	0.800254	Up

			3811	3e-05	21684	34234672 1	
HATADA_METHYLATED_IN_LUNG_CANCER_DN	0.155892389565624	-0.005088518	3.95668857614467	9.65447655624618e-05	0.000345590172921684	0.799814168103114	Up
DASU_IL6_SIGNALING_DN	0.222760077113716	0.00191362767805394	3.95362373770435	9.77232735668889e-05	0.000349204582055083	0.788465617021362	Up
WP_VITAMIN_DSENSITIVE_CALCIUM_SIGNALING_IN_DEPRESSION	0.135035616715134	0.00264931810574229	3.95248148427353	9.81659819048276e-05	0.000350451470789531	0.784238062330977	Up
REACTOME_HDACS_DEACETYLATE_HISTONES	0.13777572761691	0.00606504100012695	3.95228688895827	9.82415916181381e-05	0.000350451470789531	0.783517960963859	Up
BLANCO_MELO_BETA_INTERFERON_TREATED_BRONCHIAL_EPITHELIAL_CELLS_DN	0.121676482565922	-0.003400494	3.94847195644794	9.97350894005076e-05	0.000354963130681738	0.7694071637	Up

						89754	
REACTOME_ROLE_OF_ABL_IN_ROBO_SLIT_SIGNALING	0.189805833889362	0.0102986249909989	3.94765039165319	0.00010005952954202	0.000355913753505626	0.76636992409319	Up
KANNAN_TP53_TARGETS_DN	0.123429929045321	0.00664873306886743	3.94536992886906	0.000100965353125487	0.000358914885703672	0.757942245408693	Up
PID_INTEGRIN_CS_PATHWAY	0.138109989967296	-0.001469614	3.94389581056987	0.000101555023617234	0.000360407107828572	0.752496806073469	Up
PID_NFAT_TFPATHWAY	0.122375963219216	0.0060127756504909	3.941234218	0.000102627989062789	0.000363591283169368	0.742669400532315	Up
REACTOME_TRANSCRIPTIONAL_REGULATION_BY_VENTX	0.112554807602514	-0.003216955	3.93873812019608	0.00010364400654524	0.000366772148589684	0.733458430873211	Up

REACTOME_DEFECTIVE_B4GALT7_CAUSES_EDS_PROGEROID_TYPE	0.167574464724303	-0.010796346	3.93188111025282	0.00010648444939578	0.000376394634054447	0.708181869827227	Up
REACTOME_O_GLYCOSYLATION_OF_TSR_DOMAIN_CONTAINING_PROTEINS	0.164171245131108	-0.008363733	3.93113215116253	0.00010679913318233	0.000377292099978782	0.705423411941749	Up
WP_APOPTOSISRELATED_NETWORK_DUE_TO_ALTERED_NOTCH3_IN_OVARIAN_CANCER	0.100993246189237	0.0054293919495586	3.92351722172542	0.000110049116028656	0.000386863724369107	0.677403853659444	Up
KINSEY_TARGETS_OF_EWSR1_FLII_FUSION_DN	0.092588370002154	0.00189586685048938	3.92347015564825	0.000110069492063129	0.000386863724369107	0.67723082232564	Up
WP_CORTICOTROPINRELEASING_HORMONE_SIGNALING_PATHWAY	0.135329645536247	0.0072973410497043	3.92110789500453	0.000111096780710474	0.000389438904908843	0.668548710027329	Up
KEGG_GALACTOSE_METABOLISM	0.154007328882755	0.0075015128397351	3.9187695633	0.0001121226144	0.0003927455234	0.659959	Up

		5	3037	08797	96278	1469376	
KEGG_CYSTEINE_AND_METHIONINE_METABOLISM	0.119648322371309	0.00269736102879848	3.91849950481973	0.000112241666944486	0.000392940793414791	0.658967416428939	Up
REACTOME_SIGNALING_BY_NTRK3_TRKC_	0.124745099403964	-0.000358327	3.9173962065743	0.000112729291208931	0.000394425428711294	0.654916430750265	Up
OUELLET_CULTURED_OVARIAN_CANCER_INVASIVE_VS_LMP_DN	0.142559458275494	-0.0037807 12	3.91554654751235	0.00011355129448057	0.000396854101824831	0.648127315995280	Up
REACTOME_PHOSPHOLIPASE_C_MEDIATED_CASCADE_FGFR2	0.168024885417853	-0.008839737	3.91295236136462	0.000114713752707673	0.000400465839739329	0.638610267406052	Up
WP_STEROID_BIOSYNTHESIS	0.17540365988886	-0.00115528	3.9123944908758	0.000114965205200616	0.000401118059966398	0.636564396728152	Up

WP_SEROTONIN_RECEPTOR_467_AND_NR3C_SIGNALING	0.169143271545725	0.00687503399041943	3.9121891569922	0.00011505788815856 7	0.00040121590550574 3	0.635811444297235	Up
WP_EXRNA_MECHANISM_OF_ACTION_AND_BIOGENESIS	0.23001625199387	0.00846803026731012	3.90825832869552	0.00011684589257543 6	0.00040699352144541 8	0.621404042629655	Up
WP_MECHANOREGULATION_AND_PATHOLOGY_OF_YAPTAZ_VIA_HIPPO_AND_NONHIPPO_MECHANISMS	0.125492112660696	0.000273315845015832	3.90775927364376	0.00011707477190378 2	0.00040733358139393 2	0.619575816123664	Up
WP_HIPPOMERLIN_SIGNALING_DYSREGULATION	0.100306464259203	-0.000710187	3.90607892464789	0.00011784855178171 3	0.00040956660745189 8	0.613421600353764	Up
BERTUCCI_INVASIVE_CARCINOMA_DUCTAL_VS_LOBULAR_UP	0.124010648028156	-0.002068795	3.90482419385619	0.00011842949905293 8	0.00041135528854033 9	0.608827739327554	Up
BIOCARTA_EPO_PATHWAY	0.172220792131175	0.0067943	3.9033	0.000119	0.000413	0.60	Up

		5518258254	0488947168	136579690787	579837886306	3266979967199	
WP_MIRNA_REGULATION_OF_PROSTATE_CANCER_SIGNALING_PATHWAYS	0.126007053906045	0.0085551930153505	3.90214527568922	0.000119678952253113	0.00041523043970658	0.599024015767339	Up
KEGG_ALDOSTERONE_REGULATED_SODIUM_REABSORPTION	0.122487981485981	0.00382051871315911	3.89621992016352	0.000122487081678227	0.000424498780556537	0.577361067672561	Up
REACTOME_NETRIN_MEDIATED_REPULSION_SIGNALS	0.204566229909997	-0.008906601	3.89255751123472	0.000124253871084309	0.000429423038875449	0.563986126565958	Up
PID_NOTCH_PATHWAY	0.117538514770461	0.0101243157997388	3.89114935713517	0.000124939588186115	0.000431146064310782	0.558846612139159	Up
WP_EDA_SIGNALLING_IN_HAIR_FOLLICLE_DEVELOPMENT	0.163366867996082	0.0013544004400149	3.8911058867	0.0001249608135	0.0004311460643	0.558687	Up

		8	6745	48429	10782	979440609	
REACTOME_NRCAM_INTERACTIONS	0.198073796319757	-0.011929648	3.89008329561407	0.000125461102973928	0.000432631703421763	0.55495678252794 5	Up
MEISSNER_NPC_HCP_WITH_H3K4ME2_AND_H3K27ME3	0.132370916665876	-0.004135083	3.88879686310735	0.000126093169558358	0.000434087800027026	0.55026413693811 6	Up
MEISSNER_NPC_HCP_WITH_H3K4ME2	0.0967150204037798	-0.003574933	3.88407446282248	0.000128439407403693	0.000440942146988231	0.53304969035660 7	Up
REACTOME_P75_NTR_RECEPTOR_MEDIATED_SIGNALLING	0.107719081477782	0.00951200024875291	3.87987412160339	0.000130560974376663	0.000447598490685881	0.51775403342682 8	Up
KEGG_T_CELL_RECEPTOR_SIGNALING_PATHWAY	0.105522606969317	0.00848983883449894	3.87732838184284	0.000131862926713228	0.000451696018823956	0.5084908680	Up

						23697	
REACTOME_SYNAPTIC_ADHESION_LIKE_MOLECULES	0.16711219067187	-0.005620431	3.87602826283638	0.000132532574159198	0.000453739485827989	0.503762235284168	Up
MARTENS_TRETINOIN_RESPONSE_UP	0.133038670301955	-0.00482721	3.86763752386545	0.000136932466146718	0.000467770950672912	0.473278654611996	Up
BANDRES_RESPONSE_TO_CARMUSTIN_MGMT_24HR_DN	0.13857397685917	-0.003777278	3.86676885711337	0.000137395793615538	0.000468782077759282	0.47012616778946	Up
SANSOM_APC_TARGETS_REQUIRE_MYC	0.141480494133835	0.000397863505558064	3.86665799511047	0.000137455031661333	0.000468782077759282	0.469723883108363	Up
NGO_MALIGNANT_GLIOMA_1P_LOH	0.233824846024739	0.014252882063901	3.86548098472319	0.000138085449339598	0.000470490064965454	0.46545350597643	Up
MIKKELSEN_MEF_HCP_WITH_H3K27ME3	0.137069030744249	-0.0052653	3.8626	0.000139	0.000475	0.45	Up

		8	6335152922	605741804818	072828608829	5235428508331	
REACTOME_BETA_OXIDATION_OF_HEXANOYL_COA_TO_BUTANOYL_COA	0.274683515050609	0.0201978680269652	3.86166596682893	0.000140147682700863	0.000476655707684526	0.45162003834456 9	Up
NABA_ECM_GLYCOPROTEINS	0.128521553792555	-0.008188366	3.8610854839639	0.00014046401152649	0.000477439551175483	0.44951624866543	Up
PARK_OSTEOBLAST_DIFFERENTIATION_BY_PHENYLAMIL_UP	0.207766095227406	-0.00536063	3.86023341810094	0.000140929561858866	0.000478528331760385	0.44642869982240 6	Up
REACTOME_REGULATION_OF_COMMISSURAL_AXON_PATHFINDING_BY_SLIT_AND_ROBO	0.196140666275165	-0.010054261	3.85626670594886	0.000143116187485792	0.00048542195394771	0.43206295276553 4	Up
REACTOME_REGULATION_OF_SIGNALING_BY_CBL	0.159397210455962	0.0164042968610088	3.85456330380102	0.000144065002819185	0.000488373278262525	0.4258980192	Up

						81343	
TERAO_AOX4_TARGETS_SKIN_UP	0.100114854637683	0.0003586834842932 14	3.8521584820 1923	0.000145414660995316	0.000492410693288557	0.41719867569731 4	Up
REACTOME_OTHER_SEMAPHORIN_INTERACTIONS	0.154577169821019	0.0010400117860305 4	3.8510741455 5351	0.000146027130945309	0.000494215050042274	0.41327772650570 9	Up
KATSANOU_ELAVL1_TARGETS_DN	0.0617508408754756	0.0019608070470689 5	3.8462044961 6229	0.000148807908840967	0.000503077717960719	0.39568133824583 5	Up
WP_TYPE_2_PAPILLARY_RENAL_CELL_CARCINOMA	0.137365717881674	0.0046016751259809	3.8453604040 6522	0.00014929499017188	0.000504449648337976	0.39263325885936 1	Up
SANSOM_WNT_PATHWAY_REQUIRE_MYC	0.0800415192292322	0.0006868845153893 15	3.8443725032 5889	0.000149866972642384	0.000506106800430509	0.38906663811965	Up

						1	
WP_IL2_SIGNALING_PATHWAY	0.132237440049955	0.0103698943201513	3.84130182660649	0.000151658129334378	0.000511321025952462	0.37798581857703 6	Up
REACTOME_TFAP2_AP_2_FAMILY_REGULATES_TRANSCRIPTION_OF_CELL_CYCLE_FACTORS	0.226160705883798	-0.007521695	3.84078938964676	0.000151959004673365	0.000512057297506829	0.37613741648796 1	Up
JAEGER_METASTASIS_DN	0.10179989204128	0.000907810795130442	3.84063241846601	0.000152051282656721	0.00051209023952809	0.37557125287335 1	Up
FERREIRA_EWINGS_SARCOMA_UNSTABLE_VS_STABLE_DN	0.110783293744341	-0.002944214	3.84008546207681	0.0001523732341188	0.00051280782057306	0.37359865300677 9	Up
YUAN_ZNF143_PARTNERS	0.162105475834275	0.00129713139455579	3.83585524912002	0.000154885117749415	0.000520222903609642	0.35835089693719 9	Up

MEISSNER_BRAIN_HCP_WITH_H3K4ME2_AND_H3K27ME3	0.137006896352302	-0.007908922	3.8343522496023	0.000155786989900434	0.000522686403411889	0.352936985201889	Up
DEURIG_T_CELL_PROLYMPHOCYTIC_LEUKEMIA_UP	0.0668628382085988	0.000217525335194268	3.83109329282267	0.000157759619196668	0.000528447898733794	0.341204535296704	Up
BIOCARTA_TRKA_PATHWAY	0.149221544196811	0.00630517123638001	3.82971459908926	0.000158601229200602	0.00053063127550038	0.336243848131126	Up
KIM_WT1_TARGETS_8HR_UP	0.0825934901226965	0.00271229581737205	3.8278064883535	0.000159773026922934	0.000534039406629322	0.329380908191582	Up
REACTOME_ACYL_CHAIN_REMODELING_OF_DAG_AND_TAG	0.272979321980866	0.00780774738518958	3.8269172920256	0.000160321888523899	0.000535297451354406	0.326183767603846	Up
YAO_TEMPORAL_RESPONSE_TO_PROGESTERONE_CLU	0.149249939727217	-0.0082938	3.8264	0.000160	0.000535	0.32	Up

STER_4		41	8470365469	589550759332	902871808158	462862029306 6	
SMID_BREAST_CANCER_NORMAL_LIKE_UP	0.0647716405810468	0.00379024057536817	3.82088734495625	0.000164091240636611	0.000546413267506141	0.304520463854899	Up
LEE_INTRATHYMIC_T_PROGENITOR	0.144999908237764	0.00286126258897046	3.81889348765611	0.000165355945901853	0.000550034488860022	0.297364047841335	Up
KEGG_LONG_TERM_DEPRESSION	0.102573795272392	-0.005820578	3.81297575567972	0.000169164079370772	0.000560716806110403	0.27614370921712	Up
WP_ANDROGEN_RECEPTOR_SIGNALING_PATHWAY	0.103796102629112	0.00594501825271913	3.81292098940389	0.000169199706435441	0.000560716806110403	0.275947461523478	Up
PID_HDAC_CLASSIII_PATHWAY	0.135541709494313	0.00744050479751431	3.81257148986504	0.000169427233301442	0.000561171204430122	0.2746951359	Up

						25294	
RAMPON_ENRICHED_LEARNING_ENVIRONMENT_EARLY_UP	0.152245752365938	0.0167295560882069	3.81197243196814	0.000169817897733711	0.000562165168657678	0.27254883372782	Up
REACTOME_SEROTONIN_NEUROTRANSMITTER_RELEASE_CYCLE	0.172564015338812	0.00158934272820771	3.80436702210258	0.000174852265787073	0.000577492969837646	0.245326572418029	Up
BRUNEAU_SEPTATION_ATRIAL	0.245837895337526	-0.013353588	3.80399908050258	0.000175099366720549	0.000577492969837646	0.244010828631038	Up
TERAMOTO_OPN_TARGETS_CLUSTER_6	0.119678136396647	-0.003287283	3.80341867328202	0.000175489825875443	0.000578473366547464	0.241935549389448	Up
REACTOME_CROSSLINKING_OF_COLLAGEN_FIBRILS	0.17256188470018	-0.003049314	3.8025402888684	0.000176082310156463	0.000579810556573562	0.238795377443878	Up

REACTOME_SIGNAL_ATTENUATION	0.197407370688326	0.00896353872504119	3.79895338405179	0.000178521420822607	0.000587488107884844	0.225979179664304	Up
KEGG_GLYCOSPHINGOLIPID_BIOSYNTHESIS_GLOBO_SERIES	0.174459018557387	0.00285639558571238	3.79883392641971	0.000178603199545465	0.000587488107884844	0.225552538580584	Up
WP_MICRORNA_NETWORK_ASSOCIATED_WITH_CHRONIC_LYMPHOCYTIC_LEUKEMIA	0.215135150179033	0.0254314167651079	3.7982826381901	0.000178981061421634	0.000588419199281825	0.223583777614106	Up
KEGG_PROSTATE_CANCER	0.0966949368929579	0.00100203930581178	3.7959665179334	0.000180576840371872	0.000593351216616312	0.215315256530475	Up
CHIN_BREAST_CANCER_COPY_NUMBER_DN	0.191784159587362	0.00113603916967082	3.79233464319765	0.000183106265341695	0.00060039122502689	0.202358647993151	Up
REACTOME_SEMA4D_MEDIATED_INHIBITION_OF_CEL	0.221030252388228	0.0113316	3.7885	0.000185	0.000608	0.18	Up

L_ATTACHMENT_AND_MIGRATION		978422381	7339179797	761072337102	132371306115	8952260413248	
NOJIMA_SFRP2_TARGETS_UP	0.129003596267723	0.00824963137459732	3.78834829625706	0.000185921099858128	0.000608335406863152	0.18815032268098 5	Up
REACTOME_TRANSPORT_OF_INORGANIC_CATIONS_ANIONS_AND_AMINO_ACIDS_OLIGOPEPTIDES	0.100603108320555	-0.00653354	3.78765156692927	0.000186417252871329	0.000609637454463825	0.18566838843165 5	Up
WALLACE_JAK2_TARGETS_UP	0.193127214753779	-0.003508369	3.77914543243485	0.000192576388782358	0.000627857677363339	0.15540043546262 6	Up
REACTOME_PLATELET_AGGREGATION_PLUG_FORMATION_	0.173528711897442	-0.002945449	3.77911927687264	0.0001925956207024	0.000627857677363339	0.15530745888592 3	Up
SMID_BREAST_CANCER_ERBB2_UP	0.0948863900513173	0.0001309917911094	3.7771867669	0.0001940216065	0.0006321743370	0.148439	Up

			8783	93394	73594	4663 1453 5	
GAJATE_RESPONSE_TO_TRABECTEDIN_DN	0.156165344939868	-0.0062426 31	3.7749 827792 7295	0.000195 6600883 02748	0.000636 1771441 04327	0.14 0610 5254 1613	Up
REACTOME_KERATINIZATION	0.137295488852925	-0.0049769 04	3.7741 441124 4054	0.000196 2869939 96339	0.000637 8813464 5826	0.13 7632 5189 8319 1	Up
KUNINGER_IGF1_VS_PDGFB_TARGETS_UP	0.0974117076015865	-0.0023507 4	3.7731 258325 562	0.000197 0507116 5876	0.000640 0281356 70289	0.13 4017 5293 5397 7	Up
COLIN_PILOCYTIC_ASTROCYTOMA_VS_GLIOBLASTO MA_UP	0.146903227671548	0.0001833 775475276 26	3.7721 420079 6661	0.000197 7912531 33663	0.000642 0974415 27533	0.13 0525 6941 9029 1	Up
REACTOME_TRANSMISSION_ACROSS_CHEMICAL_SYN APSES	0.102758057111003	-0.0024028 46	3.7693 779530 5941	0.000199 8858982 19766	0.000647 8808199 73937	0.12 0719 7759 4209	Up

						5	
REACTOME_BINDING_AND_UPTAKE_OF_LIGANDS_BY_SCAVENGER_RECEPTORS	0.149136994072455	-0.004835794	3.76921951964609	0.000200006594304926	0.000647933679984695	0.120157904872349	Up
WP_VITAMIN_D_METABOLISM	0.181749125388343	0.00884581570741983	3.768945071	0.000200215834771501	0.000648273180191291	0.119184645947681	Up
SUMI_HNF4A_TARGETS	0.123887517633594	-0.008312478	3.7678361874271	0.00020106335845329	0.000650338856654285	0.115252935285294	Up
WP_CONSTITUTIVE_ANDROSTANE_RECEPTOR_PATHWAY	0.13479776614476	-0.002023951	3.76606834328009	0.000202421539004717	0.000653709933716066	0.10898693952126	Up
REACTOME_PKA_ACTIVATION_IN_GLUCAGON_SIGNALLING	0.158601153211222	-0.006159664	3.76232748736594	0.000205324144612782	0.00066152610674812	0.0957364866459169	Up
THEODOROU_MAMMARY_TUMORIGENESIS	0.124593466053235	0.0015436	3.7622	0.000205	0.000661	0.09	Up

		6256455293	6276628752	374707218242	52610674812	55073431655409	
BIOCARTA_ERK_PATHWAY	0.155564633374632	0.00432329815085801	3.76202668371829	0.000205559243731146	0.000661777088090883	0.09467152842070 36	Up
BIOCARTA_INTEGRIN_PATHWAY	0.168461295001869	0.00595537256043748	3.75812826576166	0.000208629218523291	0.000670964538535785	0.08087660629044 71	Up
LIN_MELANOMA_COPY_NUMBER_UP	0.0845006494414493	0.00211345085531962	3.75581351801402	0.000210472496862289	0.000676191919267198	0.07269175493664 02	Up
JAZAERI_BREAST_CANCER_BRCA1_VS_BRCA2_UP	0.133368784629954	0.0107435196641698	3.75279053636568	0.000212902916676088	0.000682939743570274	0.06200946749718 03	Up
REACTOME_INTERLEUKIN_3_INTERLEUKIN_5_AND_GM_CSF_SIGNALING	0.153128941693751	0.0145561911970448	3.7440199422	0.0002201051363	0.0007034977247	0.031060	Up

			9161	84347	87662	8093184932	
REACTOME_ASPARTATE_AND_ASPARAGINE_METABOLISM	0.170108562149758	-0.002695166	3.7413935555107	0.00022230621292578	0.00070943687175477 2	0.021805835165594 1	Up
BOYLAN_MULTIPLE_MYELOMA_D_CLUSTER_UP	0.115733297974698	-0.004933403	3.74014568445682	0.00022335928428 7418	0.00071243118066 3928	0.01741058821601 8	Up
BAFNA_MUC4_TARGETS_UP	0.246924834751196	-0.003556602	3.73902383699347	0.00022431001464 9195	0.00071472908671 8457	0.013460350986820 3	Up
WP_CANONICAL_NFKB_PATHWAY	0.20917219562269	0.00189885755389092	3.73729812831951	0.00022577995767 5282	0.00071867497296 9475	0.007385892921588 42	Up
REACTOME_ECM_PROTEOGLYCANS	0.142772703265392	-0.008452569	3.73653478948358	0.00022643305846 7216	0.00072038441512 3531	0.0046997640	Up

						3700034	
CHIN_BREAST_CANCER_COPY_NUMBER_UP	0.128238417070835	0.00591947537519224	3.73544880083947	0.000227365286394573	0.000722979678612251	0.000879109875056017	Up
WP_BENZENE_METABOLISM	0.21036618690895	-0.002010484	3.73268155959315	0.000229757124303012	0.000729837497722003	-0.008851883	Up
NIKOLSKY_BREAST_CANCER_17P11_AMPLICON	0.18197461570605	-0.014234464	3.72848490996095	0.000233429807802214	0.000740745816476656	-0.023596957	Up
KEGG_FOCAL_ADHESION	0.114258233078454	0.0011827731499634	3.72586254869078	0.000235752803029574	0.0007466467363926	-0.032803107	Up
MIKKELSEN_MCV6_ICP_WITH_H3K27ME3	0.121313376016403	-0.003675154	3.7237590263537	0.00023763193486406	0.000751391451707194	-0.040183573	Up
PID_PDGFRA_PATHWAY	0.14185694185228	0.006274174532298	3.71670829356017	0.000244034111968811	0.000770065954748555	-0.064894463	Up
REACTOME_EXTRACELLULAR_MATRIX_ORGANIZATION	0.122494507268266	-0.004524037	3.71462343631269	0.000245958140268388	0.000774955419617201	-0.072193209	Up
MEDINA_SMARCA4_TARGETS	0.120068207834254	0.0040202	3.7087	0.000251	0.000790	-0.0	Up

		9005391353	1410980213	489648304369	584421028363	92860676	
APPIERTO_RESPONSE_TO_FENRETINIDE_UP	0.0897654399267818	0.00179356903220255	3.70864463013577	0.000251555378045919	0.000790584421028363	-0.093103499	Up
REACTOME_MECP2_REGULATES_NEURONAL_RECEPTORS_AND_CHANNELS	0.137574961719113	0.00477413890187834	3.70667147353605	0.000253428816575591	0.000795844404472449	-0.099997737	Up
XU_GH1_EXOGENOUS_TARGETS_UP	0.148231425028114	-0.004059062	3.7066120063514	0.000253485482139847	0.000795844404472449	-0.100205464	Up
NIKOLSKY_OVERCONNECTED_IN_BREAST_CANCER	0.126845981981899	-0.002762028	3.7009142382832	0.000258970568098753	0.000811879511785149	-0.120094631	Up
BIOCARTA_BOTULIN_PATHWAY	0.251966622193742	-0.008812882	3.69791801209677	0.000261899653809125	0.000820187260945126	-0.130542435	Up
REACTOME_AMINE_LIGAND_BINDING_RECEPTORS	0.146281714639934	-0.005281564	3.69725018675693	0.000262556756588788	0.000821486189648006	-0.132870091	Up
MARTENS_BOUND_BY_PML_RARA_FUSION	0.123871544174457	0.00511750082190091	3.69722748575694	0.000262579120390147	0.000821486189648006	-0.132949207	Up
REACTOME_CREATINE_METABOLISM	0.219308531979987	-0.012158885	3.69411013912964	0.000265667272998974	0.00083031055564181	-0.143809393	Up

MIKKELSEN_IPS_ICP_WITH_H3K27ME3	0.121101059377148	-0.007762435	3.69124137802213	0.000268539385047799	0.000837599981402859	-0.153796233	Up
STEINER_ERYTHROCYTE_MEMBRANE_GENES	0.249142090481329	-0.003703858	3.68924315660331	0.000270557203447391	0.0008432362735342	-0.160748368	Up
WP_PRIMARY_FOCAL_SEGMENTAL_GLOMERULOSCLEROSIS_FSGS	0.103283660347794	0.00235569811827543	3.68537173877496	0.000274507312864801	0.000854925685374722	-0.174207957	Up
MIKKELSEN_NPC_WITH_LCP_H3K27ME3	0.208378193325508	0.004747906079382	3.68008829889531	0.000279985893586631	0.000871114005760511	-0.192556012	Up
MULLIGHAN_MLL_SIGNATURE_1_DN	0.0540203026087442	-0.000570967	3.6779951949596	0.000282184661334751	0.000876870971339911	-0.199818242	Up
REACTOME_SIGNALING_BY_GPCR	0.091554328576184	-0.00152425	3.67792369117524	0.000282260061339962	0.000876870971339911	-0.200066265	Up
TOMLINS_METASTASIS_UP	0.153160798849122	-0.005797228	3.67764815347041	0.000282550790536698	0.000877335045953618	-0.201021975	Up
SCIAN_CELL_CYCLE_TARGETS_OF_TP53_AND_TP73_UP	0.209629494782117	-0.002154821	3.67577576454986	0.000284533896077995	0.000883050946478058	-0.207514685	Up
REACTOME_DNA_METHYLATION	0.193650638494407	0.0055178156920684	3.6749087192	0.0002854566456	0.0008854719636	-0.21052	Up

		4	1244	12706	77194	0243	
REACTOME_TRANSPORT_OF_ORGANIC_ANIONS	0.1832434583495	-0.00914573	3.67409815761197	0.000286321831287221	0.000887268900049815	-0.213329423	Up
TSENG_IRS1_TARGETS_UP	0.0610625529443261	-0.001702863	3.66724952368414	0.000293731234921345	0.000909775336904584	-0.23704246	Up
WEST_ADRENOCORTICAL_TUMOR_MARKERS_DN	0.169298729510874	-0.004649349	3.66645041352812	0.000294607445484553	0.000912034121756917	-0.239806725	Up
REACTOME_UPTAKE_AND_FUNCTION_OF_DIPHTHERIA_TOXIN	0.193645949572822	0.0177355085384904	3.6656369741578	0.000295501895441258	0.000914347091228261	-0.242619996	Up
REACTOME_PKMTS_METHYLATE_HISTONE_LYSINES	0.136944886938809	0.00347155090707052	3.66489815042601	0.000296316514398232	0.000915954484496927	-0.24517472	Up
GROSS_HYPOXIA_VIA_ELK3_UP	0.0619973636665776	0.00141470254311451	3.66334938789438	0.000298031030791029	0.000920337615980057	-0.250528555	Up
REACTOME_TIGHT_JUNCTION_INTERACTIONS	0.141356325820905	-0.006261024	3.65998265203676	0.00030179036379933	0.000930623152270517	-0.26215977	Up
PID_S1P_S1P1_PATHWAY	0.141431324785572	0.00225983629612192	3.6590165049987	0.000302877390928334	0.000933445861714085	-0.265495772	Up
REACTOME_CHYLOMICRON_REMODELING	0.200908465311231	-0.0080684	3.6556	0.000306	0.000943	-0.2	Up

		61	5899056459	683716600414	770862141183	77082696	
REACTOME_CD28_DEPENDENT_PI3K_AKT_SIGNALING	0.134114771031847	0.00370684897473984	3.65393941090189	0.000308650553055788	0.000948882606645507	-0.283013303	Up
STEIN_ESRRA_TARGETS_DN	0.0842762881998654	0.00223795435788113	3.65244059107304	0.000310374566310954	0.000953238462687825	-0.288180473	Up
REACTOME_PHASE_2_PLATEAU_PHASE	0.159077630007582	-0.002234435	3.65213992361951	0.000310721498012521	0.000953832016896003	-0.289216791	Up
DASU_IL6_SIGNALING_UP	0.117785805002839	0.00233877889508327	3.65174161548688	0.000311181656666877	0.000954772388992243	-0.290589529	Up
KANG_CISPLATIN_RESISTANCE_UP	0.149299932312294	0.00616315370871104	3.65095092352704	0.000312097030985018	0.000957107841563244	-0.293314186	Up
BOSCO_EPITHELIAL_DIFFERENTIATION_MODULE	0.125811425985934	-0.006642759	3.64873037652588	0.00031468130336559	0.000964556469130973	-0.300963138	Up
REACTOME_CD28_CO_STIMULATION	0.108723131412902	0.00640706254328864	3.64618936503687	0.000317663239396233	0.000972256275607702	-0.309710791	Up
SOTIRIOU_BREAST_CANCER_GRADE_1_VS_3_DN	0.123148509123919	0.00664038874861242	3.64348417897778	0.000320867053719572	0.000979967680041881	-0.319017551	Up

WP_FOLATE_METABOLISM	0.0886252343419378	-0.002211589	3.64107445880058	0.000323746525088945	0.000987952153995616	-0.32730253	Up
KEGG_GLYCOSAMINOGLYCAN_BIOSYNTHESIS_CHONDROITIN_SULFATE	0.134466061599407	-0.000537671	3.63820496428663	0.000327207115759268	0.000997042006636119	-0.337161791	Up
STARK_HYPPOCAMPUS_22Q11_DELETION_UP	0.106433557238988	0.000719045101432399	3.63756160781319	0.000327987761318869	0.000998930340778322	-0.339371324	Up
WP_FOCAL_ADHESION	0.108091163670857	0.00122190809184407	3.63659503374822	0.000329163892265081	0.00100202073530621	-0.342690242	Up
REACTOME_CELL_CELL_JUNCTION_ORGANIZATION	0.131926512327505	-0.003456692	3.63616903331556	0.000329683510008013	0.001003110562685	-0.344152743	Up
KEGG_GLIOMA	0.104806834007548	0.00134418488795862	3.63563551729953	0.00033033535934131	0.00100460145783024	-0.345984135	Up
OLSSON_E2F3_TARGETS_UP	0.124338917680635	-0.001479912	3.63484422294713	0.00033130439529127	0.001007055035050 4	-0.34869995	Up
PID_TXA2PATHWAY	0.143473433757414	0.000560261723477266	3.63406809388663	0.000332257457024848	0.00100945767780383	-0.351363194	Up
NADELLA_PRKAR1A_TARGETS_DN	0.185713817196262	-0.005944222	3.6338978827	0.0003324668154	0.0010095995711	-0.35194	Up

			4789	52632	9104	7196	
KIM_GERMINAL_CENTER_T_HELPER_DN	0.185006747949949	0.0142689620446706	3.63340831028951	0.00033306967797 2998	0.00101044158904125	-0.3536268	Up
PID_INTEGRIN1_PATHWAY	0.112399050970306	-0.005558605	3.63013002889905	0.000337133150810162	0.00102177024759701	-0.364868491	Up
CHIARADONNA_NEOPLASTIC_TRANSFORMATION_KRAS_DN	0.0888681964870113	0.00216895270062462	3.62977231366781	0.000337579354508702	0.00102262325692314	-0.36609459	Up
MEISSNER_NPC_HCP_WITH_H3_UNMETHYLATED	0.101777012412853	-0.005526901	3.62916830967387	0.000338334032872574	0.00102390996686498	-0.368164615	Up
WP_TAMOXIFEN_METABOLISM	0.170395654226219	-0.001919859	3.6281027048647	0.000339669333728822	0.00102694961249625	-0.371815861	Up
BIOCARTA_NOS1_PATHWAY	0.126254326691331	5.92562624297867e-05	3.62671165956624	0.000341419901428076	0.0010302901643738	-0.376580749	Up
GAUTSCHI_SRC_SIGNALING	0.18845790895887	-0.003829735	3.62668984779108	0.000341447418014867	0.0010302901643738	-0.37665545	Up
REACTOME_HDMS_DEMETHYLATE_HISTONES	0.18124379594514	0.018030803190549	3.62656561598393	0.000341604182097838	0.0010302901643738	-0.377080911	Up
WP_ENVELOPE_PROTEINS_AND_THEIR_POTENTIAL_R	0.103309103448993	0.0028508	3.6198	0.000350	0.001054	-0.4	Up

OLES_IN_EDMD_PHYSIOPATHOLOGY		9422141208	032178463	240296656729	79937959647	0022034	
NABA_CORE_MATRISOME	0.121737852097878	-0.008494517	3.61861950893895	0.000351772942593557	0.00105838810212225	-0.404266694	Up
REACTOME_HCMV_INFECTION	0.100964221872906	0.00507916546794705	3.61807205054481	0.000352483913197671	0.00106001339593892	-0.406137703	Up
AIGNER_ZEB1_TARGETS	0.114153946900389	-0.002301966	3.61768748709849	0.000352984143883193	0.00106100367122662	-0.407451843	Up
BIOCARTA_MALATEX_PATHWAY	0.204582373038266	0.00480997047783506	3.61652663167593	0.000354498207967415	0.00106450523640113	-0.411417979	Up
REACTOME_METABOLISM_OF_FAT_SOLUBLE_VITAMINS	0.107721416749948	-0.005973661	3.61640012251793	0.000354663578037304	0.00106450523640113	-0.411850135	Up
REACTOME_GLUTAMATE_NEUROTRANSMITTER_RELEASE_CYCLE	0.123571158362903	-0.00312794	3.61614980590588	0.000354991000341104	0.00106497300102331	-0.412705179	Up
IVANOVA_HEMATOPOIESIS_STEM_CELL_LONG_TERM	0.0544811920043339	0.00178094084709197	3.61465327505707	0.00035695444815263	0.00107034601917071	-0.41781598	Up
REACTOME_FORMATION_OF_THE_CORNIFIED_ENVELOPE	0.129602952950042	-0.004998645	3.61303221491292	0.0003590927964639	0.00107571862338389	-0.423349892	Up

FLECHNER_BIOPSY_KIDNEY_TRANSPLANT_OK_VS_DONOR_DN	0.137858201561329	0.0125590821254915	3.61242705993387	0.000359894142012547	0.00107759910249488	-0.425415168	Up
MEBARKI_HCC_PROGENITOR_WNT_DN_CTNNB1_DEPENDENT_BLOCKED_BY_FZD8CRD	0.125978077489421	-0.001798172	3.61178211502999	0.000360750027864316	0.00107912068576087	-0.427615893	Up
LIU_CDX2_TARGETS_UP	0.149897601099336	-0.004724229	3.60617032833633	0.000368278435179677	0.00110005016706461	-0.446749736	Up
BIOCARTA_NUCLEARRS_PATHWAY	0.123662757264208	0.00143725727098335	3.60547384045481	0.000369223032598677	0.00110234120410774	-0.449122582	Up
REACTOME_G_ALPHA_I_SIGNALLING_EVENTS	0.0775516904196063	-0.0026585	3.60095472241886	0.000375407536901463	0.00111972829483296	-0.464508512	Up
MYLLYKANGAS_AMPLIFICATION_HOT_SPOT_1	0.285161377865377	-0.003857735	3.59521048001668	0.000383409214205457	0.00114194865286625	-0.484040203	Up
KEGG_LINOLEIC_ACID_METABOLISM	0.142772689870202	-0.010356699	3.59477659073264	0.000384020082595377	0.00114321949768322	-0.4855 1437	Up
REACTOME_DEADENYLATION_OF_MRNA	0.174915428502313	0.00859467235455516	3.5874287851862	0.000394504876333379	0.00117218370866505	-0.510454426	Up
REACTOME_COLLAGEN_FORMATION	0.121275694041488	-0.005537914	3.5805284794	0.0004045954489	0.0012004416593	-0.53383	Up

			0903	05058	4689	3277	
JOHNSTONE_PARVB_TARGETS_3_UP	0.0947322860549758	0.00452661617088701	3.57902869042123	0.000406820481495112	0.00120588465287083	-0.538909274	Up
YAGUE_PRETUMOR_DRUG_RESISTANCE_DN	0.1631244194324	0.000351282769618039	3.57625885749644	0.000410959945088333	0.00121641792044029	-0.548278611	Up
REACTOME_CLASS_C_3_METABOTROPIC_GLUTAMATE_PHEROMONE_RECEPTORS_	0.141374813420217	-7.87E-05	3.57448626609765	0.000413629770921782	0.00122315387713745	-0.554271175	Up
KUUSELO_PANCREATIC_CANCER_19Q13_AMPLIFICATION	0.107359302265488	-0.00354065	3.57280016969127	0.000416184427299766	0.0012295367635648	-0.559968817	Up
REACTOME_INTERLEUKIN_7_SIGNALING	0.121712614086915	0.00674810859653301	3.57114601490449	0.000418705078682862	0.001236395063456	-0.565556142	Up
KEGG_NEUROACTIVE_LIGAND_RECEPTOR_INTERACTION	0.120490872499542	-0.003422552	3.57048372065814	0.000419718315523671	0.00123879771015474	-0.567792547	Up
REACTOME_NCAM1_INTERACTIONS	0.127947870753604	-0.004617454	3.56948664704984	0.000421248070370074	0.00124272185018396	-0.571158707	Up
REACTOME_LYSINE_CATABOLISM	0.148084748084692	-0.010845334	3.5642653284838	0.000429344693439353	0.001264804229795	-0.588772098	Up
KEGG_BETA_ALANINE_METABOLISM	0.121995359663319	0.0072538	3.5597	0.000436	0.001281	-0.6	Up

		5096710259	4593709773	470566423312	60070726723	03998664	
KEGG_JAK_STAT_SIGNALING_PATHWAY	0.0828255273924143	0.000514506690389872	3.55973264579183	0.000436491686025927	0.00128160070726723	-0.604043419	Up
REACTOME_ACTIVATION_OF_THE_TFAP2_AP_2_FAMILY_OF_TRANSCRIPTION_FACTORS	0.164637676766169	0.00285301481678216	3.55952082692701	0.000436828391168787	0.00128186503022518	-0.604756636	Up
GRAESSMANN_RESPONSE_TO_MC_AND_DOXORUBICIN_UP	0.0664471725782811	0.00110731785152744	3.55941622923693	0.000436994748502735	0.00128186503022518	-0.605108814	Up
JINESH_BLEBBISHIELD_TO_IMMUNE_CELL_FUSION_PBSHMS_UP	0.0546001730374985	8.74962283240121e-05	3.55837208216851	0.000438658674171295	0.00128613811553199	-0.608623915	Up
REACTOME_SYNTHESIS_OF_PI	0.224813190055369	-0.010646384	3.55407092081167	0.000445575790257939	0.00130518590379001	-0.623093776	Up
MODY_HIPPOCAMPUS_NEONATAL	0.102588308397594	0.00047470192062369	3.55372780805886	0.000446131968824064	0.0013061986464 58	-0.624 24738	Up
REACTOME_CHYLOMICRON_ASSEMBLY	0.158094790476113	0.00193027723178037	3.55017599792289	0.000451927781164562	0.00132140834389814	-0.636183212	Up
REACTOME_N_GLYCAN_ANTENNAE_ELONGATION_IN_THE_MEDIAL_TRANS_GOLGI	0.101396002747013	0.00538397321141209	3.55015301777093	0.000451965508956943	0.00132140834389814	-0.636260401	Up

BIOCARTA_NEUTROPHIL_PATHWAY	0.23905128636333	0.00552529239590402	3.54971490976489	0.000452685340666561	0.00132288978790835	-0.637731897	Up
MYLLYKANGAS_AMPLIFICATION_HOT_SPOT_12	0.160785789650616	0.00501623911474143	3.54781810714701	0.00045581430305797	0.00133140676647387	-0.644100874	Up
CHESLER_BRAIN_QTL_TRANS	0.185435512027553	-0.005618614	3.54168025726418	0.000466078912833883	0.00136010898540663	-0.664688921	Up
TERAO_AOX4_TARGETS_HG_UP	0.159182476852207	0.00694237053283061	3.5332189713562	0.00048058495585889	0.00140112297840121	-0.693017083	Up
SCHLOSSER_MYC_TARGETS_AND_SERUM_RESPONSE_UP	0.181163595190046	0.0048806435352163	3.53201436673514	0.00048268423228108	0.0014065826430839	-0.697045038	Up
REACTOME_PHASE_I_FUNCTIONALIZATION_OF_COMPOUNDS	0.0989823086206972	-0.000843632	3.53061304116082	0.000485137155264536	0.00141306725608962	-0.701729212	Up
REACTOME_TRANSCRIPTIONAL_REGULATION_OF_PLURIPOTENT_STEM_CELLS	0.127520043471658	-0.003516444	3.52969350423672	0.000486753086756561	0.00141644463642662	-0.704802001	Up
WP_MYOMETRIAL_RELAXATION_AND_CONTRACTION_PATHWAYS	0.0818950354903168	-0.000808201	3.52894187436746	0.000488077696988647	0.00141963367629266	-0.707313157	Up
BARIS_THYROID_CANCER_DN	0.0811213970081656	0.0034636940479703	3.5280841918	0.0004895933341	0.0014233750935	-0.71017	Up

		7	4657	1227	9947	8033	
REACTOME_SLC_MEDIATED_TRANSMEMBRANE_TRANSPORT	0.0718022087854134	-0.005367901	3.52590865276054	0.000493457611773567	0.00143393791960605	-0.717442032	Up
REACTOME_INTEGRIN_SIGNALING	0.155907780206679	0.00189135528396979	3.52496057488852	0.000495150554640259	0.00143818413320173	-0.720606328	Up
BIOCARTA_ERAD_PATHWAY	0.150770643922876	0.0135387139849519	3.52472956485428	0.000495563883772594	0.00143871142496562	-0.721377228	Up
FIRESTEIN_PROLIFERATION	0.0629054951476697	0.00427147823479827	3.52174551489403	0.000500932180057151	0.00145158078506757	-0.7313311	Up
REACTOME_RECOGNITION_AND_ASSOCIATION_OF_DNA_GLYCOSYLASE_WITH_SITE_CONTAINING_AN_AFFECTED_PURINE	0.184741806533404	0.00403231408054444	3.52025242208659	0.000503638664899047	0.00145806212361399	-0.736308708	Up
NEBEN_AML_WITH_FLT3_OR_NRAS_DN	0.170158061954323	0.000757039087850208	3.51966821892006	0.000504701358423947	0.00146045749731349	-0.738255774	Up
REACTOME_PKA_MEDIATED_PHOSPHORYLATION_OF_CREB	0.127955367550825	-0.005954509	3.5185900274115	0.000506668161411463	0.00146546564672924	-0.741848458	Up
DURAND_STROMA_S_UP	0.0664708754575592	-0.00122282	3.5176036353866	0.000508473796929047	0.00147000319400959	-0.745134373	Up
NIKOLSKY_BREAST_CANCER_16Q24_AMPLICON	0.129313630491354	-0.0051640	3.5146	0.000513	0.001485	-0.7	Up

		68	4896561273	918610170194	05252017058	5497207	
REACTOME_CIRCADIAN_CLOCK	0.0879641125115871	0.00685080168228049	3.51177909052859	0.000519259435551101	0.00149978749021204	-0.764520208	Up
YAO_HOXA10_TARGETS_VIA_PROGESTERONE_DN	0.153603064766188	-0.004387254	3.51080988121087	0.000521074847574638	0.00150363160339181	-0.767743178	Up
WP_THERMOGENESIS	0.0753558688833998	0.00276373256126852	3.50424451511235	0.000533529812406658	0.00153742782990164	-0.789553982	Up
WP_SMALL_LIGAND_GPCRS	0.130038365875556	-0.004334828	3.50263733832872	0.000536620951417883	0.00154561774730896	-0.794887498	Up
YAUCH_HEDGEHOG_SIGNALING_PARACRINE_DN	0.0737179793116315	-0.004082745	3.50248296256451	0.000536918749183399	0.00154575819859989	-0.795399686	Up
SASSON_RESPONSE_TO_FORSKOLIN_DN	0.094776810568385	0.00612278202764831	3.50225832048174	0.000537352370382883	0.00154628936623392	-0.796144967	Up
CHEBOTAEV_GR_TARGETS_DN	0.0647111484898149	-0.000752529	3.50205157928547	0.000537751727792413	0.00154672148953082	-0.79683082	Up
REACTOME_PURINE_RIBONUCLEOSIDE_MONOPHOSPHATE_BIOSYNTHESIS	0.219560677126683	0.00449072287829502	3.49757082120733	0.000546475721356301	0.00156963202334964	-0.811686409	Up

MYLLYKANGAS_AMPLIFICATION_HOT_SPOT_17	0.128343745780635	0.00424955561713598	3.49345112981999	0.000554613523852505	0.00159006288339607	-0.825329563	Up
MIKKELSEN_MEF_ICP_WITH_H3K4ME3_AND_H3K27ME3	0.119342109868467	-0.008685768	3.49249470327639	0.000556518957496708	0.00159478909011176	-0.828494851	Up
KEGG_VASOPRESSIN_REGULATED_WATER_REABSORPTION	0.0836279061780593	-5.56E-05	3.48894929255179	0.000563635888490987	0.00161220643311685	-0.84022145	Up
KEGG_ASCORBATE_AND_ALDARATE_METABOLISM	0.131283941428136	-0.000456708	3.48718397830376	0.000567211212734417	0.00162093922534186	-0.84605624	Up
BIOCARTA_EPONFKB_PATHWAY	0.18402177877026	0.00327323297707887	3.48230736589902	0.000577198568314561	0.00164493641576147	-0.862160578	Up
WP_NUCLEAR_RECEPTORS_IN_LIPID_METABOLISM_AND_TOXICITY	0.11694429902386	0.00107442585705652	3.47739713635449	0.000587420918838641	0.00167253286386763	-0.878355092	Up
PID_INTEGRIN4_PATHWAY	0.150691371810819	-0.003991827	3.47707357771062	0.000588100434648579	0.00167369986146893	-0.879421492	Up
VERRECCHIA_RESPONSE_TO_TGFB1_C6	0.227806831803093	0.0108964909023824	3.47496148116819	0.000592554223418117	0.00168560222949416	-0.886380408	Up
LEIN_PONS_MARKERS	0.0792146082378572	-0.00041428	3.4737586893	0.0005951046435	0.0016920817784	-0.89034	Up

			2419	23502	4727	1625	
MEBARKI_HCC_PROGENITOR_WNT_UP_BLOCKED_BY_FZD8CRD	0.122062698460749	-0.004729973	3.47315248787848	0.000596393933324526	0.00169497121984676	-0.892337584	Up
KASLER_HDAC7_TARGETS_1_DN	0.142013809245402	0.00861107936476413	3.47290095906373	0.000596929660271237	0.00169497955699583	-0.893165666	Up
REACTOME_ADORA2B_MEDIATED_ANTI_INFLAMMATORY_CYTOKINES_PRODUCTION	0.0968281275051833	-0.004303356	3.47289469027045	0.000596943017817445	0.00169497955699583	-0.893186303	Up
KANG_IMMORTALIZED_BY_TERT_UP	0.0714211267315418	-0.001561564	3.47249827981755	0.000597788257630388	0.00169660343626512	-0.89449125	Up
REACTOME_PLASMA_LIPOPROTEIN_ASSEMBLY	0.125073467861612	-0.00424457	3.47222022857638	0.000598381796074849	0.00169751179535493	-0.895406488	Up
WP_PI3KAKT_SIGNALING_PATHWAY	0.065820397566912	-0.001486139	3.4694234013886	0.000604382756032501	0.00171296975648116	-0.904608836	Up
MILICIC_FAMILIAL_ADENOMATOUS_POLYPOSIS_UP	0.167357115725812	-0.010837531	3.46900043726028	0.000605295167977789	0.0017147727556541	-0.905999915	Up
REACTOME_MITOCHONDRIAL_UNCOUPLING	0.179225212402744	-0.003864881	3.46575715771956	0.000612334474242287	0.00173234279016494	-0.916661522	Up
MEBARKI_HCC_PROGENITOR_WNT_UP_CTNNB1_DEPE	0.122396276619748	-0.0060879	3.4608	0.000623	0.001759	-0.9	Up

NDENT_BLOCKED_BY_FZD8CRD		82	115545389	216140078588	91928183248	32901578	
REACTOME_BINDING_OF_TCF_LEF_CTNNB1_TO_TARGET_GENE_PROMOTERS	0.213261289302984	0.0129828486037765	3.45999039181205	0.000625040337126895	0.00176426801843867	-0.935596003	Up
REACTOME_OTHER_INTERLEUKIN_SIGNALING	0.148626726791163	-0.004245824	3.45601946928229	0.000633932351542876	0.0017869292034635	-0.948617252	Up
CASTELLANO_HRAS_TARGETS_UP	0.189676050869486	0.000113274506496949	3.45378699094597	0.00063898331464469	0.00179931042359115	-0.955931865	Up
REACTOME_SEROTONIN_RECEPTORS	0.17787189375686	-0.010615205	3.45369406401088	0.000639194374741178	0.00179931042359115	-0.956236242	Up
WP_LNCRNAMEDIATED_MECHANISMS_OF_THERAPEUTIC_RESISTANCE	0.181086312979467	-0.005646721	3.44978342678973	0.000648135731802486	0.00182117631837847	-0.96903851	Up
KEGG_LIMONENE_AND_PINENE_DEGRADATION	0.167171652836048	0.00727902740389025	3.44964457460333	0.000648455345938806	0.00182124992409148	-0.969492826	Up
BIOCARTA_CFTR_PATHWAY	0.134239847593825	-0.002907848	3.44936328756308	0.000649103272629465	0.0018222451439218	-0.97041313	Up
TAGHAVI_NEOPLASTIC_TRANSFORMATION	0.216589395669124	0.00626822622092718	3.44441378498299	0.000660603638182859	0.00185201751680262	-0.986595462	Up

MOOTHA_GLUCONEOGENESIS	0.115617869984436	-0.002949957	3.44290210303376	0.000664153888345247	0.00185861279754686	-0.991533638	Up
REACTOME_G_ALPHA_S_SIGNALLING_EVENTS	0.106199431698099	-0.003014861	3.43811022753571	0.000675526048617443	0.00188788391885118	-1.007174007	Up
REACTOME_ANCHORING_FIBRIL_FORMATION	0.172809527646896	-0.006422655	3.43718979691612	0.000677731162069532	0.0018923424754681	-1.010175945	Up
REACTOME_CA2_PATHWAY	0.0805949924233047	0.00458143838645031	3.43443952988462	0.000684360239763847	0.00190827673324986	-1.01914141	Up
REACTOME_DNA_DAMAGE_TELOMERE_STRESS_INDUCED_SENESCENCE	0.119764086514834	0.0101949021965253	3.43173108542647	0.000690947691588912	0.0019240521855955	-1.027964104	Up
CHIANG_LIVER_CANCER_SUBCLASS_UNANNOTATED_UP	0.088694801886919	0.0030600043715989	3.43093061497706	0.000692905902519866	0.00192863988203624	-1.030570395	Up
LIM_MAMMARY_LUMINAL_PROGENITOR_UP	0.11842699644852	0.000660378456856659	3.43061512325357	0.000693679121602185	0.00192992662832127	-1.031597467	Up
WONG_ENDMETRIUM_CANCER_DN	0.113205737358662	-0.00171693	3.42487690929737	0.000707884007768138	0.00196592216385541	-1.050262887	Up
WP_GDNFRET_SIGNALLING_AXIS	0.12616181485938	-0.009447981	3.4240400186	0.0007099782852	0.0019699755102	-1.05298	Up

			8031	17898	1345	2752	
REACTOME_NEUROFASCIN_INTERACTIONS	0.194092496721373	-0.004990558	3.42198546616055	0.00071514427430996	0.00198253707487357	-1.059657386	Up
KEGG_ACUTE_MYELOID_LEUKEMIA	0.111373089824217	0.00753833737349842	3.41828270719189	0.00072454330039322 4	0.00200501126417331	-1.07167727	Up
WP_MELATONIN_METABOLISM_AND_EFFECTS	0.099636114074036	0.00663042171887365	3.41796855542844	0.00072534602531 9011	0.00200633813687839	-1.072696519	Up
FOURNIER_ACINAR_DEVELOPMENT_EARLY_DN	0.223986228143276	0.00482676924268664	3.41701235158846	0.00072779444533304	0.00201131795288683	-1.075798342	Up
BYSTRYKH_HEMATOPOIESIS_STEM_CELL_SCP2_QTL_TRANS	0.117052525666151	-0.002047451	3.41396508627554	0.00073564875203 0366	0.00202871199402069	-1.085678027	Up
PID_SYNDECAN_4_PATHWAY	0.143353441629482	0.0009880140558263	3.41388121061559	0.00073586605623 3133	0.00202871199402069	-1.08594985	Up
PID_AR_PATHWAY	0.0892756327121636	0.00432560643411967	3.41247099520518	0.00073952861610 6645	0.00203739641374787	-1.090519134	Up
GUENTHER_GROWTH_SPHERICAL_VS_ADHERENT_UP	0.134963237958869	-0.004822685	3.41218430218226	0.00074027528378 1823	0.0020385486630141	-1.091447846	Up
DELACROIX_RAR_TARGETS_DN	0.134189077420417	-0.0016954	3.4108	0.000743	0.002045	-1.0	Up

		3	134490003	855295266965	68445623485	95887586	
PLASARI_TGFB1_SIGNALING_VIA_NFIC_1HR_DN	0.0696007872180173	0.00274934684401064	3.40724005428813	0.000753263426470154	0.00207064042874236	-1.107452929	Up
MEISSNER_ES_ICP_WITH_H3K4ME3	0.106417990921774	0.000277754262452811	3.39660426755141	0.000781927164543707	0.00214563302843625	-1.141809885	Up
MEBARKI_HCC_PROGENITOR_WNT_UP	0.1111697374178	-0.004548744	3.39629099988589	0.000782786664218911	0.00214704234414794	-1.142820343	Up
ALONSO_METASTASIS_DN	0.117916147333323	-0.002541991	3.39540995463712	0.00078520868124479	0.00215273422459647	-1.145661729	Up
BIOCARTA_ACETAMINOPHEN_PATHWAY	0.192968623425632	-0.002556037	3.39360341126247	0.000790196804602297	0.00216545323009557	-1.151485744	Up
KEGG_OLFACTORY_TRANSDUCTION	0.122739955729258	-0.003548335	3.39258607447492	0.000793018814903422	0.00217222761875796	-1.154764226	Up
BIOCARTA_PRION_PATHWAY	0.155309266082976	0.00789153404396675	3.39180639636186	0.000795187941544121	0.00217528935793934	-1.157276214	Up
BIOCARTA_PLCD_PATHWAY	0.203869619443275	0.0032234607793245	3.38902597868261	0.000802968440511037	0.00219463897413122	-1.166229918	Up

REACTOME_HIGHLY_CALCIUM_PERMEABLE_NICOTINIC_ACETYLCHOLINE_RECEPTORS	0.179812333893554	-0.008639181	3.38638740001248	0.000810417660616769	0.00221304989856942	-1.174720618	Up
REACTOME_ACTIVATION_OF_AMPK_DOWNSTREAM_OF_NMDARS	0.1431984303515	-0.002143976	3.38413878321169	0.000816816773172816	0.00222758423158333	-1.181951657	Up
MIKKELSEN_MEF_ICP_WITH_H3K27ME3	0.107018387260877	-0.003174165	3.3780088314518	0.000834501573805717	0.00227281758166393	-1.201641747	Up
FINETTI_BREAST_CANCER_KINOME_GREEN	0.155534212359632	0.00433223616363402	3.37248122737813	0.000850754162762281	0.00231505089358416	-1.219368866	Up
KRIEG_HYPOXIA_NOT_VIA_KDM3A	0.0511594119527596	0.00303211884894819	3.37222762455396	0.000851506855524795	0.00231608372140333	-1.220181534	Up
REACTOME_ROBO_RECEPTORS_BIND_AKAP5	0.145279439596852	0.00363020443596017	3.37170260665735	0.000853067081516298	0.00231931115855088	-1.22186377	Up
VISALA_AGING_LYMPHOCYTE_UP	0.194420102927156	0.00536112764608877	3.37088393136197	0.000855505305899078	0.00232492181861452	-1.224486447	Up
SERVITJA_LIVER_HNF1A_TARGETS_DN	0.075432785017498	-0.003080598	3.36764713318047	0.000865209087868854	0.00234820848640226	-1.234849989	Up
REACTOME_SIGNALING_BY_NTRKS	0.0875633944263604	0.0040382725029952	3.3656258356	0.0008713207946	0.0023634345521	-1.24131	Up

		8	0444	61137	5131	7107	
OUYANG_PROSTATE_CANCER_MARKERS	0.129578342379638	-0.008873794	3.36188143479983	0.000882749029946199	0.00238850184345077	-1.253287831	Up
MYLLYKANGAS_AMPLIFICATION_HOT_SPOT_13	0.199300324086152	-0.007532711	3.36043686672173	0.000887195204324553	0.00239739687994885	-1.257902787	Up
STREICHER_LSM1_TARGETS_UP	0.0853860582517174	0.0018860127234715	3.35647686446604	0.000899490784814789	0.00242850774308195	-1.270544424	Up
BIOCARTA_MCALPAIN_PATHWAY	0.124415401094977	0.00101061703682904	3.35589534423244	0.000901309685949289	0.00243077697355678	-1.272399673	Up
RYAN_MANTLE_CELL_LYMPHOMA_NOTCH_DIRECT_UP	0.092899370060282	0.00618290848059262	3.35583260234503	0.000901506137123846	0.00243077697355678	-1.272599824	Up
REACTOME_HIGHLY_CALCIUM_PERMEABLE_POSTSYNAPTIC_NICOTINIC_ACETYLCHOLINE_RECEPTORS	0.172557400331752	-0.007597853	3.35513818132665	0.000903683105607196	0.00243558881307159	-1.274814839	Up
TERAMOTO_OPN_TARGETS_CLUSTER_5	0.190715878741261	0.00610844410292096	3.35318928766196	0.000909818964284082	0.00244999839970121	-1.281029028	Up
ZHONG_RESPONSE_TO_AZACITIDINE_AND_TSA_UP	0.0693115110627796	-0.000580304	3.35205109409517	0.000913420363025765	0.00245862974557716	-1.284656702	Up
IGLESIAS_E2F_TARGETS_DN	0.152717822505284	-0.0073077	3.3515	0.000915	0.002461	-1.2	Up

		33	330357106	063964611261	98614145735	86307493	
WP_MFAP5MEDIATED_OVARIAN_CANCER_CELL_MOTILITY_AND_INVASIVENESS	0.137829124376258	-0.000870988	3.35014775908325	0.000919472463543746	0.00247277538180937	-1.290720516	Up
SATO_SILENCED_BY_METHYLATION_IN_PANCREATIC_CANCER_1	0.0762914935790348	-0.002788302	3.34975492766036	0.000920726205120319	0.00247507473156423	-1.291971639	Up
KEGG_NOTCH_SIGNALING_PATHWAY	0.117645802007483	0.00769645415747524	3.3467413683522	0.00093039723783014	0.0024999894611306	-1.301564978	Up
WP_GPCRS_CLASS_A_RHODOPSINLIKE	0.101125634803502	-0.001040202	3.34407529297526	0.000939031937863835	0.00252100832107302	-1.310045508	Up
REACTOME_PROTEIN_PROTEIN_INTERACTIONS_AT_SYNAPSES	0.103817034182541	-0.004877902	3.34289370320952	0.000942882609119058	0.00252915832100346	-1.313802039	Up
REACTOME_SIGNALING_BY_MODERATE_KINASE_ACTIVITY_BRAF_MUTANTS	0.121648898016691	0.00680679119378404	3.34264630528477	0.000943690711533196	0.00253023250388188	-1.314588416	Up
SARTIPY_BLUNTED_BY_INSULIN_RESISTANCE_DN	0.116206442047758	0.00365700062059915	3.34252127301623	0.000944099363197252	0.00253023521043409	-1.314985823	Up
WP_MAJOR_RECEPTORS_TARGETED_BY_EPINEPHRINE_AND_NOREPINEPHRINE	0.155680175098434	-0.010775328	3.34210030352864	0.000945476458997603	0.00253283227492366	-1.316323743	Up

PETRETTO_LEFT_VENTRICLE_MASS_QTL_CIS_DN	0.179500987059741	0.0110171544813018	3.3414308386222	0.000947670298770791	0.00253675090173392	-1.31845111	Up
SCHAEFFER_SOX9_TARGETS_IN_PROSTATE_DEVELOPMENT_UP	0.118920549675588	-0.005028952	3.34140452660633	0.000947756620125818	0.00253675090173392	-1.318534714	Up
PID_AMB2_NEUTROPHILS_PATHWAY	0.135059982332175	0.0018711879437778	3.3393236609571	0.000954606514308456	0.00255288351327556	-1.325144549	Up
REACTOME_NEUROTRANSMITTER_RECEPTORS_AND_POSTSYNAPTIC_SIGNAL_TRANSMISSION	0.0873024767219855	-0.002981964	3.33635081165778	0.00096447269723863	0.00257704779671122	-1.334581171	Up
SEKI_INFLAMMATORY_RESPONSE_LPS_DN	0.132718365680409	-0.001177764	3.32983294855138	0.000986437153944484	0.00263346899549824	-1.3552435	Up
REACTOME_DNA_DOUBLE_STRAND_BREAK_RESPONSE	0.10136242299668	0.00296877304621317	3.32769078408458	0.00099375707816867	0.0026518702425593	-1.362026257	Up
NABA_COLLAGENS	0.132869034720781	-0.006548887	3.32616581523793	0.000998998775873755	0.00266239068896813	-1.366852329	Up
REACTOME_COLLAGEN_CHAIN_TRIMERIZATION	0.132869034720781	-0.006548887	3.32616581523793	0.000998998775873755	0.00266239068896813	-1.366852329	Up
SILIGAN_BOUND_BY_EWS_FLT1_FUSION	0.0902793992022237	-0.001153328	3.3260449769	0.0009994152255	0.0026623906889	-1.36723	Up

			5563	99441	6813	4659	
REACTOME_NETRIN_1_SIGNALING	0.0881672853941538	0.0010555469147179	3.32538999151954	0.00100167534067737	0.00266726676944848	-1.369306796	Up
SCHAEFFER_PROSTATE_DEVELOPMENT_48HR_DN	0.0730580335351563	-0.000561694	3.32251233166191	0.00101166156826899	0.00269270298209503	-1.378406214	Up
BLANCO_MELO_RESPIRATORY_SYNCYTIAL_VIRUS_INFECTION_A594_CELLS_DN	0.110360115411931	-0.005021879	3.32143930208548	0.00101540891711237	0.00270151870917982	-1.38179737	Up
BRIDEAU_IMPRINTED_GENES	0.0661534101318482	-0.000875764	3.32111847386079	0.00101653185381841	0.00270334756497465	-1.382811105	Up
WP_SRF_AND_MIRS_IN_SMOOTH_MUSCLE_DIFFERENTIATION_AND_PROLIFERATION	0.139717591982295	0.00882648275708578	3.32097277176606	0.00101704220986658	0.00270354646537125	-1.383271456	Up
DUTERTRE_ESTRADIOL_RESPONSE_6HR_UP	0.0581001661416437	-0.000206448	3.32074174941366	0.00101785190908163	0.00270454058205037	-1.384001343	Up
BIOCARTA_DICER_PATHWAY	0.25059282964209	0.00727353659337557	3.3197554767881	0.00102131540058007	0.00271258223851112	-1.387111682	Up
WILCOX_RESPONSE_TO_PROGESTERONE_DN	0.107348309615619	-0.002007217	3.31880881355576	0.00102465010795894	0.00272027511552658	-1.390106375	Up
WP_METABOLIC_PATHWAY_OF_LDL_HDL_AND_TG_IN	0.141524162808405	-0.0094086	3.3182	0.001026	0.002723	-1.3	Up

CLUDING_DISEASES		38	117784<br>5451	7584251<br>9813	5425406<br>8581	9199<br>1402	
MEBARKI_HCC_PROGENITOR_WNT_UP_CTNNB1_DEPE<br>NDENT	0.103142817880776	-0.0044358<br>09	3.3178<br>675825<br>2024	0.001027<br>9757220<br>5459	0.002725<br>6067094<br>3735	-1.3<br>9307<br>7994	Up
JAZAG_TGFB1_SIGNALING_VIA_SMAD4_DN	0.0849634191814087	-0.0057380<br>97	3.3162<br>965440<br>9809	0.001033<br>5489899<br>6693	0.002738<br>0446353<br>9254	-1.3<br>9803<br>6286	Up
HEIDENBLAD_AMPLICON_12P11_12_DN	0.118713864144518	0.0025318<br>044104332<br>2	3.3154<br>559249<br>6325	0.001036<br>5426244<br>3944	0.002744<br>7489145<br>906	-1.4<br>0068<br>8441	Up
HAN_SATB1_TARGETS_DN	0.0334148288617185	0.0001466<br>808676078<br>73	3.3153<br>377535<br>1479	0.001036<br>9641058<br>0231	0.002744<br>7489145<br>906	-1.4<br>0106<br>1222	Up
REACTOME_ADHERENS_JUNCTIONS_INTERACTIONS	0.135711039894887	-0.0045636<br>32	3.3145<br>162635<br>2487	0.001039<br>8985189<br>2584	0.002750<br>1704759<br>1509	-1.4<br>0365<br>234	Up
BLANCO_MELO_COVID19_SARS_COV_2_INFECTION_A<br>594_CELLS_DN	0.119096441179309	-0.0073887<br>52	3.3134<br>926336<br>6633	0.001043<br>5657989<br>5226	0.002758<br>6937453<br>5633	-1.4<br>0688<br>0211	Up
RODRIGUES_THYROID_CARCINOMA_POORLY_DIFFER<br>ENTIATED_DN	0.0673280497648655	0.0055678<br>738089998<br>3	3.3133<br>638769<br>4697	0.001044<br>0279366<br>8151	0.002758<br>7404865<br>8244	-1.4<br>0728<br>6162	Up
WP_NICOTINE_ACTIVITY_ON_DOPAMINERGIC_NEURO<br>NS	0.132824878821664	-0.0002688<br>13	3.3123<br>471876<br>515	0.001047<br>6837631<br>04	0.002767<br>2226032<br>2832	-1.4<br>1049<br>1122	Up

WP_HEART_DEVELOPMENT	0.107951852843654	-0.00105972	3.31153499731626	0.00105061280199107	0.00277259934607082	-1.413050777	Up
MCGARVEY_SILENCED_BY_METHYLATION_IN_COLON_CANCER	0.115588885477433	-0.003212027	3.3106760570078	0.00105371872404357	0.00277961416070483	-1.415757136	Up
BIOCARTA_VITCB_PATHWAY	0.142190656726378	-0.000238253	3.30712648965928	0.00106664473248766	0.00280893672233811	-1.426934283	Up
WP_TCELL_ANTIGEN_RECEPTOR_TCR_PATHWAY_DURING_STAPHYLOCOCCUS_AUREUS_INFECTION	0.0960778259434002	0.00692658593724201	3.30559529831282	0.00107226607950344	0.00282254264439265	-1.431752398	Up
REACTOME_NR1H2_NR1H3_REGULATE_GENE_EXPRESSION_LINKED_TO_LIPOGENESIS	0.161525256361814	0.00139228093036766	3.30495106132304	0.00107463944276781	0.00282759093737167	-1.433778966	Up
MOHANKUMAR_HOXA1_TARGETS_UP	0.0647424673741295	0.00671907980782539	3.30416952839176	0.00107752516107546	0.00283278215789724	-1.436236936	Up
WP_APOE_AND_MIR146_IN_INFLAMMATION_AND_ATHEROSCLEROSIS	0.211557976798686	0.00477024885314893	3.30031665361522	0.00109185707610301	0.00286681762748367	-1.448346619	Up
LIU_VAV3_PROSTATE_CARCINOGENESIS_DN	0.13270206728278	-0.003377128	3.29955385758886	0.00109471546027637	0.002871893010164	-1.450742559	Up
WP_CELL_DIFFERENTIATION_INDEX	0.162352008336498	-0.012030645	3.2993760975	0.0010953825695	0.0028724290702	-1.45130	Up

			8947	5265	2107	083	
REACTOME_CELL_SURFACE_INTERACTIONS_AT_THE_VASCULAR_WALL	0.0773101227567408	0.00169808813066799	3.29872346504232	0.00109783505413599	0.00287764450212081	-1.453350238	Up
FONTAINE_PAPILLARY_THYROID_CARCINOMA_UP	0.0811983101885194	0.000376382269416516	3.29513995523918	0.00111139250986803	0.00290581857992875	-1.46459657	Up
WP_DRUG_INDUCTION_OF_BILE_ACID_PATHWAY	0.136607671366814	-0.001538358	3.29481610117633	0.0011126253814301	0.00290781715475227	-1.465612383	Up
KEGG_VASCULAR_SMOOTH_MUSCLE_CONTRACTION	0.0946116663214031	-0.004100145	3.29380122550262	0.00111649712474409	0.00291670776653475	-1.468795084	Up
REACTOME_POTASSIUM_CHANNELS	0.112882952811944	-0.003535925	3.29303121633973	0.00111944305618743	0.00292317334865602	-1.471209267	Up
LIM_MAMMARY_LUMINAL_PROGENITOR_DN	0.152570120735504	-0.010293911	3.29050168594732	0.00112917156577814	0.00294609832231396	-1.479136346	Up
REACTOME_ACYL_CHAIN_REMODELLING_OF_PS	0.119749276402241	0.000131759119701752	3.28740442928387	0.00114119054601215	0.00297403901582072	-1.488834908	Up
WP_DEVELOPMENT_OF_PULMONARY_DENDRITIC_CELLS_AND_MACROPHAGE_SUBSETS	0.127568976154787	-0.003123748	3.28318200468458	0.00115776735675663	0.00301437163732736	-1.502043157	Up
WP_MAMMALIAN_DISORDER_OF_SEXUAL_DEVELOPM	0.12974017618144	-0.0047710	3.2786	0.001175	0.003054	-1.5	Up

ENT		3	8210653594	67959436424	60160829588	16102105	
REACTOME_DISEASES_OF_CARBOHYDRATE_METABOLISM	0.125152648085602	0.00102894324223712	3.27736876853774	0.0011809557851228	0.00306574343716321	-1.520201982	Up
REACTOME_TRAFFICKING_OF_AMPA_RECEPTORS	0.106278171138535	-0.003453017	3.27712767032339	0.00118192675710063	0.00306698134670719	-1.520954459	Up
HE_PTEN_TARGETS_UP	0.112768672103284	-0.005872138	3.27537897366797	0.00118899148978625	0.00308273608066134	-1.526410676	Up
FOSTER_KDM1A_TARGETS_DN	0.0742452979004733	-0.002246683	3.27145671866086	0.00120498029412009	0.00312028063646366	-1.538638955	Up
YU_MYC_TARGETS_DN	0.104359550589215	0.00751474509380138	3.27119966750414	0.00120603508670884	0.00312170966772383	-1.539439882	Up
WP_HFE_EFFECT_ON_HEPCIDIN_PRODUCTION	0.217944148466786	-0.015766066	3.27098980152858	0.00120689689378975	0.00312263819080992	-1.540093744	Up
BIOCARTA_PLK3_PATHWAY	0.1646180141367	-0.002873385	3.2667528986273	0.00122441838027694	0.00316533314717992	-1.553286023	Up
KUROZUMI_RESPONSE_TO_ONCOCYTIC_VIRUS_AND_CYCLIC_RGD	0.117665880773136	0.00382742216631976	3.26440994969844	0.00123420865593892	0.0031853360197143	-1.56057438	Up

REACTOME_TRANSPORT_OF_FATTY_ACIDS	0.173328722825614	-0.013580792	3.26326306641866	0.00123902748871884	0.00319378887976654	-1.564140297	Up
REACTOME_SIGNALING_BY_BRAF_AND_RAF_FUSIONS	0.103701270700663	0.00871079250757208	3.26204531846537	0.00124416318858118	0.00320436552345368	-1.56792528	Up
WP_MAMMARY_GLAND_DEVELOPMENT_PATHWAY_PREGNANCY_AND_LACTATION_STAGE_3_OF_4	0.104558698898265	-0.000313711	3.26064757568609	0.00125008235171378	0.00321827505478532	-1.572268108	Up
BIOCARTA_PLATELETAPP_PATHWAY	0.166130699339215	-0.001560198	3.26009981577405	0.00125240912796687	0.00322292846487992	-1.573969545	Up
ONDER_CDH1_TARGETS_3_UP	0.11005960581288	-0.001680906	3.2563476936593	0.00126845581321749	0.00326151832338068	-1.585617172	Up
BLANCO_MELO_COVID19_SARS_COV_2_INFECTION_A594_ACE2_EXPRESSING_CELLS_DN	0.0818700518411296	-0.005271813	3.25497981348376	0.00127435313614714	0.00327532501700426	-1.589860363	Up
REACTOME_SEMA3A_PAK_DEPENDENT_AXON_REPULSION	0.139596740487584	0.0118326528090497	3.24950590852112	0.00129820829629221	0.00333105082882561	-1.60682401	Up
MYLLYKANGAS_AMPLIFICATION_HOT_SPOT_2	0.189490339603278	0.0118461641399577	3.24899521025336	0.00130045491316564	0.00333412790004921	-1.608405318	Up
SIMBULAN_UV_RESPONSE_NORMAL_DN	0.0884369027224377	0.0029435004934695	3.2473453328	0.0013077375791	0.0033500318422	-1.61351	Up

		3	897	4961	5409	2365	
WP_MONOAMINE_GPCRS	0.133998745677537	-0.005230313	3.24483148654976	0.00131890664293052	0.00337585712687411	-1.621289134	Up
REACTOME_TRANSCRIPTIONAL_REGULATION_OF_WHITE_ADIPOCYTE_DIFFERENTIATION	0.0816420496591552	0.00262024249755332	3.24216953073962	0.00133083018808732	0.00340076697301688	-1.629518011	Up
KEGG_ENDOMETRIAL_CANCER	0.1000125412499	0.0109331407302273	3.24088051374201	0.00133663987139967	0.00341420727645174	-1.633500488	Up
SMID_BREAST_CANCER_RELAPSE_IN_BONE_UP	0.0856377034135467	-0.001796471	3.23898275447587	0.00134523602071299	0.00343152234580119	-1.639361034	Up
REACTOME_FCGR_ACTIVATION	0.153320263164718	0.0086885873282386	3.2356001850588	0.00136068508086104	0.00346848964965275	-1.649798996	Up
PID_RAC1_REG_PATHWAY	0.11142385517906	0.0090864475066376	3.23346209281964	0.0013705349958077	0.00348643881925345	-1.656391529	Up
BIOCARTA_BIOPEPTIDES_PATHWAY	0.123790621508323	0.00529128460364761	3.23144021746871	0.00137991030216781	0.0035074132864683	-1.662622007	Up
REACTOME_DISEASES_OF_GLYCOSYLATION	0.0693947091165695	-0.003997088	3.23027532180164	0.00138533881348691	0.00351976996124161	-1.666210033	Up
WP_TGFB_SIGNALING_IN_THYROID_CELLS_FOR_EPIT	0.136599053203594	0.0017802	3.2267	0.001401	0.003560	-1.6	Up

HELIALMESENCHYMAL_TRANSITION		1408385061	4634807894	90521371869	40329850732	77072404	
ZIRN_TRETINOIN_RESPONSE_DN	0.182185443997018	-0.00168066	3.22622255825061	0.00140437968502429	0.00356522891817823	-1.678683719	Up
MIKKELSEN_IPS_WITH_HCP_H3K27ME3	0.115255760781118	-0.004715408	3.22211178885417	0.00142394069121438	0.00360751015117048	-1.69132111	Up
LI_CYTIDINE_ANALOGS_CYCTOTOXICITY	0.104499375124486	0.00639354885280561	3.21902804241952	0.00143878007655142	0.00364361808859839	-1.700791407	Up
REACTOME_ERKS_ARE_INACTIVATED	0.136945054706496	0.00122364928059822	3.21771772784823	0.00144512876006271	0.00365820318666773	-1.704812885	Up
REACTOME_NOTCH1_INTRACELLULAR_DOMAIN_REGULATES_TRANSCRIPTION	0.0973494287951968	0.00437177817139326	3.21515906542782	0.00145760079282634	0.00368827073830946	-1.712661284	Up
WEST_ADRENOCORTICAL_CARCINOMA_VS_ADENOMA_UP	0.114276198077105	-0.003034329	3.21284157377135	0.00146898325932043	0.00371555790163076	-1.719764923	Up
REACTOME_GP1B_IX_V_ACTIVATION_SIGNALLING	0.177919409580674	0.000626063617016312	3.21266740094076	0.00146984203579401	0.00371621568886901	-1.72029861	Up
MATZUK_IMPLANTATION_AND_UTERINE	0.130139560898401	-0.007191155	3.21116576682903	0.00147726530777222	0.0037319437384 38	-1.724898693	Up

BROWNE_HCMV_INFECTION_2HR_DN	0.0849651479161703	0.0020323724210530 2	3.2053896270 5115	0.0015061443752 967	0.0037969542176 4051	-1.742574636	Up
WP_PREGNANE_X_RECEPTOR_PATHWAY	0.0970155022675797	-0.0040219 48	3.2052855281 2468	0.0015066696049 6996	0.0037969542176 4051	-1.742892925	Up
WP_IL3_SIGNALING_PATHWAY	0.143823164316808	0.0121545244350584	3.2041412874 2741	0.0015124540466 1827	0.0038084390536 9557	-1.746390882	Up
REACTOME_INTERLEUKIN_9_SIGNALING	0.208932665347248	0.0052324019960927 8	3.2019376984 7206	0.0015236517627 0727	0.0038335251281 4108	-1.753124015	Up
PID_REELIN_PATHWAY	0.120844219290315	0.0023037347967473 1	3.2010773612 1429	0.0015280444377 312	0.0038414628695 8183	-1.755751635	Up
REACTOME_NEF_MEDIATES_DOWN_MODULATION_OF_CELL_SURFACE_RECEPTORS_BY_RECRUITING_THEM_TO_CLATHRIN_ADAPTERS	0.12284509725631	0.0069826445276533 1	3.1969036263 2392	0.0015495214537 0807	0.0038907280190 8009	-1.768489649	Up
SU_LIVER	0.108582063474644	-0.0044158 67	3.1966730293 0223	0.0015507161583 205	0.0038916340127 6575	-1.769192969	Up
KEGG_GNRH_SIGNALING_PATHWAY	0.0908438553526501	-0.0018163 92	3.1951600603 0287	0.0015585759075 7207	0.0039039810492 3294	-1.773806351	Up
REACTOME_VEGFR2_MEDIATED_VASCULAR_PERMEABILITY	0.102067815057344	0.0062895459303676	3.1950397758	0.0015592023555	0.0039039810492	-1.77417	Up

		4	0889	741	3294	3039	
WP_SOMATIC_SEX_DETERMINATION	0.150247863655753	-0.004926633	3.19164504060836	0.00157697873419958	0.00394371756775857	-1.78451661	Up
ODONNELL_TFRC_TARGETS_UP	0.0668328296848516	0.00142378313439456	3.18840238713969	0.00159413375423822	0.00398128396145901	-1.794387264	Up
REACTOME_NUCLEOBASE_BIOSYNTHESIS	0.199857337012294	0.00797430026760758	3.18574878661381	0.00160830076641544	0.00401396174392466	-1.802457908	Up
ZHENG_GLIOBLASTOMA_PLASTICITY_DN	0.106341160066406	0.00259131238075066	3.18367999358724	0.00161942634383056	0.00403685916311498	-1.808745593	Up
GAUSSMANN_MLL_AF4_FUSION_TARGETS_D_DN	0.15145678692202	-0.00454082	3.17969019642904	0.00164108408737903	0.00408453839913478	-1.820861077	Up
JIANG_CORE_DUPLICON_GENES	0.220201897189917	0.0132225354617324	3.17924100422256	0.00164353914073447	0.00408875649159874	-1.822224218	Up
REACTOME_FOXO_MEDIATED_TRANSCRIPTION_OF_OXIDATIVE_STRESS_METABOLIC_AND_NEURONAL_GENES	0.0864542213110554	0.00180090115199188	3.17806774556983	0.00164996760564258	0.00410258182262376	-1.825783803	Up
REACTOME_TRANSCRIPTIONAL_ACTIVATION_OF_MITOCHONDRIAL_BIOGENESIS	0.0844151457362301	0.00514625388824102	3.17706446310526	0.00165548318690536	0.00411352447602946	-1.828826723	Up
HOEBEKE_LYMPHOID_STEM_CELL_UP	0.0950170287986225	0.0057009	3.1704	0.001692	0.004199	-1.8	Up

		304222797	916103879	04166545622	32131846733	48739878	
MIKKELSEN_IPS_ICP_WITH_H3K4ME3_AND_H327ME3	0.102781625369883	-0.001921078	3.16910973666299	0.00169982207756104	0.00421694469840981	-1.852921536	Up
ZHU_CMV_8_HR_DN	0.0995817030938469	0.00592906093519479	3.16672848319361	0.00171330700413845	0.00424361395637964	-1.860123421	Up
REACTOME_RECRUITMENT_OF_NUMA_TO_MITOTIC_CENTROSOMES	0.0998260720994407	0.000309463706956999	3.16346039918382	0.00173197508384543	0.00428301567546956	-1.869999253	Up
REACTOME_SUMOYLATION_OF_CHROMATIN_ORGANIZATION_PROTEINS	0.121712863306166	0.00897013454565778	3.16287354626255	0.00173534717945063	0.00428964553677819	-1.871771662	Up
FIGUEROA_AML_METHYLATION_CLUSTER_3_DN	0.0986980913344263	0.00295899658559017	3.15980633209914	0.00175307057074981	0.00433000757367451	-1.881030265	Up
MEBARKI_HCC_PROGENITOR_FZD8CRD_DN	0.0840392468357994	-0.003857872	3.15931040674874	0.00175595185834816	0.00433367522257138	-1.882526466	Up
REACTOME_MUCOPOLYSACCHARIDOSES	0.198213637521971	-0.003296357	3.15674321346813	0.00177093715638832	0.00436545152093023	-1.890268171	Up
WP_RESISTIN_AS_A_REGULATOR_OF_INFLAMMATION	0.110547433673766	0.00201828767744111	3.15490048922109	0.0017817663371616	0.00438691933945342	-1.895821537	Up

REACTOME_CALCITONIN_LIKE_LIGAND_RECEPTORS	0.172783147669207	-0.003062147	3.14980064124676	0.00181205660438593	0.00445619466855129	-1.911175085	Up
KEGG_CHRONIC_MYELOID_LEUKEMIA	0.0957179291746513	0.00678371332222355	3.14913935742453	0.00181601892346164	0.00446417008234709	-1.913164247	Up
WEBER_METHYLATED_HCP_IN_SPERM_UP	0.119782643565511	-0.008295181	3.14688564204682	0.00182958307700648	0.00449395415867798	-1.919940574	Up
REACTOME_ROLE_OF_SECOND_MESSENGERS_IN_NETRIN_1_SIGNALING	0.136256732505424	-0.008538425	3.14582154285707	0.00183601991229506	0.00450619833753081	-1.923138471	Up
REACTOME_VITAMIN_D_CALCIFEROL_METABOLISM	0.152965946695211	0.00299732152121889	3.14083923091383	0.0018664376079651	0.00457361951545178	-1.93809824	Up
WP_SCFA_AND_SKELETAL_MUSCLE_SUBSTRATE_METABOLISM	0.199543441657823	-2.57E-05	3.14067930234896	0.00186742165984695	0.00457422503657065	-1.938578072	Up
WP_ROLE_OF_ALTERED_GLYCOLYSATION_OF_MUC1_IN_TUMOUR_MICROENVIRONMENT	0.147909806067955	-0.001198163	3.13816347905725	0.00188296489314749	0.00461047853718597	-1.946123281	Up
REACTOME_G_ALPHA_Z_SIGNALLING_EVENTS	0.0982528786014305	-0.003882588	3.13639671274032	0.00189395162765045	0.00463372398613573	-1.951418629	Up
DORN_ADENOVIRUS_INFECTION_48HR_DN	0.103168014070752	0.0090659440408786	3.1355134371	0.0018994664933	0.0046435559371	-1.95406	Up

			0731	7327	2796	494	
REACTOME_GALACTOSE_CATABOLISM	0.143976978856069	0.0046478420996222<br>9	3.1330561962<br>4015	0.0019148867501<br>1941	0.0046757285830<br>0204	-1.96142<br>3232	Up
STAEGE_EWING_FAMILY_TUMOR	0.0973515182746595	0.0029841494375682<br>8	3.1322301011<br>4785	0.0019200967618<br>4494	0.0046866066066<br>7383	-1.96389<br>5796	Up
TOMLINS_PROSTATE_CANCER_DN	0.106645848000871	0.0084112497914465<br>7	3.1316383998<br>9636	0.0019238365405<br>6288	0.0046938889179<br>5353	-1.96566<br>6429	Up
REACTOME_INTERLEUKIN_RECEPTOR_SHC_SIGNALIN<br>G	0.130404599038466	0.0131206<br>012038461	3.1294568237<br>2683	0.0019376831175<br>1398	0.0047239588022<br>0318	-1.97219<br>1983	Up
REACTOME_ACTIVATION_OF_GENE_EXPRESSION_BY_<br>SREBF_SREBP_	0.0838470924703232	0.0025556779139948<br>9	3.1286101330<br>4501	0.0019430818447<br>734	0.0047317945282<br>1865	-1.97472<br>3473	Up
REACTOME_LECTIN_PATHWAY_OF_COMPLEMENT_AC<br>TIVATION	0.18498576530211	0.0058182544961043<br>9	3.1285941034<br>4569	0.0019431841875<br>9938	0.0047317945282<br>1865	-1.97477<br>1393	Up
BOYLAN_MULTIPLE_MYELOMA_C_UP	0.113116669022169	0.0085975430540540<br>6	3.1272388859<br>0061	0.0019518547459<br>0683	0.0047491816573<br>2799	-1.97882<br>1963	Up
BIOCARTA_CB1R_PATHWAY	0.192820051418554	-0.0158954<br>04	3.1255860415<br>2177	0.0019624778260<br>3784	0.0047731582547<br>8717	-1.98375<br>9889	Up
WP_SIGNALING_PATHWAYS_IN_GLIOBLASTOMA	0.0806350506436565	0.0080436	3.1224	0.001982	0.004817	-1.9	Up

		558589231	2316493489	95501476871	30010828548	93202308	
DEN_INTERACT_WITH_LCA5	0.175057662061354	0.00780638486982334	3.12227701853396	0.00198390594919917	0.00481772465832521	-1.993638396	Up
REACTOME_REGULATION_OF_INSULIN_SECRETION	0.0944767309942955	-0.00233241	3.12127940317286	0.00199040840716876	0.00483162494458212	-1.99661469	Up
ST_PAC1_RECEPTOR_PATHWAY	0.15852276471641	-0.001332501	3.11741912179597	0.00201575544948659	0.00488551115773655	-2.00812314	Up
REACTOME_RUNX3_REGULATES_IMMUNE_RESPONSE_AND_CELL_MIGRATION	0.154674539916785	0.00311995883536665	3.11622016611046	0.00202368833349808	0.00490282337471608	-2.011694823	Up
SHEDDEN_LUNG_CANCER_GOOD_SURVIVAL_A4	0.0601249300403021	0.00148833628225094	3.11423902321741	0.00203685969769901	0.0049328084836589	-2.017593832	Up
REACTOME_PLATELET_ACTIVATION_SIGNALING_AND_AGGREGATION	0.097800624276774	-0.001121558	3.11249667157619	0.00204850880202784	0.00495715170923463	-2.022778933	Up
LOPEZ_MESOTHELIOMA_SURVIVAL_OVERALL_DN	0.134000952953044	-0.008024742	3.11209643247066	0.00205119340663509	0.00496171374707093	-2.023969631	Up
WP_ESTROGEN_METABOLISM	0.140301153126014	-0.001726717	3.10609497825547	0.00209183927255853	0.00505019306292136	-2.041806649	Up

WP_OSTEOPONTIN_SIGNALING	0.147974215104026	0.00864807021746286	3.10587496119277	0.00209334339348552	0.0050518594258805	-2.042459956	Up
HESSON_TUMOR_SUPPRESSOR_CLUSTER_3P21_3	0.198307912857925	-0.008031871	3.10031701361585	0.00213167204621385	0.00513636971694464	-2.058949112	Up
KOYAMA_SEMA3B_TARGETS_UP	0.0436087520318659	-0.000314847	3.0995981621266	0.00213667635884793	0.0051464300191576	-2.061079769	Up
LI_WILMS_TUMOR	0.139034449	-0.000655266	3.09803130571785	0.00214762164274924	0.00517078647654947	-2.065722294	Up
NIKOLSKY_BREAST_CANCER_19P13_AMPLICON	0.199660283241144	-0.01492046	3.09600050394421	0.00216188472566122	0.0051990772925917	-2.071736212	Up
SMID_BREAST_CANCER_RELAPSE_IN_LUNG_DN	0.116025437742946	-0.007997478	3.09460266387661	0.00217175295766489	0.00521876523740844	-2.075873571	Up
GROSS_HYPOXIA_VIA_ELK3_ONLY_DN	0.0832946058388131	0.000307106469657426	3.09429969286232	0.00217389728750771	0.00522189646422614	-2.076770082	Up
CHIANG_LIVER_CANCER_SUBCLASS_PROLIFERATION_DN	0.0703913268161627	-0.001867078	3.09010995468322	0.00220375164956209	0.00528951527023661	-2.089159397	Up
HUANG_FOXA2_TARGETS_DN	0.11232889280375	-0.005106896	3.0897275275	0.0022064954001	0.0052920080945	-2.09028	Up

			6455	4286	4665	9478	
KEGG_SMALL_CELL_LUNG_CANCER	0.0639648745678719	0.00102582218978491	3.08834152169151	0.00221646579607782	0.00530976580326322	-2.094384064	Up
REACTOME_POU5F1_OCT4_SOX2_NANOG_ACTIVATE_GENES_RELATED_TO_PROLIFERATION	0.157762681806017	-0.00179008	3.0874155253419	0.00222315017316962	0.00532372419940733	-2.09711872	Up
MIKKELSEN_DEDIFFERENTIATED_STATE_DN	0.158525560271481	0.00372039339833056	3.08516326960181	0.00223948585664709	0.00535870806176118	-2.103766896	Up
NAKAMURA_METASTASIS	0.0838477351500052	0.00441353859363502	3.08289489873608	0.00225605013238582	0.00539245818817993	-2.110458066	Up
REACTOME_ABERRANT_REGULATION_OF_MITOTIC_G1_S_TRANSITION_IN_CANCER_DUE_TO_RB1_DEFECTS	0.128535197878589	0.00485748685973706	3.08287487387224	0.00225619686075451	0.00539245818817993	-2.110517114	Up
REACTOME_MOLECULES_ASSOCIATED_WITH_ELASTIC_FIBRES	0.111482092922748	-0.002721079	3.07251005298115	0.00233333389529864	0.00556824432453619	-2.141032324	Up
REACTOME_MITOCHONDRIAL_FATTY_ACID_BETA_OXIDATION_OF_SATURATED_FATTY_ACIDS	0.158702881940723	0.0122954085859587	3.06989740425014	0.00235315722533307	0.00561123584235205	-2.148709124	Up
PID_ERA_GENOMIC_PATHWAY	0.069450719082636	0.00419506978917637	3.06738064186797	0.00237239952068272	0.00564844028572214	-2.156098413	Up
REACTOME_FATTY_ACYL_COA_BIOSYNTHESIS	0.0753959215043258	0.0003163	3.0665	0.002378	0.005659	-2.1	Up

		40825084346	4808654333	79676348976	32982406322	58541575	
AMIT_EGF_RESPONSE_480_MCF10A	0.0847775296903269	-0.001093788	3.06287885804401	0.00240718024900298	0.00572246947742685	-2.169301673	Up
BIOCARTA_CCR5_PATHWAY	0.133082135917652	0.0141060774177497	3.06133842198639	0.00241918898906821	0.00574881548818775	-2.173815453	Up
KEGG_BUTANOATE_METABOLISM	0.0940283488502922	0.00942091652451247	3.05943428304053	0.00243410922249891	0.00578205738386941	-2.179392022	Up
REACTOME_XENOBIOTICS	0.125433173261604	-8.51E-05	3.05865355241279	0.00244025122086793	0.00579442973524379	-2.181677578	Up
HUANG_GATA2_TARGETS_DN	0.076510161750441	0.00316396936088262	3.0549456629219	0.00246961629019411	0.0058619152249464	-2.192524827	Up
ST_MYOCYTE_AD_PATHWAY	0.103676146924915	3.36879353122713e-05	3.04753195170327	0.00252930710483308	0.00599671856367416	-2.214176418	Up
CHICAS_RB1_TARGETS_CONFLUENT	0.0546296446098173	-0.002071134	3.04610687319737	0.00254093192148433	0.00602197954816848	-2.218332698	Up
REACTOME_CS_DS_DEGRADATION	0.147629358626898	-0.005849969	3.04567577849651	0.00254445816432702	0.00602803504808313	-2.219589639	Up

ST_INTERLEUKIN_4_PATHWAY	0.123724598438321	0.0115311467636502	3.04537043430484	0.0025469585223792	0.00603165644731312	-2.22047983	Up
BIOCARTA_TNFR2_PATHWAY	0.134223749490484	0.00231146049173585	3.04510399489191	0.00254914214824786	0.00603452529144717	-2.221256531	Up
MURAKAMI_UV_RESPONSE_24HR	0.121255517439616	-0.009485677	3.04423989216202	0.00255623582222374	0.00604901096017641	-2.22377505	Up
MATZUK_LUTEAL_GENES	0.146425184724626	-0.001978637	3.04174425434386	0.00257682528090758	0.00608949612284969	-2.2310451	Up
PETRETTO_HEART_MASS_QTL_CIS_UP	0.0837164253031896	0.00233077463347845	3.04157228150539	0.00257824968758643	0.00608949612284969	-2.23154587	Up
REACTOME_ELASTIC_FIBRE_FORMATION	0.119811249508344	-0.002193577	3.04067194362455	0.00258571877627575	0.00610481416673396	-2.23416714	Up
WP_MIRNA_TARGETS_IN_ECM_AND_MEMBRANE_RECEPTORS	0.136799257656259	-0.008990769	3.04006174366789	0.00259079222126883	0.0061144666606143	-2.235943282	Up
WP_ID_SIGNALING_PATHWAY	0.133919159664335	0.00159900165840056	3.03653156226785	0.00262032359884899	0.00617711681658021	-2.246212233	Up
REACTOME_PEXOPHAGY	0.144735723711268	0.0117799757724644	3.0337858590	0.0026435060585	0.0062294009511	-2.25419	Up

			5296	2195	1835	1501	
TONKS_TARGETS_OF_RUNX1_RUNX1T1_FUSION_HSC_DN	0.0867976943729766	0.00838470084361125	3.03294466970508	0.00265064599771635	0.00624385567659407	-2.256634725	Up
WP_SEROTONIN_RECEPTOR_2_AND_ELKSRFGATA4_SIGNALING	0.0989612836226908	0.000628533258411711	3.0319291407415	0.00265928932587441	0.00625874984193189	-2.259583472	Up
WP_BLOOD_CLOTTING_CASCADE	0.148949222202881	-0.002285189	3.03111352429485	0.00266624991197529	0.00627109253642692	-2.261951073	Up
HOEGERKORP_CD44_TARGETS_DIRECT_DN	0.12162815562032	0.0051485110938849	3.03014073941217	0.00267457368498153	0.00628828744798497	-2.26477413	Up
REACTOME_INTERACTION_WITH_CUMULUS_CELLS_AND_THE_ZONA_PELLUCIDA	0.142957896611499	-0.008803135	3.02939121644336	0.00268100336393053	0.00629863280844693	-2.266948694	Up
MIKKELSEN_ES_ICP_WITH_H3K4ME3_AND_H3K27ME3	0.104141721349954	-0.002658274	3.0259847107286	0.00271040493480146	0.00635808141735173	-2.27682553	Up
COULOUARN_TEMPORAL_TGFB1_SIGNATURE_DN	0.07422974258974	0.00129626120763799	3.02386146487587	0.00272888022763886	0.00638934725498092	-2.282976418	Up
REACTOME_NOREPINEPHRINE_NEUROTRANSMITTER_RELEASE_CYCLE	0.133599256378944	-0.001362227	3.0203830429668	0.00275939764865923	0.00645617235059459	-2.293044426	Up
WP_FOCAL_ADHESIONPI3KAKTMTORSIGNALING_PAT	0.0672595808258428	-0.0011568	3.0196	0.002765	0.006468	-2.2	Up

HWAY		65	6082154269	77311564931	40758433882	95133478	
KANG_IMMORTALIZED_BY_TERT_DN	0.0742785305849702	-0.003745948	3.01648568417351	0.00279396267430964	0.00652695759105756	-2.304312133	Up
WP_OVARIAN_INFERTILITY_GENES	0.0850420087243037	-6.22E-05	3.01543717096974	0.00280332939071062	0.00654391332385889	-2.30734118	Up
WP_CANNABINOID_RECEPTOR_SIGNALING	0.103746055328943	-0.003847739	3.01180404980029	0.00283600874759421	0.00661522220831164	-2.317829271	Up
REACTOME_DEGRADATION_OF_THE_EXTRACELLULAR_MATRIX	0.106179723447775	-0.003227375	3.01162814456818	0.00283759982851235	0.00661644708323672	-2.318336774	Up
NAISHIRO_CTNNB1_TARGETS_WITH_LEF1_MOTIF	0.156383493030194	-0.001858459	3.01041668859785	0.00284857984644364	0.00663955505327663	-2.321831182	Up
TAKEDA_TARGETS_OF_NUP98_HOXA9_FUSION_6HR_DN	0.0807197810433847	0.00302395199372995	3.00995806870228	0.00285274670979168	0.00664677133171057	-2.323153714	Up
WP_G_PROTEIN_SIGNALING_PATHWAYS	0.061911699067596	0.000639822642410265	3.0094568548566	0.0028573069651552	0.00665489843629206	-2.32459886	Up
SHIN_B_CELL_LYMPHOMA_CLUSTER_9	0.151299312309415	-0.008504607	3.0089770206538	0.00286167896778602	0.00666258115268112	-2.32598215	Up

MORI_EMU_MYC_LYMPHOMA_BY_ONSET_TIME_DN	0.118215427891816	0.0112730715168354	3.00356999617634	0.0029113712099395	0.00677065608845803	-2.341555524	Up
WP_ALPHA_6_BETA_4_SIGNALING_PATHWAY	0.0991454331772824	0.00367010167939478	3.00234231161626	0.00292276375879907	0.0067946047381 52	-2.345087862	Up
PID_SMAD2_3NUCLEAR_PATHWAY	0.071537705012392	0.00526667258243029	3.00080773556623	0.00293706168848319	0.00682528712108391	-2.349501298	Up
REACTOME_ADENYLATE_CYCLASE_ACTIVATING_PATHWAY	0.163427792320322	-0.004824993	3.00008679432286	0.00294380096353655	0.00683838794186803	-2.351573994	Up
SENESE_HDAC1_TARGETS_DN	0.0470126322923308	-0.000848865	2.99580446369847	0.00298412496482192	0.00692428547912136	-2.363876018	Up
REACTOME_TFAP2A_ACTS_AS_A_TRANSCRIPTIONAL_REPRESSOR_DURING_RETINOIC_ACID_INDUCED_CELL_DIFFERENTIATION	0.194899406301496	0.0187196056603201	2.99259963519074	0.00301463359797534	0.00698985085641874	-2.373071892	Up
SPIRA_SMOKERS_LUNG_CANCER_DN	0.23001657201468	-0.004193145	2.99241168045373	0.00301643169840782	0.00699140834653374	-2.373610919	Up
WP_RANKLRANK_RECEPTOR_ACTIVATOR_OF_NFKB_LIGAND_SIGNALING_PATHWAY	0.106278121252885	0.00616736056097922	2.99126235713812	0.00302744830549312	0.00701432311765426	-2.376906326	Up
WP_GLYCOLYSIS_AND_GLUCONEOGENESIS	0.0969982100134848	0.0035492081218554	2.9905850397	0.0030339578618	0.0070267822568	-2.37884	Up

		1	7771	1773	2934	7814	
REACTOME_LEISHMANIA_INFECTION	0.0802630753337149	0.00032032298293547	2.98957170288681	0.00304372080113428	0.00704676427177936	-2.381751712	Up
REACTOME_LDL_CLEARANCE	0.122626036696087	0.00124482078272507	2.98446187813374	0.00309339146278663	0.0071537558902819	-2.396380783	Up
CEBALLOS_TARGETS_OF_TP53_AND_MYC_DN	0.111571045556873	-0.004577982	2.98429621362722	0.00309501419097023	0.0071548428615837	-2.396854677	Up
MCCLUNG_CREB1_TARGETS_DN	0.0720270040746159	0.000523954973590597	2.98346586249516	0.00310315946700021	0.00717100179138879	-2.399229576	Up
KEGG_GLYCOSAMINOGLYCAN_BIOSYNTHESIS_HEPARAN_SULFATE	0.0915097802256736	-7.93E-06	2.9794913629375	0.00314241987973242	0.00725632447674819	-2.410588521	Up
IVANOVA_HEMATOPOIESIS_STEM_CELL	0.0464096663844856	0.000483541385632842	2.9793140730274	0.00314418172373502	0.00725769280744637	-2.411094877	Up
WP_EBV_LMP1_SIGNALING	0.0981154793811447	-0.003398829	2.9791011410589	0.00314629896243645	0.00725988017094537	-2.411702993	Up
WP_DIFFERENTIATION_PATHWAY	0.114543756645888	0.00122620494146733	2.97868211411422	0.00315046927405139	0.00726680148050427	-2.41289958	Up
REACTOME_EPHRIN_SIGNALING	0.143850544214968	0.0108436	2.9774	0.003162	0.007292	-2.4	Up

		476919609	2673075609	9936572183	97980325185	16483548	
MARTIN_NFKB_TARGETS_DN	0.159453396935953	-0.008654158	2.9772808504221	0.00316445199363842	0.00729363294634744	-2.416899927	Up
REACTOME_VLDL_CLEARANCE	0.1833165729854	0.0116517468806029	2.97484550463967	0.00318888883874848	0.00734722829328575	-2.423848189	Up
WP_MIRNA_BIOGENESIS	0.176438960023909	0.0110465242125923	2.97443283552383	0.00319304675156761	0.00735407836251583	-2.425025043	Up
GOUYER_TUMOR_INVASIVENESS	0.151752428734275	0.0051460949749652	2.9737019411611	0.00320042318554735	0.00736833335040518	-2.42710904	Up
STEIN_ESTROGEN_RESPONSE_NOT_VIA_ESRRA	0.110901832963311	0.00165336662354214	2.9732714785595	0.00320477486260721	0.00737336886608357	-2.428336192	Up
KEGG_INSULIN_SIGNALING_PATHWAY	0.0793449127228305	0.00444810187488037	2.97097939567742	0.0032280376473488	0.00742364938017562	-2.434867601	Up
PID_ERBB4_PATHWAY	0.0987374539429911	0.00398284117959827	2.96983072634523	0.00323975380632184	0.00744783402808876	-2.438139017	Up
VERRECCHIA_EARLY_RESPONSE_TO_TGFB1	0.116052734053269	0.00495264690168616	2.96775313455235	0.00326104375187926	0.00749122818945765	-2.444052996	Up

BIOCARTA_RB_PATHWAY	0.130065158000663	0.00232319948220052	2.96665274778484	0.00327237171104359	0.00751446955621442	-2.447183735	Up
PID_HDAC_CLASSII_PATHWAY	0.0836771877655465	0.00468620028346896	2.96419682251713	0.00329778439396836	0.00756752664337166	-2.454167226	Up
BLALOCK_ALZHEIMERS_DISEASE_INCIPIENT_UP	0.0597201572055956	0.00391637699634434	2.96412373232858	0.00329854345654396	0.00756752664337166	-2.454374977	Up
WP_NOTCH_SIGNALING_PATHWAY_NETPATH	0.0965344578290829	0.0109662525289768	2.96406684292533	0.00329913438004893	0.00756752664337166	-2.454536675	Up
BENPORATH_SUZ12_TARGETS	0.079036615209436	-0.002895498	2.96375591931807	0.00330236572931956	0.0075704086995373	-2.455420372	Up
REACTOME_REGULATION_OF_TP53_ACTIVITY_THROUGH_METHYLATION	0.104399695064959	0.00630335307049371	2.96366778707064	0.00330328219222487	0.0075704086995373	-2.455670843	Up
BIOCARTA_CARM1_PATHWAY	0.15309926792892	0.00234530299266099	2.96359398589791	0.00330404980941623	0.0075704086995373	-2.455880579	Up
BIDUS_METASTASIS_DN	0.0674324184331808	0.00441319351061389	2.96175954265228	0.00332318270414595	0.00761072757821602	-2.461092335	Up
CLASPER_LYMPHATIC_VESSELS_DURING_METASTASIS_UP	0.132169483197879	-0.004382733	2.9594623238	0.0033472853166	0.0076553426529	-2.46761	Up

			5932	0345	6891	4594	
WP_TGFBETA_RECEPTOR_SIGNALLING_IN_SKELETAL_DYSPLASIAS	0.0843522212877791	-0.000801595	2.95843414038636	0.00335812486583047	0.0076773042512743	-2.470532273	Up
WP_CELL_MIGRATION_AND_INVASION_THROUGH_P75NTR	0.122518999676092	0.00314400563920259	2.95811118599101	0.00336153621554558	0.00768227367079949	-2.471448525	Up
REACTOME_DEFECTIVE_F9_ACTIVATION	0.208503490582154	-0.010428261	2.95539302857471	0.00339037393357193	0.00774532609704857	-2.479156476	Up
CREIGHTON_AKT1_SIGNALING_VIA_MTOR_UP	0.136163169773223	0.0056789398713392	2.95516326171087	0.00339282194743604	0.00774806689762159	-2.479807727	Up
VANTVEER_BREAST_CANCER_ESR1_UP	0.0583495292245666	0.00240786218178148	2.95445303336258	0.00340039919445412	0.00776251482161704	-2.481820498	Up
MIKKELSEN_DEDIFFERENTIATED_STATE_UP	0.154842689688431	-0.00356117	2.95315444580243	0.00341429356224648	0.00778564296137543	-2.485499492	Up
PEDRIOLI_MIR31_TARGETS_UP	0.0472616501999189	-0.00228117	2.9524929097969	0.00342139169381399	0.00779610067676339	-2.487373089	Up
KEGG_MELANOMA	0.078978275857047	-0.001866906	2.95218848935801	0.0034246625915327	0.00780069016720861	-2.488235133	Up
VALK_AML_CLUSTER_15	0.0906289348810185	-0.0016498	2.9514	0.003432	0.007815	-2.4	Up

		83	5672526499	53686811033	75801187117	9030697	
REACTOME_SIGNALING_BY_RECEPTOR_TYROSINE_KINASES	0.0603332069198061	0.00118405297940035	2.94409138862841	0.00351272063394943	0.00797784741124189	-2.511133536	Up
ABRAHAM_ALPC_VS_MULTIPLE_MYELOMA_UP	0.109877680865419	0.0063691014546832	2.9430294593887	0.00352442178145022	0.00800149451260479	-2.514132267	Up
WP_MONOAMINE_TRANSPORT	0.116254182680894	-0.002537005	2.93812149403057	0.00357896609002065	0.00811249727811185	-2.527978443	Up
TESAR_ALK_TARGETS_EPISC_4D_UP	0.176596847154048	-0.011766999	2.93804250773356	0.00357985017637319	0.00811249727811185	-2.528201099	Up
ACOSTA_PROLIFERATION_INDEPENDENT_MYC_TARGETS_UP	0.104073439063689	0.00420325873453499	2.93668754709343	0.00359504725943574	0.00813788115847991	-2.532019748	Up
KEGG_NON_SMALL_CELL_LUNG_CANCER	0.0993430095970483	0.00568637193138489	2.93667353756453	0.00359520469639594	0.00813788115847991	-2.532059222	Up
REACTOME_SUMOYLATION_OF_TRANSCRIPTION_FACTORS	0.0960979301052209	0.000467870187684173	2.93602271827562	0.00360252545843017	0.00814603844097489	-2.533892811	Up
BIOCARTA_NO2IL12_PATHWAY	0.158546322797717	-0.011626784	2.93517716867384	0.00361205701052852	0.00816461684790623	-2.536274456	Up

PID_CD40_PATHWAY	0.0911316140899454	0.00541168712418251	2.93081391136961	0.0036616098487 3298	0.00826759269955824	-2.548554126	Up
REACTOME_ASSEMBLY_OF_COLLAGEN_FIBRILS_AND_OTHER_MULTIMERIC_STRUCTURES	0.10891246713852	-0.006863011	2.92620863528921	0.00371458474221185	0.00837467996952826	-2.561496307	Up
REACTOME_DEFECTIVE_CHST3_CAUSES_SEDCJD	0.181137138605855	-0.009730772	2.92610511344076	0.00371578357275347	0.00837467996952826	-2.561787014	Up
BIOCARTA_RECK_PATHWAY	0.180777018500502	0.00234163911649569	2.92592855817261	0.00371782897969491	0.00837624844898959	-2.562282789	Up
WP_KISSPEPTINKISSPEPTIN_RECEPTOR_SYSTEM_IN_THE_OVARY	0.10663712399326	0.0054439353425921	2.92576546333725	0.00371971936075221	0.00837746664448076	-2.562740742	Up
YEMELYANOV_GR_TARGETS_DN	0.129297008935379	0.00119271664532791	2.92311724294074	0.00375053724316879	0.00844074861071381	-2.570173304	Up
SIG_IL4RECEPTOR_IN_B_LYPHOCYTES	0.118807365573539	0.0118840276252316	2.92250188446765	0.00375773162210497	0.00845387465690669	-2.571899479	Up
REACTOME_CHROMATIN_MODIFYING_ENZYMES	0.0964024731891316	0.0112843052710827	2.9221381798003	0.00376198975242789	0.00845732357599416	-2.572919566	Up
REACTOME_CALCINEURIN_ACTIVATES_NFAT	0.144181918963149	0.0133390922723915	2.9213867705	0.0037708009633	0.0084740628456	-2.57502	Up

			5742	8767	7244	6676	
GHANDHI_BYSTANDER_IRRADIATION_DN	0.13933083434392	-0.003143591	2.9189174027 6966	0.0037998903602 5253	0.0085332559573 399	-2.58194 7715	Up
NIKOLSKY_BREAST_CANCER_1Q21_AMPLICON	0.109835520510727	-0.009474258	2.9183343564 7449	0.0038067885550 1795	0.0085456551757 6724	-2.58358 105	Up
REACTOME_GPCR_LIGAND_BINDING	0.0831816195707631	-0.002477528	2.9180319267 0004	0.0038103711964 7065	0.0085506052120 3663	-2.58442 815	Up
HAHTOLA_MYCOSIS_FUNGOIDES_DN	0.139333133904015	0.0052047828002365	2.9172945433 1658	0.0038191192970 6284	0.0085671389508 0198	-2.58649 3202	Up
REACTOME_CD209_DC_SIGN_SIGNALING	0.130626356291432	0.0106715822642254	2.9164929545 2972	0.0038286499227 2407	0.0085854154878 4259	-2.58873 7505	Up
AMIT_EGF_RESPONSE_120_HELA	0.0816924368137872	0.000312930574160641	2.9136734021 161	0.0038623463406 5196	0.0086516000492 3374	-2.59662 7136	Up
REACTOME_REDUCTION_OF_CYTOSOLIC_CA_LEVELS	0.124124919866094	-0.003889177	2.9123632124 3263	0.0038780963853 96	0.0086774853151 2365	-2.60029 0849	Up
GOERING_BLOOD_HDL_CHOLESTEROL_QTL_TRANS	0.11718536604034	0.00283917523601327	2.9116161768 2008	0.0038871028813 3735	0.0086913716082 352	-2.60237 9108	Up
WP_MATRIX_METALLOPROTEINASES	0.142747110421082	-0.0032022	2.9095	0.003912	0.008744	-2.6	Up

		98	5292807641	07738382943	06349349273	08144086	
WP_INFLAMMATORY_RESPONSE_PATHWAY	0.0896361773637955	0.00201647008805887	2.90931086993027	0.00391501694942999	0.00874748387513029	-2.608820175	Up
AMIT_EGF_RESPONSE_240_HELA	0.071122663375936	0.00335701927343012	2.90751171882961	0.00393692921418336	0.00879327802534585	-2.613843703	Up
SA_PTEN_PATHWAY	0.123435656371577	0.0130510570139033	2.90487600051984	0.00396923245007343	0.00886223950273588	-2.621197789	Up
DARWICHE_SQUAMOUS_CELL_CARCINOMA_DN	0.0422406898785626	0.00237032600770207	2.90235266738509	0.00400038483547627	0.00892858276655922	-2.628232426	Up
WP_STRIATED_MUSCLE_CONTRACTION_PATHWAY	0.10986333825879	-0.006476993	2.9011610483821	0.00401517368379337	0.00895836917875824	-2.631552466	Up
REACTOME_RECYCLING_OF_BILE_ACIDS_AND_SALTS	0.126320574505869	-0.004206054	2.90035974978547	0.00402514641773891	0.00897739267513669	-2.63378429	Up
SENESE_HDAC3_TARGETS_DN	0.0372102791348748	0.00102913984506465	2.8993046304967	0.0040383126152801	0.00900352241488634	-2.636722185	Up
WP_IRON_METABOLISM_IN_PLACENTA	0.127196347554114	-0.005028415	2.89558103795041	0.00408509180504632	0.00910454751666877	-2.647082198	Up

DAVICIONI_MOLECULAR_ARMS_VS_ERMS_DN	0.0785978140198995	-0.001994546	2.89540238419943	0.00408734859910609	0.00910630752141116	-2.647578945	Up
BIOCARTA_HDAC_PATHWAY	0.103559546853193	0.00420226857155826	2.89449092299044	0.00409888009782638	0.00912872219849599	-2.650112815	Up
REACTOME_CLASS_B_2_SECRETIN_FAMILY_RECEPTORS_	0.0888165656393839	-0.005384958	2.88709288286363	0.00419358154003655	0.00932959161971573	-2.670651659	Up
CHEN_LUNG_CANCER_SURVIVAL	0.10699925033077	0.00795229257854436	2.88476786675169	0.00422375335910642	0.0093933490146806	-2.677096282	Up
KEGG_GLYCOSPHINGOLIPID_BIOSYNTHESIS_GANGLIO_SERIES	0.100661867197635	0.00151859701337142	2.88411190806068	0.00423230149098339	0.00940898830749782	-2.678913625	Up
JECHLINGER_EPITHELIAL_TO_MESENCHYMAL_TRANSITION_DN	0.0925797119849499	0.00354849591491766	2.88391996820043	0.0042348057388401	0.00941118482670265	-2.679445324	Up
PRAMOONJAGO_SOX4_TARGETS_DN	0.116936809347811	-0.000740717	2.88105892292531	0.00427229444954326	0.00948431031770924	-2.687366847	Up
XU_GH1_AUTOCRINE_TARGETS_DN	0.0440414408975969	-0.000155431	2.88053594541364	0.00427917972435211	0.00949619898071274	-2.688814042	Up
BENPORATH_ES_2	0.0841154190025769	-0.005500788	2.8792121725	0.0042966531288	0.0095247592752	-2.69247	Up

			8894	4383	6201	611	
ICHIBA_GRAFT_VERSUS_HOST_DISEASE_35D_DN	0.0655814266020202	-0.0036751 92	2.8770 075177 1256	0.004325 8983392 8619	0.009586 1660092 6434	-2.6 9857 1522	Up
REACTOME_ACETYLCHOLINE_NEUROTRANSMITTER_ RELEASE_CYCLE	0.126202597310843	-0.0047703 27	2.8760 225041 5545	0.004339 0233020 006	0.009600 2619981 7424	-2.7 0129 3459	Up
WP_SUPRESSION_OF_HMGB1_MEDIATED_INFLAMMAT ION_BY_THBD	0.1338048799061	-0.0044276 19	2.8750 114966 9768	0.004352 5323616 1072	0.009624 5701348 4778	-2.7 0408 6314	Up
REACTOME_OLFACTORY_SIGNALING_PATHWAY	0.11020839483813	-0.0020805 8	2.8731 705252 7033	0.004377 2298776 8162	0.009675 7357018 4111	-2.7 0916 9526	Up
TESAR_ALK_AND_JAK_TARGETS_MOUSE_ES_D4_UP	0.181833924849827	-0.0148722 09	2.8725 785083 5095	0.004385 1991667 5045	0.009689 9007575 721	-2.7 1080 3526	Up
DING_LUNG_CANCER_BY_MUTATION_RATE	0.10905924417826	-0.0034949 79	2.8702 529191 503	0.004416 6326602 2917	0.009752 4151270 1618	-2.7 1721 9217	Up
KEGG_VEGF_SIGNALING_PATHWAY	0.095399726336283	0.0056549 428489299 7	2.8694 100735 2203	0.004428 0754564 995	0.009770 7303087 424	-2.7 1954 3199	Up
MYLLYKANGAS_AMPLIFICATION_HOT_SPOT_23	0.120773735723808	0.0126474 533957628	2.8681 687644 4375	0.004444 9771374 7281	0.009804 5391230 6102	-2.7 2296 4695	Up
KEGG_PRION_DISEASES	0.0802362694889153	0.0013091	2.8678	0.004449	0.009810	-2.7	Up

		5563489385	6259941585	15491098695	26803996304	23808383	
VART_KSHV_INFECTION_ANGIOGENIC_MARKERS_UP	0.081835235886141	-0.001257804	2.8651962753753	0.00448568974523259	0.00988095684642709	-2.731152284	Up
BIOCARTA_PGC1A_PATHWAY	0.108593335146151	0.00397840979004124	2.86517448619218	0.0044859894301968	0.00988095684642709	-2.731212272	Up
SMID_BREAST_CANCER_RELAPSE_IN_BRAIN_DN	0.0544344047355928	-0.00139664	2.8641656811663	0.00449988436018294	0.00990804619498244	-2.733989143	Up
REACTOME_FGFR2C_LIGAND_BINDING_AND_ACTIVATION	0.139808355390861	-0.010345049	2.86148827207175	0.00453695236623291	0.00998258182814876	-2.741354604	Up
ZHAN_EARLY_DIFFERENTIATION_GENES_DN	0.111142402758224	0.00448455154474581	2.85959341301382	0.00456335400015326	0.0100300064727165	-2.746563377	Up
KEGG_ARGININE_AND_PROLINE_METABOLISM	0.068083235171591	-4.79E-05	2.85910992412708	0.00457011296104593	0.010041306601491	-2.747891917	Up
MCDOWELL_ACUTE_LUNG_INJURY_DN	0.0751639164373846	-0.004807572	2.85696641739239	0.00460018817170902	0.0101038103261847	-2.75377934	Up
BIOCARTA_SLRP_PATHWAY	0.173537591552579	-0.003136702	2.85632661529887	0.00460919999486502	0.0101200227690581	-2.755535834	Up

FIGUEROA_AML_METHYLATION_CLUSTER_5_DN	0.0713222011661129	-0.000680011	2.85452021067076	0.00463473062477706	0.0101724798401666	-2.760493084	Up
MIKKELSEN_ES_HCP_WITH_H3K27ME3	0.118683124787024	-0.010743066	2.85425429338501	0.00463849979074565	0.0101771538356869	-2.761222582	Up
MIDORIKAWA_AMPLIFIED_IN_LIVER_CANCER	0.0588531010322076	0.00243070293056184	2.85311622830642	0.00465466250591691	0.0102054009799457	-2.764343941	Up
GAUSSMANN_MLL_AF4_FUSION_TARGETS_B_UP	0.0878379373059724	0.00377466808017269	2.84657958950056	0.0047484923286936	0.0104037740501946	-2.782249194	Up
LIU_TARGETS_OF_VMYB_VS_CMYB_DN	0.0715544328405031	0.00181978510205121	2.84433750782405	0.00478107049894604	0.0104603823006549	-2.788381821	Up
WP_RAS_SIGNALING	0.0667769776122207	0.00485157430342725	2.8422490920955	0.00481159865338361	0.0105197579575738	-2.794090036	Up
MOOTHA_TCA	0.172664377999095	0.0060706621989348	2.84124963419018	0.00482627123337796	0.0105379562100731	-2.796820432	Up
NAKAMURA_ADIPOGENESIS_LATE_DN	0.0840119512434507	-6.09E-06	2.84032363631741	0.00483990169384154	0.0105630343929938	-2.799349336	Up
SCHUETZ_BREAST_CANCER_DUCTAL_INVASIVE_DN	0.0609197831611587	-0.001554481	2.8384923722	0.0048669606018	0.0106146255993	-2.80434	Up

			229	7653	843	8237	
REACTOME_MAP2K_AND_MAPK_ACTIVATION	0.105401419643575	0.00203416655042859	2.83824439718471	0.00487063526027288	0.0106176577301772	-2.805024914	Up
LINDGREN_BLADDER_CANCER_CLUSTER_1_DN	0.0590271938809543	-0.001265646	2.83813851794635	0.0048722050175949	0.0106176577301772	-2.805313822	Up
GRATIAS_RETINOBLASTOMA_16Q24	0.112437814439865	0.00631579934387749	2.83805236456537	0.00487348266042772	0.0106176577301772	-2.805548897	Up
REACTOME_BETA_OXIDATION_OF_BUTANOYL_COA_TO_ACETYL_COA	0.205167284696445	0.022868540768987	2.83653048696697	0.00489610219020908	0.0106594550314373	-2.809700329	Up
REACTOME_RETROGRADE_NEUROTROPHIN_SIGNALLING	0.143361131218274	0.0038616807269138	2.83640908085493	0.00489791074415635	0.0106596535725731	-2.810031415	Up
SCHAEFFER_PROSTATE_DEVELOPMENT_AND_CANCER_BOX2_UP	0.142280920694302	-0.010133754	2.83454681711295	0.00492572862428969	0.0107164379849163	-2.815108303	Up
REACTOME_RESPONSE_TO_ELEVATED_PLATELET_CYTOSOLIC_CA2_	0.100687485182466	-0.002721621	2.82732963302699	0.00503489908556654	0.0109347860826142	-2.834754021	Up
CHIANG_LIVER_CANCER_SUBCLASS_CTNNB1_UP	0.049253233191029	-0.002141882	2.82679484914953	0.00504307534860964	0.0109487123780413	-2.836207863	Up
HOFFMANN_IMMATURE_TO_MATURE_B_LYMPHOCYT	0.0798700590458338	0.0022056	2.8251	0.005068	0.010991	-2.8	Up

E_UP		5717635404	57588	18237441225	6869315083	40657242	
BIOCARTA_MONOCYTE_PATHWAY	0.147186531087686	0.00362700022235266	2.82449588169043	0.00507836169080 7	0.0110099165263147	-2.842454789	Up
WP_VITAMIN_A_AND_CAROTENOID_METABOLISM	0.109380367926589	-0.002849742	2.82359111743796	0.00509231014008896	0.0110324499265383	-2.844911967	Up
VERHAAK_GLIOBLASTOMA_PRONEURAL	0.0493921104833758	-0.000183178	2.82226297459701	0.00511284874926949	0.0110730817120432	-2.848517621	Up
STAMBOLSKY_BOUND_BY_MUTATED_TP53	0.101427249293869	0.0038415312216407	2.8215433730468	0.00512400819505968	0.0110933794442746	-2.850470533	Up
RAMJAUN_APOPTOSIS_BY_TGFB1_VIA_SMAD4_DN	0.115604770691349	-0.007255589	2.8211012806154	0.00513087505235226	0.0111043728904988	-2.851670085	Up
WP_BIOGENIC_AMINE_SYNTHESIS	0.134257965970141	-0.000965117	2.81933692609338	0.00515836347117963	0.0111560843434188	-2.856455634	Up
COLLER_MYC_TARGETS_DN	0.190127221681048	-0.012669141	2.81891600876997	0.00516494104646914	0.0111664190440383	-2.857596893	Up
RIZKI_TUMOR_INVASIVENESS_2D_DN	0.0631258514349465	0.00166265949974643	2.81800878974017	0.00517914386209121	0.0111932263064067	-2.860056143	Up

DARWICHE_PAPILLOMA_RISK_HIGH_DN	0.0416415202455134	0.0024898693988919	2.81668586606419	0.00519991827175324	0.0112303036578888	-2.863640929	Up
FRASOR_TAMOXIFEN_RESPONSE_DN	0.122671598134758	0.00691944427710985	2.8165494023823	0.00520206551788226	0.0112310332763462	-2.86401062	Up
BROWNE_HCMV_INFECTION_20HR_UP	0.0431580054738166	0.00351051900854064	2.81467790292734	0.0052315948068707 8	0.0112851877886415	-2.869078961	Up
REACTOME_UPTAKE_AND_FUNCTION_OF_ANTHRAX_TOXINS	0.148444908529309	0.0120208325825612	2.80988210100657	0.0053079611939883 7	0.0114437357176401	-2.8820523	Up
MEISSNER_NPC_HCP_WITH_H3K27ME3	0.103217099710157	-0.006778683	2.80891589178817	0.0053234686861803 4	0.011469201712989	-2.884663509	Up
BIOCARTA_DC_PATHWAY	0.0909514863020099	0.000802739450296106	2.8030872573085	0.0054178930547981 1	0.0116604931314604	-2.900397579	Up
SU_PLACENTA	0.101894025905039	-0.002345363	2.79844606675531	0.0054941651522908 3	0.0118082697715614	-2.91290414	Up
REACTOME_GOLGI_TO_ER_RETROGRADE_TRANSPORT	0.0611862713836648	0.000886482211040208	2.797955293	0.0055022870978298 9	0.0118186819631936	-2.914225477	Up
BIOCARTA_EGF_PATHWAY	0.111651146092711	0.0114673274829935	2.7979232403	0.0055028179271	0.0118186819631	-2.91431	Up

			1611	1928	936	1767	
REACTOME_IRS_ACTIVATION	0.178363416208537	0.000162872554775803	2.79708347032768	0.0055167420009829	0.011841783540066	-2.916572196	Up
WP_ADIPOGENESIS	0.0636566144572634	0.00014375201994859	2.79453090709066	0.00555926208394405	0.0119275284324372	-2.923439054	Up
SASSON_RESPONSE_TO_GONADOTROPHINS_DN	0.0766181981809774	0.00449009851033181	2.79414742714387	0.00556567564189963	0.0119371626500591	-2.924470173	Up
BYSTRYKH_HEMATOPOIESIS_STEM_CELL_RUNX1	0.158075476565455	-0.007924559	2.79248789000161	0.00559350822858959	0.0119885723670081	-2.928930874	Up
REACTOME_FGFR2_LIGAND_BINDING_AND_ACTIVATION	0.112086997653293	-0.003684039	2.7906940419809	0.00562373533089269	0.0120491975142737	-2.933749777	Up
LIEN_BREAST_CARCINOMA_METAPLASTIC	0.107917615781733	-0.006046021	2.78746954224263	0.00567844228386404	0.0121496350416905	-2.942404553	Up
REACTOME_MRNA_EDITING	0.140710278992169	-0.010868636	2.7868703192999	0.00568866173472186	0.0121643932592413	-2.944011867	Up
CHESLER_BRAIN_D6MIT150_QTL_CIS	0.149345769415377	0.00493453590408797	2.78683530004399	0.00568925948631826	0.0121643932592413	-2.9441 0579	Up
LIM_MAMMARY_LUMINAL_MATURE_DN	0.0562241691736068	0.0021817	2.7857	0.005707	0.012195	-2.9	Up

		1809343031	5311387457	75966644546	5470738819	47007708	
DORN_ADENOVIRUS_INFECTION_12HR_DN	0.088681711775208	0.0106233160520093	2.78550322816921	0.00571203925525885	0.0122004912792125	-2.947677634	Up
RAMPON_ENRICHED_LEARNING_ENVIRONMENT_EARLY_DN	0.13384497324961	0.00975846789122588	2.78387486872865	0.00573999816843319	0.0122475657034943	-2.952041757	Up
ANASTASSIOU_MULTICANCER_INVASIVENESS_SIGNATURE	0.106170961132382	-0.005640016	2.78275942843266	0.00575922181222337	0.0122843607520517	-2.955029828	Up
MAINA_VHL_TARGETS_DN	0.0964075649843238	-0.004482869	2.78067024779124	0.00579538413518048	0.0123530045766021	-2.960623334	Up
BIOCARTA_EXTRINSIC_PATHWAY	0.154765264504681	-0.011427443	2.77719135195521	0.00585605848336212	0.0124737663027552	-2.9699 2881	Up
IWANAGA_CARCINOGENESIS_BY_KRAS_DN	0.0413626918517502	0.00317502661496346	2.77404074807698	0.00591150335430946	0.012574606346881	-2.978346657	Up
REACTOME_PHYSIOLOGICAL_FACTORS	0.128763590118725	-0.005527018	2.77237261687717	0.00594105161703414	0.0126244804474259	-2.982799946	Up
REACTOME_FORMATION_OF_FIBRIN_CLOT_CLOTTING_CASCADE_	0.116742853717923	-0.000242398	2.77192384879288	0.00594902363204711	0.0126370943477469	-2.983997557	Up

YAUCH_HEDGEHOG_SIGNALING_PARACRINE_UP	0.051988051666352	-0.00287065	2.77147554709196	0.00595699703763886	0.012649702570176	-2.985193741	Up
ACEVEDO_NORMAL_TISSUE_ADJACENT_TO_LIVER_TUMOR_DN	0.0656661654628644	-0.00010681	2.76891196204887	0.00600277872802893	0.0127338508424045	-2.992030528	Up
REACTOME_SUMOYLATION	0.0747259841930079	0.00541489778434014	2.76562260514491	0.00606198836087261	0.0128462826070114	-3.000794103	Up
WP_ESTROGEN_RECEPTOR_PATHWAY	0.134783236582004	0.00297889767674748	2.76342055724058	0.00610192107337315	0.0129264928677226	-3.006655341	Up
IZADPANAH_STEM_CELL_ADIPOSE_VS_BONE_DN	0.0663209783453763	-0.003044641	2.76221365519525	0.0061239083741631	0.0129686452672911	-3.009865905	Up
REACTOME_SYNDECAN_INTERACTIONS	0.108156256284457	-0.007518535	2.76198177293407	0.00612814100398031	0.0129731825415095	-3.010482599	Up
VANDESLUIS_NORMAL_EMBRYOS_DN	0.102513276556834	4.0167231849839e-05	2.75721624701287	0.00621571652203812	0.0131451286038469	-3.023145738	Up
ST_STAT3_PATHWAY	0.132045618403505	0.00529600327952932	2.75401904908892	0.006275104400707	0.0132571725715413	-3.03162986	Up
REACTOME_BETA_OXIDATION_OF_LAUROYL_COA_TO_DECANOYL_COA_COA	0.184958160916026	0.0201173781507556	2.7535875694	0.0062831583332	0.0132696712401	-3.03277	Up

			163	8872	235	4126	
SMID_BREAST_CANCER_LUMINAL_A_UP	0.0951110238240278	-0.00611152	2.75203088456544	0.00631229298802605	0.0133200737541465	-3.03690098	Up
LEE_AGING_NEOCORTEX_DN	0.0565495296961605	-0.003680196	2.75196857338448	0.0063134617342837	0.0133200737541465	-3.037066125	Up
BROWNE_HCMV_INFECTION_1HR_UP	0.0737585033401352	-0.00630189	2.75129599989029	0.00632608939982891	0.0133421803957656	-3.038848431	Up
REACTOME_ACETYLCHOLINE_BINDING_AND_DOWNSTREAM_EVENTS	0.131210717380631	-0.009840157	2.75002940950635	0.00634993188112984	0.0133879168431294	-3.042203752	Up
IIZUKA_LIVER_CANCER_PROGRESSION_G1_G2_DN	0.126074355691326	0.0116397200884199	2.74898330701382	0.00636968514079406	0.0134250036227194	-3.044973877	Up
WP_NETRINUNC5B_SIGNALING_PATHWAY	0.0914715677382358	0.00128778777404778	2.74863753343837	0.00637622650078358	0.0134342287475776	-3.045889281	Up
REACTOME_DERMATAN_SULFATE_BIOSYNTHESIS	0.120661783971492	-0.006756285	2.74220827688107	0.00649896773956972	0.0136835457121809	-3.0628903	Up
REACTOME_REGULATION_OF_INSULIN_LIKE_GROWTH_FACTOR_IGF_TRANSPORT_AND_UPTAKE_BY_INSULIN_LIKE_GROWTH_FACTOR_BINDING_PROTEINS_IGFBPS_	0.0891105431912898	-0.004999954	2.73644983216681	0.0066107121937211	0.013914103284648	-3.078085483	Up

LIU_IL13_PRIMING_MODEL	0.127696875125273	0.00666445154729764	2.73178301674145	0.00670254432780229	0.0140970636861429	-3.090377926	Up
WP_GLYCOSAMINOGLYCAN_DEGRADATION	0.149868291182304	-0.004613658	2.7316866976818	0.0067044517482671	0.0140970636861429	-3.090631422	Up
WP_MTHFR_DEFICIENCY	0.0717771766226902	0.00393188513361579	2.73010312465087	0.0067358818920422	0.0141583538448716	-3.094797917	Up
REACTOME_GLI_PROTEINS_BIND_PROMOTERS_OF_HH_RESPONSIVE_GENES_TO_PROMOTE_TRANSCRIPTION	0.153141424833377	-0.007131142	2.72772741477239	0.00678328381808648	0.0142483393092598	-3.101044289	Up
LEE_DOUBLE_POLAR_THYMOCYTE	0.0827036530094881	0.00109225947795434	2.72572511916855	0.00682346894326788	0.014318212214626	-3.106304857	Up
BILBAN_B_CLL_LPL_UP	0.078502814189834	0.00739287251563946	2.72425350798546	0.00685314047170033	0.0143707577391365	-3.110168841	Up
REACTOME_SYNTHESIS_OF_12_EICOSATETRAENOIC_ACID_DERIVATIVES	0.182769567440061	-0.009950748	2.72135590461553	0.00691190464925022	0.0144793088619292	-3.117771251	Up
BIOCARTA_PYK2_PATHWAY	0.127493866017148	0.0144626429835192	2.72075921488698	0.00692406203893921	0.014496835822713	-3.119335828	Up
MEBARKI_HCC_PROGENITOR_WNT_DN_CTNNB1_DEPENDENT	0.0921821687695597	-0.001637186	2.7195971571	0.0069477940909	0.0145397700345	-3.12238	Up

			8414	648	983	1919	
REACTOME_VASOPRESSIN_LIKE_RECEPTORS	0.169927503492108	-0.019484169	2.71947652119476	0.0069502619760008	0.014540032384576	-3.122698071	Up
WP_IMATINIB_AND_CHRONIC_MYELOID_LEUKEMIA	0.106613703045982	0.00608997995671187	2.71760047895861	0.00698874272542642	0.0146156085231542	-3.127612915	Up
REACTOME_AMINO_ACID_CONJUGATION	0.142963630984472	-0.018911369	2.71585012105372	0.00702481884833085	0.014686106632398	-3.132195595	Up
BIOCARTA_FREE_PATHWAY	0.113740614179892	0.0043767463422274	2.71538223137638	0.00703449082504935	0.0147013752697243	-3.133420121	Up
WP_COMMON_PATHWAYS_UNDERLYING_DRUG_ADDICTION	0.0845036364165331	0.00259987134769666	2.7135664177959	0.00707214036740559	0.0147701128063548	-3.13817044	Up
WP_FGFR3_SIGNALLING_IN_CHONDROCYTE_PROLIFERATION_AND_TERMINAL_DIFFERENTIATION	0.0910232084183619	-0.00517551	2.71195181649574	0.00710577060697204	0.0148353576042635	-3.142391841	Up
WP_INSULIN_SIGNALING	0.0778142504647665	0.0100666958351757	2.70888406295664	0.00717006624655961	0.0149645599167436	-3.150405973	Up
PID_LPA4_PATHWAY	0.125102316048824	-0.005608017	2.70619387435703	0.00722688024756617	0.0150780657803843	-3.157426687	Up
REACTOME_HIGHLY_SODIUM_PERMEABLE_POSTSYN	0.157908819097408	-0.0078400	2.7059	0.007232	0.015084	-3.1	Up

APTIC_ACETYLCHOLINE_NICOTINIC_RECEPTORS		7	3045399501	46519064528	6476607309	58113792	
REACTOME_CD22_MEDIATED_BCR_REGULATION	0.198184304821007	0.0100244693841727	2.70357593428411	0.0072825580976 3516	0.0151738295105812	-3.164252502	Up
BIOCARTA_CDC25_PATHWAY	0.117105600939822	-0.006491798	2.7014556054203	0.00732793616812689	0.0152632549649542	-3.169776293	Up
REACTOME_NEGATIVE_REGULATION_OF_ACTIVITY_OF_TFAP2_AP_2_FAMILY_TRANSCRIPTION_FACTORS	0.119010904118461	-0.002294113	2.70061605555618	0.00734597411230131	0.015290853550433	-3.171962318	Up
REACTOME_FICOLINS_BIND_TO_REPETITIVE_CARBOHYDRATE_STRUCTURES_ON_THE_TARGET_CELL_SURFACE	0.189462718376789	-0.000554322	2.7006095842432	0.00734611330552461	0.015290853550433	-3.171979165	Up
REACTOME_LONG_TERM_POTENTIATION	0.100043184163123	-0.003140444	2.69965531432828	0.00736666501488332	0.0153245416974034	-3.17446311	Up
WP_B_CELL_RECEPTOR_SIGNALING_PATHWAY	0.0923399788442218	0.0096462647313526	2.69962884694687	0.00736723577010662	0.0153245416974034	-3.174531993	Up
MOTAMED_RESPONSE_TO_ANDROGEN_UP	0.206498291263772	-0.011822831	2.69940739090986	0.00737201291680902	0.0153293414320381	-3.175108314	Up
ZAIDI_OSTEOBLAST_TRANSCRIPTION_FACTORS	0.111767686820911	0.00850902722292177	2.69903235827336	0.00738010931872646	0.0153410376896635	-3.176084203	Up

SANSOM_APC_TARGETS_DN	0.0619504309802533	0.000341995102270715	2.69838698831418	0.00739406068129233	0.0153552349095712	-3.177763249	Up
WP_OXIDATION_BY_CYTOCHROME_P450	0.0876926379737969	-0.00061079	2.69820277225049	0.0073980473554707	0.0153577524867581	-3.17824245	Up
REACTOME_DISEASES_ASSOCIATED_WITH_O_GLYCOSYLATION_OF_PROTEINS	0.0900219873894161	-0.006222651	2.69388283043312	0.00749209396489836	0.0155322068270288	-3.18947102	Up
REACTOME_GLYCOSAMINOGLYCAN_METABOLISM	0.0785037530700225	-0.002463275	2.69333626822971	0.00750406931065868	0.0155425909226788	-3.190890451	Up
BIOCARTA_STAT3_PATHWAY	0.172732200290739	-0.005946749	2.69321596291813	0.00750670755318427	0.0155425909226788	-3.191202849	Up
REACTOME_ACETYLCHOLINE_REGULATES_INSULIN_SECRETION	0.129484691765866	0.0039951077189292	2.69319720449674	0.00750711899245866	0.0155425909226788	-3.191251558	Up
KORKOLA_CHORIOCARCINOMA	0.17527969400264	-0.008786349	2.68945975507583	0.00758950133437788	0.0157019930640867	-3.200949979	Up
PID_INTEGRIN3_PATHWAY	0.101702711474051	-0.005013571	2.68936051507447	0.00759169988485971	0.0157019930640867	-3.201207325	Up
PID_IL2_PI3K_PATHWAY	0.0824613168088887	0.0053603540833891	2.6881898255	0.0076176785542	0.0157452316970	-3.20424	Up

		8	5549	4222	301	2449	
REACTOME_REGULATION_OF_PTEN_GENE_TRANSCRIPTION	0.0623637027019089	0.00358566260440615	2.68747849749656	0.00763350267799769	0.0157726867917216	-3.206086023	Up
WP_CODEINE_AND_MORPHINE_METABOLISM	0.133243982022662	-0.00690339	2.68606305501292	0.00766507860413019	0.0158326598655029	-3.209753097	Up
REACTOME_NGF_STIMULATED_TRANSCRIPTION	0.0792392337340977	-0.0007828	2.6848781797506	0.00769160144878161	0.0158768773503783	-3.212821417	Up
REACTOME_NEUREXINS_AND_NEUROLIGINS	0.0881773712588683	-0.006774937	2.67855045195977	0.00783464953589146	0.0161560364349762	-3.229185779	Up
TESAR_ALK_TARGETS_HUMAN_ES_4D_DN	0.179655798169078	-0.01342896	2.67668537846835	0.00787726725238163	0.0162169810399777	-3.234002121	Up
TESAR_ALK_TARGETS_HUMAN_ES_5D_DN	0.179655798169078	-0.01342896	2.67668537846835	0.00787726725238163	0.0162169810399777	-3.234002121	Up
SCHAEFFER_PROSTATE_DEVELOPMENT_AND_CANCER_BOX6_UP	0.142067026038451	-0.006977151	2.67468763505879	0.00792314801384074	0.0163060277592538	-3.239157536	Up
WESTON_VEGFA_TARGETS_6HR	0.0935782238228422	-0.004417557	2.67357396844716	0.00794882913942796	0.0163426242028583	-3.242029899	Up
JOHANSSON_GLIOMAGENESIS_BY_PDGFB_DN	0.104448430503342	0.0064955	2.6721	0.007980	0.016392	-3.2	Up

		5659593631	9355665899	76541635132	9788077413	45588674	
REACTOME_CELL_DEATH_SIGNALLING_VIA_NRAGE_NRIF_AND_NADE	0.0753515732338157	0.00866665688264386	2.67217290511592	0.00798124407233675	0.0163929788077413	-3.245641902	Up
KEGG_TAURINE_AND_HYPOTAURINE_METABOLISM	0.121780673141175	-0.010876879	2.67038395819328	0.00802280603356356	0.0164674461145268	-3.250251273	Up
BAKER_HEMATOPOIESIS_STAT3_TARGETS	0.110954745480213	0.00484786800286983	2.66967865587717	0.00803924554700885	0.016495734581912	-3.252067739	Up
KEGG_ETHER_LIPID_METABOLISM	0.0851857978355174	0.00106728233050137	2.66927103440328	0.00804876039808118	0.0165098003274586	-3.253117336	Up
CUI_TCF21_TARGETS_UP	0.0909465942076342	-0.004473354	2.66055415952065	0.00825467404240575	0.0169098223700371	-3.275526278	Up
CHARAFE_BREAST_CANCER_BASAL_VS_MESENCHYMAL_UP	0.067789613413511	-0.001247211	2.660371191	0.00825904654067248	0.0169131975842805	-3.275995901	Up
REACTOME_INLB_MEDIATED_ENTRY_OF_LISTERIA_MONOCYTOGENES_INTO_HOST_CELL	0.111561549689704	0.0108159685942414	2.66015770147528	0.00826415102522026	0.0169180690677791	-3.276543819	Up
SCHAEFFER_PROSTATE_DEVELOPMENT_AND_CANCER_BOX2_DN	0.13582201291663	0.00667430336955281	2.65684482899155	0.00834372575474785	0.0170640875649819	-3.285040943	Up

GRAESSMANN_RESPONSE_TO_MC_AND_SERUM_DEPRIVATION_DN	0.0580614980176372	0.00240779722455366	2.65205814383652	0.00845991862229442	0.0172846329455502	-3.297300457	Up
BIOCARTA_AMI_PATHWAY	0.125402063388912	-0.012476492	2.65158275337403	0.00847153738893984	0.0173026760688218	-3.298516866	Up
REACTOME_CLASS_A_1_RHODOPSIN_LIKE_RECEPTORS_	0.0748011527485235	-0.00225812	2.64941326469104	0.00852474305036365	0.0173998948088152	-3.304065434	Up
PID_NFAT_3PATHWAY	0.0777937051551384	0.0102373713532903	2.64414525836544	0.00865519076882811	0.0176487414921538	-3.317520643	Up
BIOCARTA_NGF_PATHWAY	0.0934589080401801	0.00435886593344764	2.6416291559535	0.00871812584475086	0.0177709129732313	-3.323938134	Up
ZHANG_ADIPOGENESIS_BY_BMP7	0.111859341677088	0.00587211321260031	2.64152135328124	0.00872083146712784	0.0177709129732313	-3.324212962	Up
MIKKELSEN_MEF_HCP_WITH_H3_UNMETHYLATED	0.0795171567856253	-0.003829369	2.64000385172254	0.00875899773000292	0.0178369747080473	-3.328080496	Up
WANG_BARRETTS_ESOPHAGUS_UP	0.0812354488546819	-0.000574049	2.63878820975184	0.00878968018673974	0.0178935863952422	-3.331177181	Up
REACTOME_TP53_REGULATES_TRANSCRIPTION_OF_ADDITIONAL_CELL_CYCLE_GENES_WHOSE_EXACT_RO	0.119357537769051	0.0051208252568470	2.6370537011	0.0088336258540	0.0179771526806	-3.33559	Up

LE_IN_THE_P53_PATHWAY_REMAIN_UNCERTAIN		3	0974	2435	325	3263	
REACTOME_AKT_PHOSPHORYLATES_TARGETS_IN_THE_NUCLEUS	0.11728242742448	0.00435699355949786	2.63686809644976	0.00883834001974291	0.0179808510332823	-3.336065651	Up
REACTOME_LGI_ADAM_INTERACTIONS	0.153064597735159	-0.00689979	2.63492215410214	0.00888790114217295	0.0180757543871125	-3.341016429	Up
KEGG_HEDGEHOG_SIGNALING_PATHWAY	0.0764660775700665	-0.003811317	2.63439874137546	0.00890127444449203	0.0180970227569479	-3.342347479	Up
WP_STATIN_PATHWAY	0.0962981213532081	-0.006154162	2.63384047028144	0.00891555829857668	0.0181201278190129	-3.343766898	Up
ENK_UV_RESPONSE_EPIDERMIS_UP	0.0481153370447684	0.00103088338139493	2.63238291290752	0.00895294825031482	0.0181842113186204	-3.347471426	Up
REACTOME_CLEC7A_INFLAMMASOME_PATHWAY	0.14637815080593	0.0119453138525108	2.63190745080426	0.00896517544972479	0.0182030893086169	-3.34867944	Up
KEGG_GLYCOSAMINOGLYCAN_DEGRADATION	0.121406661172702	-0.004671184	2.63117954564707	0.00898392363897721	0.0182351909833655	-3.350528436	Up
CHEN_NEUROBLASTOMA_COPY_NUMBER_GAINS	0.0542823168017617	-0.0001512	2.62485277575266	0.0091483666958462	0.0185447132857993	-3.366578973	Up
REACTOME_SIGNALING_BY_NUCLEAR_RECEPTORS	0.0426900852889926	0.0036779	2.6244	0.009157	0.018557	-3.3	Up

		7481397683	90928	85287404183	7355300061	67495843	
WP_GLYCEROLIPIDS_AND_GLYCEROPHOSPHOLIPIDS	0.093603995753045	-0.008476081	2.62320610091087	0.00919160713694739	0.0186141290372047	-3.370750436	Up
NAKAMURA_LUNG_CANCER_DIFFERENTIATION_MARKERS	0.14012520269564	-0.016945478	2.62301191565693	0.00919671836566033	0.0186184053801871	-3.371242194	Up
VANDESLUIS_COMMD1_TARGETS_GROUP_3_UP	0.0714315929836419	-0.003288925	2.6190735294019	0.00930093412754118	0.0188048528109603	-3.381208369	Up
REACTOME_ADRENOCEPTORS	0.131751689867307	-0.004968576	2.6160618529169	0.00938134123290884	0.0189489049894778	-3.388819873	Up
LEE_BMP2_TARGETS_UP	0.0610945655698547	-0.000494536	2.61474961202242	0.00941657062788993	0.0190076923210773	-3.392133735	Up
GAZDA_DIAMOND_BLACKFAN_ANEMIA_ERYTHROID_DN	0.0362481114722137	0.00188051272409192	2.61400208556626	0.00943669228109321	0.0190421160561592	-3.394020791	Up
ROZANOV_MMP14_CORRELATED	0.12673879491182	0.00923095748774853	2.61364614026638	0.00944628703541128	0.0190490914973352	-3.394919158	Up
FLECHNER_PBL_KIDNEY_TRANSPLANT_REJECTED_VS_OK_DN	0.0657653530027607	0.00734655402349328	2.60306479988264	0.00973553961874017	0.019605607819876	-3.421572161	Up

ACEVEDO_METHYLATED_IN_LIVER_CANCER_DN	0.0449733420334518	-0.001064752	2.60306401223468	0.0097355614421811	0.019605607819876	-3.421574141	Up
PLASARI_TGFB1_TARGETS_10HR_UP	0.0731681629449598	-0.001705895	2.60208247824172	0.009762790991824	0.0196490414028053	-3.42404128	Up
SABATES_COLORECTAL_ADENOMA_DN	0.067399470269459	-0.001516707	2.60175412297107	0.00977191538925388	0.0196610304120255	-3.424866421	Up
WP_TRANSLATION_INHIBITORS_IN_CHRONICALLY_ACTIVATED_PDGFRA_CELLS	0.0731500497200211	0.00743302514230124	2.59939027072244	0.00983782837443182	0.0197778133508657	-3.430803744	Up
PID_ECADHERIN_NASCENT_AJ_PATHWAY	0.0810870207354651	0.00908760925219245	2.59660003799842	0.0099161427673036	0.0199253797852552	-3.437805403	Up
LEIN_LOCALIZED_TO_PROXIMAL_DENDRITES	0.0837223759021292	0.000238475951898031	2.59646678288776	0.00991989681501238	0.0199264723400589	-3.438139607	Up
DING_LUNG_CANCER_MUTATED_SIGNIFICANTLY	0.085110305027345	-0.002981533	2.59411851900766	0.00998626103327546	0.0200273739042135	-3.444026378	Up
BIOCARTA_GH_PATHWAY	0.10628373898702	0.00638566289934623	2.59365341680813	0.00999945234866	0.0200473516563736	-3.445191723	Up
ZHONG_SECRETOME_OF_LUNG_CANCER_AND_FIBROBLAST	0.0688119087804368	0.0013710549584279	2.5903315386	0.0100941226761	0.0202045209451	-3.45350	Up

		7	1954	674	697	9127	
HERNANDEZ_ABERRANT_MITOSIS_BY_DOCETACEL_2NM_DN	0.0891876115866072	-0.003115839	2.58849322831039	0.0101468569437129	0.0203035270953018	-3.458107573	Up
REACTOME_THE_FATTY_ACID_CYCLING_MODEL	0.140343890310232	-0.003861511	2.58829156739301	0.0101526568281794	0.0203085855406089	-3.458611829	Up
REACTOME_N_GLYCAN_TRIMMING_IN_THE_ER_AND_CALNEXIN_CALRETICULIN_CYCLE	0.0690504275345355	0.00413943783978867	2.58705962695368	0.0101881526303828	0.0203730229950985	-3.461691501	Up
ACOSTA_PROLIFERATION_INDEPENDENT_MYC_TARGETS_DN	0.0878224139430196	0.00353631785339857	2.58623937590639	0.010211848019392	0.0204138295189586	-3.463741236	Up
WP_DEREGULATION_OF_RAB_AND_RAB_EFFECTOR_GENES_IN_BLADDER_CANCER	0.104777486363885	0.00503731110677915	2.58558155047331	0.0102308868902172	0.0204387238260631	-3.465384636	Up
REACTOME_MET_ACTIVATES_PTK2_SIGNALING	0.111183363163305	-0.01080258	2.5849040923935	0.0102505271703164	0.020466602096607	-3.467076666	Up
LIU_SOX4_TARGETS_DN	0.0539023901353901	0.00577071173427847	2.58282698613878	0.0103109553392652	0.0205721953683122	-3.472261849	Up
REACTOME_UNWINDING_OF_DNA	0.168061304005474	-0.006474075	2.58140074518368	0.0103526326057672	0.0206288252276074	-3.475819946	Up
MIKKELSEN_ES_LCP_WITH_H3K27ME3	0.148271615853828	-0.0109229	2.5793	0.010412	0.020741	-3.4	Up

		26	5207979985	7619588553	9812190676	80927553	
BIOCARTA_CXCR4_PATHWAY	0.113870488145247	0.0153269748067931	2.57720784281413	0.0104760308312623	0.0208412574902708	-3.486269295	Up
WP_DOPAMINERGIC_NEUROGENESIS	0.104016563164741	-0.002295381	2.57462650273038	0.0105526530623666	0.0209802426515405	-3.492694339	Up
REACTOME_RUNX3_REGULATES_P14_ARF	0.117310277430669	0.0080199027969567	2.56875978057448	0.0107286625424592	0.0213096986883341	-3.507273994	Up
REACTOME_PD_1_SIGNALING	0.117474364976012	-0.000465535	2.56772955901339	0.0107598400222228	0.0213647879136075	-3.509830972	Up
BIOCARTA_LYMPHOCYTE_PATHWAY	0.131314860189908	0.00405656340447953	2.56446806671252	0.0108590768995636	0.0215411602159128	-3.517919451	Up
WP_FARNESOID_X_RECEPTOR_PATHWAY	0.109999655457918	0.00334588793121797	2.55882279920293	0.0110327789345905	0.0218634604361186	-3.531896516	Up
REACTOME_SELECTIVE_AUTOPHAGY	0.0576143162086456	0.00344564754371006	2.55054418351275	0.0112919971856982	0.0223285844318665	-3.552340352	Up
WENG_POR_TARGETS_LIVER_DN	0.0828871250163525	-0.002596998	2.55018049538677	0.0113035085734552	0.022337114840954	-3.553237023	Up

RAGHAVACHARI_PLATELET_SPECIFIC_GENES	0.163799824765455	-0.009367066	2.54699141600323	0.0114048982357031	0.0225190885788394	-3.56109445	Up
GU_PDEF_TARGETS_DN	0.0881023798715062	-0.003406573	2.54694189911468	0.0114064788934866	0.0225190885788394	-3.561216379	Up
MIKKELSEN_IPS_LCP_WITH_H3K27ME3	0.120413664029749	-0.014371306	2.54503602167099	0.0114674663091262	0.0226322936027809	-3.56590762	Up
GERHOLD_ADIPOGENESIS_DN	0.0662525203764221	0.00661510399278354	2.54215594012219	0.0115601800841804	0.0228007746369582	-3.57299047	Up
REACTOME_NEGATIVE_REGULATION_OF_THE_PI3K_AKT_NETWORK	0.0542419267096819	0.00114124455338191	2.54155647375033	0.01157956160459	0.0228317467851621	-3.574463748	Up
SABATES_COLORECTAL_ADENOMA_SIZE_UP	0.0951702517714465	0.00599620245437346	2.5374699355977	0.0117124588022084	0.0230791212016849	-3.584498192	Up
BIOCARTA_HER2_PATHWAY	0.0910669689122963	0.000973577981691286	2.53596454794906	0.0117617569063647	0.0231689067336736	-3.58819077	Up
BIOCARTA_GHRELIN_PATHWAY	0.126059996510631	-0.004880654	2.53485095268609	0.0117983436594157	0.0232188709873156	-3.590920972	Up
SWEET_LUNG_CANCER_KRAS_DN	0.0467191795590329	0.0002585614170664	2.5337717242	0.0118338980997	0.0232814597481	-3.59356	Up

		76	5668	844	336	5824	
MEISSNER_BRAIN_ICP_WITH_H3K4ME3	0.0901007104255521	-0.0008760 61	2.5323 860424 5362	0.011879 6884920 337	0.023364 1401996 367	-3.5 9696 0123	Up
VALK_AML_CLUSTER_10	0.0854006999627665	-0.0013734 2	2.5303 713210 7189	0.011946 5475458 479	0.023480 7538369 467	-3.6 0189 2126	Up
REACTOME_TP53_REGULATES_TRANSCRIPTION_OF_GENES_INVOLVED_IN_G2_CELL_CYCLE_ARREST	0.0978075108797325	-0.0031363 05	2.5287 247408 2657	0.012001 4386031 02	0.023572 0009137 083	-3.6 0592 0143	Up
WP_MICRORNAS_IN_CARDIOMYOCYTE_HYPERTROPHY	0.0791930806735768	0.0037502 198645611	2.5281 534349 6811	0.012020 5363448 441	0.023588 8299375 425	-3.6 0731 714	Up
REACTOME_CASPASE_MEDIATED_CLEAVAGE_OF_CYTOSKELETAL_PROTEINS	0.0938066462314203	0.0077482 820376461 1	2.5262 352456 3494	0.012084 8562990 088	0.023685 0972680 605	-3.6 1200 5426	Up
MYLLYKANGAS_AMPLIFICATION_HOT_SPOT_25	0.109247769046249	0.0042047 495648090 4	2.5238 142230 1085	0.012166 4747743 076	0.023837 5343826 159	-3.6 1791 7848	Up
REACTOME_CYP2E1_REACTIONS	0.132618764664106	-0.0030167 08	2.5221 378893 6194	0.012223 2753922 695	0.023933 7130472 608	-3.6 2200 8481	Up
KEGG_CIRCADIAN_RHYTHM_MAMMAL	0.11585624652628	0.0079428 227469073 6	2.5194 379478 539	0.012315 2565187 947	0.024085 3173675 945	-3.6 2859 1494	Up
LI_PROSTATE_CANCER_EPIGENETIC	0.077120775514148	0.0052899	2.5194	0.012316	0.024085	-3.6	Up

		1495674281	0966852524	2231874891	3173675945	2866041	
BIOCARTA_ACE2_PATHWAY	0.123251793042495	-0.002881903	2.51799190538183	0.0123647731110644	0.0241650335958365	-3.632114476	Up
MEBARKI_HCC_PROGENITOR_WNT_UP_CTNNB1_INDEPENDENT	0.117374679940007	-0.005133533	2.51772625816678	0.0123738888779011	0.0241752370995065	-3.63276146	Up
NUTT_GBM_VS_AO_GLIOMA_DN	0.0791334461449606	0.00190172332594928	2.51664515608402	0.0124110490018852	0.0242402080411269	-3.635393813	Up
LIU_NASOPHARYNGEAL_CARCINOMA	0.0594200767261998	0.00484289268879058	2.51154140486909	0.0125878226386077	0.0245391379138939	-3.647806253	Up
REACTOME_CELLULAR_RESPONSE_TO_HEAT_STRESS	0.0775630609033528	0.00320984074823804	2.51047836060514	0.0126249230480624	0.0245962300584962	-3.650388573	Up
CAIRO_HEPATOBLASTOMA_CLASSES_DN	0.0373407012641787	-0.000632555	2.51047517215265	0.0126250344717849	0.0245962300584962	-3.650396317	Up
WU_ALZHEIMER_DISEASE_DN	0.0835668306542977	-0.001274445	2.50631777871664	0.0127710655727677	0.0248651204548837	-3.660485312	Up
NIKOLSKY_BREAST_CANCER_7P15_AMPLICON	0.138430990769938	-0.014299199	2.50523491713327	0.0128093476472682	0.0249318346963291	-3.663110536	Up

LEE_LIVER_CANCER_MYC_DN	0.0702106279309078	0.0024835490347113 1	2.50484823	0.0128230428043214	0.0249428475983777	-3.664047734	Up
WP_ONE_CARBON_METABOLISM	0.0782517198872447	0.000935595500232981	2.50362361553018	0.0128665004910016	0.024992381825475	-3.667014871	Up
BHAT_ESR1_TARGETS_NOT_VIA_AKT1_DN	0.0579868699548856	0.0035932567915949 1	2.50288128983855	0.0128929070160418	0.0250316777755932	-3.668812788	Up
BIOCARTA_FEEDER_PATHWAY	0.130037940360777	0.0065686152197159 6	2.50113321280941	0.0129552813437112	0.0251449128519123	-3.673044629	Up
ABE_INNER_EAR	0.0847571358134243	-0.014302085	2.49999940833803	0.0129958807467747	0.025207471335234	-3.675787896	Up
REACTOME_REGULATION_OF_GENE_EXPRESSION_IN_EARLY_PANCREATIC_PRECURSOR_CELLS	0.149592054455553	-0.011262121	2.49989301115959	0.0129996964304953	0.025207471335234	-3.676045265	Up
HOSHIDA_LIVER_CANCER_SURVIVAL_UP	0.0502905423671294	0.000575080104906681	2.49936634335081	0.0130185988044608	0.0252362407180788	-3.677319094	Up
PID_TELOMERASE_PATHWAY	0.0680363208616704	0.006644468558248	2.49822160284068	0.0130597684423352	0.0253002442951231	-3.680086945	Up
MIKKELSEN_MEF_LCP_WITH_H3K27ME3	0.100348345865197	-0.006169827	2.4964312206	0.0131243901916	0.0254095726511	-3.68441	Up

			3157	512	476	3453	
MARIADASON_RESPONSE_TO_BUTYRATE_SULINDAC_6	0.0708020925346332	0.00573463650848187	2.49463399830685	0.0131895445944555	0.0255277528212615	-3.68875351	Up
HOLLERN_SOLID_NODULAR_BREAST_TUMOR_DN	0.0845584544940797	-0.003359529	2.49352312230992	0.0132299606414311	0.0255979942959361	-3.691434637	Up
RICKMAN_HEAD_AND_NECK_CANCER_F	0.0774624198731524	-0.007992322	2.49314974948703	0.01324356944095	0.0256163401433397	-3.692335526	Up
WP_ACTIVATION_OF_NLRP3_INFLAMMASOME_BY_SARSCOV2	0.16199492740468	0.000394348806832159	2.49251881731307	0.0132665941170122	0.0256521932259979	-3.69385757	Up
WP_ARYLAMINE_METABOLISM	0.186779489078376	0.00957932791170355	2.49241541599454	0.0132703709438826	0.0256521932259979	-3.694106978	Up
GUTIERREZ_CHRONIC_LYMPHOCYTIC_LEUKEMIA_DN	0.0753232692727727	0.00880785899413044	2.49197373055196	0.0132865146667855	0.0256754036540278	-3.695172227	Up
WP_IRINOTECAN_PATHWAY	0.114993189520955	-0.003976352	2.49090557430133	0.0133256282376027	0.0257429736915033	-3.697747642	Up
MURAKAMI_UV_RESPONSE_6HR_UP	0.0807452662271895	-0.004045912	2.49061254775765	0.0133363761066952	0.0257557207511689	-3.698453969	Up
HOLLERN_PAPILLARY_BREAST_TUMOR	0.0912844962543453	-0.0042619	2.4891	0.013390	0.025844	-3.7	Up

		21	4110521102	46344386	0940908081	01999614	
REACTOME_PP2A_MEDIATED_DEPHOSPHORYLATION_OF_KEY_METABOLIC_FACTORS	0.114306949790623	0.0017485353584524	2.48766591980013	0.0134448839349968	0.0259330001816423	-3.705552268	Up
JOHNSTONE_PARVB_TARGETS_2_UP	0.0825710846467381	-0.000923159	2.48712621653486	0.0134648429619295	0.0259634297187624	-3.70685152	Up
RICKMAN_HEAD_AND_NECK_CANCER_A	0.0681255296752911	-0.003596966	2.48673170701198	0.0134794491583856	0.0259835220267389	-3.707801069	Up
QI_PLASMACYTOMA_DN	0.039836967057036	0.00395587113567787	2.48321940885894	0.0136101091541433	0.0262109672726552	-3.716248515	Up
KEGG_RENIN_ANGIOTENSIN_SYSTEM	0.110196771798679	0.0030187722713982	2.48106093374477	0.0136909629130709	0.0263585008689302	-3.721434211	Up
WOO_LIVER_CANCER_RECURRENCE_DN	0.0610921706737892	0.00080766182780338	2.47935688093676	0.0137550955860749	0.0264655543405974	-3.725525121	Up
BIOCARTA_RELA_PATHWAY	0.144919209105332	0.00296351214904483	2.47654271216545	0.0138615920276747	0.0266573297394075	-3.732275196	Up
REACTOME_ACTIVATED_NTRK3_SIGNALS_THROUGH_PI3K	0.128355447763563	-0.009580709	2.47488864177863	0.0139245278951591	0.0267503133758554	-3.736239235	Up

PIONTEK_PKD1_TARGETS_DN	0.0834077878166695	0.00507827142136895	2.4747350127643	0.0139303861914379	0.0267530653125789	-3.736607284	Up
BERTUCCI_INVASIVE_CARCINOMA_DUCTAL_VS_LOBULAR_DN	0.0865276460043527	-0.001323432	2.47297783839258	0.0139975478440883	0.0268711513189164	-3.74081539	Up
GOTZMANN_EPITHELIAL_TO_MESENCHYMAL_TRANSITION_UP	0.0562479022515282	0.000170847421809103	2.47287317748378	0.0140015571823246	0.0268711513189164	-3.741065944	Up
BENPORATH_ES_CORE_NINE_CORRELATED	0.0544539568704802	0.00324556202778689	2.47278694404467	0.0140048613688891	0.0268711513189164	-3.741272375	Up
BIOCARTA_ERYTH_PATHWAY	0.126367361592542	-0.00101978	2.47023964335324	0.0141027783362179	0.0270399749348596	-3.747367162	Up
CHEMNITZ_RESPONSE_TO_PROSTAGLANDIN_E2_DN	0.0271837160757279	0.000807760232969436	2.47017338941021	0.0141053331894904	0.0270399749348596	-3.747525604	Up
JEPSEN_SMRT_TARGETS	0.0644469622318147	-0.003475275	2.47004525782308	0.0141102753043355	0.0270399749348596	-3.747832011	Up
REACTOME_TRANSCRIPTIONAL_REGULATION_OF_GRANULOPOIESIS	0.0839659872173909	0.00365160506883018	2.46720086896643	0.0142203814659441	0.0272240378339773	-3.754630012	Up
WIEMANN_TELOMERE_SHORTENING_AND_CHRONIC_LIVER_DAMAGE_UP	0.116895150318894	0.0023763353690035	2.4668302959	0.0142347823017	0.0272365270490	-3.75551	Up

			4593	716	432	5118	
REACTOME_PHASE_1_INACTIVATION_OF_FAST_NA_CHANNELS	0.156000042268342	-0.002860596	2.46521832674686	0.014297575658494	0.0273457319268461	-3.759363796	Up
SESTO_RESPONSE_TO_UV_C6	0.0781501538327298	-0.000169663	2.46424388917137	0.0143356533587923	0.0273957513540714	-3.761689159	Up
WP_SECRETION_OF_HYDROCHLORIC_ACID_IN_PARIETAL_CELLS	0.158775637754041	0.00135040608432629	2.46319047291705	0.0143769184150316	0.0274577023391081	-3.764202005	Up
PID_AR_TF_PATHWAY	0.0756031277785076	0.00615471605792286	2.45998720261222	0.014503046711388	0.0276770542190611	-3.771836853	Up
BENPORATH_PRC2_TARGETS	0.0709256549321291	-0.00469842	2.45906637870004	0.0145394850858072	0.0277340454602353	-3.774029836	Up
KEGG_GLYCEROLIPID_METABOLISM	0.0694303224501953	0.00123467601023063	2.45826618986729	0.01457121563117	0.0277774985941867	-3.775934882	Up
REACTOME_POST_CHAPERONIN_TUBULIN_FOLDING_PATHWAY	0.105594108028015	-0.009057962	2.45731100078804	0.0146091728863034	0.027841306756305	-3.778208169	Up
AGUIRRE_PANCREATIC_CANCER_COPY_NUMBER_UP	0.0546360525050748	0.00419796056131182	2.45693718077903	0.014624051570979	0.027861107458891	-3.779097605	Up
BYSTRYKH_HEMATOPOIESIS_STEM_CELL_AND_BRAI	0.0462444747535616	0.0021161	2.4534	0.014765	0.028103	-3.7	Up

N_QTL_TRANS		0999901483	119562831	0234679798	8027187214	87478866	
LINDVALL_IMMORTALIZED_BY_TERT_UP	0.0691288344945777	0.00130067634373167	2.45314055275104	0.0147759265105323	0.0281159337372391	-3.788123654	Up
ZHOU_TNF_SIGNALING_30MIN	0.068376375188437	-0.001698965	2.44965397836045	0.0149166277955377	0.0283749643661975	-3.796400818	Up
PID_S1P_META_PATHWAY	0.0884630382711388	-0.002916245	2.44875188129273	0.0149532247844828	0.0284358658815211	-3.798540571	Up
VART_KSHV_INFECTION_ANGIOGENIC_MARKERS_DN	0.0813582114065904	-0.001417267	2.44836326591393	0.0149690149356589	0.0284571747949878	-3.799462125	Up
BIOCARTA_MELANOCYTE_PATHWAY	0.134267053649812	0.00121372444124612	2.44796662662236	0.0149851463400987	0.0284704019996917	-3.800402563	Up
REACTOME_NEPHRIN_FAMILY_INTERACTIONS	0.0860025381406841	0.0121969945547744	2.44717000130656	0.0150175917836884	0.0285233146270973	-3.802290932	Up
NIKOLSKY_BREAST_CANCER_19Q13.4_AMPLICON	0.138592043460824	-0.000263669	2.44661490533598	0.0150402368373386	0.0285575864329643	-3.803606418	Up
REACTOME_G_ALPHA_Q_SIGNALLING_EVENTS	0.0600939687457054	-0.004118109	2.44543296887633	0.0150885545186725	0.0286405681643425	-3.806406461	Up

REACTOME_RUNX3_REGULATES_NOTCH_SIGNALING	0.119732535020673	0.0127327195580996	2.44177051997996	0.0152391497777927	0.0289087416475426	-3.815074685	Up
REACTOME_FCGAMMA_RECEPTOR_FCGR_DEPENDENT_PHAGOCYTOSIS	0.0849878078671346	0.00465882927953057	2.4412519997161	0.0152605778491742	0.0289405458936219	-3.816300905	Up
REACTOME_CA_DEPENDENT_EVENTS	0.079497843112998	-0.003075846	2.43982006334213	0.0153198918243587	0.0290441565527778	-3.819685918	Up
BROWNE_HCMV_INFECTION_16HR_DN	0.0584770728693302	-0.002709298	2.43889054189318	0.0153585037940519	0.0290995827379976	-3.821882234	Up
PID_ENDOTHELIN_PATHWAY	0.0894594423518674	-0.001249659	2.43773051737548	0.0154068115715626	0.0291644036061876	-3.824622069	Up
KYNG_RESPONSE_TO_H2O2_VIA_ERCC6	0.0866096002777038	0.00236138864389681	2.43365259295453	0.0155777005360088	0.0294519607758168	-3.834243717	Up
NELSON_RESPONSE_TO_ANDROGEN_DN	0.0984673581936903	-0.002741711	2.43290706054979	0.0156091233882052	0.0294844275321332	-3.836001094	Up
CHIBA_RESPONSE_TO_TSA_UP	0.104822345007901	-0.003361858	2.43265620131297	0.0156197092550656	0.0294954473216284	-3.836592306	Up
WP_CYSTEINE_AND_METHIONINE_CATABOLISM	0.0866343929720431	0.0016987998943974	2.4323952026	0.0156307297290	0.0295072808479	-3.83720	Up

		2	7578	134	277	7352	
WONG_ADULT_TISSUE_STEM_MODULE	0.0358705537470744	0.000668025265719462	2.43097891745693	0.0156906513287423	0.0296113933710865	-3.840543741	Up
KEGG_NEUROTROPHIN_SIGNALING_PATHWAY	0.0779046898945541	0.00799831253137453	2.43051859718289	0.0157101707170914	0.0296392187358621	-3.841627732	Up
ACEVEDO_LIVER_CANCER_WITH_H3K9ME3_UP	0.0555102153919976	-0.003668738	2.42979242383288	0.015741006984308	0.0296883714225463	-3.84333737	Up
ST_ADRENERGIC	0.0933038993576849	0.00150058248277348	2.42152738951485	0.0160957636027352	0.0303298132004181	-3.862761351	Up
URS_ADIPOCYTE_DIFFERENTIATION_UP	0.0730143032321962	0.00167363684391841	2.41900431094455	0.0162054593881105	0.0305087310955419	-3.868678304	Up
REACTOME_LISTERIA_MONOCYTOGENES_ENTRY_INTO_HOST_CELLS	0.0919931211215956	0.00976557508145589	2.41877656694963	0.0162153934333787	0.0305181767862285	-3.869212103	Up
KEGG_ADIPOCYTOKINE_SIGNALING_PATHWAY	0.0644989939764275	9.71921938949518e-05	2.41674076069736	0.0163044332687293	0.0306764526520165	-3.873981599	Up
VALK_AML_WITH_FLT3_ITD	0.0662728989829475	-0.000352401	2.41622323424124	0.016327137010112	0.0307098604308379	-3.875193449	Up
WP_NICOTINE_METABOLISM	0.171759493027291	-0.0074231	2.4160	0.016333	0.030712	-3.8	Up

		49	7511264351	6402017171	7854383695	75540248	
REACTOME_FGFR3B_LIGAND_BINDING_AND_ACTIVATION	0.137320854062273	-0.005669691	2.41526963462668	0.0163690443089047	0.0307700357436013	-3.877425765	Up
HOFFMANN_PRE_BI_TO_LARGE_PRE_BII_LYMPHOCYTE_DN	0.0568223915013703	0.00114265836517534	2.41436054712545	0.0164090838938603	0.0308215074152226	-3.879553094	Up
SHIRAISHI_PLZF_TARGETS_DN	0.111015440139061	-0.003202135	2.41430976618581	0.0164113230235719	0.0308215074152226	-3.879671902	Up
REACTOME_PROSTANOID_LIGAND_RECEPTORS	0.113414615777737	-0.005397007	2.41381011175625	0.0164333691315973	0.0308535759830079	-3.880840779	Up
WP_FATTY_ACID_OMEGA_OXIDATION	0.114633057447354	-0.003268981	2.41213658303112	0.0165074003931059	0.0309644709096429	-3.884754093	Up
KEGG_VIBRIO_CHOLERAE_INFECTION	0.0699369827960838	0.00227664757664416	2.4114093993282	0.0165396602289114	0.0310156105863605	-3.88864537	Up
TURASHVILI_BREAST_DUCTAL_CARCINOMA_VS_DUCTAL_NORMAL_DN	0.0621890266548668	-0.003611154	2.41119194343199	0.0165493179672777	0.0310243481192669	-3.886961852	Up
REACTOME_PLATELET_HOMEOSTASIS	0.0556218142447873	-0.004630138	2.41033653948707	0.0165873568473042	0.0310815448020392	-3.88896034	Up

PID_CD8_TCR_DOWNSTREAM_PATHWAY	0.064434297297605	0.0050124649763939	2.40603139901816	0.016779976068406	0.0314187968194859	-3.899008159	Up
BONCI_TARGETS_OF_MIR15A_AND_MIR16_1	0.0538887346946274	0.000768951837495557	2.40575284343411	0.0167925068436949	0.031432777436313	-3.89965769	Up
LIU_TARGETS_OF_VMYB_VS_CMYB_UP	0.0846132918777826	2.88746805568653e-05	2.40413504446376	0.0168654466582315	0.0315502795080299	-3.903428621	Up
PLASARI_NFIC_TARGETS_BASAL_UP	0.0942242547076671	-0.00212305	2.40244987328422	0.0169417211634258	0.0316834176744151	-3.907354004	Up
WP_CLASSICAL_PATHWAY_OF_STEROIDOGENESIS_INCLUDING_DISEASES	0.116185733044909	0.00492337134646431	2.40131271984183	0.0169933630377297	0.0317608564815381	-3.910001361	Up
WP_INTERLEUKIN1_INDUCED_ACTIVATION_OF_NFKAPPAB	0.111587938866076	0.00569097133278847	2.40078961126352	0.0170171657208057	0.031795769906394	-3.911218783	Up
REACTOME_MULTIFUNCTIONAL_ANION_EXCHANGERS	0.115495273241953	-0.003135237	2.40039584100418	0.0170351026025664	0.0318101329284386	-3.912135031	Up
REACTOME_SUMOYLATION_OF_INTRACELLULAR_RECEPTORS	0.0740366178611863	0.000366347357799223	2.39986607929466	0.0170592604316069	0.0318456630072132	-3.913367484	Up
REACTOME_RHO_GTPASES_ACTIVATE_KTN1	0.108247498888048	0.0099793794528200	2.3980219847	0.0171435894311	0.0319838471894	-3.91765	Up

		7	2792	956	893	5606	
SWEET_KRAS_TARGETS_UP	0.0691525210689151	0.00272663242827303	2.39744851546532	0.0171698885748562	0.0320232867740782	-3.918988465	Up
DACOSTA_UV_RESPONSE_VIA_ERCC3_TTD_DN	0.0725990358773184	0.00873354070043007	2.39702694400339	0.017189244414214	0.0320401321558637	-3.919968087	Up
KEGG_GLYCINE_SERINE_AND_THREONINE_METABOLISM	0.0833967011946216	-0.00402241	2.39591073668748	0.0172405864091346	0.0321261842814465	-3.922561065	Up
CROMER_TUMORIGENESIS_UP	0.0857170997495634	-0.00598156	2.39441350085016	0.0173096668471726	0.0322452287276111	-3.926037364	Up
HINATA_NFKB_TARGETS_KERATINOCYTE_DN	0.0977323713159245	-0.006822923	2.39424915272821	0.017317264493141	0.0322497031829962	-3.926418822	Up
REACTOME_DEFECTIVE_EXT2_CAUSES_EXOSTOSES_2	0.105896922760607	-0.006776761	2.39338966521669	0.0173570456416018	0.0323140918708526	-3.928413317	Up
WP_REGULATION_OF_ACTIN_CYTOSKELETON	0.0719388613675378	0.00134155877300987	2.39248800286384	0.0173988654202129	0.0323602450459735	-3.930504943	Up
WP_METABOLIC_REPROGRAMMING_IN_COLON_CANCER	0.104450901079817	0.0118531877964194	2.39233619218451	0.0174059152442747	0.0323602450459735	-3.93085703	Up
REACTOME_N_GLYCAN_ANTENNAE_ELONGATION	0.0857063587910049	0.0035901	2.3922	0.017407	0.032360	-3.9	Up

		107428386	9338284453	9036907533	2450459735	30956312	
DORN_ADENOVIRUS_INFECTION_32HR_DN	0.078549801495835	0.0109504000666832	2.38885536250421	0.0175682512312422	0.0326485435306349	-3.938924081	Up
WP_CARDIAC_PROGENITOR_DIFFERENTIATION	0.0918303949364571	-0.00190886	2.38353857120317	0.0178187871732118	0.0330822405709419	-3.951224343	Up
KEGG_INOSITOL_PHOSPHATE_METABOLISM	0.0658462614453437	0.00529609245067138	2.38233311323737	0.0178760261305551	0.0331609366982533	-3.954009484	Up
BIOCARTA_EIF4_PATHWAY	0.0913103637762376	0.0120185570542137	2.38009698043358	0.0179826337847522	0.0333288169310113	-3.959172358	Up
BERENJENO_TRANSFORMED_BY_RHOA_REVERSIBLY_UP	0.104952667860108	-0.002018917	2.37942095607672	0.0180149732872736	0.0333787878191365	-3.960732275	Up
WP_UREA_CYCLE_AND_METABOLISM_OF_AMINO_GROUPS	0.0799068860798008	-0.003725232	2.37786650763472	0.0180895289921517	0.0334969291331402	-3.964317531	Up
REACTOME_REGULATION_OF_TP53_EXPRESSION_AND_DEGRADATION	0.070498515679382	0.00504139749945415	2.37493577374071	0.0182308344821472	0.0337284022744226	-3.971071002	Up
REACTOME_HYALURONAN_METABOLISM	0.106605252541372	-0.00438697	2.37278187527094	0.0183353033876647	0.0338913722832743	-3.976029268	Up

WP_WHITE_FAT_CELL_DIFFERENTIATION	0.0857141573604033	0.00684242206599611	2.37249957119475	0.0183490347481361	0.0339066563506046	-3.976678811	Up
NAKAMURA_ALVEOLAR_EPITHELIUM	0.144785501155287	-0.004282869	2.37217542413192	0.0183648125262147	0.0339257117113734	-3.977424538	Up
REACTOME_ADRENALINE_NORADRENALINE_INHIBITS_INSULIN_SECRETION	0.0839750010358187	-0.004024704	2.3714321741577	0.0184010351182673	0.0339825126388233	-3.979134075	Up
VALK_AML_CLUSTER_12	0.070044545758245	-0.001350403	2.36985404947852	0.0184781542073408	0.034104639656546	-3.982762188	Up
TOMIDA_METASTASIS_UP	0.0726059628135138	0.002852731949548	2.36854769290355	0.01854220765349	0.0342126881406697	-3.985763753	Up
REACTOME_SYNTHESIS_OF_PIPS_AT_THE_GOLGI_MEMBRANE	0.0866337707558115	0.00511891307776641	2.36382189757605	0.0187755579302504	0.0346329533649522	-3.996608758	Up
TIAN_TNF_SIGNALING_NOT_VIA_NFKB	0.110375960533682	0.00723332398836623	2.3636013816815	0.0187865093517673	0.0346382302881777	-3.997114302	Up
WP_SEROTONIN_TRANSPORTER_ACTIVITY	0.127986580017741	-0.004740641	2.36353957738895	0.0187895797293853	0.0346382302881777	-3.997255983	Up
BIOCARTA_IL6_PATHWAY	0.106023705713428	0.0057511280971366	2.3619894254	0.0188667342584	0.0347598158331	-4.00080	Up

		5	387	029	573	8417	
LEE_NEURAL_CREST_STEM_CELL_UP	0.0571414630710884	-0.003095339	2.36085528525141	0.0189233592819635	0.0348537955676996	-4.003406073	Up
PID_TRKR_PATHWAY	0.0619470557138374	0.00651153208132586	2.35794305942575	0.0190694450242515	0.0350604399483202	-4.010070802	Up
FUJIWARA_PARK2_IN_LIVER_CANCER_UP	0.117906606442614	-0.000666456	2.35456517536673	0.0192401304608273	0.0353595872981654	-4.017791311	Up
ONDER_CDH1_TARGETS_1_UP	0.0442574216975814	0.00573312033052718	2.35404528818757	0.0192665193707447	0.0353913245736053	-4.018978624	Up
WIERENGA_STAT5A_TARGETS_DN	0.0805227715995874	5.62564809439492e-05	2.35328097909035	0.0193053726171297	0.0354522035013385	-4.020723688	Up
REACTOME_NECTIN_NECL_TRANS_HETERODIMERIZATION	0.182991524138514	-0.012641975	2.3502423747789	0.0194605201558379	0.0356948725198835	-4.02765602	Up
SCHAEFFER_PROSTATE_DEVELOPMENT_AND_CANCER_BOX1_UP	0.0870258274865827	0.00614283707221353	2.34999073507327	0.0194734175514549	0.035707977176331	-4.02822973	Up
BIOCARTA_PRC2_PATHWAY	0.138531180384793	0.0112061569990733	2.34776201303915	0.0195879752862986	0.0359074313650489	-4.03330839	Up
REACTOME_EPHA_MEDIATED_GROWTH_CONE_COLL	0.0846315632991531	-0.0059761	2.3465	0.019651	0.036012	-4.0	Up

APSE		33	3842300173	1199059256	5483484146	36094655	
HOQUE_METHYLATED_IN_CANCER	0.06171677497457	0.00143000984757473	2.34409700621459	0.0197776460471575	0.0362123448420963	-4.041649891	Up
OISHI_CHOLANGIOMA_STEM_CELL_LIKE_DN	0.0593140532105549	0.000238820103673055	2.34243514829448	0.0198641802186745	0.0363441092718529	-4.04542813	Up
MA_MYELOID_DIFFERENTIATION_DN	0.062403599614174	-0.000663607	2.34237772517716	0.0198671762138548	0.0363441092718529	-4.045558636	Up
BENPORATH_EED_TARGETS	0.054508150340115	-0.002381653	2.34214262139344	0.0198794466613333	0.0363558413161154	-4.046092924	Up
WP_NEURAL_CREST_CELL_MIGRATION_DURING_DEVELOPMENT	0.104624042225984	0.0102614326725641	2.341433662	0.0199164886665292	0.0364128557152127	-4.047703768	Up
LINDVALL_IMMORTALIZED_BY_TERT_DN	0.0632042834583605	-0.00610612	2.34119448812951	0.0199289988049628	0.0364249986991768	-4.048247096	Up
ELLWOOD_MYC_TARGETS_UP	0.090759466281001	0.00165098734748648	2.33280089439915	0.0203724205580105	0.0371588640621719	-4.067280905	Up
REACTOME_ANTIGEN_PRESENTATION_FOLDING_ASSEMBLY_AND_PEPTIDE_LOADING_OF_CLASS_I_MHC	0.10928051365613	0.0076961031053879	2.33194268033039	0.0204182427566674	0.0372205676330791	-4.06922334	Up

BIOCARTA_IRES_PATHWAY	0.144182764221332	0.0198107978841458	2.33049187100563	0.0204959106612912	0.0373292598223692	-4.072505461	Up
REACTOME_GLUCAGON_SIGNALING_IN_METABOLIC_REGULATION	0.0815247962902394	-0.003125764	2.32602036246103	0.0207369211643723	0.0376907963886555	-4.082608864	Up
WP_VITAMIN_B12_METABOLISM	0.0629986791073696	-0.00495349	2.3246403600032	0.0208118020224518	0.037815824108126	-4.085723223	Up
REACTOME_STAT5_ACTIVATION	0.141972947165605	0.00792346908227291	2.32226218446171	0.0209414012936683	0.03802904559093	-4.091086066	Up
WP_NEURAL_CREST_CELL_MIGRATION_IN_CANCER	0.103177753464337	0.0085658989105797	2.32079312918202	0.021021810665849	0.0381639013755265	-4.094396184	Up
MYLLYKANGAS_AMPLIFICATION_HOT_SPOT_30	0.139658641587445	0.0160145546506398	2.3184545423715	0.0211503722701494	0.038386070374508	-4.099661402	Up
REACTOME_HYALURONAN_UPTAKE_AND_DEGRADATION	0.11568716589757	-0.003971974	2.31822357753201	0.0211631066315422	0.0383979546512664	-4.100181131	Up
KEGG_ALPHA_LINOLENIC_ACID_METABOLISM	0.101682852983681	-0.007904621	2.31554772135906	0.0213111315863025	0.0386439362419456	-4.106198856	Up
CHASSOT_SKIN_WOUND	0.137391758383866	0.0198473885158851	2.3119658059	0.0215106971881	0.0389716571648	-4.11424	Up

			9981	385	498	3754	
REACTOME_PROLONGED_ERK_ACTIVATION_EVENTS	0.123446189757174	0.0126505074782571	2.30978911900865	0.0216327682797143	0.0391699511995878	-4.119126691	Up
PID_FOXM1_PATHWAY	0.0738096946092258	-0.005651903	2.3085617446078	0.0217018678031688	0.0392836084731025	-4.121878096	Up
GAVIN_IL2_RESPONSIVE_FOXP3_TARGETS_UP	0.0809973648035837	0.00426236136870894	2.30817371503632	0.0217237534911195	0.0392943263874947	-4.122747649	Up
REACTOME_SIGNALING_BY_LEPTIN	0.113058310671001	4.48494138484859e-05	2.30812006904522	0.0217267807575128	0.0392943263874947	-4.122867856	Up
REACTOME_OPIOID_SIGNALLING	0.0512054742205665	-0.001577104	2.30739102792374	0.0217679574724818	0.0393458683843025	-4.12450118	Up
REACTOME_GLYCOSPHINGOLIPID_METABOLISM	0.075106240810285	-0.001102131	2.30440274534274	0.0219374519879765	0.0396176213236457	-4.131190863	Up
REACTOME_NEGATIVE_FEEDBACK_REGULATION_OF_MAPK_PATHWAY	0.156198613631596	0.0109647279058821	2.30147126834064	0.0221048451086139	0.039896706481293	-4.137745275	Up
REACTOME_EPH_EPHRIN_MEDIATED_REPULSION_OF_CELLS	0.0844209864216183	-0.000617014	2.30112882414936	0.0221244720243402	0.0399205226322906	-4.138510413	Up
BIOCARTA_FMLP_PATHWAY	0.0907609063698621	0.0084345	2.2991	0.022238	0.040115	-4.1	Up

		8479962514	3674844126	9487022615	4183652825	42959225	
BIOCARTA_STEM_PATHWAY	0.090731870208387	0.000479254889204054	2.2955734121113	0.022445011587097	0.0404636035205085	-4.150907814	Up
SAKAI_CHRONIC_HEPATITIS_VS_LIVER_CANCER_DN	0.0925107401320703	-0.003515018	2.29498149014196	0.0224794027765928	0.0405138365372566	-4.152227041	Up
MIKKELSEN_ES_LCP_WITH_H3K4ME3_AND_H3K27ME3	0.135274188339686	-0.000194829	2.29483958559347	0.0224876543969342	0.040516943640572	-4.152543258	Up
REACTOME_VEGF_LIGAND_RECEPTOR_INTERACTIONS	0.123079902249759	-0.005701511	2.29406874080175	0.0225325245824488	0.0405756269813436	-4.154260661	Up
NABA_MATRISOME	0.0719645693488888	-0.003965701	2.2940554504233	0.0225332988891077	0.0405756269813436	-4.154290266	Up
KEGG_GLYCOSAMINOGLYCAN_BIOSYNTHESIS_KERATAN_SULFATE	0.0976457076661238	-0.004186162	2.29187131365867	0.0226608645416726	0.0407462300724687	-4.159153369	Up
REACTOME_GLYCEROPHOSPHOLIPID_BIOSYNTHESIS	0.0519302971288022	-0.001194111	2.28914789804854	0.0228208106178629	0.04101006702521	-4.16521096	Up
REACTOME_SIGNALING_BY_MET	0.0573004144593783	-0.000203984	2.28526428250983	0.0230506006219636	0.0413511786302104	-4.17383715	Up

WP_CORI_CYCLE	0.108401634175907	0.00517607773078092	2.28505313514149	0.0230631517253239	0.0413617401788748	-4.174305741	Up
ENGELMANN_CANCER_PROGENITORS_DN	0.0606325596984947	-0.001822332	2.28349825724795	0.0231557612812094	0.0415038435669843	-4.177755138	Up
KIM_ALL_DISORDERS_DURATION_CORR_DN	0.0872353406166889	0.000524282303914621	2.28041837182454	0.0233401601273521	0.0418222788425157	-4.184580993	Up
ZHAN_MULTIPLE_MYELOMA_LB_UP	0.0599368024269416	-0.001743036	2.27879938940967	0.0234376047573495	0.0419726522587617	-4.188165537	Up
BRUINS_UVC_RESPONSE_VIA_TP53_GROUP_A	0.0290687965945205	-0.000945522	2.27815628258658	0.0234764110031746	0.0420300210835606	-4.189588742	Up
VANHARANTA_UTERINE_FIBROID_DN	0.0645189290068573	0.00203788949001085	2.27797106018498	0.0234875980568515	0.0420379242038284	-4.18999857	Up
STEARMAN_LUNG_CANCER_EARLY_VS_LATE_DN	0.0886641179460163	0.00544278122578634	2.27592253634651	0.0236116352446152	0.0422233995860924	-4.19452905	Up
MIKKELSEN_MCV6_LCP_WITH_H3K27ME3	0.0747636412199266	-0.001341128	2.27466879934501	0.0236878300194163	0.0423474541850567	-4.197299857	Up
KEGG_REGULATION_OF_ACTIN_CYTOSKELETON	0.0672989726035799	0.0007835002812014	2.2720912895	0.0238451499238	0.0425972370923	-4.20299	Up

		12	8266	321	532	1635	
WP_ANGIOPOIETIN_LIKE_PROTEIN_8_REGULATORY_PATHWAY	0.0606809774510195	0.00622978732230399	2.2720424603472	0.0238481390198046	0.0425972370923532	-4.203099402	Up
REACTOME_GLUCAGON_TYPE_LIGAND_RECEPTORS	0.0756020016941925	-0.005389938	2.2662293378709	0.0242063341849185	0.0431625154512465	-4.215913131	Up
HELLER_HDAC_TARGETS_UP	0.0552993814512225	0.00151428487708073	2.26513581638565	0.0242742367212005	0.0432587388252919	-4.218320022	Up
REACTOME_AMYLOID_FIBER_FORMATION	0.0763913100881082	4.1525056951376e-05	2.26072945587982	0.0245495358430772	0.0437116950596616	-4.228007282	Up
STAMBOLSKY_TARGETS_OF_MUTATED_TP53_UP	0.0667825818557002	0.00521181899982568	2.26058686703285	0.0245584897101235	0.0437150976859009	-4.228320456	Up
WP_CELL_DIFFERENTIATION_INDEX_EXPANDED	0.0840644960965041	-0.008010855	2.25993882689105	0.0245992192467401	0.0437715401448199	-4.229743535	Up
REACTOME_NOTCH4_INTRACELLULAR_DOMAIN_REGULATES_TRANSCRIPTION	0.0968176845320535	0.0126870956798071	2.2598580190576	0.0246043021693695	0.0437715401448199	-4.229920959	Up
REACTOME_TGF_BETA_RECEPTOR_SIGNALING_IN_EMT_EPITHELIAL_TO_MESENCHYMAL_TRANSITION_	0.102380190414564	0.00633864105496845	2.25952211111874	0.0246254410424274	0.0437965938539675	-4.230658424	Up
WATANABE_COLON_CANCER_MSI_VS_MSS_DN	0.0734536070271128	0.0010827	2.2580	0.024718	0.043937	-4.2	Up

		6660753676	414883629	8061016144	4654847424	33907779	
REACTOME_RUNX2_REGULATES_GENES_INVOLVED_IN_CELL_MIGRATION	0.111167712484839	0.0065870284253213	2.25751601245652	0.0247520155854331	0.0439838994385294	-4.235060488	Up
REACTOME_LAMININ_INTERACTIONS	0.075676048953242	-0.006379049	2.25721622713239	0.0247709790803936	0.0440049991848893	-4.235717996	Up
LEE_LIVER_CANCER_HEPATOBLAST	0.0885303413276744	0.000844255852717297	2.25559161335274	0.0248739673570438	0.0441626760255065	-4.239279736	Up
REACTOME_STIMULI_SENSING_CHANNELS	0.0633622029672065	-0.00433994	2.25292522442858	0.0250438044723673	0.0444513604398534	-4.245120067	Up
MIKKELSEN_IPS_LCP_WITH_H3K4ME3	0.0759176601402883	-0.002998626	2.25223171669737	0.0250881429036907	0.0444793210520445	-4.246638003	Up
CHENG_IMPRINTED_BY_ESTRADIOL	0.0425539699307913	-0.002668191	2.25152578977894	0.0251333454944339	0.0445213114965615	-4.248182658	Up
REACTOME_DIGESTION	0.101906887414496	-0.000768798	2.25118412284726	0.0251552488846677	0.0445473979535328	-4.248930101	Up
KANG_AR_TARGETS_UP	0.108962625499295	0.00108540839302272	2.24871698053419	0.0253139047138064	0.04481092460141	-4.25432406	Up

PID_P38_GAMMA_DELTA_PATHWAY	0.120879782723662	-0.002095597	2.24426372739593	0.0256024874129135	0.0452877285186533	-4.264045859	Up
REACTOME_TP53_REGULATES_TRANSCRIPTION_OF_CELL_CYCLE_GENES	0.0697817603167177	0.000689592595861858	2.23764486136398	0.0260366934139785	0.0460351393204648	-4.278461034	Up
REACTOME_RETINOID_CYCLE_DISEASE_EVENTS	0.116115206872042	-0.011631091	2.23759703458797	0.0260398540606013	0.0460351393204648	-4.278565047	Up
REACTOME_DEVELOPMENTAL_BIOLOGY	0.0288227717904727	-0.000729132	2.23646077677899	0.0261150421287582	0.0461549164274494	-4.281035514	Up
GERHOLD_RESPONSE_TO_TZD_UP	0.110597947371335	0.00373738594005562	2.23468575344361	0.0262328759573653	0.0463499746847044	-4.284892374	Up
SHIPP_DLBCL_CURED_VS_FATAL_UP	0.0710872079637006	0.000763115168100234	2.23442376929901	0.0262503066798253	0.0463675735804427	-4.285461376	Up
CROMER_TUMORIGENESIS_DN	0.068249316203052	-0.00177426	2.23124413182984	0.0264626626526089	0.0467160827886074	-4.292362089	Up
PYEON_HPV_POSITIVE_TUMORS_DN	0.106633578764386	-0.002827947	2.22987159302147	0.0265547894351467	0.0468520687958942	-4.295337958	Up
REACTOME_INACTIVATION_OF_CDC42_AND_RAC1	0.11696408478123	-0.009748291	2.2290285227	0.0266115155187	0.0469388112601	-4.29716	Up

			139	094	959	4985	
REACTOME_MET_RECEPTOR_RECYCLING	0.130517193773414	0.0079184019241513	2.22751372486332	0.026713703302058	0.0470922909389021	-4.300446047	Up
REACTOME_UNBLOCKING_OF_NMDA_RECEPTORS_GLUTAMATE_BINDING_AND_ACTIVATION	0.082850869364221	-0.004928463	2.22650988636019	0.0267816094856104	0.0471985945704667	-4.302619182	Up
REACTOME_INTERLEUKIN_37_SIGNALING	0.0731364782122893	-0.004588975	2.22512185280144	0.0268757519803362	0.0473376255794401	-4.305622476	Up
REACTOME_OPSINS	0.127420928827205	-0.008248392	2.22375001423127	0.0269690781907035	0.047488530022609	-4.308588953	Up
PID_THROMBIN_PAR1_PATHWAY	0.0845713634170054	0.00331119203374553	2.22292732675636	0.027025180456553	0.0475607255420084	-4.310367095	Up
REACTOME_HUR_ELAVL1_BINDS_AND_STABILIZES_MRNA	0.103488676468769	0.0141430098960797	2.22243083378019	0.0270590872340882	0.047606506366776	-4.311439898	Up
DELASERNA_TARGETS_OF_MYOD_AND_SMARCA4	0.114431368324254	0.00373164428977759	2.22174622869484	0.0271059012164058	0.047675355299017	-4.312918789	Up
HOSHIDA_LIVER_CANCER_SUBCLASS_S3	0.0429960241544088	-0.001988806	2.22083799919237	0.0271681153189946	0.0477577150339845	-4.314880076	Up
WP_GENES_CONTROLLING_RENAL_NEPHROGENESIS	0.0785092420848567	-0.0038035	2.2200	0.027222	0.047813	-4.3	Up

		38	41096	8055418444	2297674669	16600321	
REACTOME_DIGESTION_AND_ABSORPTION	0.0972690061321693	-0.003576038	2.21988324503007	0.0272336499306527	0.0478187454369339	-4.316940998	Up
BILANGES_SERUM_SENSITIVE_VIA_TSC1	0.082981193409488	-0.003168849	2.21914616186662	0.0272843373132147	0.0478806564046151	-4.318531472	Up
BYSTRYKH_HEMATOPOIESIS_STEM_CELL_FLI1	0.109981530390967	0.000401962561158506	2.21743452156745	0.0274023583815228	0.0480334477475606	-4.322222876	Up
WP_GPCRS_CLASS_C_METABOTROPIC_GLUTAMATE_PHEROMONE	0.110543370246242	-0.008499634	2.21697527726984	0.0274340994534352	0.048075509685904	-4.323212837	Up
MAINA_VHL_TARGETS_UP	0.102385407994083	0.00783754682810182	2.21524512194475	0.027553967207818	0.0482447036555504	-4.326940633	Up
REACTOME_ORGANIC_ANION_TRANSPORTERS	0.111148599277735	-0.010272794	2.21402743149091	0.02763860271009 8	0.0483792461989786	-4.329562588	Up
BIOCARTA_IL17_PATHWAY	0.0939455532477694	0.000737659927637911	2.2127737122585	0.0277259778233339	0.0484975737551991	-4.332260668	Up
BIOCARTA_ARAP_PATHWAY	0.0979671699912401	0.00593846112899885	2.20860465651717	0.0280182551791613	0.0489747422971147	-4.341222123	Up

REACTOME_TERMINATION_OF_O_GLYCAN_BIOSYNTHESIS	0.0983008765771167	-0.00622904	2.20332882484018	0.0283919492021723	0.0495721037124848	-4.352539232	Up
BOQUEST_STEM_CELL_UP	0.0507106177040307	-0.002735578	2.20080550700478	0.0285722000976059	0.0498520672831074	-4.357942728	Up
REACTOME_FERTILIZATION	0.0835919335384741	-0.004016754	2.20077671916597	0.0285742622345442	0.0498520672831074	-4.358004341	Up
REACTOME_P75NTR_REGULATES_AXONOGENESIS	0.129608960271436	-0.00753961	2.20046297764138	0.0285967445952318	0.0498595487928662	-4.358675769	Up
BIOCARTA_NPP1_PATHWAY	0.113855936376984	0.000243898856757985	2.19653064532763	0.0288798327385788	0.0502543094500585	-4.367083385	Up
DASU_IL6_SIGNALING_SCAR_DN	0.0852503150970691	-0.000980226	2.19531863246796	0.0289675730113575	0.0503928603367421	-4.369671832	Up
KORKOLA_CORRELATED_WITH_POU5F1	0.0668128304831581	0.00190957634221545	2.19502254633153	0.0289890423671909	0.0504160790062074	-4.370303961	Up
SANSOM_APC_TARGETS_UP	0.0484072213767422	0.00683790694752558	2.19417623172394	0.029050485019178	0.0505087844577137	-4.372110347	Up
REACTOME_ORGANIC_ANION_TRANSPORT	0.161132959728071	-0.003908356	2.1919536642	0.0292123808255	0.0507760425046	-4.37685	Up

			9277	553	827	1025	
WINTER_HYPOXIA_METAGENE	0.036565628462748	0.00231623497844741	2.19074502149238	0.0293007477554624	0.0509011310714121	-4.379427081	Up
KESHELAVA_MULTIPLE_DRUG_RESISTANCE	0.0590521982302393	-0.001061321	2.18271560640972	0.0298936898535118	0.0518441276671549	-4.396505841	Up
LEE_METASTASIS_AND_RNA_PROCESSING_UP	0.120499732831695	0.010084963029594	2.17982552388471	0.0301096354581412	0.0521894742498414	-4.402638294	Up
FUNG_IL2_TARGETS_WITH_STAT5_BINDING_SITES_T1	0.11490104102529	-0.001137882	2.17507729120662	0.0304673477639069	0.0527214252679073	-4.412696505	Up
LEE_EARLY_T_LYMPHOCYTE_DN	0.0972436056492463	0.011874438419805	2.17503799540225	0.0304703233951139	0.0527214252679073	-4.412779658	Up
REACTOME_G_ALPHA_12_13_SIGNALLING_EVENTS	0.0581877800342516	0.00375016100020893	2.17496316374609	0.0304759906333577	0.0527214252679073	-4.412938002	Up
REACTOME_CTLA4_INHIBITORY_SIGNALING	0.0766641368805058	0.00450214247814415	2.17443198801836	0.0305162444228308	0.0527763525027893	-4.41406182	Up
FERRARI_RESPONSE_TO_FENRETINIDE_DN	0.143495356007088	-0.00518298	2.17279190400095	0.0306408238603652	0.0529770455992442	-4.417530102	Up
IRITANI_MAD1_TARGETS_DN	0.132648583681905	0.0083731	2.1706	0.030806	0.053233	-4.4	Up

		580512948	2026444996	4553071735	7606045729	22118574	
REACTOME_TFAP2_AP_2_FAMILY_REGULATES_TRANSCRIPTION_OF_GROWTH_FACTORS_AND_THEIR_RECEPTORS	0.0959152142542741	0.00377496521677726	2.16980655356307	0.030868715974584	0.0533265015458511	-4.423836727	Up
HUMMEL_BURKITTS_LYMPHOMA_DN	0.089380919859327	0.00412170113534611	2.16926957296689	0.0309098622269482	0.0533827253318496	-4.424970223	Up
PID_CXCR3_PATHWAY	0.071588067542097	0.00254669737994076	2.16765644606396	0.0310337532358585	0.0535817820125101	-4.428373691	Up
REACTOME_FRUCTOSE_METABOLISM	0.119274428406383	-0.006885912	2.16360696864062	0.0313466499008337	0.0540919254752501	-4.436906733	Up
GAUSSMANN_MLL_AF4_FUSION_TARGETS_A_DN	0.0437859576705811	-0.000821955	2.16277154166851	0.0314115395414536	0.0541888343340197	-4.438665223	Up
REACTOME_ION_TRANSPORT_BY_P_TYPE_ATPASES	0.0410141579707328	-0.0020935	2.15813404686559	0.0317738554820015	0.054783422493551	-4.448414747	Up
KEGG_FRUCTOSE_AND_MANNOSE_METABOLISM	0.0694320066171906	0.00639301943529288	2.1570728453118	0.0318572692317157	0.0548967435095112	-4.450642895	Up
SIG_CD40PATHWAYMAP	0.0712355034736636	0.00287102696992828	2.15561745450204	0.0319719743898727	0.0550791132494976	-4.453696977	Up

WP_COVID19_THROMBOSIS_AND_ANTICOAGULATION	0.13573402541715	-0.010005075	2.15410480489825	0.0320915693917561	0.0552698033336932	-4.456869104	Up
YIH_RESPONSE_TO_ARSENITE_C5	0.104898511487985	-0.007482458	2.15132190926788	0.0323126013495302	0.0556196108088004	-4.462699396	Up
REACTOME_HS_GAG_BIOSYNTHESIS	0.0647244854386344	-0.004616306	2.15006722264676	0.0324126831921057	0.0557764137991128	-4.465325639	Up
REACTOME_NEGATIVE_REGULATION_OF_NMDA_RECEPTOR_MEDIATED_NEURONAL_TRANSMISSION	0.0737451827857078	0.000798800891550755	2.14993094772154	0.0324235694105718	0.0557796827415241	-4.465610793	Up
SENGUPTA_NASOPHARYNGEAL_CARCINOMA_DN	0.0456790530054572	-0.003308805	2.14853821025714	0.0325350078562534	0.055955886329666	-4.468524083	Up
BIOCARTA_RACCYCD_PATHWAY	0.0730851554387795	0.0104617050760847	2.14669664127527	0.0326828653862456	0.0561790488652525	-4.472373423	Up
SAGIV_CD24_TARGETS_UP	0.0743745033042787	0.00938820402630181	2.14637891113793	0.0327084340202556	0.0562074335447748	-4.473037236	Up
SERVITJA_ISLET_HNF1A_TARGETS_UP	0.0553140493247863	-0.001043944	2.14590737794435	0.032746411361054	0.0562571202098152	-4.474022204	Up
WAGSCHAL_EHMT2_TARGETS_UP	0.080020386936323	-0.00185083	2.1451736494	0.0328055814354	0.0563294578037	-4.47555	Up

			752	102	493	4446	
UEDA_PERIFERAL_CLOCK	0.0373676613363167	0.00398295185243682	2.14516018300881	0.0328066682713958	0.0563294578037493	-4.475582563	Up
BARRIER_CANCER_RELAPSE_TUMOR_SAMPLE_DN	0.115798681438247	0.0127337819989414	2.14368882768134	0.0329256035752313	0.056502411222411	-4.478653643	Up
REACTOME_CREB1_PHOSPHORYLATION_THROUGH_THE_ACTIVATION_OF_ADENYLATE_CYCLASE	0.0850433940324909	-0.00264443	2.14040577403382	0.0331923240130341	0.0569129157869896	-4.485498836	Up
WP_TGFBETA_RECEPTOR_SIGNALING	0.0634982064256846	-0.00116424	2.1401209995843	0.0332155469132379	0.0569370062663539	-4.486092115	Up
BYSTROEM_CORRELATED_WITH_IL5_UP	0.0560160130095967	-0.00152837	2.13946206417533	0.0332693356430843	0.0570134639250757	-4.487464601	Up
WP_ACETYLCHOLINE_SYNTHESIS	0.115161401074348	0.00452482973461286	2.13912976612749	0.0332964894611515	0.0570442478844513	-4.488156585	Up
REACTOME_TRANSLOCATION_OF_SLC2A4_GLUT4_TO_THE_PLASMA_MEMBRANE	0.0666716192286688	0.00556577920680097	2.13867840885374	0.0333334027690971	0.0570759809621478	-4.489096333	Up
REACTOME_ACYL_CHAIN_REMODELLING_OF_PG	0.0755351173842095	-0.00591984	2.13773116241919	0.0334109858127193	0.0571930471427327	-4.491067922	Up
BIOCARTA_NFAT_PATHWAY	0.0586019195471483	0.0004606	2.1366	0.033496	0.057322	-4.4	Up

		13813829216	9426647852	0895073853	9190990738	93225139	
BOYLAN_MULTIPLE_MYELOMA_D_CLUSTER_DN	0.0652153596747403	-0.006067823	2.13655415222521	0.0335076037336268	0.057326818184846	-4.493516563	Up
WP_MACROPHAGE_MARKERS	0.113803840538358	0.0084226304030526	2.13479872879337	0.0336521486088881	0.0575265454187189	-4.497166106	Up
JISON_SICKLE_CELL_DISEASE_DN	0.109685409651658	0.00688019321655711	2.13342925687626	0.0337652855093711	0.0576564311297569	-4.500011238	Up
PID_HEDGEHOG_GLI_PATHWAY	0.0677660703065131	0.0100824546501506	2.1324308714249	0.0338479716156308	0.0577658399280232	-4.502084309	Up
REACTOME_GABA_RECEPTOR_ACTIVATION	0.0685608018464423	-0.00635377	2.13036503451455	0.0340196167277389	0.0580109233596361	-4.506370882	Up
REACTOME_SURFACTANT_METABOLISM	0.0843287238489903	-0.005115016	2.12901905422937	0.0341318528425278	0.0581863253484125	-4.509161603	Up
REACTOME_BASIGIN_INTERACTIONS	0.0675890403532664	-0.004758758	2.12595998099783	0.034388121157109	0.0585910151035343	-4.515497861	Up
WP_IL4_SIGNALING_PATHWAY	0.0937228336355281	0.00847681974234194	2.11663230290226	0.0351797587670575	0.0598904999087015	-4.534763855	Up

ZHAN_MULTIPLE_MYELOMA_CD2_UP	0.0632472550623167	0.00703894986249717	2.11457751137263	0.0353562352908406	0.0601579365269319	-4.538996935	Up
PID_HIF1_TFPATHWAY	0.0653919615340977	0.00506641277836615	2.11389437470816	0.0354150747219406	0.060241537078401	-4.540403385	Up
KEGG_STARCH_AND_SUCROSE_METABOLISM	0.0738354927965216	0.0101108743785141	2.11203748368163	0.0355754357881621	0.0604874946571981	-4.544224152	Up
WP_THYROXINE_THYROID_HORMONE_PRODUCTION	0.120753134144338	-0.008059567	2.11127558290279	0.0356414134965936	0.0605638725926379	-4.54579091	Up
REACTOME_TRANSPORT_TO_THE_GOLGI_AND_SUBSEQUENT_MODIFICATION	0.041856173227228	0.00458643822014796	2.11125035163386	0.0356436002224757	0.0605638725926379	-4.545842786	Up
LOPES_METHYLATED_IN_COLON_CANCER_DN	0.0712134659052569	-0.002057913	2.10947550652158	0.0357977102458399	0.060809082511201	-4.549490379	Up
PID_BETA_CATENIN_DEG_PATHWAY	0.0765857618883754	0.0111633182252215	2.10853843454205	0.035879306537226	0.0609310138649963	-4.551415015	Up
HANN_RESISTANCE_TO_BCL2_INHIBITOR_DN	0.0662887763677957	-0.005618777	2.10815942297223	0.0359123545357049	0.0609704553071992	-4.552193225	Up
REACTOME_FGFR2B_LIGAND_BINDING_AND_ACTIVATION	0.101900566528835	-0.00082345	2.1053589602	0.0361573512788	0.0613025619742	-4.55793	Up

			4069	526	797	9113	
REACTOME_HSP90_CHAPERONE_CYCLE_FOR_STEROID_HORMONE_RECEPTORS_SHR_	0.0530453278594262	0.00294417014148497	2.10519764349998	0.0361715075041562	0.0613098162420255	-4.558269871	Up
CHANDRAN_METASTASIS_TOP50_DN	0.0587444432461112	0.00666557064979491	2.10384395422288	0.0362904867416368	0.0614946904737481	-4.561044465	Up
BIOCARTA_SHH_PATHWAY	0.0879149673240437	-0.000705921	2.09894081176304	0.0367242478487303	0.062174713521788	-4.571079741	Up
REACTOME_RELEASE_OF_HH_NP_FROM_THE_SECRETING_CELL	0.124258733531593	-0.016232639	2.0932316534887	0.0372349018805514	0.0629575145662169	-4.58273612	Up
WP_PLATELETMEDIATED_INTERACTIONS_WITH_VASCULAR_AND_CIRCULATING_CELLS	0.102882598536732	2.19494455634452e-05	2.09252269994082	0.0372987362446583	0.0630311069617736	-4.584181443	Up
BILANGES_SERUM_SENSITIVE_GENES	0.0402674240253594	-0.000403463	2.09038801335497	0.0374915098432462	0.0633223949923888	-4.588530503	Up
YAO_TEMPORAL_RESPONSE_TO_PROGESTERONE_CLUSTER_14	0.0760879317725887	0.00234336541214426	2.08623656856636	0.0378688492393836	0.0638728117469712	-4.596976051	Up
TORCHIA_TARGETS_OF_EWSR1_FLI1_FUSION_TOP20_UP	0.0743968255850074	-0.00178246	2.08166120673981	0.0382884775896394	0.0644929661869449	-4.606265161	Up
HOLLERN_SQUAMOUS_BREAST_TUMOR	0.0531640820414392	-0.0035231	2.0814	0.038309	0.064510	-4.6	Up

		5	333401 3232	4798219 423	8359345 62	0672 7269	
KEGG_OTHER_GLYCAN_DEGRADATION	0.113910666364165	0.0011998 864553148	2.0751 434652 8751	0.038893 1105448 009	0.065458 1174489 097	-4.6 1946 3653	Up
REACTOME_NUCLEAR_EVENTS_KINASE_AND_TRANSCRIPTION_FACTOR_ACTIVATION_	0.0601809060852136	0.0028143 999823982	2.0696 803493 8936	0.039406 1732775 833	0.066267 7099793 985	-4.6 3049 5618	Up
REACTOME_SIGNALING_BY_PTK6	0.0534441035254305	0.0022559 560551277 4	2.0678 055323 3795	0.039583 5710899 528	0.066529 9826036 656	-4.6 3427 5041	Up
REACTOME_ACTIVATION_OF_ANTERIOR_HOX_GENES_IN_HINDBRAIN_DEVELOPMENT_DURING_EARLY_EMBRYOGENESIS	0.0519756682594007	0.0045606 889099344 7	2.0671 124804 2447	0.039649 3209373 04	0.066622 4512879 929	-4.6 3567 1317	Up
ABBUD_LIF_SIGNALING_2_DN	0.123751552647575	-0.0004202 96	2.0654 386812 0933	0.039808 4987151 357	0.066853 7206506 621	-4.6 3904 161	Up
REACTOME_THE_RETINOID_CYCLE_IN_CONES_DAYLIGHT_VISION_	0.137629496291423	-0.0146910 48	2.0636 702924 1539	0.039977 2638775 81	0.067118 9821174 318	-4.6 4259 9489	Up
HASLINGER_B_CLL_WITH_CHROMOSOME_12_TRISOMY	0.0810498082189486	0.0053907 744563202 9	2.0619 003039 9791	0.040146 7924010 593	0.067385 3814043 74	-4.6 4615 7628	Up
DAVICIONI_TARGETS_OF_PAX_FOXO1_FUSIONS_DN	0.0476890100164543	0.0030465 44345157	2.0613 673821 1101	0.040197 9552960 249	0.067434 7860871 423	-4.6 4722 8361	Up

SHETH_LIVER_CANCER_VS_TXNIP_LOSS_PAM4	0.0227032712409457	-0.000964685	2.06089613880836	0.0402432430781985	0.0674742868142566	-4.648174947	Up
LOPEZ_EPITHELIOID_MESOTHELIOMA	0.0905032222580345	-0.003238493	2.06024838725957	0.0403055647838105	0.0675532742733249	-4.649475743	Up
CHEOK_RESPONSE_TO_MERCAPTOPURINE_AND_HD_MTX_UP	0.127436540552315	0.00202052599089462	2.06018030567704	0.0403121198499107	0.0675532742733249	-4.649612439	Up
REACTOME_MATURATION_OF_PROTEIN_3A	0.0966288007732295	0.000564145617022548	2.05748769979095	0.040572100540041	0.0679705878682954	-4.655015229	Up
ZWANG_EGF_INTERVAL_DN	0.0422095161103433	0.00193238244158413	2.0567583152209	0.0406427707181801	0.0680706092411613	-4.6564758	Up
VANDESLUIS_COMMD1_TARGETS_GROUP_3_DN	0.0696063968923436	-0.00475401	2.05639945562718	0.0406775791645868	0.06811052977464	-4.657196877	Up
KEGG_PROXIMAL_TUBULE_BICARBONATE_RECLAMATION	0.0676033192139484	-0.002177129	2.0562754318356	0.0406896150554893	0.0681123086972551	-4.657445442	Up
REACTOME_TERMINAL_PATHWAY_OF_COMPLEMENT	0.108715767268879	-0.005783099	2.05519238832483	0.0407948480564613	0.0682700517353614	-4.659615427	Up
PROVENZANI_METASTASIS_DN	0.0485725539598102	-0.001050106	2.0497585248	0.0413263318783	0.0691408468918	-4.67048	Up

			3918	096	241	5974	
REACTOME_LIPID_PARTICLE_ORGANIZATION	0.117002231823348	-0.007275207	2.04609894447081	0.0416875870607055	0.0696513735897172	-4.677791307	Up
WP_TOLLLIKE_RECEPTOR_SIGNALING_RELATED_TO_MYD88	0.0904485966021552	0.00251713340586015	2.04464990784216	0.0418313696339916	0.0698727963719554	-4.680680405	Up
REACTOME_GRB2_SOS_PROVIDES_LINKAGE_TO_MAPK_SIGNALING_FOR_INTEGRINS_	0.104743644565231	-0.004183466	2.04403451941456	0.0418925599161625	0.0699561795533013	-4.68190677	Up
REACTOME_SYNTHESIS_OF_PC	0.0642938008866063	0.00177776103027751	2.04370137272551	0.0419257176546192	0.0699738987583278	-4.682570525	Up
REACTOME_REGULATION_OF_GENE_EXPRESSION_IN_LATE_STAGE_BRANCHING_MORPHOGENESIS_PANCREATIC_BUD_PRECURSOR_CELLS	0.10119677607163	0.00691220926265516	2.04313663265922	0.0419819766512571	0.0700489594285894	-4.683695464	Up
WESTON_VEGFA_TARGETS_12HR	0.0831497164028468	-0.009679528	2.04100654110099	0.0421947532247397	0.0703850667202256	-4.687935802	Up
WONG_ENDMETRIUM_CANCER_UP	0.0850648433444782	-0.007057888	2.04070132676575	0.0422253163429166	0.0704171248093722	-4.688543035	Up
YAGUE_PRETUMOR_DRUG_RESISTANCE_UP	0.109175544611109	0.00205530322566423	2.03743302861849	0.0425537746276761	0.0708886954143816	-4.695039897	Up
REACTOME_TP53_REGULATES_TRANSCRIPTION_OF_G	0.0838013073638266	-0.0068647	2.0342	0.042874	0.071368	-4.7	Up

ENES_INVOLVED_IN_G1_CELL_CYCLE_ARREST		91	6360911023	368082462	063811219	01330554	
REACTOME_HORMONE_LIGAND_BINDING_RECEPTORS	0.103471913980746	-0.00772687	2.03402947027298	0.0428981329329412	0.071385713435594	-4.701794895	Up
FRASOR_TAMOXIFEN_RESPONSE_UP	0.0516201573832701	0.00309594676048079	2.03290934690119	0.0430119792524926	0.0715368047213884	-4.704015593	Up
BIOCARTA_IGF1R_PATHWAY	0.077744388425271	0.00413695261129568	2.02984750604965	0.0433244874136397	0.0719986863123057	-4.710079781	Up
REACTOME_SIGNALING_BY_VEGF	0.055201242754479	0.00662979518335386	2.02941269687912	0.0433690224284501	0.0720534053033698	-4.710940232	Up
WILLIAMS_ESR1_TARGETS_UP	0.0752767670541701	-0.000480197	2.02779577892107	0.043534975409987	0.0722904206446734	-4.714138406	Up
PID_ATM_PATHWAY	0.0668769171500331	0.00473848896271166	2.02592531065963	0.0437276241084209	0.0725520884365058	-4.717835003	Up
DE_YY1_TARGETS_UP	0.0759125928418428	0.000931426379814303	2.02531109552473	0.0437910428003473	0.0726378948855574	-4.719048151	Up
MIKKELSEN_MCV6_ICP_WITH_H3K4ME3_AND_H3K27ME3	0.0716555561859595	0.000944845960707159	2.02374821017657	0.0439527654433554	0.0728672048896653	-4.722133427	Up

BIOCARTA_FOSB_PATHWAY	0.132090998643741	-0.004007119	2.02229751345099	0.0441033325575989	0.0730777856874042	-4.724995163	Up
ROZANOV_MMP14_TARGETS_UP	0.0318734713233488	0.00147187391658178	2.02164355965195	0.044171349117273	0.0731709538219678	-4.726284542	Up
DARWICHE_PAPILLOMA_PROGRESSION_RISK	0.0393836105895725	0.00125637739461188	2.01933234691737	0.0444124478746039	0.0735507107677871	-4.730838241	Up
PID_TCR_JNK_PATHWAY	0.0919934014932534	0.00489836328372345	2.01403429008458	0.0449693410692767	0.0744134097619304	-4.741257731	Up
REACTOME_SIGNALING_BY_RHO_GTPASES	0.0408339911287654	0.00405280273176703	2.013549863	0.0450205546365142	0.0744783002742121	-4.742209109	Up
REACTOME_PEPTIDE_LIGAND_BINDING_RECEPTORS	0.0581001955600164	-0.003065825	2.01283855437833	0.0450958437289468	0.0745829741608241	-4.743605664	Up
REACTOME_SIGNALING_BY_MAPK_MUTANTS	0.118787292305427	0.0265763569535311	2.01263200254499	0.0451177263727817	0.0745992881182354	-4.744011111	Up
ALONSO_METASTASIS_EMT_DN	0.122779246420182	-0.005615296	2.01216247085143	0.045167503282144	0.0746617024959434	-4.744932615	Up
BIOCARTA_GABA_PATHWAY	0.0932986170279953	-6.29E-05	2.0102353280	0.0453722951841	0.0749602971008	-4.74871	Up

			9409	133	228	2647	
REACTOME_ACTIVATION_OF_NMDA_RECEPTORS_AND_POSTSYNAPTIC_EVENTS	0.049933889219586	-0.001386885	2.0056419986 9134	0.0458635899245 708	0.0756913859776 152	-4.757708144	Up
WP_MAMMARY_GLAND_DEVELOPMENT_PATHWAY_EMBRYONIC_DEVELOPMENT_STAGE_1_OF_4	0.0700030013797089	0.00263010233222666	2.00533013524822	0.0458971090254 409	0.0757265698354 363	-4.758318167	Up
REACTOME_P75NTR_NEGATIVELY_REGULATES_CELL_CYCLE_VIA_SC1	0.109677173684563	0.0104280418685507	2.0050721957 093	0.0459248480556 232	0.0757522008719 779	-4.758822643	Up
REACTOME_INTERLEUKIN_2_FAMILY_SIGNALING	0.0753496217144911	0.00776089602010022	2.00156323612711	0.0463036169111 053	0.0763364011068 342	-4.765679171	Up
REACTOME_SUMOYLATION_OF_RNA_BINDING_PROTEINS	0.0848196526597124	0.00974821662828088	2.00071680414927	0.0463953784644 695	0.0764673696571 859	-4.767331358	Up
REACTOME_ERBB2_REGULATES_CELL_MOTILITY	0.0871535402770831	-0.006319801	2.0001713855 011	0.0464545887186 621	0.0765446329112 651	-4.768395625	Up
HERNANDEZ_ABERRANT_MITOSIS_BY_DOCETACEL_2NM_UP	-0.050336827	0.00143058830419931	-2.002878594	0.0461613240339 49	0.0761220346117 751	-4.76311032	Down
RUIZ_TNC_TARGETS_UP	-0.048455385	0.00497949992144643	-2.007756971	0.0456368192091 622	0.0753371640508 696	-4.753568701	Down
REACTOME_VITAMIN_B5_PANTOTHENATE_METABOLI	-0.065908592	0.0035960	-2.0095	0.045444	0.075039	-4.7	Down

SM		4179223677	58055	4536633693	5647482132	50040263	
ST_PHOSPHOINOSITIDE_3_KINASE_PATHWAY	-0.063562429	0.00776444276655567	-2.009756865	0.045423261884013	0.0750245307381769	-4.749650594	Down
DARWICHE_SKIN_TUMOR_PROMOTER_UP	-0.03681563	-0.000623225	-2.011300641	0.0452589903806863	0.0747930120588178	-4.746623502	Down
DOANE_BREAST_CANCER_CLASSES_DN	-0.077103302	0.00146898535372119	-2.018467483	0.0445029544353213	0.0736612901813438	-4.73254095	Down
PID_RANBP2_PATHWAY	-0.100317558	0.013648722483223	-2.018758919	0.0444724387122409	0.0736304153339236	-4.731967263	Down
DACOSTA_UV_RESPONSE_VIA_ERCC3_COMMON_UP	-0.050270957	-0.00106599	-2.022327532	0.0441002125267186	0.0730777856874042	-4.724935966	Down
VILLANUEVA_LIVER_CANCER_KRT19_UP	-0.06546928	0.00136050461975486	-2.023949869	0.0439318699356935	0.0728520215578011	-4.721735464	Down
HUMMERICH_SKIN_CANCER_PROGRESSION_UP	-0.067954035	0.00331742912966044	-2.026428713	0.0436757051363902	0.0724853213319716	-4.716840457	Down
PID_ATR_PATHWAY	-0.089862462	0.00480990765218774	-2.026805982	0.0436368295000728	0.0724401713578369	-4.71609495	Down

BRACHAT_RESPONSE_TO_CISPLATIN	-0.072817309	0.00378403614606272	-2.027924185	0.0435217767586692	0.0722878427458014	-4.713884517	Down
VALK_AML_CLUSTER_5	-0.140750354	0.0079208946919494	-2.030047038	0.0433040635141328	0.0719840177376064	-4.709684865	Down
WU_HBX_TARGETS_2_DN	-0.088612349	0.00407374986309365	-2.031441665	0.0431615389919092	0.0717663199900296	-4.706923545	Down
REACTOME_FANCONI_ANEMIA_PATHWAY	-0.087874578	4.02217430962178e-07	-2.033130354	0.0429894963696761	0.0715185751719592	-4.70357753	Down
IKEDA_MIR133_TARGETS_UP	-0.094480549	0.0162168363968557	-2.034247245	0.0428760286695724	0.071368063811219	-4.701363009	Down
SA_CASPASE_CASCADE	-0.077202517	0.00610666391529945	-2.035082283	0.0427913614407506	0.0712653556379766	-4.699706561	Down
TONKS_TARGETS_OF_RUNX1_RUNX1T1_FUSION_SUSTAINDED_IN_ERYTHROCYTE_UP	-0.061216546	0.000191064554161237	-2.038041748	0.042492435288761	0.0708055156610308	-4.693830624	Down
RUAN_RESPONSE_TO_TROGLITAZONE_DN	-0.085190064	-0.003802855	-2.038327183	0.0424636985320163	0.0707766317906083	-4.693263463	Down
BIOCARTA_PML_PATHWAY	-0.084102282	-0.001047431	-2.039054313	0.0423905677830	0.0706737185682	-4.69181	Down

				738	35	8301	
VALK_AML_CLUSTER_16	-0.059086149	-0.002335884	-2.043841541	0.0419117641511191	0.0699694244448619	-4.68229127	Down
HOLLMANN_APOPTOSIS_VIA_CD40_UP	-0.047401382	0.0056207300155896	-2.047691866	0.0415300129333665	0.0694067825194954	-4.674613039	Down
HWANG_PROSTATE_CANCER_MARKERS	-0.065543063	0.000106087636455753	-2.048928037	0.0414080786900922	0.0692216386828447	-4.672144922	Down
RICKMAN_METASTASIS_UP	-0.072980771	0.00110397228320609	-2.04948714	0.0413530298200432	0.0691482370940216	-4.671028154	Down
MARIADASON_RESPONSE_TO_BUTYRATE_CURCUMIN_SULINDAC_TSA_1	-0.117452253	-0.000988005	-2.049613902	0.0413405576016408	0.0691460094404162	-4.670774915	Down
TSAI_RESPONSE_TO_IONIZING_RADIATION	-0.038848633	0.00127889115737652	-2.061027662	0.0402305989154606	0.0674713124745377	-4.647910777	Down
WP_INTRACELLULAR_TRAFFICKING_PROTEINS_INVOLVED_IN_CMT_NEUROPATHY	-0.078508089	0.00427153976544275	-2.061703349	0.040165694502257	0.0673988823399592	-4.646553377	Down
HELLER_SILENCED_BY_METHYLATION_DN	-0.039251813	0.0016040560238373	-2.065889205	0.0397656005331472	0.0667997516939769	-4.638134714	Down
GENTILE_UV_RESPONSE_CLUSTER_D5	-0.076910874	0.0115517	-2.0681	0.039552	0.066496	-4.6	Down

		862754736	31557	6733373 634	0572602 965	3361 8051	
BOHN_PRIMARY_IMMUNODEFICIENCY_SYNDROM_DN	-0.074587637	0.0104272 05588704	-2.0711 97929	0.039263 0751759 406	0.066044 9614138 383	-4.6 2743 3915	Down
PHONG_TNF_RESPONSE_VIA_P38_PARTIAL	-0.055334138	0.0044805 326853046 3	-2.0748 6016	0.038919 5759115 772	0.065484 9031399 185	-4.6 2003 644	Down
REACTOME_SIGNALING_BY_INTERLEUKINS	-0.040394151	0.0022995 198675827 6	-2.0782 58866	0.038603 0934943 902	0.064987 6325792 459	-4.6 1315 9939	Down
MAEKAWA_ATF2_TARGETS	-0.068092491	0.0008587 072282481 01	-2.0825 67304	0.038205 0608784 944	0.064369 9274899 063	-4.6 0442 7129	Down
PID_WNT_NONCANONICAL_PATHWAY	-0.061088543	0.0075839 306875810 3	-2.0831 99746	0.038146 9293484 858	0.064289 4353695 497	-4.6 0314 3752	Down
ZHAN_MULTIPLE_MYELOMA_SUBGROUPS	-0.115958411	0.0034433 715685159 4	-2.0845 09914	0.038026 7446681 267	0.064104 2922745 96	-4.6 0048 3906	Down
CHEMNITZ_RESPONSE_TO_PROSTAGLANDIN_E2_UP	-0.064943624	-0.0029911 67	-2.0853 11188	0.037953 4015886 511	0.063998 0341376 683	-4.5 9885 6395	Down
KENNY_CTNNB1_TARGETS_DN	-0.064459109	0.0059348 794965544 4	-2.0866 00398	0.037835 6501232 978	0.063834 1615426 229	-4.5 9623 6539	Down

BERENJENO_TRANSFORMED_BY_RHOA_FOREVER_UP	-0.084406899	-0.001160524	-2.087741314	0.037731704066182	0.063676097645131	-4.593916731	Down
BIOCARTA_ERBB3_PATHWAY	-0.135922626	-0.007505424	-2.089416624	0.0375795133454479	0.0634365078420437	-4.59050812	Down
ZHAN_V1_LATE_DIFFERENTIATION_GENES_UP	-0.077571637	0.00418228749266192	-2.089420724	0.0375791415156475	0.0634365078420437	-4.590499774	Down
REACTOME_REGULATION_OF_FOXO_TRANSCRIPTIONAL_ACTIVITY_BY_ACETYLATION	-0.114433008	-0.001777033	-2.090975294	0.0374383904186181	0.0632498882222	-4.587334446	Down
REACTOME_COMPLEMENT_CASCADE	-0.069559417	0.00250982568639551	-2.092643175	0.0372878820334732	0.0630299247771699	-4.583935867	Down
REACTOME_SIGNALING_BY_WNT_IN_CANCER	-0.059523088	0.0125432102459442	-2.094531373	0.0371181176415988	0.0627771542783116	-4.580085188	Down
OKUMURA_INFLAMMATORY_RESPONSE_LPS	-0.041365753	0.00502504271289946	-2.097124062	0.0368860919297943	0.0624017368787771	-4.574792331	Down
LINDSTEDT_DENDRITIC_CELL_MATURATION_D	-0.070729635	0.00777679408901253	-2.097214843	0.036877990266332	0.0624017368787771	-4.57460689	Down
REACTOME_ROS_AND_RNS_PRODUCTION_IN_PHAGOCYTES	-0.09350297	0.0009782511791474	-2.098855174	0.0367318631358	0.0621747135217	-4.57125	Down

		07		783	88	4814	
PID_ERBB1_DOWNSTREAM_PATHWAY	-0.067729579	0.00593499831782303	-2.100875159	0.0365525964989044	0.061905038599918	-4.567123414	Down
REACTOME_MET_ACTIVATES_RAS_SIGNALING	-0.079426608	0.00440505775707818	-2.102195267	0.0364358469126781	0.0617241544178474	-4.564421368	Down
REACTOME_TRAF6_MEDIATED_IRF7_ACTIVATION_IN_TLR7_8_OR_9_SIGNALING	-0.083543075	0.00355732028017311	-2.106090871	0.0360931826674114	0.0612104876548149	-4.556438119	Down
REACTOME_SIGNALING_BY_NOTCH2	-0.071350994	0.00218471134600349	-2.106853303	0.0360264419657381	0.0611139998035901	-4.554873995	Down
REACTOME_DEADENYLATION_DEPENDENT_MRNA_DECAY	-0.102299801	0.000711826899489993	-2.107114672	0.036003587007061	0.0610919257935559	-4.554337672	Down
KEGG_PURINE_METABOLISM	-0.04983473	0.0002257726832356	-2.10739747	0.0359788721410747	0.0610666829039241	-4.553757304	Down
ALCALAY_AML_BY_NPM1_LOCALIZATION_UP	-0.046500471	0.00340142740789395	-2.111994451	0.0355791594962833	0.0604874946571981	-4.544312659	Down
REACTOME_NEF_MEDIATED_DOWNREGULATION_OF_MHC_CLASS_I_COMPLEX_CELL_SURFACE_EXPRESSION	-0.100926671	0.00860250703527525	-2.116511388	0.0351901226479562	0.0598917168291375	-4.535013063	Down
REACTOME_ACTIVATION_OF_NIMA_KINASES_NEK9_N	-0.118393043	0.0039918	-2.1179	0.035067	0.059715	-4.5	Down

EK6_NEK7		0818014106	48391	1236176674	1265555176	32050488	
ABE_VEGFA_TARGETS	-0.08311099	0.00262476872386545	-2.123894913	0.0345620514978 23	0.058871 2002324333	-4.519770252	Down
TONKS_TARGETS_OF_RUNX1_RUNX1T1_FUSION_ERYTHROCYTE_UP	-0.059050937	0.00168258174996476	-2.126130808	0.0343737669873687	0.0585826391242716	-4.51514426	Down
RAFFEL_VEGFA_TARGETS_DN	-0.137978061	0.0015919506319743	-2.131810948	0.0338994011375469	0.0578218144712156	-4.503371061	Down
WP_CANCER_IMMUNOTHERAPY_BY_CTLA4_BLOCKADE	-0.090696482	0.0116950713576538	-2.132188216	0.0338680945675128	0.0577842943871776	-4.502588023	Down
LASTOWSKA_COAMPLIFIED_WITH_MYCN	-0.070293779	-4.97E-05	-2.132925432	0.0338069904422241	0.0577117683374272	-4.501057508	Down
SUNG_METASTASIS_STROMA_DN	-0.075092339	-0.003454689	-2.133430282	0.0337652006607347	0.0576564311297569	-4.500009108	Down
GENTILE_UV_LOW_DOSE_UP	-0.071420745	0.00131989602391066	-2.133442511	0.0337641889410509	0.0576564311297569	-4.499983709	Down
LEE_LIVER_CANCER_E2F1_UP	-0.06527398	-0.006570813	-2.13403887	0.0337148829000599	0.0576179179957797	-4.498744961	Down

SLEBOS_HEAD_AND_NECK_CANCER_WITH_HPV_UP	-0.08175533	-5.09E-05	-2.13627298	0.0335307200318678	0.05733476012060 7	-4.494101319	Down
BIOCARTA_CK1_PATHWAY	-0.083173211	0.00629026354121682	-2.136340514	0.0335251665101827	0.05733476012060 7	-4.493960874	Down
PID_AURORA_B_PATHWAY	-0.071467075	-0.002991996	-2.13868383	0.0333329591763247	0.0570759809621478	-4.489085046	Down
LEE_TARGETS_OF_PTCH1_AND_SUFU_UP	-0.054265069	-0.000659863	-2.141441599	0.0331079719554145	0.0567839684794854	-4.483340229	Down
REACTOME_NUCLEOTIDE_BINDING_DOMAIN_LEUCINE_RICH_REPEAT_CONTAINING_RECEPTOR_NLR_SIGNALING_PATHWAYS	-0.061699758	0.00101070726613103	-2.142249404	0.0330423166359063	0.0566870258040549	-4.481656101	Down
WP_ATR_SIGNALING	-0.126540792	0.00292848095286052	-2.144823001	0.0328338913076594	0.056360609332589	-4.476286526	Down
LU_EZH2_TARGETS_DN	-0.05967998	0.00728577990155831	-2.147807544	0.0325936031955875	0.056041134358729	-4.470051741	Down
BIOCARTA_HSP27_PATHWAY	-0.079771968	-0.000351875	-2.151345731	0.0323107037507485	0.0556196108088004	-4.46264952	Down
REACTOME_ADVANCED_GLYCOSYLATION_ENDPRODUCT_RECEPTOR_SIGNALING	-0.084820504	-0.001003922	-2.157726603	0.0318058595580	0.0548233741396	-4.44927	Down

				743	743	0361	
XU_CREBBP_TARGETS_UP	-0.090656157	0.00932675829280424	-2.161985623	0.0314726894872436	0.0542792396908367	-4.440318904	Down
FRIDMAN_SENESCENCE_UP	-0.059047607	-0.001691458	-2.165053289	0.0312345840554617	0.0539135326007371	-4.433860824	Down
WP_SIGNALING_OF_HEPATOCYTE_GROWTH_FACTOR_RECEPTOR	-0.086244069	0.00878283081181197	-2.171538651	0.0307363156863089	0.0531273493358171	-4.42017865	Down
REACTOME_CYTOKINE_SIGNALING_IN_IMMUNE_SYSTEM	-0.036929347	0.000384661157452451	-2.174997909	0.030473359121512	0.0527214252679073	-4.41286448	Down
GENTILE_UV_RESPONSE_CLUSTER_D2	-0.091778233	0.00709923330778319	-2.177520861	0.0302828034806036	0.052445692300253	-4.407522921	Down
MATZUK_CUMULUS_EXPANSION	-0.109897757	-0.004471591	-2.177669468	0.030271611533012	0.0524409413300043	-4.407208106	Down
REACTOME_DEPOLYMERISATION_OF_THE_NUCLEAR_LAMINA	-0.071976838	0.00390041134000031	-2.178945938	0.0301756246372679	0.05228925240746	-4.404503124	Down
FERRANDO_T_ALL_WITH_MLL_ENL_FUSION_DN	-0.06063639	0.00174015960430262	-2.180771555	0.0300388005225461	0.0520812387830849	-4.400631778	Down
FOURNIER_ACINAR_DEVELOPMENT_EARLY_UP	-0.077205195	0.0057337	-2.1832	0.029857	0.051795	-4.3	Down

		3925411468	01643	5051913911	845366955	95473751	
MATZUK_SPERMATOGONIA	-0.084618271	-0.006995304	-2.186843225	0.0295875978874133	0.0513419681540884	-4.387733861	Down
DAZARD_UV_RESPONSE_CLUSTER_G6	-0.078360611	0.00958075663124996	-2.187175613	0.0295630672780938	0.0513137468107181	-4.387026775	Down
LI_WILMS_TUMOR_VS_FETAL_KIDNEY_1_DN	-0.084142882	0.00350775358986164	-2.189298629	0.0294068009892235	0.0510567870601707	-4.382508064	Down
BACOLOD_RESISTANCE_TO_ALKYLATING_AGENTS_UP	-0.087036635	-0.000297546	-2.189615691	0.0293835248496546	0.0510306487805837	-4.381832854	Down
COLIN_PILOCYTIC_ASTROCYTOMA_VS_GLIOBLASTOMA_DN	-0.076105966	0.00504158570320851	-2.191604545	0.0292378820919026	0.0508061405779506	-4.377595265	Down
LOPEZ_MESOTHELIOMA_SURVIVAL_UP	-0.08785759	0.00344814702040419	-2.196828073	0.0288583364928597	0.050230985589226	-4.366447971	Down
REACTOME_PI3K_EVENTS_IN_ERBB2_SIGNALING	-0.085218888	-0.002116749	-2.196934365	0.0288506576902017	0.050230985589226	-4.366220871	Down
KIM_RESPONSE_TO_TSA_AND_DECITABINE_UP	-0.04834402	-0.005894745	-2.197768118	0.0287904867588337	0.0501410076633224	-4.364439142	Down

NEMETH_INFLAMMATORY_RESPONSE_LPS_DN	-0.066364129	0.0054462712737869 9	-2.1986 11934	0.028729 7002745 378	0.050049 1859680 202	-4.3 6263 5245	Down
CHYLA_CBFA2T3_TARGETS_UP	-0.042934239	0.0021866827434237 5	-2.1986 59052	0.028726 3093273 098	0.050049 1859680 202	-4.3 6253 4498	Down
REACTOME_GASTRIN_CREB_SIGNALLING_PATHWAY_VIA_PKC_AND_MAPK	-0.076430053	0.0019640955243332 4	-2.1998 45287	0.028641 0523638 482	0.049922 7778776 764	-4.3 5999 7398	Down
REACTOME_SIGNALING_BY_FGFR1	-0.056430008	0.0006440433307723 7	-2.2006 34913	0.028584 4220171 708	0.049852 0672831 074	-4.3 5830 7827	Down
BIOCARTA_EFP_PATHWAY	-0.092 02392 4	-0.0024574 65	-2.2007 46923	0.028576 3967250 156	0.049852 0672831 074	-4.3 5806 811	Down
REACTOME_MICRORNA_MIRNA_BIOGENESIS	-0.084 16912 3	0.0057085600310186 4	-2.2069 93787	0.028131 9005303 978	0.049131 8814271 747	-4.3 4468 0346	Down
MARSON_FOXP3_TARGETS_DN	-0.085 11591 5	0.0058222837764392 4	-2.2078 81777	0.028069 2043554 491	0.049036 1810960 52	-4.3 4277 4306	Down
SESTO_RESPONSE_TO_UV_C5	-0.095 02272 1	0.0161737660278367	-2.2083 48396	0.028036 3074517 668	0.048992 5000994 135	-4.3 4177 2427	Down
BHATI_G2M_ARREST_BY_2METHOXYESTRADIOL_DN	-0.045130185	0.0016461209765294	-2.2102 08377	0.027905 5096338	0.048791 4079711	-4.3 3777	Down

		6		984	57	6828	
REACTOME_GLYCOGEN_SYNTHESIS	-0.081062907	-0.0003539 49	-2.2127 21208	0.027729 6422196 233	0.048497 5737551 991	-4.3 3237 3629	Down
REACTOME_RUNX1_REGULATES_EXPRESSION_OF_C OMPONENTS_OF_TIGHT_JUNCTIONS	-0.132346513	-0.0139388 06	-2.2137 50595	0.027657 8756636 116	0.048399 3330262 299	-4.3 3015 8485	Down
LASTOWSKA_NEUROBLASTOMA_COPY_NUMBER_DN	-0.052207357	0.0048469 444109650 4	-2.2154 06455	0.027542 7706210 447	0.048238 7068975 238	-4.3 2659 3143	Down
ZHENG_GLIOBLASTOMA_PLASTICITY_UP	-0.047918146	-0.0050679 13	-2.2159 32509	0.027506 2897586 433	0.048188 4111012 981	-4.3 2545 9925	Down
WANG_RESPONSE_TO_GSK3_INHIBITOR_SB216763_DN	-0.058077271	-0.0019863 9	-2.2180 11913	0.027362 4966887 708	0.047977 1234314 125	-4.3 2097 7954	Down
PHESSE_TARGETS_OF_APC_AND_MBD2_UP	-0.07947525	0.0037543 011635720 7	-2.2183 32801	0.027340 3651127 225	0.047951 8638752 948	-4.3 2028 5948	Down
CHUNG_BLISTER_CYTOTOXICITY_UP	-0.110530325	0.0036890 786623577 2	-2.2184 74481	0.027330 5983811 969	0.047948 2826885 498	-4.3 1998 0378	Down
BLANCO_MELO_COVID19_SARS_COV_2_INFECTION_A 594_CELLS_UP	-0.061894675	0.0007793 594534587 7	-2.2197 40587	0.027243 4537722 28	0.047822 4314378 448	-4.3 1724 8864	Down
IM_SREBF1A_TARGETS	-0.122526314	0.0110218	-2.2201	0.027215	0.047813	-4.3	Down

		234177168	53312	0985450684	2229462892	1635812	
ZHANG_RESPONSE_TO_CANTHARIDIN_UP	-0.117793103	0.0140326343767431	-2.22017214	0.0272138056592725	0.0478132229462892	-4.316317483	Down
RICKMAN_TUMOR_DIFFERENTIATED_WELL_VS_POORLY_DN	-0.053194975	0.0015194552186449	-2.221442403	0.027126699644611	0.0476984205932296	-4.313574975	Down
REACTOME_ADP_SIGNALLING_THROUGH_P2Y_PURINOCEPTOR_12	-0.080543123	-0.003001957	-2.22292406	0.02702540341335	0.0475607255420084	-4.310374154	Down
REACTOME_VITAMIN_B1_THIAMIN_METABOLISM	-0.129204364	0.00510943581531141	-2.225976987	0.0268177191252225	0.0472488170906205	-4.303772432	Down
REACTOME_B_WICH_COMPLEX_POSITIVELY_REGULATES_RRNA_EXPRESSION	-0.09376014	-0.002218122	-2.228302617	0.0266604424661061	0.0470117518145229	-4.29873757	Down
WP_CANONICAL_AND_NONCANONICAL_TGFB_SIGNALING	-0.092109406	0.00761766830750906	-2.230007576	0.0265456496711633	0.0468492600252802	-4.295043207	Down
REACTOME_FREE_FATTY_ACIDS_REGULATE_INSULIN_SECRETION	-0.116355943	0.00266067763429457	-2.233734571	0.0262962095404181	0.0464354402894382	-4.286957937	Down
RUAN_RESPONSE_TO_TNF_UP	-0.126122433	-0.0051355	-2.245046328	0.0255515665267708	0.0452105397467692	-4.262338726	Down

MIZUKAMI_HYPOXIA_UP	-0.121061454	-0.001690817	-2.248645766	0.0253184972735854	0.0448109246014 1	-4.254479674	Down
BREUHAHN_GROWTH_FACTOR_SIGNALING_IN_LIVER_CANCER	-0.082635209	0.00389391849087723	-2.251895862	0.0251096398279247	0.0444920166748298	-4.247372952	Down
DALESSIO_TSA_RESPONSE	-0.099398294	-0.004483643	-2.252056765	0.0250993389849891	0.0444864640433545	-4.247020862	Down
BUCKANOVICH_T_LYMPHOCYTE_HOMING_ON_TUMOR_DN	-0.06789834	-0.001302366	-2.25230014	0.025083765302322	0.0444793210520445	-4.246488258	Down
TURASHVILI_BREAST_LOBULAR_CARCINOMA_VS_LOBULAR_NORMAL_UP	-0.057497213	-0.00019464	-2.252658956	0.0250608199088042	0.0444562758427972	-4.245702924	Down
REN_MIF_TARGETS_DN	-0.140113492	-0.003030028	-2.252814365	0.0250508875170948	0.0444513604398534	-4.245362744	Down
WP_PHOTODYNAMIC_THERAPYINDUCED_NFE2L2_NRF2_SURVIVAL_SIGNALING	-0.092242611	-7.31E-06	-2.25697482	0.0247862589376906	0.0440195448429887	-4.236247404	Down
NIKOLSKY_BREAST_CANCER_7Q21_Q22_AMPLICON	-0.050051095	0.0055209319815407	-2.259072151	0.0246537820509845	0.0438344386108452	-4.231646117	Down
WP_OSTEOCLAST_SIGNALING	-0.082268286	-0.005075712	-2.264534193	0.0243116654113	0.0433005759563	-4.21964	Down

				126	895	3744	
REACTOME_NOTCH3_ACTIVATION_AND_TRANSMISSION_OF_SIGNAL_TO_THE_NUCLEUS	-0.083349768	-0.001052452	-2.264584005	0.0243085645701049	0.0433005759563895	-4.219534158	Down
DAVICIONI_PAX_FOXO1_SIGNATURE_IN_ARMS_DN	-0.084557801	0.00617675860051386	-2.265702724	0.0242390137594994	0.0432083740394063	-4.217072373	Down
VANHARANTA_UTERINE_FIBROID_WITH_7Q_DELETION_UP	-0.100166631	0.00155862414795271	-2.267017509	0.0241574951964256	0.0430878082425901	-4.214177637	Down
YAMASHITA_LIVER_CANCER_STEM_CELL_UP	-0.07113638	-0.004542609	-2.268096962	0.0240907462924136	0.042981104408454	-4.211799818	Down
PID_MAPK_TRK_PATHWAY	-0.100253989	0.00414791938475346	-2.268631476	0.0240577537571247	0.0429345823951906	-4.210621988	Down
PID_HIV_NEF_PATHWAY	-0.082120148	0.00388519683451422	-2.269797369	0.023985926339454	0.0428187071581797	-4.208051952	Down
STEIN_ESR1_TARGETS	-0.058938092	0.00029814661007203	-2.27006643	0.0239693767832055	0.042801473444579	-4.207458666	Down
SCHAEFFER_PROSTATE_DEVELOPMENT_48HR_UP	-0.035366126	-0.000897707	-2.274519063	0.0236969444641917	0.042351550328027	-4.197630683	Down
KAPOSI_LIVER_CANCER_MET_UP	-0.078798487	7.0659812	-2.2771	0.023539	0.042107	-4.1	Down

		9635146e-05	0797	7882811513	050680434	91907843	
SUZUKI_CTCFL_TARGETS_UP	-0.117374311	0.00684814278881896	-2.277841011	0.023495455548888	0.0420398652614436	-4.190286301	Down
WP_IL10_ANTIINFLAMMATORY_SIGNALING_PATHWAY	-0.099459115	0.000510111170423391	-2.278912633	0.0234307772429877	0.0419725351651442	-4.187914888	Down
REACTOME_FGFR2_ALTERNATIVE_SPLICING	-0.111581434	0.00774283650358227	-2.283935545	0.0231296833858707	0.0414690770583765	-4.176785272	Down
ROSS_LEUKEMIA_WITH_MLL_FUSIONS	-0.064586876	0.00338696218025988	-2.285872287	0.0230144926657001	0.041298339397514	-4.172487596	Down
BIOCARTA_TNFR1_PATHWAY	-0.089359836	0.0119044755268009	-2.286183568	0.0229960256078728	0.041277134455774	-4.171796531	Down
BIOCARTA_RANBP2_PATHWAY	-0.103514065	0.0106573113873628	-2.286417815	0.0229821371537726	0.0412641380715843	-4.171276425	Down
REACTOME_EPHB_MEDIATED_FORWARD_SIGNALING	-0.09329492	0.00572629258406422	-2.28788719	0.0228951852318841	0.0411199116708057	-4.168012758	Down
OISHI_CHOLANGIOMA_STEM_CELL_LIKE_UP	-0.073601939	0.00332386967050191	-2.288166828	0.0228786699461013	0.0411021430840668	-4.167391419	Down

WP_INTEGRATED_CANCER_PATHWAY	-0.064195555	0.0055068421598162	-2.290805902	0.02272331911 9292	0.0408466961405866	-4.16152395	Down
REACTOME_PLATELET_SENSITIZATION_BY_LDL	-0.088350502	0.00980961690679156	-2.291923075	0.0226578341244339	0.0407462300724687	-4.159038171	Down
PECE_MAMMARY_STEM_CELL_DN	-0.076514036	0.00980325537098687	-2.292109371	0.0226469301314926	0.0407447812539637	-4.158623538	Down
LI_ADIPOGENESIS_BY_ACTIVATED_PPARG	-0.105042466	0.00251480253509229	-2.292753083	0.0226092885827694	0.040688853068498	-4.157190596	Down
REACTOME_INTRINSIC_PATHWAY_FOR_APOPTOSIS	-0.065469335	0.00614516989034563	-2.292781782	0.0226076116737639	0.040688853068498	-4.157126702	Down
RAO_BOUND_BY_SALL4	-0.047341562	0.00563420678337366	-2.296334495	0.0224008595540954	0.0403957394689919	-4.149211093	Down
REACTOME_SIALIC_ACID_METABOLISM	-0.062945337	0.00370579926543705	-2.301939926	0.0220780090585064	0.0398598610314569	-4.136697955	Down
REACTOME_HEME_DEGRADATION	-0.084378087	0.00311517290667998	-2.304524529	0.0219305219446956	0.039616632628267	-4.130918396	Down
STEIN_ESRRA_TARGETS_RESPONSIVE_TO_ESTROGEN_UP	-0.079496182	0.0024376254319453	-2.306341001	0.0218273836814	0.0394417963641	-4.12685	Down

		1		612	426	2761	
VALK_AML_CLUSTER_4	-0.082861657	0.00894579407522676	-2.307720293	0.0217493519197997	0.03932368988237 6	-4.123763565	Down
REACTOME_SYNTHESIS_SECRETION_AND_INACTIVATION_OF_GLUCOSE_DEPENDENT_INSULINOTROPIC_POLYPEPTIDE_GIP_	-0.109791347	0.00251734629769703	-2.308312621	0.0217159166667013	0.0392943263874947	-4.122436384	Down
KRISHNAN_FURIN_TARGETS_DN	-0.102861202	-7.93E-05	-2.311589133	0.0215317782246111	0.0389984673008932	-4.115089058	Down
PEDERSEN_TARGETS_OF_611CTF_ISOFORM_OF_ERBB2	-0.076433773	0.000742735866729858	-2.312530408	0.0214791323024362	0.0389258318835683	-4.112976466	Down
WP_PROSTAGLANDIN_SYNTHESIS_AND_REGULATION	-0.069450749	-0.002208354	-2.313136505	0.0214452927664779	0.0388758563672688	-4.11161571	Down
SASAKI_TARGETS_OF_TP73_AND_TP63	-0.105888574	0.00147004906511835	-2.315907441	0.0212911797553723	0.0386190393751011	-4.105390273	Down
REACTOME_CONSTITUTIVE_SIGNALING_BY_OVEREXPRESSED_ERBB2	-0.101323978	0.00871335183231344	-2.324268051	0.0208320445746742	0.037841527853381	-4.086563137	Down
BENPORATH_NOS_TARGETS	-0.047108096	0.00132788347489717	-2.326395975	0.020716580842677	0.0376648556797002	-4.081760883	Down
BIOCARTA_RANMS_PATHWAY	-0.127997264	-0.0082055	-2.3265	0.020706	0.037656	-4.0	Down

		94	88886	1410226028	905164751	81325316	
CUI_GLUCOSE_DEPRIVATION	-0.068216753	0.00218713420332653	-2.326734824	0.0206982463534566	0.0376535800456932	-4.080995786	Down
ST_TUMOR_NECROSIS_FACTOR_PATHWAY	-0.098178519	0.00162668353917536	-2.327079444	0.0206796141788299	0.0376307139278795	-4.080217548	Down
HEIDENBLAD_AMPLIFIED_IN_BONE_CANCER	-0.127375525	0.00814936860140829	-2.327717303	0.0206451666130213	0.0375790466765463	-4.078776812	Down
WANG_HCP_PROSTATE_CANCER	-0.050522352	0.00182334698945606	-2.329950764	0.020524944625893	0.0373711737438891	-4.073729087	Down
REACTOME_ACTIVATION_OF_PPARGC1A_PGC_1ALPHA_BY_PHOSPHORYLATION	-0.11016226	-0.000368057	-2.330769397	0.0204810335348833	0.0373131127534549	-4.071877775	Down
RUIZ_TNC_TARGETS_DN	-0.079145434	-0.007319255	-2.331026355	0.0204672674134212	0.0372989808676175	-4.071296543	Down
REACTOME_SIGNALING_BY_FGFR4	-0.062620907	0.000227336303761383	-2.332075831	0.0204111276061773	0.0372185279234849	-4.06892202	Down
WU_CELL_MIGRATION	-0.055672002	-0.002898222	-2.333120005	0.0203554054739671	0.0371387424388341	-4.066558473	Down

WANG_TUMOR_INVASIVENESS_DN	-0.062641647	0.0095261694529651 6	-2.334443836	0.0202849513306469	0.0370210799498164	-4.063560445	Down
RAO_BOUND_BY_SALL4_ISOFORM_A	-0.04297217	-0.000609618	-2.334866098	0.0202625236361518	0.0369910247675278	-4.062603821	Down
REACTOME_SYNTHESIS_OF_PIPS_AT_THE_EARLY_ENDOSOME_MEMBRANE	-0.127711921	0.0175981132238133	-2.335631853	0.0202219073767218	0.0369277373013568	-4.060868598	Down
MOLENAAR_TARGETS_OF_CCND1_AND_CDK4_UP	-0.070962615	0.0028394293874831 8	-2.338697791	0.0200600022640226	0.0366428587559707	-4.053915619	Down
KORKOLA_YOLK_SAC_TUMOR	-0.070261493	-0.003114323	-2.340562284	0.0199620998220821	0.0364747581971338	-4.049683008	Down
REACTOME_TELOMERE_MAINTENANCE	-0.102907268	0.000762314202400358	-2.342762987	0.0198470831397822	0.0363287658651219	-4.044682991	Down
GRAESSMANN_RESPONSE_TO_MC_AND_DOXORUBICIN_DN	-0.071767249	0.0042508349150296 3	-2.345081667	0.0197265303352169	0.0361294109739425	-4.039410049	Down
LUCAS_HNF4A_TARGETS_DN	-0.124862429	0.0160723036715001	-2.346285168	0.0196642116780651	0.0360259037443182	-4.036671172	Down
REACTOME_SHC1_EVENTS_IN_EGFR_SIGNALING	-0.089103802	0.0072801081214566	-2.350253336	0.0194599585060	0.0356948725198	-4.02763	Down

		8		226	835	1028	
KYNG_ENVIRONMENTAL_STRESS_RESPONSE_DN	-0.099367464	0.000529954077376453	-2.35065949	0.0194391580959375	0.0356767753700426	-4.026704915	Down
PID_RET_PATHWAY	-0.087029496	0.00657332749451183	-2.350893547	0.0194271801961589	0.0356653379111382	-4.026171148	Down
KEGG_CYTOSOLIC_DNA_SENSING_PATHWAY	-0.070141013	-0.009254412	-2.354497864	0.019243545334816	0.0353595872981654	-4.017945051	Down
SCHUHMACHER_MYC_TARGETS_DN	-0.115860136	0.00436171561128213	-2.358381078	0.0190474095869458	0.0350303026092364	-4.009068886	Down
REACTOME_LOSS_OF_FUNCTION_OF_SMAD2_3_IN_CANCER	-0.115655942	0.00502862487496333	-2.359665162	0.018982940047666	0.0349220832471437	-4.006130666	Down
REACTOME_SIGNALING_BY_TGF_BETA_RECEPTOR_COMPLEX_IN_CANCER	-0.115655942	0.00502862487496333	-2.359665162	0.018982940047666	0.0349220832471437	-4.006130666	Down
BIOCARTA_P35ALZHEIMERS_PATHWAY	-0.127512168	0.00101443852801084	-2.359899239	0.0189712084959035	0.0349212014039363	-4.005594889	Down
REACTOME_APOBEC3G_MEDIATED_RESISTANCE_TO_HIV_1_INFECTION	-0.165891611	0.00177811344673409	-2.360536243	0.0189393152537059	0.034872835888387	-4.004136598	Down
REACTOME_GLUTATHIONE_SYNTHESIS_AND_RECYCL	-0.090972261	-0.0048542	-2.3632	0.018803	0.034653	-3.9	Down

ING		57	61029	4232198757	4584102637	97894488	
NAKAMURA_TUMOR_ZONE_PERIPHERAL_VS_CENTRAL_DN	-0.051336536	0.00592789323820182	-2.370553481	0.0184439397019155	0.0340516162194495	-3.98115448	Down
WP_METHYLATION_PATHWAYS	-0.108555423	-0.004948309	-2.372918916	0.0183286409244424	0.0338891493053363	-3.975713929	Down
KEGG_INTESTINAL_IMMUNE_NETWORK_FOR_IGA_PRODUCTION	-0.079147702	-0.000301122	-2.374683072	0.0182430638840243	0.0337409706579675	-3.971652944	Down
NAKAMURA_ADIPOGENESIS_EARLY_UP	-0.063264885	0.00777575444768039	-2.374945064	0.0182303849975901	0.0337284022744226	-3.971049606	Down
BIOCARTA_ALK_PATHWAY	-0.066019388	0.00167942361037729	-2.37578905	0.0181895937912507	0.0336721767558285	-3.96910557	Down
TURASHVILI_BREAST_LOBULAR_CARCINOMA_VS_DUCTAL_NORMAL_DN	-0.053281784	-0.002308627	-2.378842239	0.0180426985042848	0.0334201819206492	-3.962067319	Down
REACTOME_SYNTHESIS_OF_DIPHTHAMIDE_EEF2	-0.15262829	-0.00425509	-2.380796133	0.01794924168159 2	0.0332768647304784	-3.957558627	Down
REACTOME_RESOLUTION_OF_SISTER_CHROMATID_COHESION	-0.076092583	-4.36E-05	-2.38148304	0.0179164876086228	0.0332260647107026	-3.955972715	Down

GAVIN_FOXP3_TARGETS_CLUSTER_P6	-0.089688201	-0.005062229	-2.38295517	0.0178464687270622	0.0331160034047458	-3.952572427	Down
BOYLAN_MULTIPLE_MYELOMA_PCA1_UP	-0.065592695	0.0018617004386024	-2.383450916	0.0178229438487562	0.0330822405709419	-3.95142691	Down
ROYLANCE_BREAST_CANCER_16Q_COPY_NUMBER_DN	-0.085981614	-0.000683675	-2.384195906	0.0177876429292092	0.0330364750633158	-3.949705041	Down
BIOCARTA_SARS_PATHWAY	-0.09920498	-0.000690406	-2.388630377	0.0175787896564263	0.0326583500141988	-3.939445111	Down
REACTOME_SIGNALING_BY_HIPPO	-0.093023079	0.00799011034138539	-2.392365372	0.0174045599867785	0.0323602450459735	-3.930789356	Down
BALDWIN_PRKCI_TARGETS_UP	-0.061919562	0.00708020628118216	-2.39260368	0.0173934952833479	0.0323602450459735	-3.930236644	Down
REACTOME_RELAXIN_RECEPTORS	-0.122203192	0.00534863827310399	-2.397268274	0.0171781617480652	0.0320290928117275	-3.919407319	Down
REACTOME_PTEN_REGULATION	-0.064774181	0.00225695869088602	-2.39960344	0.0170712483175565	0.0318584601043516	-3.913978399	Down
MOOTHA_GLYCOGEN_METABOLISM	-0.110092621	-0.003025595	-2.400638346	0.0170240541490	0.0317990683428	-3.91157	Down

				78	611	0774	
LI_CISPLATIN_RESISTANCE_DN	-0.070039578	-0.001877528	-2.402155322	0.0169550843851006	0.0316988580657589	-3.90803985	Down
KEGG_NOD_LIKE_RECEPTOR_SIGNALING_PATHWAY	-0.079102922	3.60010010880933e-05	-2.405144622	0.0168198962837949	0.0314745541855637	-3.90107568	Down
REACTOME_RRNA_PROCESSING	-0.141600695	0.000798961776266513	-2.408679219	0.0166612758347594	0.0312059562783197	-3.89283042	Down
PID_EPHB_FWD_PATHWAY	-0.085883543	0.00426836702546782	-2.410280683	0.0165898433911964	0.0310815448020392	-3.889090814	Down
REACTOME_BASE_EXCISION_REPAIR	-0.110586191	0.00180935882921314	-2.412743574	0.0164805151775582	0.0309233850384231	-3.883335029	Down
BIOCARTA_ACTINY_PATHWAY	-0.095022547	0.00127851672043862	-2.413584189	0.0164433460374867	0.0308629721362807	-3.88136922	Down
KEGG_SPHINGOLIPID_METABOLISM	-0.083649076	0.00692403584823973	-2.414991393	0.0163812899160911	0.0307837319131631	-3.878076951	Down
CERVERA_SDHB_TARGETS_2	-0.05839907	-0.000218099	-2.419010081	0.0162052077766154	0.0305087310955419	-3.86866478	Down
BIOCARTA_SAM68_PATHWAY	-0.129161983	0.0067656	-2.4213	0.016103	0.030334	-3.8	Down

		9087892857	52254	3567134827	9120244573	63172258	
RASHI_RESPONSE_TO_IONIZING_RADIATION_4	-0.064269082	0.00301702238774533	-2.427704909	0.015829949637369	0.0298379888852565	-3.848249304	Down
RHODES_CANCER_META_SIGNATURE	-0.124779163	0.00204269502702109	-2.429597899	0.0157492763885052	0.0296949448795418	-3.843795259	Down
GREGORY_SYNTHETIC_LETHAL_WITH_IMATINIB	-0.041482557	0.00091766331939433	-2.432912667	0.015608886883764	0.0294844275321332	-3.835987881	Down
GOZGIT_ESR1_TARGETS_DN	-0.034857546	0.000279741226075103	-2.4331732	0.0155978996491663	0.0294811702565088	-3.835373806	Down
REACTOME_PI_3K_CASCADE_FGFR4	-0.090328925	0.000967214450921857	-2.43425241	0.015552459959337	0.0294131989541758	-3.832829448	Down
GRAESSMANN_RESPONSE_TO_MC_AND_SERUM_DEPRIVATION_UP	-0.072012978	-0.004587341	-2.435094961	0.0155170661584443	0.0293552056219029	-3.83084229	Down
BIOCARTA_CHREBP_PATHWAY	-0.086115538	0.0024367713492816	-2.436444505	0.0154605230890984	0.0292571545164737	-3.827658014	Down
REACTOME_TNFR1_MEDIATED_CERAMIDE_PRODUCTION	-0.134399169	0.012580722977587	-2.437753127	0.0154058687360443	0.0291644036061876	-3.824568679	Down

WP_DNA_IRDAMAGE_AND_CELLULAR_RESPONSE_VIA_ATR	-0.086928301	0.0029285048607219	-2.438118845	0.0153906251851571	0.0291515442551938	-3.823705025	Down
REACTOME_FGFR2_MUTANT_RECEPTOR_ACTIVATION	-0.070743308	-0.004370564	-2.439257151	0.0153432647223171	0.0290795859943274	-3.821016089	Down
WP_NANOPARTICLE_TRIGGERED_AUTOPHAGIC_CELL_DEATH	-0.084958876	0.00588860801684878	-2.443398827	0.0151720323573774	0.0287902185393585	-3.811222371	Down
REACTOME_NONHOMOLOGOUS_END_JOINING_NHEJ_	-0.091997651	0.00566236985085196	-2.448114814	0.0149791177272168	0.028467661890029	-3.800051226	Down
ELVIDGE_HIF1A_AND_HIF2A_TARGETS_DN	-0.056951528	0.00107467809778177	-2.453660307	0.0147550527796029	0.0280934394487714	-3.786888787	Down
REACTOME_SULFIDE_OXIDATION_TO_SULFATE	-0.152837726	-0.011786501	-2.453847059	0.0147475590357402	0.0280877873380913	-3.786445027	Down
FIRESTEIN_CTNNB1_PATHWAY_AND_PROLIFERATION	-0.120665979	0.00381194755841774	-2.458310275	0.0145694659095214	0.0277774985941867	-3.775829943	Down
REACTOME_METALLOTHIONEINS_BIND_METALS	-0.193264719	-0.005787033	-2.459934031	0.0145051486023209	0.0276770542190611	-3.771963506	Down
ST_P38_MAPK_PATHWAY	-0.101651424	0.0108348914355934	-2.460130867	0.0144973690115	0.0276770542190	-3.77149	Down

				104	611	464	
KASLER_HDAC7_TARGETS_2_UP	-0.122461586	0.0108461377663297	-2.463803866	0.014352877413186	0.027420224716419 1	-3.762738926	Down
WP_MITOCHONDRIAL_GENE_EXPRESSION	-0.112567042	0.00588741989675866	-2.46472473	0.0143168525013709	0.0273682486837109	-3.760541807	Down
MARIADASON_RESPONSE_TO_BUTYRATE_SULINDAC_4	-0.107371993	0.00467505162618704	-2.465139107	0.0143006679288774	0.0273457319268461	-3.759552875	Down
ZHENG_IL22_SIGNALING_DN	-0.06799257	0.00203930038944113	-2.467111095	0.0142238689698064	0.0272240378339773	-3.754844446	Down
WP_GLYCEROPHOSPHOLIPID_BIOSYNTHETIC_PATHWAY	-0.071290615	0.0035369914347496	-2.467573853	0.014205899998853	0.0272064243421416	-3.753739019	Down
REACTOME_NEGATIVE_REGULATORS_OF_DDX58_IFIH1_SIGNALING	-0.080979139	-0.002167071	-2.468622877	0.0141652407091947	0.0271369287290035	-3.751232394	Down
NAKAMURA_TUMOR_ZONE_PERIPHERAL_VS_CENTRAL_UP	-0.052010341	-0.002096478	-2.470060236	0.0141096975025009	0.0270399749348596	-3.747796193	Down
YAN_ESCAPE_FROM_ANOIKIS	-0.112094978	-0.01217454	-2.474885608	0.013924643550409	0.0267503133758554	-3.736246502	Down
WP_INHIBITION_OF_EXOSOME_BIOGENESIS_AND_SEC	-0.105130857	0.0098842	-2.4755	0.013897	0.026715	-3.7	Down

RETION_BY_MANUMYCIN_A_IN_CRPC_CELLS		1044592684	91665	7475788561	1809296871	34554724	
REACTOME_FLT3_SIGNALING	-0.032257762	0.000650777952068224	-2.47649622	0.0138633575638485	0.0266573297394075	-3.732386651	Down
REACTOME_INHIBITION_OF_DNA_RECOMBINATION_AT_TELOMERE	-0.114925115	0.0076353979307865	-2.480164531	0.0137246661912417	0.0264151947438874	-3.723586535	Down
REACTOME_PHOSPHORYLATION_SITE_MUTANTS_OF_CTNNB1_ARE_NOT_TARGETED_TO_THE_PROTEASOME_BY_THE_DESTRUCTION_COMPLEX	-0.097803473	0.0148213159611641	-2.483612889	0.0135954156582774	0.0261907960865697	-3.715302721	Down
PID_PLK1_PATHWAY	-0.090872985	-0.001666181	-2.485801672	0.0135139381945046	0.0260419169119186	-3.710039011	Down
WP_FACTORS_AND_PATHWAYS_AFFECTING_INSULINLIKE_GROWTH_FACTOR_IGF1AKT_SIGNALING	-0.070191556	0.00275224362906614	-2.488190168	0.0134255216166649	0.0259037030384331	-3.704289963	Down
REACTOME_MITOCHONDRIAL_FATTY_ACID_BETA_OXIDATION_OF_UNSATURATED_FATTY_ACIDS	-0.14196535	0.0213473811533685	-2.490058146	0.0133567319783441	0.0257870094524361	-3.699790111	Down
FINAK_BREAST_CANCER_SDPP_SIGNATURE	-0.088587602	0.00294065390397004	-2.497359183	0.0130908610738794	0.0253525662045458	-3.682171376	Down
CORRE_MULTIPLE_MYELOMA_DN	-0.055333595	-0.001667401	-2.499043098	0.0130302123594744	0.0252508673478793	-3.678100788	Down

REACTOME_TNFR1_INDUCED_NFKAPPAB_SIGNALING_PATHWAY	-0.063187978	-0.003026657	-2.500630297	0.0129732758848822	0.0251719673077411	-3.674261593	Down
BIOCARTA_RAN_PATHWAY	-0.173575726	-0.011119775	-2.503563403	0.0128686406177248	0.024992381825475	-3.671607725	Down
FLECHNER_BIOPSY_KIDNEY_TRANSPLANT_REJECTED_VS_OK_UP	-0.102144522	0.000754085637414583	-2.504119078	0.0128489023503788	0.0249696734154044	-3.665814575	Down
JAZAG_TGFB1_SIGNALING_DN	-0.071346209	-0.002301462	-2.504456965	0.0128369133515315	0.0249541876520377	-3.649958887	Down
HUANG_DASATINIB_RESISTANCE_UP	-0.073778881	-0.003585248	-2.504590154	0.0128321902403291	0.024952821059437	-3.646731446	Down
CAFFAREL_RESPONSE_TO_THC_UP	-0.116344114	0.0136378104685604	-2.504857847	0.0128227020403717	0.0249428475983777	-3.640244427	Down
GROSS_HYPOXIA_VIA_ELK3_ONLY_UP	-0.109630341	0.00963668430762737	-2.510312539	0.0126307190235836	0.0245995836144913	-3.650791289	Down
DAVICIONI_RHABDOMYOSARCOMA_PAX_FOXO1_FUSION_DN	-0.100142418	3.29242836905343e-05	-2.512513075	0.0125539961094151	0.0244808840248633	-3.645444982	Down
HEDENFALK_BREAST_CANCER_BRACX_UP	-0.106269673	0.0036165645048719	-2.514194157	0.0124956640754	0.0243747916141	-3.64135	Down

		7		131	072	7701	
REACTOME_REGULATION_OF_GENE_EXPRESSION_IN_ENDOCRINE_COMMITTED_NEUROG3_PROGENITOR_CELLS	-0.159659669	0.0039473639222787 8	-2.514656383	0.0124796676278448	0.0243512407941002	-3.640233416	Down
PID_FOXO_PATHWAY	-0.098879399	0.0127379866598189	-2.514724643	0.0124773068505647	0.0243512407941002	-3.640067368	Down
WANG_LMO4_TARGETS_UP	-0.059283883	0.0039246974375397 7	-2.515565961	0.012448242646178	0.0243052035560952	-3.638020445	Down
DESERT_PERIVENOUS_HEPATOCELLULAR_CARCINOMA_SUBCLASS_UP	-0.060597268	0.0031371767222397	-2.5184461	0.0123492011413156	0.0241422020422506	-3.631008133	Down
REACTOME_CREB_PHOSPHORYLATION	-0.146761011	0.0105647397440475	-2.520982527	0.0122625609092741	0.0239954967099194	-3.624826317	Down
FURUKAWA_DUSP6_TARGETS_PCI35_UP	-0.07245206	-0.002613021	-2.521592468	0.0122418072444911	0.0239624401029821	-3.623338874	Down
WORSCHECH_TUMOR_EVASION_AND_TOLEROGENICITY_DN	-0.138878619	-0.004601001	-2.523155722	0.0121887591947386	0.0238736599311274	-3.619525047	Down
TAKAO_RESPONSE_TO_UVB_RADIATION_DN	-0.073123336	0.0069479778273997 6	-2.526603022	0.0120725004519236	0.0236683544867625	-3.611106799	Down
FONTAINE_FOLLICULAR_THYROID_ADENOMA_DN	-0.05269713	-0.0004396	-2.5268	0.012063	0.023659	-3.6	Down

		49	5761	9538971367	0716712567	10484665	
SHAFFER_IRF4_TARGETS_IN_ACTIVATED_DENDRITIC_CELL	-0.120883402	0.00411195222924936	-2.527804675	0.0120322080668721	0.0236042716406686	-3.608169803	Down
WP_TNF_ALPHA_SIGNALING_PATHWAY	-0.074373699	0.00258036617730544	-2.528418929	0.0120116579941317	0.0235788618499607	-3.606667974	Down
REACTOME_NF_KB_ACTIVATION_THROUGH_FADD_RIP_1_PATHWAY_MEDIATED_BY_CASPASE_8_AND_10	-0.146158282	0.0010189659673808	-2.528637139	0.0120043652147949	0.0235720009137083	-3.606134372	Down
LE_SKI_TARGETS_UP	-0.095736761	-0.003069948	-2.52903866	0.0119909563425293	0.0235605780367456	-3.605152397	Down
REACTOME_DISEASES_OF_MISMATCH_REPAIR_MMR_	-0.199574016	0.0107598325762739	-2.531862345	0.0118970353544394	0.0233908452470716	-3.598242485	Down
REACTOME_RUNX1_AND_FOXP3_CONTROL_THE_DEVELOPMENT_OF_REGULATORY_T_LYMPHOCYTES_TREGS_	-0.122852714	0.00109982582501789	-2.535717175	0.0117698755048338	0.0231701925970515	-3.588797353	Down
BIOCARTA_NKCELLS_PATHWAY	-0.104629296	0.00242947474599616	-2.535729308	0.0117694771936629	0.0231701925970515	-3.588767604	Down
SIMBULAN_PARP1_TARGETS_DN	-0.117900295	-0.001667912	-2.539000743	0.0116625173303171	0.0229880105015174	-3.580741119	Down

TING_SILENCED_BY_DICER	-0.092985855	0.0050671062442594 1	-2.54283117	0.0115383836858879	0.0227650182893535	-3.571330595	Down
MANNE_COVID19_NONICU_VS_HEALTHY_DONOR_PLATELETS_DN	-0.057815024	-0.000130529	-2.549648001	0.0113203818687038	0.022363338720256	-3.554549662	Down
WP_UNFOLDED_PROTEIN_RESPONSE	-0.116127667	0.013716821341551	-2.550322073	0.0112990261549277	0.0223353679438332	-3.55288798	Down
WP_CELL_CYCLE	-0.082498081	0.000528923484780143	-2.551897436	0.0112492561483328	0.0222511577159661	-3.549002847	Down
KEGG_RNA_POLYMERASE	-0.128542366	-0.002095331	-2.551914445	0.0112487198801144	0.0222511577159661	-3.548960889	Down
TONKS_TARGETS_OF_RUNX1_RUNX1T1_FUSION_SUSTAINED_IN_MONOCYTE_DN	-0.139047204	0.0124383079901227	-2.555219351	0.0111449488744472	0.0220588959386779	-3.540802904	Down
BLANCO_MELO_COVID19_SARS_COV_2_INFECTION_A594_ACE2_EXPRESSING_CELLS_RUXOLITINIB_DN	-0.075981859	-0.005637115	-2.55597516	0.0111213376217818	0.0220191842482933	-3.538935816	Down
GRYDER_PAX3FOXO1_ENHANCERS_IN_TADS	-0.055558767	0.00548306399784685	-2.557878735	0.0110620683355224	0.0219088251941887	-3.53423105	Down
CHIARETTI_T_ALL_RELAPSE_PROGNOSIS	-0.142860579	0.0044135828408286	-2.558730461	0.0110356406551	0.0218634604361	-3.53212	Down

		8		248	186	4892	
REACTOME_PI_3K_CASCADE_FGFR1	-0.089585539	-0.0018264 84	-2.5588 27143	0.011032 6443180 675	0.021863 4604361 186	-3.5 3188 5771	Down
PUJANA_BRCA2_PCC_NETWORK	-0.114637431	0.0024311 558844851 2	-2.5637 7089	0.010880 3955829 605	0.021576 5544356 026	-3.5 1964 7173	Down
BORLAK_LIVER_CANCER_EGF_UP	-0.075048029	0.0008441 055076763 25	-2.5654 99598	0.010827 6025989 246	0.021485 5912185 182	-3.5 1536 232	Down
BIOCARTA_AKAP95_PATHWAY	-0.114971462	0.0035384 120816648	-2.5667 71624	0.010788 9025242 879	0.021415 6437378 495	-3.5 1220 766	Down
STEARMAN_LUNG_CANCER_EARLY_VS_LATE_UP	-0.084758915	0.0053183 709541545 5	-2.5696 4364	0.010701 9787830 122	0.021263 5026588 21	-3.5 0507 9502	Down
REACTOME_G1_S_SPECIFIC_TRANSCRIPTION	-0.112916966	-0.0077213 21	-2.5722 61899	0.010623 2812878 193	0.021113 8991205 554	-3.4 9857 4534	Down
BENPORATH_ES_1	-0.072087501	-0.0003856 93	-2.5749 54643	0.010542 8850988 246	0.020967 5385480 308	-3.4 9187 7926	Down
GAURNIER_PSMD4_TARGETS	-0.077672056	-0.0006723 49	-2.5778 86543	0.010455 9676936 966	0.020808 0126562 279	-3.4 8457 897	Down
BOYLAN_MULTIPLE_MYELOMA_PCA3_UP	-0.048768114	0.0027363	-2.5782	0.010446	0.020795	-3.4	Down

		0568554918	17129	2076339368	2568261211	83755479	
REACTOME_PHASE_II_CONJUGATION_OF_COMPOUNDS	-0.059993596	-0.001271846	-2.579043391	0.0104218491601899	0.0207534224373753	-3.481696822	Down
REACTOME_SIRT1_NEGATIVELY_REGULATES_RRNA_EXPRESSION	-0.159338206	0.00282176787837715	-2.581509956	0.0103494359564285	0.0206288252276074	-3.47554756	Down
GROSS_HYPOXIA_VIA_ELK3_AND_HIF1A_UP	-0.064094199	0.00879504264366342	-2.582453272	0.0103218613856126	0.020580723938483	-3.473194349	Down
FLECHNER_BIOPSY_KIDNEY_TRANSPLANT_REJECTED_VS_OK_DN	-0.068466454	0.00406938472055204	-2.582454864	0.0103218148919977	0.020580723938483	-3.473190376	Down
REACTOME_CONSTITUTIVE_SIGNALING_BY_EGFRVIII	-0.09523292	0.00462011751748602	-2.584759144	0.0102547337716204	0.020466602096607	-3.467438637	Down
TERAMOTO_OPN_TARGETS_CLUSTER_3	-0.149297407	0.00189877996673764	-2.584817532	0.0102530390698861	0.020466602096607	-3.467292829	Down
REACTOME_SIGNALING_BY_NODAL	-0.092490279	-0.002782182	-2.585814925	0.0102241289085202	0.0204317991420427	-3.464801658	Down
LY_AGING_MIDDLE_DN	-0.153229119	-0.017917784	-2.591191904	0.0100695264055626	0.0201617904513959	-3.451355896	Down

REACTOME_IRAK1_RECRUITS_IKK_COMPLEX	-0.095925559	0.010690984396486	-2.59187868	0.0100499312521261	0.0201290491390 6	-3.449636617	Down
REACTOME_ZINC_INFLUX_INTO_CELLS_BY_THE_SLC39_GENE_FAMILY	-0.119985802	-0.001442281	-2.592215709	0.0100403276205859	0.020116305210128	-3.448792739	Down
REACTOME_RNA_POLYMERASE_III_CHAIN_ELONGATION	-0.140543883	-0.00796629	-2.592400655	0.0100350610582616	0.0201122453951017	-3.448329611	Down
EPPERT_HSC_R	-0.087374498	0.00702983148997717	-2.594395533	0.0099784117085953	0.0200181000243216	-3.443332205	Down
PID_TGFBR_PATHWAY	-0.071498051	0.00433406837353792	-2.595238384	0.0099545631202 4027	0.0199767129929943	-3.441219664	Down
REACTOME_FRS_MEDIATED_FGFR3_SIGNALING	-0.086072947	-0.000521682	-2.595377421	0.00995063396191244	0.0199752862230241	-3.440871116	Down
WP_NAD_BIOSYNTHETIC_PATHWAYS	-0.088039522	0.00365954798219293	-2.595568909	0.0099452248262456	0.0199708866051461	-3.440391052	Down
REACTOME_TLR3_MEDIATED_TICAM1_DEPENDENT_PROGRAMMED_CELL_DEATH	-0.143571982	0.00138923808859094	-2.599329865	0.00983951790357231	0.0197778133508657	-3.430955398	Down
CHANGOLKAR_H2AFY_TARGETS_UP	-0.075099413	0.0045956069530112	-2.600982135	0.0097933976334	0.0196978675018	-3.42680	Down

		8		329	529	5999	
KEGG_TGF_BETA_SIGNALING_PATHWAY	-0.054080266	0.000122800285455295	-2.602974133	0.00973805202330882	0.019605607819876	-3.421800094	Down
REACTOME_TRAIL_SIGNALING	-0.163251379	8.0207787988476e-05	-2.60710568	0.00962415385277274	0.0193951697935586	-3.411405858	Down
REACTOME_SIGNALING_BY_ERBB2_ECD_MUTANTS	-0.091506756	0.00145693433119967	-2.611117375	0.00951470388122616	0.0191808272136313	-3.401298139	Down
KIM_ALL_DISORDERS_CALB1_CORR_UP	-0.05942663	0.00546355729243223	-2.61366137	0.00944587633985682	0.0190490914973352	-3.394880724	Down
REACTOME_MRNA_DECAY_BY_3_TO_5_EXORIBONUCLEASE	-0.161968062	-0.0040961	-2.615312824	0.00940143570302734	0.018983315357414	-3.390711623	Down
MYLLYKANGAS_AMPLIFICATION_HOT_SPOT_6	-0.129660916	0.0139525740581893	-2.617041068	0.00935512957442314	0.0189021123920718	-3.386345987	Down
TONKS_TARGETS_OF_RUNX1_RUNX1T1_FUSION_SUSTAINED_IN_GRANULOCYTE_UP	-0.113404555	0.00486268452308853	-2.618675135	0.00931153503013116	0.0188201556274907	-3.382215721	Down
YAGI_AML_WITH_T_9_11_TRANSLOCATION	-0.066445069	0.00242149906490384	-2.619799022	0.00928165724136858	0.0187719929935402	-3.379373564	Down
SAMOLS_TARGETS_OF_KHSV_MIRNAS_DN	-0.057852886	0.0013405	-2.6212	0.009243	0.018700	-3.3	Down

		5886580395	54071	10348523839	1119077167	7569222	
REACTOME_DOWNSTREAM_SIGNALING_OF_ACTIVATED_FGFR1	-0.071318865	-0.000364868	-2.622765061	0.00920321961714976	0.0186254920650957	-3.371867285	Down
WP_APOPTOSIS	-0.063781577	0.0012784483074787	-2.624379708	0.00916077036635068	0.0185577355300061	-3.367777633	Down
BIOCARTA_CHEMICAL_PATHWAY	-0.09220749	0.0131924292007249	-2.625822777	0.00912298072547583	0.0184992947935408	-3.364120538	Down
FRIDMAN_IMMORTALIZATION_DN	-0.090067169	-0.003559999	-2.629171719	0.00903582055148342	0.0183285418833521	-3.35562612	Down
WP_FAS_LIGAND_FASL_PATHWAY_AND_STRESS_INDUCTION_OF_HEAT_SHOCK_PROTEINS_HSP_REGULATION	-0.08946504	0.00853331800087986	-2.629476439	0.00902792706259787	0.0183185169262978	-3.354852702	Down
DEMAGALHAES_AGING_UP	-0.089204631	0.00461341877904451	-2.633335443	0.00892849757915471	0.0181404859161418	-3.345050695	Down
DUTERTRE_ESTRADIOL_RESPONSE_6HR_DN	-0.065404072	0.00260675278403443	-2.640359826	0.0087500312772654	0.0178245632221813	-3.327173443	Down
MARIADASON_RESPONSE_TO_BUTYRATE_CURCUMIN_SULINDAC_TSA_2	-0.200630573	0.00575760042315013	-2.644467327	0.00864716442510389	0.0176381694336575	-3.316698765	Down

LEONARD_HYPOXIA	-0.091805012	0.00686118261717016	-2.645081383	0.00863187996758352	0.017612780722811	-3.315131513	Down
REACTOME_DOWNSTREAM_SIGNALING_OF_ACTIVATED_FGFR3	-0.079497177	0.000817519885854092	-2.649750949	0.00851644181286936	0.0173886691883158	-3.303202074	Down
JECHLINGER_EPITHELIAL_TO_MESENCHYMAL_TRANSITION_UP	-0.069413381	-0.003590403	-2.652878813	0.00843989480213544	0.0172493997487174	-3.295200076	Down
LIAN_NEUTROPHIL_GRANULE_CONSTITUENTS	-0.139408929	0.00259590721813443	-2.65457495	0.00839864509223393	0.0171707477231542	-3.290857108	Down
RUTELLA_RESPONSE_TO_HGF_DN	-0.088186118	0.00981165672161743	-2.657064461	0.00833842898079795	0.0170588756373806	-3.284477925	Down
WP_PYRIMIDINE_METABOLISM_AND_RELATED_DISEASES	-0.097830493	-0.002627923	-2.65821412	0.00831075238722759	0.0170078602266804	-3.281530093	Down
MORI_MATURE_B_LYMPHOCYTE_UP	-0.08021917	0.0067590691814361	-2.663440693	0.00818596820263312	0.016774613612989	-3.268113418	Down
VANTVEER_BREAST_CANCER_BRCA1_UP	-0.131202626	0.00481845739077823	-2.667508685	0.00809001457818395	0.0165834611911452	-3.257653517	Down
HEIDENBLAD_AMPLIFIED_IN_PANCREATIC_CANCER	-0.087674503	0.0027978694014467	-2.669016602	0.0080547045939	0.0165165349898	-3.25377	Down

		8		0245	753	2406	
CHO_NR4A1_TARGETS	-0.067877839	-0.002413629	-2.670593065	0.00801793789275588	0.0164628979491683	-3.249712643	Down
MCBRYAN_PUBERTAL_BREAST_5_6WK_UP	-0.053572852	0.00203129573236608	-2.672449187	0.00797484262526921	0.0163906782036576	-3.244929777	Down
DAVIES_MULTIPLE_MYELOMA_VS_MGUS_DN	-0.147633969	0.00867507234730421	-2.673682683	0.0079463188848746	0.0163426242028583	-3.241749553	Down
CHEOK_RESPONSE_TO_MERCAPTOPURINE_AND_HD_MTX_DN	-0.104583727	0.00184825835266902	-2.67377573	0.00794417097017327	0.0163426242028583	-3.241509601	Down
WP_FATTY_ACID_BETA_OXIDATION	-0.080434164	0.0062759984364867	-2.677637702	0.00785548023181912	0.0161828628605713	-3.231543249	Down
MORI_PRE_BI_LYMPHOCYTE_UP	-0.113671416	-0.01041318	-2.677940069	0.00784857413259337	0.0161740038648762	-3.230762375	Down
REACTOME_NADE_MODULATES_DEATH_SIGNALLING	-0.118944873	0.0112943732903579	-2.677993525	0.00784735374869583	0.0161740038648762	-3.230624313	Down
REACTOME_DOWNREGULATION_OF_TGF_BETA_RECEPTOR_SIGNALING	-0.093030796	0.00492283987015997	-2.67906039	0.00782303344433991	0.016137443864745	-3.227868368	Down
RAY_TARGETS_OF_P210_BCR_ABL_FUSION_DN	-0.102697227	0.0056411	-2.6809	0.007779	0.016052	-3.2	Down

		6448024463	96132	07945149826	1097591256	22865262	
GINESTIER_BREAST_CANCER_20Q13_AMPLIFICATION_UP	-0.080271308	0.00166704926936387	-2.685851706	0.0076698035001 8591	0.0158371491435975	-3.210300495	Down
WP_OMEGA9_FA_SYNTHESIS	-0.122548656	0.00180027375937565	-2.68915083	0.00759634710911618	0.0157063712545917	-3.201751046	Down
WP_CHOLESTEROL_BIOSYNTHESIS_PATHWAY	-0.139065375	-0.000216527	-2.692110512	0.00753098876731849	0.0155868113633697	-3.194072765	Down
WP_COVID19_ADVERSE_OUTCOME_PATHWAY	-0.108785669	0.00466942070083577	-2.693558027	0.00749920842828842	0.0155417651800956	-3.190314572	Down
KEGG_RNA_DEGRADATION	-0.130626016	0.00508727290442039	-2.694302998	0.00748289969009996	0.0155183288929002	-3.188379653	Down
BIOCARTA_CYTOKINE_PATHWAY	-0.09360595	0.00643616868642515	-2.695609589	0.00745437327403714	0.0154643365347422	-3.184984805	Down
KIM_TIAL1_TARGETS	-0.084910882	-0.004045246	-2.696289952	0.0074395579815858	0.0154387617491485	-3.183216436	Down
LINDSTEDT_DENDRITIC_CELL_MATURATION_C	-0.066101116	0.00265825907595406	-2.69837312	0.00739436074507949	0.0153552349095712	-3.177799326	Down

PID_ERBB_NETWORK_PATHWAY	-0.107857933	-0.003924853	-2.698447683	0.00739274758516447	0.0153552349095712	-3.177605356	Down
AMIT_EGF_RESPONSE_240_MCF10A	-0.096838039	0.00300026676349778	-2.705446718	0.0072427313293984	0.0150959144934775	-3.159375404	Down
GRABARCZYK_BCL11B_TARGETS_DN	-0.077869671	0.00355812543711788	-2.7055404	0.0072407421169352	0.0150959144934775	-3.159131091	Down
REACTOME_METAL_ION_SLC_TRANSPORTERS	-0.078297513	0.00352222413822351	-2.713869766	0.00706583803485562	0.0147619174292659	-3.137377065	Down
BIOCARTA_NDKDYNAMIN_PATHWAY	-0.098675061	0.00153344359127685	-2.720716037	0.00692494251882458	0.014496835822713	-3.119449031	Down
REACTOME_SYNTHESIS_OF_PROSTAGLANDINS_PG_AND_THROMBOXANES_TX_	-0.096723538	0.0034420224552602	-2.722959776	0.00687932170070871	0.0144159182296756	-3.113564137	Down
KYNG_DNA_DAMAGE_BY_UV	-0.081590694	0.0040296719590617	-2.723059952	0.00687729122867814	0.0144159182296756	-3.113301289	Down
WP_NUCLEOTIDEBINDING_OLIGOMERIZATION_DOMAIN_NOD_PATHWAY	-0.1137844	-0.000396001	-2.724542624	0.00684730194984155	0.0143633670843753	-3.10940987	Down
EHLERS_ANEUPLOIDY_DN	-0.123353814	-0.003201238	-2.726375808	0.0068103863984	0.0142955929574	-3.10459	Down

				4483	39	5724	
FU_INTERACT_WITH_ALKBH8	-0.191146524	0.0007296961622811 03	-2.727148404	0.0067948822572 7441	0.0142678735354 879	-3.10256 5882	Down
GRUETZMANN_PANCREATIC_CANCER_UP	-0.056639442	0.0033869237200125 3	-2.729757448	0.0067427604360 7955	0.0141680142270 636	-3.09570 7114	Down
ZHAN_MULTIPLE_MYELOMA_CD1_AND_CD2_UP	-0.093637233	0.0073210956297393 9	-2.733349854	0.0066715848220 3189	0.0140374667763 905	-3.08625 3075	Down
MONTERO_THYROID_CANCER_POOR_SURVIVAL_UP	-0.1870943	-0.022657659	-2.745388127	0.0064379964781 8174	0.0135597706617 15	-3.05448 6464	Down
RORIE_TARGETS_OF_EWSR1_FLI1_FUSION_UP	-0.083156119	0.0034820530545265 6	-2.753029304	0.0062935927073 621	0.0132871870525 839	-3.03425 4371	Down
KEGG_CELL_CYCLE	-0.089644797	0.0005706828084019 25	-2.755204927	0.0062530170940 0325	0.0132150075255 288	-3.02848 4087	Down
GAUSSMANN_MLL_AF4_FUSION_TARGETS_E_UP	-0.051231635	0.0007937990092056 44	-2.756320678	0.0062323001944 7785	0.0131757109356 689	-3.02552 317	Down
CHOI_ATL_CHRONIC_VS_ACUTE_DN	-0.134771312	0.0197896586072965	-2.760143191	0.0061617951681 6357	0.0130355359948 164	-3.01537 0599	Down
REACTOME_HOMOLOGY_DIRECTED_REPAIR	-0.097826644	0.0009858	-2.7611	0.006143	0.013000	-3.0	Down

		67853579334	61605	1330912122	486565685	12663455	
LUI_TARGETS_OF_PAX8_PPARG_FUSION	-0.100358636	0.00433161388446454	-2.766009328	0.00605499987517386	0.0128358552681708	-2.999764298	Down
SARTIPY_BLUNTED_BY_INSULIN_RESISTANCE_UP	-0.09924861	-0.000714179	-2.768531302	0.00600960384072203	0.012743973706649	-2.993045194	Down
GALE_APL_WITH_FLT3_MUTATED_UP	-0.130496369	0.00675092244444025	-2.769901322	0.00598507264592578	0.0127006310814569	-2.989392727	Down
SUH_COEXPRESSED_WITH_ID1_AND_ID2_UP	-0.118765749	0.00391428366133238	-2.771063219	0.00596433917216707	0.0126609621209443	-2.986293776	Down
REACTOME_2_LTR_CIRCLE_FORMATION	-0.167621588	0.00153909045039329	-2.772731778	0.0059346783814909	0.0126152564088747	-2.981841334	Down
REACTOME_TICAM1_TRAF6_DEPENDENT_INDUCTION_OF_TAK1_COMPLEX	-0.120185461	-0.002543366	-2.77307579	0.00592857975262433	0.0126066099775742	-2.980923043	Down
REACTOME_ACTIVATED_NTRK2_SIGNALS_THROUGH_RAS	-0.113253922	-0.00044939	-2.774094848	0.0059105472939716	0.01257460634681	-2.978202187	Down
SCHLOSSER_MYC_TARGETS_REPRESSED_BY_SERUM	-0.153013363	0.00505098124507159	-2.775859901	0.00587943183443298	0.0125149634418126	-2.97348731	Down

WP_GASTRIC_CANCER_NETWORK_1	-0.112630301	-4.46E-05	-2.776815092	0.0058626550959 9156	0.0124835335097 151	-2.97093 4583	Down
REACTOME_SIGNALING_BY_NTRK2_TRKB_	-0.081512326	0.0071228078107630 9	-2.778391786	0.0058350573373 351	0.0124332993109 643	-2.96671 909	Down
CHIANG_LIVER_CANCER_SUBCLASS_CTNNB1_DN	-0.058431252	-8.53E-05	-2.781927064	0.0057736048973 8199	0.0123108092057 884	-2.95725 885	Down
BIOCARTA_CLASSIC_PATHWAY	-0.147601086	-0.007072627	-2.784353686	0.0057317640287 2785	0.0122342019691 588	-2.95075 8742	Down
GARGALOVIC_RESPONSE_TO_OXIDIZED_PHOSPHOLIPIDS_YELLOW_DN	-0.0849475	0.0093327710101171 7	-2.785158867	0.0057179416231 048	0.0122088970260 102	-2.94860 0749	Down
KAAB_FAILED_HEART_VENTRICLE_DN	-0.08308561	-0.001381706	-2.786390729	0.0056968529290 6983	0.0121764346180 222	-2.94529 8052	Down
REACTOME_TOLL_LIKE_RECEPTOR_10_TLR10_CASCADE	-0.078843921	0.0050012746013917	-2.788064468	0.0056683125973 9267	0.0121321435489 711	-2.94080 8444	Down
AGARWAL_AKT_PATHWAY_TARGETS	-0.154647403	0.0118810222620222	-2.788255822	0.0056650579202 2648	0.0121293599554 487	-2.94029 4995	Down
DAZARD_UV_RESPONSE_CLUSTER_G3	-0.12503155	-0.001036046	-2.790167748	0.0056326316762	0.0120640941388	-2.93516	Down

				2753	351	3031	
REACTOME_ANTIVIRAL_MECHANISM_BY_IFN_STIMULATED_GENES	-0.106441432	0.00484668220821665	-2.793500379	0.00557651250565026	0.0119562739628916	-2.926209685	Down
WP_FERROPTOSIS	-0.087594835	0.00419503771457639	-2.797044453	0.00551738972093941	0.011841783540066	-2.916677205	Down
REACTOME_ACTIVATION_OF_C3_AND_C5	-0.189317409	-0.00434692	-2.800144698	0.0054661379610057	0.0117521019480299	-2.908329128	Down
REACTOME_TP53_REGULATES_TRANSCRIPTION_OF_GENES_INVOLVED_IN_CYTOCHROME_C_RELEASE	-0.087658017	0.00707645330403529	-2.800919589	0.0054533955251891	0.0117287685463786	-2.906241205	Down
MARTINEZ_RB1_AND_TP53_TARGETS_UP	-0.043100387	0.00333355209593076	-2.801818543	0.00543864674199068	0.0117011023665637	-2.903818312	Down
REACTOME_RAF_ACTIVATION	-0.089432641	0.0106662696646646	-2.803562359	0.00541013983416757	0.0116478452829269	-2.899116223	Down
PID_ALK2_PATHWAY	-0.128559119	0.00246592424565453	-2.806314088	0.00536543194465713	0.0115555989175874	-2.891690744	Down
BOYAULT_LIVER_CANCER_SUBCLASS_G5_DN	-0.112892162	0.00589435929346173	-2.809663848	0.00531146052663268	0.0114473039891698	-2.88264221	Down
WP_IL1_AND_MEGAKARYOCYTES_IN_OBESITY	-0.137588693	0.0056161	-2.8146	0.005232	0.011285	-2.8	Down

		3645453586	14138	60358558246	1877886415	6925159	
WP_OXIDATIVE_STRESS	-0.099072545	0.000938940876852782	-2.816026047	0.00521030794594637	0.0112449170446763	-2.865428276	Down
BLUM_RESPONSE_TO_SALIRASIB_DN	-0.091558313	-0.000630003	-2.817233747	0.00519130550625963	0.0112156050391067	-2.862156503	Down
VANDESLUIS_COMMD1_TARGETS_GROUP_2_UP	-0.100149725	0.00573735633896153	-2.820359768	0.00514241146579587	0.0111254611251987	-2.853681671	Down
HOFFMANN_LARGE_TO_SMALL_PRE_BII_LYMPHOCYTE_UP	-0.105911558	-0.006697932	-2.823958807	0.00508663739643858	0.0110240077931893	-2.843913477	Down
ZWANG_CLASS_1_TRANSIENTLY_INDUCED_BY_EGF	-0.055808349	0.00323871016207935	-2.82541639	0.0050642061579357	0.0109869023496354	-2.83995409	Down
SHIN_B_CELL_LYMPHOMA_CLUSTER_5	-0.105475349	0.00294891858874406	-2.825998169	0.00505527810591451	0.0109713675536403	-2.838373205	Down
NOUSHMEHR_GBM_SILENCED_BY_METHYLATION	-0.072307418	0.000674942725449959	-2.828044784	0.00502398404285599	0.0109148998788964	-2.832809436	Down
PID_RAC1_PATHWAY	-0.112609465	0.00242989337789243	-2.830651146	0.00498438603626086	0.0108326625094787	-2.825718481	Down

REACTOME_EGFR_TRANSACTIVATION_BY_GASTRIN	-0.11534841	0.00481128384056623	-2.833317986	0.00494416310062278	0.0107490088846114	-2.818456611	Down
REACTOME_TP53_REGULATES_TRANSCRIPTION_OF_DNA_REPAIR_GENES	-0.108967397	0.00105718024326348	-2.833563561	0.00494047406993393	0.0107447521205606	-2.817787581	Down
VANLOO_SP3_TARGETS_UP	-0.139711626	-0.003066362	-2.836983594	0.00488935774532615	0.010648506500084	-2.808464548	Down
HASLINGER_B_CLL_WITH_11Q23_DELETION	-0.098240559	0.0055905179698819	-2.839787295	0.00484781251242069	0.0105765807608419	-2.800813731	Down
YAMASHITA_SILENCED_BY_METHYLATION	-0.140789788	-0.012219247	-2.841219564	0.00482671330839983	0.0105379562100731	-2.796902566	Down
WEST_ADRENOCORTICAL_TUMOR_MARKERS_UP	-0.141246606	-0.016195876	-2.841288721	0.00482569665747454	0.0105379562100731	-2.796713669	Down
GENTILE_RESPONSE_CLUSTER_D3	-0.110303821	0.0128927742553362	-2.841961483	0.0048158167395199	0.0105252727120423	-2.794875841	Down
KYNG_WERNER_SYNDROM_AND_NORMAL_AGING_UP	-0.076054114	0.00843850714528049	-2.843955091	0.00478664739263026	0.0104688937160169	-2.789427366	Down
JI_RESPONSE_TO_FSH_UP	-0.068554164	-0.000214303	-2.845810063	0.0047596509630	0.0104171909477	-2.78435	Down

				9398	871	4546	
REACTOME_CHAPERONE_MEDIATED_AUTOPHAGY	-0.079154346	0.0030965369761724 5	-2.846179408	0.0047542922387 1923	0.0104091329544 022	-2.783344119	Down
BOYLAN_MULTIPLE_MYELOMA_PCA3_DN	-0.061245537	0.0005761273753828 82	-2.846220508	0.0047536962775 1024	0.0104091329544 022	-2.783231674	Down
STOSSI_RESPONSE_TO_ESTRADIOL	-0.077605982	-0.002851202	-2.850390905	0.0046935759362 3803	0.0102870853941 488	-2.7181389	Down
REACTOME_PROCESSIVE_SYNTHESIS_ON_THE_LAGGING_STRAND	-0.170416828	0.0043008149796544 1	-2.853938888	0.0046429740264 7142	0.0101833709478 12	-2.762087758	Down
STEIN_ESRRA_TARGETS_UP	-0.06792718	0.0012154665144038	-2.860310003	0.0045533531383 7717	0.0100115702904 382	-2.744593928	Down
REACTOME_CASPASE_ACTIVATION_VIA_EXTRINSIC_APOPTOTIC_SIGNALLING_PATHWAY	-0.108938096	0.0029369201102870 2	-2.86077152	0.0045469226784 5014	0.0100009741549 043	-2.743325263	Down
REACTOME_SIGNALING_BY_BMP	-0.095460077	0.0009715882354321 97	-2.863424767	0.0045101144162 4676	0.0099270497098 0272	-2.736028023	Down
GARGALOVIC_RESPONSE_TO_OXIDIZED_PHOSPHOLIPIDS_BROWN_UP	-0.129763516	0.0010097372066477	-2.866860316	0.0044628566003 9286	0.0098369854114 4832	-2.726569743	Down
REACTOME_SIGNALING_BY_FGFR3	-0.080469366	0.0010118	-2.8701	0.004418	0.009752	-2.7	Down

		8606348549	24612	37287244289	78820030336	17573041	
FIGUEROA_AML_METHYLATION_CLUSTER_4_UP	-0.050170513	-0.000867224	-2.872257753	0.00438952245782814	0.00969600209812786	-2.711688697	Down
IKEDA_MIR30_TARGETS_DN	-0.101218786	0.00392303818414983	-2.875949854	0.00433999277700611	0.00960026199817424	-2.701494182	Down
FURUKAWA_DUSP6_TARGETS_PCI35_DN	-0.103402591	-0.005312766	-2.876411621	0.00433383412225098	0.00959347660371321	-2.700218297	Down
CALVET_IRINOTECAN_SENSITIVE_VS_REVERTED_DN	-0.168560059	0.00142954765612932	-2.87650906	0.00433253558559196	0.00959347660371321	-2.699949045	Down
REACTOME_ACTIVATED_NOTCH1_TRANSMITS_SIGNAL_TO_THE_NUCLEUS	-0.105555236	0.00331495609938145	-2.876677942	0.00433028578973267	0.00959246391751275	-2.699482353	Down
KYNG_DNA_DAMAGE_BY_4NQO	-0.085131211	-0.005022362	-2.87926726	0.00429592470037613	0.00952475927526201	-2.692323749	Down
TIEN_INTESTINE_PROBIOTICS_24HR_DN	-0.082224906	0.00629185923198771	-2.879889345	0.00428770654401167	0.00951172070002875	-2.690602986	Down
IKEDA_MIR1_TARGETS_UP	-0.11313238	0.0133491473342161	-2.881980522	0.00426018568752468	0.00946081308138307	-2.684815975	Down

MANN_RESPONSE_TO_AMIFOSTINE_UP	-0.096733892	0.0129351199996838	-2.88262528	0.00425173291816135	0.00944542098175644	-2.68303091	Down
TERAO_AOX4_TARGETS_SKIN_DN	-0.105204052	0.00337027412626353	-2.888849978	0.00417091020878829	0.00928248105627426	-2.665777993	Down
RAY_TUMORIGENESIS_BY_ERBB2_CDC25A_UP	-0.039796052	0.00019491040203352	-2.891603013	0.00413561325269823	0.00920722792664918	-2.658136271	Down
REN_ALVEOLAR_RHABDOMYOSARCOMA_DN	-0.077396	0.000407356664750418	-2.911808109	0.00388478706109655	0.00868932370746893	-2.601842629	Down
BREDEMEYER_RAG_SIGNALING_NOT_VIA_ATM_UP	-0.071548033	-0.001974298	-2.91277511	0.00387313857559452	0.00866951717948618	-2.599139217	Down
BIOCARTA_ETC_PATHWAY	-0.166422517	-0.002414053	-2.913540456	0.00386394184731819	0.00865205160400577	-2.596998967	Down
REACTOME_METABOLISM_OF_VITAMINS_AND_COFACTORS	-0.041949627	-0.000885468	-2.915229455	0.00384371663746023	0.00861297803924753	-2.592273898	Down
ZWANG_CLASS_3_TRANSIENTLY_INDUCED_BY_EGF	-0.098327217	0.00530073357140551	-2.915381103	0.00384190543832973	0.00861202855027541	-2.591849528	Down
REACTOME_DNA_REPAIR	-0.098163369	0.0014803827978885	-2.920579507	0.0037802881498	0.0084923085580	-2.57728	Down

				2727	8102	9846	
TSUNODA_CISPLATIN_RESISTANCE_UP	-0.126532449	0.00936595815269591	-2.92226224	0.00376053679936554	0.00845712025857315	-2.572571626	Down
BIOCARTA_MET_PATHWAY	-0.116351899	0.00846610640382864	-2.924165491	0.00373831077752762	0.00841628400294303	-2.567232022	Down
GENTILE_UV_HIGH_DOSE_UP	-0.107073685	-0.005776041	-2.926504653	0.00371115867218846	0.00837033498483785	-2.560664985	Down
BIOCARTA_PAR1_PATHWAY	-0.124503374	0.00553935144865353	-2.927541423	0.00369918198934746	0.00834635500104677	-2.557752747	Down
REACTOME_TRAF6_MEDIATED_INDUCTION_OF_TAK1_COMPLEX_WITHIN_TLR4_COMPLEX	-0.108660652	0.00697604441849101	-2.930665234	0.00366330925224852	0.00826842201043875	-2.548972252	Down
AMIT_SERUM_RESPONSE_40_MCF10A	-0.129465691	0.00726174770735969	-2.931444592	0.00365440908615448	0.00825433668040788	-2.546780239	Down
BERENJENO_TRANSFORMED_BY_RHOA_FOREVER_DN	-0.103354993	-0.004551126	-2.934611437	0.00361844715382101	0.00817608353977684	-2.537867583	Down
DURCHDEWALD_SKIN_CARCINOGENESIS_DN	-0.075613265	0.00515170086110347	-2.936050763	0.00360220970998256	0.00814603844097489	-2.533813806	Down
XU_HGF_TARGETS_INDUCED_BY_AKT1_6HR	-0.171845008	-0.0117952	-2.9365	0.003596	0.008137	-2.5	Down

		06	76461	29579792338	88115847991	32332744	
REACTOME_ACTIVATED_NTRK2_SIGNALS_THROUGH_PI3K	-0.175966654	0.00565315757035291	-2.936893128	0.00359273769497708	0.00813788115847991	-2.53144047	Down
WP_PHOTODYNAMIC_THERAPYINDUCED_HIF1_SURVIVAL_SIGNALING	-0.087201841	0.00511603025947496	-2.940844184	0.00354861307825173	0.00804758545002138	-2.520299969	Down
FARMER_BREAST_CANCER_CLUSTER_3	-0.129018189	-0.002377182	-2.941708575	0.00353902604613986	0.00802877729107827	-2.517860832	Down
BOYAULT_LIVER_CANCER_SUBCLASS_G2	-0.094193449	0.00410405084261594	-2.942263242	0.00353288668154171	0.00801777975587913	-2.516295321	Down
ZAMORA_NOS2_TARGETS_UP	-0.093987732	0.00163802287150435	-2.945341474	0.00349899178662279	0.00794957614186224	-2.507602178	Down
WP_APOPTOSIS_MODULATION_AND_SIGNALING	-0.074852764	0.00534993250548959	-2.946174295	0.00348987274215113	0.00793176130008497	-2.505248767	Down
GROSS_HIF1A_TARGETS_DN	-0.100535886	0.0075441755907894	-2.946516022	0.00348613726457052	0.00792617362680923	-2.504282923	Down
ROSS_AML_WITH_MLL_FUSIONS	-0.102532131	0.00258390096914277	-2.946754968	0.00348352747949563	0.00792314220052378	-2.503607513	Down

WENG_POR_TARGETS_LIVER_UP	-0.095634275	6.02389259234752e-05	-2.949241989	0.00345646999648702	0.00786448286957291	-2.496574594	Down
GOBERT_OLIGODENDROCYTE_DIFFERENTIATION_UP	-0.084505524	-0.003162637	-2.949770574	0.00345074410936628	0.00785433395190191	-2.495079118	Down
HUMMERICH_MALIGNANT_SKIN_TUMOR_UP	-0.138791377	0.00316883569666646	-2.952505035	0.00342126146668585	0.00779610067676339	-2.48733875	Down
REACTOME_BUDDING_AND_MATURATION_OF_HIV_VIRION	-0.109732133	0.00258439808353986	-2.953159328	0.00341424122369742	0.00778564296137543	-2.485485662	Down
ONDER_CDH1_TARGETS_1_DN	-0.083090774	0.00325149194269108	-2.954309899	0.00340192813212515	0.0077631499691547	-2.482226083	Down
WANG_TUMOR_INVASIVENESS_UP	-0.084416428	0.00733401012598488	-2.959731093	0.00334445712096969	0.00765169382597082	-2.466685175	Down
GARCIA_TARGETS_OF_FLI1_AND_DAX1_DN	-0.091475259	0.000710171504871757	-2.959731127	0.00334445677076229	0.00765169382597082	-2.466851655	Down
SANDERSON_PPARA_TARGETS	-0.140038627	0.000577717263423126	-2.961671932	0.00332409899541544	0.00761072757821602	-2.461341165	Down
REACTOME_MRNA_SPLICING_MINOR_PATHWAY	-0.143253122	1.64053444172876e-	-2.969305038	0.0032451286825	0.0074574282609	-2.43963	Down

		05		9922	7495	5783	
KEGG_DNA_REPLICATION	-0.172753106	-0.002335832	-2.973250589	0.00320498617700877	0.00737336886608357	-2.428395739	Down
MARKEY_RB1_CHRONIC_LOF_DN	-0.087461656	-0.001085323	-2.983335865	0.00310443645127752	0.00717128286307391	-2.399601329	Down
MISSIAGLIA_REGULATED_BY_METHYLATION_DN	-0.129851442	-0.00788652	-2.986809138	0.00307048309130823	0.00710342472894155	-2.389663655	Down
BIOCARTA_CASPASE_PATHWAY	-0.113263031	0.010203656773046	-2.987664466	0.00306217414242282	0.00708684373677049	-2.387214739	Down
OUILLETTE_CLL_13Q14_DELETION_DN	-0.069380599	0.00532180663706504	-2.993888823	0.00300232683401046	0.00696391728651081	-2.369373828	Down
HOLLEMAN_PREDNISOLONE_RESISTANCE_ALL_DN	-0.147079958	-0.001671149	-2.997198736	0.00297094069035556	0.00689627107892183	-2.359872442	Down
BROWN_MYELOID_CELL_DEVELOPMENT_UP	-0.131179829	0.00219016121691946	-2.99804579	0.00296295704716132	0.00688031215552949	-2.357439322	Down
REACTOME_INNATE_IMMUNE_SYSTEM	-0.089198764	0.00196487464804994	-3.007294207	0.00287706047189057	0.00669337119528664	-2.330831819	Down
BIOCARTA_IGF1MTOR_PATHWAY	-0.121194429	0.0125446	-3.0073	0.002876	0.006693	-2.3	Down

		630430685	25359	7750474951	37119528664	30742067	
REACTOME_SIGNALING_BY_PDGFR_IN_DISEASE	-0.106111941	0.0104989198320977	-3.012693979	0.0028279717953535	0.00659895523825533	-2.31526132	Down
REACTOME_CONSTITUTIVE_SIGNALING_BY_LIGAND_RESPONSIVE_EGFR_CANCER_VARIANTS	-0.092404314	0.00452831128257772	-3.016225828	0.00279628137180574	0.0065299166571852	-2.305062922	Down
RUTELLA_RESPONSE_TO_HGF_VS_CSF2RB_AND_IL4_DN	-0.064383727	0.00735129665345906	-3.01669438	0.00279210176004907	0.00652506612372914	-2.303709115	Down
REACTOME_TGF_BETA_RECEPTOR_SIGNALING_ACTIVATES_SMADS	-0.098223904	0.00694227980023331	-3.018330589	0.00277755126196443	0.00649350684859255	-2.298979999	Down
SNIJDERS_AMPLIFIED_IN_HEAD_AND_NECK_TUMORS	-0.096062003	0.00437409409885691	-3.020371277	0.00275950140907483	0.00645617235059459	-2.293078464	Down
REACTOME_SIGNALING_BY_FGFR2	-0.070916619	0.00298489969887615	-3.024483498	0.00272345569568662	0.00637905264268937	-2.281174851	Down
HUTTMANN_B_CLL_POOR_SURVIVAL_DN	-0.145761548	0.00410786348810644	-3.024517572	0.00272315884269797	0.00637905264268937	-2.281076156	Down
REACTOME_CONVERSION_FROM_APC_C_CDC20_TO_APC_C_CDH1_IN_LATE_ANAPHASE	-0.119734517	-0.009595664	-3.025158603	0.00271757955761218	0.00637009679535454	-2.279219182	Down

BRACHAT_RESPONSE_TO_CAMPTOTHECIN_DN	-0.086616083	0.0042592946484346	-3.02542945	0.0027152253769 1673	0.0063669829673 2986	-2.27843447	Down
KAYO_CALORIE_RESTRICTION_MUSCLE_DN	-0.060012285	-0.000350416	-3.027862308	0.0026941629443 4195	0.0063223702818 6408	-2.27138 2902	Down
NAKAMURA_ADIPOGENESIS_LATE_UP	-0.062531133	0.000709304813750428	-3.028799325	0.0026860908584 3315	0.0063058116332 4303	-2.26866 5574	Down
JOHANSSON_BRAIN_CANCER_EARLY_VS_LATE_UP	-0.181036342	0.00516245826259667	-3.0289691	0.0026846306812 4107	0.0063047683081 5866	-2.26817 3145	Down
YOSHIMURA_MAPK8_TARGETS_DN	-0.054810479	0.00413896430011125	-3.029860128	0.0026769792142 1732	0.0062915600085 7513	-2.26558 8318	Down
WP_CHOLESTEROL_BIOSYNTHESIS_WITH_SKELETAL_ DYSPLASIAS	-0.179694285	0.000542803896858407	-3.031846493	0.0026599938912 5444	0.0062587498419 3189	-2.25982 3412	Down
LI_WILMS_TUMOR_ANAPLASTIC_DN	-0.180900486	0.00725629319205793	-3.032260535	0.0026564659363 9759	0.0062551912242 8674	-2.25862 1319	Down
REACTOME_MITOCHONDRIAL_PROTEIN_IMPORT	-0.163254179	-0.002122888	-3.03671399	0.0026187899674 4395	0.0061758470090 8989	-2.24568 1841	Down
WP_SENESCENCE_AND_AUTOPHAGY_IN_CANCER	-0.085702739	0.0031739916630303	-3.037521904	0.0026120079231	0.0061621942908	-2.24333	Down

		8		7712	2493	2551	
REACTOME_ACTIVATION_OF_IRF3_IRF7_MEDIATED_BY_TBK1_IKK_EPSILON	-0.101252212	0.00210634344169059	-3.04162513	0.00257781188220965	0.00608949612284969	-2.231391983	Down
LEE_EARLY_T_LYMPHOCYTE_UP	-0.140183693	-0.012670745	-3.042617192	0.00256960611560595	0.00607601720364424	-2.228502777	Down
RIZ_ERYTHROID_DIFFERENTIATION	-0.07085108	0.00524615995226157	-3.043648993	0.00256109713156519	0.00605820499071079	-2.225496903	Down
ZHU_SKIL_TARGETS_DN	-0.17005566	-0.02383959	-3.047703846	0.00252790822116181	0.00599569213937766	-2.213674962	Down
REACTOME_PI3K_EVENTS_IN_ERBB4_SIGNALING	-0.148147072	0.00168694020367383	-3.050948604	0.0025016353110 09	0.00593564616797893	-2.204204263	Down
YAO_TEMPORAL_RESPONSE_TO_PROGESTERONE_CLUSTER_10	-0.148076894	-0.003055732	-3.063529497	0.00240212457560909	0.00571263878958071	-2.167394536	Down
ELVIDGE_HYPOXIA_UP	-0.066681784	0.00108389798842968	-3.066968394	0.00237556519960696	0.00565380874001549	-2.157308248	Down
GAVIN_FOXP3_TARGETS_CLUSTER_P7	-0.072038964	0.00433635447724631	-3.068001752	0.00236763730894577	0.00563926507161412	-2.15427534	Down
REACTOME_SIGNALING_BY_MRAS_COMPLEX_MUTAN	-0.166883261	0.0118804	-3.0683	0.002364	0.005634	-2.1	Down

TS		993085847	7541	77661958724	61361910863	53178419	
MILI_PSEUDOPODIA	-0.100447064	0.00741690738542318	-3.068710368	0.00236221492101663	0.00563067128062606	-2.152195002	Down
DANG_MYC_TARGETS_UP	-0.145070453	-0.003077355	-3.07189799	0.00233796404724687	0.00557714943937792	-2.142831307	Down
GARGALOVIC_RESPONSE_TO_OXIDIZED_PHOSPHOLIPIDS_MAGENTA_DN	-0.208024763	0.00510918903201401	-3.07618614	0.00230570207278345	0.00550442029452573	-2.13022048	Down
REACTOME_M_PHASE	-0.094690376	0.00137551615614968	-3.076275291	0.00230503571824814	0.00550442029452573	-2.129958127	Down
KALMA_E2F1_TARGETS	-0.203448291	-0.00099129	-3.081875819	0.00226352841501785	0.005407898719 0207	-2.113462621	Down
REACTOME_FRS_MEDIATED_FGFR4_SIGNALING	-0.098882479	-0.00199638	-3.084197199	0.0022465266262363	0.00537348391870857	-2.106617141	Down
REACTOME_SIGNALLING_TO_P38_VIA_RIT_AND_RIN	-0.2061011	0.00406238365434076	-3.085242477	0.00223890949245014	0.00535870806176118	-2.103533169	Down
REACTOME_INTERFERON_ALPHA_BETA_SIGNALING	-0.169126734	-0.013120556	-3.088980882	0.00221186133658624	0.00530078120316247	-2.092495457	Down

DUNNE_TARGETS_OF_AML1_MTG8_FUSION_DN	-0.113751196	0.00586649322299251	-3.089237829	0.00221001337308209	0.00529839822584802	-2.091736358	Down
SWEET_LUNG_CANCER_KRAS_UP	-0.051950023	0.00225237119263462	-3.089842432	0.00220567067999563	0.00529200809454665	-2.089949947	Down
FLECHNER_PBL_KIDNEY_TRANSPLANT_REJECTED_VS_OK_UP	-0.103820431	0.0110173771474	-3.091629202	0.00219288272821239	0.00526546347930922	-2.084668699	Down
GAVIN_FOXP3_TARGETS_CLUSTER_P4	-0.058387698	0.00480841241000121	-3.095164134	0.00216778421197978	0.00521124577992195	-2.07421193	Down
REACTOME_NEUTROPHIL_DEGRANULATION	-0.127170597	0.0015580379817321	-3.096459485	0.00215865351420971	0.00519331874523242	-2.070377329	Down
MANNE_COVID19_NONICU_VS_HEALTHY_DONOR_PLATELETS_UP	-0.107373355	-0.0053852	-3.097202533	0.00215343191381028	0.00518276536991873	-2.068177025	Down
CASTELLANO_HRAS_TARGETS_DN	-0.187423368	0.00214349310662547	-3.100494815	0.00213043594143326	0.00513538481105874	-2.058422041	Down
BUYTAERT_PHOTODYNAMIC_THERAPY_STRESS_DN	-0.070253717	0.00130037346208899	-3.102468852	0.00211675645335302	0.00510439289275921	-2.052568367	Down
WP_MITOCHONDRIAL_LCFATTY_ACID_BETAOXIDATION	-0.117573955	-0.001830263	-3.105605062	0.0020951898909	0.0050543504286	-2.04326	Down

				215	6294	1321	
MARTINEZ_TP53_TARGETS_UP	-0.046910861	0.00342209321216778	-3.106687493	0.00208779357137602	0.00504238704184083	-2.040047055	Down
REACTOME_INHIBITION_OF_REPLICATION_INITIATION_OF_DAMAGED_DNA_BY_RB1_E2F1	-0.161889056	0.00428094517842351	-3.107980799	0.00207898789938723	0.00502307430575965	-2.036205228	Down
ROVERSI_GLIOMA_LOH_REGIONS	-0.080922778	0.00158725442242184	-3.109182783	0.00207083472950262	0.00500532366278144	-2.032633341	Down
REACTOME_CELL_CYCLE	-0.095431433	-3.90E-05	-3.109418253	0.00206924097656115	0.00500341984476628	-2.031933456	Down
KEGG_FOLATE_BIOSYNTHESIS	-0.131090676	-0.006534151	-3.113773798	0.00203996413287061	0.00493839991136031	-2.018978571	Down
PUJANA_XPRSS_INT_NETWORK	-0.160641296	0.00085314849662575	-3.119247317	0.00200371441721771	0.00485822476080873	-2.002674492	Down
REACTOME_PROTEIN_METHYLATION	-0.141265208	0.00964814550266581	-3.119348295	0.00200305128588565	0.00485822476080873	-2.002373457	Down
EINAV_INTERFERON_SIGNATURE_IN_CANCER	-0.228456419	-0.022336699	-3.120211058	0.00199739368441666	0.00484668592618227	-1.999801017	Down
REACTOME_BBSOME_MEDIATED_CARGO_TARGETING	-0.152706312	0.0015070	-3.1242	0.001970	0.004789	-1.9	Down

_TO_CILIUM		2642650397	74688	94402710157	99591864505	87675869	
BLANCO_MELO_BRONCHIAL_EPITHELIAL_CELLS_INFLUENZA_A_INFECTION_UP	-0.086488564	0.000100089105830413	-3.124956417	0.00196653853591464	0.00478116125829306	-1.985640273	Down
YAO_TEMPORAL_RESPONSE_TO_PROGESTERONE_CLUSTER_5	-0.115568255	-0.001401901	-3.127244612	0.00195181803821495	0.00474918165732799	-1.978804853	Down
ST_G_ALPHA_S_PATHWAY	-0.144929598	-0.003538782	-3.128918646	0.00194111307975212	0.00473046285277637	-1.973801136	Down
GABRIELY_MIR21_TARGETS	-0.125767665	0.010272026829438	-3.12965957	0.0019363924080888	0.00472266706365704	-1.971585704	Down
WP_GLYCOSYLATION_AND_RELATED_CONGENITAL_DEFECTS	-0.160341428	0.00101771979336091	-3.134670026	0.00190474629491709	0.00465279821037008	-1.956591169	Down
REACTOME_CHROMOSOME_MAINTENANCE	-0.138057096	-0.000758359	-3.135237061	0.00190119513456159	0.0046459520473322	-1.954892826	Down
WP_REGULATION_OF_SISTER_CHROMATID_SEPARATION_AT_THE_METAPHASEANAPHASE_TRANSITION	-0.159061803	-0.00122598	-3.135992742	0.00189647204187555	0.00463806223952778	-1.952629018	Down
ONGUSAHA_BRCA1_TARGETS_DN	-0.151326223	0.00045352606534081	-3.13662346	0.00189253828128573	0.00463209192111221	-1.950739176	Down

REACTOME_SYNTHESIS_OF_UDP_N_ACETYL_GLUCOSAMINE	-0.167389035	0.00959919922715069	-3.142036811	0.0018590840182778 8	0.00455739909220015	-1.934504428	Down
PID_P38_ALPHA_BETA_DOWNSTREAM_PATHWAY	-0.108702367	0.00707991061805499	-3.142059601	0.00185894434062765	0.00455739909220015	-1.934436026	Down
TURASHVILI_BREAST_CARCINOMA_DUCTAL_VS_LOBULAR_DN	-0.171935094	0.0040122092304122	-3.142718271	0.00185491155818303	0.00455076523385062	-1.932458872	Down
PID_CDC42_PATHWAY	-0.110424256	0.00680293245716042	-3.145862313	0.00183577290608173	0.00450619833753081	-1.923015965	Down
HU_GENOTOXIC_DAMAGE_4HR	-0.106369979	-0.005826286	-3.148298642	0.00182106792956691	0.00447480943737998	-1.915692584	Down
REACTOME_PINK1_PRKN_MEDIATED_MITOPHAGY	-0.108425655	0.00329586229406803	-3.151991149	0.00179898837639724	0.00442581088081556	-1.904583196	Down
FARMER_BREAST_CANCER_CLUSTER_6	-0.126931552	0.00344161763422279	-3.152139584	0.0017981059971653	0.00442539410166732	-1.904136358	Down
MULLIGHAN_NPM1_SIGNATURE_3_UP	-0.049955634	0.00382191408162188	-3.155826724	0.00177631548417587	0.00437523421042841	-1.893030546	Down
HAHTOLA_MYCOSIS_FUNGOIDES_SKIN_DN	-0.110567426	0.0033029584047924	-3.155952652	0.0017755755923	0.0043751479562	-1.89265	Down

		3		7314	7634	1032	
CHANG_POU5F1_TARGETS_DN	-0.163475305	-0.003968397	-3.156987607	0.00176950550172211	0.00436365540293569	-1.889531423	Down
KYNG_DNA_DAMAGE_BY_4NQO_OR_UV	-0.079575249	0.00675809605192522	-3.158902215	0.00175832670318599	0.00433781154478356	-1.883757812	Down
KIM_MYCL1_AMPLIFICATION_TARGETS_DN	-0.114460408	-0.0020434	-3.159558836	0.00175450795636303	0.00433183408319225	-1.881776985	Down
VECCHI_GASTRIC_CANCER_ADVANCED_VS_EARLY_DN	-0.071717615	-0.003481021	-3.160400161	0.00174962622137689	0.00432322052391974	-1.879238402	Down
IWANAGA_E2F1_TARGETS_NOT_INDUCED_BY_SERUM	-0.183980319	0.0112431928689768	-3.164369673	0.00172676229773337	0.00427182685613034	-1.867252467	Down
BONOME_OVARIAN_CANCER_POOR_SURVIVAL_UP	-0.112034777	0.000559704822049267	-3.166004152	0.00171742843755043	0.00425042994891367	-1.862313097	Down
REACTOME_REGULATION_OF_TLR_BY_ENDOGENOUS_LIGAND	-0.17365134	0.00162859826983907	-3.166466743	0.00171479524702625	0.00424560594267726	-1.860914724	Down
MYLLYKANGAS_AMPLIFICATION_HOT_SPOT_7	-0.167801992	-0.000638002	-3.167520966	0.00170880826992874	0.00423416085087732	-1.857727188	Down
HESS_TARGETS_OF_HOXA9_AND_MEIS1_DN	-0.141957882	0.0032152	-3.1682	0.001704	0.004225	-1.8	Down

		9395944331	24493	82367019774	97464892865	55599464	
REACTOME_CYCLIN_A_B1_B2_ASSOCIATED_EVENTS_DURING_G2_M_TRANSITION	-0.124709579	-0.002507	-3.168750572	0.00170184970334681	0.00422028809775215	-1.854008118	Down
WONG_EMBRYONIC_STEM_CELL_CORE	-0.153658336	-0.002782002	-3.17054622	0.0016917348747625	0.00419932131846733	-1.848574591	Down
IVANOV_MUTATED_IN_COLON_CANCER	-0.129930886	0.0059959090015097	-3.174299378	0.00167077274684786	0.00414985451768094	-1.837208511	Down
KUMAR_AUTOPHAGY_NETWORK	-0.067962618	0.00514335380663954	-3.177985636	0.0016504183681475	0.00410258182262376	-1.826032873	Down
DUTERTRE_ESTRADIOL_RESPONSE_24HR_UP	-0.093887826	-0.006850341	-3.179671618	0.00164118555943969	0.00408453839913478	-1.820917459	Down
YAGI_AML_RELAPSE_PROGNOSIS	-0.106472295	9.12370928788742e-05	-3.182707733	0.00162467953683092	0.00404670380622372	-1.811699275	Down
KEGG_UBIQUITIN_MEDIATED_PROTEOLYSIS	-0.086636929	0.00421993287609203	-3.183176199	0.00162214641651	0.0040420163807421	-1.810276202	Down
MARTENS_TRETINOIN_RESPONSE_DN	-0.103462836	-0.002499161	-3.183899348	0.00161824332848715	0.00403553087180382	-1.808079088	Down

REACTOME_REMOVAL_OF_THE_FLAP_INTERMEDIATE_FROM_THE_C_STRAND	-0.190742072	0.0116075473060738	-3.184450691	0.00161527338035165	0.00402974351762167	-1.806403654	Down
GROSS_HYPOXIA_VIA_HIF1A_ONLY	-0.172121184	0.00850276786981062	-3.188321078	0.0015945661234392	0.00398128396145901	-1.794634649	Down
BURTON_ADIPOGENESIS_10	-0.107868527	-0.00063802	-3.18914103	0.00159021089783929	0.00397360670003562	-1.792139652	Down
DIRMEIER_LMP1_RESPONSE_LATE_DN	-0.112589291	-0.001465285	-3.190598397	0.00158249715956421	0.00395592423254734	-1.787703617	Down
GRASEMANN_RETINOBLASTOMA_WITH_6P_AMPLIFICATION	-0.156970871	0.00938465363827751	-3.193449361	0.00156750726514937	0.00392161128366874	-1.779020214	Down
REACTOME_NICOTINAMIDE_SALVAGING	-0.13889133	-0.002693534	-3.194383431	0.00156262473974275	0.00391097248370293	-1.776173679	Down
KEGG_TYPE_I_DIABETES_MELLITUS	-0.105538625	-0.003144747	-3.195638108	0.00155608851331688	0.00389933040054818	-1.772348896	Down
WORSCHECH_TUMOR_REJECTION_UP	-0.088334858	-0.000820187	-3.195702523	0.00155575362893154	0.00389933040054818	-1.772152495	Down
REACTOME_MITOTIC_G2_G2_M_PHASES	-0.096593995	-0.000478891	-3.195925769	0.0015545935146	0.0038987320991	-1.77147	Down

				371	3232	1786	
REACTOME_SYNTHESIS_OF_GLYCOSYLPHOSPHATIDYLINOSITOL_GPI_	-0.146660505	0.00234725183551242	-3.196591989	0.00155113622484009	0.00389163401276575	-1.769440131	Down
MODY_HIPPOCAMPUS_PRENATAL	-0.160600857	0.00351261935893333	-3.197285778	0.00154754343499488	0.00388733391380544	-1.767323985	Down
DACOSTA_UV_RESPONSE_VIA_ERCC3_DN	-0.120853681	0.0112753933529642	-3.198743501	0.00154001969326307	0.00387000090529712	-1.762876352	Down
GAVIN_FOXP3_TARGETS_CLUSTER_T7	-0.112168368	0.00594072256583142	-3.201091705	0.00152797110692025	0.00384146286958183	-1.755707832	Down
WP_PHOTODYNAMIC_THERAPYINDUCED_AP1_SURVIVAL_SIGNALING	-0.094295222	-0.00074259	-3.203993468	0.0015132028117651	0.0038087793400754	-1.746842683	Down
BANDRES_RESPONSE_TO_CARMUSTIN_WITHOUT_MGMT_48HR_UP	-0.106347343	0.0095316777061884	-3.204544072	0.00151041552156986	0.00380484948960393	-1.745159697	Down
KEGG_O_GLYCAN_BIOSYNTHESIS	-0.096303589	0.000747675961574368	-3.206570267	0.00150019932868503	0.00378372093992198	-1.738964082	Down
REACTOME_ACTIVATION_OF_THE_MRNA_UPON_BINDING_OF_THE_CAP_BINDING_COMPLEX_AND_EIFS_AND_SUBSEQUENT_BINDING_TO_43S	-0.208151541	-0.001242274	-3.208331975	0.0014913687552931	0.00376297799353833	-1.733574254	Down
KYNG_RESPONSE_TO_H2O2	-0.064107775	0.0033049	-3.2086	0.001489	0.003760	-1.7	Down

		0729425193	51127	77416472548	48322100491	32597536	
PID_MYC_PATHWAY	-0.10869439	0.00927630205436011	-3.210804813	0.00147905483940947	0.00373494442156818	-1.726004136	Down
WP_SPHINGOLIPID_PATHWAY	-0.119387952	0.00743971258597751	-3.211809883	0.00147407690002465	0.00372540525995643	-1.722925762	Down
GUTIERREZ_WALDENSTROEMS_MACROGLOBULINEMIA_1_DN	-0.158007183	-0.000400043	-3.223024002	0.00141957826837905	0.00359792662794152	-1.68851806	Down
REACTOME_TOLL_LIKE_RECEPTOR_9_TLR9_CASCADE	-0.098335249	0.00425061375043363	-3.223606377	0.00141679969381091	0.00359235118442988	-1.686728152	Down
REACTOME_CELL_CYCLE_MITOTIC	-0.09963607	0.000284690787524128	-3.223765927	0.00141603934501632	0.00359189056580152	-1.68623773	Down
REACTOME_RNA_POLYMERASE_III_TRANSCRIPTION_INITIATION_FROM_TYPE_3_PROMOTER	-0.150087343	-0.010732533	-3.223865827	0.0014155634512125	0.00359189056580152	-1.685930644	Down
SCHMAHL_PDGF_SIGNALING	-0.179249801	0.01524385529696	-3.23221959	0.00137628938130966	0.00349964284710735	-1.660220772	Down
WP_NRF2ARE_REGULATION	-0.123102279	0.0108107418141392	-3.233587597	0.00136995499384639	0.00348639222911215	-1.656004665	Down

MASSARWEH_RESPONSE_TO_ESTRADIOL	-0.081983379	0.00201791139953408	-3.234403898	0.0013661881002830 9	0.0034782319681940 7	-1.65348809	Down
REACTOME_RESOLUTION_OF_D_LOOP_STRUCTURES_THROUGH_SYNTHESIS_DEPENDENT_STRAND_ANNEALING_SDSA_	-0.121499476	0.00075022095032844	-3.234463527	0.0013659133169926	0.0034782319681940 7	-1.653304239	Down
REACTOME_SIGNALING_BY_ACTIVIN	-0.105537879	-0.005401978	-3.235149735	0.0013627547727307 1	0.0034723394393840 3	-1.651188238	Down
ZHAN_MULTIPLE_MYELOMA_CD1_VS_CD2_DN	-0.101768863	0.00345449315927922	-3.238896047	0.0013456299967262 9	0.0034315223458011 9	-1.639628723	Down
LIU_TOPBP1_TARGETS	-0.131959162	0.00469058066286339	-3.239044059	0.0013449575321039 3	0.0034315223458011 9	-1.639171765	Down
WP_PILOCYTIC_ASTROCYTOMA	-0.194593793	0.0187358425642905	-3.239604714	0.0013424131413401 8	0.0034275435492795 2	-1.637440686	Down
KEGG_RIBOFLAVIN_METABOLISM	-0.137663691	0.00425584326358874	-3.242366241	0.0013299456672408 5	0.0033999064071515 4	-1.628910138	Down
CROONQUIST_NRAS_SIGNALING_DN	-0.152490765	-0.014225519	-3.244219719	0.0013216380937103 9	0.0033800608354595 9	-1.623180838	Down
ICHIBA_GRAFT_VERSUS_HOST_DISEASE_35D_UP	-0.117839825	-0.002884544	-3.244273406	0.0013213981803	0.0033800608354	-1.62301	Down

				6197	5959	4841	
BIOCARTA_CELLCYCLE_PATHWAY	-0.124569321	-0.006630938	-3.245514369	0.00131586385904303	0.00336945832222776	-1.619177137	Down
REACTOME_TOLL_LIKE_RECEPTOR_4_TLR4_CASCADE	-0.092963712	0.00314801720984993	-3.247943226	0.00130509408147951	0.00334464036488164	-1.61166192	Down
REACTOME_DOWNSTREAM_SIGNALING_OF_ACTIVATED_FGFR4	-0.093691146	-2.21E-05	-3.249389957	0.00129871806118221	0.00333105082882561	-1.607183057	Down
PEDERSEN_METASTASIS_BY_ERBB2_ISOFORM_3	-0.151480826	0.00802787733278617	-3.252045038	0.00128709176258619	0.00330396136078265	-1.598958529	Down
WP_OVERVIEW_OF_INTERFERONSMEDIATED_SIGNALING_PATHWAY	-0.113293458	-0.002743073	-3.253223115	0.00128196409357323	0.00329216016913903	-1.595307257	Down
REACTOME_GRB2_EVENTS_IN_ERBB2_SIGNALING	-0.10576979	-0.00273089	-3.254089051	0.00127820714046409	0.00328387074538932	-1.59262264	Down
PEDERSEN_METASTASIS_BY_ERBB2_ISOFORM_7	-0.04515566	-6.86E-05	-3.258115332	0.00126087253586584	0.00324336337758776	-1.58013148	Down
REACTOME_ERYTHROPOIETIN_ACTIVATES_PHOSPHOINOSITIDE_3_KINASE_PI3K_	-0.208228124	0.00176831973056232	-3.262613923	0.00124176271623767	0.00319951065989506	-1.566158117	Down
MARTINELLI_IMMATURE_NEUTROPHIL_UP	-0.258703082	-0.0260288	-3.2634	0.001238	0.003192	-1.5	Down

		7	61576	1921687116	96169139714	6352317	
MORI_IMMATURE_B_LYMPHOCYTE_DN	-0.14642224	-0.010238924	-3.263582823	0.00123768222335787	0.00319296169139714	-1.56314622	Down
REACTOME_TOLL_LIKE_RECEPTOR_TLR1_TLR2_CASCADE	-0.099723199	0.00538931356894208	-3.26541784	0.00122998819902181	0.00317576403965406	-1.557439666	Down
PHONG_TNF_RESPONSE_VIA_P38_COMPLETE	-0.077270046	0.00531277853291258	-3.265540346	0.00122947613068742	0.00317576293931618	-1.557058591	Down
REACTOME_HIV_LIFE_CYCLE	-0.114989396	0.00395529933917361	-3.265640867	0.00122905610331328	0.00317576293931618	-1.556745892	Down
BURTON_ADIPOGENESIS_9	-0.087004902	0.00544034091317709	-3.270039161	0.00121080783369761	0.00313145175990045	-1.543055093	Down
ZHANG_RESPONSE_TO_CANTHARIDIN_DN	-0.155445003	0.00152956933820471	-3.272559656	0.0012004641802037	0.00310988362542754	-1.535201735	Down
RAMJAUN_APOPTOSIS_BY_TGFB1_VIA_MAPK1_UP	-0.238931415	0.0183909253364692	-3.274770342	0.00119145954879345	0.00308784526904423	-1.528309074	Down
KEGG_ALLOGRAFT_REJECTION	-0.125262629	-0.00187872	-3.276636093	0.00118390877806325	0.00307084069596263	-1.522488532	Down

RODRIGUES_DCC_TARGETS_DN	-0.086960511	0.005173795137898	-3.277412432	0.0011807800189549	0.00306574343716321	-1.520065701	Down
KIM_MYC_AMPLIFICATION_TARGETS_UP	-0.116906402	0.00131995836746009	-3.278825447	0.00117510506833315	0.003054387420077	-1.515654545	Down
REACTOME_ENDOSOMAL_VACUOLAR_PATHWAY	-0.201480835	0.00253153224082174	-3.280121916	0.00116992048707032	0.00304218536373919	-1.511605674	Down
WP_DOPAMINE_METABOLISM	-0.120161353	0.000195167378878876	-3.280820394	0.00116713609008935	0.00303621697870269	-1.509423715	Down
RIZKI_TUMOR_INVASIVENESS_3D_DN	-0.038361883	0.00182868007689675	-3.281618817	0.001163960819430 3	0.00302922633383811	-1.506929017	Down
DACOSTA_UV_RESPONSE_VIA_ERCC3_COMMON_DN	-0.125231274	0.0104859287729987	-3.285245563	0.00114963828121143	0.002994462782828 1	-1.495590061	Down
ZHAN_MULTIPLE_MYELOMA_HP_UP	-0.105021638	0.000788284182490093	-3.287371743	0.00114131801767117	0.00297403901582072	-1.488937215	Down
REACTOME_SYNTHESIS_OF_WYBUTOSINE_AT_G37_OF_TRNA_PHE_	-0.23712145	0.00361199932847084	-3.28762474	0.00114033171087785	0.00297396593673059	-1.488145318	Down
LEE_BMP2_TARGETS_DN	-0.134282344	0.0023331042540806	-3.290702236	0.0011283973968	0.0029453165021	-1.47850	Down

		3		1959	2749	8065	
WAKABAYASHI_ADIPOGENESIS_PPARG_RXRA_BOUND_WITH_H4K20ME1_MARK	-0.084499858	0.00554345358718005	-3.295807324	0.00110885592413261	0.00290040822633422	-1.462502983	Down
REACTOME_BETA_CATENIN_INDEPENDENT_WNT_SIGNALING	-0.066661796	0.00334955118516071	-3.297109841	0.00110392071944065	0.00288871665496127	-1.458415765	Down
BENPORATH_SOX2_TARGETS	-0.061335172	0.00258567862153574	-3.29735725	0.00110298560107527	0.00288748697843703	-1.457639239	Down
REACTOME_HOST_INTERACTIONS_OF_HIV_FACTORS	-0.118421783	0.00368226031722574	-3.297385264	0.0011028797658443	0.00288748697843703	-1.457551311	Down
CARDOSO_RESPONSE_TO_GAMMA_RADIATION_AND_3AB	-0.135475438	0.01271925713629	-3.297932777	0.00110081316655511	0.00288423272469716	-1.455832669	Down
TIEN_INTESTINE_PROBIOTICS_2HR_UP	-0.090735749	0.00304679422939257	-3.300003074	0.00109303129620297	0.00286868721164137	-1.449331635	Down
LAU_APOPTOSIS_CDKN2A_UP	-0.090474674	0.00962563103127958	-3.302411437	0.00108404306660952	0.0028475054229561	-1.441764281	Down
BILANGES_SERUM_AND_RAPAMYCIN_SENSITIVE_GENES	-0.212747272	-0.002322136	-3.303019566	0.00108178436823085	0.00284277543336532	-1.439852664	Down
WP_SYNTHESIS_AND_DEGRADATION_OF_KETONE_BO	-0.203892875	0.0022836	-3.3043	0.001076	0.002832	-1.4	Down

DIES		8896056054	68682	78912683577	0466568939	35610637	
TAKEDA_TARGETS_OF_NUP98_HOXA9_FUSION_8D_DN	-0.110901174	0.00716941024585017	-3.308538897	0.00106148378385231	0.00279653219285707	-1.422488115	Down
MORI_SMALL_PRE_BII_LYMPHOCYTE_UP	-0.064690316	0.00377008849844383	-3.308678007	0.0010609767310584	0.00279638325676412	-1.422050109	Down
KEGG_GRAFT_VERSUS_HOST_DISEASE	-0.141776714	-0.002882845	-3.310418667	0.00105465110780339	0.00278089185477299	-1.416567999	Down
REACTOME_RHO_GTPASES_ACTIVATE_CIT	-0.139188759	0.00138766622675126	-3.312099371	0.00104857666771528	0.00276840296746437	-1.411272187	Down
MORI_LARGE_PRE_BII_LYMPHOCYTE_UP	-0.146657898	-0.007700524	-3.315014632	0.00103811739572371	0.00274663029635852	-1.402080473	Down
YAO_TEMPORAL_RESPONSE_TO_PROGESTERONE_CLUSTER_17	-0.150098803	0.00168622881218049	-3.317537316	0.00102914501650332	0.00272754189472079	-1.394120514	Down
BOCHKIS_FOXA2_TARGETS	-0.036037181	0.00128585418087131	-3.318653884	0.0010251968257448	0.00272056293176484	-1.390595566	Down
BLANCO_MELO_SARS_COV_1_INFECTION_MCR5_CELLS_UP	-0.17965114	0.00316005344707578	-3.326470993	0.00099794774914082	0.00266190875759221	-1.365886696	Down

IKEDA_MIR1_TARGETS_DN	-0.232548618	0.00535041069709994	-3.33441577	0.000970945567910643	0.00259322682444981	-1.340719349	Down
GENTILE_UV_RESPONSE_CLUSTER_D4	-0.15033292	0.0146854709964652	-3.337719429	0.000959918843698095	0.00256598460931 7	-1.330237774	Down
PUJANA_BRCA_CENTERED_NETWORK	-0.172181951	-3.77E-05	-3.340438983	0.000950929329493687	0.00254414583972729	-1.321602218	Down
WHITFIELD_CELL_CYCLE_G1_S	-0.111805319	0.0012978605349464	-3.343266297	0.000941666783374046	0.00252698907237471	-1.312617616	Down
GARGALOVIC_RESPONSE_TO_OXIDIZED_PHOSPHOLIPIDS_PINK_DN	-0.112516555	-0.002696533	-3.344310282	0.000938267883145659	0.002520047057847 3	-1.309298281	Down
REACTOME_RESOLUTION_OF_ABASIC_SITES_AP_SITES_	-0.161331836	-0.001621044	-3.353533543	0.000908732303666702	0.00244813429203959	-1.279931588	Down
WP_CILIARY_LANDSCAPE	-0.08719366	0.00300621669077779	-3.356095354	0.000900683700262409	0.00243067118588207	-1.271761606	Down
PYEON_HPV_POSITIVE_TUMORS_UP	-0.158551819	0.00140479043154101	-3.358144618	0.000894293277183581	0.00241552583615252	-1.265222078	Down
AMIT_SERUM_RESPONSE_60_MCF10A	-0.11284105	0.0092654253109019	-3.360627014	0.0008866087679	0.0023968556719	-1.25729	Down

		4		8646	9127	5429	
CHEN_LVAD_SUPPORT_OF_FAILING_HEART_DN	-0.103394258	0.00404659236077311	-3.361741072	0.000883180134850544	0.00238862705752389	-1.253736328	Down
UDAYAKUMAR_MED1_TARGETS_UP	-0.128298538	0.00175066660725194	-3.362339327	0.000881344044509396	0.00238574028969464	-1.251824624	Down
FEVR_CTNNB1_TARGETS_DN	-0.133534013	0.0024729338459166	-3.362457905	0.000880980540068769	0.00238574028969464	-1.251445673	Down
WP_AMYOTROPHIC_LATERAL_SCLEROSIS_ALS	-0.097789772	0.000481872639367708	-3.365228838	0.000872525898033527	0.00236393201619123	-1.242586878	Down
REACTOME_ALPHA_LINOLENIC_OMEGA3_AND_LINOLEIC_OMEGA6_ACID_METABOLISM	-0.175372537	0.00423430013852034	-3.365355414	0.000872141501577604	0.00236392240187432	-1.242182047	Down
JIANG_HYPOXIA_NORMAL	-0.051588996	0.00388743555994088	-3.365540156	0.000871580745911768	0.00236343455215131	-1.241591158	Down
POMEROY_MEDULLOBLASTOMA_DESMOPLASIC_VS_CLASSIC_DN	-0.089298884	0.000787415657604245	-3.368015609	0.00086409925915589	0.00234622226665819	-1.233670669	Down
STEGMEIER_PREMITOTIC_CELL_CYCLE_REGULATORS	-0.178943924	0.00587477008645755	-3.369811717	0.000858708461222843	0.00233260543492787	-1.227920463	Down
BENPORATH_OCT4_TARGETS	-0.05571565	0.0005430	-3.3774	0.000836	0.002276	-1.2	Down

		58875243056	80475	042469737968	0156182735	03337348	
WP_TARGET_OF_RAPAMYCIN_TOR_SIGNALING	-0.114275614	0.00304485381840864	-3.381397537	0.000824681561539864	0.0022470581441 9576	-1.190760905	Down
FISCHER_DREAM_TARGETS	-0.13537216	0.000503703115718204	-3.383821222	0.000817724278340348	0.0022290797521557	-1.182972507	Down
REACTOME_BETA_CATENIN_PHOSPHORYLATION_CASCADE	-0.130060325	0.0125087626046077	-3.384440426	0.000815955628575738	0.00222621388420642	-1.180981898	Down
WU_APOPTOSIS_BY_CDKN1A_VIA_TP53	-0.158271584	-0.012584877	-3.385699364	0.000812370689998756	0.00221740759578816	-1.176933654	Down
PUJANA_CHEK2_PCC_NETWORK	-0.166291418	0.000682550528947388	-3.388141433	0.000805458537511711	0.00220047585490105	-1.169076982	Down
MATZUK_MEIOTIC_AND_DNA_REPAIR	-0.100666069	-0.004323567	-3.389200859	0.00080247698320333	0.00219426195363131	-1.165666954	Down
LU_AGING_BRAIN_UP	-0.067389194	0.00419825649981189	-3.392125761	0.000794298775381264	0.00217381503474052	-1.156247341	Down
GAUSSMANN_MLL_AF4_FUSION_TARGETS_C_UP	-0.074812831	0.0024961327057843	-3.392416443	0.000793490271795327	0.00217256026335845	-1.155310793	Down

REACTOME_NONCANONICAL_ACTIVATION_OF_NOTCH3	-0.186971472	0.00687176781461828	-3.401184668	0.000769460268877725	0.00211235731487131	-1.127025843	Down
REACTOME_FRS_MEDIATED_FGFR1_SIGNALING	-0.104536571	-0.002152714	-3.401313798	0.000769111508980707	0.00211233413108108	-1.126608789	Down
BOYAULT_LIVER_CANCER_SUBCLASS_G12_UP	-0.108250146	0.00322016505287993	-3.404226952	0.000761282615197954	0.00209175794268867	-1.117196224	Down
SHAFFER_IRF4_TARGETS_IN_ACTIVATED_B_LYMPHOCYTE	-0.107522098	0.00486113168476371	-3.410852502	0.000743753084047196	0.00204568445623485	-1.095761129	Down
LEE_SP4_THYMOCYTE	-0.149961319	0.00364786037773229	-3.411846944	0.000741154807113361	0.00204006558215194	-1.092540591	Down
TURASHVILI_BREAST_CARCINOMA_DUCTAL_VS_LOBULAR_UP	-0.127307143	0.00736711347195251	-3.413810412	0.000736049526427354	0.00202871199402069	-1.086179287	Down
VERHAAK_AML_WITH_NPM1_MUTATED_UP	-0.149060624	0.00526024238193989	-3.414232945	0.000734955188347639	0.00202839788976158	-1.084809915	Down
FUJII_YBX1_TARGETS_UP	-0.109629752	-0.003813735	-3.41449647	0.000734273440409433	0.00202741781344366	-1.083955785	Down
PID_P75_NTR_PATHWAY	-0.091107084	0.0083215741713926	-3.415835143	0.0007308193640	0.0020187787239	-1.07961	Down

		6		72798	8747	5987	
REACTOME_PURINE_SALVAGE	-0.138321131	0.00630113629495777	-3.417682903	0.000726076650058966	0.00200746448414967	-1.073623229	Down
LANDIS_ERBB2_BREAST_PRENEOPLASTIC_DN	-0.107665524	0.0056922830023593	-3.418472659	0.000724058334833974	0.00200456292788335	-1.071060939	Down
GRANDVAUX_IFN_RESPONSE_NOT_VIA_IRF3	-0.276053085	-0.025333227	-3.419645611	0.000721070387650246	0.00199718156900717	-1.067254405	Down
REACTOME_SIGNALING_BY_THE_B_CELL_RECEPTOR_BCR_	-0.111474318	0.00208802107222849	-3.421603362	0.000716108906203408	0.0019843249914306	-1.060898326	Down
REACTOME_ABORTIVE_ELONGATION_OF_HIV_1_TRANSCRIPT_IN_THE_ABSENCE_OF_TAT	-0.157264628	0.00645111436294074	-3.422827903	0.000713021809876471	0.00197753636009976	-1.056921003	Down
TANG_SENESCENCE_TP53_TARGETS_DN	-0.15189036	-0.013483605	-3.424353399	0.000709193390106397	0.00196867771573811	-1.051964349	Down
TAKEDA_TARGETS_OF_NUP98_HOXA9_FUSION_8D_UP	-0.11676454	-0.002326701	-3.425145347	0.000707213480042247	0.00196493915426241	-1.049390346	Down
CAFFAREL_RESPONSE_TO_THC_24HR_5_DN	-0.092886979	-0.001139077	-3.42561102	0.000706051684756977	0.00196258970321834	-1.047876551	Down
LIANG_HEMATOPOIESIS_STEM_CELL_NUMBER_LARG	-0.076409724	-9.40E-05	-3.4300	0.000695	0.001932	-1.0	Down

E_VS_TINY_DN			60011	0415683 32209	8508130 0987	3340 4402	
WP_NANOPARTICLE_TRIGGERED_REGULATED_NECROSIS	-0.144062036	-0.001478613	-3.432152484	0.000689918893351985	0.00192204962793347	-1.026591828	Down
REACTOME_BIOSYNTHESIS_OF_EPA_DERIVED_SPMS	-0.197319878	0.00384818003757208	-3.432693172	0.000688600957724946	0.00191923940035866	-1.02483086	Down
REACTOME_MITOCHONDRIAL_IRON_SULFUR_CLUSTER_BIOGENESIS	-0.187062734	-0.006051728	-3.434909391	0.000683223440793467	0.00190596309977755	-1.017610199	Down
WP_FLUOROPYRIMIDINE_ACTIVITY	-0.108781149	-0.00916057	-3.436059479	0.000680448314465528	0.00189907494958972	-1.013861411	Down
LINDGREN_BLADDER_CANCER_WITH_LOH_IN_CHR9Q	-0.099692559	0.00701337983131869	-3.437610909	0.000676721453662384	0.00189037356565365	-1.008802602	Down
REACTOME_CYCLIN_D_ASSOCIATED_EVENTS_IN_G1	-0.10973958	-0.001327677	-3.439203089	0.000672916542872044	0.00188143828000305	-1.003608736	Down
REACTOME_SIGNALING_BY_EGFR_IN_CANCER	-0.096382064	0.00354623509496699	-3.440644519	0.000669489143291472	0.0018726990141551	-0.998904733	Down
REACTOME_G_BETA_GAMMA_SIGNALLING_THROUGH_CDC42	-0.124822567	-0.000210302	-3.44307616	0.000663744201386083	0.00185830413080894	-0.990965152	Down

LIM_MAMMARY_STEM_CELL_DN	-0.04785965	-0.000328973	-3.443471275	0.000662815075962979	0.00185654024210389	-0.989674572	Down
PAPASPYRIDONOS_UNSTABLE_ATEROSCLEROTIC_PLAQUE_UP	-0.131567633	0.0115567228849522	-3.443935754	0.000661724392759973	0.00185432203424883	-0.988157251	Down
CHESLER_BRAIN_HIGHEST_EXPRESSION	-0.120293944	-3.47E-05	-3.447511903	0.000653382946510141	0.00183260187482532	-0.976468702	Down
FERRARI_RESPONSE_TO_FENRETINIDE_UP	-0.123189247	0.000798351646128721	-3.448646241	0.000650757684002933	0.0018260637181764	-0.972758825	Down
TERAMOTO_OPN_TARGETS_CLUSTER_7	-0.113548132	-0.003584398	-3.450256231	0.000647048521109309	0.00181894482360756	-0.967491395	Down
HELLER_SILENCED_BY_METHYLATION_UP	-0.091272285	-0.000210214	-3.451518064	0.000644155285905203	0.00181163201613665	-0.963361461	Down
REACTOME_P38MAPK_EVENTS	-0.135326682	-0.001051214	-3.452294745	0.000642380467856122	0.00180745945783452	-0.960818726	Down
REACTOME_ACTIVATION_OF_ATR_IN_RESPONSE_TO_REPLICATION_STRESS	-0.168050021	-0.00167928	-3.454582277	0.000637179686362833	0.00179526750488157	-0.953326643	Down
SCHUETZ_BREAST_CANCER_DUCTAL_INVASIVE_UP	-0.08876573	0.0017773088496630	-3.459119515	0.0006269804285	0.0017681360834	-0.93845	Down

		3		01062	6483	291	
REACTOME_ADAPTIVE_IMMUNE_SYSTEM	-0.051876213	0.00197509317628261	-3.459825089	0.000625408157589107	0.00176450383370709	-0.936138329	Down
GAZDA_DIAMOND_BLACKFAN_ANEMIA_ERYTHROID_UP	-0.116861072	0.00751955624787535	-3.462358901	0.000619792266105552	0.00175104715326225	-0.927822784	Down
MATHEW_FANCONI_ANEMIA_GENES	-0.174447262	-0.003409487	-3.462764592	0.000618897493482583	0.00174931545630528	-0.926490856	Down
REACTOME_EUKARYOTIC_TRANSLATION_INITIATION	-0.229547564	-0.000209314	-3.463071163	0.000618222137622323	0.0017482026461147	-0.925484256	Down
MARKEY_RB1_ACUTE_LOF_UP	-0.132221643	-0.002589337	-3.467203047	0.000609186866386254	0.00172422383933401	-0.911909591	Down
GEORGES_CELL_CYCLE_MIR192_TARGETS	-0.127654894	0.00748013797425092	-3.468708842	0.000605924942433589	0.00171577377631628	-0.906958846	Down
KANG_CISPLATIN_RESISTANCE_DN	-0.169196147	0.00382317962825121	-3.470582728	0.000601888467529687	0.00170667963360291	-0.900795149	Down
GAUSSMANN_MLL_AF4_FUSION_TARGETS_B_DN	-0.180882691	-0.001329861	-3.477413505	0.000587386561459647	0.00167253286386763	-0.878830114	Down
BAELDE_DIABETIC_NEPHROPATHY_DN	-0.080355134	0.0070537	-3.4829	0.000575	0.001641	-0.8	Down

		1967769216	45681	881991658689	93822794005	60053803	
DUNNE_TARGETS_OF_AML1_MTG8_FUSION_UP	-0.147644047	0.0067024556448568	-3.483084682	0.000575595664267403	0.00164187605152012	-0.859594981	Down
GARGALOVIC_RESPONSE_TO_OXIDIZED_PHOSPHOLIPIDS_CYAN_UP	-0.188551909	0.00770001753390105	-3.484751001	0.000572173577067724	0.001632865008212	-0.854093389	Down
GRADE_COLON_AND_RECTAL_CANCER_UP	-0.117772916	0.0016747831785934	-3.485139263	0.000571378951444326	0.00163134735584863	-0.85281114	Down
NIKOLSKY_BREAST_CANCER_19Q13.1_AMPLICON	-0.227703646	-0.005617814	-3.4852161	0.000571221818397979	0.00163134735584863	-0.852557369	Down
REACTOME_MITOCHONDRIAL_FATTY_ACID_BETA_OXIDATION	-0.145600797	0.00673637303214771	-3.488323392	0.000564901115900554	0.00161508117291328	-0.842290513	Down
REACTOME_IRON_UPTAKE_AND_TRANSPORT	-0.09876381	-0.000524435	-3.490171269	0.000561173367532946	0.0016059027626911	-0.836180941	Down
KUMAMOTO_RESPONSE_TO_NUTLIN_3A_DN	-0.244123161	-0.022325288	-3.490486518	0.000560539716386799	0.00160482934483988	-0.835135835	Down
REACTOME_MITOTIC_SPINDLE_CHECKPOINT	-0.126093275	-0.002342026	-3.491004061	0.000559500905464459	0.00160259442557356	-0.833426543	Down

BOSCO_TH1_CYTOTOXIC_MODULE	-0.116560637	-0.002755554	-3.496151801	0.000549266061639762	0.00157545954001756	-0.816387428	Down
AUJLA_IL22_AND_IL17A_SIGNALING	-0.188115387	0.000617631473277333	-3.49626656	0.000549039906402511	0.00157545954001756	-0.816007313	Down
WAKABAYASHI_ADIPOGENESIS_PPARG_BOUND_36HR	-0.11793741	1.33072133147172e-07	-3.496525962	0.000548529024109518	0.0015748009494208	-0.815148053	Down
REACTOME_INTERFERON_SIGNALING	-0.126184518	-0.00661295	-3.501583318	0.000538657286193727	0.00154789156268725	-0.798384116	Down
WP_P38_MAPK_SIGNALING_PATHWAY	-0.115443257	0.00813498576759927	-3.501835336	0.000538169736707785	0.00154720683452766	-0.797548155	Down
ST_JNK_MAPK_PATHWAY	-0.121929985	0.00947126696335004	-3.506659182	0.000528916943195178	0.00152484322638759	-0.781536555	Down
GYORFFY_DOXORUBICIN_RESISTANCE	-0.087825081	-0.00119922	-3.507918853	0.00052652538237622	0.0015186538329039	-0.777352062	Down
DANG_BOUND_BY_MYC	-0.093771723	0.00400143331636898	-3.510883828	0.000520936130166772	0.00150363160339181	-0.767497308	Down
REACTOME_MYD88_INDEPENDENT_TLR4_CASCADE_	-0.095523604	0.0042150546729747	-3.52042595	0.0005033234130	0.0014578294094	-0.73573	Down

		3		03944	7993	0306	
BREDEMEYER_RAG_SIGNALING_VIA_ATM_NOT_VIA_NFKB_DN	-0.101081954	0.00359062102274658	-3.521855537	0.000500733286689348	0.00145158078506757	-0.730964237	Down
BOYLAN_MULTIPLE_MYELOMA_D_DN	-0.090892433	0.000150690355400581	-3.521970535	0.00050052547729303	0.00145158078506757	-0.730580771	Down
CASORELLI_ACUTE_PROMYELOCYTIC_LEUKEMIA_UP	-0.107820319	0.00864275753900088	-3.524409312	0.000496137422904819	0.00143970312481076	-0.722445861	Down
ZWANG_DOWN_BY_2ND_EGF_PULSE	-0.085998606	0.00122596527655219	-3.530094441	0.000486047887812606	0.00141505592854261	-0.703462291	Down
KORKOLA_EMBRYONAL_CARCINOMA	-0.178416669	-0.005284042	-3.538535562	0.000471421551799591	0.00137505337031018	-0.675224496	Down
WP_BIOMARKERS_FOR_PYRIMIDINE_METABOLISM_DISORDERS	-0.149333649	-0.00231709	-3.543762178	0.000462573164024808	0.00135051346618155	-0.657709228	Down
LIANG_HEMATOPOIESIS_STEM_CELL_NUMBER_SMALL_VS_HUGE_UP	-0.103033517	-0.003195491	-3.551247893	0.000450171271349032	0.0013174036215292	-0.632582263	Down
WP_G1_TO_S_CELL_CYCLE_CONTROL	-0.131352382	-0.000190873	-3.556392924	0.000441828938125511	0.00129482163311853	-0.615284131	Down
WP_ALLOGRAFT_REJECTION	-0.089005335	0.0006213	-3.5598	0.000436	0.001281	-0.6	Down

		90175089	45916	3117331 16483	6007072 6723	0366 201	
WP_VITAMIN_B12_DISORDERS	-0.158490154	-0.0040749 86	-3.5600 37704	0.000436 0071968 83152	0.001281 6007072 6723	-0.6 0301 6186	Down
LIU_VAV3_PROSTATE_CARCINOGENESIS_UP	-0.126142244	-0.0013728 01	-3.5606 92052	0.000434 9696716 42686	0.001279 5529629 7922	-0.6 0081 2506	Down
HAN_SATB1_TARGETS_UP	-0.045790435	0.0012304 816779607 9	-3.5608 37049	0.000434 7400799 59406	0.001279 4839622 1339	-0.6 0032 4142	Down
REACTOME_NEGATIVE_EPIGENETIC_REGULATION_OF _RRNA_EXPRESSION	-0.141266106	0.0032165 486675512 6	-3.5630 66647	0.000431 2239775 10131	0.001269 7377743 8585	-0.5 9281 2367	Down
EGUCHI_CELL_CYCLE_RB1_TARGETS	-0.198517252	-0.0151410 49	-3.5676 60022	0.000424 0641477 31404	0.001249 8414838 4085	-0.5 7732 3244	Down
NUYTTEN_NIPP1_TARGETS_DN	-0.066191572	0.0031876 673685292	-3.5689 79572	0.000422 0280538 68865	0.001244 4314158 499	-0.5 7287 0284	Down
STONER_ESOPHAGEAL_CARCINOGENESIS_DN	-0.217384707	0.0041803 800084391 8	-3.5740 33859	0.000414 3137773 6128	0.001224 5931505 1498	-0.5 5580 0185	Down
WP_TP53_NETWORK	-0.115596632	0.0003603 115159821 53	-3.5754 79206	0.000412 1322332 5605	0.001219 3063736 0358	-0.5 5091 4695	Down

REACTOME_TOLL_LIKE_RECEPTOR_CASCADES	-0.104271448	0.00237030233949734	-3.576474902	0.000410635654178178	0.0012160379320057	-0.547548049	Down
REACTOME_IRAK4_DEFICIENCY_TLR2_4_	-0.249484862	0.00696071494654894	-3.578899431	0.000407012783593426	0.00120588465287083	-0.53934666	Down
KYNG_NORMAL_AGING_UP	-0.128006776	0.00500041093285839	-3.57890638	0.000407002443274555	0.00120588465287083	-0.539323146	Down
STAMBOLSKY_TARGETS_OF_MUTATED_TP53_DN	-0.171349131	-0.008340999	-3.582379459	0.000401865159426492	0.00119291106865626	-0.527566015	Down
REACTOME_TRANSCRIPTIONAL_REGULATION_BY_E2F6	-0.136454081	0.00651806731743725	-3.586263939	0.000396191520843929	0.00117663194731018	-0.514403904	Down
SHAFFER_IRF4_TARGETS_IN_PLASMA_CELL_VS_MATURE_B_LYMPHOCYTE	-0.108402236	-0.00495239	-3.590554909	0.000390011672527679	0.00115938814721231	-0.499849357	Down
ROSS_AML_WITH_CBFB_MYH11_FUSION	-0.15742451	0.00607568816578221	-3.593649394	0.000385611328283758	0.00114685649959621	-0.489343337	Down
MCBRYAN_PUBERTAL_BREAST_3_4WK_DN	-0.088044072	-0.00152953	-3.594485944	0.000384429792784226	0.00114389056750321	-0.486501771	Down
REACTOME_DEFECTS_IN_COBALAMIN_B12_METABOLISM	-0.163616267	-0.000368211	-3.597686299	0.0003799409665	0.0011321620638	-0.47562	Down

				03463	9198	535	
REACTOME_METABOLISM_OF_WATER_SOLUBLE_VITAMINS_AND_COFACTORS	-0.061250707	0.0019538061127563	-3.598404217	0.00037894079939653	0.00112972408350349	-0.473184293	Down
ZHONG_RESPONSE_TO_AZACITIDINE_AND_TSA_DN	-0.139603226	0.00192328961429595	-3.601548631	0.00037458924063162	0.00111782471951945	-0.462487475	Down
BREDEMEYER_RAG_SIGNALING_NOT_VIA_ATM_DN	-0.071842659	0.00507024335579706	-3.607343255	0.000366692809684288	0.00109584124685141	-0.442752784	Down
ZHENG_BOUND_BY_FOXP3	-0.133899491	0.00540543446183639	-3.608642858	0.000364943431565801	0.00109113867038966	-0.43832278	Down
BASSO_B_LYMPHOCYTE_NETWORK	-0.14474034	0.000215812898780542	-3.612204621	0.000360189117220702	0.00107796231947391	-0.426174228	Down
GOTTWEIN_TARGETS_OF_KSHV_MIR_K12_11	-0.110769549	0.0100993139106936	-3.613655313	0.000358269443095315	0.00107377036856235	-0.421223049	Down
REACTOME_DETOXIFICATION_OF_REACTIVE_OXYGEN_SPECIES	-0.121912398	-0.002251212	-3.61680635	0.000354132824045755	0.00106394116110939	-0.410462411	Down
PARK_HSC_AND_MULTIPOTENT_PROGENITORS	-0.112157525	0.00712593309174475	-3.618694206	0.000351676039470421	0.00105838810212225	-0.404011386	Down
KAUFFMANN_DNA_REPAIR_GENES	-0.128630896	0.0008898	-3.6228	0.000346	0.001043	-0.3	Down

		972970836 47	8529	2790913 87348	3758836 1227	8967 9039	
REACTOME_ACTIVATION_OF_THE_PRE_REPLICATIVE_COMPLEX	-0.198502378	-0.0048154 01	-3.6263 89984	0.000341 8259216 33824	0.001030 4582300 0541	-0.3 7768 238	Down
REACTOME_PROCESSING_OF_CAPPED_INTRONLESS_PRE_MRNA	-0.15136081	0.0065066 939474238 3	-3.6276 64722	0.000340 2196026 09439	0.001027 6122011 6632	-0.3 7331 6307	Down
REACTOME_NRIF_SIGNALS_CELL_DEATH_FROM_THE_NUCLEUS	-0.139674809	0.0088644 833462542 1	-3.6279 08171	0.000339 9136376 10382	0.001027 1879009 969	-0.3 7248 2319	Down
VISALA_RESPONSE_TO_HEAT_SHOCK_AND_AGING_DN	-0.17660995	0.0055386 690899231 8	-3.6282 4042	0.000339 4964851 50757	0.001026 9272335 9198	-0.3 7134 404	Down
NIKOLSKY_BREAST_CANCER_15Q26_AMPLICON	-0.146083814	0.0004909 207548034 29	-3.6292 80509	0.000338 1937244 35471	0.001023 9099668 6498	-0.3 6778 0112	Down
MORI_PRE_BI_LYMPHOCYTE_DN	-0.130809645	0.0096049 439313272	-3.6318 85939	0.000334 9509135 56692	0.001015 6523304 5744	-0.3 5884 8374	Down
REACTOME_TRIF_MEDIATED_PROGRAMMED_CELL_DEATH	-0.228025132	0.0008777 400517935 4	-3.6337 49155	0.000332 6498504 74056	0.001009 6614287 9827	-0.3 5245 7464	Down
PUJANA_BREAST_CANCER_WITH_BRCA1_MUTATED_UP	-0.20021623	-0.0024392 57	-3.6384 33462	0.000326 9302782 70954	0.000996 6877393 06392	-0.3 3637 6956	Down

BLANCO_MELO_COVID19_SARS_COV_2_INFECTION_CALU3_CELLS_UP	-0.142337673	-0.010954234	-3.639020028	0.000326220632262802	0.000995013004646296	-0.334362042	Down
REACTOME_GLUTATHIONE_CONJUGATION	-0.125903783	-0.006128184	-3.643395849	0.000320972175531681	0.000979967680041881	-0.31932133	Down
DEMAGALHAES_AGING_DN	-0.140599587	-0.006616489	-3.644743502	0.000319371843404906	0.0009760418670 6758	-0.31468583	Down
UZONYI_RESPONSE_TO_LEUKOTRIENE_AND_THROMBIN	-0.159468614	0.00857155434059476	-3.645058538	0.000318998823879171	0.000975382118136952	-0.313601982	Down
CREIGHTON_ENDOCRINE_THERAPY_RESISTANCE_2	-0.048078851	0.000290528050814279	-3.645400306	0.000318594616831538	0.000974626311815355	-0.312426071	Down
XU_HGF_SIGNALING_NOT_VIA_AKT1_48HR_DN	-0.162434198	0.00628341822783217	-3.646977177	0.000316735890695464	0.000969896237566229	-0.30699927	Down
GRAHAM_CML_DIVIDING_VS_NORMAL_DIVIDING_UP	-0.210219055	-0.010647849	-3.646982495	0.000316729640281635	0.000969896237566229	-0.306980967	Down
RODRIGUES_DCC_TARGETS_UP	-0.190816386	-0.000155992	-3.652927232	0.00030981381755941	0.000951987309698644	-0.286502993	Down
MARSON_FOXP3_TARGETS_STIMULATED_UP	-0.15828784	0.0034790780048534	-3.655073273	0.0003073523245	0.0009453597020	-0.27910	Down

		8		85395	32482	3043	
KERLEY_RESPONSE_TO_CISPLATIN_UP	-0.084147449	0.000472852876461186	-3.657975325	0.000304052965403535	0.000936139264017729	-0.26908996	Down
WP_CHOLESTEROL_METABOLISM_INCLUDES_BOTH_BLOCH_AND_KANDUTSCHRUSSELL_PATHWAYS	-0.096276812	0.00113551863755525	-3.658369857	0.000303607002905648	0.000935230107709855	-0.267728131	Down
REACTOME_FIBRONECTIN_MATRIX_FORMATION	-0.218195111	0.000126120672470935	-3.659963774	0.000301811568474392	0.000930623152270517	-0.262224962	Down
REACTOME_SIGNALING_BY_ERBB2_IN_CANCER	-0.103237476	-0.002367683	-3.660858951	0.000300807603447228	0.000928449922723493	-0.259133313	Down
TURJANSKI_MAPK14_TARGETS	-0.166497127	0.00846871348104943	-3.663941444	0.000297374510959346	0.000918767341724571	-0.24848215	Down
BIOCARTA_CTCF_PATHWAY	-0.149336841	0.0119749396225761	-3.665452625	0.000295704958746439	0.000914519521145564	-0.243257486	Down
BLANCO_MELO_RESPIRATORY_SYNCYTIAL_VIRUS_INFECTION_A594_CELLS_UP	-0.141378161	-0.003843201	-3.674603392	0.000285782259964105	0.000886039204594006	-0.21157849	Down
ZHANG_RESPONSE_TO_IKK_INHIBITOR_AND_TNF_UP	-0.110886959	0.00119355228540842	-3.679870301	0.000280214141317559	0.000871387362303651	-0.193312553	Down
HOWLIN_CITED1_TARGETS_2_DN	-0.172081159	0.0037386	-3.6835	0.000276	0.000860	-0.1	Down

		7711692449	32982	402420214055	396099432618	80596199	
REICHERT_MITOSIS_LIN9_TARGETS	-0.203647848	-0.014174501	-3.689183053	0.000270618117750947	0.0008432362735342	-0.160957426	Down
RASHI_RESPONSE_TO_IONIZING_RADIATION_1	-0.103994488	0.00581279239106502	-3.691332592	0.00026844761543939	0.000837599981402859	-0.153478802	Down
KANG_DOXORUBICIN_RESISTANCE_UP	-0.201870941	-0.018011455	-3.691575374	0.000268203500282462	0.00083739392668674	-0.152633875	Down
GUILLAUMOND_KLF10_TARGETS_DN	-0.137021234	0.0122189296164515	-3.692238716	0.000267537580342487	0.000835735159127237	-0.15032506	Down
VECCHI_GASTRIC_CANCER_EARLY_UP	-0.101456809	-0.004574533	-3.696451488	0.000263344671789184	0.000823466185287389	-0.135653395	Down
REACTOME_GLOBAL_GENOME_NUCLEOTIDE_EXCISION_REPAIR_GG_NER_	-0.154501935	0.00173052044320133	-3.70033125	0.000259538061345769	0.00081320179039535	-0.122128103	Down
ZHAN_MULTIPLE_MYELOMA_HP_DN	-0.112134136	0.00597584848214836	-3.700899143	0.000258985247838665	0.000811879511785149	-0.120147289	Down
BUYTAERT_PHOTODYNAMIC_THERAPY_STRESS_UP	-0.112217813	0.00890243644399514	-3.704794662	0.000255222979526542	0.000800894354864127	-0.106552263	Down

BROWNE_HCMV_INFECTION_20HR_DN	-0.073676964	0.00263281685757655	-3.709562204	0.0002506886309 7189	0.000788659063579584	-0.089896352	Down
REACTOME_PROTEIN_LOCALIZATION	-0.14096179	-0.001676726	-3.710978997	0.000249355847668726	0.000784863968803135	-0.084942904	Down
PID_TOLL_ENDOGENOUS_PATHWAY	-0.178355751	0.00641288768661937	-3.713191681	0.000247287724042295	0.000778749316656787	-0.077203406	Down
KYNG_DNA_DAMAGE_BY_GAMMA_AND_UV_RADIATION	-0.082798202	0.00650597969826689	-3.715091172	0.000245525245521896	0.000773984357010874	-0.070556064	Down
IKEDA_MIR30_TARGETS_UP	-0.159389376	0.0114258674311197	-3.715591319	0.000245063148689171	0.000772920205240693	-0.068805268	Down
TSAI_DNAJB4_TARGETS_UP	-0.244709309	-0.005998769	-3.717772942	0.000243057076359991	0.000767372977093828	-0.061165877	Down
SCHOEN_NFKB_SIGNALING	-0.159833496	0.00647912968424464	-3.72079319	0.000240305411396393	0.000759071597220057	-0.050583175	Down
SHETH_LIVER_CANCER_VS_TXNIP_LOSS_PAM6	-0.089523436	0.0053223960286888	-3.721467673	0.000239694935738256	0.000757528750574008	-0.048218784	Down
REACTOME_CROSS_PRESENTATION_OF_PARTICULATE_EXOGENOUS_ANTIGENS_PHAGOSOMES_	-0.243462338	0.0069077295471843	-3.724798693	0.0002367014147	0.0007488306224	-0.03653	Down

		9		28444	36009	6247	
NEMETH_INFLAMMATORY_RESPONSE_LPS_UP	-0.14958935	0.0060705288637859 8	-3.7255 54056	0.000236 0275074 49901	0.000747 0794180 22202	-0.0 3388 5726	Down
REACTOME_OAS_ANTIVIRAL_RESPONSE	-0.248970386	-0.0090547 69	-3.7258 42645	0.000235 7705176 94457	0.000746 6467363 926	-0.0 3287 2959	Down
YAO_TEMPORAL_RESPONSE_TO_PROGESTERONE_CLUSTER_3	-0.172120259	0.0022636550944775 4	-3.7265 13482	0.000235 1741531 4981	0.000745 5188807 97177	-0.0 3051 8464	Down
BROWNE_HCMV_INFECTION_10HR_DN	-0.114120348	0.0047091498069257 2	-3.7276 81312	0.000234 1393628 6027	0.000742 6177952 34387	-0.0 2641 8716	Down
KAMIKUBO_MYELOID_MN1_NETWORK	-0.172452935	0.0078698398872445 6	-3.7297 25593	0.000232 3383013 03807	0.000737 6592512 49478	-0.0 1923 9335	Down
IIZUKA_LIVER_CANCER_PROGRESSION_L1_G1_UP	-0.139004463	0.0040862726851975 5	-3.7344 64387	0.000228 2134496 15695	0.000725 3051109 90588	-0.0 0258 3324	Down
REACTOME_MATURATION_OF_NUCLEOPROTEIN	-0.172039862	0.0061092288797052 5	-3.7382 63872	0.000224 9562299 16317	0.000716 4203792 15278	0.01 0784 9767 5519 42	Down
REACTOME_SYNTHESIS_OF_PIPS_AT_THE_LATE_ENDOSOME_MEMBRANE	-0.190464477	0.0071865611028241	-3.7395 39503	0.000223 8725307	0.000713 7014886	0.01 5275	Down

		1		48241	25748	9753 1662 47	
SEMBA_FHIT_TARGETS_DN	-0.21362983	-0.0104724 42	-3.7427 78167	0.000221 1432494 33458	0.000706 0885541 32446	0.02 6684 2585 0595 21	Down
ODONNELL_TFRC_TARGETS_DN	-0.125495419	-0.0055838 07	-3.7435 95286	0.000220 4596267 45946	0.000704 2680922 34732	0.02 9563 9897 0266 52	Down
ODONNELL_TARGETS_OF_MYC_AND_TFRC_DN	-0.169731177	-0.0089743 74	-3.7481 26511	0.000216 7047438 13247	0.000692 9862673 10059	0.04 5543 4592 4263 45	Down
BURTON_ADIPOGENESIS_5	-0.14141035	0.0008034 227415269 45	-3.7483 45632	0.000216 5247039 86269	0.000692 7674420 83904	0.04 6316 6380 5946 93	Down
BOYLAN_MULTIPLE_MYELOMA_C_D_DN	-0.086903703	0.0006258 397584892	-3.7483 87074	0.000216 4906697 8934	0.000692 7674420 83904	0.04 6462 8702	Down

						886918	
FOURNIER_ACINAR_DEVELOPMENT_LATE_DN	-0.169358987	-0.011028165	-3.749576471	0.000215516014408523	0.000690251755125748	0.0506604585568384	Down
LEE_LIVER_CANCER_E2F1_DN	-0.091741953	0.00214933479752938	-3.750407374	0.000214837586816691	0.000688434125643366	0.0535935751618686	Down
BIOCARTA_PTC1_PATHWAY	-0.172311635	-0.005993574	-3.751972187	0.00021356539746338	0.000684710961805372	0.0591190063739786	Down
FUJII_YBX1_TARGETS_DN	-0.130838558	-0.000894066	-3.753221714	0.000212554644861625	0.000682175119263756	0.0635326435795998	Down
FARMER_BREAST_CANCER_CLUSTER_1	-0.240138753	-0.008574147	-3.754943328	0.000211169414781453	0.000678079957345307	0.06961597491873	Down

						93	
BIOCARTA_SM_PATHWAY	-0.254168564	0.00307661616978418	-3.757934019	0.000208783313275826	0.000671112390213907	0.080189582350183 9	Down
BRUNO_HEMATOPOIESIS	-0.092645471	0.00448510220287206	-3.760195205	0.000206996167657626	0.000666057653007197	0.088189060922166 7	Down
BIOCARTA_P38MAPK_PATHWAY	-0.131367361	0.00804860206125849	-3.762643576	0.000205077372973831	0.000661254677427828	0.096855641565007 9	Down
FLECHNER_PBL_KIDNEY_TRANSPLANT_OK_VS_DONOR_DN	-0.146155952	0.00443079838897028	-3.762865519	0.000204904268183723	0.000661039913002272	0.097641513625783 1	Down
REACTOME_GDP_FUCOSE_BIOSYNTHESIS	-0.222704401	0.00381334748506516	-3.764050696	0.000203982222986023	0.000658407518499348	0.101838773925849	Down

ABRAHAM_ALPC_VS_MULTIPLE_MYELOMA_DN	-0.121685837	0.00490310111761724	-3.766366098	0.000202192178006565	0.000653309135287219	0.110042123802781	Down
PETRETTO_LEFT_VENTRICLE_MASS_QTL_CIS_UP	-0.247486155	0.00711315210816429	-3.767167012	0.000201576450246501	0.000651658868062517	0.112880777898484	Down
BLANCO_MELO_BRONCHIAL_EPITHELIAL_CELLS_INFLUENZA_A_DEL_NS1_INFECTION_UP	-0.12866886	-0.008840053	-3.76794177	0.000200982515948708	0.000650338856654285	0.115627247301169	Down
BENPORATH_MYC_MAX_TARGETS	-0.11937977	0.00340974545215462	-3.770827722	0.000198784641051129	0.000644647997389947	0.125862250923292	Down
BLANCO_MELO_HUMAN_PARAINFLUENZA_VIRUS_3_INFECTION_A594_CELLS_UP	-0.196865915	-0.018173013	-3.771074829	0.000198597509253969	0.000644377804463871	0.126738940873723	Down
DER_IFN_GAMMA_RESPONSE_UP	-0.161390611	-0.0062094	-3.7764	0.000194	0.000632	0.14	Down

		49	36351	578017326181	990436867718	5773399327386	
HOUSTIS_ROS	-0.125395426	0.00132766702059572	-3.776487515	0.000194540033031231	0.000632990436867718	0.14595515840543 9	Down
REACTOME_DEPOSITION_OF_NEW_CENPA_CONTAINING_NUCLEOSOMES_AT_THE_CENTROMERE	-0.203883922	-0.004330728	-3.776598984	0.000194457302226485	0.000632990436867718	0.14635115826168 7	Down
REACTOME_PROPIONYL_COA_CATABOLISM	-0.279419472	0.00413721363442826	-3.780924916	0.000191272205372963	0.0006241990424553	0.16172742169816 7	Down
MELLMAN_TUT1_TARGETS_UP	-0.120716348	0.00864328953994121	-3.781250097	0.00019103478356683	0.000623752183902848	0.16288390027760 3	Down
CHUANG_OXIDATIVE_STRESS_RESPONSE_UP	-0.11790309	-0.005506769	-3.781610898	0.0001907716812	0.0006232209608	0.164167	Down

				77876	90408	160447214	
IZADPANAH_STEM_CELL_ADIPOSE_VS_BONE_UP	-0.083390766	0.00067449042603106	-3.784086255	0.00018897581863 5343	0.00061767925553 9532	0.172974237169348	Down
BONOME_OVARIAN_CANCER_SURVIVAL_OPTIMAL_DEBULKING	-0.041562305	0.00019695899861651	-3.789088325	0.00018539547965 8005	0.00060725580065 2895	0.190786950029875	Down
BROWNE_HCMV_INFECTION_14HR_UP	-0.088580849	0.00647972440496915	-3.789573688	0.00018505150382 6339	0.00060644914691 1346	0.192516487919312	Down
KEGG_PANTOTHENATE_AND_COA_BIOSYNTHESIS	-0.156011924	0.00289236921510136	-3.793468463	0.00018231304114 2283	0.00059810626129 5006	0.206402319872984	Down
KEGG_SYSTEMIC_LUPUS_ERYTHEMATOSUS	-0.112183862	-0.004624813	-3.794862718	0.00018134206885 0628	0.00059523544228 2308	0.2113762996	Down

						4693	
NAM_FXYD5_TARGETS_DN	-0.182048108	0.00243310543933655	-3.794987809	0.000181255192988833	0.000595235442282308	0.211822643452527	Down
NIKOLSKY_BREAST_CANCER_12Q13_Q21_AMPLICON	-0.127535033	0.00956208036111219	-3.802688294	0.000175982345957807	0.000579788970997933	0.239324441644493	Down
WP_PENTOSE_PHOSPHATE_METABOLISM	-0.234977123	0.0031520635987133	-3.804033869	0.000175075989501191	0.000577492969837646	0.244135225713342	Down
ONGUSAHA_BRCA1_TARGETS_UP	-0.148534627	-0.002086921	-3.804275923	0.000174913414915617	0.000577492969837646	0.245000796021222	Down
WALLACE_PROSTATE_CANCER_RACE_DN	-0.103937661	0.00443973703024393	-3.806203068	0.000173624140450963	0.000573847199030419	0.251893905589729	Down
VANASSE_BCL2_TARGETS_UP	-0.13557132	-0.0044061	-3.8105	0.000170	0.000564	0.26	Down

		76	70773	73529462662	599879460539	752815997034	
REACTOME_METAL_SEQUESTRATION_BY_ANTIMICROBIAL_PROTEINS	-0.255999893	-0.013087694	-3.810610994	0.000170708904092616	0.000564599879460539	0.267672208575839	Down
PELLICCIOTTA_HDAC_IN_ANTIGEN_PRESENTATION_UP	-0.148398634	0.000822112192449853	-3.813052656	0.000169114065126365	0.000560716806110403	0.276419277318324	Down
WHITEFORD_PEDIATRIC_CANCER_MARKERS	-0.171877296	-0.013461711	-3.813913583	0.000168555094890555	0.000559476724056511	0.279504683671893	Down
PHONG_TNF_TARGETS_DN	-0.213487252	0.000526141450908639	-3.816651516	0.000166789041289361	0.000553910957347814	0.289321101329656	Down
AZARE_NEOPLASTIC_TRANSFORMATION_BY_STAT3_UP	-0.09348627	0.00447007961626042	-3.817203879	0.000166434877681609	0.000553030666900293	0.2913022775	Down

						37223	
REACTOME_FORMATION_OF_RNA_POL_II_ELONGATION_COMPLEX_	-0.152800535	0.00388637556055944	-3.817852322	0.00016602001719695	0.000551947641532654	0.293628395775513	Down
ZHAN_MULTIPLE_MYELOMA_PR_UP	-0.222284273	-0.019280624	-3.820644079	0.00016424505286261	0.00054663219470146	0.30364714633581	Down
PIEPOLI_LGI1_TARGETS_UP	-0.149018697	-0.000987455	-3.824368813	0.000161904851527604	0.00053942212207828	0.317024307633932	Down
REACTOME_SIGNALING_BY_FGFR	-0.077504032	0.00167641255369561	-3.824978029	0.000161525103931132	0.000538445929162479	0.319213383745261	Down
KEGG_PROTEIN_EXPORT	-0.226268006	-0.003995005	-3.826147486	0.000160798495679361	0.00053631180154852	0.323416440054554	Down

SUZUKI_RESPONSE_TO_TSA	-0.135415672	0.00100662784669498	-3.82758723	0.000159908200149281	0.000534203551306022	0.328592493493412	Down
VANTVEER_BREAST_CANCER_POOR_PROGNOSIS	-0.106834008	-0.002517845	-3.82960568	0.000158667898715757	0.00053063127550038	0.335852012890832	Down
LIANG_SILENCED_BY_METHYLATION_DN	-0.240848437	-0.021476008	-3.83074433	0.000157972239883827	0.000528874699546339	0.33994877767632	Down
ITO_PTTG1_TARGETS_UP	-0.147908658	0.00515960925054015	-3.83178373	0.000157339738367834	0.000527326002186365	0.343689404154068	Down
REACTOME_G2_M_DNA_REPLICATION_CHECKPOINT	-0.283621341	-0.012686054	-3.834029151	0.000155981512211443	0.000523056318906769	0.351773411521134	Down
WP_PHYTOCHEMICAL_ACTIVITY_ON_NRF2_TRANSCRIPTIONAL_ACTIVATION	-0.168695862	0.0105051580818385	-3.835205805	0.0001552742075	0.0005212477049	0.356011	Down

				72058	21451	31940591 5	
REACTOME_NUCLEOTIDE_EXCISION_REPAIR	-0.152819012	0.0018215520309768	-3.83594859	0.00015482927169105	0.000520222903609642	0.358687182047008	Down
SMIRNOV_RESPONSE_TO_IR_6HR_DN	-0.096069415	0.00580027882412774	-3.839519423	0.00015270709736437 8	0.00051346313832107 1	0.371557496971541	Down
MILI_PSEUDOPODIA_CHEMOTAXIS_UP	-0.139594351	0.00809273836725559	-3.839989843	0.000152429584172273	0.00051280782057306	0.373253827241996	Down
VALK_AML_WITH_EVI1	-0.107625811	-0.003163249	-3.841714108	0.000151416469185478	0.00051078370882297	0.379473112909194	Down
REACTOME_GOLGI_ASSOCIATED_VESICLE_BIOGENESIS	-0.103679921	0.00962488652995202	-3.84396482	0.000150103620322024	0.00050663032699228	0.3875950181	Down

						36015	
REACTOME_TRISTETRAPROLIN_TTP_ZFP36_BINDS_AND_DESTABILIZES_MRNA	-0.173285507	0.00510726640108098	-3.85003703	0.000146615213442987	0.000495934948142026	0.409528452983242	Down
ZHANG_INTERFERON_RESPONSE	-0.291676533	-0.033458344	-3.852781521	0.000145063848427488	0.000491490888203832	0.419452026198255	Down
WP_PEROXIREDOXIN_2_INDUCED_OVARIAN_FAILURE	-0.174780709	0.00659099062569259	-3.857802299	0.000142265917436438	0.000482801831343887	0.437622648820732	Down
MEBARKI_HCC_PROGENITOR_FZD8CRD_UP	-0.086241238	-0.003809968	-3.860960901	0.000140531989688675	0.000477439551175483	0.449064772938629	Down
WP_CHROMOSOMAL_AND_MICROSATELLITE_INSTABILITY_IN_COLORECTAL_CANCER	-0.100933639	0.0118264496435191	-3.864222757	0.000138762395763343	0.000472461980528287	0.46088974710555	Down

						9	
BENPORATH_NANOG_TARGETS	-0.077084245	0.00383449819290135	-3.865440014	0.000138107442946199	0.000470490064965454	0.4653048783497768	Down
AMIT_EGF_RESPONSE_40_MCF10A	-0.197734738	0.00404989452814522	-3.867331042	0.000137095767557021	0.000468071193193856	0.4721663256874 63	Down
HOWLIN_PUBERTAL_MAMMARY_GLAND	-0.11436522	0.00310505778822585	-3.871580711	0.000134847800854406	0.000460903248845429	0.4875968922583 14	Down
REACTOME_MET_ACTIVATES_PTPN11	-0.239336879	0.00458560246218777	-3.875046199	0.000133040536835554	0.000454976645806218	0.5001913338241 84	Down
BLANCO_MELO_COVID19_SARS_COV_2_INFECTION_A594_ACE2_EXPRESSING_CELLS_RUXOLITINIB_UP	-0.088932334	0.00461249287703403	-3.875878946	0.000132609688393574	0.000453753217121784	0.5032192476543 57	Down

TAKADA_GASTRIC_CANCER_COPY_NUMBER_DN	-0.125297596	0.0024189178342073 9	-3.879808012	0.0001305946297 1357	0.0004475984906 85881	0.51751341288387 5	Down
ZHENG_IL22_SIGNALING_UP	-0.140826123	-0.0015154 9	-3.880055662	0.0001304685972 49989	0.0004475984906 85881	0.51841480942131 1	Down
KEGG_CARDIAC_MUSCLE_CONTRACTION	-0.104844226	-0.0010293 92	-3.884673174	0.0001281396753 91337	0.0004401565938 87121	0.53523111936416 7	Down
PID_P38_MKK3_6PATHWAY	-0.146754337	0.0043383877252251 4	-3.886558656	0.0001272000727 38659	0.0004371710141 13431	0.54210292225292 2	Down
REACTOME_PEROXISOMAL_PROTEIN_IMPORT	-0.110624133	0.0011211945682007 6	-3.886641012	0.0001271591808 60445	0.0004371710141 13431	0.54240314490991 4	Down
UROSEVIC_RESPONSE_TO_IMIQUIMOD	-0.295430387	-0.0313595	-3.8881	0.000126	0.000434	0.54	Down

		92	22829	425548060138	990785370996	7805953126505	
FARMER_BREAST_CANCER_BASAL_VS_LULMINAL	-0.070563346	0.0017272667430862	-3.889227133	0.000125881428672093	0.000433599349482619	0.551833520113302	Down
YANAGIHARA_ESX1_TARGETS	-0.148103785	0.00554033585941577	-3.889267056	0.000125861799455699	0.000433599349482619	0.551979142692608	Down
XU_GH1_EXOGENOUS_TARGETS_DN	-0.072451938	-0.001741002	-3.891377038	0.000124828473665472	0.000431146064310782	0.559677495396922	Down
KIM_MYC_AMPLIFICATION_TARGETS_DN	-0.08795612	-0.000812894	-3.893851816	0.000123626741367928	0.000427493695638289	0.568711579735943	Down
WHITFIELD_CELL_CYCLE_LITERATURE	-0.193685588	-0.013249421	-3.894479934	0.0001233234818	0.0004266827490	0.571005	Down

				44299	56613	316999428	
PHESSE_TARGETS_OF_APC_AND_MBD2_DN	-0.220463217	0.0101510936344634	-3.894666962	0.000123233319512971	0.000426608596886229	0.57168836485152 8	Down
BOHN_PRIMARY_IMMUNODEFICIENCY_SYNDROM_UP	-0.152626157	-0.002234571	-3.895464375	0.000122849604751721	0.000425517576280095	0.57460093117873 5	Down
LEE_AGING_CEREBELLUM_UP	-0.116695766	-0.000453475	-3.89805755	0.000121609584161845	0.000421693122286354	0.58407624402052 2	Down
CHICAS_RB1_TARGETS_GROWING	-0.099881901	-0.001416284	-3.9075533	0.000117169360658098	0.000407434297817824	0.61882131689597	Down
REACTOME_ACTIVATED_TAK1_MEDIATES_P38_MAPK_ACTIVATION	-0.138314044	0.00229006695856709	-3.907977983	0.000116974413840094	0.00040721266781013	0.62037700608665	Down

						4	
CASTELLANO_NRAS_TARGETS_UP	-0.098589724	-0.003754947	-3.90993737	0.000116078952264429	0.000404549161541442	0.627556536822244	Down
WP_AUTOPHAGY	-0.128894433	0.00488983528699557	-3.912991445	0.000114696155756273	0.000400465839739329	0.638753608862982	Down
SENESE_HDAC1_TARGETS_UP	-0.109034487	0.00761033643936486	-3.915859174	0.000113411962973802	0.000396590453058248	0.649274598541203	Down
WP_MITOCHONDRIAL_CII_ASSEMBLY	-0.251007952	0.00972016355644716	-3.92106416	0.000111115885386428	0.000389438904908843	0.668388013533249	Down
BARRIER_CANCER_RELAPSE_NORMAL_SAMPLE_UP	-0.134872716	0.00614752511080367	-3.921287557	0.0001110183317371	0.000389438904908843	0.669208865048367	Down

GOTZMANN_EPITHELIAL_TO_MESENCHYMAL_TRANSITION_DN	-0.067316335	0.00383465598442291	-3.923116922	0.000110222529777485	0.000386963372357948	0.675932270067925	Down
REACTOME_CLASS_I_PEROXISOMAL_MEMBRANE_PROTEIN_IMPORT	-0.204751836	0.00210448390994757	-3.923147287	0.000110209366210727	0.000386963372357948	0.676043893929213	Down
FOSTER_TOLERANT_MACROPHAGE_DN	-0.087370956	0.00280114739969463	-3.924285526	0.000109717004133544	0.000386062043456296	0.680228670649548	Down
MARSON_BOUND_BY_E2F4_UNSTIMULATED	-0.135643721	-0.002314713	-3.924386079	0.000109673608995849	0.000386062043456296	0.680598408636494	Down
WP_CONTROL_OF_IMMUNE_TOLERANCE_BY_VASOACTIVE_INTESTINAL_PEPTIDE	-0.157575732	0.003399980413978	-3.925073056	0.000109377568165289	0.000385304520772955	0.683124689435743	Down
DELPUECH_FOXO3_TARGETS_DN	-0.166167135	-0.0069511	-3.9254	0.000109	0.000385	0.68	Down

		01	16318	229929691578	002937873721	4387143290163	
WHITFIELD_CELL_CYCLE_M_G1	-0.107733625	0.00287797232453096	-3.928346492	0.000107977320355844	0.000380804106504957	0.69516779297596	Down
SCIBETTA_KDM5B_TARGETS_UP	-0.158873566	0.0012973167398962	-3.928666254	0.000107841454178129	0.000380541163208442	0.696344690581152	Down
HOSHIDA_LIVER_CANCER_LATE_RECURRENCE_UP	-0.091572013	0.00232951316122178	-3.929788836	0.0001073657539216	0.000379078062907491	0.700477083528134	Down
DURCHDEWALD_SKIN_CARCINOGENESIS_UP	-0.076492832	0.00125255280866165	-3.936821727	0.000104430528869406	0.000369344896121029	0.726390191055372	Down
BROWNE_HCMV_INFECTION_18HR_UP	-0.097338207	0.00598395542201052	-3.939430071	0.000103361401087368	0.000365980728208381	0.7360113103	Down

						36399	
GALLUZZI_PERMEABILIZE_MITOCHONDRIA	-0.095398113	0.00859706737181555	-3.941909695	0.0001023546707 4256	0.000362830063563148	0.745162903678724	Down
PUJANA_BREAST_CANCER_WITH_BRCA1_MUTATED_DN	-0.267078071	0.0152767230012961	-3.942711256	0.000102031230754852	0.000361890199597351	0.748122341012097	Down
BIOCARTA_D4GDI_PATHWAY	-0.191087202	0.0142832488788823	-3.944601431	0.000101272357822346	0.000359609568079693	0.755103162742681	Down
PLASARI_NFIC_TARGETS_BASAL_DN	-0.13341626	-0.000375942	-3.945235733	0.000101018898876158	0.000358914885703672	0.757446446290592	Down
MOOTHA_MITOCHONDRIA	-0.147136198	-0.000870073	-3.950146715	9.9076808284117 6e-05	0.000352822575455834	0.77560031813495	Down

						8	
BOWIE_RESPONSE_TO_TAMOXIFEN	-0.310700721	-0.028079927	-3.950755328	9.8838609911266 3e-05	0.00035217637871368	0.77785151206499 7	Down
VANHARANTA_UTERINE_FIBROID_WITH_7Q_DELETION_DN	-0.10490475	0.00060768316097824	-3.951164612	9.8678730492615 3e-05	0.000351808661784987	0.77936558368796 1	Down
PAL_PRMT5_TARGETS_UP	-0.142552905	0.00188448765905704	-3.952314135	9.8231001768605 8e-05	0.000350451470789531	0.78361878432443 8	Down
HOOI_ST7_TARGETS_DN	-0.081739322	0.00125086858086275	-3.953971092	9.7589024107334 3e-05	0.00034892573308423	0.78975141401270 4	Down
REACTOME_TRAFFICKING_OF_MYRISTOYLATED_PROTEINS_TO_THE_CILIUM	-0.255840647	0.00641362312993915	-3.954165064	9.7514131784918 2e-05	0.00034885891411469	0.79046948156204 3	Down

REACTOME_TRANSPORT_AND_SYNTHESIS_OF_PAPS	-0.265155257	0.0106367564375202	-3.957888996	9.60868611722415e-05	0.000344348237468882	0.804261245857089	Down
REACTOME_ESTABLISHMENT_OF_SISTER_CHROMATID_COHESION	-0.255624915	0.011695108886244	-3.958586151	9.58218760677471e-05	0.000343596987147606	0.806844480187467	Down
RADAEVA_RESPONSE_TO_IFNA1_UP	-0.213829266	-0.019716581	-3.962219721	9.44519679208359e-05	0.000339665912447641	0.820314857292153	Down
TURJANSKI_MAPK11_TARGETS	-0.247331949	0.0142996679593883	-3.966279695	9.29432994821768e-05	0.000334514900389111	0.835379033394672	Down
ZHAN_MULTIPLE_MYELOMA_PR_DN	-0.127907932	0.00710710549207101	-3.966832953	9.2739491421199e-05	0.000334172637077344	0.837432913933523	Down
KIM_MYCN_AMPLIFICATION_TARGETS_DN	-0.111127727	-0.0007715	-3.9718	9.091677	0.000328	0.85	Down

		19	32888	44182924e-05	093266752524	6005932023423	
ZHAN_MULTIPLE_MYELOMA_CD2_DN	-0.127970612	0.000923294668844543	-3.972783327	9.0574164234472 2e-05	0.000327427977520891	0.85953883765553 2	Down
WP_DNA_REPLICATION	-0.19737844	-0.004916159	-3.974594833	8.9924552529574 5e-05	0.00032526905451694	0.86627453219637 1	Down
REACTOME_METABOLISM_OF_NUCLEOTIDES	-0.104744057	-0.000400603	-3.976417123	8.9275539583866 2e-05	0.00032310978087292	0.87305308503969 3	Down
ST_INTERFERON_GAMMA_PATHWAY	-0.166137045	0.00763724326256914	-3.978862562	8.8411579145117 1e-05	0.000320543616678588	0.88215397313113 8	Down
SMIRNOV_RESPONSE_TO_IR_2HR_UP	-0.136675802	0.0061388149073826	-3.978922785	8.8390403163075	0.0003205436166	0.882378	Down

		7		3e-05	78588	160974589	
REACTOME_SHC1_EVENTS_IN_ERBB4_SIGNALING	-0.14003259	0.00206017127551042	-3.979508754	8.81846126148526e-05	0.000320169991886656	0.884559661857908	Down
CROONQUIST_IL6_DEPRIVATION_DN	-0.183746376	-0.014859725	-3.982198473	8.72458175258589e-05	0.000317243579017578	0.89457686013682	Down
KORKOLA_EMBRYONAL_CARCINOMA_UP	-0.111082052	0.00819645035977415	-3.985763293	8.60162066926204e-05	0.00031313935187161	0.907862444306241	Down
GARGALOVIC_RESPONSE_TO_OXIDIZED_PHOSPHOLIPIDS_GREEN_UP	-0.152934661	0.0109842795267861	-3.986050862	8.59177367189088e-05	0.00031313935187161	0.908934633676356	Down
BENPORATH_CYCLING_GENES	-0.097192246	0.00201761325077241	-3.991976844	8.39122307263466e-05	0.000306558691064411	0.93104477782713	Down

						8	
XU_AKT1_TARGETS_6HR	-0.213142152	-0.003032567	-3.992049428	8.38879444047036e-05	0.000306558691064411	0.931315773526273	Down
REACTOME_DISORDERS_OF_TRANSMEMBRANE_TRANSPORTERS	-0.077277058	-0.001806672	-3.995892949	8.2611414434279e-05	0.000302340241387718	0.94567200421728	Down
APRELIKOVA_BRCA1_TARGETS	-0.106164876	0.00590176310302108	-3.996158257	8.25239832808399e-05	0.000302198444970014	0.946663431640026	Down
REACTOME_MEIOTIC_RECOMBINATION	-0.136289212	0.00521473962421386	-3.997021008	8.22402751800357e-05	0.000301337301087652	0.949887838799188	Down
GHO_ATF5_TARGETS_UP	-0.209106623	0.00514696110756189	-3.99730523	8.21470144606491e-05	0.000301173371977111	0.950950212359213	Down

LEE_LIVER_CANCER_MYC_UP	-0.103683492	-0.003537604	-3.997874396	8.1960557790825e-05	0.000300845169844856	0.95307786440106	Down
CHIANG_LIVER_CANCER_SUBCLASS_PROLIFERATION_UP	-0.135583373	-0.009364063	-4.005852567	7.9388903641601 5e-05	0.000291923533710557	0.982930128279342	Down
ISHIDA_E2F_TARGETS	-0.212566819	-0.011205462	-4.006763725	7.9100132691259 8e-05	0.000291206716260172	0.986342813245866	Down
ZHENG_FOXP3_TARGETS_UP	-0.19413079	0.00661814791370252	-4.018292385	7.5531545970853 6e-05	0.000279228294128105	1.02958229231723	Down
THEILGAARD_NEUTROPHIL_AT_SKIN_WOUND_UP	-0.138306663	0.00825344439804845	-4.020684961	7.4810378434313 5e-05	0.00027672706730738	1.03856974059128	Down
GAVIN_PDE3B_TARGETS	-0.123948204	-0.000456776	-4.026206288	7.3171051292704 2e-05	0.000271147889775412	1.05932812988149	Down

SOUCEK_MYC_TARGETS	-0.180689035	-0.00326332	-4.028297044	7.2559251258296 2e-05	0.000269041381457733	1.06719529898352	Down
CREIGHTON_ENDOCRINE_THERAPY_RESISTANCE_5	-0.081692644	0.00563306281303127	-4.029147357	7.2311825737885 3e-05	0.00026828422137182	1.07039592185473	Down
XU_HGF_TARGETS_REPRESSED_BY_AKT1_UP	-0.184787344	-0.003806569	-4.031582848	7.160757483164e-05	0.000265830273313391	1.07956655942362	Down
REACTOME_ACTIVATION_OF_RAS_IN_B_CELLS	-0.288692101	0.00469594479281348	-4.033437545	7.1075647587120 1e-05	0.000264013491665622	1.08655356552868	Down
ZHENG_FOXP3_TARGETS_IN_THYMUS_UP	-0.192551896	0.00985014511601381	-4.033936833	7.0933094929097 5e-05	0.00026364174863767	1.08843496974977	Down
WILLIAMS_ESR2_TARGETS_UP	-0.12911654	0.00144416855025444	-4.038125679	6.9747794687866 7e-05	0.000259547099297116	1.10422740243024	Down
WP_MITOCHONDRIAL_CIII_ASSEMBLY	-0.259655208	-0.004729518	-4.048128816	6.6992995068069	0.0002501958606	1.141999	Down

				4e-05	42303	22028549	
SHEPARD_CRUSH_AND_BURN_MUTANT_UP	-0.092251372	-0.001687428	-4.048812011	6.6808673636482 8e-05	0.000249657698531998	1.14458198922464	Down
GENTLES_LEUKEMIC_STEM_CELL_DN	-0.187289942	-0.009584279	-4.050544774	6.6343347563051 6e-05	0.000248217696397746	1.15113430016078	Down
WAKASUGI_HAVE_ZNF143_BINDING_SITES	-0.163211492	-0.000744808	-4.05252655	6.5814930378814 5e-05	0.000246602847814302	1.15863128468214	Down
BOSCO_INTERFERON_INDUCED_ANTIVIRAL_MODULE	-0.262512569	-0.026701376	-4.056453406	6.4779686496991 e-05	0.000243100068976314	1.17349604198558	Down
REACTOME_BUTYRATE_RESPONSE_FACTOR_1_BRF1_ BINDS_AND_DESTABILIZES_MRNA	-0.177862886	0.0039845959164028	-4.059345861	6.4027074085433 2e-05	0.00024042108218287	1.18445332983547	Down
REACTOME_RHO_GTPASES_ACTIVATE_ROCKS	-0.198581072	0.00220523729350136	-4.059443344	6.4001854853097 8e-05	0.00024042108218287	1.18482273595533	Down

KEGG_NICOTINATE_AND_NICOTINAMIDE_METABOLISM	-0.129992319	0.00323117024238715	-4.061990867	6.3346137621695 2e-05	0.00023829665225325	1.19447928268158	Down
SENESE_HDAC2_TARGETS_UP	-0.13037259	0.00585581349479838	-4.062308701	6.3264779291763 4e-05	0.00023813492120313 9	1.19568442605564	Down
WP_PYRIMIDINE_METABOLISM	-0.160139566	-0.00268665	-4.063380353	6.2991192142108 4e-05	0.00023739303559566 9	1.199748 4770487	Down
BROWNE_HCMV_INFECTION_16HR_UP	-0.113668878	0.00523022367349572	-4.066368617	6.2234239389832 9e-05	0.00023496832353570 1	1.21108595392553	Down
REACTOME_DNA_REPLICATION_INITIATION	-0.26324145	-0.0090056	-4.069305514	6.1498739363321 4e-05	0.00023247422364685 5	1.22223574280193	Down
BIOCARTA_SODD_PATHWAY	-0.19188532	-0.000917869	-4.069415184	6.1471435164850 6e-05	0.00023247422364685 5	1.22265223709722	Down
HEIDENBLAD_AMPLICON_8Q24_DN	-0.102794552	0.0019523359198377	-4.069417746	6.1470797486243	0.0002324742236	1.222661	Down

		9		6e-05	46855	9663 7747	
GRAHAM_CML_DIVIDING_VS_NORMAL_DIVIDING_DN	-0.201280775	0.0142480 863947947	-4.0694 19571	6.147034 3228830 8e-05	0.000232 4742236 46855	1.22 2668 8971 9988	Down
CROSBY_E2F4_TARGETS	-0.293589356	-0.0223133 04	-4.0698 19299	6.137092 3504418 7e-05	0.000232 4742236 46855	1.22 4187 0320 0841	Down
SMITH_TERT_TARGETS_UP	-0.11800451	0.0048828 673817333 2	-4.0703 1501	6.124784 3264280 8e-05	0.000232 2329646 55706	1.22 6069 8916 4983	Down
BIDUS_METASTASIS_UP	-0.169220885	0.0099803 788460265 2	-4.0709 48347	6.109093 4049629 3e-05	0.000231 7796012 50641	1.22 8475 7859 9397	Down
ZHU_CMV_8_HR_UP	-0.20268946	-0.0230178 76	-4.0815 4509	5.852167 8470150 2e-05	0.000222 7124820 74937	1.26 8779 4582 5255	Down
REACTOME_SYNTHESIS_OF_ACTIVE_UBIQUITIN_ROLES_OF_E1_AND_E2_ENZYMES	-0.132077341	0.0006887 271576720 2	-4.0891 95636	5.673105 8830522 8e-05	0.000216 5619201 4825	1.29 7935 2111 6147	Down

WAMUNYOKOLI_OVARIAN_CANCER_GRADES_1_2_UP	-0.115752471	0.0027340688872508	-4.094693181	5.5476699911483 2e-05	0.000212679258768974	1.31891584350688	Down
REACTOME_REGULATION_BY_C_FLIP	-0.207872752	-0.002376981	-4.098858511	5.4543945089440 1e-05	0.00020924243953656	1.33482885470451	Down
GOLDRATH_IMMUNE_MEMORY	-0.098977557	0.00232114928007632	-4.103763088	5.3464796371235 8e-05	0.000205483585805734	1.35358439435332	Down
WANG_RESPONSE_TO_FORSKOLIN_DN	-0.254648482	0.00754702092713479	-4.104473276	5.3310230562172 7e-05	0.000205016481474229	1.35630185951277	Down
DURAND_STROMA_NS_UP	-0.063647746	-0.000589217	-4.104560096	5.3291364296641 9e-05	0.000205016481474229	1.35663409501905	Down
TOMIDA_LUNG_CANCER_POOR_SURVIVAL	-0.235428492	-0.013256546	-4.109175266	5.2297574497742 4e-05	0.000201621767023284	1.37430405149535	Down
REACTOME_DISEASES_OF_SIGNAL_TRANSDUCTION_BY_GROWTH_FACTOR_RECEPTORS_AND_SECOND_MES	-0.070205748	0.0043130733492935	-4.111018243	5.1905678435249	0.0002004844717	1.381365	Down

SENGERS		2		6e-05	15989	1034 6396	
REACTOME_TYSND1_CLEAVES_PEROXISOMAL_PROTEINS	-0.254253616	0.0213861964947059	-4.111117758	5.1884597199498 4e-05	0.000200484471715989	1.38174645588786	Down
FLOTHO_PEDIATRIC_ALL_THERAPY_RESPONSE_UP	-0.195996628	0.000864242194555714	-4.113180692	5.1449416557230 6e-05	0.000199093845742351	1.38965372217827	Down
BURTON_ADIPOGENESIS_3	-0.16741191	-0.00724841	-4.126419687	4.8738309788333 7e-05	0.000189785877576027	1.44048244402605	Down
BYSTRYKH_HEMATOPOIESIS_STEM_CELL_AND_BRAIN_QTL_CIS	-0.127261687	0.00724100266040808	-4.132500795	4.7539158995206 9e-05	0.000185465468185574	1.46387808703567	Down
REACTOME_UCH_PROTEINASES	-0.169885576	-0.001687113	-4.134087726	4.7230885912751 7e-05	0.000184378684817893	1.46998843481681	Down
YAGI_AML_SURVIVAL	-0.094982507	0.0060875826490929	-4.136065166	4.6849419842735 2e-05	0.000183004624898589	1.47760530199159	Down

DER_IFN_BETA_RESPONSE_UP	-0.182079393	-0.0015755 37	-4.1441 75721	4.531532 9313264 9e-05	0.000177 5708642 97623	1.50 8879 8371 1016	Down
REACTOME_CHOLESTEROL_BIOSYNTHESIS	-0.193227559	-0.0051963 19	-4.1475 05486	4.469948 4458208 9e-05	0.000175 3790771 37865	1.52 1735 1526 7984	Down
KEGG_RIG_I_LIKE_RECEPTOR_SIGNALING_PATHWAY	-0.111175826	-0.0019718 68	-4.1489 33657	4.443779 5216989 e-05	0.000174 4626153 77515	1.52 7251 7182 6173	Down
TAKEDA_TARGETS_OF_NUP98_HOXA9_FUSION_10D_U P	-0.132583763	-0.0030218 88	-4.1545 82016	4.341706 8501686 9e-05	0.000170 9960305 77393	1.54 9085 9302 898	Down
WP_TYPE_II_INTERFERON_SIGNALING_IFNG	-0.221881329	-0.0131371 76	-4.1546 18	4.341063 7953882 5e-05	0.000170 9960305 77393	1.54 9225 1147 0872	Down
REACTOME_REGULATION_OF_TP53_ACTIVITY_THROU GH_PHOSPHORYLATION	-0.138518742	0.0041771 999389104 1	-4.1552 936	4.329007 3915601 5e-05	0.000170 9297002 50724	1.55 1838 4775 7309	Down
SCHRAETS_MLL_TARGETS_UP	-0.114483499	0.0049419 013280287	-4.1610 27524	4.227960 6376686	0.000167 3657632	1.57 4033	Down

		2		9e-05	52612	5699 6588	
CHEN_ETV5_TARGETS_SERTOLI	-0.177631838	0.0051191179149722 9	-4.1625 14271	4.2021298912251 8e-05	0.000166662110126739	1.57 9792 9285 2458	Down
GAZDA_DIAMOND_BLACKFAN_ANEMIA_MYELOID_UP	-0.143339672	0.013649286172163	-4.1667 51368	4.1293377954782 3e-05	0.00016408962673837	1.59 6216 5329 6493	Down
MCLACHLAN_DENTAL_CARIES_UP	-0.148717404	0.0068628950669402 5	-4.1725 16021	4.0322321947094 6e-05	0.000160642267217982	1.61 8584 7968 6956	Down
REACTOME_INFECTIOUS_DISEASE	-0.062488183	0.0029440829151135 1	-4.1755 09502	3.9826703175301 3e-05	0.000158973856340254	1.63 0210 9716 6787	Down
GILDEA_METASTASIS	-0.16089963	-0.0014159 54	-4.1756 74056	3.9799627825970 5e-05	0.000158968011528828	1.63 0850 2850 5769	Down
BENNETT_SYSTEMIC_LUPUS_ERYTHEMATOSUS	-0.305225292	-0.0304756 68	-4.1790 95682	3.9240604867180 5e-05	0.000157038320058407	1.64 4148 7547 1566	Down

LIU_SOX4_TARGETS_UP	-0.098067614	0.0033283963972281 8	-4.180103766	3.9077338876278 4e-05	0.000156586857588806	1.64806859630522	Down
REACTOME_NUCLEOBASE_CATABOLISM	-0.117267144	-0.001948139	-4.181149628	3.8908639732842 9e-05	0.000156011580634209	1.65213621217567	Down
SHAFFER_IRF4_MULTIPLE_MYELOMA_PROGRAM	-0.16953069	0.0024483052615384 1	-4.182219348	3.8736811301697 e-05	0.000155423004363047	1.65629754419484	Down
REACTOME_COPII_MEDIATED_VESICLE_TRANSPORT	-0.123983539	0.0088354209900695 3	-4.182946617	3.8620404280488 4e-05	0.000155156536808409	1.65912723901535	Down
WP_MEVALONATE_PATHWAY	-0.222835602	0.0013789157065650 1	-4.186196472	3.8104297622076 4e-05	0.000153182237914656	1.67177722434856	Down
YAO_TEMPORAL_RESPONSE_TO_PROGESTERONE_CLUSTER_12	-0.121860485	0.0028403026764576 6	-4.192135689	3.7178056160664 2e-05	0.000149749639577704	1.69491780972008	Down
WP_LDLRAD4_AND_WHAT_WE_KNOW_ABOUT_IT	-0.271377619	0.0034766656840307	-4.197902974	3.6299221505904	0.0001464949726	1.717416	Down

		7		5e-05	18433	11370402	
VETTER_TARGETS_OF_PRKCA_AND_ETS1_DN	-0.187302001	-0.002302982	-4.199781607	3.601725764591e-05	0.00014545160586087 4	1.72475057465208	Down
REACTOME_INTEGRATION_OF_PROVIRUS	-0.22968029	0.00628461879132817	-4.20368305	3.5438356157451e-05	0.000143206950956574	1.73999159150035	Down
WP_INTRAFLAGELLAR_TRANSPORT_PROTEINS_BINDING_TO_DYNEIN	-0.168024767	-0.000988257	-4.205743563	3.5136209987972 2e-05	0.000142078472570256	1.74804602017762	Down
CHANG_IMMORTALIZED_BY_HPV31_DN	-0.138309276	-0.003542397	-4.208950931	3.4670781906012 4e-05	0.000140287837868722	1.76059034208794	Down
WP_DNA_MISMATCH_REPAIR	-0.230752507	7.48308408765395e-05	-4.209778581	3.4551638869444 1e-05	0.000139896948768845	1.76382872109007	Down
WANG_RECURRENT_LIVER_CANCER_DN	-0.212892116	0.00631070236030662	-4.212577828	3.4151567844154 9e-05	0.000138367350919497	1.77478559751584	Down

REACTOME_HDR_THROUGH_HOMOLOGOUS_RECOMBINATION_HRR_	-0.159308816	-0.000752076	-4.214310344	3.3906175187379e-05	0.000137552698946441	1.7815702581742	Down
REACTOME_NOD1_2_SIGNALING_PATHWAY	-0.135500809	0.0051761652337416	-4.216551816	3.3591192164069e-05	0.000136363982839994	1.79035165526095	Down
DER_IFN_ALPHA_RESPONSE_DN	-0.311027935	0.00913146960083028	-4.222576065	3.2758421705467 8e-05	0.0001333321465 7432	1.8139731332 8401	Down
HOSHIDA_LIVER_CANCER_SURVIVAL_DN	-0.064846878	-0.002253396	-4.222843329	3.2721936759753 2e-05	0.000133271037708523	1.8150217778398	Down
BIOCARTA_FAS_PATHWAY	-0.152344985	0.012801770047934	-4.224644568	3.2477054761643 e-05	0.000132534568642681	1.8220906952 1204	Down
GENTILE_UV_HIGH_DOSE_DN	-0.142553606	0.00963014246517737	-4.225722838	3.2331300486698 1e-05	0.000132088243187176	1.8263236030 9933	Down
KORKOLA_TERATOMA_UP	-0.136799425	0.0008538262355942	-4.226637962	3.2208089678326	0.0001316967145	1.829916	Down

		04		2e-05	14737	80140588	
RHEIN_ALL_GLUCOCORTICOID_THERAPY_DN	-0.196715868	0.000506693926182245	-4.228584312	3.19475258807493e-05	0.000130717398247733	1.83756134035557	Down
CHIARADONNA_NEOPLASTIC_TRANSFORMATION_CDC25_UP	-0.084941172	0.00450939363497677	-4.22964306	3.18066348734481e-05	0.000130226769564309	1.84172100569136	Down
IKEDA_MIR133_TARGETS_DN	-0.275489346	0.000302187400587129	-4.229657991	3.18046522188713e-05	0.000130226769564309	1.8417796736008	Down
YAMAZAKI_TCEB3_TARGETS_DN	-0.093611782	0.00542644793407971	-4.23661483	3.08935946717776e-05	0.000126739287592679	1.86913486878235	Down
WP_REGULATION_OF_APOPTOSIS_BY_PARATHYROID_HORMONERELATED_PROTEIN	-0.139275733	0.0027473031413091	-4.23734806	3.07990376747855e-05	0.000126434938391134	1.87202032026355	Down
REACTOME_NOTCH2_ACTIVATION_AND_TRANSMISSION_OF_SIGNAL_TO_THE_NUCLEUS	-0.153364786	0.000766474502650268	-4.244356204	2.99091138648621e-05	0.000123107340689124	1.89962129116455	Down

REACTOME_ESTROGEN_STIMULATED_SIGNALING_THROUGH_PRKCZ	-0.231549684	0.00498371820044782	-4.244420711	2.9901037836464 6e-05	0.00012310734068912 4	1.8998755298 8727	Down
RAHMAN_TP53_TARGETS_PHOSPHORYLATED	-0.200248835	0.00240087592996638	-4.246316367	2.9664635068334 4e-05	0.00012226320708443	1.9073483790 5614	Down
PEPPER_CHRONIC_LYMPHOCYTIC_LEUKEMIA_UP	-0.14853074	0.00188383121283573	-4.247656959	2.9498532365641 6e-05	0.000121659395610324	1.9126348780 7631	Down
JONES_TCOF1_TARGETS	-0.285111613	0.0118423377822489	-4.25548932	2.8545707846511 8e-05	0.000118043443439906	1.9435502867 6996	Down
GREENBAUM_E2A_TARGETS_UP	-0.209079694	-0.009682579	-4.256125725	2.8469593575993 3e-05	0.000117885768729947	1.9460644551 4425	Down
KEGG_GLUTATHIONE_METABOLISM	-0.122454638	-0.001923156	-4.272351093	2.6593279813589 5e-05	0.000110930435351445	2.0102752502 7522	Down
VANTVEER_BREAST_CANCER_ESR1_DN	-0.082620001	-0.001312987	-4.285699937	2.5138837548678	0.0001052169687	2.063262	Down

				4e-05	55662	6464 0602	
JAEGER_METASTASIS_UP	-0.130183871	-0.000258939	-4.285775833	2.5130789940815 3e-05	0.000105216968755662	2.06 3564 3225 8183	Down
REACTOME_INTERCONVERSION_OF_NUCLEOTIDE_DI_AND_TRIPHOSPHATES	-0.182601929	-0.004769503	-4.286334493	2.5071628090670 8e-05	0.000105077377149759	2.06 5785 0738 4508	Down
REACTOME_SIGNALING_BY_NOTCH1_HD_DOMAIN_MUTANTS_IN_CANCER	-0.15813512	-0.002025237	-4.287158196	2.4984641183749 1e-05	0.000104783559342926	2.06 9059 8710 2446	Down
HAHTOLA_MYCOSIS_FUNGOIDES_SKIN_UP	-0.127970347	0.00692831040217786	-4.292096123	2.4469194125727 8e-05	0.000102899924077502	2.08 8703 1128 7224	Down
ACEVEDO_LIVER_CANCER_WITH_H3K9ME3_DN	-0.092642002	0.00359500258847258	-4.292969813	2.4379059196227 4e-05	0.00010259038673287	2.09 2180 7389 9506	Down
REACTOME_GAP_FILLING_DNA_REPAIR_SYNTHESIS_AND_LIGATION_IN_GG_NER	-0.196288869	0.00214653786245598	-4.302137714	2.3452197182406 8e-05	9.9093116345268 4e-05	2.12 8709 8403 2993	Down

WU_HBX_TARGETS_3_UP	-0.144204899	-0.002314042	-4.303690988	2.3298541070039 4e-05	9.8510929442598 3e-05	2.13490553554923	Down
ENK_UV_RESPONSE_KERATINOCYTE_DN	-0.163444325	0.00986608741941975	-4.303790986	2.3288682004126 7e-05	9.8510929442598 3e-05	2.13530447144261	Down
WIEMANN_TELOMERE_SHORTENING_AND_CHRONIC_LIVER_DAMAGE_DN	-0.236524285	-0.002060342	-4.305678475	2.3103335129118 9e-05	9.7818827521446 7e-05	2.14283606626722	Down
ZHAN_MULTIPLE_MYELOMA_CD1_AND_CD2_DN	-0.114322304	0.00519685144058168	-4.310106053	2.2674079962257 3e-05	9.6198232621825 9e-05	2.16051460980834	Down
REACTOME_MET_ACTIVATES_PI3K_AKT_SIGNALING	-0.250795574	0.000604353027173675	-4.324125072	2.1364692809075 5e-05	9.0829211141048 9e-05	2.216594 6773634	Down
KIM_WT1_TARGETS_12HR_DN	-0.105334926	0.00264199579622916	-4.328788665	2.0945384484816 3e-05	8.9219414900220 9e-05	2.23528557833157	Down
WACKER_HYPOXIA_TARGETS_OF_VHL	-0.176108713	-0.002062561	-4.331778117	2.0680755263658	8.8223675547441	2.247276	Down

				4e-05	9e-05	04180313	
ODONNELL_METASTASIS_DN	-0.138071349	0.00090636589400531	-4.332703094	2.05995257811531e-05	8.79375904564082e-05	2.25098751606297	Down
VANOEVELEN_MYOGENESIS_SIN3A_TARGETS	-0.099217718	0.00303037059764782	-4.333557159	2.05247948696409e-05	8.77392574076179e-05	2.25441507013872	Down
REACTOME_SIGNALING_BY_FGFR1_IN_DISEASE	-0.146512083	0.00513308684430078	-4.339888174	1.99788662908159e-05	8.56549887629365e-05	2.27984119408143	Down
REACTOME_VXPX_CARGO_TARGETING_TO_CILIUM	-0.152682621	0.00517856056442367	-4.340973356	1.98866969170284e-05	8.53642653969539e-05	2.28420266625064	Down
XU_HGF_TARGETS_INDUCED_BY_AKT1_48HR_DN	-0.194148847	-0.005353661	-4.341691739	1.98259054803626e-05	8.51622112917719e-05	2.28709044933882	Down
REACTOME_PHOSPHORYLATION_OF_THE_APC_C	-0.184668172	-0.015541608	-4.34511383	1.95387536997705e-05	8.41617239517527e-05	2.30085242138728	Down

BLANCO_MELO_COVID19_SARS_COV_2_LOW_MOI_INFECTION_A594_ACE2_EXPRESSING_CELLS_UP	-0.107100303	0.00103190126885517	-4.349188723	1.9202017401623 5e-05	8.2852225062412 1e-05	2.31725196184373	Down
REACTOME_JNK_C_JUN_KINASES_PHOSPHORYLATION_AND_ACTIVATION_MEDIATED_BY_ACTIVATED_HUMAN_TAK1	-0.14936533	0.00431505800227242	-4.35065141	1.9082506484382 7e-05	8.2425273311456 7e-05	2.32314185949619	Down
TERAMOTO_OPN_TARGETS_CLUSTER_1	-0.181265309	0.00553830753583812	-4.351993923	1.8973441733308 4e-05	8.2011248494878 5e-05	2.32854936123124	Down
REACTOME_GENOME_REPLICATION_AND_TRANSCRIPTION	-0.183378815	0.00839286669267593	-4.353114112	1.8882895519316 8e-05	8.1676747378675 4e-05	2.33306247545405	Down
REACTOME_INFLUENZA_INFECTION	-0.240143193	0.00204822572554422	-4.355070693	1.8725734460896 2e-05	8.1053440584925 2e-05	2.34094774347855	Down
MARCINIAK_ER_STRESS_RESPONSE_VIA_CHOP	-0.142557302	-0.006462847	-4.355339349	1.8704252904487 7e-05	8.1016955881476 1e-05	2.342030 7019281	Down
TAKEDA_TARGETS_OF_NUP98_HOXA9_FUSION_16D_UP	-0.119421838	0.0003613654793440	-4.355876273	1.8661391521701	8.0887749424024	2.344195	Down

		22		9e-05	9e-05	22837861	
NAKAYAMA_FRA2_TARGETS	-0.126770059	0.00507925447521576	-4.356076346	1.8645444204792 1e-05	8.0875102850555 2e-05	2.34500185264084	Down
REACTOME_PROTEIN_UBIQUITINATION	-0.140323182	0.00623157843901568	-4.357719921	1.8514932242852 1e-05	8.0365163938029 9e-05	2.35162938173279	Down
HASINA_NOL7_TARGETS_DN	-0.236975269	0.00912509575162967	-4.357861351	1.8503742578968 6e-05	8.0365163938029 9e-05	2.35219978654912	Down
REACTOME_APC_CDC20_MEDIATED_DEGRADATION_OF_NEK2A	-0.187494356	-0.012054174	-4.358775524	1.8431571461619 6e-05	8.0115380995989 6e-05	2.35588712948049	Down
LANDIS_ERBB2_BREAST_TUMORS_324_UP	-0.082147681	0.00383233909474796	-4.363954426	1.8027775656511 7e-05	7.8580339536494 6e-05	2.37678909259222	Down
GALE_APL_WITH_FLT3_MUTATED_DN	-0.22132044	0.000920323995838778	-4.364675748	1.7972211430111 1e-05	7.8393194902810 6e-05	2.37970204843271	Down

REACTOME_HIV_TRANSCRIPTION_ELONGATION	-0.184169716	0.00326431550393033	-4.365613919	1.7900188442171 7e-05	7.8133944908973 e-05	2.38349134607558	Down
YAMANAKA_GLIOBLASTOMA_SURVIVAL_DN	-0.256481339	0.00850858064612662	-4.36605863	1.7866144872011 3e-05	7.8040226052480 1e-05	2.38528779101917	Down
WP_CYTOPLASMIC_RIBOSOMAL_PROTEINS	-0.298530364	-0.000210039	-4.367127155	1.7784600229663 2e-05	7.7793526163156 7e-05	2.38960483904057	Down
REACTOME_RNA_POLYMERASE_I_TRANSCRIPTION_TERMINATION	-0.214456442	-0.005409518	-4.372095765	1.7410080700873 4e-05	7.6316646123108 4e-05	2.40969104134737	Down
GRADE_COLON_VS_RECTAL_CANCER_UP	-0.162257247	0.00615413412368154	-4.37254988	1.7376230229524 5e-05	7.6222092603999 e-05	2.41152784459132	Down
REACTOME_N_GLYCAN_TRIMMING_AND_ELONGATION_IN_THE_CIS_GOLGI	-0.254289242	0.0179767316628795	-4.372940149	1.7347189278052 9e-05	7.6148517573461 3e-05	2.41310653894435	Down
REACTOME_INTERLEUKIN_12_FAMILY_SIGNALING	-0.117658652	0.0041188767189706	-4.375274642	1.7174442207621	7.5496999137892	2.422552	Down

		3		1e-05	6e-05	44705328	
WP_APOPTOSIS_MODULATION_BY_HSP70	-0.176434602	0.0118757011974103	-4.377525381	1.7009453990553 4e-05	7.4824720708267 1e-05	2.431663616 68232	Down
DAIRKEE_CANCER_PRONE_RESPONSE_BPA_E2	-0.090338675	0.00382858137873149	-4.384289908	1.6522677472189 9e-05	7.2941862780855 2e-05	2.459071469 02826	Down
GARY_CD5_TARGETS_DN	-0.220238618	0.000966128300251898	-4.386038209	1.6399057039972 5e-05	7.2447649143850 1e-05	2.466161043 04991	Down
LY_AGING_PREMATURE_DN	-0.17086743	0.0027226837812425	-4.387943943	1.6265314390243 3e-05	7.1907981780798 e-05	2.473891824 23476	Down
REACTOME_HIV_INFECTION	-0.146476712	0.00304838027801951	-4.39180222	1.5997737207752 7e-05	7.0825930705079 3e-05	2.489552201 33698	Down
PLASARI_TGFB1_SIGNALING_VIA_NFIC_1HR_UP	-0.137047496	7.4833637067329e-05	-4.391809699	1.5997222663690 4e-05	7.0825930705079 3e-05	2.489582568 57001	Down

GAZDA_DIAMOND_BLACKFAN_ANEMIA_PROGENITOR_UP	-0.141795544	0.0106747127131896	-4.394061363	1.5843025939963e-05	7.0241187149535 9e-05	2.49872739396264	Down
YANG_BREAST_CANCER_ESR1_LASER_DN	-0.135845367	0.00571762288785743	-4.399098056	1.5503247264432 3e-05	6.9030599548300 6e-05	2.51919796093421	Down
ALCALAY_AML_BY_NPM1_LOCALIZATION_DN	-0.130184324	-0.000739738	-4.399497841	1.5476578783236e-05	6.896132412602e-05	2.52082367405173	Down
KEGG_MISMATCH_REPAIR	-0.234552167	0.00460935677421596	-4.402701714	1.5264440295379 8e-05	6.8162863966491e-05	2.53385675373118	Down
RAY_TARGETS_OF_P210_BCR_ABL_FUSION_UP	-0.177098252	0.0028015837783833	-4.405058357	1.5110181677414 2e-05	6.7522604515270 2e-05	2.54344862866949	Down
GENTILE_UV_RESPONSE_CLUSTER_D6	-0.168420168	0.010728126213182	-4.405140463	1.5104834349733 5e-05	6.7522604515270 2e-05	2.54378288908545	Down
VANLOO_SP3_TARGETS_DN	-0.098363949	-0.001769223	-4.410278989	1.4773759385777	6.6257748921617	2.564713	Down

				2e-05	7e-05	2055552	
AIYAR_COBRA1_TARGETS_UP	-0.09908198	0.00217605443705501	-4.410617185	1.4752215223348e-05	6.6208965937325 6e-05	2.56609149087418	Down
PID_RAS_PATHWAY	-0.10388396	0.00463771704619014	-4.412845681	1.4611004103309 4e-05	6.5670168333991 1e-05	2.57517581208871	Down
APPIERTO_RESPONSE_TO_FENRETINIDE_DN	-0.142273838	0.00711799390277491	-4.41319548	1.4588956670565 6e-05	6.5618589894348 3e-05	2.57660210922482	Down
GINESTIER_BREAST_CANCER_ZNF217_AMPLIFIED_UP	-0.184450121	0.00603521890336176	-4.418758102	1.4242609167558 6e-05	6.4153755517443e-05	2.59929670202185	Down
REACTOME_ER_QUALITY_CONTROL_COMPARTMENT_ERQC_	-0.136263425	2.72891951021726e-05	-4.418949015	1.4230863346097 3e-05	6.4147399265959 3e-05	2.60007603578237	Down
AFFAR_YY1_TARGETS_DN	-0.092075141	-0.002185378	-4.421004676	1.4104976884585 6e-05	6.3626156629812 9e-05	2.60846938315991	Down

CUI_TCF21_TARGETS_2_DN	-0.130762885	0.0061591874837551 3	-4.421356172	1.4083558850094e-05	6.3593413731145 2e-05	2.60990489283367	Down
REACTOME_ABERRANT_REGULATION_OF_MITOTIC_EXIT_IN_CANCER_DUE_TO_RB1_DEFECTS	-0.175563995	-0.010581359	-4.423125053	1.3976246363765 8e-05	6.3183220087322 9e-05	2.6171305118264	Down
REACTOME_SIGNALING_BY_NOTCH1_T_7_9_NOTCH1_M1580_K2555_TRANSLOCATION_MUTANT	-0.257599944	-0.002980206	-4.425214105	1.3850519789361 4e-05	6.2689308129242 7e-05	2.62566720925914	Down
REACTOME_HYDROLYSIS_OF_LPC	-0.257851956	0.0035133766418312 5	-4.425951127	1.3806422593605 3e-05	6.2552164261684 8e-05	2.6286798054885	Down
HOFMANN_MYELODYSPLASTIC_SYNDROM_HIGH_RISK_DN	-0.151054359	-0.000520454	-4.428219659	1.3671536047132 3e-05	6.2122418919875 8e-05	2.63795521882121	Down
CHEN_ETV5_TARGETS_TESTIS	-0.21976296	0.0011079473851642	-4.431486831	1.3479489207046 1e-05	6.1294644328304 1e-05	2.65132103873428	Down
CHOW_RASSF1_TARGETS_DN	-0.134525518	0.0024433864487115	-4.435129629	1.3268413619261	6.0509065597352	2.666233	Down

		7		8e-05	6e-05	5881 9347	
STEIN_ESRRA_TARGETS_RESPONSIVE_TO_ESTROGEN_DN	-0.172637739	-0.003655534	-4.435964329	1.3220496724115 3e-05	6.0337958210723 2e-05	2.66 9652 1012 5853	Down
REACTOME_DUAL_INCISION_IN_GG_NER	-0.219114277	2.18791706405367e-05	-4.437743787	1.3118898468174 7e-05	5.9918324350228 3e-05	2.67 6941 7240 5368	Down
GROSS_HYPOXIA_VIA_ELK3_DN	-0.123234251	0.00725887004296452	-4.440000745	1.2991114202438 7e-05	5.9378384281691 4e-05	2.68 6191 0914 5143	Down
KIM_GERMINAL_CENTER_T_HELPER_UP	-0.204102052	0.00648184446990778	-4.440722477	1.2950503819179 4e-05	5.9236387034374 9e-05	2.68 9149 7190 6258	Down
YAGI_AML_FAB_MARKERS	-0.085393131	0.00195168119673462	-4.44104368	1.2932469578815 8e-05	5.9236387034374 9e-05	2.69 0466 5734 681	Down
REACTOME_NONSENSE_MEDIATED_DECAY_NMD_	-0.272354031	0.00101829632715009	-4.443636591	1.2787767533464 3e-05	5.8708338077080 6e-05	2.70 1099 9129 3092	Down

SENESE_HDAC3_TARGETS_UP	-0.112770381	0.00742560998858099	-4.449373913	1.2473086188456 8e-05	5.7433565260943 e-05	2.72464733357874	Down
SARRIO_EPITHELIAL_MESENCHYMAL_TRANSITION_DN	-0.121474	0.00341827477242931	-4.450411286	1.2416987044416 6e-05	5.7217697538748 3e-05	2.72890777969526	Down
FERREIRA_EWINGS_SARCOMA_UNSTABLE_VS_STABLE_UP	-0.169190341	-0.000359413	-4.454757749	1.2184559423490 3e-05	5.6271994301789 e-05	2.74676786188607	Down
WIELAND_UP_BY_HBV_INFECTION	-0.181252403	-0.00798395	-4.457267647	1.2052249652496 9e-05	5.5743899845788 7e-05	2.7570881682119	Down
BROWNE_INTERFERON_RESPONSIVE_GENES	-0.295675836	-0.026115499	-4.460029783	1.1908236644567 e-05	5.5160018546886 1e-05	2.76845144360268	Down
HOFMANN_MYELODYSPLASTIC_SYNDROM_RISK_DN	-0.163931614	0.00963434947742744	-4.465975445	1.1603821921008 1e-05	5.3991696149698 1e-05	2.79293221594455	Down
ZHENG_RESPONSE_TO_ARSENITE_UP	-0.237442854	0.0006861303771023	-4.46811745	1.1495993378313	5.3570293467861	2.801758	Down

		23		5e-05	6e-05	6523 5775	
CASORELLI_APL_SECONDARY_VS_DE_NOVO_UP	-0.206400278	0.0053664332791609 8	-4.4748 81823	1.116175 2034007 3e-05	5.209097 3590288 e-05	2.82 9656 2125 157	Down
TONKS_TARGETS_OF_RUNX1_RUNX1T1_FUSION_MON OCYTE_DN	-0.160541184	0.0121676 871337141	-4.4754 25945	1.113527 5059577 9e-05	5.200651 0379834 5e-05	2.83 1901 8562 6612	Down
RUTELLA_RESPONSE_TO_CSF2RB_AND_IL4_DN	-0.157335046	0.0072187 943992489 5	-4.4789 50592	1.096521 9925718 2e-05	5.132814 4855907 1e-05	2.84 6454 1398 9192	Down
KEGG_REGULATION_OF_AUTOPHAGY	-0.157348428	0.0007877 562485544 66	-4.4790 3566	1.096114 6587872 3e-05	5.132814 4855907 1e-05	2.84 6805 4837 7704	Down
DARWICHE_PAPILLOMA_RISK_LOW_UP	-0.084500499	-0.0003294 91	-4.4793 068	1.094817 3154530 9e-05	5.132576 3421581 1e-05	2.84 7925 3756 0152	Down
CROONQUIST_NRAS_SIGNALING_UP	-0.178317066	0.0034249 384380903	-4.4818 81121	1.082573 0121496 6e-05	5.082852 2590113 e-05	2.85 8561 0056 8696	Down

IVANOVA_HEMATOPOIESIS_INTERMEDIATE_PROGENITOR	-0.173130919	0.00045661545934379	-4.483779265	1.07362913223283e-05	5.04849698770392e-05	2.86640643820742	Down
ZHAN_MULTIPLE_MYELOMA_LB_DN	-0.23169109	-0.007462883	-4.484592828	1.06981745454296e-05	5.03820708675885e-05	2.86976994421802	Down
GAVIN_FOXP3_TARGETS_CLUSTER_T4	-0.159465333	0.00905333095749023	-4.484986551	1.06797746670782e-05	5.03336077134048e-05	2.87139789721114	Down
SANA_RESPONSE_TO_IFNG_DN	-0.13728552	0.000564574489930716	-4.486543916	1.06072914414194e-05	5.00680288797646e-05	2.8778384574895	Down
REACTOME_LAGGING_STRAND_SYNTHESIS	-0.252628938	0.00233809306131317	-4.488969289	1.0495348903729e-05	4.9615103309555e-05	2.88787254385434	Down
KONG_E2F3_TARGETS	-0.199043279	-0.007431491	-4.492814632	1.03201891651311e-05	4.88614905781609e-05	2.90379082286344	Down
SOTIRIOU_BREAST_CANCER_GRADE_1_VS_3_UP	-0.210593172	-0.016568727	-4.493793512	1.0276050307547	4.8689652106067	2.907844	Down

				6e-05	3e-05	8935 7108	
ONO_AML1_TARGETS_UP	-0.18514019	-8.94E-05	-4.4955 21007	1.019859 7278620 1e-05	4.835958 2359354 7e-05	2.91 5001 2470 7787	Down
COATES_MACROPHAGE_M1_VS_M2_UP	-0.112999335	0.0006382 259755575 9	-4.4968 59959	1.013895 0656721 2e-05	4.815031 8841827 5e-05	2.92 0549 6481 7003	Down
AMIT_EGF_RESPONSE_60_HELA	-0.162002021	0.0102505 927725379	-4.4978 47414	1.009517 7038988 e-05	4.801591 1019922 4e-05	2.92 4642 4127 4751	Down
LIANG_SILENCED_BY_METHYLATION_2	-0.199241842	-0.0004513 3	-4.4986 71119	1.005880 1272047 3e-05	4.787958 5502759 e-05	2.92 8057 0676 2255	Down
MOOTHA_PYR	-0.276808932	0.0056421 011292275 8	-4.5002 4536	9.989630 8067963 5e-06	4.758682 9177118 2e-05	2.93 4584 5548 1558	Down
SONG_TARGETS_OF_IE86_CMV_PROTEIN	-0.215245067	-0.0028834 83	-4.5011 82404	9.948675 1831839 6e-06	4.744452 1862648 e-05	2.93 8470 8822 6409	Down

BYSTRYKH_SCP2_QTL	-0.245179629	0.0112181089148304	-4.50222785	9.9031719427391 9e-06	4.7283837114294 e-05	2.94280761969009	Down
BERTUCCI_MEDULLARY_VS_DUCTAL_BREAST_CANCER_UP	-0.137273692	-0.001835331	-4.502456943	9.8932273618487 9e-06	4.7272719195531 5e-05	2.94375806270048	Down
BROWNE_HCMV_INFECTION_4HR_UP	-0.146212356	-0.001616651	-4.502988373	9.8701956660414 6e-06	4.7199001925361 6e-05	2.94596298137073	Down
SHAFFER_IRF4_TARGETS_IN_MYELOMA_VS_MATURE_B_LYMPHOCYTE	-0.136063416	0.002452023539917	-4.505029176	9.7822267282696 4e-06	4.6814403471372 1e-05	2.95443241693804	Down
REACTOME_FORMATION_OF_INCISION_COMPLEX_IN_GG_NER	-0.195874918	0.00377808957582326	-4.505440577	9.7645847250410 4e-06	4.6766031935439 6e-05	2.9561401485737	Down
BASSO_HAIRY_CELL_LEUKEMIA_UP	-0.159870434	0.000954646670182176	-4.505818533	9.7484038392508 3e-06	4.6724588903652 4e-05	2.95770917082141	Down
MAYBURD_RESPONSE_TO_L663536_DN	-0.199985766	0.0084680872155282	-4.506732134	9.7093974339722	4.6645688755933	2.961502	Down

				6e-06	3e-05	30407604	
REACTOME_RESOLUTION_OF_AP_SITES_VIA_THE_MULTIPLE_NUCLEOTIDE_PATCH_REPLACEMENT_PATHWAY	-0.246961129	-0.002537762	-4.509170291	9.60603214170871e-06	4.62241792488248e-05	2.97162840963034	Down
REACTOME_INHIBITION_OF_THE_PROTEOLYTIC_ACTIVITY_OF_APC_C_REQUIRED_FOR_THE_ONSET_OF_ANAPHASE_BY_MITOTIC_SPINDLE_CHECKPOINT_COMPONENTS	-0.210449372	-0.01101459	-4.510432652	9.55293063367427e-06	4.60365220832424e-05	2.9768730751143	Down
WP_PI3KAKTMTOR_SIGNALING_PATHWAY_AND_THERAPEUTIC_OPPORTUNITIES	-0.171234614	0.00857190587043121	-4.511971317	9.48858751710474e-06	4.57619756943816e-05	2.98326739694748	Down
REN_BOUND_BY_E2F	-0.220980061	-0.004107944	-4.51962869	9.17451674979957e-06	4.42816683250435e-05	3.015117537	Down
KRIGE_AMINO_ACID_DEPRIVATION	-0.139580051	0.00459685312529064	-4.520769235	9.12859985121488e-06	4.41630703635937e-05	3.01986550660333	Down
MENSE_HYPOXIA_UP	-0.118246211	0.00600100262144716	-4.521715374	9.09067707484177e-06	4.40139099871629e-05	3.02380496862497	Down
RUTELLA_RESPONSE_TO_CSF2RB_AND_IL4_UP	-0.074918905	0.0032514	-4.5231	9.034035	4.377381	3.02	Down

		026831232	35624	0976758 1e-06	4091548 6e-05	9719 8332 4741	
PETROVA_PROX1_TARGETS_UP	-0.197504333	-0.0105344 23	-4.5287 80726	8.812232 7650792 8e-06	4.276585 5178144 7e-05	3.05 3245 5805 4093	Down
BURTON_ADIPOGENESIS_12	-0.232346052	0.0139172 307194946	-4.5292 80044	8.792867 8318258 7e-06	4.270526 6535323 3e-05	3.05 5327 6864 7243	Down
WATANABE_COLON_CANCER_MSI_VS_MSS_UP	-0.132129802	-0.0099017 53	-4.5293 92383	8.788516 6671089 3e-06	4.270526 6535323 3e-05	3.05 5796 154	Down
REACTOME_TETRAHYDROBIOPTERIN_BH4_SYNTHESIS_RECYCLING_SALVAGE_AND_REGULATION	-0.180411837	0.0043399 412739250 1	-4.5372 49302	8.489276 8923251 5e-06	4.132779 7388754 7e-05	3.08 8585 3957 6546	Down
SCHAEFFER_PROSTATE_DEVELOPMENT_AND_CANCER_BOX4_DN	-0.21999289	0.0050429 655607487 8	-4.5398 69728	8.391670 3564025 5e-06	4.094897 6338200 2e-05	3.09 9532 0774 4675	Down
YANG_BREAST_CANCER_ESR1_BULK_UP	-0.145336402	0.0025618 065849674 1	-4.5413 78887	8.335947 0146494 9e-06	4.077322 5468817 5e-05	3.10 5838 9701 0771	Down

HU_GENOTOXIC_DAMAGE_24HR	-0.155642649	0.000667910616497914	-4.546711573	8.1418800903446e-06	3.9981526677823 5e-05	3.12813910921732	Down
TOMIDA_METASTASIS_DN	-0.180796192	-0.001960268	-4.547309412	8.1203962336981 5e-06	3.9907600492925 1e-05	3.13064054194852	Down
MARTINEZ_RESPONSE_TO_TRABECTEDIN_DN	-0.178419911	0.00817011389271576	-4.547621021	8.1092198940124 4e-06	3.9884253472373 4e-05	3.13194446634905	Down
WANG_IMMORTALIZED_BY_HOXA9_AND_MEIS1_UP	-0.16650575	0.00468662085307664	-4.549703825	8.0348956387922 2e-06	3.9550037454388 1e-05	3.14066190992981	Down
PURBEY_TARGETS_OF_CTBP1_NOT_SATB1_DN	-0.042688891	0.00117943872744119	-4.562872632	7.5799074214703 9e-06	3.7488832960212 5e-05	3.195858476064 2	Down
MOOTHA_HUMAN_MITODB_6_2002	-0.17138917	8.78555858108325e-05	-4.571576298	7.2928981914403 7e-06	3.6242609346893 8e-05	3.23241473956584	Down
GRABARCZYK_BCL11B_TARGETS_UP	-0.189145673	0.0052314326138659	-4.576920776	7.1218694077386	3.5449433371157	3.254891	Down

		1		1e-06	6e-05	7017 0083	
GRAHAM_CML_QUIESCENT_VS_NORMAL_QUIESCENT_DN	-0.135059701	0.00188790372775137	-4.57912358	7.0525055803124 6e-06	3.5132345214285 3e-05	3.26 4162 4640 1334	Down
SANA_RESPONSE_TO_IFNG_UP	-0.260970463	-0.021826695	-4.581850868	6.9675260942163 9e-06	3.4736895154057 1e-05	3.27 5645 8771 9298	Down
ZHANG_BREAST_CANCER_PROGENITORS_UP	-0.219298123	0.00284743033218934	-4.584433925	6.8879492524303 6e-06	3.4423108703571 e-05	3.28 6527 3997 5869	Down
GARGALOVIC_RESPONSE_TO_OXIDIZED_PHOSPHOLIPIDS_GREY_DN	-0.09874239	0.000602768904352978	-4.587459468	6.7958518427152 7e-06	3.4045078601883 5e-05	3.29 9279 6532 0675	Down
BERNARD_PPAPDC1B_TARGETS_UP	-0.167768682	-2.62E-05	-4.592172751	6.6547349781247 4e-06	3.3446105270623 7e-05	3.31 9159 8648 7011	Down
REACTOME_TNF_SIGNALING	-0.110924411	-4.28E-05	-4.592443424	6.6467171348664 e-06	3.3432879462006 3e-05	3.32 0302 0690 0472	Down

REACTOME_TRANSLATION	-0.28352597	-0.0048262 25	-4.5954 89858	6.557114 2661598 9e-06	3.300892 8021130 9e-05	3.33 3161 6139 4802	Down
REACTOME_G2_M_DNA_DAMAGE_CHECKPOINT	-0.18415208	0.0030692 281181265 8	-4.6007 00267	6.406550 0643012 8e-06	3.227715 6046362 1e-05	3.35 5172 6173 9825	Down
GRANDVAUX_IRF3_TARGETS_UP	-0.264262848	-0.0041532 91	-4.6015 98578	6.380930 1221378 6e-06	3.220035 2250495 7e-05	3.35 8969 6279 5193	Down
JAERVINEN_AMPLIFIED_IN_LARYNGEAL_CANCER	-0.134235282	0.0095127 822250788	-4.6044 98106	6.298905 9368742 8e-06	3.181229 3856939 5e-05	3.37 1229 7800 6168	Down
WANG_METASTASIS_OF_BREAST_CANCER_ESR1_UP	-0.233878196	-0.0063849 53	-4.6066 34235	6.239127 3133607 9e-06	3.157927 5058770 5e-05	3.38 0266 2565 7782	Down
REACTOME_PERK_REGULATES_GENE_EXPRESSION	-0.206220466	0.0037428 086542765 9	-4.6098 17987	6.151043 2233484 6e-06	3.126906 2479380 7e-05	3.39 3741 1595 7522	Down
WEST_ADRENOCORTICAL_TUMOR_UP	-0.138948774	0.0009666 067721989	-4.6099 32473	6.147898 1526971	3.126906 2479380	3.39 4225	Down

		69		6e-06	7e-05	8589 6163	
ELVIDGE_HYPOXIA_BY_DMOG_UP	-0.104408906	0.0013233 757553875 4	-4.6113 11261	6.110142 5330448 8e-06	3.111210 3939794 6e-05	3.40 0064 0320 9993	Down
CHENG_RESPONSE_TO_NICKEL_ACETATE	-0.128991766	0.0080717 340024611 6	-4.6139 20388	6.039305 8735003 4e-06	3.080194 8690892 8e-05	3.41 1115 8963 2795	Down
ALTEMEIER_RESPONSE_TO_LPS_WITH_MECHANICAL_ VENTILATION	-0.217659724	0.0009132 031752633 97	-4.6143 05925	6.028905 9490152 e-06	3.078046 0400821 6e-05	3.41 2749 4259 7495	Down
SANA_TNF_SIGNALING_UP	-0.184775302	-0.0099439 21	-4.6164 6817	5.970897 6065344 5e-06	3.052830 4319406 4e-05	3.42 1913 0722 5543	Down
NAKAYAMA_SOFT_TISSUE_TUMORS_PCA1_UP	-0.172716294	-0.0016531 15	-4.6174 56192	5.944570 2165134 7e-06	3.044385 0935560 3e-05	3.42 6101 5585 8931	Down
WP_THE_HUMAN_IMMUNE_RESPONSE_TO_TUBERCUL OSIS	-0.284550964	-0.0109424 46	-4.6188 94133	5.906453 5387018 4e-06	3.028756 0505607 2e-05	3.43 2198 7366 2769	Down

MIKKELSEN_NPC_ICP_WITH_H3K4ME3	-0.0568925	-0.00097407	-4.619326072	5.895049799174e-06	3.0265156413129e-05	3.43403056533136	Down
REACTOME_APC_C_CDC20_MEDIATED_DEGRADATION_OF_CYCLIN_B	-0.190211384	-0.013432763	-4.619986405	5.8776570696843 5e-06	3.0200842244644 7e-05	3.436831 2894328	Down
MISSIAGLIA_REGULATED_BY_METHYLATION_UP	-0.141794887	-0.000431014	-4.626963539	5.6968757299775 8e-06	2.9320487276924 4e-05	3.46644486204628	Down
REACTOME_TRANSCRIPTIONAL_REGULATION_BY_TP53	-0.116354577	0.00480290068294073	-4.627029094	5.6952027977972 6e-06	2.9320487276924 4e-05	3.46672328519835	Down
HOLLEMAN_ASPARAGINASE_RESISTANCE_B_ALL_DN	-0.218069437	0.0116129345853236	-4.627044252	5.6948160418450 8e-06	2.9320487276924 4e-05	3.46678766399032	Down
ANDERSEN_LIVER_CANCER_KRT19_UP	-0.218567664	0.00146240233268404	-4.628963416	5.6460524235656 2e-06	2.9131377716601 7e-05	3.47494013564755	Down
ZHANG_PROLIFERATING_VS_QUIESCENT	-0.108098541	0.0011468510434050	-4.631013753	5.5943998868728	2.8888885272728	3.483653	Down

		6		6e-06	7e-05	0166	
MULLIGHAN_NPM1_MUTATED_SIGNATURE_1_UP	-0.077558413	0.0037120601311022 7	-4.633326707	5.5366773439395 7e-06	2.8614618046488 7e-05	1464 3.49 3485 8393 1187	Down
REACTOME_THE_ROLE_OF_GTSE1_IN_G2_M_PROGRESSION_AFTER_G2_CHECKPOINT	-0.170413817	-0.002332838	-4.634743183	5.5016109420427 6e-06	2.8457082597716 2e-05	3.49 9509 6312 9547	Down
DAUER_STAT3_TARGETS_DN	-0.310079534	-0.011674382	-4.639808715	5.3779489833841 2e-06	2.7863880918084 5e-05	3.52 1064 4832 4384	Down
REACTOME_TCR_SIGNALING	-0.144234974	0.0051779984751095 9	-4.640086896	5.3712359829647 6e-06	2.7852348994371 2e-05	3.52 2248 7820 053	Down
REACTOME_NICOTINATE_METABOLISM	-0.159583186	0.0025910213268043 7	-4.640184477	5.3688830839413 5e-06	2.7852348994371 2e-05	3.52 2664 2289 6269	Down
IVANOVSKA_MIR106B_TARGETS	-0.135688488	0.0058554665064954 8	-4.641606522	5.3347068542745 4e-06	2.7709226313374 1e-05	3.52 8719 3497 1929	Down

PICCALUGA_ANGIOIMMUNOBLASTIC_LYMPHOMA_DN	-0.198672282	0.0112571504672896	-4.6445501	5.2646275363998 5e-06	2.7391067157111 4e-05	3.5412582472839	Down
SUZUKI_RESPONSE_TO_TSA_AND_DECITABINE_1B	-0.176009042	0.00329686397434759	-4.648132906	5.1805250617072 8e-06	2.7044170780502 2e-05	3.5565292542 1626	Down
REACTOME_CASPASE_ACTIVATION_VIA_DEATH_RECEPTORS_IN_THE_PRESENCE_OF_LIGAND	-0.227797566	0.00193736917356733	-4.650326788	5.1296661632417 5e-06	2.6841879574997 5e-05	3.56588520606247	Down
WP_NAD_BIOSYNTHESIS_II_FROM_TRYPTOPHAN	-0.257236825	0.00337518528900227	-4.657080173	4.9761076709939 1e-06	2.6086740129948 7e-05	3.59470910358225	Down
RASHI_RESPONSE_TO_IONIZING_RADIATION_6	-0.100802243	0.00116319178802557	-4.658818125	4.9373119433209 e-06	2.5971097654400 7e-05	3.60213257069605	Down
SMITH_TERT_TARGETS_DN	-0.104263824	0.00251726883534498	-4.660780888	4.8938479965384 9e-06	2.5786175309435 e-05	3.61051913469965	Down
KIM_LRRC3B_TARGETS	-0.224456404	-0.006657856	-4.667438571	4.7491459511773	2.5130391235258	3.638988	Down

				7e-06	3e-05	75600444	
WP_HOSTPATHOGEN_INTERACTION_OF_HUMAN_CORONA_VIRUSES_AUTOPHAGY	-0.219298252	3.19677594857613e-05	-4.66916408	4.71232036110568e-06	2.49781148431964e-05	3.646373 0396682	Down
DAZARD_UV_RESPONSE_CLUSTER_G2	-0.169164973	0.00137186803417581	-4.671611728	4.66055433068256e-06	2.47459886488851e-05	3.65685169576636	Down
KAMMINGA_EZH2_TARGETS	-0.260181599	-0.006787032	-4.672357258	4.64489599446557e-06	2.468396355963e-05	3.66004432589654	Down
GRAHAM_NORMAL_QUIESCENT_VS_NORMAL_DIVIDING_DN	-0.236491437	-0.010295669	-4.67271762	4.63734552519192e-06	2.4664956019594e-05	3.66158767762863	Down
KRASNOSELSKAYA_ILF3_TARGETS_UP	-0.259037639	-0.013525201	-4.675652409	4.5762929130995e-06	2.43820172631834e-05	3.67416054601695	Down
REACTOME_MTOR_SIGNALLING	-0.153189609	0.0044043033468315	-4.679982548	4.48762445634894e-06	2.39713296046109e-05	3.69272348045829	Down

HOLLEMAN_PREDNISOLONE_RESISTANCE_B_ALL_DN	-0.214891418	0.0092816241320523 3	-4.680001697	4.4872360504165 7e-06	2.3971329604610 9e-05	3.69280560111936	Down
LIN_NPAS4_TARGETS_UP	-0.106687737	0.0036727855342444	-4.680159106	4.4840444087929 7e-06	2.3971329604610 9e-05	3.69348068010819	Down
KYNG_WERNER_SYNDROM_UP	-0.195144768	0.0073451346623923 3	-4.681477563	4.4573970364837 9e-06	2.3871495604361 4e-05	3.69913588073194	Down
PELLICCIOTTA_HDAC_IN_ANTIGEN_PRESENTATION_DN	-0.213404707	-0.004781464	-4.683592199	4.4149763011203 7e-06	2.3685184011282 8e-05	3.70820892801937	Down
MOSERLE_IFNA_RESPONSE	-0.394000799	-0.038656123	-4.685214026	4.3827053500891 8e-06	2.3552772387881 9e-05	3.71516989485044	Down
WP_HOSTPATHOGEN_INTERACTION_OF_HUMAN_CORONA_VIRUSES_APOPTOSIS	-0.198150389	0.0057321847549677 3	-4.685843436	4.3702427026068 6e-06	2.3506149441144 5e-05	3.71787191100891	Down
REACTOME_E3_UBIQUITIN_LIGASES_UBIQUITINATE_TARGET_PROTEINS	-0.176624656	0.0083321983312003	-4.686611915	4.3550727024888	2.3491399472297	3.721171	Down

		9		2e-06	7e-05	35900772	
SARRIO_EPITHELIAL_MESENCHYMAL_TRANSITION_UP	-0.149395509	-0.006822429	-4.687741702	4.3328624580884 9e-06	2.3406507639125 5e-05	3.72602290899045	Down
RAMALHO_STEMNESS_UP	-0.205343025	0.00357907090006013	-4.689787204	4.2929276665156 2e-06	2.3212697444104 7e-05	3.7348092716813	Down
RIZ_ERYTHROID_DIFFERENTIATION_CCNE1	-0.11667238	0.00457870072138375	-4.692067276	4.2488312198872 7e-06	2.3012648675253 3e-05	3.74460706290576	Down
NAGASHIMA_NRG1_SIGNALING_DN	-0.151867034	0.00346171077371667	-4.695837768	4.1768659493302 5e-06	2.2682245798331 5e-05	3.76081829530457	Down
ONKEN_UVEAL_MELANOMA_UP	-0.115586238	0.00515587402516782	-4.696090817	4.1720784243819 7e-06	2.2676086497494 7e-05	3.76190667736904	Down
LIANG_HEMATOPOIESIS_STEM_CELL_NUMBER_SMALL_VS_HUGE_DN	-0.125664088	0.00373693563593165	-4.697673705	4.1422509799885 5e-06	2.2533700116379 4e-05	3.76871591793379	Down

XU_RESPONSE_TO_TRETINOIN_AND_NSC682994_UP	-0.199933688	0.0030494610494123 7	-4.701314922	4.0744148991097 1e-06	2.2184116911205 2e-05	3.78438705353163	Down
MULLIGHAN_NPM1_MUTATED_SIGNATURE_2_UP	-0.113459463	0.0036218702094183 9	-4.704624245	4.0136915021222 4e-06	2.1930442916965 5e-05	3.79863873348282	Down
ROETH_TERT_TARGETS_UP	-0.349781252	-0.012677772	-4.705844037	3.9915300084692 4e-06	2.1847819014610 7e-05	3.80389394966566	Down
CHEOK_RESPONSE_TO_MERCAPTOPURINE_UP	-0.285683756	-0.000135307	-4.706095332	3.9869790917848 4e-06	2.1842170540784 2e-05	3.80497674379213	Down
WP_MIRNA_REGULATION_OF_DNA_DAMAGE_RESPONSE	-0.136250094	0.0033972323318015 9	-4.706579715	3.9782210757329 2e-06	2.1813443654659 2e-05	3.80706402216022	Down
REACTOME_RAS_SIGNALING_DOWNSTREAM_OF_NF1_LOSS_OF_FUNCTION_VARIANTS	-0.254139025	0.0100213192012881	-4.709493743	3.9259231726543 9e-06	2.1564783303244 1e-05	3.81962484883021	Down
REACTOME_EUKARYOTIC_TRANSLATION_ELONGATION	-0.335011721	-0.00269896	-4.712162294	3.8786124559288	2.1323779906067	3.831133	Down

				4e-06	6e-05	3510197	
PLASARI_TGFB1_TARGETS_10HR_DN	-0.091946043	0.00197397292576233	-4.714351204	3.8402158999146 3e-06	2.1131400789689 8e-05	3.84057747367245	Down
WANG_RESPONSE_TO_FORSKOLIN_UP	-0.219903291	0.00254820846413009	-4.715140268	3.8264646595802 4e-06	2.1074415387768 e-05	3.84398282552446	Down
FULCHER_INFLAMMATORY_RESPONSE_LECTIN_VS_LPS_DN	-0.159945237	0.000190927925485649	-4.719156442	3.7572058303987 1e-06	2.0766675502479 8e-05	3.86132288961826	Down
INAMURA_LUNG_CANCER_SCC_SUBTYPES_UP	-0.205957717	0.00552938808553068	-4.723084621	3.6906341573783 9e-06	2.047164094267e-05	3.8782951580365	Down
CAFFAREL_RESPONSE_TO_THC_24HR_3_DN	-0.212477011	-0.002501052	-4.728542223	3.6000272500630 9e-06	2.0004806751245 8e-05	3.90189542260334	Down
MORI_MATURE_B_LYMPHOCYTE_DN	-0.143297893	-0.00189478	-4.729874561	3.5782356215209 3e-06	1.9919379823121 4e-05	3.90766035382143	Down

FLORIO_NEOCORTEX_BASAL_RADIAL_GLIA_DN	-0.178597195	-0.011142547	-4.730840166	3.5625217717350 3e-06	1.9849706137485 9e-05	3.91183932171628	Down
SAMOLS_TARGETS_OF_KHSV_MIRNAS_UP	-0.265648742	0.00585074334693855	-4.730984498	3.5601787036455 2e-06	1.9849706137485 9e-05	3.91246402516241	Down
HUANG_FOXA2_TARGETS_UP	-0.128346899	0.00227901340741627	-4.735433372	3.4886802202624 9e-06	1.9525913550197 7e-05	3.93172781045348	Down
REACTOME_SRP_DEPENDENT_COTRANSLATIONAL_PROTEIN_TARGETING_TO_MEMBRANE	-0.319812288	-0.00347438	-4.736616652	3.4698978540485 8e-06	1.9438317671551 9e-05	3.93685403970859	Down
POS_RESPONSE_TO_HISTAMINE_DN	-0.207736366	0.00192499389717479	-4.738134691	3.4459445284953 5e-06	1.9356540894453 1e-05	3.94343210944812	Down
REACTOME_PCP_CE_PATHWAY	-0.156896197	0.00214018500774468	-4.740521374	3.4086067682939 1e-06	1.9181525123119 e-05	3.95377786691923	Down
GARGALOVIC_RESPONSE_TO_OXIDIZED_PHOSPHOLIPIDS_GREY_UP	-0.174838869	0.0149829009300493	-4.742292379	3.3811532105560	1.9061596710192	3.961457	Down

				7e-06	1e-05	6493 2857	
GUTIERREZ_CHRONIC_LYMPHOCYTIC_LEUKEMIA_UP	-0.250280839	0.0063017424998001 1	-4.744940648	3.3404979636770 8e-06	1.8849518964130 6e-05	3.97 2946 1319 4964	Down
REACTOME_ALPHA_PROTEIN_KINASE_1_SIGNALING_PATHWAY	-0.21430033	0.0022292756923201 3	-4.748838285	3.2815190667842 7e-06	1.8550445216329 7e-05	3.98 9864 3992 9445	Down
CHEOK_RESPONSE_TO_MERCAPTOPURINE_AND_LD_MTX_DN	-0.191424392	0.0025123073793650 3	-4.749382376	3.2733661819604 6e-06	1.8538123988529 7e-05	3.99 2227 0467 2804	Down
REACTOME_POLYMERASE_SWITCHING	-0.260227133	-0.00051491	-4.752991381	3.2197797937150 8e-06	1.8267982796699 7e-05	4.00 7904 4863 7553	Down
HECKER_IFNB1_TARGETS	-0.291605918	-0.02208945	-4.753325715	3.2148586408406 e-06	1.8256749847847 8e-05	4.00 9357 3389 0193	Down
KEGG_RIBOSOME	-0.3419213	-0.003769767	-4.758977525	3.1327577424347 3e-06	1.7852615162660 4e-05	4.03 3930 4175 1126	Down

ROSTY_CERVICAL_CANCER_PROLIFERATION_CLUSTER	-0.235959426	-0.016583391	-4.761690256	3.0940731146162 8e-06	1.7651573366197 8e-05	4.04573368017421	Down
WUNDER_INFLAMMATORY_RESPONSE_AND_CHOLESTEROL_UP	-0.190635609	-0.008280389	-4.763881812	3.0631574805660 3e-06	1.7507383500804 2e-05	4.05527343752081	Down
ZWANG_EGF_PERSISTENTLY_UP	-0.145260338	0.00761750077986021	-4.769360556	2.9871682327972 4e-06	1.7152038132259 4e-05	4.07913845979813	Down
REACTOME_DEUBIQUITINATION	-0.113384013	0.00309858916867979	-4.772742932	2.9411665066614 3e-06	1.6903537506340 3e-05	4.09388343975645	Down
JIANG_AGING_HYPOTHALAMUS_UP	-0.235505829	0.00728651679824747	-4.774718907	2.9146094263552 8e-06	1.6766432538820 4e-05	4.10250151093357	Down
PID_P53_DOWNSTREAM_PATHWAY	-0.100227019	0.00362225817545426	-4.777155017	2.8821864111447 7e-06	1.6595297823725 e-05	4.113130580713 7	Down
REACTOME_BIOSYNTHESIS_OF_THE_N_GLYCAN_PRECURSOR_DOLICHOL_LIPID_LINKED_OLIGOSACCHARI	-0.139243339	0.0011599836093970	-4.779258665	2.8544684248516	1.6466250476815	4.122312	Down

DE_LLO_AND_TRANSFER_TO_A_NASCENT_PROTEIN		7		1e-06	9e-05	76849926	
WP_DISORDERS_OF_THE_KREBS_CYCLE	-0.281366478	-0.000973841	-4.78002495	2.8444357842446e-06	1.6423639918887e-05	4.12565836328721	Down
TAKEDA_TARGETS_OF_NUP98_HOXA9_FUSION_10D_DN	-0.130209761	0.00366977804610839	-4.782010559	2.81859705354361e-06	1.63047827691941e-05	4.13432963659275	Down
TOOKER_GEMCITABINE_RESISTANCE_DN	-0.148213597	0.00865076083594077	-4.784661662	2.78445103637697e-06	1.61524183016746e-05	4.14591190465574	Down
LY_AGING_OLD_DN	-0.207685561	-0.001216023	-4.785990636	2.76748446613832e-06	1.6069014107877e-05	4.15172003280056	Down
KEGG_P53_SIGNALING_PATHWAY	-0.107965302	0.00139111663146321	-4.787325589	2.75054199805777e-06	1.59855938033189e-05	4.1575556650679	Down
SMITH_LIVER_CANCER	-0.144588845	0.00294432029080007	-4.78807837	2.74103229370228e-06	1.59452553392784e-05	4.16084699084196	Down

REACTOME_ENDOSOMAL_SORTING_COMPLEX_REQUIRED_FOR_TRANSPORT_ESCRT_	-0.181096401	0.000488740766402313	-4.788604775	2.7344012010923 1e-06	1.5921602490788 e-05	4.16314880968758	Down
WAMUNYOKOLI_OVARIAN_CANCER_LMP_DN	-0.1334201	0.0047716745927529	-4.788655512	2.7337628879287 1e-06	1.5921602490788 e-05	4.16337067961415	Down
PID_FAS_PATHWAY	-0.163085081	0.00941466297781719	-4.792470835	2.6861727243539 2e-06	1.5684923894698 7e-05	4.18006053849629	Down
BROWNE_HCMV_INFECTION_48HR_UP	-0.092136056	0.00309376064595081	-4.793924124	2.6682562855937 5e-06	1.5609676498285 e-05	4.18642080424672	Down
IVANOVA_HEMATOPOIESIS_MATURE_CELL	-0.143860388	0.00286708054768332	-4.797451724	2.6252456668614 8e-06	1.5401606667494 5e-05	4.20186598640105	Down
BILBAN_B_CLL_LPL_DN	-0.225025338	0.0105236610729319	-4.797790127	2.6211550033851 e-06	1.5392156202470 5e-05	4.20334814766667	Down
KEGG_PYRIMIDINE_METABOLISM	-0.171172776	-0.003438394	-4.800353016	2.5903734811424	1.5225803217283	4.214576	Down

				3e-06	2e-05	1437525	
MASSARWEH_TAMOXIFEN_RESISTANCE_UP	-0.088181226	0.00304011263142676	-4.800429818	2.5894564514102 3e-06	1.5225803217283 2e-05	4.21491269287753	Down
HAN_JNK_SINGALING_DN	-0.137545295	1.34231108932398e-06	-4.80168526	2.5745106802045 2e-06	1.5161278740065 9e-05	4.22041469577997	Down
WP_HOSTPATHOGEN_INTERACTION_OF_HUMAN_CORONA_VIRUSES_MAPK_SIGNALING	-0.176943912	0.00569744899737693	-4.808580186	2.4939005124455 3e-06	1.4700513277064 9e-05	4.25065352585761	Down
KOMMAGANI_TP63_GAMMA_TARGETS	-0.211302593	0.00576056801581291	-4.811654814	2.4587447609388 3e-06	1.4534694029664 1e-05	4.26414962056379	Down
WP_TRYPTOPHAN_CATABOLISM_LEADING_TO_NAD_PRODUCTION	-0.206330536	0.00217608247625899	-4.813098421	2.4424038179667 8e-06	1.4465649330267 e-05	4.27048885278709	Down
KRISHNAN_FURIN_TARGETS_UP	-0.258500031	0.0170879889986817	-4.81745162	2.3937598320178 5e-06	1.4231865208175 1e-05	4.28961452635524	Down

GAZDA_DIAMOND_BLACKFAN_ANEMIA_PROGENITOR_DN	-0.200565707	0.00014480217433044	-4.820602482	2.3591359142099 7e-06	1.4039459846118 2e-05	4.303466859972 05	Down
DER_IFN_ALPHA_RESPONSE_UP	-0.241517543	-0.010511135	-4.823507666	2.3276403913239 4e-06	1.3865320450045 8e-05	4.316245873577 8	Down
WP_DNA_DAMAGE_RESPONSE	-0.139675424	0.0035503635704638	-4.828133158	2.2783306865240 3e-06	1.3597690933898 7e-05	4.336605386699 89	Down
REACTOME_REGULATION_OF_MRNA_STABILITY_BY_PROTEINS_THAT_BIND_AU_RICH_ELEMENTS	-0.186170593	0.00424785361166199	-4.828506453	2.2743954755153 2e-06	1.3587269217058 3e-05	4.338249197051 17	Down
CEBALLOS_TARGETS_OF_TP53_AND_MYC_UP	-0.193977839	0.00789788721097381	-4.82898614	2.2693483437493 3e-06	1.3570178390801 6e-05	4.340361658223 71	Down
DUTTA_APOPTOSIS_VIA_NFKB	-0.17151056	-0.000418087	-4.830673336	2.2516818674643 3e-06	1.3477521071698 3e-05	4.347793204730 55	Down
BROWNE_HCMV_INFECTION_12HR_UP	-0.1153763	0.0042556878166107	-4.835022909	2.2067480935999	1.3259714827661	4.366961	Down

				e-06	8e-05	7340 7567	
REACTOME_RNA_POLYMERASE_I_PROMOTER_ESCAPE	-0.244647403	-0.003323791	-4.843203786	2.1245688706603 6e-06	1.2778293585454 3e-05	4.40305415324305	Down
KOBAYASHI_EGFR_SIGNALING_24HR_DN	-0.190707322	-0.011796946	-4.843292922	2.1236899596012 9e-06	1.2778293585454 3e-05	4.40344768630207	Down
TOMLINS_METASTASIS_DN	-0.192986635	0.00792923749609265	-4.84443323	2.1124770592035 1e-06	1.2742609432921 5e-05	4.40848265686894	Down
BOYAULT_LIVER_CANCER_SUBCLASS_G1_DN	-0.155064628	0.000952639861641342	-4.845445279	2.1025732521426 1e-06	1.2707567844254 3e-05	4.41295214562616	Down
RUAN_RESPONSE_TO_TNF_TROGLITAZONE_UP	-0.228632613	-0.002925842	-4.846365398	2.0936079896525 3e-06	1.2665716171318 9e-05	4.41701632627667	Down
DITTMER_PTHLH_TARGETS_UP	-0.118471303	0.00613134750566721	-4.848319474	2.0746905548147 1e-06	1.2563516364619 4e-05	4.4256496653418	Down

BROWNE_HCMV_INFECTION_8HR_UP	-0.132107396	0.00488612199466292	-4.852616878	2.0336662209006 5e-06	1.2339165428279 9e-05	4.44464641739862	Down
REACTOME_VITAMIN_C_ASCORBATE_METABOLISM	-0.221387405	0.000621041545150868	-4.860771327	1.9579646118500 8e-06	1.1926483165606 9e-05	4.48073222098058	Down
REACTOME_MASTL_FACILITATES_MITOTIC_PROGRESSION	-0.275602035	0.0109220206095321	-4.861049887	1.9554272898733 7e-06	1.1922728082754 4e-05	4.48196583003115	Down
LEE_NEURAL_CREST_STEM_CELL_DN	-0.098071836	0.0044692021025478	-4.868700737	1.8869663609371 9e-06	1.1527952955056 3e-05	4.51587102350908	Down
SIMBULAN_UV_RESPONSE_NORMAL_UP	-0.235712779	-0.007952791	-4.873684173	1.8436252911118 4e-06	1.1285386767190 5e-05	4.53797951223862	Down
AMIT_EGF_RESPONSE_120_MCF10A	-0.155530938	0.00538358193549086	-4.874055344	1.8404359735000 9e-06	1.1276985278889 5e-05	4.53962693406988	Down
BIOCARTA_TERC_PATHWAY	-0.306664398	-0.004270499	-4.874652094	1.8353195213960	1.1256747301685	4.542275	Down

				8e-06	2e-05	7957 5078	
REACTOME_SIGNALING_BY_FGFR2_IIIA_TM	-0.216699307	0.0025748 092876712 4	-4.8763 28817	1.821016 9683570 7e-06	1.118007 1535699 6e-05	4.54 9719 9126 8286	Down
JAATINEN_HEMATOPOIETIC_STEM_CELL_UP	-0.132751497	-0.0001544 21	-4.8773 39116	1.812451 0986318 8e-06	1.113849 8979413 9e-05	4.55 4206 3601 1571	Down
YANG_BCL3_TARGETS_UP	-0.101390126	-0.0035907 41	-4.8774 08218	1.811866 6368849 2e-06	1.113849 8979413 9e-05	4.55 4513 2515 3468	Down
OSMAN_BLADDER_CANCER_UP	-0.195985264	0.0086473 688798143 8	-4.8776 55165	1.809779 4613223 5e-06	1.113849 8979413 9e-05	4.55 5610 0046 7316	Down
ZHANG_GATA6_TARGETS_UP	-0.202140953	-0.0011724 83	-4.8791 56221	1.797142 5570438 2e-06	1.107732 2593417 e-05	4.56 2277 5634 0303	Down
YAO_TEMPORAL_RESPONSE_TO_PROGESTERONE_CLUSTER_8	-0.137895976	0.0061419 954095290 7	-4.8801 48418	1.788836 4120108 7e-06	1.104806 7272986 5e-05	4.56 6685 7623 0889	Down

VALK_AML_WITH_11Q23_REARRANGED	-0.166800659	0.000255615440294904	-4.882277302	1.7711395050021 7e-06	1.0970629977131 1e-05	4.57614665040581	Down
PARK_HSC_VS_MULTIPOTENT_PROGENITORS_DN	-0.208551354	0.00365264998424881	-4.885256747	1.7466558551611 8e-06	1.0830662230754 7e-05	4.58939329740924	Down
REACTOME_METABOLISM_OF_AMINO_ACIDS_AND_DERIVATIVES	-0.136186619	2.67897814719363e-05	-4.886666026	1.7351893033974 5e-06	1.0770320006188 e-05	4.59566133337167	Down
ONO_FOXP3_TARGETS_UP	-0.157597099	0.00871618131598141	-4.891937098	1.6929427311069 1e-06	1.0529153839659 9e-05	4.6191188728258	Down
ZEILSTRA_CD44_TARGETS_UP	-0.219512198	0.000120664809438394	-4.892307163	1.6900143737259 3e-06	1.0521483668723 e-05	4.62076654607762	Down
MOREAUX_B_LYMPHOCYTE_MATURATION_BY_TACI_DN	-0.260319448	-0.00275136	-4.892875956	1.6855229932427 3e-06	1.0504057448853 1e-05	4.62329924394245	Down
REACTOME_PROCESSING_AND_ACTIVATION_OF_SUMO	-0.294615258	0.0051368543943868	-4.894940015	1.6693211765204	1.0434518169851	4.632492	Down

		6		1e-06	1e-05	06482831	
REACTOME_TRANSLESION_SYNTHESIS_BY_POLH	-0.228984892	0.00650801493932276	-4.902560694	1.6107962374859e-06	1.0099204288964 6e-05	4.66646089458495	Down
KIM_LIVER_CANCER_POOR_SURVIVAL_UP	-0.170030868	0.00306596601059431	-4.903013683	1.6073805545893 9e-06	1.0087978869905 3e-05	4.66848146840515	Down
BRUINS_UVC_RESPONSE_EARLY_LATE	-0.108140454	0.00485016924097491	-4.908764243	1.5646233483951 1e-06	9.8295719873364 6e-06	4.69414563832834	Down
MOLENAAR_TARGETS_OF_CCND1_AND_CDK4_DN	-0.217926154	-0.00767763	-4.911460911	1.5449534077997 7e-06	9.7256853977821 e-06	4.70618928126756	Down
TAKEDA_TARGETS_OF_NUP98_HOXA9_FUSION_3D_UP	-0.19328314	-0.002234	-4.914104356	1.5259037812770 7e-06	9.6252894007995 9e-06	4.71800060305976	Down
WEI_MYCN_TARGETS_WITH_E_BOX	-0.180658806	0.00156915325610335	-4.914696081	1.5216707977352 8e-06	9.6083526363610 2e-06	4.72064525270799	Down

REACTOME_ZINC_TRANSPORTERS	-0.188901192	0.00292939748398474	-4.917593328	1.5011082767911 3e-06	9.5075296673903 5e-06	4.73359801996135	Down
BIOCARTA_CDC42RAC_PATHWAY	-0.285025219	0.0114077536091623	-4.917656828	1.5006606172790 9e-06	9.5075296673903 5e-06	4.73388198521213	Down
BOYLAN_MULTIPLE_MYELOMA_C_DN	-0.134299037	0.00867729254903123	-4.917944154	1.4986366779512 e-06	9.5075296673903 5e-06	4.73516689815191	Down
FARMER_BREAST_CANCER_CLUSTER_2	-0.29047327	-0.014898451	-4.919571731	1.4872216314612 e-06	9.4485001703988 6e-06	4.7424465716738	Down
WP_OMEGA3OMEGA6_FA_SYNTHESIS	-0.25547545	0.000448564055431337	-4.92029911	1.4821473597619 1e-06	9.4259105143874 9e-06	4.74570057650181	Down
IIZUKA_LIVER_CANCER_PROGRESSION_L1_G1_DN	-0.215734464	-0.005721541	-4.922278183	1.4684257119351 2e-06	9.3482239938269 6e-06	4.75455620111275	Down
LI_INDUCED_T_TO_NATURAL_KILLER_UP	-0.120460423	0.0008845430738223	-4.929798436	1.4173962698603	9.0419102230452	4.788233	Down

		05		2e-06	1e-06	7586 2662	
REACTOME_PREGNENOLONE_BIOSYNTHESIS	-0.207688139	0.0050336621569288 9	-4.931539956	1.4058258347943 9e-06	8.9773260870049 4e-06	4.79603885378923	Down
SHEPARD_BMYB_MORPHOLINO_UP	-0.083662794	0.0029710884334805	-4.938966946	1.3574988332022 7e-06	8.7316013033020 9e-06	4.82935080653371	Down
ZHENG_FOXP3_TARGETS_IN_T_LYMPHOCYTE_UP	-0.226761502	0.0033715323554916	-4.948988468	1.2948287818273 7e-06	8.3631657115530 3e-06	4.87436632994776	Down
AMUNDSON_RESPONSE_TO_ARSENITE	-0.109782255	0.0061869246372312 3	-4.94963967	1.2908547490168 1e-06	8.3461827366117 8e-06	4.87729409281769	Down
FOSTER_TOLERANT_MACROPHAGE_UP	-0.142870042	-0.003381362	-4.953205979	1.2692992601626 e-06	8.2153707068084 3e-06	4.89333369919296	Down
SMIRNOV_RESPONSE_TO_IR_2HR_DN	-0.190126238	0.0110963933193008	-4.955827882	1.2536744185279 1e-06	8.1311986581010 9e-06	4.90513195919823	Down

GARGALOVIC_RESPONSE_TO_OXIDIZED_PHOSPHOLIPIDS_YELLOW_UP	-0.20008949	0.00955798137023363	-4.956419782	1.2501728990868 7e-06	8.1169698584018 9e-06	4.90779616484893	Down
REACTOME_FC_EPSILON_RECEPTOR_FCERI_SIGNALING	-0.125004845	0.00721127617753402	-4.960083264	1.2287102714161 7e-06	7.9859734603980 9e-06	4.92429178660874	Down
LI_WILMS_TUMOR_ANAPLASTIC_UP	-0.254563386	-0.011890353	-4.961391692	1.2211315185145 1e-06	7.9450349427878 1e-06	4.93018573576703	Down
TCGA_GLIOBLASTOMA_COPY_NUMBER_UP	-0.124320031	0.00453723521702858	-4.962174472	1.2166190935315 4e-06	7.9239818610180 9e-06	4.93371246818942	Down
REACTOME_CARGO_CONCENTRATION_IN_THE_ER	-0.178557043	0.00494815514065956	-4.964290679	1.204500566515e-06	7.8532930844102 9e-06	4.9432491395418	Down
NAKAMURA_CANCER_MICROENVIRONMENT_DN	-0.191785992	-0.003806466	-4.966152631	1.1939346631135 6e-06	7.7925893311733 4e-06	4.95164282051337	Down
BOWIE_RESPONSE_TO_EXTRACELLULAR_MATRIX	-0.391491726	-0.029123554	-4.966635733	1.1912079167632	7.7911775967857	4.953821	Down

				8e-06	3e-06	0734<br>3808	
BLANCO_MELO_COVID19_SARS_COV_2_INFECTION_A594_ACE2_EXPRESSING_CELLS_UP	-0.122218236	0.0010045119127755<br>8	-4.968570896	1.1803456452831<br>e-06	7.7446146091672<br>6e-06	4.96254828274911	Down
REACTOME_RESPONSE_OF_EIF2AK4_GCN2_TO_AMINO_ACID_DEFICIENCY	-0.335730675	-0.003566816	-4.969579948	1.1747198004202<br>1e-06	7.7240315690765<br>1e-06	4.96710003540769	Down
KAMIKUBO_MYELOID_CEBPA_NETWORK	-0.276991903	-0.003435347	-4.969691576	1.1740990309906<br>1e-06	7.7240315690765<br>1e-06	4.96760362537601	Down
ZHU_CMV_ALL_UP	-0.167306855	-0.011914824	-4.970459158	1.1698390294286<br>2e-06	7.7082705474134<br>4e-06	4.97106670817648	Down
TUOMISTO_TUMOR_SUPPRESSION_BY_COL13A1_UP	-0.190399941	0.0100368275971009	-4.971403849	1.1646166096172<br>2e-06	7.6820141295367<br>5e-06	4.975329 4648011	Down
REACTOME_CELL_CYCLE_CHECKPOINTS	-0.183798489	-0.002570686	-4.973922644	1.1508022642142<br>2e-06	7.5989677169974<br>8e-06	4.98669839067183	Down

REACTOME_MRNA_CAPPING	-0.232831377	-0.001045446	-4.974523256	1.1475316870260 2e-06	7.5854410877215 e-06	4.98941004293601	Down
WP_EXERCISEINDUCED_CIRCADIAN_REGULATION	-0.186135395	0.0074929917652450 4	-4.975484923	1.1423137302628 3e-06	7.5589992790419 6e-06	4.99375236413634	Down
KOKKINAKIS_METHIONINE_DEPRIVATION_96HR_UP	-0.097316696	0.0043927517038066 6	-4.988023286	1.0763467710792 1e-06	7.1683309099663 6e-06	5.05043223930345	Down
CHANG_IMMORTALIZED_BY_HPV31_UP	-0.115452113	0.0022812720451411 8	-5.000971086	1.0120991130496 3e-06	6.7768060352740 8e-06	5.10908769555349	Down
TAKEDA_TARGETS_OF_NUP98_HOXA9_FUSION_3D_DN	-0.200106635	0.0032425389575998	-5.001663622	1.0087693724008 2e-06	6.7618050696456 9e-06	5.11222855413689	Down
REACTOME_TRANSCRIPTIONAL_REGULATION_BY_RUNX3	-0.136826083	0.0010095191489071 6	-5.002466684	1.0049214939430 6e-06	6.7432948247617 3e-06	5.11587113248656	Down
REACTOME_SELENOAMINO_ACID_METABOLISM	-0.307690663	0.0014749111913716	-5.007671966	9.8032256470270	6.5996335782100	5.139493	Down

		5		3e-07	6e-06	3784	
CAFFAREL_RESPONSE_TO_THC_8HR_3_UP	-0.282940458	0.0027163070711889 8	-5.014391834	9.4942660462598 2e-07	6.4125037376642 8e-06	1016 5.17 0019 2399 5703	Down
NOUZOVA_METHYLATED_IN_APL	-0.107089291	0.0033518849632987 1	-5.014715576	9.4796218223602 6e-07	6.4095874347919 6e-06	5.17 1490 7381 606	Down
NIKOLSKY_BREAST_CANCER_8P12_P11_AMPLICON	-0.114300328	0.0055820901325399 1	-5.016637486	9.3931347758863 e-07	6.3580357201664 4e-06	5.18 0227 9856 4285	Down
GENTILE_UV_RESPONSE_CLUSTER_D7	-0.141341782	0.0044434488276443 3	-5.022181243	9.1479197492333 2e-07	6.2055888397258 2e-06	5.20 5446 2150 8073	Down
SPIELMAN_LYMPHOBLAST_EUROPEAN_VS_ASIAN_2FC_DN	-0.196620256	0.0052405717619469 5	-5.023955085	9.0707750806362 8e-07	6.1599891603401 9e-06	5.21 3520 2043 8871	Down
BERENJENO_TRANSFORMED_BY_RHOA_UP	-0.164796227	-0.000430479	-5.037579955	8.4988643433673 3e-07	5.8033499427151 9e-06	5.27 5615 4490 0834	Down

CHEN_HOXA5_TARGETS_6HR_UP	-0.262352333	0.0025693109753444 2	-5.040836348	8.3674349952601 6e-07	5.7325241739050 6e-06	5.29047710798664	Down
REACTOME_PROCESSING_OF_DNA_DOUBLE_STRAND_ BREAK_ENDS	-0.184076001	0.0026575674444227 1	-5.046305171	8.1511264248717 6e-07	5.5905018474230 9e-06	5.31545387402514	Down
REACTOME_MITOTIC_METAPHASE_AND_ANAPHASE	-0.16283203	-0.001639022	-5.047186312	8.1167849572001 8e-07	5.5731066625377 8e-06	5.31948025580221	Down
DAZARD_RESPONSE_TO_UV_NHEK_UP	-0.087497156	-0.000860332	-5.050909478	7.9732192841846 2e-07	5.4866709641833 7e-06	5.3364997170953	Down
KYNG_WERNER_SYNDROM_DN	-0.182160594	0.0104465660868367	-5.053904907	7.8595031907576 2e-07	5.4204373672258 4e-06	5.35020007642754	Down
WP_NOTCH1_REGULATION_OF_HUMAN_ENDOTHELIA L_CELL_CALCIFICATION	-0.199958272	-0.006198412	-5.054331463	7.8434380408362 3e-07	5.4153748519989 4e-06	5.35215158924328	Down
JIANG_AGING_CEREBRAL_CORTEX_UP	-0.185735696	0.0019328509766544	-5.055721177	7.7913183306748	5.3853800532849	5.358510	Down

		6		2e-07	2e-06	5318 3859	
LIANG_HEMATOPOIESIS_STEM_CELL_NUMBER_QTL	-0.178243997	0.0010464745259966 3	-5.059602508	7.6475234680119 2e-07	5.2918816238517 2e-06	5.37 6278 0989 7185	Down
HEIDENBLAD_AMPLICON_12P11_12_UP	-0.139475447	0.0034212148875543 1	-5.063597115	7.5022169175779 3e-07	5.2029341237325 4e-06	5.39 4576 0073 3779	Down
BIOCARTA_DNAFRAGMENT_PATHWAY	-0.311229802	-0.000252622	-5.06566657	7.4279942431018 e-07	5.1572215063683 3e-06	5.40 4060 1653 4771	Down
LANDEMAINE_LUNG_METASTASIS	-0.198257699	0.0064412861228337 5	-5.068937324	7.3121341763318 e-07	5.0824654907605 2e-06	5.41 9056 3384 9429	Down
BOYAULT_LIVER_CANCER_SUBCLASS_G23_UP	-0.204886899	0.0013855153724320 3	-5.070629116	7.2528945859536 1e-07	5.0469413335217 6e-06	5.42 6816 2271 6628	Down
FARMER_BREAST_CANCER_APOCRINE_VS_LUMINAL	-0.093860258	0.0015034107223411 5	-5.075463714	7.0861578869803 9e-07	4.9364514034216 9e-06	5.44 9003 3153 5392	Down

SYED_ESTRADIOL_RESPONSE	-0.223978932	0.0102267144118045	-5.07801786	6.9995750899501 8e-07	4.8926083990226 1e-06	5.46073195114784	Down
RODRIGUES_THYROID_CARCINOMA_UP	-0.259629494	0.00705065178990609	-5.078365577	6.9878673985093 1e-07	4.8926083990226 1e-06	5.46232904604758	Down
TAKAO_RESPONSE_TO_UVB_RADIATION_UP	-0.174927368	0.00171317339546328	-5.083108854	6.8300426402210 5e-07	4.7957098040556 6e-06	5.48412435287284	Down
RUTELLA_RESPONSE_TO_HGF_VS_CSF2RB_AND_IL4_UP	-0.150443589	0.00412489430677984	-5.087227579	6.6958045776580 9e-07	4.7067790502291 9e-06	5.50306350456337	Down
BRUECKNER_TARGETS_OF_MIRLET7A3_UP	-0.104381771	-0.000492842	-5.087242529	6.6953220127547 5e-07	4.7067790502291 9e-06	5.50313227230066	Down
PETROVA_ENDOTHELIUM_LYMPHATIC_VS_BLOOD_UP	-0.127407191	-0.002189827	-5.100157219	6.2908001331619 9e-07	4.4321221823537 5e-06	5.5626003165885	Down
DACOSTA_UV_RESPONSE_VIA_ERCC3_XPCS_UP	-0.174805646	0.0029562124073777	-5.103523636	6.1893095576318	4.3705397524710	5.578122	Down

		5		e-07	5e-06	0683 2402	
HU_ANGIOGENESIS_DN	-0.259171385	0.0006684239673067 83	-5.110256935	5.9910532745141 7e-07	4.2401901567741 7e-06	5.60 9193 0991 9688	Down
GRAHAM_CML_DIVIDING_VS_NORMAL_QUIESCENT_UP	-0.215530141	-0.010231094	-5.113831418	5.8883208323524 e-07	4.1722382884031 2e-06	5.62 5701 4105 6621	Down
BOYAULT_LIVER_CANCER_SUBCLASS_G123_UP	-0.227585885	0.0081498309399919 2	-5.11450713	5.8690933284343 2e-07	4.1633671188105 e-06	5.62 8823 1772 6536	Down
SESTO_RESPONSE_TO_UV_C1	-0.13640178	0.0047666296117716 5	-5.115540498	5.8398063309735 e-07	4.1473315670883 9e-06	5.63 3597 9546 9478	Down
ABDELMOHSEN_ELAVL4_TARGETS	-0.209189623	0.0145553304691444	-5.116410858	5.8152491131675 6e-07	4.1346221357882 1e-06	5.63 7620 1571 6749	Down
GARGALOVIC_RESPONSE_TO_OXIDIZED_PHOSPHOLIPIDS_TURQUOISE_UP	-0.203722968	0.0093350408011943 3	-5.119799384	5.7205916632046 4e-07	4.0737634087647 7e-06	5.65 3284 9606 9144	Down

HAHTOLA_MYCOSIS_FUNGOIDES_CD4_UP	-0.260309069	0.0138850376012003	-5.120364238	5.7049585306002e-07	4.0701928275213 1e-06	5.65589705704865	Down
FARMER_BREAST_CANCER_CLUSTER_5	-0.215736628	0.00824582684132659	-5.127353515	5.51491395638523e-07	3.94822040683773e-06	5.68823771987424	Down
SMID_BREAST_CANCER_RELAPSE_IN_LIVER_UP	-0.270981059	-0.003353022	-5.127734736	5.50472666267023e-07	3.94547787473373e-06	5.69000274389014	Down
PALOMERO_GSI_SENSITIVITY_UP	-0.278689981	0.00190937195474605	-5.134547968	5.32570451564756e-07	3.83933193131526e-06	5.7215657560048	Down
IIZUKA_LIVER_CANCER_PROGRESSION_G1_G2_UP	-0.263837817	-0.0027969	-5.134872231	5.31732652499575e-07	3.83774950472659e-06	5.72306879791878	Down
WP_RALA_DOWNSTREAM_REGULATED_GENES	-0.200618072	0.00820748117988524	-5.137060171	5.26112962709467e-07	3.81048209981058e-06	5.73321251506332	Down
BIOCARTA_RAB_PATHWAY	-0.302397197	0.0159676275465286	-5.138277875	5.2301029245478	3.7958043733742	5.738859	Down

				1e-07	1e-06	57264803	
ZHU_CMV_24_HR_UP	-0.169298817	-0.008806201	-5.139720696	5.1935699070641 4e-07	3.7836254006041 2e-06	5.74555202519748	Down
ZHAN_MULTIPLE_MYELOMA_MS_DN	-0.126254358	0.00418746247567521	-5.14273389	5.1180702858430 6e-07	3.7330037913311 2e-06	5.75953356777165	Down
OSWALD_HEMATOPOIETIC_STEM_CELL_IN_COLLAGEN_GEL_DN	-0.128308804	0.0033907375169634	-5.145040836	5.0609869564752 3e-07	3.7000643155290 7e-06	5.77024260375863	Down
BLALOCK_ALZHEIMERS_DISEASE_INCIPIENT_DN	-0.124855984	0.00593830784414636	-5.145977737	5.0379808376898 3e-07	3.6875880966439 6e-06	5.77459290769921	Down
GERY_CEBP_TARGETS	-0.122803908	0.00314673944544182	-5.147621877	4.9978529644197 2e-07	3.6668644621930 5e-06	5.78222870071884	Down
PYEON_CANCER_HEAD_AND_NECK_VS_CERVICAL_UP	-0.233026716	0.000766676343698614	-5.149121602	4.9615201467957 1e-07	3.6488335961091 2e-06	5.78919554772474	Down

VANTVEER_BREAST_CANCER_METASTASIS_DN	-0.181269062	-0.002847964	-5.149846079	4.9440605867971 1e-07	3.6403065317022 1e-06	5.7925616461744	Down
LE_EGR2_TARGETS_UP	-0.176729199	-0.005059412	-5.150588692	4.9262258086946 6e-07	3.6358006652280 3e-06	5.7960124099 0217	Down
REACTOME_INTERLEUKIN_12_SIGNALING	-0.154742	0.00505426200499848	-5.151797882	4.8973190026266 4e-07	3.6187689344409 e-06	5.8016321335552	Down
KRIEG_HYPOXIA_VIA_KDM3A	-0.136467207	-0.000638667	-5.154111442	4.8424689005236 7e-07	3.5867785758413 4e-06	5.8123874560 1602	Down
MELLMAN_TUT1_TARGETS_DN	-0.132905272	-0.000389041	-5.161677093	4.6672291651225 e-07	3.4652501708032 7e-06	5.8475865375 8068	Down
IWANAGA_CARCINOGENESIS_BY_KRAS_UP	-0.089297941	0.00469785253312187	-5.162324383	4.6525251756028 9e-07	3.4584699119721 1e-06	5.8506000185 6669	Down
WP_GENES_RELATED_TO_PRIMARY_CILIUM_DEVELOPMENT_BASED_ON_CRISPR	-0.202306875	-0.000820123	-5.164014876	4.6143351213174	3.4341940165488	5.858471	Down

				e-07	1e-06	6269 9003	
DER_IFN_GAMMA_RESPONSE_DN	-0.292521165	0.0087285717816208 7	-5.173552252	4.4045052392503 e-07	3.2859091370224 3e-06	5.90 2921 1471 8157	Down
WINZEN_DEGRADED_VIA_KHSRP	-0.1501934	0.0026696819193581 1	-5.176971434	4.3315581487268 e-07	3.2353768266121 9e-06	5.91 8872 8617 1898	Down
FISCHER_G2_M_CELL_CYCLE	-0.173578151	-0.001043987	-5.183244412	4.2007616077706 2e-07	3.1490491907526 8e-06	5.94 8161 0730 7707	Down
REACTOME_PROCESSING_OF_SMDT1	-0.260325152	-0.001826923	-5.184032084	4.1846112780597 1e-07	3.1407354537988 6e-06	5.95 1840 7283 5274	Down
ICHIBA_GRAFT_VERSUS_HOST_DISEASE_D7_UP	-0.203844757	-0.007574122	-5.186014505	4.1442300162147 1e-07	3.1141931853080 8e-06	5.96 1103 7631 4667	Down
BIOCARTA_RAC1_PATHWAY	-0.226529969	0.0032761786348357 7	-5.186398043	4.1364612374181 2e-07	3.1121230182611 2e-06	5.96 2896 2123 915	Down

RAO_BOUND_BY_SALL4_ISOFORM_B	-0.11153402	0.0030135627742172	-5.187851008	4.1071585369461 1e-07	3.0975860314489 e-06	5.969687572 19049	Down
SHETH_LIVER_CANCER_VS_TXNIP_LOSS_PAM2	-0.096459103	0.000120785252449883	-5.188338673	4.0973687414478 7e-07	3.0939620168086 3e-06	5.971967338 76172	Down
MANN_RESPONSE_TO_AMIFOSTINE_DN	-0.311516567	0.0225263978808556	-5.190430763	4.0556266699449 7e-07	3.0714745041093 4e-06	5.981749554 23092	Down
SEITZ_NEOPLASTIC_TRANSFORMATION_BY_8P_DELETION_UP	-0.218299707	-0.007200512	-5.192415455	4.0164088940637 9e-07	3.0513892295537 2e-06	5.991032594 23395	Down
LIN_MELANOMA_COPY_NUMBER_DN	-0.241663207	0.00398063852452305	-5.193915708	3.9870081196616 1e-07	3.0327646321984 8e-06	5.998051686 76261	Down
REACTOME_SEPARATION_OF_SISTER_CHROMATIDS	-0.169935505	-0.00330479	-5.194335159	3.9788254656665 e-07	3.0302539466738 6e-06	6.000014430 6512	Down
COULOUARN_TEMPORAL_TGFB1_SIGNATURE_UP	-0.124471085	0.0056278116682468	-5.198916552	3.8905057238082	2.9776040724633	6.021460	Down

		4		e-07	2e-06	67756394	
REACTOME_LIPOPHAGY	-0.237391654	-0.00186856	-5.216518347	3.5685365568768 7e-07	2.7549634836486 e-06	6.10400169560449	Down
SENGUPTA_NASOPHARYNGEAL_CARCINOMA_WITH_LMP1_UP	-0.175327909	0.00434595649602329	-5.218002376	3.5426050322873 2e-07	2.7383498674230 9e-06	6.11097127982364	Down
GENTILE_UV_RESPONSE_CLUSTER_D1	-0.277844123	-0.002463322	-5.230368434	3.3335183706493 3e-07	2.5863935658275 5e-06	6.16911022250993	Down
TAVOR_CEBPA_TARGETS_UP	-0.194415549	0.00844491668788968	-5.233750244	3.2784563855102 2e-07	2.5468559180052 5e-06	6.18502938337011	Down
MCBRYAN_PUBERTAL_BREAST_6_7WK_DN	-0.129948363	-0.00029454	-5.236656696	3.2318402765386 6e-07	2.5264524680699 6e-06	6.19871762298744	Down
DIAZ_CHRONIC_MEYLOGENOUS_LEUKEMIA_DN	-0.141700078	0.00661572544937166	-5.23793404	3.2115572602110 4e-07	2.5137624103568 7e-06	6.2047353747582	Down

JOHNSTONE_PARVB_TARGETS_2_DN	-0.244707497	0.0075280898540801 5	-5.246252204	3.0824628965549 2e-07	2.4280262942787 3e-06	6.24395277731484	Down
CHARAFE_BREAST_CANCER_LUMINAL_VS_BASAL_DN	-0.121249028	0.0034496255882088 6	-5.251986132	2.9964228265860 8e-07	2.3722954699770 2e-06	6.27101596265496	Down
REACTOME_ACTIVATION_OF_CASPASES_THROUGH_APOPTOSOME_MEDIATED_CLEAVAGE	-0.280945755	0.011184083269367	-5.255904888	2.9389662413691 9e-07	2.3297782196907 5e-06	6.28952572526565	Down
REACTOME_RUNX1_REGULATES_TRANSCRIPTION_OF_GENES_INVOLVED_IN_DIFFERENTIATION_OF_KERATINOCYTES	-0.299725563	0.0071830368516237 2	-5.256819941	2.9257046174058 3e-07	2.3222312736877 2e-06	6.29384949174828	Down
FARMER_BREAST_CANCER_APOCRINE_VS_BASAL	-0.100755872	0.0011842795569512 4	-5.2596575	2.8849489956348 5e-07	2.2928141377599 9e-06	6.30726129584167	Down
HAHTOLA_CTCL_PATHOGENESIS	-0.284911875	-0.000515478	-5.261286771	2.8617975877697 4e-07	2.2802538674309 1e-06	6.31496476049878	Down
GARGALOVIC_RESPONSE_TO_OXIDIZED_PHOSPHOLIPIDS_BLACK_DN	-0.373734723	0.0006506187568172	-5.273865335	2.6890321646816	2.1536545349908	6.374503	Down

		57		7e-07	6e-06	8226 8583	
GAL_LEUKEMIC_STEM_CELL_DN	-0.16227328	0.0003049 898335449 9	-5.2757 03962	2.664642 8042065 1e-07	2.136878 2798074 7e-06	6.38 3216 4465 1744	Down
WP_ZINC_HOMEOSTASIS	-0.184146688	-0.0017088 11	-5.2773 5523	2.642921 9451434 5e-07	2.124950 3255836 e-06	6.39 1043 3515 3619	Down
MOREAUX_MULTIPLE_MYELOMA_BY_TACI_DN	-0.274224707	0.0011401 487814880 9	-5.2785 96909	2.626702 2023074 6e-07	2.114648 5823245 7e-06	6.39 6930 1421 7222	Down
KAYO_AGING_MUSCLE_DN	-0.108868533	0.0025387 080709139 8	-5.2837 47524	2.560447 0499126 5e-07	2.069361 3071364 4e-06	6.42 1361 2280 3182	Down
REACTOME_PCNA_DEPENDENT_LONG_PATCH_BASE_ EXCISION_REPAIR	-0.289604816	-0.0021716 07	-5.2889 229	2.495509 2174739 3e-07	2.022144 3489374 3e-06	6.44 5929 3142 5978	Down
REACTOME_ZINC_EFFLUX_AND_COMPARTMENTALIZ ATION_BY_THE_SLC30_FAMILY	-0.264898058	0.0042849 628061770 6	-5.2930 20601	2.445230 7996327 3e-07	1.986589 9965079 e-06	6.46 5395 4481 5113	Down

HOWLIN_CITED1_TARGETS_2_UP	-0.202497194	0.0035106570512898 5	-5.2984385	2.3802618656127 5e-07	1.9363414678713 4e-06	6.49115202274062	Down
WINNEPENNINCKX_MELANOMA_METASTASIS_UP	-0.240634249	-0.005831056	-5.307723531	2.2727987251025 1e-07	1.8562186429883 2e-06	6.53534270828993	Down
NATSUME_RESPONSE_TO_INTERFERON_BETA_DN	-0.168220901	-0.003319667	-5.315269263	2.1889511781316 5e-07	1.7924564594542 4e-06	6.57130180264623	Down
LI_WILMS_TUMOR_VS_FETAL_KIDNEY_1_UP	-0.133336984	0.0046835290154108 7	-5.320562589	2.1319338220553 6e-07	1.7524049685108 6e-06	6.59655185481437	Down
REACTOME_CDC6_ASSOCIATION_WITH_THE_ORC_ORIGIN_COMPLEX	-0.243128233	-0.00209168	-5.32406905	2.0949610935148 7e-07	1.7291786579051 6e-06	6.61328950502804	Down
PANGAS_TUMOR_SUPPRESSION_BY_SMAD1_AND_SMAD5_DN	-0.103668107	-0.000225687	-5.325563209	2.0793964058606 3e-07	1.7186169761886 7e-06	6.62042440451257	Down
REACTOME_E2F_MEDIATED_REGULATION_OF_DNA_REPLICATION	-0.263380711	-0.000886498	-5.325701716	2.0779592828201	1.7186169761886	6.621085	Down

				9e-07	7e-06	8829 4014	
SESTO_RESPONSE_TO_UV_C3	-0.229845849	0.0012379799974675 8	-5.325885807	2.0760506769144 e-07	1.7186169761886 7e-06	6.62 1965 0844 2722	Down
HORIUCHI_WTAP_TARGETS_DN	-0.191315568	-0.001680654	-5.326419524	2.0705268583014 4e-07	1.7181497606252 7e-06	6.62 4514 2098 7555	Down
FLECHNER_BIOPSY_KIDNEY_TRANSPLANT_OK_VS_DONOR_UP	-0.204205175	0.0088864939014170 6	-5.327778414	2.0565271456764 9e-07	1.7088171342990 6e-06	6.63 1005 4308 6659	Down
REACTOME_RELEASE_OF_APOPTOTIC_FACTORS_FROM_THE_MITOCHONDRIA	-0.386765707	-0.006909684	-5.331824141	2.0153891373064 8e-07	1.6791302517129 3e-06	6.65 0339 2470 9125	Down
KEGG_PEROXISOME	-0.146598323	0.0011471735687323 3	-5.333801132	1.9955785178974 5e-07	1.6671003850053 2e-06	6.65 9791 2721 8655	Down
LI_CYTIDINE_ANALOG_PATHWAY	-0.184670423	0.0091672520747095 8	-5.335726512	1.9764670097999 2e-07	1.6555911916097 3e-06	6.66 8999 2788 316	Down

TONKS_TARGETS_OF_RUNX1_RUNX1T1_FUSION_HSC_UP	-0.124097574	0.00318540968868795	-5.341435137	1.9208421923090 8e-07	1.6177296455444 3e-06	6.69631624825748	Down
THUM_SYSTOLIC_HEART_FAILURE_UP	-0.137163714	0.0059077970569071	-5.347544358	1.8629994452335 9e-07	1.5732840213013 5e-06	6.72557639477641	Down
NIKOLSKY_BREAST_CANCER_14Q22_AMPLICON	-0.295943364	0.0049584528778168	-5.349396784	1.8457974679460 1e-07	1.5608807743243 7e-06	6.73445395361135	Down
REACTOME_HDR_THROUGH_SINGLE_STRAND_ANNEALING_SSA_	-0.221604335	0.00461720992285268	-5.352392875	1.8183014370380 5e-07	1.5418301939474 3e-06	6.7488176857491	Down
FISCHER_DIRECT_P53_TARGETS_META_ANALYSIS	-0.062669402	0.00380556879647596	-5.356595151	1.7804056460709 1e-07	1.5117616751247 8e-06	6.76897503660912	Down
MARSON_BOUND_BY_FOXP3_STIMULATED	-0.132464021	0.00500294999795683	-5.358286818	1.7653680230057 9e-07	1.5031055306991 7e-06	6.77709319048549	Down
REACTOME_MODULATION_BY_MTB_OF_HOST_IMMUNE_SYSTEM	-0.257147919	-0.004591768	-5.358703782	1.7616805371514	1.5020262491894	6.779094	Down

				3e-07	1e-06	4823 1251	
HOFMANN_CELL_LYMPHOMA_UP	-0.200819524	0.0067147937949820 5	-5.360121811	1.7491959188281 3e-07	1.4934331593075 9e-06	6.78 5901 5036 0785	Down
CHICAS_RB1_TARGETS_SENESCENT	-0.112938536	0.0026689738748362 5	-5.362905208	1.7249399110885 9e-07	1.4747523454720 2e-06	6.79 9266 9906 1922	Down
CHOI_ATL_STAGE_PREDICTOR	-0.257761838	0.0068734211518845 6	-5.36495843	1.7072569021201 3e-07	1.4616473919254 7e-06	6.80 9129 8790 6345	Down
JOHANSSON_GLIOMAGENESIS_BY_PDGFB_UP	-0.134731412	0.0023594664483192	-5.366255686	1.6961754818717 3e-07	1.4581940742351 6e-06	6.81 5362 9748 7963	Down
HOWLIN_CITED1_TARGETS_1_DN	-0.194589564	0.0032684598090724 7	-5.368415473	1.6778810737416 7e-07	1.4444671046760 8e-06	6.82 5743 0893 5686	Down
SA_FAS_SIGNALING	-0.271091847	0.0012569791904076 5	-5.379562405	1.5864691721123 1e-07	1.3733910950210 7e-06	6.87 9369 8807 3757	Down

VERNELL_RETINOBLASTOMA_PATHWAY_UP	-0.233848373	-0.001419576	-5.379604017	1.5861371698506 1e-07	1.3733910950210 7e-06	6.87957024145 77	Down
REACTOME_SIGNALING_BY_HEDGEHOG	-0.090253045	-0.000858215	-5.390277952	1.5031692125796 e-07	1.3104173177642 6e-06	6.93100608887 037	Down
REACTOME_CLEC7A_DECTIN_1_SIGNALING	-0.158271032	0.00122459893424744	-5.39937441	1.4358063781051 5e-07	1.2534529098310 4e-06	6.97490534423 085	Down
FRASOR_RESPONSE_TO_SERM_OR_FULVESTRANT_UP	-0.27704474	0.00930434718433265	-5.401042612	1.4237753659869 1e-07	1.2447005206592 6e-06	6.98296252574 582	Down
MALONEY_RESPONSE_TO_17AAG_DN	-0.273524935	-0.002859867	-5.403290209	1.4077206925752 e-07	1.2327024103905 3e-06	6.99382127734 886	Down
HERNANDEZ_MITOTIC_ARREST_BY_DOCETAXEL_1_DN	-0.176173469	0.000117258519607656	-5.405674973	1.3908787372166 9e-07	1.2228306404963 2e-06	7.00534670728 135	Down
SAKAI_CHRONIC_HEPATITIS_VS_LIVER_CANCER_UP	-0.256618247	0.012828853201209	-5.411857647	1.3481221239306	1.1902978695928	7.035246	Down

				9e-07	6e-06	33027829	
CHIARADONNA_NEOPLASTIC_TRANSFORMATION_KRAS_UP	-0.156317473	0.00434487044496194	-5.416045001	1.31989320282916e-07	1.16869858915273e-06	7.05551217125804	Down
GOLDRATH_HOMEOSTATIC_PROLIFERATION	-0.218060583	0.00290447856471525	-5.418739424	1.30203370735162e-07	1.15453188879021e-06	7.06855924508946	Down
WP_CYTOSOLIC_DNASENSING_PATHWAY	-0.14330632	-0.006762383	-5.425384447	1.25898708469756e-07	1.11795891769925e-06	7.10075847188889	Down
BRUINS_UVC_RESPONSE_MIDDLE	-0.133293081	0.00257490569792512	-5.428394322	1.23994759403197e-07	1.10421158051025e-06	7.11535362977535	Down
MOOTHA_PGC	-0.122676644	-0.000301734	-5.430515911	1.22669570631104e-07	1.09397992084377e-06	7.12564532467504	Down
REACTOME_DUAL_INCISION_IN_TC_NER	-0.231195071	-0.000519554	-5.430975653	1.22384227879804e-07	1.09300561503589e-06	7.1278759294974	Down

REACTOME_DOWNSTREAM_SIGNALING_EVENTS_OF_B_CELL_RECEPTOR_BCR_	-0.210761448	0.00145393574790574	-5.43443579	1.2025728168171 5e-07	1.0771095922054 9e-06	7.14466892693565	Down
YIH_RESPONSE_TO_ARSENITE_C2	-0.292245556	0.0130220831789717	-5.437260592	1.1854757627720 9e-07	1.0633306444402 3e-06	7.15838485199263	Down
WP_NOVEL_INTRACELLULAR_COMPONENTS_OF_RIGILIKE_RECEPTOR_RLR_PATHWAY	-0.15580974	0.00218945428605179	-5.44052618	1.1660054518368 3e-07	1.0488979477610 5e-06	7.17424817859772	Down
IGARASHI_ATF4_TARGETS_UP	-0.360682591	0.0091934564235522	-5.441019548	1.1630910431372 5e-07	1.0477947902399 e-06	7.17664548990381	Down
REACTOME_PYRIMIDINE_SALVAGE	-0.246412167	0.00319540900080587	-5.441978866	1.1574444588575 5e-07	1.0442235110652 4e-06	7.18130738912892	Down
BLANCO_MELO_COVID19_SARS_COV_2_POS_PATIENT_LUNG_TISSUE_UP	-0.28080005	-0.001026579	-5.447463859	1.1256681296547 5e-07	1.0185163383042 4e-06	7.20797493275491	Down
DE_YY1_TARGETS_DN	-0.214289529	0.0072900838744844	-5.451506503	1.1027927501811	1.0022012592056	7.227643	Down

		3		8e-07	5e-06	7384	
ACEVEDO_NORMAL_TISSUE_ADJACENT_TO_LIVER_TUMOR_UP	-0.156422621	0.00426864036367366	-5.452241862	1.0986806130843 4e-07	9.9992823539802 1e-07	7042 7.23 1222 7619 3006	Down
WANG_CISPLATIN_RESPONSE_AND_XPC_DN	-0.114037023	0.00252047060994338	-5.453319552	1.0926810954184 7e-07	9.9592827595630 4e-07	7.23 6468 6280 5201	Down
PID_FANCONI_PATHWAY	-0.222713183	0.00574299683390989	-5.460916192	1.0512855356779 1e-07	9.5960725293423 1e-07	7.27 3470 3965 9982	Down
BECKER_TAMOXIFEN_RESISTANCE_UP	-0.205029286	-0.00673577	-5.464616459	1.0316787422560 3e-07	9.4448819368483 1e-07	7.29 1508 6682 546	Down
GENTLES_LEUKEMIC_STEM_CELL_UP	-0.212143886	0.000901834303695131	-5.466051816	1.0241693247200 4e-07	9.4038742581912 5e-07	7.29 8508 4687 2378	Down
REACTOME_TRANSCRIPTION_OF_THE_HIV_GENOME	-0.217465095	0.0047782730082404	-5.470851742	9.9944168789568 3e-08	9.2040572058879 9e-07	7.32 1926 9755 7993	Down

REACTOME_FORMATION_OF_THE_EARLY_ELONGATION_COMPLEX	-0.233941574	0.00202224614754212	-5.470980448	9.9878670151559 3e-08	9.2040572058879 9e-07	7.32255514939759	Down
REACTOME_AMINO_ACIDS_REGULATE_MTORC1	-0.147527734	0.00304546455921885	-5.471422522	9.9654015986206 5e-08	9.2040572058879 9e-07	7.32471286655488	Down
BHATTACHARYA_EMBRYONIC_STEM_CELL	-0.22344636	-0.005545947	-5.472415052	9.9151419626419 3e-08	9.1855650988236 5e-07	7.32955781339203	Down
MARKEY_RB1_ACUTE_LOF_DN	-0.178563369	0.000362031130012997	-5.480250616	9.5269329755869 5e-08	8.8523462544114 1e-07	7.36783118846653	Down
MARSHALL_VIRAL_INFECTION_RESPONSE_UP	-0.238576707	-0.003046476	-5.482463734	9.4199913346874 7e-08	8.7660998822196 6e-07	7.37864927465113	Down
JOHNSTONE_PARVB_TARGETS_1_DN	-0.188323687	0.00618248334900867	-5.482530497	9.4167834496442 1e-08	8.7660998822196 6e-07	7.37897567818984	Down
REACTOME_TRANSCRIPTION_COUPLED_NUCLEOTIDE_EXCISION_REPAIR_TC_NER_	-0.222734176	-0.00034016	-5.486684907	9.2192470163492	8.6180521431445	7.399292	Down

				7e-08	7e-07	7787 5851	
NUYTTEN_EZH2_TARGETS_DN	-0.160469935	0.0018431939233404 2	-5.491731743	8.984698 0523206 7e-08	8.449700 1228415 7e-07	7.42 3990 8723 5476	Down
REACTOME_RAB_GERANYLGERANYLATION	-0.161599635	0.0003112572750431 1	-5.50553919	8.372295 4046752 5e-08	7.909716 5261521 e-07	7.49 1654 3833 1231	Down
BIOCARTA_HBX_PATHWAY	-0.248370455	-0.002076595	-5.509318341	8.211888 7167650 5e-08	7.769998 9733171 7e-07	7.51 0197 8508 9749	Down
KEGG_TERPENOID_BACKBONE_BIOSYNTHESIS	-0.239522529	-0.002901873	-5.510674617	8.155054 1641837 3e-08	7.728003 2361967 1e-07	7.51 6855 2800 2053	Down
SU_TESTIS	-0.146314904	-0.000270729	-5.512744793	8.069041 3167593 5e-08	7.669914 1582121 4e-07	7.52 7019 5005 5893	Down
FORTSCHEGGER_PHF8_TARGETS_DN	-0.073706369	0.0044107508674028 9	-5.514782255	7.985250 4023188 6e-08	7.601909 3937412 8e-07	7.53 7026 0786 1962	Down

VISALA_AGING_LYMPHOCYTE_DN	-0.185059867	0.00929151505973306	-5.51708939	7.8913917456022 6e-08	7.5240965537562 6e-07	7.54836066763711	Down
KYNG_RESPONSE_TO_H2O2_VIA_ERCC6_UP	-0.225434976	0.000880092539894021	-5.520232097	7.7652666685720 3e-08	7.4266579679239 7e-07	7.56380639259311	Down
CHIBA_RESPONSE_TO_TSA_DN	-0.235169756	0.0131084869086322	-5.523355394	7.6418659976692 3e-08	7.3425792952837 4e-07	7.57916368719319	Down
KRIGE_RESPONSE_TO_TOSEDOSTAT_24HR_UP	-0.159132332	0.00715942455915683	-5.525743755	7.5487928538473 5e-08	7.2643964719117 e-07	7.590911974	Down
WP_HYPERTROPHY_MODEL	-0.197607889	0.00531779135338528	-5.527382523	7.4855704188987 9e-08	7.2147415512585 1e-07	7.59897537937118	Down
GRANDVAUX_IRF3_TARGETS_DN	-0.258439783	-0.001252937	-5.531121394	7.3432515351831 6e-08	7.0996202926607 3e-07	7.61737929636416	Down
WP_NUCLEOTIDE_METABOLISM	-0.240216313	-0.003920257	-5.537433156	7.1089530914659 4e-08	6.8838177595521 2e-07	7.6484703500	Down

						7064	
SCIBETTA_KDM5B_TARGETS_DN	-0.196617218	0.00011767981611384	-5.539950066	7.01756753084051e-08	6.82727925766881e-07	7.66087626931806	Down
MYLLYKANGAS_AMPLIFICATION_HOT_SPOT_16	-0.277909419	0.00272213668104534	-5.548664539	6.70990992157656e-08	6.56746030599778e-07	7.70386489399858	Down
REACTOME_PURINE_CATABOLISM	-0.199674466	0.0023289054635921	-5.561614674	6.27684459343958e-08	6.17438579896663e-07	7.76784760584361	Down
REACTOME_ACTIVATED_NTRK2_SIGNALS_THROUGH_FRS2_AND_FRS3	-0.248720016	0.0087190869191297	-5.562972015	6.23306249091338e-08	6.14105061604752e-07	7.77456069829936	Down
LU_IL4_SIGNALING	-0.149297273	0.00606068869607966	-5.570240702	6.00361966585205e-08	5.95279029807407e-07	7.81053213380752	Down
ZHOU_CELL_CYCLE_GENES_IN_IR_RESPONSE_24HR	-0.254487492	-0.009660468	-5.57412041	5.88454257291639e-08	5.85342239584808e-07	7.82974742445172	Down
REACTOME_CYCLIN_A_CDK2_ASSOCIATED_EVENTS_	-0.213141283	-0.0020602	-5.5769	5.798589	5.777182	7.84	Down

AT_S_PHASE_ENTRY		05	68713	50751015e-08	19472159e-07	386118092701	
SINGH_NFE2L2_TARGETS	-0.236015447	0.00128816099863973	-5.579841012	5.71315133581314e-08	5.70121066581868e-07	7.85809965024688	Down
NELSON_RESPONSE_TO_ANDROGEN_UP	-0.173921979	-0.000371503	-5.602788947	5.07320593342257e-08	5.08713880916864e-07	7.97206566380602	Down
BLUM_RESPONSE_TO_SALIRASIB_UP	-0.116443876	0.00412478058716573	-5.602932072	5.06944266839396e-08	5.08713880916864e-07	7.97277762615836	Down
BURTON_ADIPOGENESIS_11	-0.281430096	0.00981429609295167	-5.606841648	4.96769115284143e-08	5.01373316840435e-07	7.99223112820382	Down
SHEDDEN_LUNG_CANCER_POOR_SURVIVAL_A6	-0.187831569	-0.004586994	-5.614733363	4.76832688735441e-08	4.83611192643935e-07	8.03153199395257	Down
BREDEMEYER_RAG_SIGNALING_VIA_ATM_NOT_VIA_NFKB_UP	-0.143030309	0.0012170367864698	-5.618223466	4.68266542598346e-08	4.75700561359727e-07	8.048926745	Down

WP_DUAL_HIJACK_MODEL_OF_VIF_IN_HIV_INFECTION	-0.39684597	-0.019482952	-5.61920928	4.6587423804576 2e-08	4.7404613041804 e-07	8.0538416182591	Down
KRIGE_RESPONSE_TO_TOSEDOSTAT_6HR_UP	-0.136188238	0.00474583660027046	-5.620302528	4.6323515019324 2e-08	4.7213474174867 9e-07	8.05929291133693	Down
WP_COPPER_HOMEOSTASIS	-0.181404971	-0.001408515	-5.627856204	4.4539467323945 9e-08	4.5469814749955 9e-07	8.0969809815795	Down
ZHAN_V2_LATE_DIFFERENTIATION_GENES	-0.178962192	0.00323695962043382	-5.634731787	4.2973916918474 1e-08	4.4016353516991 6e-07	8.13132065916286	Down
DUTERTRE_ESTRADIOL_RESPONSE_24HR_DN	-0.135775441	0.00301284221369221	-5.635061931	4.2900109494534 9e-08	4.4013385063236 e-07	8.13297037794885	Down
REACTOME_THE_CITRIC_ACID_TCA_CYCLE_AND_RESPIRATORY_ELECTRON_TRANSPORT	-0.283188196	-0.000342167	-5.638944259	4.2041387574119 6e-08	4.3203790177576 2e-07	8.15237599090333	Down
REACTOME_SYNTHESIS_OF_SUBSTRATES_IN_N_GLYCAN_BIOSYTHESIS	-0.143214369	0.0022948994410969	-5.647229567	4.0264443962860	4.1584260179280	8.193825	Down

		5		3e-08	2e-07	03266083	
REACTOME_RNA_POLYMERASE_II_TRANSCRIBES_SNRNA_GENES	-0.222379988	0.0050787048213611	-5.64858726	3.9980329718729 6e-08	4.1359651094025 8e-07	8.20062177848986	Down
BROWNE_HCMV_INFECTION_30MIN_DN	-0.088859762	0.0022506762375381	-5.654515466	3.8762509580345 9e-08	4.0166760762138 1e-07	8.23031408893188	Down
ZHAN_LATE_DIFFERENTIATION_GENES_UP	-0.196527436	0.00576978486003558	-5.656105903	3.8441990537087 9e-08	3.9959301016279 3e-07	8.23828422523395	Down
SPIELMAN_LYMPHOBLAST_EUROPEAN_VS_ASIAN_DN	-0.187610073	0.0116660414647111	-5.656284301	3.8406199792966 2e-08	3.9959301016279 3e-07	8.23917833864892	Down
BILANGES_RAPAMYCIN_SENSITIVE_GENES	-0.184199797	-0.001514249	-5.662280108	3.7222017881488 5e-08	3.8960719222664 3e-07	8.2692417065613	Down
REACTOME_TP53_REGULATES_TRANSCRIPTION_OF_CELL_DEATH_GENES	-0.155369629	0.00327417524190642	-5.662283307	3.7221395833604 3e-08	3.8960719222664 3e-07	8.26925774923825	Down

TIEN_INTESTINE_PROBIOTICS_6HR_UP	-0.259378112	0.0040511951687451 7	-5.6828 49974	3.3423852865950 5e-08	3.5222725762131 5e-07	8.37257144838797	Down
TURASHVILI_BREAST_DUCTAL_CARCINOMA_VS_DUCTAL_NORMAL_UP	-0.188012813	0.0038706226253904 4	-5.6856 06546	3.2944499311448 e-08	3.4776616875196 9e-07	8.38644114530394	Down
TARTE_PLASMA_CELL_VS_PLASMABLAST_DN	-0.217183393	0.0002631617306961 34	-5.6897 55775	3.2235586187189 e-08	3.4086249312416 e-07	8.4073279993797	Down
BIOCARTA_MHC_PATHWAY	-0.351211819	-0.0166340 46	-5.6913 80295	3.1962101986955 4e-08	3.3854738401541 4e-07	8.41550896203587	Down
ZHOU_CELL_CYCLE_GENES_IN_IR_RESPONSE_6HR	-0.265764433	-0.0116413 46	-5.6915 85748	3.1927675954448 1e-08	3.3854738401541 4e-07	8.41654374218228	Down
YAMASHITA_LIVER_CANCER_WITH_EPCAM_UP	-0.237380411	0.0015102304081894 5	-5.6937 54853	3.1566417663097 2e-08	3.3550129184048 7e-07	8.42747037112418	Down
RHEIN_ALL_GLUCOCORTICOID_THERAPY_UP	-0.197287556	0.0057367413692767	-5.6937 55897	3.1566244660283	3.3550129184048	8.427475	Down

		1		7e-08	7e-07	6337 2276	
OUELLET_CULTURED_OVARIAN_CANCER_INVASIVE_VS_LMP_UP	-0.168873094	0.0049184575899331 5	-5.701369607	3.0329491474148 6e-08	3.2513843450784 2e-07	8.46 5854 8320 6587	Down
REACTOME_MITOTIC_G1_PHASE_AND_G1_S_TRANSITION	-0.224825626	-0.0046652 16	-5.712886905	2.8547982574770 4e-08	3.0924490024712 e-07	8.52 3987 9704 404	Down
COLLIS_PRKDC_REGULATORS	-0.272045226	0.0030385625020090 3	-5.716537305	2.8004947639431 2e-08	3.0495914034728 e-07	8.54 2432 4848 4311	Down
HEDVAT_ELF4_TARGETS_UP	-0.286743323	0.0016674499358545 7	-5.723772754	2.6958217321311 4e-08	2.9407672216762 7e-07	8.57 9018 6966 3835	Down
KYNG_DNA_DAMAGE_DN	-0.09784477	0.0030747235019268	-5.724485241	2.6857232617899 e-08	2.9349092052693 5e-07	8.58 2623 3709 1309	Down
REACTOME_REGULATION_OF_EXPRESSION_OF_SLITS_AND_ROBOS	-0.290793701	0.0031907094284182	-5.735423545	2.5352436160295 1e-08	2.7851782521584 3e-07	8.63 8007 5854 6238	Down

REACTOME_SIGNALING_BY_ROBO_RECEPTORS	-0.219321937	0.00401124773311086	-5.735659286	2.5320927938007 5e-08	2.7851782521584 3e-07	8.63920213488137	Down
REACTOME_CARGO_TRAFFICKING_TO_THE_PERICILIARY_MEMBRANE	-0.202884734	0.00362683086797557	-5.738988044	2.4880077594246 e-08	2.7429954107901 4e-07	8.6560737448038	Down
REACTOME_ATF4_ACTIVATES_GENES_IN_RESPONSE_TO_ENDOPLASMIC_RETICULUM_STRESS	-0.237801937	0.000939229332421577	-5.741940829	2.4495294511428 4e-08	2.7053788795807 2e-07	8.67104619070696	Down
REACTOME_SOS_MEDIATED_SIGNALLING	-0.246953575	0.0133351012170878	-5.745152389	2.4083381830073 4e-08	2.6693848396297 4e-07	8.68733764320385	Down
WHITESIDE_CISPLATIN_RESISTANCE_DN	-0.251538987	0.00813673508019974	-5.750163975	2.3454067071736 6e-08	2.6089497188937 1e-07	8.71277445601768	Down
GRAHAM_CML_QUIESCENT_VS_CML_DIVIDING_UP	-0.223761253	0.00253917666445089	-5.753596485	2.3032329433389 7e-08	2.5758859241991 e-07	8.73020654425704	Down
RHODES_UNDIFFERENTIATED_CANCER	-0.278857077	-0.014477399	-5.756531638	2.2677576988406	2.5453837317728	8.745119	Down

				4e-08	5e-07	2570 7816	
PECE_MAMMARY_STEM_CELL_UP	-0.220128926	-0.0005681 1	-5.7636 71464	2.183674 7588417 5e-08	2.455447 3239367 3e-07	8.78 1419 6507 3229	Down
KYNG_NORMAL_AGING_DN	-0.243565616	0.0125449 213809115	-5.7651 01369	2.167204 8446227 7e-08	2.441350 3576358 5e-07	8.78 8693 8265 9484	Down
KIM_WT1_TARGETS_DN	-0.222810336	0.0028477 505982217 2	-5.7651 95687	2.166122 7375019 1e-08	2.441350 3576358 5e-07	8.78 9173 6915 2652	Down
REACTOME_C_TYPE_LECTIN_RECEPTORS_CLRS_	-0.130831827	0.0024911 924823994 1	-5.7681 14729	2.132892 3560022 1e-08	2.418737 4521733 9e-07	8.80 4027 9455 9583	Down
MITSIADES_RESPONSE_TO_APLIDIN_UP	-0.132512075	0.0048636 272407815 4	-5.7689 24543	2.123761 9470040 5e-08	2.414320 5870062 5e-07	8.80 8149 9195 742	Down
WALLACE_PROSTATE_CANCER_RACE_UP	-0.156439695	-0.0032262 66	-5.7708 35118	2.102371 6698662 3e-08	2.394389 1660293 1e-07	8.81 7876 5999 5484	Down

TIEN_INTESTINE_PROBIOTICS_24HR_UP	-0.229147668	0.00261981861343463	-5.773200436	2.0761813577119e-08	2.3689076631098 8e-07	8.82992185713162	Down
REACTOME_AUF1_HNRNP_D0_BINDS_AND_DESTABILIZES_MRNA	-0.277052233	-0.001684336	-5.773448718	2.0734507580940 8e-08	2.3689076631098 8e-07	8.83118644479148	Down
SEMBA_FHIT_TARGETS_UP	-0.234092697	0.000422053344963667	-5.775496814	2.0510591800202 2e-08	2.3488790277464e-07	8.84161973505059	Down
SARTIPY_NORMAL_AT_INSULIN_RESISTANCE_DN	-0.267820809	0.00298346889692839	-5.775901808	2.0466594656599 8e-08	2.3481728841684 9e-07	8.8436831743699	Down
DEURIG_T_CELL_PROLYMPHOCYTIC_LEUKEMIA_DN	-0.239569583	0.00449933005229895	-5.776407739	2.0411761448753 4e-08	2.3462185798594 9e-07	8.84626104640708	Down
DARWICHE_PAPILLOMA_RISK_HIGH_UP	-0.113268536	-0.002086664	-5.781476589	1.9870250710777 1e-08	2.2924655420407 7e-07	8.87209811192345	Down
REACTOME_SIGNALING_BY_FGFR2_IN_DISEASE	-0.155116047	-0.000991138	-5.784274819	1.9577346567388	2.2671005623839	8.886368	Down

				6e-08	7e-07	91713961	
REACTOME_S_PHASE	-0.222997031	-0.004317323	-5.788604235	1.91324525130968e-08	2.22387889042681e-07	8.90845930764139	Down
KAN_RESPONSE_TO_ARSENIC_TRIOXIDE	-0.133437414	-0.00073506	-5.792178856	1.87725734974378e-08	2.18614190804121e-07	8.92670817024938	Down
HOELZEL_NF1_TARGETS_UP	-0.118161021	-0.00154268	-5.794546471	1.85378581257927e-08	2.16286626666908e-07	8.93879996740518	Down
REACTOME_APOPTOSIS_INDUCED_DNA_FRAGMENTATION	-0.310833023	0.00879981144655178	-5.798464436	1.815572602271e-08	2.1302947338934e-07	8.95881813864333	Down
REACTOME_G2_M_CHECKPOINTS	-0.234954535	-0.001024768	-5.798707672	1.8132257619363e-08	2.1302947338934e-07	8.96006126033159	Down
REACTOME_REGULATION_OF_PTEN_STABILITY_AND_ACTIVITY	-0.229265204	0.00126547946370717	-5.801927599	1.78243632270495e-08	2.09935147154262e-07	8.9765213687164	Down

LIAN_LIPA_TARGETS_6M	-0.214051076	-0.002139713	-5.802657629	1.7755269670111 9e-08	2.0951893316042 7e-07	8.980254239 61047	Down
BLANCO_MELO_BETA_INTERFERON_TREATED_BRONCHIAL_EPITHELIAL_CELLS_UP	-0.205906709	-0.007476853	-5.806149715	1.7428362338532 8e-08	2.0697117303589 7e-07	8.998115433 23813	Down
REACTOME_SMAC_XIAP_REGULATED_APOPTOTIC_RESPONSE	-0.283638851	0.0119133736197764	-5.808031239	1.7254669603341 6e-08	2.0556570869086 7e-07	9.007742454 88249	Down
WP_NUCLEOTIDE_EXCISION_REPAIR	-0.262528105	0.00188931238981911	-5.814391557	1.6679939150184 3e-08	1.9910073520229 6e-07	9.040303737 73565	Down
MARCHINI_TRABECTEDIN_RESISTANCE_UP	-0.166272881	0.002287291915676	-5.816810768	1.6466277985342 3e-08	1.9692907024088 5e-07	9.052696042 45828	Down
CHIARADONNA_NEOPLASTIC_TRANSFORMATION_KRAS_CDC25_UP	-0.149161186	0.00279894409265039	-5.832178579	1.5170099930215 3e-08	1.8177762599777 3e-07	9.131510791 74891	Down
MIZUSHIMA_AUTOPHAGOSOME_FORMATION	-0.193870084	0.0057089114806871	-5.83655848	1.4819300406909	1.7791759695491	9.154003	Down

		9		8e-08	1e-07	0339 7005	
HAMAI_APOPTOSIS_VIA_TRAIL_UP	-0.271471403	0.0021092635284176 4	-5.844763275	1.4183348458426 7e-08	1.7061248814235 4e-07	9.19 6172 7227 5601	Down
MACAEVA_PBMC_RESPONSE_TO_IR	-0.171639288	0.0070109278336859 2	-5.851050288	1.3714156759862 5e-08	1.6625736525091 1e-07	9.22 8516 8742 686	Down
FISCHER_G1_S_CELL_CYCLE	-0.17144396	0.0013457779405339 4	-5.851706845	1.3666041987561 1e-08	1.6599828300742 1e-07	9.23 1896 1562 4972	Down
REACTOME_TICAM1_RIP1_MEDIATED_IKK_COMPLEX_RECRUITMENT	-0.208643223	0.0048450555616728 2	-5.854081049	1.3493424323737 8e-08	1.6422291132831 5e-07	9.24 4118 5804 9053	Down
JOSEPH_RESPONSE_TO_SODIUM_BUTYRATE_UP	-0.209944769	-0.005040026	-5.854382796	1.3471638835002 1e-08	1.6422291132831 5e-07	9.24 5672 2483 7742	Down
WU_HBX_TARGETS_2_UP	-0.204676304	0.0022575442910488 5	-5.856213811	1.3340177946555 9e-08	1.6299701676037 9e-07	9.25 5101 3386 5141	Down

REACTOME_SIGNALING_BY_PDGFRA_TRANSMEMBRANE_JUXTAMEMBRANE_AND_KINASE_DOMAIN_MUTANTS	-0.223186637	0.0108228542015117	-5.870342578	1.2367060662978 7e-08	1.5200464462397 8e-07	9.32793655419125	Down
WANG_LMO4_TARGETS_DN	-0.189642009	0.00720000115005473	-5.870573205	1.2351766047904 8e-08	1.5200464462397 8e-07	9.32912659001294	Down
BIOCARTA_MITOCHONDRIA_PATHWAY	-0.235925652	0.01332659691461	-5.876800358	1.1945711235419 5e-08	1.4740960166649 8e-07	9.36127240845818	Down
BOYLAN_MULTIPLE_MYELOMA_C_CLUSTER_DN	-0.225859684	0.00667127471137345	-5.878937177	1.1809403720903 7e-08	1.4601786632599 5e-07	9.37230919407603	Down
SA_PROGRAMMED_CELL_DEATH	-0.249856299	0.00883429348561771	-5.883322673	1.1534395897441 6e-08	1.4318799067084 e-07	9.39497024970763	Down
GARGALOVIC_RESPONSE_TO_OXIDIZED_PHOSPHOLIPIDS_MAGENTA_UP	-0.306251026	0.00575984695806481	-5.884484899	1.1462569516631 5e-08	1.4258150098142 7e-07	9.40097798872325	Down
REACTOME_HOMOLOGOUS_DNA_PAIRING_AND_STRAND_EXCHANGE	-0.24186071	0.0047893193290731	-5.886288251	1.1351985433565	1.4176255128202	9.410301	Down

		3		9e-08	6e-07	62598405	
WANG_RESPONSE_TO_GSK3_INHIBITOR_SB216763_UP	-0.163329171	0.00482436776932925	-5.88944286	1.1161041517949e-08	1.3967053367320 4e-07	9.42661682221132	Down
WARTERS_IR_RESPONSE_5GY	-0.143180256	0.00760344108240917	-5.898333287	1.0639643058530 3e-08	1.3341467568545e-07	9.472633 2874302	Down
OUELLET_OVARIAN_CANCER_INVASIVE_VS_LMP_UP	-0.23906111	0.00592620452200361	-5.90876637	1.0058078579950 8e-08	1.2663386155325 5e-07	9.52670296906182	Down
BOYAULT_LIVER_CANCER_SUBCLASS_G3_UP	-0.259708176	0.00483255834477814	-5.90909121	1.0040479113303 1e-08	1.2663386155325 5e-07	9.52838764885768	Down
SHEN_SMARCA2_TARGETS_UP	-0.333097655	0.00877778402272994	-5.917635772	9.5882013489546 3e-09	1.2145707300604 4e-07	9.57272695071215	Down
LEE_RECENT_THYMIC_EMIGRANT	-0.250492867	0.0029817362983537	-5.921052201	9.4129905502949 4e-09	1.1997214034020 7e-07	9.59046927967194	Down

REACTOME_FORMATION_OF_TC_NER_PRE_INCISION_COMPLEX	-0.237300536	-0.003543781	-5.922932676	9.3178899777010 3e-09	1.1900440965347 8e-07	9.600238412115 83	Down
REACTOME_E2F_ENABLED_INHIBITION_OF_PRE_REPLICATION_COMPLEX_FORMATION	-0.35763001	0.000347671989573157	-5.929749971	8.980929842773e-09	1.1530943460666 2e-07	9.635674573640 94	Down
JINESH_BLEBBISHIELD_TO_IMMUNE_CELL_FUSION_PBSHMS_DN	-0.106027647	0.000499588249908065	-5.934542431	8.7512111285344 6e-09	1.1316409890586 1e-07	9.660604520355 17	Down
WANG_RESPONSE_TO_ANDROGEN_UP	-0.272203949	0.00316511789733703	-5.946402896	8.2071118074304 7e-09	1.0657226566678 e-07	9.722368375431 55	Down
TIEN_INTESTINE_PROBIOTICS_2HR_DN	-0.219283221	0.00506339070239963	-5.946447984	8.2051078651409 6e-09	1.0657226566678 e-07	9.722603353856 87	Down
KEGG_NUCLEOTIDE_EXCISION_REPAIR	-0.267831636	0.00153853268943164	-5.956972306	7.7502044462487 1e-09	1.0148843670435 8e-07	9.777489086916 02	Down
GAZDA_DIAMOND_BLACKFAN_ANEMIA_MYELOID_DN	-0.231723731	-0.000584269	-5.975238228	7.0185167365375	9.2886851564367	9.872925	Down

				e-09	3e-08	6023 0011	
GUTIERREZ_WALDENSTROEMS_MACROGLOBULINEMIA_2	-0.311007768	-0.004089368	-5.976017014	6.9888702890407 3e-09	9.2692132230931 3e-08	9.87699962876159	Down
FAELT_B_CLL_WITH_VH3_21_DN	-0.227994681	0.00980310881925762	-5.977867457	6.9189187561102 e-09	9.1960875201661 7e-08	9.88668140022933	Down
BASAKI_YBX1_TARGETS_DN	-0.139784722	0.00464388731611549	-5.981010874	6.8016554794217 4e-09	9.0596299486632 6e-08	9.90313347233578	Down
WARTERS_RESPONSE_TO_IR_SKIN	-0.130341337	0.00691573717293545	-5.987482103	6.5663356174978 7e-09	8.7992744491916 3e-08	9.9370236217612	Down
WP_MIRNA_REGULATION_OF_P53_PATHWAY_IN_PROSTATE_CANCER	-0.205078052	0.00851346811853117	-5.992399151	6.3928729658575 6e-09	8.5888663417917 5e-08	9.96279326423203	Down
DARWICHE_SQUAMOUS_CELL_CARCINOMA_UP	-0.114813917	-0.000336229	-5.994736221	6.3120052611079 e-09	8.5356463302171 5e-08	9.97504724960038	Down

REACTOME_RECOGNITION_OF_DNA_DAMAGE_BY_PCNA_CONTAINING_REPLICATION_COMPLEX	-0.265907565	0.00246546770370464	-5.999823247	6.1394260570733 2e-09	8.3203968419768 8e-08	10.00173279374 24	Down
REACTOME_NOTCH4_ACTIVATION_AND_TRANSMISSION_OF_SIGNAL_TO_THE_NUCLEUS	-0.322390211	0.00824658644560679	-6.002180075	6.0610423322673 6e-09	8.2389062330331 2e-08	10.01410213665 88	Down
GHANDHI_DIRECT_IRRADIATION_UP	-0.172963585	-0.002684016	-6.00321941	6.0267877203318 6e-09	8.2215981055164 5e-08	10.01955806535 08	Down
LANDIS_ERBB2_BREAST_PRENEOPLASTIC_UP	-0.207802189	0.00240076419437726	-6.009483328	5.8243137282409 7e-09	7.9804669561129 6e-08	10.05245546241 05	Down
ZHENG_FOXP3_TARGETS_DN	-0.297125612	0.00434749227821005	-6.012575601	5.7268275293564 3e-09	7.8642518749370 2e-08	10.06870543197 42	Down
REACTOME_SWITCHING_OF_ORIGINS_TO_A_POST_REPLICATIVE_STATE	-0.264157938	-0.006337564	-6.012992758	5.7137991180644 7e-09	7.8637585644847 3e-08	10.07089809324 13	Down
REACTOME_G1_S_DNA_DAMAGE_CHECKPOINTS	-0.255958071	-0.00264717	-6.018620723	5.5408381237207	7.6426627186521	10.10049	Down

				5e-09	5e-08	1189 0378	
REACTOME_DNA_REPLICATION	-0.261764789	-0.0070830 25	-6.0239 44473	5.381945 1903150 2e-09	7.456637 0080994 1e-08	10.1 2850 4161 7952	Down
BORCZUK_MALIGNANT_MESOTHELIOMA_UP	-0.227940637	0.0069068 166001778 4	-6.0302 46215	5.199617 0641968 7e-09	7.220139 3998814 3e-08	10.1 6168 7720 6193	Down
REACTOME_CYTOCHROME_C_MEDIATED_APOPTOTIC _RESPONSE	-0.239390912	0.0055986 338632820 5	-6.0329 75065	5.122551 0454161 1e-09	7.129074 9638784 3e-08	10.1 7606 5464 0816	Down
BHATI_G2M_ARREST_BY_2METHOXYESTRADIOL_UP	-0.167355474	-0.0002346 43	-6.0337 52806	5.100792 0425516 9e-09	7.114745 2153074 9e-08	10.1 8016 4134 7239	Down
REACTOME_REGULATION_OF_IFNG_SIGNALING	-0.296340505	0.0048270 899810209 3	-6.0357 39021	5.045632 8397580 5e-09	7.053658 3415266 2e-08	10.1 9063 3249 7963	Down
REACTOME_GENE_AND_PROTEIN_EXPRESSION_BY_JA K_STAT_SIGNALING_AFTER_INTERLEUKIN_12_STIMU LATION	-0.194640972	0.0079347 574870856 7	-6.0389 28274	4.958282 4107874 7e-09	6.947191 6306451 e-08	10.2 0744 8957 6389	Down

REACTOME_FBXW7_MUTANTS_AND_NOTCH1_IN_CANCER	-0.272337001	0.00639278722450946	-6.040017754	4.9287830187727 5e-09	6.9214833026068 9e-08	10.2131949215381	Down
REACTOME_ASYMMETRIC_LOCALIZATION_OF_PCP_PROTEINS	-0.257872217	-0.002055718	-6.045611765	4.7799995368117 8e-09	6.7430584374979 e-08	10.2427104528759	Down
GRAESSMANN_APOPTOSIS_BY_SERUM_DEPRIVATION_UP	-0.107097312	0.000933824393601275	-6.047037618	4.7427844855536 e-09	6.7058002965446 9e-08	10.2502369828388	Down
REACTOME_SLBP_DEPENDENT_PROCESSING_OF_REPLICATION_DEPENDENT_HISTONE_PRE_MRNAS	-0.371748073	0.00631276603811388	-6.048639864	4.7013037162272 e-09	6.6775725781744 3e-08	10.2586962413658	Down
HORIUCHI_WTAP_TARGETS_UP	-0.124896974	0.00329250970672978	-6.048801201	4.6971465781964 4e-09	6.6775725781744 3e-08	10.259548131872	Down
FULCHER_INFLAMMATORY_RESPONSE_LECTIN_VS_LPS_UP	-0.101394403	0.00199791908653278	-6.055035955	4.5392221852527 2e-09	6.4919244478948 4e-08	10.2924822499374	Down
WATANABE_RECTAL_CANCER_RADIOTHERAPY_RESPONSIVE_DN	-0.161921393	0.0040731070415289	-6.057759709	4.4718719351517	6.4103716169715	10.30687	Down

		8		2e-09	2e-08	8182 4302	
KEGG_BASAL_TRANSCRIPTION_FACTORS	-0.265309184	0.0065410295913610 3	-6.0757 93496	4.049979 3998966 2e-09	5.873416 3867192 4e-08	10.4 0231 7214 5799	Down
LINDGREN_BLADDER_CANCER_CLUSTER_1_UP	-0.213040205	0.0048891969237648 8	-6.0840 39254	3.870296 3536464 8e-09	5.639185 3209116 7e-08	10.4 4602 7709 621	Down
HOLLMANN_APOPTOSIS_VIA_CD40_DN	-0.153760608	0.0060231545727700 4	-6.0942 88407	3.657804 5566095 4e-09	5.354715 3025649 6e-08	10.5 0042 1023 4441	Down
GARGALOVIC_RESPONSE_TO_OXIDIZED_PHOSPHOLIPIDS_RED_UP	-0.249202814	0.0010547617956673 3	-6.0965 89671	3.611686 6616382 8e-09	5.299701 9169713 4e-08	10.5 1264 3642 9881	Down
REACTOME_SIGNALING_BY_FGFR_IN_DISEASE	-0.145596163	0.0016580213350406 6	-6.1085 64976	3.380732 8765131 3e-09	4.972561 3659992 9e-08	10.5 7630 4258 5707	Down
GARGALOVIC_RESPONSE_TO_OXIDIZED_PHOSPHOLIPIDS_BLACK_UP	-0.227853445	0.0078346915394142 6	-6.1172 35438	3.222601 8392861 5e-09	4.785332 4441265 9e-08	10.6 2245 5572 151	Down

RUTELLA_RESPONSE_TO_HGF_UP	-0.135069122	0.00215024686388051	-6.11946065	3.1832005082299 1e-09	4.7381596054156 e-08	10.6343079878658	Down
REACTOME_CELLULAR_RESPONSES_TO_EXTERNAL_STIMULI	-0.133723395	0.0026448627752294	-6.12128858	3.1511868913905 1e-09	4.7017829410723 4e-08	10.6440467577305	Down
FINETTI_BREAST_CANCER_KINOME_RED	-0.335472341	-0.013631264	-6.126945678	3.0540947703190 5e-09	4.5789290433261 8e-08	10.674200396691	Down
KOBAYASHI_RESPONSE_TO_ROMIDEPSIN	-0.206822937	0.000634877642820148	-6.128782011	3.0232122186073 6e-09	4.5436024796358 1e-08	10.6839930198549	Down
WP_GASTRIC_CANCER_NETWORK_2	-0.222832862	0.0119049303667358	-6.131135175	2.9840844250444 9e-09	4.4956825306434 9e-08	10.6965449971848	Down
MMS_MOUSE_LYMPH_HIGH_4HRS_UP	-0.268185156	0.0101180931850431	-6.139212124	2.8535173863591 6e-09	4.3094361112241 6e-08	10.7396559086108	Down
WANG_ESOPHAGUS_CANCER_PROGRESSION_UP	-0.290400045	0.0027910331468731	-6.143398784	2.7880563912141	4.241535789281e-	10.76201	Down

		1		9e-09	08	9225528	
JOHNSTONE_PARVB_TARGETS_3_DN	-0.291938781	0.00298210389630741	-6.146674252	2.7378696632064 7e-09	4.1857036944636 9e-08	10.77952339489 43	Down
VIETOR_IFRD1_TARGETS	-0.249092355	-0.001813958	-6.15737504	2.5799993250521 3e-09	3.9737111192552 3e-08	10.83675784287 93	Down
DELASERNA_MYOD_TARGETS_DN	-0.174240272	0.00107195291760823	-6.162805623	2.5033263812917 1e-09	3.8652106588750 3e-08	10.86583276062 13	Down
REACTOME_PIWI_INTERACTING_RNA_PIRNA_BIOGENESIS	-0.180916113	-0.002109334	-6.165717804	2.463133770957e-09	3.8126362384863 1e-08	10.88143233220 76	Down
REACTOME_COMPLEX_I_BIOGENESIS	-0.398981234	-0.002294885	-6.17608711	2.3250867514035 6e-09	3.6169958561308 e-08	10.93702239405 17	Down
BROCKE_APOPTOSIS_REVERSED_BY_IL6	-0.186037406	0.00692892767168117	-6.18224446	2.2467297693657 8e-09	3.5038823312696 9e-08	10.97006544075 09	Down

REACTOME_ORC1_REMOVAL_FROM_CHROMATIN	-0.292010858	-0.006066025	-6.183084222	2.2362455894644 5e-09	3.4963164669536 1e-08	10.97457389 84517	Down
REACTOME_UB_SPECIFIC_PROCESSING_PROTEASES	-0.171446587	0.00309935414181116	-6.189788874	2.1542407388229 9e-09	3.3937493060594 7e-08	11.01058594 14961	Down
REACTOME_SCF_SKP2_MEDIATED_DEGRADATION_OF_P27_P21	-0.290977232	-0.006203729	-6.194023652	2.1039703910941 3e-09	3.3229883505143 2e-08	11.03334694 88554	Down
PEDERSEN_METASTASIS_BY_ERBB2_ISOFORM_5	-0.290234753	0.00558432100128064	-6.196019621	2.0806767277138 e-09	3.2945817471733 5e-08	11.04407891 48553	Down
LEE_LIVER_CANCER_SURVIVAL_DN	-0.260653798	0.00216794820820611	-6.198201898	2.0554977994316 5e-09	3.2630370437524 9e-08	11.05581560 90475	Down
IWANAGA_CARCINOGENESIS_BY_KRAS_PTEN_UP	-0.104039113	0.00217515579020403	-6.202732645	2.0041716979888 6e-09	3.1897163408761 2e-08	11.08019274 68352	Down
REACTOME_TRANSCRIPTIONAL_REGULATION_BY_RUNX2	-0.152707195	-0.001846436	-6.207216935	1.9546086784912	3.1188318939318	11.10433	Down

				2e-09	8e-08	31391983	
WELCSH_BRCA1_TARGETS_UP	-0.179140197	0.00850918027800251	-6.209324039	1.93173629591566e-09	3.09028020328569e-08	11.1156809	Down
BIOCARTA_TH1TH2_PATHWAY	-0.215037831	-0.000721836	-6.222443539	1.79511091098076e-09	2.9061663462522e-08	11.1864008321511	Down
REACTOME_APC_C_MEDIATED_DEGRADATION_OF_CELL_CYCLE_PROTEINS	-0.27709252	-0.008497933	-6.227849761	1.7416067776263e-09	2.82988305464043e-08	11.2155754088277	Down
REACTOME_HEDGEHOG_OFF_STATE	-0.129797707	-0.001130326	-6.231565125	1.70574570358449e-09	2.78325295507543e-08	11.2356363387236	Down
BIOCARTA_IFNG_PATHWAY	-0.389667877	0.0075668179209561	-6.241601706	1.61244931797452e-09	2.64075802550603e-08	11.2898732790225	Down
WP_MITOCHONDRIAL_COMPLEX_I_ASSEMBLY_MODEL_OXPHOS_SYSTEM	-0.383993607	-0.00329918	-6.242033703	1.60854791621666e-09	2.64075802550603e-08	11.2922092288117	Down
STARK_PREFRONTAL_CORTEX_22Q11_DELETION_DN	-0.244782597	-0.0013309	-6.2423	1.605366	2.640758	11.2	Down

		73	86754	35768538e-09	02550603e-08	941183780342	
GRAHAM_CML_QUIESCENT_VS_NORMAL_DIVIDING_UP	-0.185812325	0.00171067345145321	-6.25537542	1.49250796476494e-09	2.4638289726851e-08	11.3644119625238	Down
LIANG_HEMATOPOIESIS_STEM_CELL_NUMBER_LARGE_VS_TINY_UP	-0.16464715	0.00174937664086265	-6.256011886	1.48718224227325e-09	2.46158404741068e-08	11.3678592768895	Down
WONG_PROTEASOME_GENE_MODULE	-0.242325701	0.00270747285019625	-6.256042599	1.48692571436965e-09	2.46158404741068e-08	11.3680256380154	Down
REACTOME_MITOCHONDRIAL_TRANSLATION	-0.413434312	-0.007307045	-6.256560661	1.48260523612985e-09	2.46158404741068e-08	11.3708318406491	Down
LIU_VMYB_TARGETS_UP	-0.155089963	0.00343887269412663	-6.25743754	1.47532034325869e-09	2.46158404741068e-08	11.3755820589075	Down
REACTOME_NEDDYLATION	-0.183206613	0.000137202485006667	-6.274808049	1.33803124147531e-09	2.24463781509116e-08	11.469784044	Down

						0306	
FINETTI_BREAST_CANCERS_KINOME_GRAY	-0.455560056	0.0106250 65857195	-6.2771 4035	1.320578 6453735 4e-09	2.221363 5912828 1e-08	11.4 8244 7221 3382	Down
REACTOME_RESPONSE_OF_EIF2AK1_HRI_TO_HEME_D EFICIENCY	-0.210425076	-0.0031275 78	-6.2783 21613	1.311824 5671116 1e-09	2.212634 5347993 9e-08	11.4 8886 2206 2404	Down
REACTOME_PROTEIN_REPAIR	-0.396810838	-0.0017244 31	-6.2785 27723	1.310302 9753920 2e-09	2.212634 5347993 9e-08	11.4 8998 1598 6813	Down
COLINA_TARGETS_OF_4EBP1_AND_4EBP2	-0.114694376	-0.0012317 35	-6.2964 747	1.184236 0018811 7e-09	2.019382 6548561 6e-08	11.5 8755 8023 8688	Down
RODRIGUES_THYROID_CARCINOMA_POORLY_DIFFER ENTIATED_UP	-0.286402374	0.0004565 446382857 41	-6.3049 78548	1.128730 5916569 9e-09	1.935367 6194516 4e-08	11.6 3386 5422 4182	Down
REACTOME_ANTIGEN_PROCESSING_UBIQUITINATION _PROTEASOME_DEGRADATION	-0.143545314	0.0019350 523998891 2	-6.3114 8122	1.088015 3271380 2e-09	1.870723 3062453 5e-08	11.6 6930 6937 5361	Down
WP_OXIDATIVE_PHOSPHORYLATION	-0.382640229	-0.0008632	-6.3142	1.070988	1.850365	11.6	Down

		99	71659	23385187e-09	04384597e-08	845240150282	
VISALA_RESPONSE_TO_HEAT_SHOCK_AND_AGING_UP	-0.200877067	0.0112747702528107	-6.319413677	1.04029341823244e-09	1.80871183388481e-08	11.7125780683296	Down
ACEVEDO_LIVER_TUMOR_VS_NORMAL_ADJACENT_TISSUE_UP	-0.199429382	0.0029261827260226	-6.321852529	1.0260380702902e-09	1.78893772536271e-08	11.7258900110121	Down
KOKKINAKIS_METHIONINE_DEPRIVATION_96HR_DN	-0.155636316	-0.000481997	-6.334382909	9.5576268766304 8e-10	1.67110394431677e-08	11.794346474 09	Down
REACTOME_HEDGEHOG_LIGAND_BIOGENESIS	-0.259287913	-0.00270303	-6.334631726	9.54416138784715e-10	1.67110394431677e-08	11.7957049745641	Down
ZERBINI_RESPONSE_TO_SULINDAC_UP	-0.316232619	0.0220104230361224	-6.335250562	9.51075168129662e-10	1.67110394431677e-08	11.7990884461464	Down
REACTOME_IKK_COMPLEX_RECRUITMENT_MEDIATED_BY_RIP1	-0.208192558	0.00735535293009273	-6.340559073	9.22882702600186e-10	1.63666655286839e-08	11.828122694	Down

						2912	
REACTOME_DNA_REPLICATION_PRE_INITIATION	-0.292167163	-0.005978291	-6.341410827	9.1843614765579 8e-10	1.6334478992835 4e-08	11.8327829374955	Down
BECKER_TAMOXIFEN_RESISTANCE_DN	-0.158953968	0.00347717977315967	-6.351979004	8.6498222481590 9e-10	1.5472463024300 7e-08	11.8906438430133	Down
KENNY_CTNNB1_TARGETS_UP	-0.169469135	0.00442239586591424	-6.353909352	8.5555353288471 8e-10	1.5354695934169 9e-08	11.9012202465148	Down
SAKAI_TUMOR_INFILTRATING_MONOCYTES_DN	-0.254189717	0.00471251837777597	-6.358259729	8.3467226362031 e-10	1.5060496338056 e-08	11.92506478	Down
WP_CELLULAR_PROTEOSTASIS	-0.441335602	-0.005161489	-6.388416413	7.0304965355210 8e-10	1.2797153077999 8e-08	12.0906868333369	Down
TURASHVILI_BREAST_NORMAL_DUCTAL_VS_LOBULAR_UP	-0.190124568	0.00689072540928049	-6.395634356	6.7470006872553 7e-10	1.2317245078174 7e-08	12.1304141812582	Down
CHOW_RASSF1_TARGETS_UP	-0.317072477	0.0005059938755715	-6.399906012	6.5845385116905	1.2056115204148	12.15394	Down

		63		1e-10	4e-08	08682288	
IWANAGA_E2F1_TARGETS_INDUCED_BY_SERUM	-0.329378042	0.00223028999471488	-6.404379707	6.41850616231105e-10	1.17868839495458e-08	12.1785927605497	Down
KEGG_PROTEASOME	-0.352314031	-0.00350148	-6.404925014	6.39855109353471e-10	1.17851058271721e-08	12.1815984974015	Down
REACTOME_STABILIZATION_OF_P53	-0.302760263	-0.002272921	-6.406358712	6.34637599413938e-10	1.1723796367745e-08	12.1895019471492	Down
GRAHAM_CML_QUIESCENT_VS_NORMAL_DIVIDING_DN	-0.262382941	0.0145948798835669	-6.410521307	6.19724425321662e-10	1.14824761431987e-08	12.2124562153467	Down
BIOCARTA_P27_PATHWAY	-0.323601339	-0.001577081	-6.42089545	5.84037186692341e-10	1.08862426960942e-08	12.2697114050258	Down
KAUFFMANN_MELANOMA_RELAPSE_UP	-0.2790614	-0.002826964	-6.425021176	5.70411918219796e-10	1.06965159407561e-08	12.2925003788599	Down

TONG_INTERACT_WITH_PTTG1	-0.222347872	-0.002478729	-6.437773311	5.3023951009679 4e-10	1.0003637201127 1e-08	12.3630066286045	Down
REACTOME_TNFR2_NON_CANONICAL_NF_KB_PATHWAY	-0.206881868	-0.005923987	-6.443083053	5.1434321144521 4e-10	9.7333180287818 4e-09	12.3923944250478	Down
MARKS_HDAC_TARGETS_UP	-0.234081504	0.00342369021821752	-6.448477266	4.9867301248938 4e-10	9.4946729709251 8e-09	12.4222679920975	Down
REACTOME_FASL_CD95L_SIGNALING	-0.402732907	-0.0056721	-6.450456194	4.9304246912976 9e-10	9.416352633503e-09	12.433232063107	Down
JISON_SICKLE_CELL_DISEASE_UP	-0.261135017	-0.004220118	-6.453088181	4.8565037039495 9e-10	9.3038020032145 5e-09	12.4478181762609	Down
RIGGINS_TAMOXIFEN_RESISTANCE_DN	-0.222050881	0.00475884132505296	-6.455178208	4.7985795954084 e-10	9.2499327790993 6e-09	12.4594039399451	Down
WHITFIELD_CELL_CYCLE_S	-0.212681299	0.0079649630911592	-6.457940477	4.7230636058479	9.1327276640180	12.47472	Down

		2		e-10	4e-09	04053592	
NOUZOVA_TRETINOIN_AND_H4_ACETYLATION	-0.197141878	0.00471102374976682	-6.461095121	4.6382464012857 3e-10	8.9967485664939 1e-09	12.492218442451	Down
IIZUKA_LIVER_CANCER_PROGRESSION_L0_L1_DN	-0.270026218	0.00639772363406533	-6.472056056	4.3550032610996 2e-10	8.5173506266025 e-09	12.553064833104	Down
GEORGES_TARGETS_OF_MIR192_AND_MIR215	-0.20536427	0.00382390836554099	-6.4747492	4.2880494089844 7e-10	8.4227603422679 1e-09	12.5680266089877	Down
PEART_HDAC_PROLIFERATION_CLUSTER_UP	-0.174302147	0.00435514534336169	-6.476328625	4.2492545669085 2e-10	8.3730549513654 6e-09	12.5768032389244	Down
GRAHAM_CML_QUIESCENT_VS_CML_DIVIDING_DN	-0.360440853	-0.012070865	-6.485173233	4.0382894501207 6e-10	7.9826951009234 2e-09	12.6259804159806	Down
REACTOME_PENTOSE_PHOSPHATE_PATHWAY	-0.258331728	0.00538295827339729	-6.488668231	3.9577893195163 3e-10	7.8485617591814 2e-09	12.6454266265125	Down

BUFFA_HYPOXIA_METAGENE	-0.232442424	0.0015740854798278 2	-6.5079 29526	3.541609 5570084 4e-10	7.091216 2968875 4e-09	12.7 5273 4298 4041	Down
REACTOME_NUCLEOTIDE_SALVAGE	-0.192956371	0.0022681341856687 6	-6.5131 08397	3.437241 3947712 6e-10	6.904516 9376521 8e-09	12.7 8162 6282 7399	Down
SCHLOSSER_SERUM_RESPONSE_DN	-0.282763318	0.0082438520896663 7	-6.5184 85273	3.332077 8111572 8e-10	6.715002 2642380 7e-09	12.8 1164 0674 4729	Down
PODAR_RESPONSE_TO_ADAPHOSTIN_UP	-0.238523548	0.0088314207810647 4	-6.5204 51182	3.294421 4985581 9e-10	6.682507 9220754 e-09	12.8 2261 9143 7637	Down
LINDGREN_BLADDER_CANCER_CLUSTER_3_UP	-0.220371362	0.0006098351033827 12	-6.5230 86349	3.244600 3871101 3e-10	6.603027 7386205 1e-09	12.8 3733 8821 5103	Down
IGARASHI_ATF4_TARGETS_DN	-0.186905665	0.0022364393852994 2	-6.5272 46785	3.167444 0035053 7e-10	6.467212 1479466 5e-09	12.8 6058 7278 2494	Down
SHIN_B_CELL_LYMPHOMA_CLUSTER_6	-0.280650075	-0.0043416 71	-6.5298 88681	3.119387 3729222	6.390111 3609665	12.8 7535	Down

				8e-10	4e-09	5776 2563	
WILSON_PROTEASES_AT_TUMOR_BONE_INTERFACE_UP	-0.288592292	0.0074839868010927 9	-6.54421851	2.8709332092719 8e-10	5.9006233211758 9e-09	12.9 5553 7065 6467	Down
BIOCARTA_ACH_PATHWAY	-0.267193693	0.0021027068108918 2	-6.570799085	2.460458419378e-10	5.1077141836385 4e-09	13.1 0460 5154 9205	Down
WENDT_COHESIN_TARGETS_UP	-0.362818001	0.0038378827247848 7	-6.572973136	2.4295574179367 2e-10	5.0604909037359 9e-09	13.1 1681 7018 8403	Down
REACTOME_ASSEMBLY_OF_THE_PRE_REPLICATIVE_COMPLEX	-0.297581448	-0.006559746	-6.582604803	2.2971792508251 7e-10	4.8170917600918 4e-09	13.1 7095 4357 0304	Down
RODRIGUES_THYROID_CARCINOMA_ANAPLASTIC_UP	-0.265361363	0.0046474191404889 1	-6.585534942	2.2583313577905 7e-10	4.7516822840020 5e-09	13.1 8743 5398 4143	Down
REACTOME_MTORC1_MEDIATED_SIGNALLING	-0.253116274	0.0028860475974388 4	-6.595292792	2.1335559125031 e-10	4.5352676537351 8e-09	13.2 4235 8367 3604	Down

SASAKI_ADULT_T_CELL_LEUKEMIA	-0.215318834	-0.001943361	-6.600023251	2.0755285915696 6e-10	4.4423468854734 1e-09	13.2690054188796	Down
WINTER_HYPOXIA_UP	-0.139209602	0.000898794446082002	-6.607885056	1.9825002466245 7e-10	4.2875885124734 1e-09	13.3133222064866	Down
BIOCARTA_SALMONELLA_PATHWAY	-0.405509346	0.00197837816463328	-6.611912629	1.9364412214480 6e-10	4.2026191124224 1e-09	13.33604032	Down
REACTOME_DECTIN_1_MEDIATED_NONCANONICAL_NF_KB_SIGNALING	-0.289540987	-0.004506853	-6.620567355	1.8409932792529 8e-10	4.0094895734467 6e-09	13.3848924744449	Down
MILI_PSEUDOPODIA_HAPTOTAXIS_UP	-0.358638768	0.00187823063863552	-6.629700983	1.7452755577079 8e-10	3.8144103474272 6e-09	13.4364979281694	Down
REACTOME_RIPK1_MEDIATED_REGULATED_NECROSIS	-0.231258645	-0.002473685	-6.635081159	1.6911989536836 2e-10	3.7092833588389 5e-09	13.4669202528313	Down
GRAHAM_NORMAL_QUIESCENT_VS_NORMAL_DIVIDING_UP	-0.206191797	0.00323011653622761	-6.647152233	1.5757899067121 6e-10	3.4931885539151 3e-09	13.535241213	Down

CONCANNON_APOPTOSIS_BY_EPOXOMICIN_DN	-0.14936624	-0.002396873	-6.652530225	1.52689765644775e-10	3.40915602646446e-09	234413.5657089086108	Down
TIEN_INTESTINE_PROBIOTICS_6HR_DN	-0.205479943	0.00744506412983219	-6.6634875	1.43184951408836e-10	3.22010504853133e-09	13.6278395974798	Down
SEIDEN_ONCOGENESIS_BY_MET	-0.389320728	0.00546636341451621	-6.663538718	1.43141917283571e-10	3.22010504853133e-09	13.6281301902119	Down
REACTOME_METALLOPROTEASE_DUBS	-0.250733087	0.00607970462807712	-6.664907581	1.41996475119865e-10	3.21668657324454e-09	13.6358972266013	Down
HADDAD_T_LYMPHOCYTE_AND_NK_PROGENITOR_DN	-0.180752621	0.00242827724952826	-6.666764775	1.40456791162374e-10	3.19346264741705e-09	13.6464369351837	Down
REACTOME_MAPK6_MAPK4_SIGNALING	-0.173045656	0.00140655164845523	-6.677947626	1.3152659815933e-10	3.02365035101838e-09	13.7099450431532	Down
EHLERS_ANEUPLOIDY_UP	-0.29910014	0.0023737	-6.6789	1.307593	3.017186	13.7	Down

		6961585586	4298	34787622e-10	58374264e-09	156014350512	
REACTOME_DEGRADATION_OF_BETA_CATENIN_BY_THE_DESTRUCTION_COMPLEX	-0.228937574	0.00696349641395739	-6.688502308	1.2361097321500 3e-10	2.8628854878564 3e-09	13.7699559372364	Down
LINDGREN_BLADDER_CANCER_CLUSTER_2A_DN	-0.226148074	0.0101848330632025	-6.698626968	1.1645856655693 4e-10	2.7175124910484 7e-09	13.8275858	Down
IGLESIAS_E2F_TARGETS_UP	-0.122457452	0.000553996864827817	-6.7036051	1.1309258271517 8e-10	2.6489270223136 2e-09	13.855944410384	Down
BIOCARTA_PROTEASOME_PATHWAY	-0.393080395	-0.00308495	-6.704855085	1.1226253073887 3e-10	2.6394451829401 e-09	13.863067498444	Down
GUTIERREZ_MULTIPLE_MYELOMA_UP	-0.32453046	0.010975795175069	-6.72281575	1.0097649463031 8e-10	2.4106196237322 5e-09	13.9655220539183	Down
TIAN_BHLHA15_TARGETS	-0.249551111	0.00823410034234855	-6.724500737	9.9976774348368 9e-11	2.3959684879549 3e-09	13.9751439346228	Down

REACTOME_FCERI_MEDIATED_NF_KB_ACTIVATION	-0.23123514	0.0020949244162551 3	-6.7246 92556	9.986358 4731556 5e-11	2.395968 4879549 3e-09	13.9 7623 9399 6824	Down
POOLA_INVASIVE_BREAST_CANCER_UP	-0.181291472	-0.0026498 05	-6.7256 64964	9.929171 5050691 5e-11	2.395968 4879549 3e-09	13.9 8179 3104 8236	Down
TURASHVILI_BREAST_DUCTAL_CARCINOMA_VS_LOBULAR_NORMAL_UP	-0.213216145	0.0022715240689242 1	-6.7263 81348	9.887247 3048578 8e-11	2.395968 4879549 3e-09	13.9 8588 4944 4606	Down
KOINUMA_COLON_CANCER_MSI_UP	-0.336003229	-0.0092317 98	-6.7282 80345	9.776954 3074377 2e-11	2.379825 7014221 9e-09	13.9 9673 3139 7209	Down
REACTOME_APC_C_CDH1_MEDIATED_DEGRADATION_OF_CDC20_AND_OTHER_APC_C_CDH1_TARGETED_PROTEINS_IN_LATE_MITOSIS_EARLY_G1	-0.295923342	-0.0079167 08	-6.7317 4174	9.579020 1486821 5e-11	2.350078 1843031 7e-09	14.0 1651 2318 585	Down
REACTOME_METABOLISM_OF_POLYAMINES	-0.29064335	-0.0034175 29	-6.7323 83366	9.542765 0382068 1e-11	2.350078 1843031 7e-09	14.0 2017 9508 3644	Down
REACTOME_INTERLEUKIN_1_FAMILY_SIGNALING	-0.169824381	0.0008694495617965	-6.7411 48529	9.060790 0118152	2.240650 3427624	14.0 7030	Down

		15		3e-11	4e-09	1522 3258	
YIH_RESPONSE_TO_ARSENITE_C3	-0.206684241	-0.0027903 48	-6.7428 9576	8.967614 9371519 3e-11	2.226479 4365960 8e-09	14.0 8029 8320 4765	Down
WILLIAMS_ESR2_TARGETS_DN	-0.315710864	0.0023780 259784989 7	-6.7540 47764	8.394711 9522332 2e-11	2.101047 4632061 1e-09	14.1 4414 8187 7241	Down
WIERENGA_STAT5A_TARGETS_GROUP2	-0.18547648	0.0039501 035450100 4	-6.7543 59166	8.379242 1273739 3e-11	2.101047 4632061 1e-09	14.1 4593 2176 4498	Down
INGA_TP53_TARGETS	-0.257276504	0.0066084 635783129 7	-6.7603 07732	8.089053 2740748 3e-11	2.041006 2468366 9e-09	14.1 8002 2104 7514	Down
TAKEDA_TARGETS_OF_NUP98_HOXA9_FUSION_6HR_UP	-0.187270641	0.0032050 014980736 2	-6.7618 3943	8.015946 3337808 5e-11	2.030815 4650521 5e-09	14.1 8880 3394 3897	Down
REACTOME_CLASS_I_MHC_MEDIATED_ANTIGEN_PROCESSING_PRESENTATION	-0.142004502	0.0042566 941343443 8	-6.7657 87934	7.830469 0202922 7e-11	1.991955 7872522 2e-09	14.2 1144 6873 2073	Down

WOOD_EBV_EBNA1_TARGETS_DN	-0.238428409	0.0048299228996695 2	-6.786646891	6.918488 4789532 4e-11	1.781869 6260938 9e-09	14.3 3122 2879 5665	Down
REACTOME_RESPIRATORY_ELECTRON_TRANSPORT_ATP_SYNTHESIS_BY_CHEMIOSMOTIC_COUPLING_AND_HEAT_PRODUCTION_BY_UNCOUPLING_PROTEINS_	-0.401473386	-0.000469415	-6.794046319	6.620759 5007867 e-11	1.712293 9258909 6e-09	14.3 7377 4786 3074	Down
LIAN_LIPA_TARGETS_3M	-0.272248886	-0.000481248	-6.807817757	6.099806 6347604 4e-11	1.584163 1707932 2e-09	14.4 5305 7898 3218	Down
SENGUPTA_NASOPHARYNGEAL_CARCINOMA_UP	-0.269793313	-0.00063713	-6.812131311	5.945081 9410583 2e-11	1.550467 3784936 5e-09	14.4 7791 4757 5541	Down
YAO_TEMPORAL_RESPONSE_TO_PROGESTERONE_CLUSTER_13	-0.327478142	-0.001110572	-6.822504961	5.588611 9131682 2e-11	1.463650 3858664 6e-09	14.5 3773 8622 5325	Down
KEGG_SNARE_INTERACTIONS_IN_VESICULAR_TRANSPORT	-0.204097517	0.0006568092650855 76	-6.839613105	5.046108 1640301 5e-11	1.344257 2263577 3e-09	14.6 3654 0488 5007	Down
DOUGLAS_BMI1_TARGETS_DN	-0.148206725	0.0051923420597394	-6.848187178	4.794053 5502602	1.288168 4149985	14.6 8612	Down

		6		4e-11	e-09	276	
YU_MYC_TARGETS_UP	-0.36229109	-0.0133051 93	-6.8560 27467	4.574433 2895351 e-11	1.234500 3229628 e-09	14.7 3150 0109 2062	Down
REACTOME_SIGNALING_BY_FGFR4_IN_DISEASE	-0.317411519	0.0100687 886380639	-6.8660 23952	4.308718 9421583 5e-11	1.167869 8023570 7e-09	14.7 8941 0047 9541	Down
REACTOME_REGULATION_OF_RUNX3_EXPRESSION_A ND_ACTIVITY	-0.309822117	-0.0026716 42	-6.8697 41846	4.213818 1883535 2e-11	1.147156 5568030 8e-09	14.8 1096 3107 2363	Down
MCCABE_HOXC6_TARGETS_CANCER_DN	-0.246240222	0.0055941 258846418 5	-6.8770 94209	4.032159 8556985 6e-11	1.107416 6470938 5e-09	14.8 5360 9800 9883	Down
DAZARD_UV_RESPONSE_CLUSTER_G1	-0.249276298	-0.0006247 09	-6.8793 25534	3.978569 3236015 9e-11	1.097554 6574042 2e-09	14.8 6655 8751 739	Down
WONG_MITOCHONDRIA_GENE_MODULE	-0.336802795	-0.0028304 46	-6.9005 57691	3.502442 2895180 5e-11	9.972320 7757057 6e-10	14.9 8992 2260 8604	Down
NUYTTEN_EZH2_TARGETS_UP	-0.162595689	0.0016123	-6.9141	3.227083	9.230648	15.0	Down

		8583033737	74226	49924526e-11	51604393e-10	691779159685	
FORTSCHEGGER_PHF8_TARGETS_UP	-0.157200094	0.00227174392806549	-6.917277966	3.1673638114752 1e-11	9.1441056641054 2e-10	15.0872587201809	Down
CREIGHTON_ENDOCRINE_THERAPY_RESISTANCE_3	-0.147333802	0.00277802431479798	-6.923117102	3.0579444466854 6e-11	8.8694678413909 6e-10	15.1212899716488	Down
SPIRA_SMOKERS_LUNG_CANCER_UP	-0.31292649	0.0068032351791811	-6.932408506	2.8914547098231 5e-11	8.42594337271e-10	15.1754829792613	Down
REACTOME_RUNX1_REGULATES_TRANSCRIPTION_OF_GENES_INVOLVED_IN_DIFFERENTIATION_OF_HSCS	-0.26178029	-0.002261344	-6.941700542	2.7338820666620 4e-11	8.0422777193228 9e-10	15.2297305779863	Down
REACTOME_DEGRADATION_OF_DVL	-0.310981368	-0.001025146	-6.955771483	2.5112239298687 4e-11	7.5300323346354 e-10	15.3119745136514	Down
REACTOME_RESPIRATORY_ELECTRON_TRANSPORT	-0.432673162	-0.000395255	-6.95695693	2.493302063359e-11	7.5125853918783 1e-10	15.318908710	Down

						9243	
PARK_APL_PATHOGENESIS_UP	-0.350667663	0.00192392636916424	-6.965620902	2.3660854668455 4e-11	7.1991629866226 7e-10	15.3696130972029	Down
REACTOME_ENERGY_DEPENDENT_REGULATION_OF_MTOR_BY_LKB1_AMPK	-0.251860249	0.000312470145131493	-6.969947248	2.3049735657844 1e-11	7.0826588726850 6e-10	15.3949487686791	Down
WANG_SMARCE1_TARGETS_DN	-0.210225759	0.00140696430464792	-6.989097592	2.0526048605444 5e-11	6.5002644741833 8e-10	15.5072274780477	Down
REACTOME_MRNA_DECAY_BY_5_TO_3_EXORIBONUCLEASE	-0.383033414	0.00166446171134999	-6.998484686	1.9390372092385 6e-11	6.2685437279915 3e-10	15.5623424671343	Down
REACTOME_NEGATIVE_REGULATION_OF_NOTCH4_SIGNALING	-0.327793737	-0.001395428	-7.003727729	1.8783216369960 5e-11	6.1243214689176 6e-10	15.5931486406343	Down
NIKOLSKY_BREAST_CANCER_8Q12_Q22_AMPLICON	-0.258442655	0.00477359577317389	-7.013166096	1.7737042370202 5e-11	5.8250699466585 6e-10	15.6486453861891	Down
REACTOME_ASSEMBLY_OF_THE_ORC_COMPLEX_AT_	-0.432953421	-0.0006004	-7.0163	1.739271	5.742372	15.6	Down

THE_ORIGIN_OF_REPLICATION		54	92763	83041844e-11	47415281e-10	676298016661	
GEISS_RESPONSE_TO_DSRNA_UP	-0.275695134	-0.003264758	-7.017480634	1.72781193660459e-11	5.73504208048378e-10	15.6740317657908	Down
REACTOME_TRANSLESION_SYNTHESIS_BY_POLK	-0.355086051	0.00696041577204946	-7.025673534	1.64386133532317e-11	5.51537692343294e-10	15.7222679163808	Down
KEGG_HUNTINGTONS_DISEASE	-0.228698512	0.00154648050738828	-7.036674921	1.53742000997451e-11	5.18628587060422e-10	15.7871005604594	Down
REACTOME_ABC_FAMILY_PROTEINS_MEDIATED_TRANSPORT	-0.196155793	-0.0012217	-7.052831694	1.39329025492059e-11	4.72576645480443e-10	15.8824418854976	Down
GROSS_ELK3_TARGETS_DN	-0.219423864	0.00324169920887084	-7.058900966	1.34265843303975e-11	4.60435408501533e-10	15.9182958106207	Down
WP_ELECTRON_TRANSPORT_CHAIN_OXPHOS_SYSTEM_IN_MITOCHONDRIA	-0.410341804	-0.000310161	-7.068326649	1.26759665046047e-11	4.37109578300452e-10	15.974019795	Down

						8298	
ACEVEDO_LIVER_CANCER_UP	-0.237654952	0.00415583435934139	-7.069678078	1.2571788831947 5e-11	4.3593906860278 4e-10	15.982013557927	Down
KIM_WT1_TARGETS_8HR_DN	-0.136376896	0.00281080491296795	-7.124545825	8.9847693820600 9e-12	3.2236106100836 4e-10	16.307446998353	Down
REACTOME_CROSS_PRESENTATION_OF_SOLUBLE_EXOGENOUS_ANTIGENS_ENDOSOMES_	-0.356128113	-0.000857527	-7.128111987	8.7902070548742 7e-12	3.1842213535017 7e-10	16.3286585646663	Down
BARIS_THYROID_CANCER_UP	-0.314680892	0.00354390992065412	-7.142496188	8.0466927916761 1e-12	2.9553740921854 2e-10	16.4142897883209	Down
LINDSTEDT_DENDRITIC_CELL_MATURATION_A	-0.24247608	-0.005205023	-7.15469886	7.4647812271567 9e-12	2.7911986190941 1e-10	16.4870267077333	Down
GARGALOVIC_RESPONSE_TO_OXIDIZED_PHOSPHOLIPIDS_LIGHTYELLOW_UP	-0.378005711	0.000111996220882231	-7.173933954	6.6306381350501 9e-12	2.5249307303224 9e-10	16.6018543408534	Down
NAGY_PCAF_COMPONENTS_HUMAN	-0.307442204	0.0087633	-7.1931	5.888249	2.270084	16.7	Down

		9762356543	75509	0400344e-12	58332258e-10	169309393898	
DAZARD_UV_RESPONSE_CLUSTER_G28	-0.317525713	0.000772452807805381	-7.212151228	5.2365042435383 3e-12	2.0571507493444 6e-10	16.8306231468794	Down
TAYLOR_METHYLATED_IN_ACUTE_LYMPHOBLASTIC_LEUKEMIA	-0.204771774	0.00351045938616206	-7.212396891	5.2285509990489 8e-12	2.0571507493444 6e-10	16.8320963617741	Down
REACTOME_UBIQUINOL_BIOSYNTHESIS	-0.379505981	-0.008172695	-7.219765467	4.9954515617374 5e-12	2.0004366350777e-10	16.8763007696469	Down
DING_LUNG_CANCER_EXPRESSION_BY_COPY_NUMBER	-0.380946586	0.00841669049922892	-7.220254446	4.9803506635940 8e-12	2.0004366350777e-10	16.8792352577171	Down
REACTOME_PROGRAMMED_CELL_DEATH	-0.16426083	0.00412798248633447	-7.246860674	4.2230921864062 7e-12	1.7245219211199 8e-10	17.0391093036621	Down
REACTOME_DEGRADATION_OF_AXIN	-0.338561032	-0.001068242	-7.253809111	4.0447888956583 6e-12	1.6626493162484 4e-10	17.080927314	Down

						9361	
CHEOK_RESPONSE_TO_MERCAPTOPURINE_DN	-0.218097819	-0.001223576	-7.275798845	3.5280084806260 1e-12	1.4796181513003 8e-10	17.2134473452135	Down
VALK_AML_CLUSTER_3	-0.227586976	0.00502025453712187	-7.278805549	3.4626079990018 9e-12	1.4620685612112 1e-10	17.2315881373291	Down
YIH_RESPONSE_TO_ARSENITE_C1	-0.27650714	0.00748937812630165	-7.281974326	3.3949755399886 5e-12	1.4433296696376 4e-10	17.2507122645409	Down
SEIDEN_MET_SIGNALING	-0.415172952	0.00428979173597924	-7.289056291	3.2484943151966 7e-12	1.3905796009948 8e-10	17.2934734509539	Down
CHEN_HOXA5_TARGETS_9HR_UP	-0.339704722	0.00641777789453411	-7.296990339	3.0917808065486 3e-12	1.3326863518227 3e-10	17.3414128004827	Down
DAZARD_RESPONSE_TO_UV_SCC_UP	-0.302490773	0.011140039987563	-7.298131737	3.0698579293734 4e-12	1.3324900816518 1e-10	17.3483122797935	Down
MOOTHA_VOXPHOS	-0.425522517	-0.0013199	-7.3006	3.021544	1.330122	17.3	Down

		36	75763	6751803 3e-12	5389251 3e-10	6369 2920 5954	
HALMOS_CEBPA_TARGETS_UP	-0.267001505	0.0071662 081809489 4	-7.3155 86253	2.753088 2305485 5e-12	1.229382 6364758 9e-10	17.4 5391 107	Down
SCHAVOLT_TARGETS_OF_TP53_AND_TP63	-0.303592155	-0.0001987 1	-7.3319 88091	2.484896 7061951 e-12	1.125821 4492958 4e-10	17.5 5329 5602 0085	Down
REACTOME_DEFECTIVE_CFTR_CAUSES_CYSTIC_FIBR OSIS	-0.315122244	-0.0047587 31	-7.3339 48302	2.454620 6345392 9e-12	1.120281 6381312 8e-10	17.5 6518 3198 7877	Down
TCGA_GLIOBLASTOMA_COPY_NUMBER_DN	-0.301749099	0.0050517 952995364 9	-7.3352 03851	2.435419 6842763 6e-12	1.119751 8503928 4e-10	17.5 7279 8530 971	Down
REACTOME_DEGRADATION_OF_GLI1_BY_THE_PROTE ASOME	-0.314343896	0.0002780 259066986 25	-7.3355 2978	2.430459 5546663 4e-12	1.119751 8503928 4e-10	17.5 7477 5543 9644	Down
AKL_HTLV1_INFECTION_DN	-0.274899696	0.0041372 755266129 9	-7.3360 17977	2.423048 5866204 8e-12	1.119751 8503928 4e-10	17.5 7773 6949 4644	Down

REACTOME_REGULATION_OF_RUNX2_EXPRESSION_AND_ACTIVITY	-0.283730881	-0.003257862	-7.355921004	2.1391625369837 8e-12	1.0058925656862 4e-10	17.6985811167482	Down
REACTOME_HIV_TRANSCRIPTION_INITIATION	-0.321694042	0.00286298440546684	-7.365553683	2.0138069586019 e-12	9.5994931370946 7e-11	17.7571459857673	Down
BENPORATH_PROLIFERATION	-0.335060097	-0.005288308	-7.370059528	1.9576844080656 9e-12	9.4748019619991 6e-11	17.7845582417793	Down
PETRETTO_BLOOD_PRESSURE_DN	-0.378735387	0.0121446717800042	-7.373016564	1.9216942693427 5e-12	9.3920915982759 6e-11	17.8025540809036	Down
REACTOME_ANTIGEN_PROCESSING_CROSS_PRESENTATION	-0.271269714	-0.000328627	-7.385682808	1.7747755387903 7e-12	8.8128254154174 4e-11	17.8796923862364	Down
REACTOME_SUMO_IS_PROTEOLYTICALLY_PROCESSED	-0.469759779	0.00418007308536855	-7.3873494	1.7562883354061 4e-12	8.7913562079564 e-11	17.8898486203133	Down
GRAHAM_CML_DIVIDING_VS_NORMAL_QUIESCENT_DN	-0.217067634	0.0014633414265967	-7.398116066	1.6413497211856	8.2828111539829	17.95549	Down

		6		1e-12	7e-11	7590 5283	
KUWANO_RNA_STABILIZED_BY_NO	-0.434158919	0.0227360 9288893	-7.4000 28245	1.621726 9028678 6e-12	8.250867 9394268 8e-11	17.9 6716 3620 2562	Down
WP_HIJACK_OF_UBIQUITINATION_BY_SARSCOV2	-0.476886468	0.0007109 705800373 16	-7.4108 21414	1.515229 5543057 e-12	7.772751 9368392 5e-11	18.0 3304 9350 6605	Down
REACTOME_TERMINATION_OF_TRANSLESION_DNA_S YNTHESIS	-0.292884863	0.0043032 187071122 9	-7.4111 63784	1.511966 7361490 7e-12	7.772751 9368392 5e-11	18.0 3514 0358 5535	Down
WP_OXIDATIVE_DAMAGE	-0.203448542	0.0045841 369883172 2	-7.4204 23462	1.426302 4981621 e-12	7.439545 8874724 1e-11	18.0 9171 7642 1842	Down
BYSTRYKH_HEMATOPOIESIS_STEM_CELL_QTL_CIS	-0.186589492	0.0045403 34733812	-7.4213 34177	1.418140 3797570 8e-12	7.439545 8874724 1e-11	18.0 9728 4705 4298	Down
WP_PROTEASOME_DEGRADATION	-0.311166827	0.0012890 776521808 7	-7.4235 88584	1.398133 3138140 4e-12	7.417276 4776442 2e-11	18.1 1106 7513 4263	Down

SUBTIL_PROGESTIN_TARGETS	-0.203559177	0.00477868724697676	-7.440527868	1.25645001784459e-12	6.72309074203566e-11	18.2147182884349	Down
HOEBEKE_LYMPHOID_STEM_CELL_DN	-0.177708482	0.00255247704069753	-7.451003516	1.17601997827724e-12	6.3474400044929e-11	18.2788966381642	Down
REACTOME_TRANSLESION_SYNTHESIS_BY_Y_FAMILY_DNA_POLYMERASES_BYPASSES_LESIONS_ON_DNA_TEMPLATE	-0.272852673	0.00544301891675479	-7.45419898	1.1525126889219e-12	6.27512829836687e-11	18.298485324753	Down
SCHAEFFER_PROSTATE_DEVELOPMENT_6HR_UP	-0.235465644	0.00371547815556836	-7.477543278	9.94287869383769e-13	5.5103078618438e-11	18.4417577456469	Down
KEGG_PARKINSONS_DISEASE	-0.349054033	0.000613746052856715	-7.47798361	9.91519370342766e-13	5.5103078618438e-11	18.4444630607427	Down
REACTOME_METABOLISM_OF_COFACTORS	-0.297569199	-0.001939582	-7.506370216	8.28211678244133e-13	4.67337262441939e-11	18.6190864275665	Down
BURTON_ADIPOGENESIS_6	-0.164262941	0.0011624461812342	-7.508144336	8.1893658649897	4.6634306352285	18.63001	Down

		8		3e-13	5e-11	4576304	
WP_NANOMATERIAL_INDUCED_APOPTOSIS	-0.285808274	0.00989820691062043	-7.5092308	8.1330724332113 9e-13	4.6634306352285 5e-11	18.63670776580 32	Down
BLANCO_MELO_INFLUENZA_A_INFECTION_A594_CELLS_UP	-0.305518726	-0.000671663	-7.511314567	8.0261716772559e-13	4.6559296823109 7e-11	18.64954664883 82	Down
REACTOME_PHOSPHATE_BOND_HYDROLYSIS_BY_NUDT_PROTEINS	-0.37422458	0.00207442772261739	-7.567733874	5.6046161485560 4e-13	3.3449858109699 4e-11	18.99805427855 56	Down
KAAB_FAILED_HEART_ATRIUM_DN	-0.169798292	0.0027318988669446	-7.592215574	4.7933869701901 8e-13	2.9169169533304 3e-11	19.14980986569 58	Down
GARGALOVIC_RESPONSE_TO_OXIDIZED_PHOSPHOLIPIDS_BLUE_UP	-0.223378172	0.00603409477675502	-7.597633288	4.6301727424961e-13	2.8454932883835e-11	19.18343595917 2	Down
REACTOME_CELLULAR_RESPONSE_TO_HYPOXIA	-0.269753604	0.00309596099531755	-7.611242955	4.2439752107265 8e-13	2.6342354132979 9e-11	19.26797578092 68	Down

KRIEG_KDM3A_TARGETS_NOT_HYPOXIA	-0.281119778	0.0016109573067535 3	-7.618924811	4.0402160254086 1e-13	2.5330930171425 5e-11	19.3157369041898	Down
YAO_TEMPORAL_RESPONSE_TO_PROGESTERONE_CLUSTER_15	-0.229496399	0.0010726140743943 1	-7.628282383	3.8050199995175 4e-13	2.4099754221434 e-11	19.3739588240899	Down
WP_EUKARYOTIC_TRANSCRIPTION_INITIATION	-0.296968399	-0.000145756	-7.655379619	3.197549446499e-13	2.0891778330967 6e-11	19.542816129311	Down
REACTOME_INITIATION_OF_NUCLEAR_ENVELOPE_NE_REFORMATION	-0.292666518	0.0024355559021454 9	-7.659227196	3.1194483504868 3e-13	2.0598314799438 e-11	19.5668238013083	Down
SESTO_RESPONSE_TO_UV_C0	-0.243310767	0.0079593974641992 5	-7.674237176	2.8323670146595 3e-13	1.9109241369556 2e-11	19.6605557745783	Down
REACTOME_INTERLEUKIN_1_SIGNALING	-0.241800858	0.0015076750480909 9	-7.687646989	2.5980537274673 6e-13	1.7721010424604 3e-11	19.7443952711378	Down
KOKKINAKIS_METHIONINE_DEPRIVATION_48HR_UP	-0.161955626	0.0026045836658551	-7.699120788	2.4128414888769	1.6827536091527	19.81620	Down

		3		6e-13	3e-11	5360 2431	
REACTOME_APOPTOTIC_FACTOR_MEDIATED_RESPONSE	-0.282873521	0.000627456433928287	-7.713340894	2.2013065236233 5e-13	1.570518343923e-11	19.9 0529 8975 2244	Down
SMIRNOV_RESPONSE_TO_IR_6HR_UP	-0.160856383	0.00546488252358725	-7.733307112	1.9348787787932 7e-13	1.4129167741141 e-11	20.0 3057 1727 3916	Down
DEBIASI_APOPTOSIS_BY_REOVIRUS_INFECTION_UP	-0.301102403	0.0053043161817578	-7.741956786	1.8295928047508 5e-13	1.3519383975105 4e-11	20.0 8490 6162 9982	Down
HOLLEMAN_ASPARAGINASE_RESISTANCE_ALL_DN	-0.31069676	0.0108287402470634	-7.743120752	1.8158637887576 6e-13	1.3519383975105 4e-11	20.0 9222 0782 3863	Down
CHIANG_LIVER_CANCER_SUBCLASS_UNANNOTATED_DN	-0.353662801	0.0047157314071444	-7.748926159	1.7488937022834 5e-13	1.3401707666757 2e-11	20.1 2871 3734 5489	Down
REACTOME_ABC_TRANSPORTER_DISORDERS	-0.259991096	-0.002578721	-7.748968192	1.7484178292621 4e-13	1.3401707666757 2e-11	20.1 2897 8016 7036	Down

GHANDHI_BYSTANDER_IRRADIATION_UP	-0.269619644	-0.003633705	-7.759416346	1.6340107564261 2e-13	1.3002954827098 6e-11	20.1946996352876	Down
NIKOLSKY_BREAST_CANCER_6P24_P22_AMPLICON	-0.282929546	0.00424903690928787	-7.766650279	1.5591642550861 5e-13	1.2568483806908 7e-11	20.2402360476806	Down
JIANG_TIP30_TARGETS_UP	-0.243993461	0.00514352814596447	-7.795104348	1.2962323839215 7e-13	1.0727619209334 9e-11	20.419611767872	Down
PUIFFE_INVASION_INHIBITED_BY_ASCITES_DN	-0.33588179	0.00209158564699146	-7.826080292	1.0596475349618 3e-13	9.0099071911069 6e-12	20.6153587675915	Down
REACTOME_DNA_DAMAGE_BYPASS	-0.276374256	0.00629054747451181	-7.827005602	1.0532797797288 2e-13	9.0099071911069 6e-12	20.6212136669748	Down
SHARMA_PILOCYTIC_ASTROCYTOMA_LOCATION_UP	-0.274814972	0.00760600592760437	-7.84498788	9.3677408861285 5e-14	8.3065096685999 8e-12	20.7350833405454	Down
WANG_CLIM2_TARGETS_DN	-0.287372122	0.0028790674285075	-7.849530681	9.0941623252440	8.3010978754103	20.76387	Down

		8		9e-14	e-12	59566701	
BOUDOUKHA_BOUND_BY_IGF2BP2	-0.242235957	0.00245624484612373	-7.860278412	8.4779464397181 1e-14	7.8541214255716 9e-12	20.8320377363896	Down
YU_BAP1_TARGETS	-0.263037053	-0.001621754	-7.864200331	8.2635169865069 8e-14	7.7714621114013 4e-12	20.856925058836	Down
NUYTTEN_NIPP1_TARGETS_UP	-0.249083957	0.00160153010883526	-7.898212501	6.6151234028914 3e-14	6.4705751049505 8e-12	21.0730834708373	Down
GOLDRATH_ANTIGEN_RESPONSE	-0.224785872	-0.001084452	-7.918210386	5.8023960216958 9e-14	6.0025786844444 e-12	21.2004496624173	Down
GALI_TP53_TARGETS_APOPTOTIC_UP	-0.365981654	0.00739464684365836	-7.988341109	3.6582548221537 8e-14	3.8833097205270 8e-12	21.6486981262694	Down
REACTOME_SIGNALING_BY_NOTCH4	-0.256888638	0.00316511372312565	-7.991392386	3.5853681094943 9e-14	3.8833097205270 8e-12	21.6682564818086	Down

REACTOME_REGULATION_OF_RAS_BY_GAPS	-0.313024385	-5.36E-05	-8.009080177	3.190280610563e-14	3.5360842410293 8e-12	21.78172442563 11	Down
MULLIGAN_NTF3_SIGNALING_VIA_INSR_AND_IGF1R_DN	-0.471378003	0.0140394848334286	-8.019636723	2.9753291726244e-14	3.3577942135417 6e-12	21.84951907250 52	Down
REACTOME_WAX_AND_PLASMALOGEN_BIOSYNTHESIS	-0.498178925	0.00739760099926991	-8.02641426	2.8449403395186e-14	3.2701008680355 4e-12	21.89307382042 56	Down
GAJATE_RESPONSE_TO_TRABECTEDIN_UP	-0.200892798	0.00107944969393619	-8.031915625	2.7432707498410 5e-14	3.2127323668421 5e-12	21.92844415186 4	Down
REACTOME_SIGNALING_BY_FGFR3_FUSIONS_IN_CANCER	-0.407298464	0.0109447694055277	-8.041072914	2.5819454344224 7e-14	3.0819490983577 4e-12	21.98735291954 02	Down
HUANG_GATA2_TARGETS_UP	-0.277881529	0.00327008564982718	-8.067868055	2.1618482152524 4e-14	2.7384881371575 3e-12	22.15996309830 11	Down
CHICAS_RB1_TARGETS_LOW_SERUM	-0.296598965	-0.010458753	-8.120740927	1.5212538380095	2.0090260792607	22.50159	Down

				5e-14	e-12	40499566	
CAFFAREL_RESPONSE_TO_THC_24HR_5_UP	-0.333818727	0.00884590198687803	-8.145523678	1.2896192868811 9e-14	1.7401449812329 5e-12	22.6621931193791	Down
CHIANG_LIVER_CANCER_SUBCLASS_INTERFERON_UP	-0.319154395	0.000347366055189345	-8.158669914	1.1812837709892 8e-14	1.6293840814512 1e-12	22.7475051355887	Down
HUMMERICH_BENIGN_SKIN_TUMOR_UP	-0.329937302	0.00184821504900031	-8.17047532	1.0916991740591 2e-14	1.5400401757693 1e-12	22.82418703	Down
SASSON_FSH_RESPONSE	-0.420659349	0.0156550866556467	-8.175008939	1.0591118007403 7e-14	1.5288155691152 3e-12	22.8536529572634	Down
ALONSO_METASTASIS_UP	-0.280450705	0.00635309948606162	-8.19733579	9.1214602405499 9e-15	1.3480215169784 2e-12	22.9989089926145	Down
REACTOME_TP53_REGULATES_METABOLIC_GENES	-0.246234529	0.00523514663786432	-8.223326878	7.6631889397629 6e-15	1.1891353437277 2e-12	23.1683052966971	Down
MARZEC_IL2_SIGNALING_DN	-0.327278613	0.0059277	-8.2320	7.229796	1.171511	23.2	Down

		4595105967	02323	35633013e-15	44375915e-12	249191730784	
REACTOME_HEDGEHOG_ON_STATE	-0.233151363	0.00166891619653938	-8.241066482	6.8028862148813 6e-15	1.1412301279937 5e-12	23.2841080367246	Down
KEGG_OXIDATIVE_PHOSPHORYLATION	-0.364014173	0.00433971733700331	-8.417723611	2.0615380674260 9e-15	3.7635196425040 4e-13	24.4454074629695	Down
WILCOX_RESPONSE_TO_PROGESTERONE_UP	-0.282602192	0.00113935965657441	-8.425421448	1.9563879859950 7e-15	3.7532737337278 1e-13	24.4963415890655	Down
THILLAINADESAN_ZNF217_TARGETS_DN	-0.461129539	0.00582392402125341	-8.497048687	1.2004216006312 9e-15	2.4035538306833 6e-13	24.9715788555752	Down
WP_MITOCHONDRIAL_CIV_ASSEMBLY	-0.519985109	-0.004438265	-8.505797905	1.1307173995397 3e-15	2.3394542996477 1e-13	25.0297891277236	Down
KEGG_ALZHEIMERS_DISEASE	-0.261011957	0.00346744460332698	-8.536362943	9.1722815388103 2e-16	1.9631845348757 1e-13	25.233416494	Down

						1093	
HIRSCH_CELLULAR_TRANSFORMATION_SIGNATURE_UP	-0.243926214	0.00392482946219393	-8.546178922	8.5753844836858 4e-16	1.9009789817942 2e-13	25.2989010385495	Down
NEWMAN_ERCC6_TARGETS_UP	-0.332867295	-0.000938314	-8.648161982	4.2513846454239 1e-16	9.7734609237578 6e-14	25.9818041316619	Down
WP_NONALCOHOLIC_FATTY_LIVER_DISEASE	-0.266837551	0.00442206949827951	-8.654693671	4.0639258361273 6e-16	9.7018414095548 1e-14	26.0256993590146	Down
HAHTOLA_SEZARY_SYNDROM_UP	-0.329140412	0.0034003540178339	-8.696499854	3.0436992313828 4e-16	7.5568964516773 1e-14	26.3070961745239	Down
CLAUS_PGR_POSITIVE_MENINGIOMA_UP	-0.412504197	-0.005278321	-8.70225138	2.9248523290352 6e-16	7.5568964516773 1e-14	26.3458696044877	Down
LAIHO_COLORECTAL_CANCER_SERRATED_UP	-0.354872731	0.00518535763758195	-8.729023094	2.4294358814522 5e-16	6.5563080505104 7e-14	26.5265389260991	Down
JINESH_BLEBBISHIELD_VS_LIVE_CONTROL_DN	-0.184846088	-0.0006313	-8.7575	1.993375	5.624037	26.7	Down

		02	11971	7397831 6e-16	8258336 8e-14	1913 8866 3825	
WP_GENOTOXICITY_PATHWAY	-0.299578385	0.0042806994438073 7	-8.7650 68611	1.891361 1066086 3e-16	5.590323 0422475 e-14	26.7 7028 4759 4399	Down
YAO_TEMPORAL_RESPONSE_TO_PROGESTERONE_CLUSTER_9	-0.22296721	0.0030030019246978 9	-8.7814 78777	1.687301 8479290 2e-16	5.236541 2850477 2e-14	26.8 8143 9193 3831	Down
CHEN_LIVER_METABOLISM_QTL_CIS	-0.20270913	-0.0006838 23	-8.9073 6594	7.001312 3620459 6e-17	2.287218 2016431 2e-14	27.7 3796 6854 9194	Down
WU_HBX_TARGETS_1_UP	-0.461486202	0.0002182501377946 77	-8.9586 94455	4.881887 7495696 3e-17	1.683437 6256432 6e-14	28.0 8912 2988 4029	Down
IVANOVA_HEMATOPOIESIS_LATE_PROGENITOR	-0.222365474	0.0057077523824118	-8.9790 04891	4.231495 2684693 2e-17	1.544993 5959640 6e-14	28.2 2837 6957 7185	Down
MARTINEZ_RESPONSE_TO_TRABECTEDIN_UP	-0.259707003	0.0001960021489582 52	-8.9895 53786	3.928385 3358589 6e-17	1.544993 5959640 6e-14	28.3 0077 0589	Down

						5154	
BAE_BRCA1_TARGETS_UP	-0.317846527	0.0020781246800071 2	-9.247849271	6.2762751575311 3e-18	2.9966799925227 5e-15	30.0874904986576	Down
MONNIER_POSTRADIATION_TUMOR_ESCAPE_DN	-0.187000445	0.0003506027573107	-9.317828115	3.8012338755168 9e-18	1.9661882221111 1e-15	30.5761253464954	Down
MARIADASON_RESPONSE_TO_BUTYRATE_CURCUMIN _SULINDAC_TSA_8	-0.462308158	0.0085322449845951 2	-9.373248005	2.5519417882095 9e-18	1.5839902679416 9e-15	30.9644465224422	Down
YANAGISAWA_LUNG_CANCER_RECURRENCE	-0.575226612	0.0004116986012949 27	-9.455894374	1.4055720392517 1e-18	9.6937618307059 8e-16	31.5457188157657	Down
MARTORIATI_MDM4_TARGETS_FETAL_LIVER_UP	-0.132110294	0.0018879857845241 6	-9.642382216	3.6250841281169 5e-19	3.2144138833174 2e-16	32.8666992442738	Down
STONER_ESOPHAGEAL_CARCINOGENESIS_UP	-0.25018627	0.0021779257328500 7	-9.846475145	8.1088870029399 5e-20	1.2302644818630 6e-16	34.3267662938929	Down
BARRIER_CANCER_RELAPSE_TUMOR_SAMPLE_UP	-0.553848132	0.0050013	-10.095	1.282493	2.653479	36.1	Down

		3683555568	15959	54519059e-20	14499934e-17	252246479694	
VERHAAK_GLIOBLASTOMA_NEURAL	-0.228820856	0.00080618095033852	-10.13832027	9.29267344872382e-21	2.65347914499934e-17	36.4394472952843	Down
HAHTOLA_CTCL_CUTANEOUS	-0.391997234	0.0113769800950132	-10.40065009	1.29470750395034e-21	8.03624947701975e-18	38.3620245660849	Down

S6 Table Results of Immune Cell Infiltration Analysis in RA and Control Samples.

Low	High	padj	pvalue	log2FoldChange	immune_cell	sig
0.1445	0.1103	0.000780731244552939	0.000141951135373262	-0.034221791	CD8 T cells	CD8 T cells***
0.1212	0.1017	0.0114619404109064	0.00312598374842902	-0.019505471	Naive CD4 T cells	Naive CD4 T cells**
0.007036	0.0209	0.00359994856672123	0.00081817012880028	0.013861313857563	Resting CD4 memory T cells	Resting CD4 memory T cells***
0.003317	0.01813	3.26233580307503e-10	2.9657598209773e-11	0.0148151361410601	Activated CD4 memory T cells	Activated CD4 memory T cells****
0.005269	0.001153	2.06078176180453e-10	9.36718982638424e-12	-0.004116005	Regulatory T cells (Tregs)	Regulatory T cells (Tregs)****
0	0.001868	0.0522993101371543	0.0190179309589652	0.00186796621078107	Gamma delta T cells	Gamma delta T cells*
0.005563	0.005066	0.0522993101371543	0.0189077566490779	-0.000497663	Activated NK cells	Activated NK cells*
0.0004925	0.002922	1.6813701560855e-05	2.29277748557114e-06	0.0024290869097526	M2 Macrophages	M2 Macrophages****

S7 Table Detailed Statistical Results of Correlation Analysis between Biomarkers and Immune Cells.

	cell	Type	correlation	p
1	Resting CD4 memory T cells	COX7B	0.402806562007495	3.76234459276175e-12
2	Activated CD4 memory T cells	COX7B	0.514651641561629	5.32932787746532e-20
3	Regulatory T cells (Tregs)	COX7B	-0.463378031	4.81768769639054e-16
4	Gamma delta T cells	COX7B	0.384649113980484	3.96160706968748e-11
5	M2 Macrophages	COX7B	0.481049143177685	2.47535910632524e-17
1	Resting CD4 memory T cells	NDUFB3	0.3857984394769	3.42762200004272e-11
2	Activated CD4 memory T cells	NDUFB3	0.47818645530206	4.05109933121643e-17
3	Regulatory T cells (Tregs)	NDUFB3	-0.457717611	1.20308122890429e-15
4	Gamma delta T cells	NDUFB3	0.380813016346286	6.39700170238778e-11
5	M2 Macrophages	NDUFB3	0.363709487338974	5.02807086771835e-10
1	Resting CD4 memory T cells	UQCRQ	0.390139008616747	1.97382599922329e-11
2	Activated CD4 memory T cells	UQCRQ	0.503065118045347	4.78236088944152e-19
3	Regulatory T cells (Tregs)	UQCRQ	-0.402481847	3.9291188587435e-12
4	Gamma delta T cells	UQCRQ	0.379261813573885	7.7510644953712e-11
5	M2 Macrophages	UQCRQ	0.517106012670681	3.3120852391008e-20

S8 Table Regulatory Networks of Biomarkers with Transcription Factors (TFs), miRNAs, and lncRNAs.

mirna	mrna	mi_mrna
hsa-miR-106a-3p	UQCRQ	hsa-miR-106a-3p_UQCRQ
hsa-miR-520a-5p	UQCRQ	hsa-miR-520a-5p_UQCRQ
hsa-miR-106a-3p	UQCRQ	hsa-miR-106a-3p_UQCRQ
hsa-miR-520a-5p	UQCRQ	hsa-miR-520a-5p_UQCRQ
hsa-miR-525-5p	UQCRQ	hsa-miR-525-5p_UQCRQ
hsa-miR-520a-5p	UQCRQ	hsa-miR-520a-5p_UQCRQ
hsa-miR-497-3p	NDUFB3	hsa-miR-497-3p_NDUFB3
hsa-miR-511-5p	UQCRQ	hsa-miR-511-5p_UQCRQ
hsa-miR-511-5p	UQCRQ	hsa-miR-511-5p_UQCRQ
hsa-miR-144-3p	UQCRQ	hsa-miR-144-3p_UQCRQ
hsa-miR-139-5p	UQCRQ	hsa-miR-139-5p_UQCRQ
hsa-miR-139-5p	UQCRQ	hsa-miR-139-5p_UQCRQ
hsa-miR-630	NDUFB3	hsa-miR-630_NDUFB3
hsa-miR-485-3p	UQCRQ	hsa-miR-485-3p_UQCRQ
hsa-miR-10b-3p	UQCRQ	hsa-miR-10b-3p_UQCRQ
hsa-miR-323a-3p	UQCRQ	hsa-miR-323a-3p_UQCRQ
hsa-miR-323a-3p	UQCRQ	hsa-miR-323a-3p_UQCRQ
hsa-miR-548b-3p	UQCRQ	hsa-miR-548b-3p_UQCRQ
hsa-miR-525-5p	UQCRQ	hsa-miR-525-5p_UQCRQ
hsa-miR-342-5p	UQCRQ	hsa-miR-342-5p_UQCRQ
hsa-miR-518d-5p	UQCRQ	hsa-miR-518d-5p_UQCRQ
hsa-miR-518e-5p	UQCRQ	hsa-miR-518e-5p_UQCRQ
hsa-miR-148b-3p	UQCRQ	hsa-miR-148b-3p_UQCRQ
hsa-miR-148a-3p	UQCRQ	hsa-miR-148a-3p_UQCRQ
hsa-miR-511-5p	UQCRQ	hsa-miR-511-5p_UQCRQ
hsa-miR-518f-5p	UQCRQ	hsa-miR-518f-5p_UQCRQ
hsa-miR-376a-5p	UQCRQ	hsa-miR-376a-5p_UQCRQ
hsa-miR-525-5p	UQCRQ	hsa-miR-525-5p_UQCRQ
hsa-miR-139-5p	UQCRQ	hsa-miR-139-5p_UQCRQ
hsa-miR-17-3p	UQCRQ	hsa-miR-17-3p_UQCRQ
hsa-miR-522-3p	UQCRQ	hsa-miR-522-3p_UQCRQ
hsa-miR-591	UQCRQ	hsa-miR-591_UQCRQ
hsa-miR-411-5p	UQCRQ	hsa-miR-411-5p_UQCRQ
hsa-miR-552-3p	COX7B	hsa-miR-552-3p_COX7B
hsa-miR-10b-3p	UQCRQ	hsa-miR-10b-3p_UQCRQ
hsa-miR-323a-3p	UQCRQ	hsa-miR-323a-3p_UQCRQ
hsa-miR-345-5p	UQCRQ	hsa-miR-345-5p_UQCRQ
hsa-miR-345-5p	UQCRQ	hsa-miR-345-5p_UQCRQ
hsa-miR-200b-5p	NDUFB3	hsa-miR-200b-5p_NDUFB3
hsa-miR-708-3p	NDUFB3	hsa-miR-708-3p_NDUFB3
hsa-miR-514a-3p	COX7B	hsa-miR-514a-3p_COX7B

hsa-miR-539-5p	NDUFB3	hsa-miR-539-5p_NDUFB3
hsa-miR-380-3p	UQCRQ	hsa-miR-380-3p_UQCRQ
hsa-miR-518d-5p	UQCRQ	hsa-miR-518d-5p_UQCRQ
hsa-miR-518e-5p	UQCRQ	hsa-miR-518e-5p_UQCRQ
hsa-miR-628-5p	UQCRQ	hsa-miR-628-5p_UQCRQ
hsa-miR-566	COX7B	hsa-miR-566_COX7B
hsa-miR-768-5p	NDUFB3	hsa-miR-768-5p_NDUFB3
hsa-miR-125b-2-3p	NDUFB3	hsa-miR-125b-2-3p_NDUFB3
hsa-miR-376a-5p	UQCRQ	hsa-miR-376a-5p_UQCRQ
hsa-miR-518f-5p	UQCRQ	hsa-miR-518f-5p_UQCRQ
hsa-miR-634	UQCRQ	hsa-miR-634_UQCRQ
hsa-miR-591	UQCRQ	hsa-miR-591_UQCRQ
hsa-miR-17-3p	UQCRQ	hsa-miR-17-3p_UQCRQ
hsa-miR-889-3p	UQCRQ	hsa-miR-889-3p_UQCRQ
hsa-miR-591	UQCRQ	hsa-miR-591_UQCRQ
hsa-miR-599	UQCRQ	hsa-miR-599_UQCRQ
hsa-miR-599	UQCRQ	hsa-miR-599_UQCRQ
hsa-miR-193a-5p	UQCRQ	hsa-miR-193a-5p_UQCRQ
hsa-miR-345-5p	UQCRQ	hsa-miR-345-5p_UQCRQ
hsa-miR-187-5p	UQCRQ	hsa-miR-187-5p_UQCRQ
hsa-miR-625-3p	UQCRQ	hsa-miR-625-3p_UQCRQ
hsa-miR-493-5p	UQCRQ	hsa-miR-493-5p_UQCRQ
hsa-miR-152-3p	UQCRQ	hsa-miR-152-3p_UQCRQ
hsa-miR-379-5p	NDUFB3	hsa-miR-379-5p_NDUFB3
hsa-miR-518d-5p	UQCRQ	hsa-miR-518d-5p_UQCRQ
hsa-miR-139-3p	UQCRQ	hsa-miR-139-3p_UQCRQ
hsa-miR-200a-5p	NDUFB3	hsa-miR-200a-5p_NDUFB3
hsa-miR-135b-3p	NDUFB3	hsa-miR-135b-3p_NDUFB3
hsa-miR-331-5p	NDUFB3	hsa-miR-331-5p_NDUFB3
hsa-miR-518e-5p	UQCRQ	hsa-miR-518e-5p_UQCRQ
hsa-miR-139-3p	UQCRQ	hsa-miR-139-3p_UQCRQ
hsa-miR-518f-5p	UQCRQ	hsa-miR-518f-5p_UQCRQ
hsa-miR-628-5p	UQCRQ	hsa-miR-628-5p_UQCRQ
hsa-miR-589-5p	UQCRQ	hsa-miR-589-5p_UQCRQ
hsa-miR-451a	UQCRQ	hsa-miR-451a_UQCRQ
hsa-miR-190a-5p	NDUFB3	hsa-miR-190a-5p_NDUFB3

S9 Table List of Mitochondrial Metabolism-Related Genes (MMRGs) Used in the Study.

id
ADH5
AFG1L
AIFM2
AKR1A1
AKR1B1
AKR1C4
AKR7A2
AKR7A3
ALDH2
ALDH4A1
AOC2
AOX1
ASPH
BID
C15orf48
CCNB1
CDK1
CHCHD2
CIAPIN1
COA6
COQ9
COX10
COX11
COX15
COX4I1
COX4I2
COX5A
COX5B
COX6A1
COX6A2
COX6B1
COX6C
COX7A1
COX7A2
COX7A2L
COX7A2P2
COX7B
COX7B2
COX7C
COX8A
COX8C
CYB561

CYB5A
CYB5B
CYBA
CYBB
CYC1
CYCS
CYP19A1
CYP1A2
DEGS1
DGUOK
DHDH
DHRS3
DLD
DMGDH
DNAJC15
ETFA
ETFB
ETFDH
ETFRF1
FDX1
FDX2
GFUS
GHITM
GLDC
GLRX
GLRX2
GPD1
GPD2
GPX2
GSR
HAAO
HSD17B6
IDH3B
IDO1
IMMP2L
ISCU
LOXL2
MAOB
ME1
ME2
MIR210
MT-CO1
MT-CO2
MT-CO3

MT-CYB
MT-ND1
MT-ND2
MT-ND3
MT-ND4
MT-ND4L
MT-ND5
MT-ND6
MTCO2P12
MYBBP1A
NCF1
NCF2
NDUFA1
NDUFA10
NDUFA11
NDUFA12
NDUFA13
NDUFA2
NDUFA3
NDUFA4
NDUFA4L2
NDUFA5
NDUFA6
NDUFA7
NDUFA8
NDUFA9
NDUFAB1
NDUFAF1
NDUFAF2
NDUFB1
NDUFB10
NDUFB11
NDUFB2
NDUFB3
NDUFB4
NDUFB5
NDUFB6
NDUFB7
NDUFB8
NDUFB9
NDUFC1
NDUFC2
NDUFC2-KCTD14
NDUFS1

NDUFS2
NDUFS3
NDUFS4
NDUFS5
NDUFS6
NDUFS7
NDUFS8
NDUFV1
NDUFV2
NDUFV3
NOX4
NQO2
P4HA2
PARK7
PHGDH
PINK1
POLG2
PPARGC1A
QDPR
RDH16
SCO2
SDHA
SDHAF2
SDHB
SDHC
SDHD
SLC25A12
SLC25A13
SLC25A18
SLC25A22
SNCA
SRD5A1
STEAP4
SURF1
TAZ
UQCC3
UQCR10
UQCR11
UQCRB
UQCRC1
UQCRC2
UQCRFS1
UQCRFS1P1
UQCRH

UQCRHL
UQCRQ
WDR93
XDH
ATP5F1A
ATP5F1B
ATP5F1C
ATP5F1D
ATP5F1E
ATP5IF1
ATP5MC1
ATP5MC2
ATP5MC3
ATP5ME
ATP5MF
ATP5MG
ATP5PB
ATP5PD
ATP5PF
ATP5PO
COX17
DMAC2L
MIR4691
MIR7113
MT-ATP6
MT-ATP8
SCO1
SLC25A14
SLC25A27
SLC25A4
SLC25A5
SLC25A6
SNORD138
UCP1
UCP2
UCP3
ACO2
CS
FH
IDH2
IDH3A
IDH3G
MDH2
OGDH

RPL18AP16
SUCLA2
SUCLG1
DLST
FAHD1
ME3
NNT
SUCLG2
ACLY
ACO1
ACOT4
GAD1
GAD2
IDH1
MDH1
ACAA1
ACADM
ACSS1
ADH1C
ADH4
ADH7
ALDH18A1
ALDH1A1
ALDH7A1
AOC3
ARG1
ARG2
ASNS
ASS1
AUH
BCAT1
BHMT
CAD
CBS
CPS1
CTH
DBH
DDC
EHHADH
EPRS1
FAH
FARSB
FTCD
G6PC2

GCLM
GLS
GLUD1
GLUL
GOT1
GOT2
GPT2
GSS
HADH
HAL
HDC
HIBADH
HIBCH
HMGCL
HMGCS2
HNMT
IARS1
LARS2
LDHA
MAOA
MARS2
MCCC1
MMUT
MPST
OAT
ODC1
OTC
PC
PCK1
PDHA1
PDHX
PDK4
PKM
PNMT
PPM1L
PYCR1
RARS1
SDS
SMS
SRM
TAT
TDO2
TH
TPH1

TPO
VARS1
WARS1
ACAA2
ACADL
ACADS
ACADSB
ACADVL
ACAT1
ACAT2
ACOX1
ACOX3
ACSL1
ACSL3
ACSL4
ACSL5
ACSL6
ADH1A
ADH1B
ADH6
ALDH1B1
ALDH3A2
ALDH9A1
CPT1A
CPT1B
CPT1C
CPT2
CYP4A11
CYP4A22
ECHS1
ECI1
ECI2
GCDH
HADHA
HADHB
AGK
AGPAT1
AGPAT2
AGPAT3
AGPAT4
AWAT2
CEL
DGAT1
DGAT2

DGKA
DGKB
DGKD
DGKE
DGKG
DGKH
DGKI
DGKQ
DGKZ
GK
GK2
GLA
GLYCTK
GPAM
GPAT2
GPAT3
GPAT4
LCLAT1
LIPC
LIPF
LIPG
LPL
MBOAT1
MBOAT2
MGLL
PLPP1
PLPP2
PLPP3
PNLIP
PNLIPRP1
PNLIPRP2
PNPLA3
TKFC
ACHE
ADPRM
CDIPT
CDS1
CDS2
CHAT
CHKA
CHKB
CHPT1
CRLS1
ETNK1

ETNK2
GNPAT
GPD1L
JMJD7-PLA2G4B
LCAT
LPCAT1
LPCAT2
LPCAT3
LPCAT4
LPGAT1
LYPLA1
LYPLA2
MBOAT7
PCYT1A
PCYT1B
PCYT2
PEMT
PGS1
PHOSPHO1
PISD
PLA2G10
PLA2G12A
PLA2G12B
PLA2G15
PLA2G1B
PLA2G2A
PLA2G2C
PLA2G2D
PLA2G2E
PLA2G2F
PLA2G3
PLA2G4A
PLA2G4B
PLA2G4E
PLA2G5
PLA2G6
PLD1
PLD2
PTDSS1
PTDSS2
ABCC1
ABCD1
ACACA
ACACB

ACAD10
ACAD11
ACBD4
ACBD5
ACBD6
ACBD7
ACOT1
ACOT11
ACOT12
ACOT13
ACOT2
ACOT6
ACOT7
ACOT8
ACOT9
ACOX2
ACOXL
ACSBG1
ACSBG2
ACSF2
ACSF3
ACSM3
ACSM6
AKR1C3
ALOX12
ALOX12B
ALOX15
ALOX15B
ALOX5
ALOX5AP
ALOXE3
AMACR
AWAT1
CBR1
CBR4
CRAT
CROT
CYP1A1
CYP1B1
CYP2C19
CYP2C8
CYP2C9
CYP2J2
CYP2U1

CYP4B1
CYP4F11
CYP4F2
CYP4F22
CYP4F3
CYP4F8
CYP8B1
DBI
DECR1
DECR2
DPEP1
DPEP2
DPEP3
ELOVL1
ELOVL2
ELOVL3
ELOVL4
ELOVL5
ELOVL6
ELOVL7
EPHX2
FAAH
FAAH2
FADS1
FADS2
FASN
GGT1
GGT5
GPX1
GPX4
HACD1
HACD2
HACD3
HACD4
HACL1
HAO2
HPGD
HPGDS
HSD17B12
HSD17B3
HSD17B4
HSD17B8
HTD2
LTA4H

LTC4S
MAPKAPK2
MCAT
MCEE
MECR
MID1IP1
MLYCD
MMAA
MORC2
NUDT19
NUDT7
OLAH
PCCA
PCCB
PCTP
PECR
PHYH
PON1
PON2
PON3
PPARD
PPT1
PPT2
PRKAA2
PRKAB2
PRKAG2
PRXL2B
PTGDS
PTGES
PTGES2
PTGES3
PTGIS
PTGR1
PTGR2
PTGS1
PTGS2
RXRA
SCD
SCD5
SCP2
SLC22A5
SLC25A1
SLC25A17
SLC25A20

SLC27A2
SLC27A3
TBXAS1
TECR
TECRL
THEM4
THEM5
THRSP
ABHD5
AGMO
CAV1
FABP1
FABP12
FABP2
FABP3
FABP4
FABP5
FABP6
FABP7
FABP9
GK3P
LIPE
LPIN1
LPIN2
LPIN3
MOGAT1
MOGAT2
MOGAT3
PLIN1
PLIN3
PNPLA4
PNPLA5
PPP1CA
PPP1CB
PPP1CC
PRKACA
PRKACB
PRKACG
AACS
ABCA1
ABCB11
ABCB4
ABCC3
ABHD3

ABHD4
ACER1
ACER2
ACER3
ACP6
ACSS3
AGPAT5
AGPS
AGT
AHR
AHRR
AKR1B15
AKR1C1
AKR1C2
AKR1D1
ALAS1
ALB
ALDH3B1
ALDH3B2
ALPI
ANGPTL4
ANKRD1
APOA1
APOA2
APOA5
ARF1
ARF3
ARNT
ARNT2
ARNTL
ARSA
ARSB
ARSD
ARSF
ARSG
ARSH
ARSI
ARSJ
ARSK
ARSL
ARV1
ASAH1
ASAH2
B3GALNT1

B4GALNT1
BAAT
BCHE
BDH1
BDH2
BMX
CARM1
CCNC
CD36
CDK19
CDK8
CEPT1
CERK
CERS1
CERS2
CERS3
CERS4
CERS5
CERS6
CERT1
CGA
CH25H
CHD9
CIDEA
CIDEC
CLOCK
CPNE1
CPNE3
CPNE6
CPNE7
CPTP
CREBBP
CSNK1G2
CSNK2A1
CSNK2A2
CSNK2B
CTSA
CUBN
CYP11A1
CYP11B1
CYP11B2
CYP17A1
CYP21A2
CYP24A1

CYP27A1
CYP27B1
CYP2D6
CYP2E1
CYP2R1
CYP39A1
CYP3A4
CYP46A1
CYP51A1
CYP7A1
CYP7B1
DDHD1
DDHD2
DEGS2
DGAT2L6
DHCR24
DHCR7
DHRS7B
EBP
ENPP6
ENPP7
EP300
ESRRA
ESYT1
ESYT2
ESYT3
ETNPPL
FA2H
FAM120B
FAR1
FAR2
FDFT1
FDPS
FDXR
FHL2
FIG4
FITM1
FITM2
G0S2
GALC
GBA
GBA2
GC
GDE1

GDPD1
GDPD3
GDPD5
GGPS1
GLB1
GLB1L
GLIPR1
GLTP
GM2A
GPCPD1
GPS2
GRHL1
GSTM4
HDAC3
HELZ2
HEXA
HEXB
HILPDA
HMGCLL1
HMGCR
HMGCS1
HSD11B1
HSD11B2
HSD17B1
HSD17B11
HSD17B13
HSD17B14
HSD17B2
HSD17B7
HSD3B1
HSD3B2
HSD3B7
IDI1
IDI2
INPP4A
INPP4B
INPP5D
INPP5E
INPP5F
INPP5J
INPP5K
INPPL1
INSIG1
INSIG2

KDSR
KPNB1
LBR
LGMN
LHB
LIPH
LIPI
LRP2
LSS
MBTPS1
MBTPS2
MED1
MED10
MED11
MED12
MED13
MED13L
MED14
MED15
MED16
MED17
MED18
MED19
MED20
MED21
MED22
MED23
MED24
MED25
MED26
MED27
MED28
MED29
MED30
MED31
MED4
MED6
MED7
MED8
MED9
MFSD2A
MIGA1
MIGA2
MSMO1

MTF1
MTM1
MTMR1
MTMR10
MTMR12
MTMR14
MTMR2
MTMR3
MTMR4
MTMR6
MTMR7
MTMR8
MTMR9
MVD
MVK
NCOA1
NCOA2
NCOA3
NCOA6
NCOR1
NCOR2
NEU1
NEU2
NEU3
NEU4
NFYA
NFYB
NFYC
NPAS2
NR1D1
NR1H2
NR1H3
NR1H4
NRF1
NSDHL
OCRL
ORMDL1
ORMDL2
ORMDL3
OSBP
OSBPL10
OSBPL1A
OSBPL2
OSBPL3

OSBPL5
OSBPL6
OSBPL7
OSBPL8
OSBPL9
OXCT1
OXCT2
PDCD6-AHRR
PEX11A
PI4K2A
PI4K2B
PI4KA
PI4KB
PIAS4
PIK3C2A
PIK3C2B
PIK3C2G
PIK3C3
PIK3CA
PIK3CB
PIK3CD
PIK3CG
PIK3R1
PIK3R2
PIK3R3
PIK3R4
PIK3R5
PIK3R6
PIKFYVE
PIP4K2A
PIP4K2B
PIP4K2C
PIP4P1
PIP5K1A
PIP5K1B
PIP5K1C
PITPNB
PITPNM1
PITPNM2
PITPNM3
PLA1A
PLA2G4C
PLA2G4D
PLA2G4F

PLA2R1
PLAAT1
PLAAT2
PLAAT3
PLAAT4
PLAAT5
PLB1
PLBD1
PLD3
PLD4
PLD6
PLEKHA1
PLEKHA2
PLEKHA3
PLEKHA4
PLEKHA5
PLEKHA6
PLEKHA8
PLIN2
PLPP6
PMVK
PNPLA2
PNPLA6
PNPLA7
PNPLA8
POMC
PPARA
PPARG
PPARGC1B
PRKD1
PRKD2
PRKD3
PSAP
PTEN
PTPMT1
PTPN13
RAB14
RAB4A
RAB5A
RAN
RGL1
RORA
RUFY1
RXRB

SACM1L
SAMD8
SAR1B
SBF1
SBF2
SC5D
SCAP
SEC23A
SEC24A
SEC24B
SEC24C
SEC24D
SELENOI
SERPINA6
SGMS1
SGMS2
SGPL1
SGPP1
SGPP2
SIN3A
SIN3B
SLC10A1
SLC10A2
SLC27A1
SLC27A5
SLC44A1
SLC44A2
SLC44A3
SLC44A4
SLC44A5
SLCO1A2
SLCO1B1
SLCO1B3
SMARCD3
SMPD1
SMPD2
SMPD3
SMPD4
SP1
SPHK1
SPHK2
SPNS2
SPTLC1
SPTLC2

SPTLC3
SPTSSA
SPTSSB
SQLE
SRD5A2
SRD5A3
SREBF1
SREBF2
STAR
STARD10
STARD3
STARD3NL
STARD4
STARD5
STARD6
STARD7
STS
SULT2A1
SUMF1
SUMF2
SUMO2
SYNJ1
SYNJ2
TBL1X
TBL1XR1
TGS1
THRAP3
TIAM2
TM7SF2
TMEM86B
TNFAIP8
TNFAIP8L1
TNFAIP8L2
TNFAIP8L3
TNFRSF21
TPTE
TPTE2
TRIB3
TSPO
TSPOAP1
TXNRD1
UBE2I
UGCG
UGT1A9

UGT8
VAC14
VAPA
VAPB
VDR
ADSL
AHCYL2
ALG3
ALMS1
ANKEF1
ARHGAP11A
BYSL
CABYR
CD70
CDC45
CENPF
DDX21
DLEU2
DONSON
E2F1
GMNN
GPSM2
HDAC2
HLCS
HPRT1
IER3
IFRD2
ILF3
KCNN4
MAS1L
MDC1
MDM1
METAP1
MRTO4
NCAPD2
NME1
NME2
NUP155
NUP205
NUP50
NUP88
ORC1
PADI3
PLGRKT

PLK4
PMCHL1
POLA1
PRPF38A
PRPS2
RAB6B
RFPL3S
RRM1
RRM2
SAC3D1
SEMA4B
SLC25A37
SLC30A10
SNRPA
SPEF2
SRPK1
UCK2
UTP18
ZBTB5
ZIC2
ZNF490
ZNF706
ADA
ADAL
ADK
ADSS1
ADSS2
AGXT2
AK1
AK2
AK4
AK5
AK6
AK7
AK8
AK9
AMPD1
AMPD2
AMPD3
APRT
ATIC
CDA
CMPK1
CTPS1

CTPS2
DCK
DCTD
DCTPP1
DHODH
DNPH1
DPYD
DPYS
DTYMK
DUT
ENTPD1
ENTPD2
ENTPD3
ENTPD4
ENTPD5
ENTPD6
ENTPD7
ENTPD8
GART
GDA
GMPR
GMPR2
GMPS
GUK1
IMPDH1
IMPDH2
ITPA
LHPP
NME3
NME4
NT5C
NT5C1A
NT5C1B
NT5C2
NT5C3A
NT5E
NT5M
NUDT1
NUDT13
NUDT15
NUDT16
NUDT18
NUDT5
NUDT9

PAICS
PFAS
PNP
PPAT
PUDP
RRM2B
SAMHD1
TK1
TK2
TXN
TYMP
TYMS
UCK1
UCKL1
UMPS
UPB1
UPP1
UPP2
AHCY
ALDH1L1
AMT
CHDH
DHFR
DNMT1
DNMT3A
DNMT3B
FOLH1
FOLH1B
MAT1A
MAT2B
MIR6778
MTFMT
MTHFD1
MTHFD1L
MTHFD2
MTHFR
MTHFS
MTR
MTRR
SHMT1
SHMT2
TCN2
OAZ1
POLB

POLD1
POLG
PRPS1
SAT1

S10 Table Primer Sequences Used for qPCR Validation of Biomarkers.

**Steps**	**Reaction**	**Cycle times**	**Temperature**	**Time**
Stage 1	Predenaturation	Reps:1	95 °C	5 min
Stage 2	Cyclic reaction	Reps:40	95 °C	10 s
			60 °C	30 s
Stage 3	Melting curve	Reps:1	95 °C	15 s
			60 °C	60 s
			95 °C	15 s

S11 Table qPCR Validation Experimental Steps and Parameters.

Gene	Sense/antisense primer	Sequence（5′-3′)
GAPDH	forward	GGAGCGAGATCCCTCCAAAAT
	reverse	GGCTGTTGTCATACTTCTCATGG
COX7B	forward	GCCTCCCGAGTAGCCAGAAC
	reverse	GCCGAGATCACGCCATTGC
NDUFB3	forward	GTCAGGCAGAAGAACAGCATCAC
	reverse	GGTGTCCCTTCTATCTTCCATTGTC
UQCRQ	forward	AGGACTGCGGTTGGACAAGC
	reverse	ACTGCTGTGGAGGCTAAGAGATC
