# Supplementary material for: Identification of mitochondria metabolism-related biomarkers associated with the development of rheumatoid arthritis using bioinformatics: An observational study
Source: Medicine (Baltimore). 2026 Jan 9;105(2):e44435. doi: 10.1097/MD.0000000000044435 (PMC12795057; doi:10.1097/MD.0000000000044435)

**Fig. S1 DEGs screening validation and association analysis with MMRGs.** **(a)** Volcano plot of differentially expressed genes (DEGs) between the RA group and the control group in the GSE15573 dataset, where red dots represent genes significantly upregulated in the RA group compared to the control group, and blue dots represent genes significantly downregulated in the RA group. **(b**) Heatmap of the top 10 upregulated and downregulated genes between the RA group and the control group in the GSE15573 dataset. **(c)** Venn diagram showing the intersection of differentially expressed genes (DEGs) and mitochondrial metabolism-related genes (MMRGs) in the GSE15573 dataset, with the overlapping region representing candidate genes shared by the two groups.


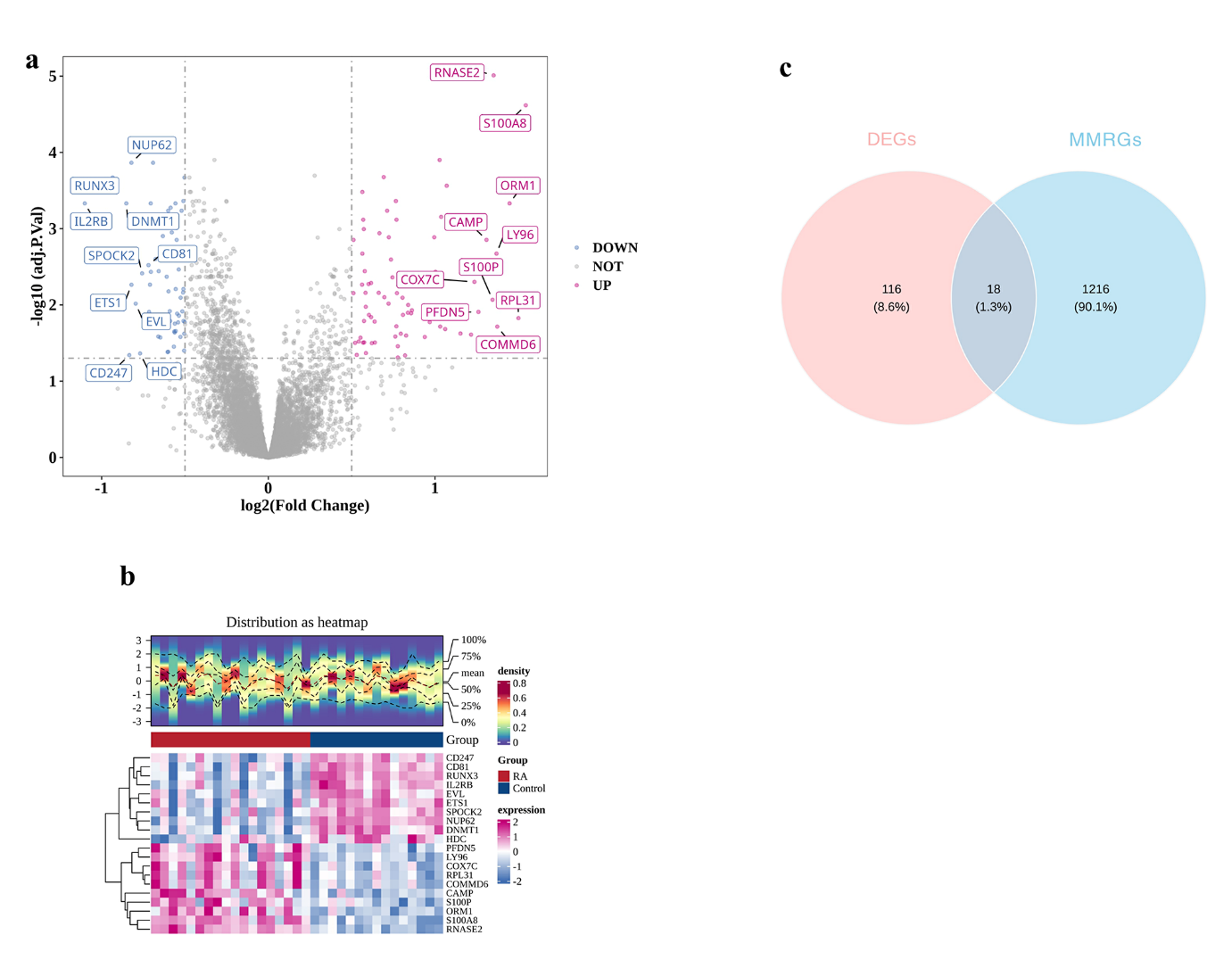


**Fig. S2 Analysis of Expression Trends of Differentially Expressed Genes (DEGs).** **(a)** Venn Diagram of the Intersection of Differentially Expressed Genes between GSE93272 and GSE15573. (DEGs1: differentially expressed genes in the training set, DEGs2: differentially expressed genes in the validation set). **(b)** Box Plot of Expression Trends of Intersected Differential Genes in the GSE93272 Dataset. **(c)** Box Plot of Expression Trends of Intersected Differential Genes in the GSE15573 Dataset


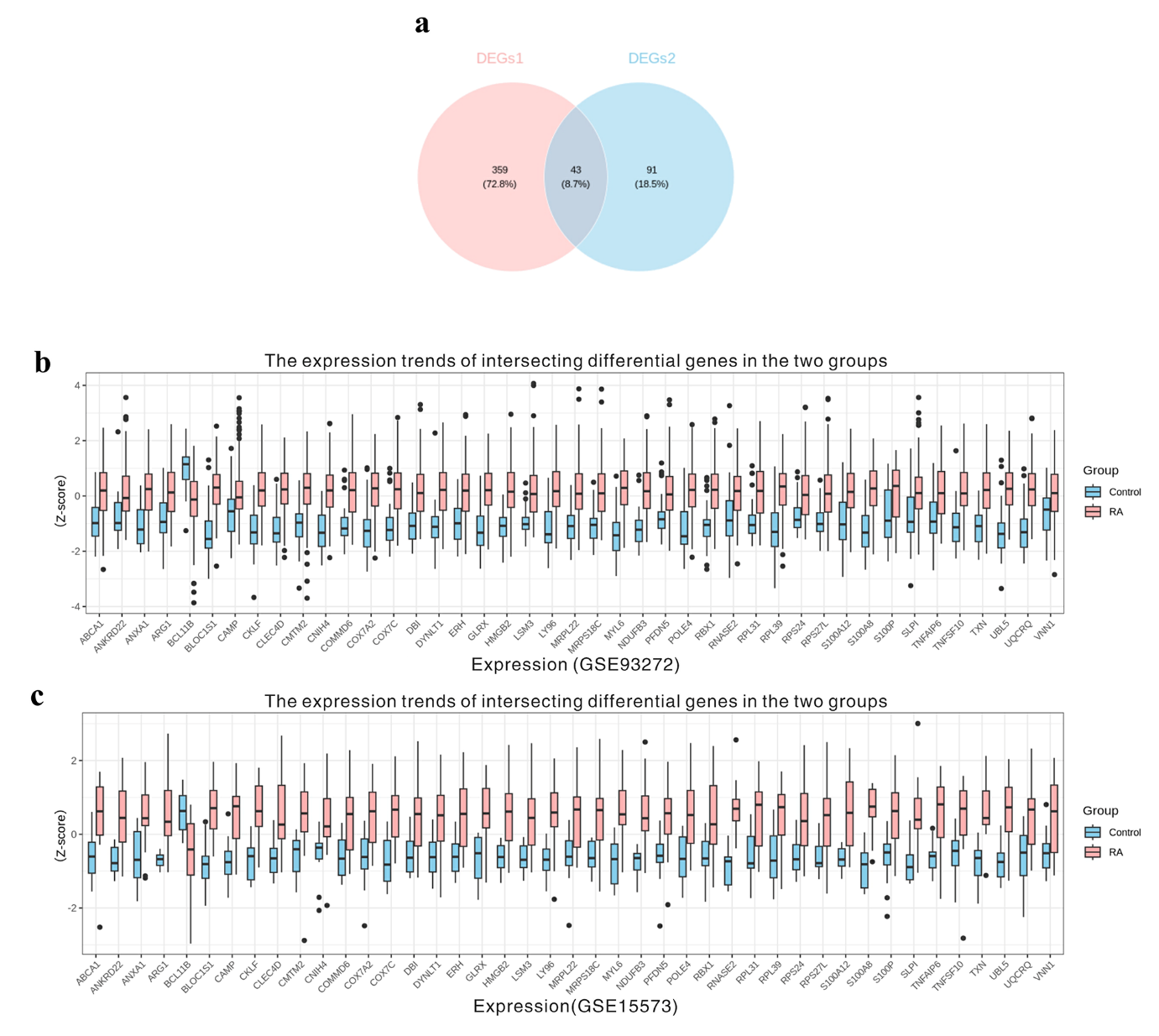


**Fig. S3 Analysis and validation of protein-protein interaction networks of rheumatoid arthritis-related core genes in the GSE15573 dataset.** **(a)** Protein-protein interaction (PPI) network of candidate genes in the GSE15573 dataset. **(b)** The top 10 core genes selected based on Degree scores as candidate biomarkers


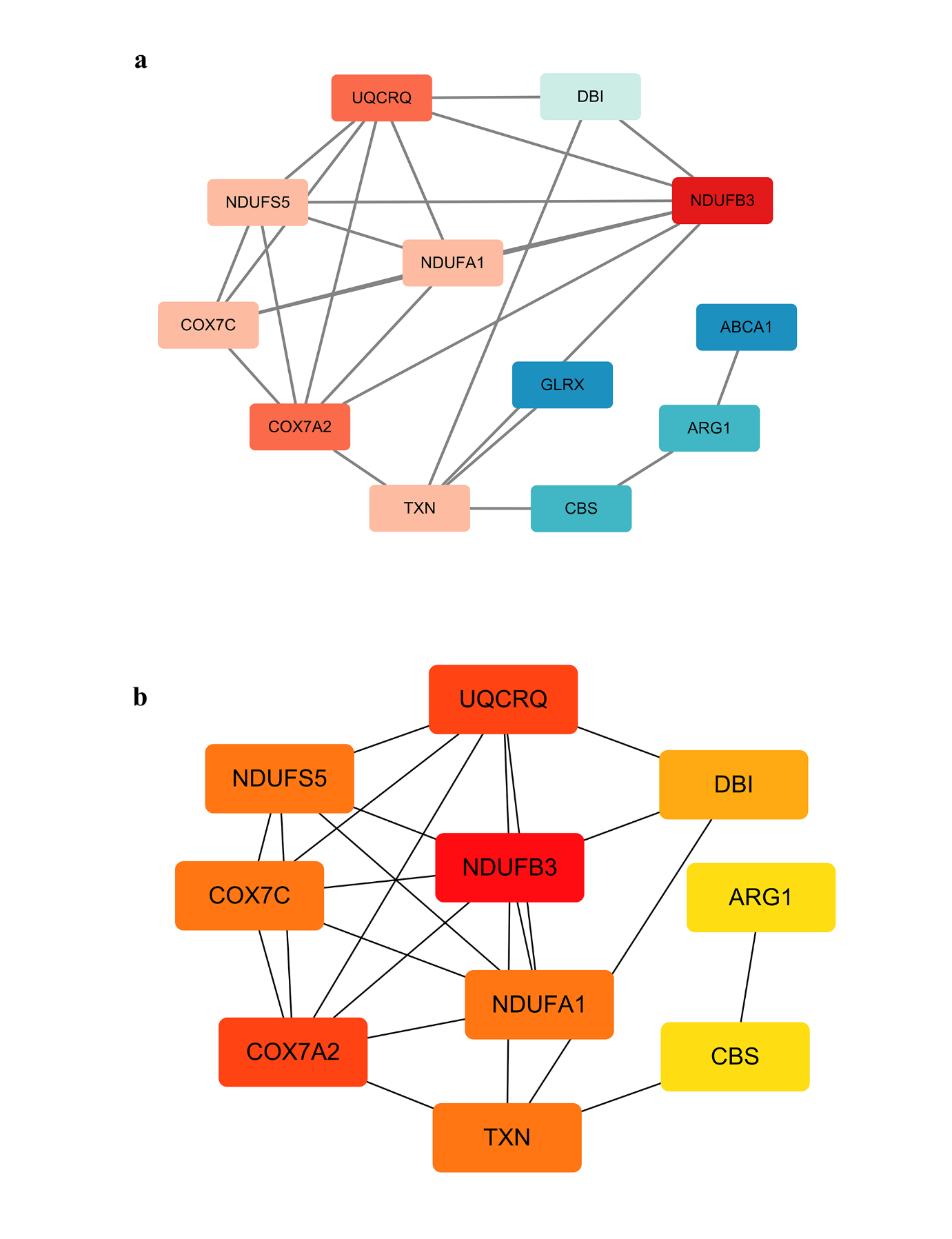


**Fig. S4 Validation set prediction model based on the same biomarkers and its efficacy evaluation. (a)** Construction of the RA risk prediction nomogram in GSE15573: The RA risk prediction nomogram was constructed based on the same biomarkers (COX7B, NDUFB3, and UQCRQ). **(b)** Calibration curve of the validation set prediction model: The thick gray line represents the reference line where the predicted disease probability is completely consistent with the actual disease probability. The dashed line indicates the predicted probability of the nomogram, and the solid black line represents the perfect prediction after bias correction via Bootstrapping (1000 repetitions). **(c)** ROC curve of the validation set prediction modelSupplementary.


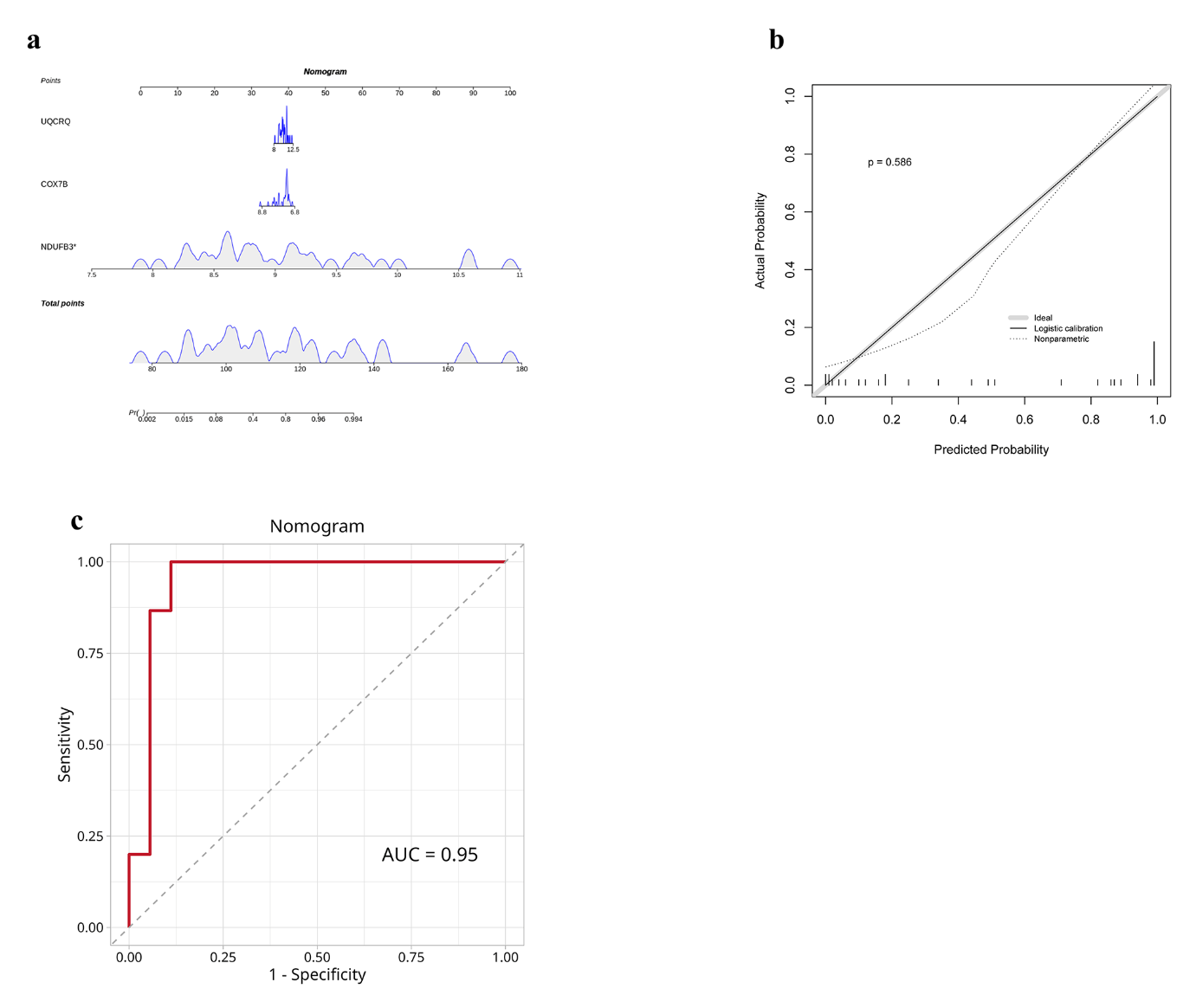

Supplement: Supplementary file 2 [file medi-105-e44435-s002.docx]
